# Global, regional, and national levels and trends in under 5, infant, and neonatal mortality during 1990-2024 with scenario based projections to 2030: modelling study

**DOI:** 10.1136/bmj-2025-088684

**Published:** 2026-06-04

**Authors:** David Sharrow, Lucia Hug, Yang Liu, Graeme Wilson Fell, Danzhen You

**Affiliations:** 1 Data and Analytics Section, UNICEF Office of Strategy and Evidence–Innocenti, Florence, Italy; 2 Data and Analytics Section, UNICEF Office of Strategy and Evidence–Innocenti, UNICEF Headquarters, New York, NY 10017, USA

## Abstract

**Objective:**

To estimate all cause neonatal, infant, and under 5 mortality for 200 countries and areas in 1990-2024; assess levels and trends to identify where mortality declines have slowed or accelerated and which countries risk missing international child survival targets; and project possible numbers of deaths during 2025-30 under scenarios of accelerating, decelerating, or stagnating mortality trends.

**Design:**

Modelling study.

**Data sources:**

Country specific household surveys, vital registration and sample vital registration systems, UNAIDS, CRED International Disaster Database, Uppsala Conflict Data Program/PRI Oslo datasets, the Armed Conflict Location and Event Data Project, the Center for Systemic Peace/Integrated Network for Societal Conflict Research, UN and other organisational crisis reports, and the World Population Prospects 2024 revision.

**Inclusion criteria:**

Nationally representative data. Surveys were excluded from estimation if data quality concerns or crisis related fieldwork disruptions were documented or suspected. Vital registration data needed to include at least 80% population coverage.

**Results:**

An estimated 4.9 million (90% uncertainty interval 4.7 to 5.2 million) children died before age 5 years in 2024, including 2.3 million (2.1 to 2.5 million) neonatal deaths. Mortality was highest in sub-Saharan Africa and South Asia, where uncertainty intervals were widest due to data sparsity and quality. The rate of decline was slower for neonates compared with children aged 1-59 months: the global neonatal mortality rate declined by 1.3% (0.5% to 1.9%) annually during 2015-24, compared with 1.7% (0.8% to 2.3%) for children aged 1-59 months. Progress has slowed significantly since 2015, with the global mortality rate in under 5s declining by 3.9% (3.7% to 4.0%) annually during 2000-15 versus 1.5% (0.8% to 1.9%) during 2015-24. Based on recent trends, 60 countries will not meet the sustainable development goal target for under 5 mortality, and 66 will not meet the neonatal mortality target. If recent trends continue, 27.3 million deaths in under 5s are projected between 2025 and 2030, nearly half in the neonatal period and predominantly in sub-Saharan Africa and South Asia.

**Conclusion:**

Millions of preventable deaths occur in children each year, and progress in reducing child mortality has slowed since 2015. Achieving global targets requires renewed commitment and sustained investment across the continuum of care, with emphasis on the neonatal period and regions with high mortality. Faster progress could avert millions of deaths, whereas stagnation or slower progress could result in substantially higher numbers of deaths. Strengthening data systems remains essential for tracking progress and guiding policy.

## Introduction

For almost five decades, child survival has remained central to the global health and development agenda. Beginning in the early 1980s with the “child survival revolution,” substantial declines in child mortality occurred in many low and middle income countries driven by high impact interventions such as immunisation, oral rehydration therapy, and improved nutrition.^1^ ^2^ The millennium development goals, adopted in 2000, called for a two thirds reduction in the under 5 mortality rate (U5MR) between 1990 and 2015.^3^ Owing to immense mobilisation of resources, political attention, and technical collaboration, child survival substantially improved over that 15 year period, with the global U5MR falling by more than half.^4^ The sustainable development goals, launched in 2015, set new targets for all countries to reduce U5MR to 25 deaths per 1000 live births or lower and the neonatal mortality rate to 12 deaths per 1000 live births or lower by 2030.^5^

Monitoring progress towards these goals requires systematic, timely, and comparable estimates of child mortality that enable the assessment of trends, evaluation of programmes, identification of high risk populations, and targeting of resources to those settings with the greatest need. Yet many countries, particularly those with the highest burden of child mortality, lack a single, reliable source of accurate and timely mortality data, making global estimation efforts essential for monitoring progress.

The United Nations Inter-agency Group for Child Mortality Estimation (UN IGME) was established in 2004 to produce annual estimates of child mortality for monitoring progress towards these goals. Comprising Unicef, the World Health Organization (WHO), the UN Population Division, and the World Bank Group, the UN IGME also works to advance estimation methods, assess and improve data quality, strengthen national data systems and country capacity, and enhance the global evidence base. It is the only organisation that updates estimates of child mortality annually in consultation with national governments.

This study provides updated estimates of levels and trends in under 5 mortality for 200 countries and areas from 1990 to 2024. We analysed global, regional, and national trends over the past three decades, assessed the progress of countries towards reaching the child survival targets of the sustainable development goals, and provide scenario based projections to estimate the potential burden of deaths in under 5s during 2025-30. These findings provide governments, researchers, and global health practitioners with updated global child mortality estimates and an assessment of where progress is accelerating, slowing, or at risk of falling short of global child survival goals.

## Methods

### Estimation of mortality in under 5s

**Fig 1:**
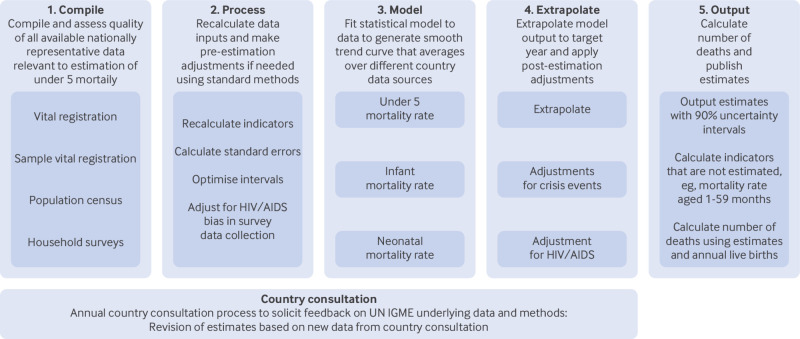
United Nations Inter-agency Group for Child Mortality Estimation (UN IGME) process for estimation of under 5 mortality rate, infant mortality rate, and neonatal mortality rate

We estimated three mortality rates: the U5MR, defined as the probability that a newborn dies before age 5 years; the infant mortality rate, defined as the probability that a newborn dies before age 1 year; and the neonatal mortality rate, defined as the probability a newborn dies before age 28 days—all expressed as deaths per 1000 live births. Mortality rates for other ages can be calculated from these estimated rates, such as the mortality rate for children aged 1-59 months (see supplementary appendix section 1.10 for more details). Following the strategy outlined in figure 1, estimates were produced by the UN IGME, with support from an independent technical advisory group composed of scholars and experts in demography, biostatistics, mortality data collection, and related disciplines that provides guidance on methodological approaches, analytical strategies, and data quality assessments. The supplementary appendix provides a more detailed description of the complete estimation process.

The estimation process begins by compiling and assessing the quality of all available nationally representative mortality data derived from vital registration and sample vital registration systems, population censuses, and household surveys. Data can be drawn directly from reports, or when microdata are available, data are processed using standard demographic methods,^6^ recalculating mortality indicators over shorter or longer intervals where appropriate,^7^ ^8^ assigning calendar year reference points,^8^ adding standard errors,^7^ ^8^ and adjusting for bias in data collection due to HIV/AIDS.^9^ ^10^ Because these sources may be affected by sampling and non-sampling errors, data quality is assessed before data are included as input for estimation. Information on survey quality, such as response rates and other methodological indicators, is drawn from survey and census reports as well as survey datasets when available. Vital registration data are assessed in terms of coverage and completeness.

As of March 2026, the UN IGME databases on the U5MR and infant mortality rate contained more than 23 000 country-year data points each, drawn from more than 1800 series across 200 countries and areas, with the earliest observation dating to 1912 and the most recent to 2024. The neonatal mortality rate database contains more than 8600 observations from 800 series. The database contains fewer observations and series than the U5MR and infant mortality rate databases because the neonatal mortality rate cannot be estimated from summary birth histories using indirect methods. A “series” refers to all observations derived from a single survey, census, vital registration source, or sample vital registration source.

The neonatal mortality rate, infant mortality rate, and U5MR databases are publicly available from the UN IGME web portal, www.childmortality.org (see supplementary appendix section 3.7 for list of sources by country).

### Modelling mortality rates in under 5s

Once data have been compiled, processed, and assessed for quality, a statistical model is fitted to these empirical data to produce a smooth mortality trend curve for each country that averages through possibly disparate estimates from different data sources.

Estimation of the U5MR and infant mortality rate was undertaken using the bayesian B splines bias adjusted model,^7^ known as the B3, which has been used by the UN IGME for annual estimation since 2014. In this model, the logarithm of the true mortality rate is estimated using a flexible spline regression fitted to all empirical observations of mortality rate for a country. Observed U5MR or infant mortality rate is conceptualised as the true mortality rate multiplied by an error multiplier that captures the relative difference between the observation and the unknown true value. No covariates are used in the estimation.

In estimating the true mortality rate, the data model incorporates information on the properties of each observation, including its standard error, source type, survey programme, and proximity to other observations in overlapping time periods. These characteristics inform adjustments for errors in observations, including the average systematic biases associated with different types of data sources. This approach ensures that the fitted mortality trends reflect not only empirical observations but also their systematic data quality.

Neonatal mortality rates were estimated using a similar bayesian framework that models the ratio of the neonatal mortality rate to the difference between the neonatal mortality rate and U5MR in each country-year.^11^ For each country-year, the ratio is estimated as the product of the expected ratio—derived from a global relation between observed ratios and the UN IGME estimated U5MR—and a country specific multiplier—modelled based on observed data—that captures deviations from the expected ratio over time. Seven small countries (Anguilla, British Virgin Islands, Micronesia, Monaco, Montserrat, Nauru, and Palau) do not have empirical data on the neonatal mortality rate (all countries have infant mortality rate and U5MR data), therefore neonatal mortality rate estimates are derived from the expected ratio. Supplementary appendix sections 1.3-1.6 provide additional details on the modelling approaches.

All estimates are presented with 90% uncertainty intervals (UIs) derived from the posterior distribution of mortality rate trajectories. Given the inherent uncertainty in neonatal mortality rate and U5MR estimates,^7^ we report 90% UIs to balance the need to reflect statistical uncertainty with the need for estimates that are interpretable and useful for policy and programmatic planning. Using 95% UIs would widen the intervals, particularly for data sparse countries and, in some cases, make them less informative for prioritising health interventions, whereas a narrower interval may not convey the appropriate level of uncertainty.

### Extrapolation and post-estimation adjustments

**Fig 2:**
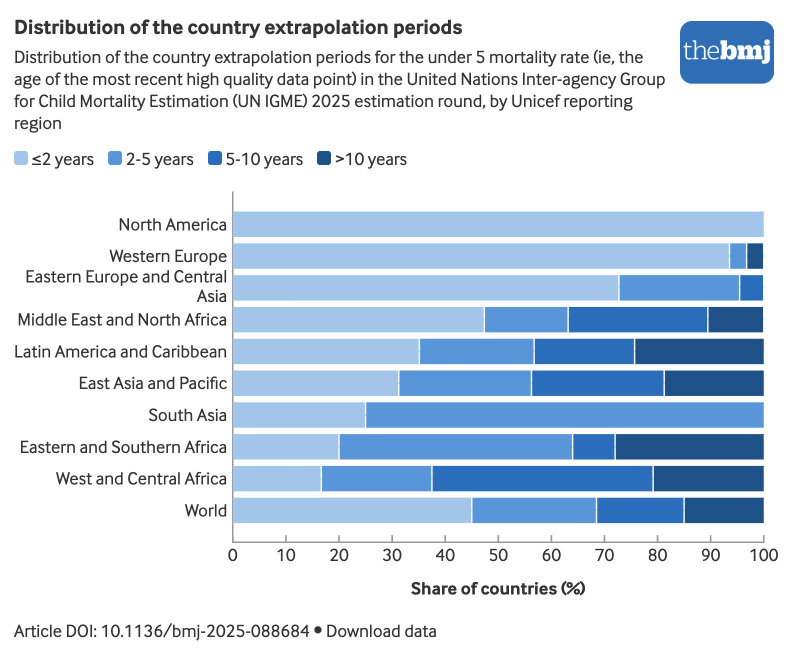
Distribution of country extrapolation periods for estimating under 5 mortality rate (age of the most recent high quality data point) in United Nations Inter-agency Group for Child Mortality Estimation 2025 estimation round, by Unicef region. An interactive version of this graphic is available at https://public.flourish.studio/visualisation/28936052/

In the interest of comparability and consistent with its mandate to produce annual mortality estimates for monitoring progress towards sustainable development goal targets, the UN IGME extrapolates U5MRs and infant mortality rates from the most recent empirical country data point to a common reference year—2024 for this update. To avoid relying solely on short term national fluctuations, this extrapolation uses an equally weighted combination of recent country specific trends and the broader global trend.^7^ Thirty eight countries had empirical U5MR data available for 2024 at the time of estimation; the remaining 162 countries required extrapolation. Figure 2 shows the distribution of country extrapolation periods for U5MR for the world and by region. In this update, the mean extrapolation period globally was 4.5 years for the U5MR and 4.9 years for the infant mortality rate, with half of all countries having last observed, included U5MR data within the past 3.0 years and infant mortality rate data within 3.8 years. No extrapolation is directly applied in the neonatal mortality rate estimation as the expected neonatal mortality rate is already derived from the extrapolated U5MR estimates. The mean length in years between the last observed empirical data point on the neonatal mortality rate and the common reference year was 5.5 years in this round, as relatively fewer data are available on the neonatal mortality rate compared with the U5MR.

#### Adjustments for crisis

The UN IGME adjusts child mortality estimates to account for deaths from major crisis events, including conflicts, natural disasters, famines, and epidemics. Data on crisis related deaths are compiled from multiple regularly updated sources,^12^ ^13^ ^14^ ^15^ along with reports from the UN and other organisations. Where age specific crisis deaths were well captured in vital registration systems, we took them directly from those data; otherwise, we applied modelled age-sex patterns to allocate total crisis deaths across age-sex groups.^16^

Deaths related to major crises were incorporated into the estimates when they met predefined criteria, resulting in adjustments for 56 countries.^5^ Supplementary appendix section 1.7 provides further details on adjustment for crisis events.

#### Adjustment for HIV/AIDS

Estimates were adjusted to capture the rapid changes in child mortality due to the large scale HIV/AIDS epidemic in 17 countries, using estimates of HIV/AIDS child morality from UNAIDS (the Joint United Nations Programme on HIV/AIDS).^17^ Supplementary appendix section 1.5 provides further details on adjustment for HIV/AIDS.

#### Calculating deaths

We calculated the number of deaths in neonates, infants, and under 5s by dividing each annual birth cohort into 52 equal birth week cohorts, then exposing those cohorts to the appropriate age specific and calendar year specific mortality rates over the first five years of life. All deaths occurring in a given calendar year were allocated to that year and aggregated by age group. Annual live births were drawn from the World Population Prospects 2024 Revision.^18^ UIs for death estimates reflect uncertainty in the mortality estimates; they do not incorporate uncertainty in live birth estimates.

### Assessing the pace of change in child mortality

The pace of change in child mortality was measured using the annual rate of reduction, defined as the annual percentage decrease in the mortality rate over a specified period. The annual rate of reduction is calculated as log(mortality rateₜ_2_/mortality rateₜ_1_)/(t_1_–t_2_), where t refers to a year and t_1_<t_2_. Uncertainty in the annual rate of reduction is derived from the distribution of annual rates of reduction calculated across the posterior trajectories for each country and indicator.

Supplementary appendix section 3 provides all mortality rate estimates, deaths, and annual rates of reduction with 90% UIs by region and country.

### Scenario based projections

We projected the U5MR, infant mortality rate, and neonatal mortality rate for each country and calculated corresponding deaths using projected live births from the World Population Prospects 2024^18^ for 2025-30 under four scenarios: constant 2024 rates, current trends, achieving the sustainable development goal targets, and achieving the high income country thresholds. To ensure internal consistency among the U5MR, infant mortality rate, and neonatal mortality rate, we first derived the component rates of the estimated infant mortality rate and U5MR: the post-neonatal mortality rate (ages 1-11 months) and the child mortality rate (ages 1-4 years). These components, together with the neonatal mortality rate, were projected under each scenario and recombined to obtain the projected infant mortality rate and U5MR.

In the constant 2024 scenario, mortality rates remained at 2024 values for 2025-30. In the current trends scenario, each country’s rate specific, crisis-free annual rate of reduction for 2015-24 was applied to project mortality; a negative annual rate of reduction results in rates held at 2024 levels. If a country reached the lowest observed 2024 neonatal mortality rate (0.7 deaths per 1000 live births), post-neonatal mortality rate (0.1 deaths), or child mortality rate (0.3 deaths) among countries with at least 10 000 live births in 2024, rates were held constant thereafter. Two additional scenarios used the same approach as the current trends scenario but the upper and lower bounds of the 90% UIs for country specific annual rates of reduction were applied to project mortality rates. In the achieving sustainable development goal targets scenario, countries not yet at the target were projected to reach a U5MR of 25 deaths per 1000 live births and a neonatal mortality rate of 12 deaths per 1000 live births by 2030; countries already at or on track to reach the target used the current trends projections. In the achieving high income country scenario, countries were projected to reach the average 2024 high income country U5MR (5.1 deaths per 1000 live births) and neonatal mortality rate (2.8 deaths per 1000 live births) by 2030 unless already below these thresholds.

We calculated regional and global totals by aggregating country level estimates and projections. The term “world” refers to the 200 countries and areas with estimates, and regional groupings follow the Unicef reporting classification (see supplementary appendix section 2).

### Patient and public involvement

This study uses publicly available survey, census, and civil registration data and therefore no patients or members of the public were involved in its design or conduct. Since no participants were recruited, there was no assessment of burden or participation experience.

## Results

### Prevailing levels of child mortality

**Fig 3:**
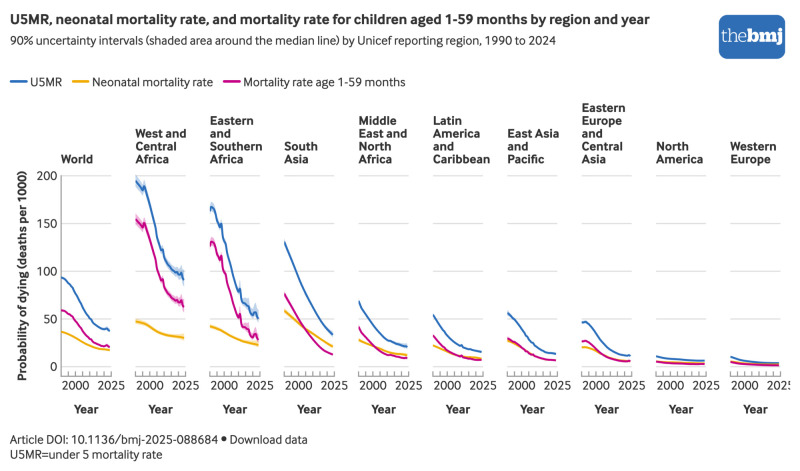
Under 5 mortality rate, neonatal mortality rate, and mortality rate in children aged 1-59 months by region, 1990-2024. An interactive version of this graphic is available at https://public.flourish.studio/visualisation/29086955/

**Fig 4:**
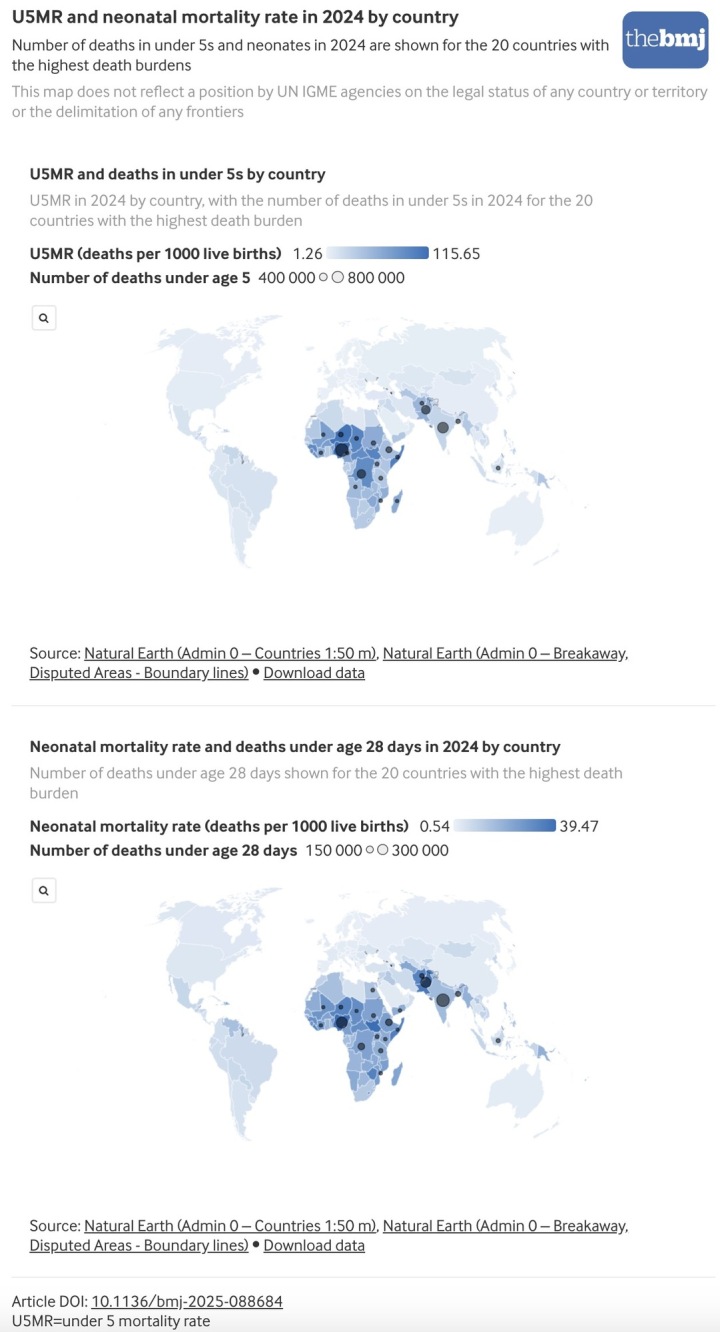
Under 5 mortality rate and neonatal mortality rate in 2024 by country with number of deaths in 2024 for countries with top 20 deaths burdens. An interactive version of this graphic is available at https://public.flourish.studio/visualisation/28940066/

In 2024 globally, the U5MR was 37.4 deaths per 1000 live births (90% UI 36.0 to 40.2) and the neonatal mortality rate was 17.2 deaths per 1000 live births (16.3 to 18.8) (table 1), while the infant mortality rate reached 27.7 deaths per 1000 live births (26.8 to 29.6). West and Central Africa had the highest regional U5MR (91.0 deaths per 1000 live births, (84.0 to 101.4)), followed by Eastern and Southern Africa (50.2 deaths per 1000 live births, (46.1 to 59.7)) (fig 3). Western Europe had the lowest regional U5MR (3.8 deaths per 1000 live births, (3.7 to 3.9)). A similar regional pattern was observed for the infant mortality rate and neonatal mortality rate. U5MRs ranged from 2.0 (1.7 to 2.5) to 115.6 (98.8 to 136.2) deaths per 1000 live births among countries with greater than 10 000 live births in 2024, with similarly wide ranges for the neonatal mortality rate: 0.7 (0.4 to 1.3) to 39.5 (9.4 to 124.3) deaths per 1000 live births (fig 4).

**Table 1:** U5MR and neonatal mortality rate by region and globally, 1990-2024

Region	U5MR (deaths per 1000 live births) (90% UI)					Neonatal mortality rate (deaths per 1000 live births) (90% UI)			
	1990	2000	2015	2024		1990	2000	2015	2024
West and Central Africa	194.9 (188.6 to 201.7)	169.5 (164.5 to 175.1)	102.6 (98.5 to 107.2)	91.0 (84.0 to 101.4)		47.5 (45.0 to 50.4)	41.9 (39.8 to 44.2)	32.3 (30.5 to 34.3)	30.2 (27.0 to 34.8)
Eastern and Southern Africa	163.0 (158.9 to 167.6)	131.6 (128.4 to 135.4)	65.7 (61.8 to 72.4)	50.2 (46.1 to 59.7)		42.4 (40.4 to 44.8)	36.1 (34.5 to 38.1)	25.8 (24.4 to 28.1)	22.7 (20.3 to 27.6)
South Asia	131.0 (127.6 to 134.5)	93.5 (91.0 to 96.2)	49.4 (47.6 to 51.3)	33.5 (30.6 to 37.0)		58.9 (56.3 to 61.6)	45.7 (43.7 to 47.7)	29.5 (28.2 to 30.9)	21.1 (19.1 to 23.4)
Middle East and North Africa	69.0 (67.1 to 71.1)	44.0 (42.7 to 45.5)	25.1 (23.6 to 26.8)	20.5 (18.3 to 23.9)		28.2 (26.1 to 30.3)	21.7 (20.8 to 22.7)	13.8 (12.9 to 15.0)	11.9 (10.5 to 14.1)
Latin America and Caribbean	54.6 (53.0 to 56.3)	32.7 (31.9 to 33.6)	18.3 (17.8 to 18.9)	15.4 (14.3 to 17.2)		22.4 (21.2 to 23.7)	15.8 (15.0 to 16.8)	10.0 (9.7 to 10.4)	8.3 (7.3 to 9.7)
East Asia and Pacific	56.4 (53.7 to 59.5)	39.6 (38.4 to 41.0)	16.6 (16.0 to 17.4)	13.2 (12.1 to 15.0)		27.6 (25.5 to 29.9)	19.9 (18.9 to 21.0)	8.3 (7.9 to 8.8)	6.7 (6.0 to 7.7)
Eastern Europe and Central Asia	46.5 (45.1 to 48.1)	34.9 (33.7 to 36.4)	13.8 (13.4 to 14.3)	11.1 (10.3 to 12.4)		20.3 (19.2 to 21.6)	16.5 (15.8 to 17.4)	7.2 (6.8 to 7.6)	5.6 (5.1 to 6.4)
North America	11.0 (10.8 to 11.2)	8.3 (8.1 to 8.4)	6.6 (6.5 to 6.7)	6.4 (6.1 to 6.6)		5.6 (5.5 to 5.8)	4.6 (4.5 to 4.7)	3.9 (3.8 to 3.9)	3.6 (3.5 to 3.8)
Western Europe	10.4 (10.4 to 10.5)	6.2 (6.2 to 6.2)	4.0 (4.0 to 4.0)	3.8 (3.7 to 3.9)		5.5 (5.4 to 5.6)	3.5 (3.4 to 3.5)	2.4 (2.3 to 2.4)	2.4 (2.3 to 2.4)
World	93.5 (92.2 to 95.0)	76.7 (75.7 to 77.9)	42.9 (42.1 to 44.1)	37.4 (36.0 to 40.2)		36.6 (35.4 to 37.8)	30.3 (29.5 to 31.2)	19.4 (18.9 to 20.1)	17.2 (16.3 to 18.8)

U5MR=under 5 mortality rate; UI=uncertainty interval.

**Fig 5:**
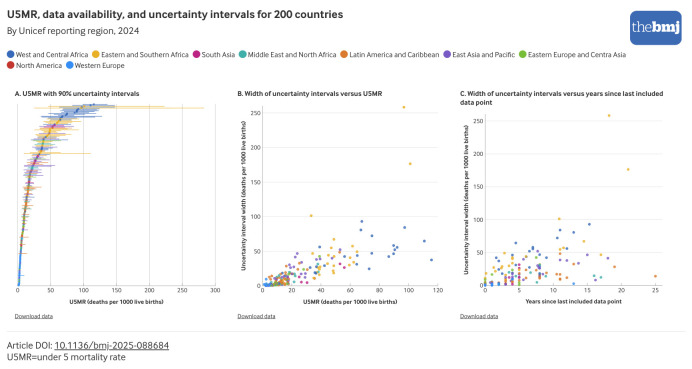
U5MR with 90% uncertainty intervals for 200 countries in 2024 by region; width of uncertainty intervals versus U5MR in 2024 by region; and width of uncertainty intervals versus years since most recent included data point by region. An interactive version of this graphic is available at https://public.flourish.studio/visualisation/28940984/

The width of the UIs is associated with the level of mortality, data availability, and geography. The widest UIs are estimated for countries in West and Central Africa and Eastern and Southern Africa, where the countries with the top 10 highest U5MRs have UIs so wide that mortality levels are statistically indistinguishable (fig 5 panel A). The width of the UIs is also positively associated with both the level of mortality (fig 5 panel B, correlation r=0.76) and sparse or outdated data (fig 5 panel C, correlation r=0.61).

Globally in 2024, an estimated 4.9 million (90% UI 4.7 to 5.2 million) children died before age 5 years, including 3.6 million (3.5 to 3.9 million) infant deaths (75% of under 5 deaths) and 2.3 million (2.1 to 2.5 million) neonatal deaths (47% of under 5 deaths) (table 2). Sub-Saharan Africa, which includes the regions of West and Central Africa and Eastern and Southern Africa, accounted for 60% of global under 5 deaths and 50% of neonatal deaths (table 2 and fig 4), despite representing only 32% (42.4 million of 132.4 million) of global live births. South Asia contributed an additional 25% of under 5 deaths and 33% of neonatal deaths, while having about 27% (35.9 million) of global live births in 2024. The relatively larger share of neonatal deaths in South Asia reflects both a high neonatal mortality rate relative to U5MR and large birth cohorts in the region. As with the mortality rates, uncertainty in estimated deaths was substantial in some cases. For example, in West and Central Africa, the UI for under 5 deaths spanned about 370 000 deaths in 2024.

**Table 2:** Deaths in under 5s and neonates by region and globally, 1990-2024

Region	Under 5 deaths (in thousands) (90% UI)					Neonatal deaths (in thousands) (90% UI)			
	1990	2000	2015	2024		1990	2000	2015	2024
West and Central Africa	2062 (1996 to 2132)	2275 (2208 to 2350)	1963 (1885 to 2052)	1918 (1770 to 2139)		529 (500 to 560)	595 (565 to 628)	635 (601 to 676)	656 (588 to 755)
Eastern and Southern Africa	1869 (1823 to 1919)	1818 (1775 to 1870)	1160 (1092 to 1277)	1014 (931 to 1200)		510 (486 to 539)	519 (496 to 547)	466 (440 to 507)	468 (417 to 568)
South Asia	4988 (4861 to 5119)	3750 (3648 to 3858)	1839 (1772 to 1908)	1196 (1095 to 1320)		2283 (2183 to 2389)	1868 (1788 to 1952)	1100 (1052 to 1153)	756 (686 to 842)
Middle East and North Africa	590 (573 to 607)	344 (333 to 356)	267 (251 to 284)	204 (182 to 237)		244 (226 to 261)	172 (165 to 180)	148 (138 to 161)	119 (105 to 141)
Latin America and Caribbean	644 (626 to 665)	377 (367 to 388)	192 (187 to 199)	143 (133 to 159)		267 (253 to 282)	183 (173 to 193)	105	77
								(101 to 109)	(68 to 90)
East Asia and Pacific	2371 (2258 to 2498)	1250 (1211 to 1296)	539 (518 to 563)	283 (260 to 320)		1186 (1096 to 1285)	635 (603 to 670)	264 (250 to 280)	141 (126 to 163)
Eastern Europe and Central Asia	339 (329 to 350)	184 (177 to 191)	89 (86 to 92)	58 (54 to 65)		146 (137 to 155)	86 (82 to 90)	47 (44 to 49)	29 (26 to 33)
North America	49 (48 to 50)	35 (34 to 36)	29 (29 to 29)	26 (25 to 27)		26 (25 to 26)	20 (19 to 20)	17 (16 to 17)	15 (14 to 15)
Western Europe	58 (58 to 59)	31 (30 to 31)	20 (20 to 20)	16 (16 to 17)		31 (30 to 31)	17 (17 to 17)	12 (11 to 12)	10 (10 to 10)
World	12 970 (12 787 to 13 176)	10 063 (9930 to 10 217)	6096 (5980 to 6269)	4858 (4689 to 5210)		5220 (5059 to 5401)	4095 (3983 to 4217)	2793 (2723 to 2888)	2271 (2148 to 2488)

Estimates of lives births used in the calculation of deaths are from the World Population Prospects 2024.^18^

### Trends in mortality in under 5s

In 2024, the global ratio of neonatal mortality rate to U5MR reached 0.46, increasing from 0.39 in 1990, 0.40 in 2000, and 0.45 in 2015. This trend reflects faster progress in reducing mortality among children aged 1-59 months than among neonates. Globally, U5MR has decreased by 60% (90% UI 57% to 62%) since 1990, driven largely by a 65% (62% to 67%) decline among children aged 1-59 months, compared with a 53% (48% to 56%) decline in the neonatal mortality rate.

Progress has been uneven across regions, in addition to age groups. Eastern Europe and Central Asia achieved a 76% reduction in U5MR from 1990 to 2024, and East Asia and the Pacific achieved a 77% reduction, while North America had a 42% decline. In the highest mortality regions of West and Central Africa, Eastern and Southern Africa, and South Asia, U5MR declined by 53%, 69%, and 74%, respectively, while neonatal mortality rates declined by 37%, 46%, and 64%, respectively. These regional inequalities highlight differing baseline mortality levels, health system capacities, and exposure to conflict and crisis.

### Pace of decline in mortality in under 5s

**Fig 6:**
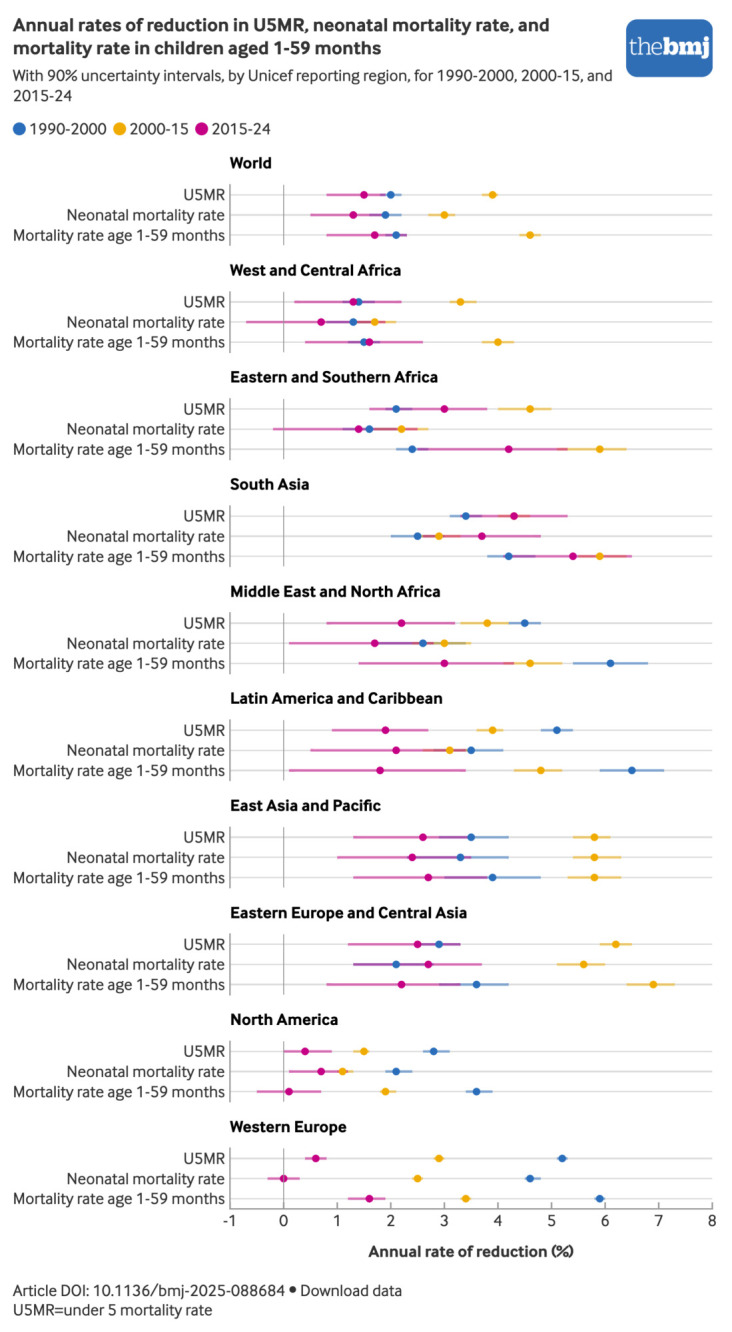
Annual rate of reduction for neonatal mortality rate, mortality rate in children aged 1-59 months, and U5MR for three periods by Unicef reporting regions. An interactive version of this graphic is available at https://public.flourish.studio/visualisation/28941624/

Considering three major periods in the global child survival agenda for which estimates were available—the child survival revolution (1990–2000) and the eras of the millennium development goals (2000-15) and the sustainable development goals (2015-24)—there was a clear acceleration in global mortality reduction during the era of the millennium development goals and subsequent slowdown during the era of the sustainable development goals (fig 6). The global annual rate of reduction for U5MR increased from 2.0% (1.8% to 2.2%) in the 1990s to 3.9% (3.7% to 4.0%) during 2000-15 but then slowed to 1.5% (0.8% to 1.9%) during 2015-24 (fig 6). Globally, progress in reducing U5MR during 2015-24 was 61% slower than during 2000-15 and 23% slower than in the 1990s. Similarly, the decline in the neonatal mortality rate slowed from a 3.0% (2.7% to 3.2%) annual rate of reduction during 2000-15 to 1.3% (0.5% to 1.9%) during 2015-24, a 54% reduction. Likewise, the decline in the neonatal mortality rate in the era of the sustainable development goals was 28% slower than in the 1990s.

Several regions mirror this global pattern, with some notable exceptions. The median annual rates of reduction for both the U5MR and neonatal mortality rate in South Asia increased over all three periods (although with overlapping UIs), while both decreased in Latin America and the Caribbean. Notably, West and Central Africa and Eastern and Southern Africa not only experienced a slowdown in U5MR and neonatal mortality rate reduction during 2015-24, but also their annual rates of reduction for neonatal mortality rate during the era of sustainable development goals were among the lowest across all regions, indicators, and periods.

Across all three periods, reductions in the mortality rate in children aged 1-59 months outpaced reductions in the neonatal mortality rate at the global level (fig 6), especially during 2000-15 and in the highest mortality regions. This persistent differential in pace contributed to the rising share of under 5 deaths occurring in the neonatal period globally and in some regions.

**Fig 7:**
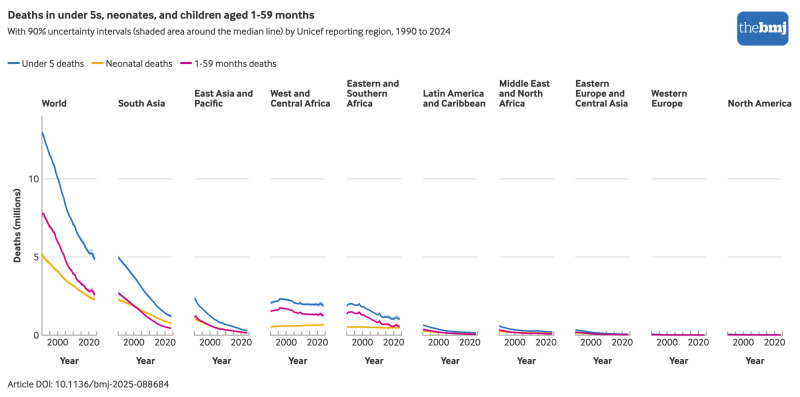
Deaths in under 5s, neonates, and children aged 1-59 months by region, 1990-2024. An interactive version of this graphic is available at https://public.flourish.studio/visualisation/28939534/

Despite declines in mortality rates, some regions experienced stagnation in the number of under 5 or neonatal deaths, as growth in live births outpaced gains from falling mortality rates. In particular, combined neonatal deaths in the two sub-Saharan African regions have remained close to one million annually because of modest reductions in neonatal mortality and increasing numbers of live births (fig 7). In addition, the global pattern of under 5 deaths increasingly concentrated in the neonatal period was visible in most regions but especially in South Asia, where the trajectories for under 5 and neonatal deaths are converging and neonatal deaths now outnumber deaths in children aged 1-59 months.

### Assessment of progress towards sustainable development goal targets

An estimated 134 countries have achieved the sustainable development goal target for U5MR, and based on the current trends projection, six more countries are expected to do so by 2030. That leaves 60 countries—with about 42% (274.2 million) of the global under 5 population (645.8 million) in 2024—falling short of the target. One hundred and twenty six countries have met the target for the neonatal mortality rate, with eight more expected to do so by 2030. Sixty six countries, representing 62% (403.4 million) of the global under 5 population in 2024, are at risk of missing the neonatal mortality rate target.

This assessment is based on the median estimated mortality rates and therefore reflects the most likely trajectory, or a “greatest certainty” scenario. However, incorporating uncertainty provides a broader view of potential progress. Assuming a slower pace of decline based on the lower UI bounds of country specific annual rates of reduction, the number of countries at risk of missing the target would increase to 63 for the U5MR and to 73 for the neonatal mortality rate. Conversely, assuming a faster pace of decline with the upper bound of the UI for country specific annual rates of reduction reduces the number of countries at risk of missing the U5MR and neonatal mortality rate targets to 45 and 54, respectively.

### Projections of future mortality and deaths in 2025-30

If the median current trends continue, an estimated 27.3 million children will die before age 5 years between 2025 and 2030, with a projected global U5MR of 33.3 deaths per 1000 live births in 2030. Of these deaths, 12.9 million (47%) are expected to occur in the neonatal period.

**Fig 8:**
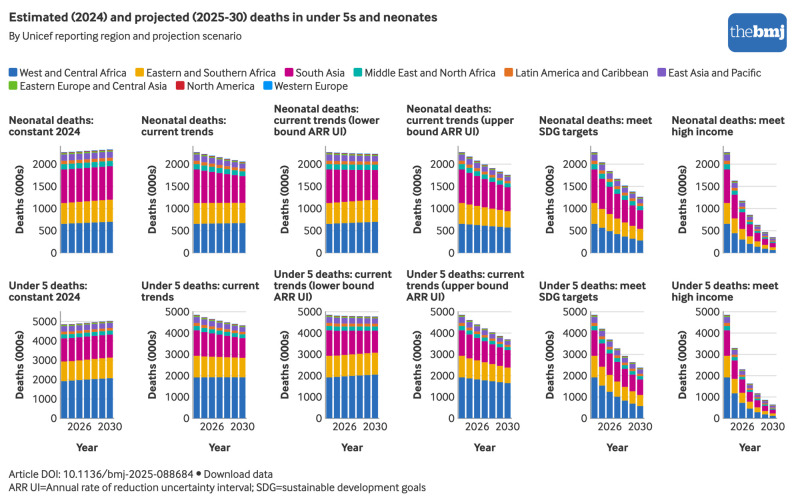
Estimated under 5 and neonatal deaths in 2024 and projected under 5 and neonatal deaths during 2025-30 by region and projection scenario. An interactive version of this graphic is available at https://public.flourish.studio/visualisation/28942293/

The burden of these projected deaths will remain heavily concentrated in a small number of regions (fig 8). West and Central Africa is projected to account for 42% (11.6 million) of all under 5 deaths during 2025-30, with Eastern and Southern Africa contributing an additional 21% (5.7 million) and South Asia another 23% (6.2 million). The geographical concentration of neonatal deaths shifts slightly—31% (4.0 million) of neonatal deaths during 2025-30 are projected to occur in South Asia, with West and Central Africa accounting for 31% (4.0 million), followed by Eastern and Southern Africa with 22% (2.8 million). Notably, South Asia has the largest share of neonatal deaths for the first three years of the projection, 2025-27, in this scenario, but in the final three years, 2028-30, West and Central Africa has the largest share.

If the upper bound of the country UIs for the annual rate of reduction in 2015-24 is used in the continuing current trends scenario, reflecting a faster pace of decline, the projected number of under 5 deaths during 2025-30 would be almost 9% lower than the median scenario, at 24.9 million deaths, including 11.7 million neonatal deaths. Conversely, if mortality is declining more slowly, as implied by the lower bound of the UI for the annual rate of reduction, global under 5 deaths could reach 28.8 million, with 13.5 million neonatal deaths (fig 8). Meanwhile, under the scenario where mortality rates stagnate at 2024 levels, 29.7 million under 5 deaths, including 13.8 million neonatal deaths, would occur before 2030.

Under a scenario where all countries at risk of missing the sustainable development goal targets were instead to meet the U5MR and neonatal mortality rate thresholds by 2030, deaths would remain highly concentrated in the regions of sub-Saharan Africa and South Asia (fig 8), but total estimated under 5 deaths would be just 19.1 million, including 9.7 million neonatal deaths. If all countries were to substantially accelerate progress and reach the average U5MR and neonatal mortality rate levels observed in high income countries, the global number of under 5 deaths during 2025-30 would fall to 9.9 million, including 5.1 million neonatal deaths (fig 8).

## Discussion

This study describes current levels and trends in global child mortality, including progress in mortality reduction and remaining gaps in achieving international targets. In 2024, an estimated 4.9 million children died before age 5 years, with almost half of these deaths occurring in the first month of life. The burden of child mortality remains unevenly distributed, with sub-Saharan Africa and South Asia accounting for a disproportionately large share of global deaths in under 5s and neonates, driven by a combination of higher mortality rates and larger numbers of births relative to other regions. Since 1990, the ratio of the global neonatal mortality rate to U5MR has increased, reflecting the greater decline in mortality among children aged 1-59 months than among neonates. Globally, the U5MR fell by 60% between 1990 and 2024, driven by a 65% decline in mortality in children aged 1-59 months and a 53% decline in the neonatal mortality rate. Despite those declines, progress in reducing mortality from all causes in under 5s has slowed significantly during the era of the sustainable development goals (2015-24) compared with the era of the millennium development goals (2000-15), with global reduction in U5MR slowing by 61% between the two periods. This finding is reflected in the results of the paper examining cause specific mortality in this series, which found slower progress across most main causes for both newborns and children aged 1-59 months.^19^ Finally, while many countries have met or are on track to meet the sustainable development goals’ targets for child survival, 67 countries, with more than 400 million children under age 5 in 2024, are at risk of missing one or both of these targets.

Under the median current trends scenario, more than 27 million under 5 deaths are projected to occur globally between 2025 and 2030. Despite this sobering projection, historical trends show that rapid acceleration in mortality decline is possible. During the millennium development goals era (2000-15), the world achieved historically rapid declines in mortality rates—and persistently high rates in several regions today indicate substantial room for further decline. At the same time, several countries have already achieved U5MRs below 5 deaths per 1000 live births, showing what is biologically possible. If all countries currently at risk of missing the sustainable development goals’ targets for under 5 and neonatal mortality were to meet those targets by 2030, 8.2 million deaths in under 5s would be averted compared with the current trends scenario. An even more ambitious scenario illustrates the potential for further gains: if all countries reached the 2024 high income averages for the U5MR and neonatal mortality rate by 2030, 17.4 million deaths in under 5s could be averted relative to the current trends scenario.

Given the global slowdown in mortality reduction since 2015, it is unlikely that all countries will achieve either the sustainable development goals’ targets or the high income levels by 2030 without renewed investment. These scenarios illustrate both the scale of the challenge and the potential gains from accelerated progress. Uncertainty intervals also provide alternative and perhaps more plausible scenarios. Under a faster pace of decline represented by the scenario using the upper bound of the UI for country annual rates of reduction in 2015-25, 24.9 million under 5 deaths are projected for 2025-30. In contrast, a further slowdown in mortality decline would result in millions more preventable deaths: under a scenario based on the lower bound of the interval, representing a slower pace of decline, 28.8 million under 5 deaths would occur. Stagnation is also possible. If mortality rates were to remain at 2024 levels with no further progress, almost 30 million deaths would occur in under 5s before 2030, underscoring the considerable deaths burden associated with stagnation or further slowdown.

Mortality patterns mirror geographical and cause specific distributions. Globally, the greater decline in mortality among children aged 1-59 months than among neonates reflects the relative success of interventions targeting infectious and communicable diseases, the major causes of death in the 1-59 month age group,^19^ compared with the more complex, often non-communicable causes associated with the time around birth. South Asia continues to have relatively high neonatal mortality given its level of under 5 mortality, consistent with a cause-of-death structure dominated by conditions and complications in the prenatal period and around the time of birth. In contrast, in lower mortality regions, neonatal deaths represent a large share of under 5 deaths due to the near elimination of infectious disease mortality in children aged 1-59 months. Stagnation in neonatal deaths in sub-Saharan Africa reflects the combination of slowly declining neonatal mortality rates and increasing number of births. The regions of West and Central Africa and Eastern and Southern Africa are projected to experience a combined 264 million live births during 2025-30,^18^ reinforcing the scale of the challenge and the urgency of accelerating progress in mortality reduction.

### Limitations of this study

This study has several limitations that should be considered when interpreting the results. Firstly, interpreting the levels and trends in the estimates requires careful attention to the associated uncertainty. A well functioning vital registration system that continuously records all births and deaths is the preferred data source for monitoring under 5 mortality. However, these systems operate in only about a third of countries, covering approximately 20% of the global under 5 population in 2024. Most low and middle income countries—including the countries with the highest estimated mortality rates in sub-Saharan Africa and South Asia—rely on household surveys and, less frequently, censuses, to estimate child mortality at the national level. While these sources can provide essential, high quality information, they are inherently limited—they are retrospective, conducted infrequently, and may be subject to reporting errors, and, in the case of household surveys, sampling errors. The estimated UIs reflect the quantity and quality of available data. Consequently, data gaps are greatest where mortality levels are highest and resulting uncertainty in estimates for these countries is substantial. In addition, in the absence of empirical data, recent estimates are an extrapolation based on a weighted combination of country and global trends, which may not adequately reflect recent levels or trends in mortality. In countries with ongoing conflicts or emergencies, the lack of data poses even more challenges to informative mortality estimation. Furthermore, although this study provides a systematic assessment of global, regional, and national child mortality, it does not examine specific interventions that might be directly associated with deaths in newborns or children, nor the underlying causes of death; the latter are addressed in another paper in this series.^19^

Important questions remain for future research. Although the UN IGME estimates include adjustments for crisis related mortality, these inputs are themselves uncertain, and further work is needed to incorporate both the impacts of crises and their uncertainty into mortality models and projections. Likewise, further work is needed to incorporate uncertainty around live birth estimates, an input to the calculations of deaths. The UIs for death counts from the UN IGME reflect uncertainty in the mortality rates but do not incorporate uncertainty in live births. This approach may underestimate the true uncertainty around death counts—particularly in settings where vital registration and data systems are weak and birth estimates are themselves uncertain. Declines in funding in recent years for maternal, newborn, and child health interventions may introduce additional risks not reflected in the mortality estimates and projections, and more research is needed to understand the impact of funding cuts in both the short term and the long term. Reduction in funding for global data and monitoring systems such as household surveys threaten future data availability, and researchers will need to explore alternative data sources, including those of potentially lower quality, to sustain monitoring in data sparse regions. More research is also needed to understand how shifts in cause-of-death patterns relate to intervention coverage and mortality trends, especially in regions where neonatal related causes are increasingly predominant. Finally, future work should prioritise more granular, subnational modelling of all cause and cause specific mortality. Large within country inequalities persist in many settings and may widen if service disruptions intensify because of potential shocks such as the covid-19 pandemic, highlighting the need for more localised approaches to monitoring and intervention planning.

### Strengths of this study

Despite the limitations mentioned, this study has notable strengths. The UN IGME estimates are updated and published annually using robust methods, the latest data, and a country consultation process, providing the most comprehensive, transparent, and up-to-date source of national, regional, and global child survival statistics. Using a consistent methodology for all countries enhances comparability despite differences in data sources and availability and ensures internal consistency across neonatal, infant, and under 5 mortality estimates. The estimated mortality rates and deaths also serve as essential inputs for other global analyses, including cause-of-death modelling presented in companion papers in this series.^19^ ^20^ The explicit assessment of uncertainty offers a transparent view of data limitations and helps contextualise progress towards global child survival goals.

The findings underscore several important implications for governments, policymakers, and global health practitioners. The slower decline in neonatal mortality reflects the distinct cause-of-death structure for neonates. While mortality reductions among children aged 1-59 months have been driven largely by declines in infectious diseases, neonatal deaths remain dominated by complications of birth, prematurity, and maternal health conditions.^19^ These causes can be more complex to prevent and may require specialised equipment, skilled providers, and stronger health systems. Nevertheless, most neonatal deaths are preventable, and many effective and scalable interventions exist, including attendance by skilled health staff at birth, high quality care for small and sick newborns, access to maternity care, and timely management of complications in newborn babies.^21^

Declining resources for maternal, newborn, and child health programmes present additional risk. The Organisation for Economic Co-operation and Development projects a 9-17% reduction in official development assistance in 2025 compared with 2024, including a 14-29% reduction for health and population services. This comes after a 9% reduction in 2024.^22^ Several studies have linked adequately funded donor programmes to reductions in under 5 mortality in low and middle income countries, including associated declines in mortality from HIV/AIDS, malaria, tuberculosis, nutritional deficiencies, diarrhoeal diseases, lower respiratory tract infections, and maternal and perinatal conditions.^23^ ^24^ At a time when progress is slowing, reductions in funding for essential services could reverse improvements and leave millions of children at increased risk of death.^23^ ^24^ ^25^ ^26^ Disruptions to data systems as a result of funding cuts, including the major household survey programmes, health management information systems, and civil registration and vital statistics systems, threaten the ability to monitor trends and target interventions effectively. Given the substantial share of mortality data derived from household surveys in high mortality settings,^27^ diminished coverage will make it harder to track progress and maintain accountability for child survival goals.

### Conclusion

Despite decades of progress, child survival remains an urgent concern. It is estimated that more than 13 000 children under 5 years of age died every day in 2024 from causes that are largely preventable or treatable. Each of these deaths represents a profound loss for families and communities, and the persistence of such large scale, unequal mortality, especially when effective interventions are well known and widely accessible, represents an ongoing collective failure. Although global agreements have affirmed survival as a fundamental right of every child, this right remains unrealised for many, particularly in the regions where mortality is highest and resources are most constrained.

Achieving the global ambition of ending preventable deaths in children will require renewed commitment and coordinated action to ensure universal healthcare and high quality interventions across the continuum of care, beginning in the prenatal period and extending through early childhood and beyond. Interventions must be targeted to the leading causes of death in each age group, with particular emphasis on the neonatal period, where progress has been slowest. The burden of deaths concentrated in sub-Saharan Africa and South Asia further underscores the need for intensified investment in high impact maternal, newborn, and child health interventions in these regions.

Strengthening national data systems is also essential. Reliable and timely information is needed to monitor trends, identify populations at highest risk, and target resources effectively. Yet many of the countries facing the greatest mortality risk have the weakest data systems, and recent funding cuts threaten to widen these gaps. Reversing these trends and improving the availability and use of high quality data will be critical for accountability and for guiding policies that can accelerate progress in child survival. In the longer term, the development of complete and accurate civil registration and vital statistics systems is needed in low and middle income countries to gather accurate, timely, and disaggregated data.

The rapid declines in child mortality observed during earlier global initiatives show what is possible when international cooperation, political commitment, and investment come together. That legacy should not be squandered. Instead, it should motivate renewed efforts to usher in a new era of child survival in which preventable and treatable deaths are eliminated and the right of every child to survive is finally upheld.

**What is already known on this topic**

Child mortality has declined substantially since the 1990s, driven by high impact interventions such as immunisation, oral rehydration therapy, and improved nutrition, with the global mortality rate in under 5s falling by more than half during the millennium development goal era (2000-15)
The sustainable development goals (SDGs) set targets for all countries to reduce under 5 mortality to 25 deaths per 1000 live births or lower and neonatal mortality to 12 deaths per 1000 live births or lower by 2030, requiring continued monitoring of progress at global, regional, and national levels
Many countries with the highest burdens of child mortality lack reliable and timely data, making the work of the UN Inter-agency Group for Child Mortality Estimation, the only body producing annual child mortality estimates in consultation with national governments, essential for tracking progress towards these targets

**What this study adds**

This study provides updated estimates of mortality in under 5s and neonates for 200 countries and areas from 1990 to 2024, documenting a significant slowdown in the pace of mortality reduction during the SDG era (2015-present) compared with the millennium development goals era, with more than 60 countries at risk of missing one or both SDG child survival targets by 2030
Using scenario based projections, the study estimates that more than 27 million deaths in under 5s could occur between 2025 and 2030 under current trends, while showing that meeting SDG targets could avert 8.2 million of those deaths, and slowdown or stagnation in mortality rates could result in even more deaths by 2030

## Data Availability

The B3 model codes are available upon request to the corresponding author. The data underlying the findings in this paper are openly and publicly available and can be found here: www.childmortality.org. If you encounter problems accessing the data, please contact the corresponding author.

## Web extra

Extra material supplied by authors

Supplementary information: Additional methods, regional classifications, tables, and figures

## Notes

Contributors: DS, LH, and DY conceived the study and completed the data analysis. LH, DS, YL, and GF contributed to the database construction, and all authors reviewed and had access to the underlying data for this study. YL and GF verified the raw data. DS, LH, YL, and GF produced the estimates, and all authors reviewed the final results. DS drafted the original manuscript. All authors reviewed the manuscript and provided input and edits to the article and approved its final version for submission. DY and DS are the guarantors. The corresponding author attests that all listed authors meet authorship criteria and that no others meeting the criteria have been omitted.

Transparency: The lead author (the manuscript’s guarantor) affirms that the manuscript is an honest, accurate, and transparent account of the study being reported; that no important aspects of the study have been omitted; and that any discrepancies from the study as planned (and, if relevant, registered) have been explained.

Dissemination to participants and related patient and public communities: Estimates and underlying data are publicly available on www.childmortality.org. All estimates have been shared with national governments.

Provenance and peer review: Not commissioned; externally peer reviewed.

Published maps are provided without any warranty of any kind, either express or implied. BMJ remains neutral with regard to jurisdictional claims in published maps.

This article is part of a collection proposed by Unicef, the World Health Organization, and Johns Hopkins University. The three organisations provided funding for the collection. Unicef funded the open access fees for this article. *The BMJ* peer reviewed, edited, and decided to publish this article. Elizabeth Loder, Navjoyt Ladher, and Paul Simpson were the lead editors for *The BMJ*.
