# Supplementary material for: Global, regional, and national levels and trends in under 5, infant, and neonatal mortality during 1990-2024 with scenario based projections to 2030: modelling study
Source: BMJ. 2026 Jun 4;393:e088684. doi: 10.1136/bmj-2025-088684 (PMC13235747; doi:10.1136/bmj-2025-088684)
Supplement: Supplementary file 1 — Supplementary information: Additional methods, regional classifications, tables, and figures [file shad088684.ww.pdf]

# Supplementary appendix to Global, regional and national levels and trends in under 5, infant and neonatal mortality 1990-2024 with scenario based projections to 2030

David Sharrow[1], Lucia Hug[1], Yang Liu[1], Graeme Wilson Fell[1], Danzhen You[2]
[1] UNICEF Office of Strategy and Evidence – Innocenti, Florence, Italy
[2] UNICEF Office of Strategy and Evidence – Innocenti, New York, New York, USA

## Contents

1. Methods ... 3
1.1. Overview ... 3
1.2. Data sources ... 3
Civil registration data ... 4
Survey Data ... 5
Adjustment for missing mothers in high-HIV-prevalence settings ... 6
Systematic and random measurement error ... 6
1.3. Estimating levels and trends in under-5 mortality rate ... 7
Summary ... 7
Splines regression ... 8
1.4. Estimation levels and trends in infant mortality rates ... 10
1.5. Adjustment for rapidly changing child mortality driven by HIV/AIDS ... 11
1.6. Estimating levels and trends in neonatal mortality rates ... 11
1.7. Adjustment for crisis ... 12
1.8. Estimation of uncertainty intervals ... 13
1.9. Extrapolation to common reference year ... 13
1.10. Calculated indicators ... 13
1.11. Calculating number of deaths ... 13
2. Regional Classifications ... 15
3. Supplementary tables ... 17
3.1. Table A.1: Global and regional mortality rates, 1990–2024 ... 17
3.2. Table A.2: Global and regional deaths, 1990–2024 ... 18
3.3. Table A.3: Global and regional annual rate of reduction ... 19
3.4. Table A.4: Country mortality rates, 1990–2024 ... 20
3.5. Table A.5: Country deaths, 1990–2024 ... 33
3.6. Table A.6: Country annual rate of reduction ... 44

3.7. Table A.7: Data series in U5MR database .......... 55
4. Supplementary figures: Estimates, with underlying data, by country .......... 134

# 1. Methods

## 1.1. Overview

The UN IGME employs the following broad strategy to arrive at annual estimates of child mortality:

1. Compile and assess the quality of all available nationally representative data relevant to the estimation of child mortality, including data from vital registration systems, population censuses, household surveys and sample registration systems;
2. Assess data quality, recalculate data inputs and make adjustments as needed by applying standard methods;
3. Fit a statistical model to these data to generate a smooth trend curve that averages possibly disparate estimates from the different data sources for a country;
4. Extrapolate the model to a target year (in this case, 2024).

To increase the transparency of the estimation process, the UN IGME has developed a child mortality web portal, Child Mortality Estimation (CME) Info, available at <childmortality.org>. It includes all available data and shows estimates for each country as well as which data are currently officially used by the UN IGME. Once new estimates are finalized, CME Info is updated accordingly.

The UN IGME applies a common methodology across countries and uses empirical data from each country to produce comparable estimates, i.e., country values for the same reference year produced using a common method. Applying a consistent methodology allows for comparisons between countries, despite the varied number and types of data sources. UN IGME estimates are based on nationally available data from censuses, surveys or vital registration systems. The UN IGME does not use covariates to derive its estimates, but, rather, applies a curve-fitting method to empirical data after data quality assessment.

Countries may use a single data source for their official estimates or apply valid methods different from those used by the UN IGME. The UN IGME does not report figures produced by individual countries using other methods, as these estimates would not be comparable across countries. The differences between UN IGME and national official estimates are usually not large if the empirical data are of good quality. The UN IGME aims to minimize errors for each estimate, harmonize trends over time, and produce up-to-date and comparable estimates of child mortality. Because errors are inevitable in data, there will always be uncertainty around data and estimates. To allow for added comparability, the UN IGME generates all child mortality estimates with uncertainty bounds.

## 1.2. Data sources

The first step in the process of arriving at estimates of levels and recent trends of child mortality is to compile all newly available data and add the data to the UN IGME database (newly available may include newer, recently released data and occasionally, results from older censuses or surveys not previously available). Nationally representative estimates of under-five mortality can be derived from several different sources, including civil registration and sample surveys. Demographic surveillance sites and hospital data are excluded as they are rarely representative. The preferred source of data is a civil registration system that records births and deaths on a continuous basis. If registration is complete and this system functions efficiently, the resulting estimates will be accurate and timely. However, many low- and middle-income countries do not have well-functioning vital registration (VR) systems. Therefore, household surveys such as the UNICEF-supported Multiple Indicator Cluster Surveys, Demographic and Health Surveys, and periodic population censuses have become the primary sources of data on mortality among children under age 5. These surveys ask women about the survival of their children, and it is these

reports (or microdata upon availability) that provide the basis of childhood mortality estimates for a majority of low- and middle-income countries.

Whatever the source of child mortality data, quality is critical. The UN IGME assesses data quality and does not include data sources with substantial non-sampling errors or omissions as underlying empirical data in its statistical model.

The full set of empirical data used in this analysis is publicly available from the UN IGME web portal, CME Info <www.childmortality.org>.

## Civil registration data

Data from civil registration systems are the preferred data source for child mortality estimation. For data from civil registration, the calculation of U5MR and IMR is derived from a standard period abridged life table. The inputs are number of deaths for age group <1 year (noted $D_0$) and for the age group 1-4 years ($D_{1\text{-}4}$), as well as the mid-year population for the same age groups ($P_0$ and $P_{1\text{-}4}$).

The formulae are as follows:

Given that:

$_nq_x$ is the probability of dying between age $x$ and age $x+n$,

$_1M_0 = D_0 / P_0$ , death rate for age <1,

$_4M_1 = D_{1\text{-}4} / P_{1\text{-}4}$ , death rate for age group 1-4,

Then:

$_1q_0 = {_1M_0} / [1+(1- {_1a_0})* {_1M_0}]$

where $_1a_0$ is the fraction of year lived by an infant who died

$_1a_0$= 0.1 for low mortality country and $_1a_0$= 0.3 for high mortality country

$_5q_0 = 1-(1-{_1q_0})*(1-{_4q_1})$

where $_4q_1 = 4*{_4M_1} /[1+ (4- {_4a_1}) * {_4M_1}]$

where $_4a_1$ is the fraction of years lived by a child aged 1-4 years who died

$_4a_1$= 1.6

Finally: IMR = $_1q_0$ *1000 and U5MR= $_5q_0$*1000

For NMR, the number of deaths under one month of age and live births are used to calculate the neonatal mortality rate.

For civil registration data (with available data on the number of deaths and mid-year populations), annual observations were initially constructed for all observation years in a country. For country-years in which the coefficient of variation exceeded 10 per cent for children under 5 years, deaths and mid-year populations were pooled over longer periods. Starting from more recent years, deaths and population were combined with adjacent previous years to reduce spurious fluctuations in countries where small

numbers of births and deaths were observed. The coefficient of variation is defined to be the stochastic standard error of the ${}_5q_0$ (${}_5q_0$ = U5MR/1,000) or ${}_1q_0$ (${}_1q_0$ = IMR/1,000) observation divided by the value of the ${}_5q_0$ or ${}_1q_0$ observation. The stochastic standard error of the observation is calculated with a Poisson approximation using live birth numbers, given by sqrt(${}_5q_0$/lb) or similarly sqrt(${}_1q_0$/lb), where lb is the number of live births in the year of the observation.[1] After this recalculation of the civil registration data, the standard errors are set to a minimum of 2.5 per cent for input into the model. Figure 1 is an illustration of the recalculation of civil registration data for Iceland.

*Figure 1 Illustration of the recalculation of civil registration data for Iceland. Black dots refer to annual observations while red dots refer to the recalculated observations with longer observation periods, with observation-specific uncertainty intervals*

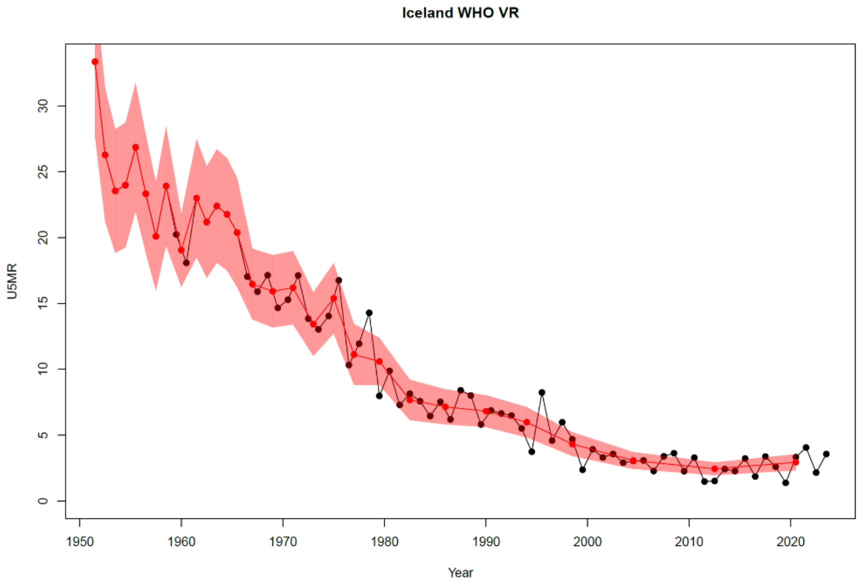


## Survey Data

The majority of survey data on child mortality comes in one of two forms: the full birth history (FBH), whereby women are asked for the date of birth of each of their children, whether the child is still alive, and if not, the child's age at death; and the summary birth history (SBH), whereby women are asked only about the number of children ever born to them and the number who have died (or equivalently, the number still alive).

FBH data, collected by all Demographic and Health Surveys and increasingly, by Multiple Indicator Cluster Surveys and other nationally representative surveys, allow for the calculation of child mortality indicators for specific time periods in the past. This enables these survey programmes to publish under-five child mortality estimates for three 5-year periods before the survey; that is, 0 to 4, 5 to 9, and 10 to 14.[2, 3, 4] The UN IGME has recalculated estimates to refer to calendar year periods using single calendar years for periods shortly before the survey and gradually increasing the number of years for periods further in the past, whenever microdata from the survey are available. The cut-off points of a given survey for shifting from estimates for single calendar years to two years, or two years to three, etc., are based on the coefficients of variation of the estimates.[5]

In general, SBH data collected by censuses and many household surveys use the woman's age as an indicator of the age of her children and their exposure time to the risk of dying and employ models to estimate mortality indicators for periods in the past for women ages 25 to 29 through ages 45 to 49. This

method is well known but has several shortcomings. Starting with the 2014 round of estimation, the UN IGME changed the method of estimation for SBHs to one based on classification of women by the time that had passed since their first birth. This method has several benefits over the previous one. Firstly, it generally has lower sampling errors and, secondly, it avoids the problematic assumption that the mortality estimates derived for each age group of women adequately represent the mortality of the whole population. As a result, it has less susceptibility to the selection effect of young women who give birth early, since all women who give birth necessarily must have a first birth and therefore, are not selected for. Thirdly, the method tends to show less fluctuation across time, particularly in countries with relatively low fertility and mortality. The UN IGME considers the improvements in estimates based on time since first birth worthwhile when compared to the estimates derived from the classification by age of mother. Hence, in cases where the microdata are available, the UN IGME has reanalysed the data using the new method. Due to known biases in the estimation for the 0–4 year period by time since first birth and for the 15–19 and 20–24 age groups of women, these data points are excluded in the estimation model.

Moreover, following advice from UN IGME's TAG, child mortality estimates from SBH were not included if estimates from FBH in the same survey were available.[6]

SBH data are not used to derive neonatal mortality.

### Adjustment for missing mothers in high-HIV-prevalence settings

In populations severely affected by HIV/AIDS, HIV-positive children will be more likely to die than other children and will also be less likely to be reported since their mothers will also have been more likely to die. Child mortality estimates will thus be biased downwards. The magnitude of the bias will depend on the extent to which the elevated under-five mortality of HIV-positive children is not reported because of the deaths of their mothers. The TAG developed a method to adjust HIV/AIDS-related mortality for each survey data observation from FBH during HIV/AIDS epidemics (1980–present) by adopting a set of simplified but reasonable assumptions about the distribution of births to HIV-positive women, primarily relating to the duration of their infection, vertical transmission rates, and survival times of both mothers and children from the time of the birth.[7] This method was applied to all direct estimates from FBHs. The model was improved to incorporate the impact of antiretroviral therapies (ART) and prevention of mother to child transmission (PMTCT).[8]

### Systematic and random measurement error

Data from these different sources require varied calculation methods and may suffer from different errors, such as random errors in sample surveys or systematic errors due to misreporting. Thus, different surveys often yield widely divergent estimates of U5MR for a given period, as illustrated in Figure 2. To reconcile these differences and take better account of the systematic biases associated with the various types of data inputs, the TAG developed an estimation method to fit a smoothed trend curve to a set of observations and to extrapolate that trend to a defined time point, in this case, 2024. This method is described in the following section.

*Figure 2 Empirical child mortality data in Nigeria and Equatorial Guinea*

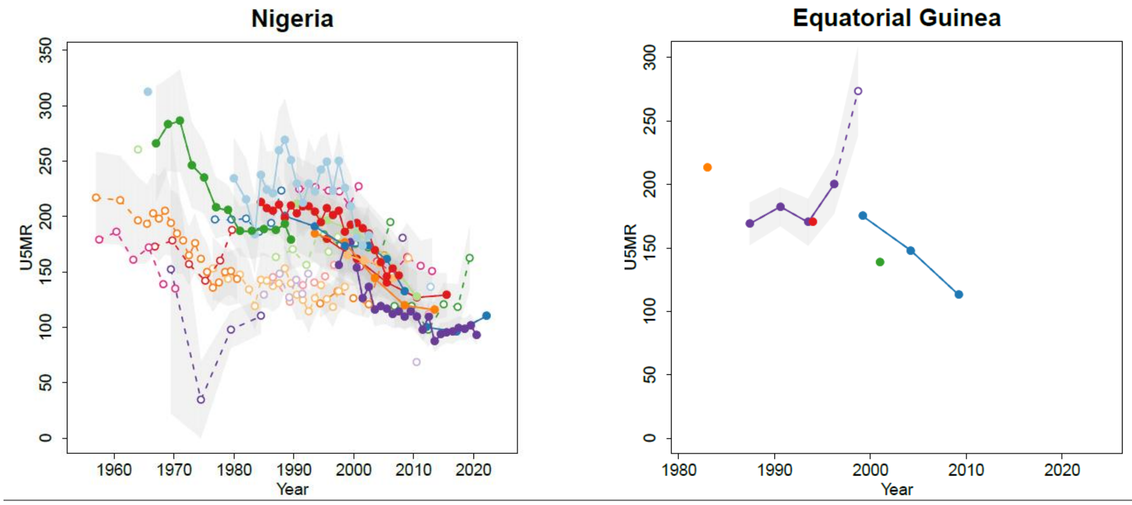


*Note*: All data available for the country are shown as coloured points, with observations from the same data series joined by lines, and each colour identifying different data sources. Solid circles and lines represent data series/observations that were included in the statistical model. Unfilled circles and dash lines represent data series/observations that were excluded. Grey bands represent the standard errors of the observations where available or applicable.

## 1.3. Estimating levels and trends in under-5 mortality rate

### Summary

Estimation and projection of under-five mortality rates was undertaken using the Bayesian B-splines bias-adjusted model, referred to as the B3 model. This model was developed, validated and used to produce previous rounds of UN IGME child mortality estimates, including the previously published round in 2024.[9, 10]

In the B3 model, log(U5MR) is estimated with a flexible splines regression model. The spline regression model is fitted to all U5MR observations in the country. An observed value for U5MR is considered to be the true value for U5MR multiplied by an error multiplier, i.e., observed U5MR = true U5MR * error multiplier, or on the log scale, log(observed U5MR) = log(true U5MR) + log(error multiplier). The error multiplier refers to the relative difference between an observation and the truth with error multiplier equal to 1 (and log(error multiplier) equal to zero) meaning no error.

While estimating the true U5MR, properties of the errors that provide information about the quality of the observation or in other words, the extent of error that we expect, are taken into account. These properties include: the standard error of the observation; its source type (e.g., Demographic and Health Surveys versus census); and whether the observation is part of a data series from a specific survey (and how far the data series is from other series with overlapping observation periods). These properties are summarized in the data model. When estimating the U5MR, the data model adjusts for errors in observations, including the average systematic biases associated with different types of data sources, using information on data quality for different source types from all countries.

Figure 3 displays the U5MR data and B3 model fit over time for Senegal, used here for illustrative purposes.

Compared with the previously applied LOESS (locally estimated scatterplot smoothing) estimation approach,[11] the B3 model better accounts for data errors, including biases and sampling and non-sampling errors in the data. It can more accurately capture short-term fluctuations in the U5MR and its annual rate of reduction and, thus, is better able to account for evidence of acceleration in the decline of under-five mortality from new surveys. Validation exercises show that the B3 model also performs better in short-term projections.

*Figure 3 U5MR estimates and data over time for Senegal. B3 estimates in red and uncertainty in the pink shaded area.*

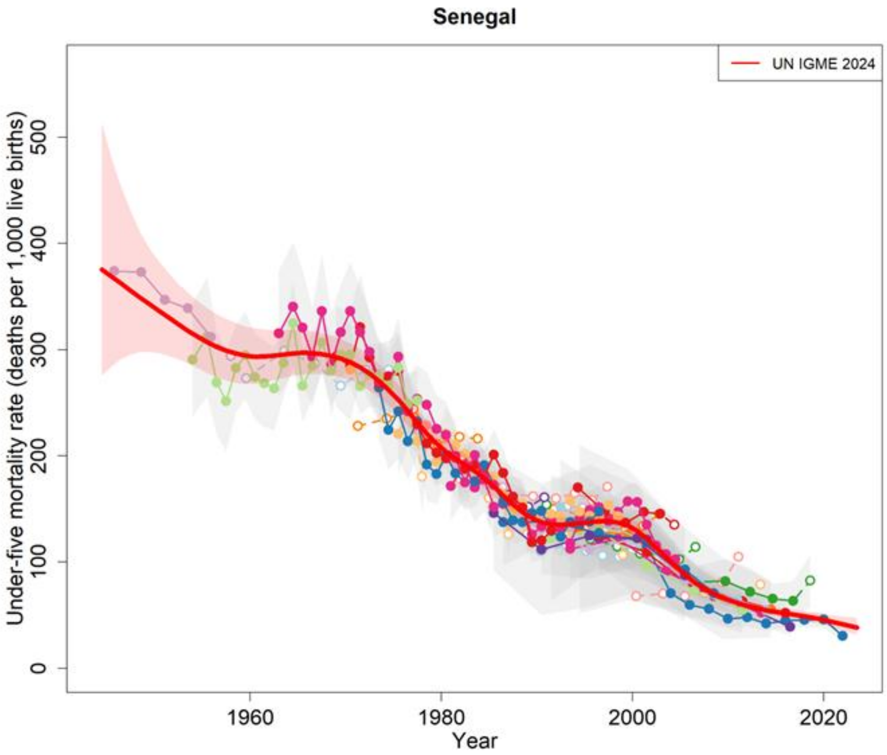


Note: The B3 estimates are in red. Ninety per cent uncertainty intervals for the U5MR are given by the pink shaded area. All data available for the country are shown as coloured points, with observations from the same data series joined by lines. Solid circles and lines represent data series/observations that were included for curve-fitting. Unfilled circles and dash lines represent data series/observations that were excluded. Grey bands represent the standard errors of the observations where available or applicable.

The B3 method was developed and implemented for the UN IGME by Leontine Alkema and Jin Rou New with guidance and review by the UN IGME's TAG. A more complete technical description of the B3 model is available elsewhere.[1, 10] In general, the B3 model described above is applied to the U5MR for all countries (except the Democratic People's Republic of Korea where a non-standard method was employed).

## Splines regression

The splines regression fitting method for Norway is shown in Figure 4 for illustrative purposes. Splines are smooth curves, placed at knots 2.5 years apart, that add up to 1 at any point in time. For any year, the estimated log(U5MR) is the sum of the non-zero splines in that year multiplied by the corresponding spline coefficients (displayed by dots). For example, log(U5MR) in 1980 in Norway is given by the sum of the

yellow and grey splines to the left of black line (at the year 1980) and the black and red splines to the right, multiplied by their respective spline coefficients in the same colour.

The spline coefficients determine what the resulting fitted curve looks like. When estimating the spline coefficients, we obtain a flexible yet reasonably smooth U5MR curve by assuming that the difference between two adjacent coefficients (for example for years 1981 and 1983.5) is given by the difference between the previous two coefficients (for years 1978.5 and 1981) with an estimated data-driven "distortion term" added to it. For example, in Norway during the early 1980s, these distortion terms are estimated to be around zero when U5MR did not change much, but they are negative in the late 1980s when the U5MR started to decline again. The resulting fit in Norway illustrates that the spline fit is able to follow the observed changes in the data closely.

*Figure 4 Illustration of the B-splines regression model for an example country with VR data. Top row: B-splines and the estimated spline coefficients. Bottom row: Observed log(U5MR) and U5MR (black dots) plotted against time, together with the spline estimates (red line).*

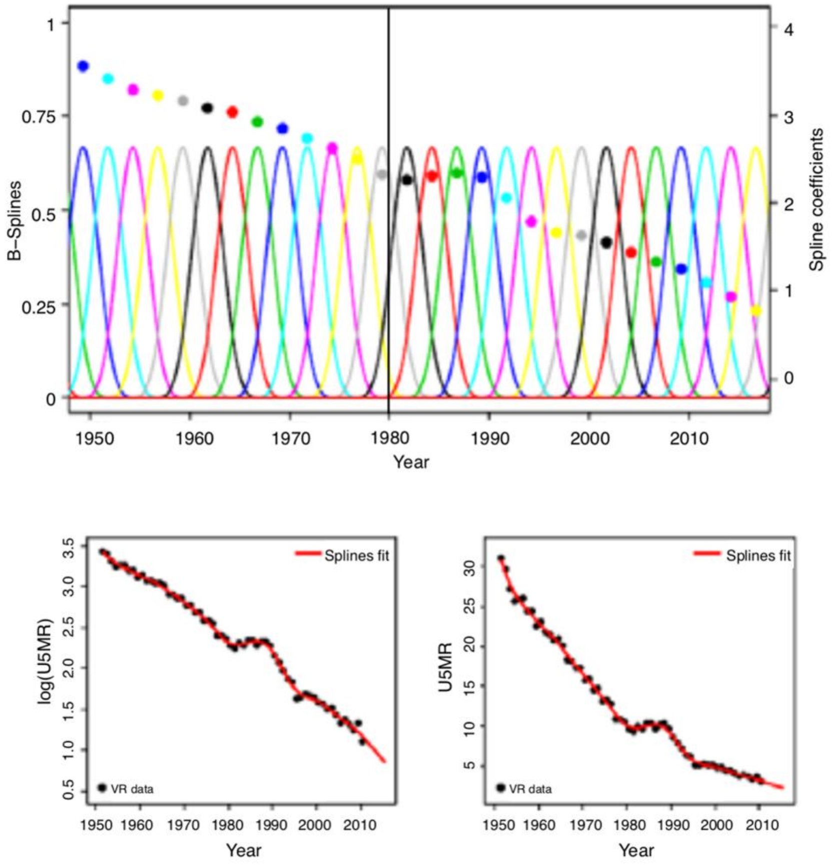


The variance of the distortion terms determines the smoothness of the fit during the observation period; large fluctuations in these distortion terms imply that the trend can vary greatly from one period to the next. The amount of smoothing is country-specific for the majority of countries. An average global level of smoothing is used for countries with a small number of live births, countries with both vital registration

(VR) and non-VR data included in the fitting and countries with a gap of more than five years in their VR data.

Due to the nature of the data in such countries, a small variance for the distortion terms tends to be estimated, so a global level of smoothing helps to reduce fluctuations in the trend.

After the most recent observation period ends, country-specific U5MR projections are obtained through the estimation of "future spline coefficients", or equivalently, by projecting the differences between adjacent spline coefficients. The mean projected difference in spline coefficients is given by the estimated difference in the two most recent adjacent spline coefficients, and the uncertainty therein is based on the variability in the observed distortions in the country's past. Based on out-of-sample validation exercises, this approach is shown to work well for the majority of countries but leads to unnecessarily wide uncertainty intervals (or extreme extrapolations) for a subset of countries where the most recent change in spline coefficients is very uncertain (or an extreme value). We avoid such uncertain and extreme U5MR extrapolations in longer-term projections by combining the country-specific projected differences in spline coefficients with a global distribution of observed differences in the past. This final step results in the removal of very extreme U5MR extrapolations in the country-specific U5MR projections.

### 1.4. Estimation levels and trends in infant mortality rates

The B3 model is also used to estimate the IMR but is fitted to the logit transform of r, i.e., log(r/1-r), where r is the ratio of the IMR estimate to the median B3 estimate of U5MR in the corresponding country-year. This helps to restrict the IMR estimate to be lower than the U5MR estimate for any given year. Since 2024, the B3 method has been applied to all countries (except for the Democratic People's Republic of Korea). Previously, for countries lacking high-quality VR data, the IMR was derived from the U5MR using model life tables that reflect known regularities in age patterns of child mortality.[12] However, recent results have shown that these model life tables were not able to correctly represent the age patterns of child mortality in sub-Saharan Africa and Southern Asia, introducing biases in the IMR estimates.[13]

To address this issue, a new method was introduced to derive the IMR from U5MR values in SBH data during the data processing stage before fitting the B3 model. This new method is based on the observation of log-quadratic relationships between cumulative probabilities of dying *q(x)* and the U5MR:

$$ln[q(x)] = a_x + b_x * ln[U5MR] + c_x * ln[U5MR]^2 + v_x^* k,$$

where *x* corresponds to 22 specific ages between 0 and 5, including 28 days and 12 months, allowing the prediction of the IMR based on U5MR.[14]

The coefficients of this log-quadratic model are based on high-quality VR data and were used to derive IMR from U5MR in SBH data in all countries except for those in sub-Saharan Africa and Southern Asia. In these two regions, the coefficients were updated based on DHS data collected between 1985 and 2022. The parameter *k* determines the shape of the age pattern of mortality between age 0 and 5, and thus the IMR for a given level of U5MR. The advantage of this model is that it allows for precise estimation of the parameter *k* for each country-year based on the neonatal mortality rate (NMR) and U5MR estimated with the B3 model, thereby increasing accuracy for all countries. When only the U5MR is available, the *k* value is based on the earliest year when both NMR and U5MR were available. Then, each U5MR derived from SBH data is converted into an IMR using the corresponding *k* value for the given year.

## 1.5. Adjustment for rapidly changing child mortality driven by HIV/AIDS

To capture the extraordinarily rapid changes in child mortality driven by HIV/AIDS over the epidemic period in some countries, the regression models were fitted to data points for the U5MR from all causes other than HIV/AIDS. UNAIDS estimates of HIV/AIDS under-five mortality were then added to estimates from the regression model. This method was used for 17 countries where the HIV prevalence rate exceeded 5 per cent at any point in time since 1980. Steps were as follows:

1. Compile and assess the quality of all newly available nationally representative data relevant to the estimation of child mortality;
2. Adjust survey data to account for possible biases in data collection and in HIV/AIDS epidemic (i.e., the missing mothers bias);
3. Use UNAIDS estimates of HIV/AIDS child mortality[15] to adjust the data points from 1980 onwards to exclude HIV/AIDS deaths;
4. Fit the standard statistical model to the observations to HIV-free data points;
5. Extrapolate the model to the target year; in this case 2024;
6. Add back estimates of deaths due to HIV/AIDS (from UNAIDS); and
7. Derive a non-AIDS curve of IMR from the estimated U5MR using model life tables; add the UNAIDS estimates of HIV/AIDS deaths for children under age 1 to generate the final IMR estimates.

## 1.6. Estimating levels and trends in neonatal mortality rates

The NMR is defined as the the probability of dying between birth and exactly 28 days of age, expressed per 1,000 live births. In 2015, the UN IGME method for estimating NMR was updated to a Bayesian methodology similar to that used to estimate U5MR and derive estimates by sex. It has the advantage that, compared to the previous model, it can capture data-driven trends in NMR within countries and over time, for all countries. A more complete technical description of the model is available elsewhere.[16]

We model the ratio R(c,t), which refers to the ratio of NMR to the difference of U5MR and NMR in country c and year t, i.e. R(c,t) = NMR/(U5MR - NMR). For each country-year, we assume that the ratio is given by:

R(c,t) = W(c,t) * P(c,t),

where

W(c,t) refers to the expected ratio for that country-year, and

Country multiplier P(c,t) represents country-specific trends in the ratio over time that differ from the expected level.

As U5MR decreases, the proportional share of mortality in the first month of life tends to increase. The W(c,t) term accounts for this relationship; it is the expected ratio for the country-year based on the UN IGME-estimated U5MR for that country-year. It is modelled as a linear function of U5MR with a changing slope:

W(c,t) = β0 if U5MR(c,t) < Ucut

$W(c,t) = \beta0 + \beta1 * U5MR(c,t) \quad \text{if } U5MR(c,t) \geq Ucut$

Ucut is an estimated constant that represents the level of U5MR after which as U5MR increases, the ratio NMR/(U5MR - NMR) decreases. The parameters of this model are estimated based on all available data such that W(c,t) represents a ‘global relation’ between the ratio and U5MR.

The country multiplier P(c,t) is modelled with a B-splines regression model. The P(c,t) represents a country-specific intercept, which is modelled hierarchically, and fluctuations around that intercept over time. For any particular country, the ratio can overall be higher- or lower-than-expected given the level of U5MR in that country, but the fluctuations allow this relationship to change over time within a country. A degree of smoothness is imposed on the fluctuations to ensure relatively smooth trajectories for any given country through time. We model the ratio of NMR/(U5MR - NMR); estimates of NMR are obtained by recombining the estimates of the ratio with UN IGME-estimated U5MR.

For neonatal mortality in HIV-affected and crisis-affected populations, the ratio is estimated initially for non-AIDS and non-crisis mortality. After estimation, crisis neonatal deaths are added back on to the neonatal deaths to compute the total estimated neonatal mortality rate. No AIDS deaths are added to the NMR, thereby assuming these deaths only affect child mortality after the first month of life.

### 1.7. Adjustment for crisis

Estimated deaths from major crises were derived from various data sources from 1950 to the present. Data on natural disasters were obtained from the Centre for Research on the Epidemiology of Disasters’ International Disaster Database.[17] Conflict death data were taken from the Uppsala Conflict Data Program/Peace Research Institute Oslo datasets,[18, 19] Armed Conflict Location & Event Data Project[20] Center for Systemic Peace/Integrated Network for Societal Conflict Research dataset,[21] as well as from reports prepared by the UN and other organizations.

For crises where deaths were adequately recorded in death registration data, age-specific deaths were obtained directly from the data. For many countries, however, age-sex specific data on crisis deaths is not available.For this update, the UN IGME undertook a comprehensive review of more than 1,000 studies and datasets and analyzed vital registration data and survey data (DHS, MICS, and World Fertility Surveys) for regions and years affected by crises. These sources provided age-sex distributions for 174 crisis events: 51 conflicts, 32 earthquakes, 35 famines, 30 epidemics, 10 floods, 9 tsunamis, 4 genocides and 3 cyclones. These data were analysed to prepare age-sex distributions by five-year age groups and for more detailed age groups under 5 for each of the event types as described elsewhere[22].

Estimated child and youth deaths due to major crises were included if they met the following criteria: (1) the crisis was isolated to a few years; (2) under-five crisis deaths, were greater than 10 per cent of non-crisis deaths in the age group; (3) crisis U5MR was > 0.2 deaths per 1,000; (4) the number of crisis deaths among children under 5 years was > 10 deaths.

Crisis deaths were included in the estimates by first excluding data points from crisis years, then fitting the B3 model to the remaining data and adding the crisis-specific mortality rate to the fitted B3 curve. Crisis death estimates are uncertain but, presently, no uncertainty around crisis deaths is included in the uncertainty intervals of the estimates. Instead, we assume the relative uncertainty in the adjusted estimates is equal to the relative uncertainty in the non-adjusted estimates; this assumption will be revisited in the future.

The UN IGME has assessed recent humanitarian crises and, based on the scarcity of currently available data and the difficulties of estimating the broader impact of these crises on health systems, decided to hold the estimates constant from the start of the crisis while increasing the uncertainty over the crisis time for two countries: South Sudan and Venezuela (Bolivarian Republic of). Where applicable, direct crisis deaths have been added to the constant trend estimate. The UN IGME will review new data, if available, in the next estimation round and revise estimates accordingly.

### 1.8. Estimation of uncertainty intervals

Given the inherent uncertainty in child mortality estimates, 90 per cent uncertainty intervals are used by the UN IGME instead of the more conventional 95 per cent intervals. Reporting intervals based on higher levels of uncertainty (i.e., 95 per cent instead of 90 per cent) has the advantage that the chance of not having included the true value in the interval is smaller. The disadvantage of choosing higher uncertainty levels, however, is that intervals lose their utility to present meaningful summaries of a range of likely outcomes if the indicator of interest is highly uncertain. Given this trade-off and the substantial uncertainty associated with child mortality estimates, the UN IGME chose to report 90 per cent uncertainty intervals or in other words, intervals for which there is a 90 per cent chance that they contain the true value, to encourage wider use and interpretation of uncertainty intervals.

### 1.9. Extrapolation to common reference year

If the underlying empirical data refer to an earlier reference period than the end year of the period the estimates are reported, the UN IGME extrapolates the log mortality estimates to the common end year; in this round, to 2024. The UN IGME does not use covariates to derive the estimates but uses and equally weighted combination of the past trend in a country and the global trend to extrapolate to the target year. See section ‘Splines regression’ for details.

### 1.10. Calculated indicators

While U5MR, IMR, and NMR are estimated from empirical data, additional indicators for alternative age intervals can be derived from these estimated probabilities. For example, the probability of death for those aged 1–59 months (MR1to59) can be calculated from the estimated NMR and U5MR using the formula:

$$\text{MR1to59} = 1{,}000 \times (1 - (1{,}000 - \text{U5MR}) / (1{,}000 - \text{NMR})),$$

when expressed per 1,000. Other age-specific probabilities are derived in a similar way. The probability of death for those aged 1-4 years is derived from the estimated IMR and U5MR. The probability of deaths for those aged 1-11 months is derived from the estimated NMR and IMR.

### 1.11. Calculating number of deaths

A birth-week cohort method is used to calculate the absolute number of deaths among neonates, infants and children under age 5. First, each annual birth cohort is divided into 52 equal birth-week cohorts. Then each birth-week cohort is exposed throughout the first five years of life to the appropriate calendar year- and age-specific mortality rates depending on cohort age. For example, the 20th birth-week cohort of the year 2000 will be exposed to the infant mortality rates in both 2000 and 2001. All deaths from birth-week cohorts occurring as a result of exposure to the mortality rate for a given calendar year are allocated to that year and are summed by age group at death to get the total number of deaths for a given year and age group. Continuing with the above example, deaths from the 20th birth-week cohort of the year 2000

would contribute to infant deaths in year 2000 and 2001. Any deaths occurring among the 20th birth-week cohort of year 2000 after the 20th week in 2001 would contribute to under-five deaths for year 2001 and so forth. Under-five deaths in each calendar year are calculated by summing up all the deaths under age 5 across all age group cohorts in that year. The annual estimate of the number of live births in each country from the *World Population Prospects 2024*[23] is used to calculate the number of deaths.

## 2. Regional Classifications

Estimates at the regional level in the paper were based on the UNICEF regional classifications.

**Map A1: UNICEF regional classifications.**

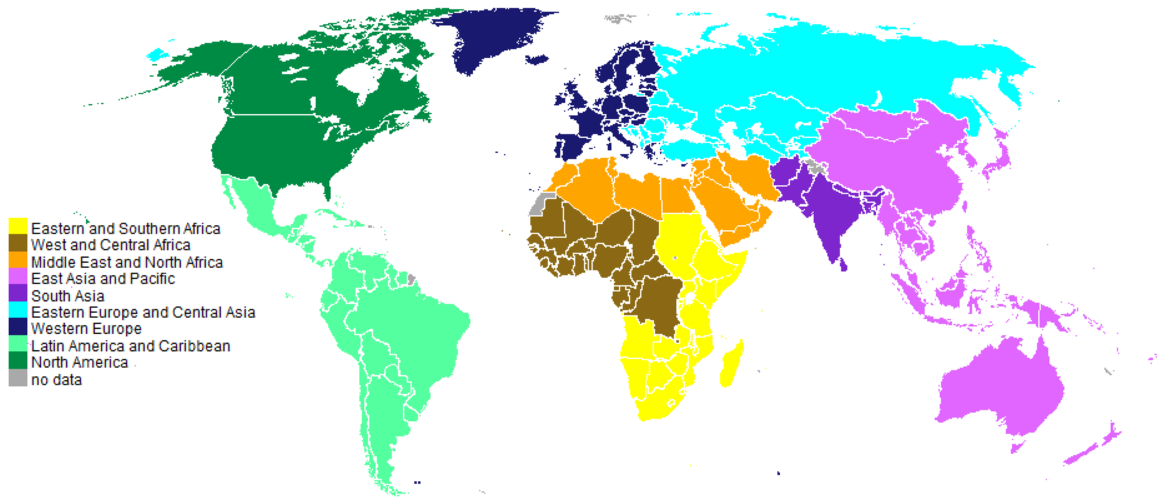


**Note:** This map does not reflect a position by UN IGME agencies or those of the institutions to which the authors are affiliated on the legal status of any country or territory or the delimitation of any frontiers.

### East Asia and Pacific

Australia; Brunei Darussalam; Cambodia; China; Cook Islands; Democratic People's Republic of Korea; Fiji; Indonesia; Japan; Kiribati; Lao People's Democratic Republic; Malaysia; Marshall Islands; Micronesia (Federated States of); Mongolia; Myanmar; Nauru; New Zealand; Niue; Palau; Papua New Guinea; Philippines; Republic of Korea; Samoa; Singapore; Solomon Islands; Thailand; Timor-Leste; Tonga; Tuvalu; Vanuatu; Viet Nam

### Europe and Central Asia

Eastern Europe and Central Asia; Western Europe

### Eastern Europe and Central Asia

Albania; Armenia; Azerbaijan; Belarus; Bosnia and Herzegovina; Bulgaria; Croatia; Georgia; Kazakhstan; Kyrgyzstan; Montenegro; Republic of Moldova; Northern Macedonia; Romania; Russian Federation; Serbia; Tajikistan; Turkey; Turkmenistan; Ukraine; Uzbekistan

### Western Europe

Andorra; Austria; Belgium; Cyprus; Czechia; Denmark; Estonia; Finland; France; Germany; Greece; Hungary; Iceland; Ireland; Italy; Latvia; Lithuania; Luxembourg; Malta; Monaco; Netherlands; Norway; Poland; Portugal; San Marino; Slovakia; Slovenia; Spain; Sweden; Switzerland; United Kingdom

### Latin America and Caribbean

Antigua and Barbuda; Argentina; Bahamas; Barbados; Belize; Bolivia (Plurinational State of); Brazil; Chile; Colombia; Costa Rica; Cuba; Dominica; Dominican Republic; Ecuador; El Salvador; Grenada; Guatemala;

Guyana; Haiti; Honduras; Jamaica; Mexico; Nicaragua; Panama; Paraguay; Peru; Saint Kitts and Nevis; Saint Lucia; Saint Vincent and the Grenadines; Suriname; Trinidad and Tobago; Uruguay; Venezuela (Bolivarian Republic of)

**Middle East and North Africa**
Algeria; Bahrain; Egypt; Iran (Islamic Republic of); Iraq; Israel; Jordan; Kuwait; Lebanon; Libya; Morocco; Oman; Qatar; Saudi Arabia; State of Palestine; Syrian Arab Republic; Tunisia; United Arab Emirates; Yemen

**North America**
Canada; United States of America

**South Asia**
Afghanistan; Bangladesh; Bhutan; India; Maldives; Nepal; Pakistan; Sri Lanka

**Sub-Saharan Africa**
Eastern and Southern Africa; West and Central Africa

**Eastern and Southern Africa**
Angola; Botswana; Burundi; Comoros; Djibouti; Eritrea; Eswatini; Ethiopia; Kenya; Lesotho; Madagascar; Malawi; Mauritius; Mozambique; Namibia; Rwanda; Seychelles; Somalia; South Africa; South Sudan; Sudan; Uganda; United Republic of Tanzania; Zambia; Zimbabwe

**West and Central Africa**
Benin; Burkina Faso; Cabo Verde; Cameroon; Central African Republic; Chad; Congo; Côte d'Ivoire; Democratic Republic of the Congo; Equatorial Guinea; Gabon; Gambia; Ghana; Guinea; Guinea-Bissau; Liberia; Mali; Mauritania; Niger; Nigeria; Sao Tome and Principe; Senegal; Sierra Leone; Togo

# 3. Supplementary tables

## 3.1. Table A.1: Global and regional mortality rates, 1990–2024

Under-five mortality rate (U5MR), infant mortality rate (IMR), neonatal mortality rate (NMR), and mortality rate age 1–59 months (deaths per 1,000 live births) for 1990, 2000, 2015, and 2024, by UNICEF region and World. Values shown are medians with 90% uncertainty intervals in parentheses.

| | Under-five mortality rate (U5MR) (per 1,000 live births) | | | | Infant mortality rate (IMR) (per 1,000 live births) | | | | Neonatal mortality rate (NMR) (per 1,000 live births) | | | | Mortality rate age 1–59 months (per 1,000 children aged 28 days) | | | |
|---|---|---|---|---|---|---|---|---|---|---|---|---|---|---|---|---|
| Region | 1990 | 2000 | 2015 | 2024 | 1990 | 2000 | 2015 | 2024 | 1990 | 2000 | 2015 | 2024 | 1990 | 2000 | 2015 | 2024 |
| Sub-Saharan Africa | 178.4 | 150.4 | 85.0 | 71.2 | 102.1 | 88.9 | 54.2 | 47.1 | 44.9 | 39.0 | 29.2 | 26.6 | 139.8 | 115.9 | 57.5 | 45.8 |
| | (174.7-182.5) | (147.5-153.8) | (82.3-89.1) | (67.5-78.8) | (100.0-104.3) | (87.2-90.9) | (52.6-56.7) | (44.8-52.0) | (43.0-47.1) | (37.5-40.7) | (28.0-30.8) | (24.4-30.4) | (136.4-143.5) | (113.2-118.9) | (55.2-60.9) | (42.5-51.6) |
| West and Central Africa | 194.9 | 169.5 | 102.6 | 91.0 | 103.5 | 94.1 | 60.9 | 56.4 | 47.5 | 41.9 | 32.3 | 30.2 | 154.8 | 133.2 | 72.6 | 62.7 |
| | (188.6-201.7) | (164.5-175.1) | (98.5-107.2) | (84.0-101.4) | (100.2-106.9) | (91.4-97.2) | (58.6-63.7) | (52.2-62.8) | (45.0-50.4) | (39.8-44.2) | (30.5-34.3) | (27.0-34.8) | (149.1-160.8) | (128.7-138.1) | (69.3-76.3) | (56.9-70.7) |
| Eastern and Southern Africa | 163.0 | 131.6 | 65.7 | 50.2 | 100.8 | 83.7 | 46.9 | 37.3 | 42.4 | 36.1 | 25.8 | 22.7 | 126.0 | 99.0 | 40.9 | 28.1 |
| | (158.9-167.6) | (128.4-135.4) | (61.8-72.4) | (46.1-59.7) | (98.2-103.6) | (81.7-86.2) | (44.5-51.0) | (34.3-44.0) | (40.4-44.8) | (34.5-38.1) | (24.4-28.1) | (20.3-27.6) | (122.2-130.0) | (96.2-102.3) | (37.7-46.4) | (25.0-34.4) |
| Middle East and North Africa | 69.0 | 44.0 | 25.1 | 20.5 | 54.7 | 37.4 | 21.9 | 18.4 | 28.2 | 21.7 | 13.8 | 11.9 | 41.9 | 22.8 | 11.4 | 8.7 |
| | (67.1-71.1) | (42.7-45.5) | (23.6-26.8) | (18.3-23.9) | (53.2-56.4) | (36.2-38.7) | (20.7-23.4) | (16.4-21.4) | (26.1-30.3) | (20.8-22.7) | (12.9-15.0) | (10.5-14.1) | (39.8-44.3) | (21.8-23.9) | (10.5-12.3) | (7.5-10.3) |
| South Asia | 131.0 | 93.5 | 49.4 | 33.5 | 90.0 | 69.4 | 41.3 | 29.1 | 58.9 | 45.7 | 29.5 | 21.1 | 76.6 | 50.1 | 20.6 | 12.7 |
| | (127.6-134.5) | (91.0-96.2) | (47.6-51.3) | (30.6-37.0) | (87.8-92.4) | (67.5-71.3) | (39.8-42.8) | (26.6-32.1) | (56.3-61.6) | (43.7-47.7) | (28.2-30.9) | (19.1-23.4) | (73.7-79.6) | (48.0-52.4) | (19.4-21.7) | (11.4-14.3) |
| East Asia and Pacific | 56.4 | 39.6 | 16.6 | 13.2 | 44.5 | 31.6 | 13.3 | 10.7 | 27.6 | 19.9 | 8.3 | 6.7 | 29.6 | 20.1 | 8.4 | 6.6 |
| | (53.7-59.5) | (38.4-41.0) | (16.0-17.4) | (12.1-15.0) | (42.3-47.0) | (30.6-32.7) | (12.8-14.0) | (9.8-12.2) | (25.5-29.9) | (18.9-21.0) | (7.9-8.8) | (6.0-7.7) | (27.6-31.9) | (19.1-21.2) | (8.0-8.9) | (5.9-7.6) |
| Latin America and Caribbean | 54.6 | 32.7 | 18.3 | 15.4 | 44.1 | 27.1 | 15.6 | 13.3 | 22.4 | 15.8 | 10.0 | 8.3 | 32.9 | 17.1 | 8.4 | 7.1 |
| | (53.0-56.3) | (31.9-33.6) | (17.8-18.9) | (14.3-17.2) | (42.8-45.5) | (26.4-27.9) | (15.2-16.2) | (12.4-14.8) | (21.2-23.7) | (15.0-16.8) | (9.7-10.4) | (7.3-9.7) | (31.5-34.4) | (16.3-18.0) | (8.0-8.8) | (6.1-8.5) |
| North America | 11.0 | 8.3 | 6.6 | 6.4 | 9.2 | 7.0 | 5.7 | 5.4 | 5.6 | 4.6 | 3.9 | 3.6 | 5.4 | 3.7 | 2.8 | 2.8 |
| | (10.8-11.2) | (8.1-8.4) | (6.5-6.7) | (6.1-6.6) | (9.0-9.3) | (6.9-7.1) | (5.6-5.7) | (5.2-5.6) | (5.5-5.8) | (4.5-4.7) | (3.8-3.9) | (3.5-3.8) | (5.2-5.5) | (3.6-3.8) | (2.7-2.9) | (2.6-2.9) |
| Europe and Central Asia | 30.8 | 20.9 | 9.6 | 7.8 | 25.6 | 17.5 | 8.1 | 6.6 | 13.8 | 10.2 | 5.1 | 4.1 | 17.2 | 10.9 | 4.5 | 3.6 |
| | (30.0-31.7) | (20.3-21.7) | (9.3-9.9) | (7.4-8.5) | (25.0-26.4) | (17.0-18.2) | (7.8-8.3) | (6.2-7.2) | (13.2-14.6) | (9.8-10.6) | (4.9-5.3) | (3.9-4.6) | (16.4-18.0) | (10.4-11.4) | (4.3-4.7) | (3.3-4.1) |
| Eastern Europe and Central Asia | 46.5 | 34.9 | 13.8 | 11.1 | 38.8 | 29.4 | 11.6 | 9.4 | 20.3 | 16.5 | 7.2 | 5.6 | 26.7 | 18.7 | 6.7 | 5.5 |
| | (45.1-48.1) | (33.7-36.4) | (13.4-14.3) | (10.3-12.4) | (37.6-40.2) | (28.3-30.6) | (11.3-12.0) | (8.8-10.5) | (19.2-21.6) | (15.8-17.4) | (6.8-7.6) | (5.1-6.4) | (25.3-28.1) | (17.8-19.7) | (6.3-7.0) | (4.9-6.3) |
| Western Europe | 10.4 | 6.2 | 4.0 | 3.8 | 8.8 | 5.1 | 3.3 | 3.2 | 5.5 | 3.5 | 2.4 | 2.4 | 5.0 | 2.8 | 1.7 | 1.4 |

| Region | Under-five mortality rate (U5MR) (per 1,000 live births) | | | | Infant mortality rate (IMR) (per 1,000 live births) | | | | Neonatal mortality rate (NMR) (per 1,000 live births) | | | | Mortality rate age 1–59 months (per 1,000 children aged 28 days) | | | |
|---|---|---|---|---|---|---|---|---|---|---|---|---|---|---|---|---|
| | 1990 | 2000 | 2015 | 2024 | 1990 | 2000 | 2015 | 2024 | 1990 | 2000 | 2015 | 2024 | 1990 | 2000 | 2015 | 2024 |
| | (10.4-10.5) | (6.2-6.2) | (4.0-4.0) | (3.7-3.9) | (8.7-8.8) | (5.1-5.1) | (3.3-3.4) | (3.2-3.3) | (5.4-5.6) | (3.4-3.5) | (2.3-2.4) | (2.3-2.4) | (4.9-5.1) | (2.7-2.8) | (1.6-1.7) | (1.4-1.5) |
| World | 93.5 | 76.7 | 42.9 | 37.4 | 64.0 | 53.3 | 31.5 | 27.7 | 36.6 | 30.3 | 19.4 | 17.2 | 59.1 | 47.8 | 23.9 | 20.5 |
| | (92.2-95.0) | (75.7-77.9) | (42.1-44.1) | (36.0-40.2) | (63.1-65.1) | (52.6-54.1) | (30.9-32.3) | (26.8-29.6) | (35.4-37.8) | (29.5-31.2) | (18.9-20.1) | (16.3-18.8) | (57.8-60.5) | (46.9-48.9) | (23.2-24.9) | (19.3-22.6) |

## 3.2. Table A.2: Global and regional deaths, 1990–2024

Number of under-five deaths, infant deaths, neonatal deaths, and deaths age 1–59 months (thousands) for 1990, 2000, 2015, and 2024, by UNICEF region and World. Values shown are medians with 90% uncertainty intervals in parentheses.

| Region | Number of under-five deaths (thousands) | | | | Number of infant deaths (thousands) | | | | Number of neonatal deaths (thousands) | | | | Number of deaths age 1 to 59 months (thousands) | | | |
|---|---|---|---|---|---|---|---|---|---|---|---|---|---|---|---|---|
| | 1990 | 2000 | 2015 | 2024 | 1990 | 2000 | 2015 | 2024 | 1990 | 2000 | 2015 | 2024 | 1990 | 2000 | 2015 | 2024 |
| Sub-Saharan Africa | 3,931 | 4,093 | 3,123 | 2,932 | 2,300 | 2,474 | 2,018 | 1,965 | 1,039 | 1,115 | 1,101 | 1,124 | 2,893 | 2,979 | 2,022 | 1,808 |
| | (3,850-4,019) | (4,014-4,185) | (3,022-3,274) | (2,782-3,240) | (2,255-2,350) | (2,428-2,527) | (1,958-2,107) | (1,867-2,164) | (996-1,090) | (1,072-1,164) | (1,057-1,163) | (1,033-1,287) | (2,819-2,968) | (2,910-3,055) | (1,940-2,140) | (1,675-2,027) |
| West and Central Africa | 2,062 | 2,275 | 1,963 | 1,918 | 1,120 | 1,297 | 1,182 | 1,206 | 529 | 595 | 635 | 656 | 1,533 | 1,680 | 1,327 | 1,263 |
| | (1,996-2,132) | (2,208-2,350) | (1,885-2,052) | (1,770-2,139) | (1,085-1,156) | (1,260-1,338) | (1,137-1,235) | (1,116-1,340) | (500-560) | (565-628) | (601-676) | (588-755) | (1,478-1,591) | (1,624-1,741) | (1,267-1,394) | (1,145-1,422) |
| Eastern and Southern Africa | 1,869 | 1,818 | 1,160 | 1,014 | 1,181 | 1,177 | 836 | 759 | 510 | 519 | 466 | 468 | 1,359 | 1,299 | 695 | 546 |
| | (1,823-1,919) | (1,775-1,870) | (1,092-1,277) | (931-1,200) | (1,151-1,213) | (1,149-1,211) | (792-906) | (698-891) | (486-539) | (496-547) | (440-507) | (417-568) | (1,319-1,401) | (1,262-1,340) | (639-786) | (484-661) |
| Middle East and North Africa | 590 | 344 | 267 | 204 | 468 | 293 | 233 | 183 | 244 | 172 | 148 | 119 | 346 | 172 | 118 | 84 |
| | (573-607) | (333-356) | (251-284) | (182-237) | (455-482) | (284-303) | (220-249) | (163-213) | (226-261) | (165-180) | (138-161) | (105-141) | (327-366) | (165-180) | (109-128) | (73-100) |
| South Asia | 4,988 | 3,750 | 1,839 | 1,196 | 3,434 | 2,789 | 1,528 | 1,039 | 2,283 | 1,868 | 1,100 | 756 | 2,705 | 1,882 | 739 | 440 |
| | (4,861-5,119) | (3,648-3,858) | (1,772-1,908) | (1,095-1,320) | (3,349-3,521) | (2,714-2,867) | (1,473-1,586) | (951-1,146) | (2,183-2,389) | (1,788-1,952) | (1,052-1,153) | (686-842) | (2,596-2,814) | (1,799-1,968) | (699-778) | (395-493) |
| East Asia and Pacific | 2,371 | 1,250 | 539 | 283 | 1,882 | 993 | 429 | 226 | 1,186 | 635 | 264 | 141 | 1,185 | 615 | 274 | 142 |
| | (2,258-2,498) | (1,211-1,296) | (518-563) | (260-320) | (1,790-1,984) | (962-1,029) | (413-449) | (206-257) | (1,096-1,285) | (603-670) | (250-280) | (126-163) | (1,101-1,279) | (586-648) | (260-290) | (128-163) |
| Latin America and Caribbean | 644 | 377 | 192 | 143 | 520 | 312 | 164 | 123 | 267 | 183 | 105 | 77 | 377 | 194 | 87 | 66 |
| | (626-665) | (367-388) | (187-199) | (133-159) | (505-537) | (304-321) | (160-170) | (115-137) | (253-282) | (173-193) | (101-109) | (68-90) | (361-395) | (184-204) | (84-92) | (57-78) |
| North America | 49 | 35 | 29 | 26 | 41 | 30 | 25 | 22 | 26 | 20 | 17 | 15 | 23 | 15 | 12 | 11 |
| | (48-50) | (34-36) | (29-29) | (25-27) | (41-42) | (29-30) | (24-25) | (21-23) | (25-26) | (19-20) | (16-17) | (14-15) | (23-24) | (15-16) | (12-12) | (11-12) |
| Europe and Central Asia | 397 | 214 | 108 | 74 | 329 | 178 | 92 | 63 | 176 | 103 | 58 | 39 | 220 | 112 | 50 | 35 |

| Region | Number of under-five deaths (thousands) | | | | Number of infant deaths (thousands) | | | | Number of neonatal deaths (thousands) | | | | Number of deaths age 1 to 59 months (thousands) | | | |
|---|---|---|---|---|---|---|---|---|---|---|---|---|---|---|---|---|
| | 1990 | 2000 | 2015 | 2024 | 1990 | 2000 | 2015 | 2024 | 1990 | 2000 | 2015 | 2024 | 1990 | 2000 | 2015 | 2024 |
| | (387-408) | (208-222) | (106-112) | (71-81) | (320-338) | (173-185) | (89-94) | (59-68) | (168-186) | (99-108) | (56-61) | (36-43) | (211-231) | (107-117) | (48-53) | (32-39) |
| Eastern Europe and Central Asia | 339 | 184 | 89 | 58 | 280 | 153 | 75 | 49 | 146 | 86 | 47 | 29 | 193 | 98 | 42 | 29 |
| | (329-350) | (177-191) | (86-92) | (54-65) | (272-290) | (148-160) | (73-78) | (46-54) | (137-155) | (82-90) | (44-49) | (26-33) | (183-203) | (93-103) | (40-44) | (26-33) |
| Western Europe | 58 | 31 | 20 | 16 | 49 | 25 | 16 | 14 | 31 | 17 | 12 | 10 | 27 | 13 | 8 | 6 |
| | (58-59) | (30-31) | (20-20) | (16-17) | (49-49) | (25-25) | (16-17) | (14-14) | (30-31) | (17-17) | (11-12) | (10-10) | (27-28) | (13-14) | (8-8) | (6-7) |
| World | 12,970 | 10,063 | 6,096 | 4,858 | 8,975 | 7,068 | 4,490 | 3,620 | 5,220 | 4,095 | 2,793 | 2,271 | 7,750 | 5,968 | 3,303 | 2,587 |
| | (12,787-13,176) | (9,930-10,217) | (5,980-6,269) | (4,689-5,210) | (8,846-9,123) | (6,977-7,174) | (4,410-4,603) | (3,501-3,865) | (5,059-5,401) | (3,983-4,217) | (2,723-2,888) | (2,148-2,488) | (7,570-7,934) | (5,846-6,104) | (3,208-3,435) | (2,433-2,836) |

## 3.3. Table A.3: Global and regional annual rate of reduction

Annual rate of reduction (ARR, %) for under-five mortality rate (U5MR), infant mortality rate (IMR), neonatal mortality rate (NMR), and mortality rate age 1–59 months for the periods 1990-2000, 2000-2015, and 2015-2024, by UNICEF region and World. Values shown are medians with 90% uncertainty intervals in parentheses.

| Region | Annual rate of reduction in under-five mortality rate (%) | | | Annual rate of reduction in infant mortality rate (%) | | | Annual rate of reduction in neonatal mortality rate (%) | | | Annual rate of reduction in mortality rate age 1–59 months (%) | | |
|---|---|---|---|---|---|---|---|---|---|---|---|---|
| | 1990-2000 | 2000-2015 | 2015-2024 | 1990-2000 | 2000-2015 | 2015-2024 | 1990-2000 | 2000-2015 | 2015-2024 | 1990-2000 | 2000-2015 | 2015-2024 |
| Sub-Saharan Africa | 1.7 | 3.8 | 2.0 | 1.4 | 3.3 | 1.6 | 1.4 | 1.9 | 1.0 | 1.9 | 4.7 | 2.5 |
| | (1.5-1.9) | (3.5-4) | (1-2.6) | (1.2-1.6) | (3-3.5) | (0.7-2.1) | (1.1-1.7) | (1.6-2.2) | (-0.2-1.9) | (1.6-2.1) | (4.3-4.9) | (1.4-3.3) |
| West and Central Africa | 1.4 | 3.3 | 1.3 | 0.9 | 2.9 | 0.9 | 1.3 | 1.7 | 0.7 | 1.5 | 4.0 | 1.6 |
| | (1.1-1.7) | (3.1-3.6) | (0.2-2.2) | (0.7-1.2) | (2.6-3.2) | (-0.2-1.7) | (0.8-1.7) | (1.3-2.1) | (-0.7-1.9) | (1.2-1.8) | (3.7-4.3) | (0.4-2.6) |
| Eastern and Southern Africa | 2.1 | 4.6 | 3.0 | 1.9 | 3.9 | 2.6 | 1.6 | 2.2 | 1.4 | 2.4 | 5.9 | 4.2 |
| | (1.9-2.4) | (4-5) | (1.6-3.8) | (1.6-2.1) | (3.4-4.2) | (1.2-3.3) | (1.1-2.1) | (1.7-2.7) | (-0.2-2.5) | (2.1-2.7) | (5.1-6.4) | (2.5-5.3) |
| Middle East and North Africa | 4.5 | 3.8 | 2.2 | 3.8 | 3.6 | 2.0 | 2.6 | 3.0 | 1.7 | 6.1 | 4.6 | 3.0 |
| | (4.2-4.8) | (3.3-4.2) | (0.8-3.2) | (3.5-4.1) | (3.1-4) | (0.5-3) | (1.7-3.4) | (2.4-3.5) | (0.1-2.8) | (5.4-6.8) | (4.1-5.2) | (1.4-4.3) |
| South Asia | 3.4 | 4.3 | 4.3 | 2.6 | 3.5 | 3.9 | 2.5 | 2.9 | 3.7 | 4.2 | 5.9 | 5.4 |
| | (3.1-3.7) | (4-4.6) | (3.3-5.3) | (2.3-2.9) | (3.2-3.8) | (2.8-4.9) | (2-3) | (2.5-3.3) | (2.6-4.8) | (3.8-4.7) | (5.5-6.4) | (4.1-6.5) |
| East Asia and Pacific | 3.5 | 5.8 | 2.6 | 3.4 | 5.7 | 2.4 | 3.3 | 5.8 | 2.4 | 3.9 | 5.8 | 2.7 |
| | (2.9-4.2) | (5.4-6.1) | (1.3-3.5) | (2.8-4.1) | (5.4-6.1) | (1.2-3.4) | (2.3-4.2) | (5.4-6.3) | (1-3.5) | (3-4.8) | (5.3-6.3) | (1.3-3.8) |
| Latin America and Caribbean | 5.1 | 3.9 | 1.9 | 4.9 | 3.7 | 1.8 | 3.5 | 3.1 | 2.1 | 6.5 | 4.8 | 1.8 |
| | (4.8-5.4) | (3.6-4.1) | (0.9-2.7) | (4.5-5.2) | (3.4-3.9) | (0.7-2.5) | (2.8-4.1) | (2.6-3.5) | (0.5-3.4) | (5.9-7.1) | (4.3-5.2) | (0.1-3.4) |
| North America | 2.8 | 1.5 | 0.4 | 2.7 | 1.4 | 0.5 | 2.1 | 1.1 | 0.7 | 3.6 | 1.9 | 0.1 |
| | (2.6-3.1) | (1.3-1.6) | (0-0.9) | (2.5-3) | (1.3-1.5) | (0-1) | (1.9-2.4) | (1-1.3) | (0.1-1.2) | (3.4-3.9) | (1.8-2.1) | (-0.5-0.7) |
| Europe and Central Asia | 3.9 | 5.2 | 2.3 | 3.8 | 5.2 | 2.2 | 3.1 | 4.6 | 2.3 | 4.6 | 5.9 | 2.4 |
| | (3.5-4.2) | (4.9-5.5) | (1.4-2.9) | (3.5-4.1) | (4.9-5.5) | (1.3-2.8) | (2.5-3.7) | (4.2-4.9) | (1.2-3.1) | (4-5.1) | (5.5-6.3) | (1.1-3.3) |

| Region | Annual rate of reduction in under-five mortality rate (%) | | | Annual rate of reduction in infant mortality rate (%) | | | Annual rate of reduction in neonatal mortality rate (%) | | | Annual rate of reduction in mortality rate age 1–59 months (%) | | |
|---|---|---|---|---|---|---|---|---|---|---|---|---|
| | 1990-2000 | 2000-2015 | 2015-2024 | 1990-2000 | 2000-2015 | 2015-2024 | 1990-2000 | 2000-2015 | 2015-2024 | 1990-2000 | 2000-2015 | 2015-2024 |
| Eastern Europe and Central Asia | 2.9 | 6.2 | 2.5 | 2.8 | 6.2 | 2.4 | 2.1 | 5.6 | 2.7 | 3.6 | 6.9 | 2.2 |
| | (2.5-3.3) | (5.9-6.5) | (1.2-3.3) | (2.4-3.2) | (5.9-6.5) | (1.2-3.1) | (1.3-2.8) | (5.1-6) | (1.3-3.7) | (2.9-4.2) | (6.4-7.3) | (0.8-3.3) |
| Western Europe | 5.2 | 2.9 | 0.6 | 5.4 | 2.8 | 0.5 | 4.6 | 2.5 | 0.0 | 5.9 | 3.4 | 1.6 |
| | (5.1-5.3) | (2.8-3) | (0.4-0.8) | (5.3-5.5) | (2.8-2.9) | (0.3-0.7) | (4.5-4.8) | (2.4-2.6) | (-0.3-0.3) | (5.8-6) | (3.3-3.5) | (1.2-1.9) |
| World | 2.0 | 3.9 | 1.5 | 1.8 | 3.5 | 1.4 | 1.9 | 3.0 | 1.3 | 2.1 | 4.6 | 1.7 |
| | (1.8-2.2) | (3.7-4) | (0.8-1.9) | (1.7-2) | (3.3-3.6) | (0.8-1.8) | (1.6-2.2) | (2.7-3.2) | (0.5-1.9) | (1.9-2.3) | (4.4-4.8) | (0.8-2.3) |

## 3.4. Table A.4: Country mortality rates, 1990–2024

Under-five mortality rate (U5MR), infant mortality rate (IMR), neonatal mortality rate (NMR), and mortality rate age 1–59 months (deaths per 1,000 live births) for 1990, 2000, 2015, and 2024, by country. Values shown are medians with 90% uncertainty intervals in parentheses.

| Country | Under-five mortality rate (U5MR) (per 1,000 live births) | | | | Infant mortality rate (IMR) (per 1,000 live births) | | | | Neonatal mortality rate (NMR) (per 1,000 live births) | | | | Mortality rate age 1–59 months (per 1,000 children aged 28 days) | | | |
|---|---|---|---|---|---|---|---|---|---|---|---|---|---|---|---|---|
| | 1990 | 2000 | 2015 | 2024 | 1990 | 2000 | 2015 | 2024 | 1990 | 2000 | 2015 | 2024 | 1990 | 2000 | 2015 | 2024 |
| Afghanistan | 181.4 | 131.7 | 71.9 | 53.0 | 145.9 | 110.6 | 64.0 | 48.3 | 75.8 | 62.2 | 41.5 | 33.1 | 114.2 | 74.2 | 31.7 | 20.6 |
| | (164.9-199.6) | (122.1-142.4) | (63.7-80.7) | (39.7-71.4) | (132.6-160.5) | (102.5-119.6) | (56.7-71.9) | (36.1-65.0) | (67.4-85.3) | (56.5-68.4) | (36.4-47.2) | (24.5-44.9) | (102.0-128.0) | (67.5-81.7) | (27.4-36.3) | (14.9-28.8) |
| Albania | 40.6 | 27.0 | 9.6 | 9.2 | 27.3 | 17.8 | 7.6 | 8.3 | 12.5 | 12.1 | 6.0 | 6.7 | 28.3 | 15.0 | 3.6 | 2.5 |
| | (36.0-45.8) | (24.5-29.7) | (9.2-10.0) | (7.8-10.9) | (24.2-30.8) | (16.2-19.6) | (7.4-7.9) | (7.0-9.7) | (9.7-15.7) | (9.7-14.7) | (5.5-6.6) | (5.3-8.3) | (24.2-33.0) | (12.5-17.8) | (3.1-4.1) | (1.6-3.8) |
| Algeria | 51.6 | 41.7 | 24.9 | 21.6 | 39.8 | 34.8 | 21.9 | 19.5 | 24.2 | 22.6 | 15.2 | 15.0 | 28.0 | 19.5 | 9.9 | 6.8 |
| | (47.6-55.6) | (39.2-44.3) | (23.5-26.4) | (19.0-24.6) | (36.7-42.8) | (32.7-36.9) | (20.7-23.3) | (17.1-22.2) | (21.6-27.1) | (20.4-25.0) | (14.2-16.3) | (13.1-17.1) | (25.1-31.1) | (17.4-21.7) | (9.1-10.7) | (5.8-7.9) |
| Andorra | 13.0 | 7.6 | 3.5 | 2.5 | 9.1 | 6.6 | 3.3 | 2.4 | 6.3 | 3.5 | 1.8 | 1.2 | 6.4 | 4.0 | 1.7 | 1.2 |
| | (3.2-51.9) | (3.8-15.2) | (1.2-10.6) | (0.6-10.1) | (2.2-36.3) | (3.3-13.2) | (1.1-10.1) | (0.6-9.8) | (1.5-26.4) | (1.6-7.3) | (0.6-5.4) | (0.3-5.3) | (1.5-26.9) | (1.9-8.5) | (0.6-5.3) | (0.2-5.1) |
| Angola | 212.7 | 185.0 | 61.8 | 49.0 | 118.1 | 108.3 | 38.8 | 32.1 | 45.8 | 39.1 | 20.9 | 18.1 | 174.0 | 151.7 | 41.8 | 31.4 |
| | (188.5-240.6) | (162.8-210.0) | (49.9-76.2) | (33.3-75.8) | (104.7-133.6) | (95.4-123.0) | (31.3-47.8) | (21.9-49.7) | (37.0-60.9) | (32.3-46.5) | (16.2-26.4) | (11.5-29.2) | (151.1-199.5) | (132.6-173.2) | (33.3-52.2) | (20.8-49.5) |
| Anguilla | 19.2 | 12.0 | 7.4 | 5.6 | 16.3 | 10.7 | 6.8 | 5.2 | 10.3 | 6.4 | 3.9 | 2.9 | 8.8 | 5.4 | 3.4 | 2.5 |
| | (13.3-27.6) | (8.2-17.3) | (4.4-12.9) | (2.7-12.0) | (11.3-23.5) | (7.4-15.5) | (4.0-11.8) | (2.5-11.1) | (6.5-15.8) | (3.8-10.2) | (1.9-7.7) | (1.2-7.0) | (5.4-13.9) | (3.1-9.0) | (1.5-6.7) | (0.9-6.1) |
| Antigua and Barbuda | 13.6 | 14.9 | 11.0 | 9.1 | 11.1 | 12.1 | 8.6 | 7.0 | 8.5 | 11.0 | 6.5 | 5.2 | 5.1 | 3.9 | 4.5 | 3.8 |
| | (12.0-15.3) | (13.4-16.7) | (9.6-12.7) | (6.4-13.0) | (9.8-12.5) | (10.9-13.6) | (7.4-9.9) | (5.0-10.1) | (7.3-9.9) | (9.7-12.4) | (5.5-7.7) | (3.5-7.7) | (4.1-6.2) | (3.2-4.8) | (3.7-5.5) | (2.5-5.9) |
| Argentina | 28.8 | 19.4 | 11.7 | 9.5 | 24.8 | 16.7 | 10.0 | 8.0 | 14.9 | 11.0 | 6.8 | 5.1 | 14.1 | 8.4 | 5.0 | 4.5 |
| | (28.2-29.3) | (19.0-19.7) | (11.4-12.1) | (8.4-10.8) | (24.4-25.3) | (16.4-17.0) | (9.7-10.4) | (7.1-9.1) | (14.5-15.4) | (10.7-11.4) | (6.5-7.1) | (4.4-5.8) | (13.6-14.5) | (8.1-8.7) | (4.8-5.2) | (3.9-5.2) |
| Armenia | 48.7 | 30.6 | 14.7 | 9.6 | 45.6 | 27.8 | 12.8 | 8.5 | 22.7 | 16.0 | 7.7 | 5.1 | 26.6 | 14.8 | 6.9 | 4.5 |
| | (44.0-54.1) | (27.3-33.8) | (12.4-16.9) | (7.7-12.3) | (41.1-50.6) | (24.8-30.7) | (10.8-14.8) | (6.8-10.9) | (19.6-26.5) | (13.8-18.4) | (5.9-9.9) | (3.5-7.1) | (22.9-30.4) | (12.6-17.0) | (5.1-9.0) | (3.0-6.5) |
| Australia | 9.2 | 6.2 | 3.9 | 3.6 | 7.6 | 5.1 | 3.3 | 3.1 | 4.6 | 3.5 | 2.4 | 2.3 | 4.6 | 2.7 | 1.5 | 1.3 |

| | Under-five mortality rate (U5MR) (per 1,000 live births) | | | | Infant mortality rate (IMR) (per 1,000 live births) | | | | Neonatal mortality rate (NMR) (per 1,000 live births) | | | | Mortality rate age 1–59 months (per 1,000 children aged 28 days) | | | |
|---|---|---|---|---|---|---|---|---|---|---|---|---|---|---|---|---|
| **Country** | **1990** | **2000** | **2015** | **2024** | **1990** | **2000** | **2015** | **2024** | **1990** | **2000** | **2015** | **2024** | **1990** | **2000** | **2015** | **2024** |
| | (9.0-9.4) | (6.1-6.3) | (3.8-4.0) | (3.4-3.8) | (7.4-7.7) | (5.0-5.2) | (3.2-3.4) | (2.9-3.3) | (4.5-4.8) | (3.4-3.6) | (2.3-2.4) | (2.1-2.4) | (4.4-4.8) | (2.6-2.8) | (1.5-1.6) | (1.2-1.4) |
| Austria | 9.5 | 5.5 | 3.7 | 3.3 | 8.0 | 4.6 | 3.1 | 2.8 | 4.6 | 3.1 | 2.3 | 2.2 | 4.9 | 2.4 | 1.4 | 1.1 |
| | (9.3-9.8) | (5.3-5.7) | (3.6-3.9) | (3.1-3.6) | (7.8-8.2) | (4.4-4.7) | (3.0-3.2) | (2.6-3.0) | (4.4-4.8) | (2.9-3.2) | (2.1-2.4) | (2.0-2.4) | (4.7-5.2) | (2.3-2.6) | (1.3-1.5) | (1.0-1.3) |
| Azerbaijan | 95.4 | 74.3 | 27.9 | 17.9 | 75.1 | 61.1 | 21.1 | 12.5 | 29.5 | 34.7 | 16.0 | 12.4 | 67.8 | 41.0 | 12.1 | 5.5 |
| | (85.7-106.2) | (66.0-83.9) | (22.0-35.2) | (11.4-28.2) | (67.5-83.6) | (54.2-68.9) | (16.6-26.6) | (8.0-19.7) | (24.6-35.3) | (29.2-40.8) | (11.9-20.8) | (7.9-19.8) | (59.8-76.7) | (34.8-47.8) | (8.9-16.4) | (3.4-8.8) |
| Bahamas | 23.3 | 16.2 | 14.0 | 12.4 | 19.6 | 13.7 | 12.3 | 11.1 | 13.5 | 7.9 | 8.7 | 9.0 | 10.0 | 8.4 | 5.3 | 3.4 |
| | (22.2-24.6) | (15.2-17.2) | (13.0-15.0) | (9.7-16.0) | (18.7-20.6) | (12.9-14.6) | (11.5-13.2) | (8.7-14.3) | (12.3-14.7) | (7.1-8.7) | (7.8-9.5) | (6.9-11.8) | (8.9-11.2) | (7.6-9.2) | (4.7-6.1) | (2.3-4.8) |
| Bahrain | 23.0 | 12.5 | 7.7 | 8.8 | 20.0 | 10.5 | 6.3 | 7.3 | 15.4 | 5.0 | 3.4 | 4.4 | 7.7 | 7.6 | 4.3 | 4.3 |
| | (22.2-23.9) | (11.9-13.1) | (7.3-8.1) | (7.8-9.8) | (19.3-20.7) | (10.0-11.0) | (6.0-6.7) | (6.5-8.2) | (14.4-16.5) | (4.5-5.5) | (3.1-3.7) | (3.8-5.1) | (6.8-8.6) | (7.0-8.1) | (4.0-4.7) | (3.7-5.0) |
| Bangladesh | 146.5 | 85.5 | 38.5 | 30.5 | 96.3 | 59.8 | 29.8 | 24.4 | 65.7 | 43.4 | 23.5 | 17.9 | 86.5 | 44.0 | 15.4 | 12.8 |
| | (141.6-151.7) | (82.6-88.4) | (36.9-40.3) | (28.6-33.1) | (93.1-99.8) | (57.8-61.8) | (28.6-31.2) | (22.9-26.5) | (62.0-69.5) | (41.2-45.6) | (22.3-24.8) | (16.3-19.8) | (82.2-90.9) | (41.8-46.4) | (14.3-16.5) | (11.5-14.5) |
| Barbados | 18.1 | 14.4 | 12.8 | 9.8 | 16.2 | 13.0 | 11.7 | 9.1 | 11.8 | 9.0 | 8.8 | 6.6 | 6.4 | 5.4 | 4.0 | 3.1 |
| | (17.0-19.3) | (13.3-15.5) | (10.5-15.5) | (6.0-16.1) | (15.2-17.3) | (12.0-14.0) | (9.6-14.3) | (5.6-14.9) | (10.9-12.8) | (8.1-9.9) | (7.0-10.8) | (4.0-11.2) | (5.7-7.2) | (4.7-6.2) | (3.0-5.3) | (1.7-5.6) |
| Belarus | 15.2 | 12.8 | 4.1 | 2.3 | 12.1 | 9.9 | 3.1 | 1.8 | 10.0 | 5.2 | 1.6 | 0.7 | 5.3 | 7.6 | 2.5 | 1.6 |
| | (14.9-15.5) | (12.5-13.1) | (3.9-4.2) | (1.8-3.0) | (11.9-12.4) | (9.7-10.2) | (3.0-3.2) | (1.4-2.3) | (7.6-12.4) | (4.1-6.4) | (1.2-1.9) | (0.4-1.3) | (2.9-7.7) | (6.4-8.7) | (2.1-2.9) | (1.1-2.2) |
| Belgium | 10.0 | 5.9 | 4.1 | 3.7 | 8.3 | 4.8 | 3.4 | 3.1 | 4.6 | 3.0 | 2.3 | 2.2 | 5.4 | 2.9 | 1.8 | 1.5 |
| | (9.8-10.2) | (5.7-6.0) | (4.0-4.2) | (3.5-3.9) | (8.2-8.5) | (4.7-5.0) | (3.3-3.5) | (3.0-3.3) | (4.4-4.8) | (2.8-3.1) | (2.2-2.4) | (2.1-2.4) | (5.2-5.6) | (2.8-3.0) | (1.7-1.9) | (1.4-1.6) |
| Belize | 39.4 | 24.5 | 15.6 | 12.7 | 31.0 | 19.6 | 13.1 | 10.8 | 18.8 | 12.1 | 10.5 | 9.2 | 21.0 | 12.5 | 5.2 | 3.5 |
| | (34.6-44.7) | (22.9-26.1) | (14.8-16.5) | (11.1-14.5) | (27.3-35.2) | (18.4-20.9) | (12.4-13.8) | (9.4-12.3) | (15.7-22.4) | (10.9-13.4) | (9.7-11.4) | (7.9-10.7) | (17.6-24.8) | (11.2-13.8) | (4.6-5.9) | (2.8-4.3) |
| Benin | 171.2 | 135.0 | 96.1 | 74.7 | 98.1 | 79.7 | 57.0 | 45.2 | 44.5 | 38.2 | 31.4 | 27.2 | 132.5 | 100.6 | 66.8 | 48.8 |
| | (160.3-183.2) | (126.0-144.4) | (86.0-107.3) | (55.1-102.1) | (91.8-105.0) | (74.4-85.3) | (51.0-63.7) | (33.3-61.7) | (40.1-49.3) | (34.3-42.3) | (26.9-36.3) | (19.2-38.1) | (123.3-142.7) | (93.1-108.6) | (59.0-75.5) | (35.2-68.2) |
| Bhutan | 128.0 | 77.7 | 29.2 | 17.2 | 95.4 | 59.4 | 22.9 | 13.7 | 42.3 | 31.0 | 14.1 | 7.3 | 89.0 | 47.9 | 15.3 | 9.8 |
| | (110.6-150.3) | (69.3-86.9) | (23.9-36.1) | (12.0-24.3) | (82.5-112.1) | (53.0-66.4) | (18.7-28.3) | (9.5-19.3) | (28.9-64.0) | (24.9-38.3) | (10.2-18.7) | (4.0-11.5) | (67.6-110.1) | (40.2-56.0) | (11.5-20.3) | (6.2-15.2) |
| Bolivia (Plurinational State of) | 117.8 | 70.7 | 26.7 | 15.7 | 89.0 | 57.2 | 23.5 | 14.2 | 40.1 | 27.9 | 12.8 | 7.3 | 81.0 | 44.0 | 14.0 | 8.4 |
| | (111.1-125.1) | (66.1-75.7) | (23.1-30.8) | (11.7-21.2) | (83.9-94.5) | (53.4-61.2) | (20.3-27.1) | (10.6-19.2) | (36.7-43.7) | (25.3-30.6) | (10.9-15.1) | (5.3-10.1) | (75.6-86.8) | (40.6-47.8) | (11.9-16.4) | (6.1-11.5) |
| Bosnia and Herzegovina | 18.2 | 9.5 | 6.7 | 6.9 | 16.1 | 8.3 | 5.9 | 5.5 | 11.3 | 6.5 | 4.8 | 4.4 | 6.9 | 3.0 | 2.0 | 2.5 |
| | (17.7-18.7) | (9.2-9.9) | (6.4-7.1) | (6.2-7.7) | (15.7-16.5) | (8.1-8.6) | (5.6-6.2) | (4.9-6.1) | (10.9-11.8) | (6.2-6.9) | (4.5-5.0) | (3.9-5.0) | (6.5-7.4) | (2.8-3.3) | (1.8-2.2) | (2.1-2.9) |
| Botswana | 51.5 | 79.8 | 46.7 | 33.3 | 40.0 | 39.4 | 44.2 | 32.1 | 21.6 | 9.1 | 22.0 | 17.6 | 30.5 | 71.5 | 24.8 | 15.3 |
| | (42.1-64.6) | (58.5-118.2) | (25.5-100.2) | (9.6-111.0) | (32.7-50.1) | (28.9-58.4) | (24.1-94.7) | (9.2-106.9) | (17.3-27.5) | (6.5-13.7) | (11.3-49.1) | (4.8-59.5) | (24.6-38.9) | (52.1-105.9) | (12.9-56.7) | (4.1-55.2) |
| Brazil | 62.8 | 34.4 | 16.0 | 14.2 | 51.9 | 29.1 | 13.7 | 12.3 | 25.1 | 18.1 | 9.5 | 7.1 | 38.8 | 16.7 | 6.5 | 7.1 |
| | (58.5-67.7) | (32.2-36.8) | (15.4-16.5) | (12.2-16.6) | (48.4-55.9) | (27.2-31.2) | (13.3-14.2) | (10.5-14.3) | (22.0-28.4) | (15.7-20.6) | (9.0-10.0) | (6.0-8.5) | (35.0-42.8) | (14.3-19.2) | (6.1-6.9) | (6.0-8.5) |

| Country | Under-five mortality rate (U5MR) (per 1,000 live births) | | | | Infant mortality rate (IMR) (per 1,000 live births) | | | | Neonatal mortality rate (NMR) (per 1,000 live births) | | | | Mortality rate age 1–59 months (per 1,000 children aged 28 days) | | | |
|---|---|---|---|---|---|---|---|---|---|---|---|---|---|---|---|---|
| | 1990 | 2000 | 2015 | 2024 | 1990 | 2000 | 2015 | 2024 | 1990 | 2000 | 2015 | 2024 | 1990 | 2000 | 2015 | 2024 |
| British Virgin Islands | 22.9 | 16.8 | 15.5 | 12.4 | 19.2 | 14.6 | 14.1 | 11.4 | 12.3 | 9.0 | 8.2 | 6.4 | 10.7 | 7.8 | 7.1 | 5.6 |
| | (19.3-27.5) | (13.8-20.4) | (10.4-23.4) | (6.5-23.4) | (16.2-23.0) | (12.0-17.7) | (9.4-21.3) | (6.0-21.5) | (8.8-16.4) | (5.8-12.6) | (4.3-14.0) | (2.8-13.9) | (7.4-14.6) | (4.8-11.4) | (3.5-12.7) | (2.3-12.5) |
| Brunei Darussalam | 13.3 | 10.3 | 10.7 | 9.9 | 10.3 | 8.3 | 9.0 | 8.6 | 6.0 | 5.0 | 5.1 | 5.7 | 7.4 | 5.3 | 5.6 | 4.3 |
| | (12.5-14.1) | (9.7-11.0) | (10.0-11.4) | (8.7-11.3) | (9.7-10.9) | (7.8-8.8) | (8.4-9.6) | (7.6-9.7) | (5.3-6.7) | (4.5-5.5) | (4.6-5.7) | (4.9-6.6) | (6.6-8.1) | (4.8-5.9) | (5.0-6.1) | (3.6-5.1) |
| Bulgaria | 18.4 | 17.5 | 8.2 | 5.7 | 14.6 | 14.3 | 6.8 | 4.6 | 8.0 | 7.9 | 4.1 | 2.6 | 10.5 | 9.7 | 4.1 | 3.2 |
| | (18.0-18.8) | (17.0-17.9) | (7.9-8.4) | (5.3-6.2) | (14.3-15.0) | (14.0-14.7) | (6.6-7.0) | (4.3-5.0) | (7.7-8.3) | (7.5-8.2) | (3.9-4.3) | (2.4-2.8) | (10.1-10.8) | (9.3-10.1) | (3.9-4.3) | (2.9-3.4) |
| Burkina Faso | 196.6 | 176.0 | 100.8 | 74.9 | 102.8 | 92.4 | 56.3 | 43.6 | 45.2 | 40.1 | 28.1 | 23.8 | 158.4 | 141.6 | 74.6 | 51.8 |
| | (182.5-211.0) | (163.5-189.7) | (83.6-122.2) | (46.8-119.1) | (95.5-110.4) | (85.9-99.6) | (46.7-68.3) | (27.3-69.4) | (39.8-51.0) | (35.1-45.6) | (20.2-37.7) | (13.4-41.0) | (146.0-171.2) | (130.4-153.7) | (60.1-92.4) | (31.2-84.8) |
| Burundi | 168.6 | 152.4 | 66.9 | 47.1 | 92.2 | 88.4 | 41.8 | 30.5 | 39.1 | 36.3 | 23.5 | 19.1 | 134.7 | 120.5 | 44.4 | 28.4 |
| | (150.7-188.6) | (138.1-168.7) | (50.8-87.8) | (26.7-81.5) | (82.4-103.1) | (80.2-97.9) | (31.7-54.9) | (17.3-52.8) | (33.4-45.4) | (31.3-41.7) | (17.2-31.4) | (10.6-33.5) | (119.4-151.8) | (108.3-134.4) | (33.4-59.4) | (15.8-50.4) |
| Cabo Verde | 56.4 | 36.0 | 19.2 | 11.1 | 42.0 | 29.4 | 17.6 | 10.6 | 18.7 | 18.1 | 12.3 | 8.0 | 38.3 | 18.2 | 6.9 | 3.1 |
| | (54.3-58.5) | (34.9-37.1) | (18.2-20.1) | (9.6-13.0) | (40.5-43.6) | (28.5-30.2) | (16.8-18.5) | (9.1-12.4) | (16.1-21.6) | (15.7-20.5) | (11.3-13.3) | (6.7-9.6) | (35.4-41.3) | (15.8-20.6) | (6.1-7.8) | (2.3-4.1) |
| Cambodia | 116.3 | 105.2 | 29.6 | 18.4 | 88.8 | 85.8 | 25.8 | 16.4 | 40.3 | 35.2 | 15.4 | 9.5 | 79.1 | 72.5 | 14.4 | 8.9 |
| | (106.9-126.3) | (96.0-115.1) | (22.6-38.6) | (8.6-39.0) | (81.6-96.4) | (78.3-93.8) | (19.7-33.7) | (7.7-34.6) | (36.0-45.0) | (30.9-39.6) | (11.5-20.5) | (4.4-20.4) | (71.8-86.6) | (65.6-80.4) | (10.7-19.3) | (4.1-19.3) |
| Cameroon | 137.4 | 143.9 | 88.5 | 64.8 | 81.5 | 83.1 | 53.3 | 40.2 | 40.4 | 35.4 | 29.5 | 24.8 | 101.2 | 112.6 | 60.8 | 40.9 |
| | (127.3-148.8) | (132.9-156.3) | (79.4-99.1) | (46.1-89.8) | (75.5-88.2) | (76.7-90.2) | (47.8-59.6) | (28.6-55.7) | (36.2-44.9) | (31.5-39.6) | (25.4-34.2) | (17.0-35.8) | (92.9-110.3) | (103.3-123.2) | (53.8-68.8) | (28.3-58.0) |
| Canada | 8.3 | 6.2 | 5.3 | 5.4 | 6.8 | 5.3 | 4.6 | 4.7 | 4.4 | 3.7 | 3.5 | 3.4 | 3.9 | 2.5 | 1.8 | 2.0 |
| | (8.1-8.4) | (6.1-6.3) | (5.3-5.4) | (5.2-5.7) | (6.7-6.9) | (5.2-5.4) | (4.6-4.7) | (4.5-4.9) | (4.2-4.5) | (3.6-3.8) | (3.4-3.6) | (3.2-3.6) | (3.8-4.1) | (2.4-2.6) | (1.7-1.9) | (1.9-2.2) |
| Central African Republic | 178.6 | 165.5 | 113.7 | 89.7 | 109.0 | 99.6 | 72.0 | 58.8 | 50.9 | 42.9 | 33.9 | 29.9 | 134.3 | 127.9 | 82.5 | 61.2 |
| | (160.1-199.2) | (149.9-184.1) | (97.1-132.9) | (64.8-123.1) | (97.7-121.6) | (90.2-110.8) | (61.5-84.1) | (42.5-80.7) | (43.9-59.7) | (36.8-49.7) | (27.3-41.7) | (20.4-44.0) | (119.2-151.9) | (114.7-143.6) | (69.3-98.0) | (43.4-86.2) |
| Chad | 211.4 | 182.4 | 128.7 | 97.3 | 116.8 | 101.7 | 72.1 | 56.3 | 51.6 | 43.7 | 35.4 | 30.4 | 168.6 | 145.0 | 96.3 | 68.7 |
| | (194.7-229.3) | (168.0-198.3) | (108.0-151.7) | (63.3-147.6) | (107.6-126.7) | (93.7-110.5) | (60.5-84.9) | (36.6-85.4) | (45.3-58.8) | (38.3-49.6) | (27.6-44.4) | (18.5-49.0) | (153.6-184.2) | (132.3-159.0) | (79.7-115.6) | (43.4-107.8) |
| Chile | 19.0 | 10.9 | 7.9 | 6.8 | 15.9 | 9.2 | 6.8 | 5.8 | 8.6 | 5.7 | 5.2 | 4.7 | 10.6 | 5.2 | 2.7 | 2.1 |
| | (18.6-19.5) | (10.6-11.2) | (7.7-8.1) | (6.5-7.1) | (15.6-16.3) | (8.9-9.4) | (6.6-7.0) | (5.6-6.1) | (8.2-8.9) | (5.5-5.9) | (5.1-5.4) | (4.5-4.9) | (10.2-10.9) | (5.0-5.4) | (2.6-2.8) | (1.9-2.2) |
| China | 53.6 | 36.6 | 10.7 | 5.7 | 43.3 | 29.9 | 8.2 | 4.1 | 29.6 | 21.4 | 5.3 | 2.6 | 24.7 | 15.6 | 5.4 | 3.1 |
| | (49.5-58.1) | (34.7-38.9) | (10.1-11.4) | (5.2-6.3) | (40.0-46.9) | (28.3-31.8) | (7.7-8.7) | (3.7-4.5) | (26.4-33.1) | (19.7-23.2) | (4.9-5.8) | (2.3-3.0) | (21.6-28.1) | (14.1-17.2) | (4.9-5.9) | (2.8-3.6) |
| Colombia | 35.8 | 25.0 | 15.5 | 11.5 | 29.6 | 21.6 | 13.9 | 10.5 | 18.1 | 13.5 | 8.5 | 6.3 | 17.9 | 11.7 | 7.0 | 5.2 |
| | (33.1-38.6) | (23.0-27.3) | (12.3-19.3) | (7.1-19.0) | (27.4-32.0) | (19.8-23.5) | (11.1-17.4) | (6.5-17.3) | (16.5-19.9) | (12.2-15.0) | (6.6-10.9) | (3.8-10.6) | (16.3-19.7) | (10.4-13.0) | (5.3-9.1) | (3.1-8.9) |
| Comoros | 120.5 | 79.9 | 48.9 | 39.0 | 93.5 | 65.2 | 42.8 | 35.0 | 48.7 | 34.8 | 26.3 | 22.1 | 75.3 | 46.4 | 23.0 | 16.9 |
| | (106.6-135.4) | (66.4-93.1) | (40.0-57.4) | (28.6-52.6) | (82.7-105.0) | (54.2-76.0) | (35.0-50.2) | (25.7-47.2) | (40.2-57.7) | (27.0-43.2) | (20.7-32.1) | (15.2-31.5) | (64.4-87.2) | (37.3-56.7) | (17.9-28.7) | (11.2-25.2) |
| Congo | 91.5 | 112.5 | 52.7 | 39.1 | 57.6 | 73.0 | 35.3 | 27.0 | 28.4 | 30.9 | 19.7 | 16.5 | 64.8 | 84.1 | 33.4 | 22.8 |

| Country | Under-five mortality rate (U5MR) (per 1,000 live births) | | | | Infant mortality rate (IMR) (per 1,000 live births) | | | | Neonatal mortality rate (NMR) (per 1,000 live births) | | | | Mortality rate age 1–59 months (per 1,000 children aged 28 days) | | | |
|---|---|---|---|---|---|---|---|---|---|---|---|---|---|---|---|---|
| | 1990 | 2000 | 2015 | 2024 | 1990 | 2000 | 2015 | 2024 | 1990 | 2000 | 2015 | 2024 | 1990 | 2000 | 2015 | 2024 |
| | (80.0-104.5) | (100.4-126.2) | (36.3-74.1) | (20.2-76.4) | (50.3-65.7) | (65.2-81.9) | (24.3-49.7) | (13.9-52.7) | (24.1-33.6) | (26.5-36.1) | (13.1-28.7) | (8.4-33.0) | (56.1-74.9) | (74.2-95.2) | (22.5-48.4) | (11.5-45.8) |
| Cook Islands | 23.8 | 18.7 | 13.4 | 13.3 | 19.8 | 15.6 | 11.2 | 11.3 | 12.8 | 10.0 | 7.2 | 7.0 | 11.1 | 8.7 | 6.2 | 6.1 |
| | (21.3-26.4) | (16.5-21.0) | (10.6-16.9) | (8.5-20.9) | (17.8-22.0) | (13.8-17.5) | (8.9-14.2) | (7.2-17.7) | (9.3-16.3) | (6.6-13.6) | (4.0-10.8) | (3.4-12.7) | (7.8-14.7) | (5.4-12.3) | (3.3-9.8) | (2.8-11.6) |
| Costa Rica | 16.9 | 13.1 | 9.0 | 10.4 | 14.4 | 11.2 | 7.8 | 9.1 | 9.0 | 7.6 | 6.2 | 7.3 | 8.0 | 5.5 | 2.8 | 3.1 |
| | (16.6-17.3) | (12.8-13.4) | (8.8-9.3) | (9.9-11.0) | (14.1-14.7) | (11.0-11.5) | (7.6-8.0) | (8.6-9.6) | (8.6-9.4) | (7.3-7.9) | (6.0-6.4) | (6.9-7.8) | (7.7-8.5) | (5.2-5.8) | (2.6-3.0) | (2.8-3.4) |
| Croatia | 12.9 | 8.3 | 4.9 | 5.6 | 11.2 | 7.2 | 4.2 | 4.1 | 8.2 | 5.5 | 3.1 | 2.8 | 4.8 | 2.8 | 1.8 | 2.9 |
| | (12.5-13.3) | (8.1-8.7) | (4.7-5.1) | (5.0-6.5) | (10.8-11.5) | (6.9-7.5) | (4.0-4.4) | (3.6-4.7) | (7.8-8.6) | (5.3-5.9) | (2.9-3.3) | (2.3-3.2) | (4.4-5.1) | (2.6-3.1) | (1.7-2.0) | (2.5-3.4) |
| Cuba | 13.5 | 8.6 | 6.1 | 8.6 | 10.8 | 6.8 | 4.8 | 6.8 | 6.8 | 4.1 | 2.6 | 4.3 | 6.7 | 4.5 | 3.6 | 4.3 |
| | (12.0-15.1) | (7.6-9.7) | (5.4-6.9) | (7.4-10.1) | (9.6-12.2) | (6.0-7.7) | (4.3-5.5) | (5.9-8.0) | (6.0-7.7) | (3.7-4.7) | (2.2-2.9) | (3.6-5.0) | (5.9-7.6) | (3.9-5.0) | (3.1-4.1) | (3.7-5.1) |
| Cyprus | 11.2 | 6.6 | 2.5 | 5.0 | 10.0 | 5.7 | 2.1 | 4.1 | 6.2 | 3.6 | 1.4 | 2.7 | 5.1 | 3.0 | 1.1 | 2.3 |
| | (10.6-11.9) | (6.1-7.1) | (2.2-2.9) | (3.9-6.3) | (9.4-10.6) | (5.3-6.2) | (1.8-2.4) | (3.3-5.2) | (5.1-7.1) | (3.2-4.1) | (1.2-1.7) | (2.1-3.6) | (4.1-6.2) | (2.5-3.4) | (0.9-1.3) | (1.7-3.0) |
| Czechia | 12.2 | 5.5 | 3.2 | 2.6 | 10.4 | 4.5 | 2.6 | 2.1 | 7.3 | 2.7 | 1.6 | 1.4 | 4.9 | 2.8 | 1.6 | 1.2 |
| | (11.9-12.4) | (5.3-5.7) | (3.0-3.3) | (2.4-2.8) | (10.1-10.6) | (4.3-4.6) | (2.5-2.7) | (1.9-2.3) | (7.0-7.6) | (2.5-2.9) | (1.5-1.7) | (1.3-1.6) | (4.7-5.2) | (2.6-2.9) | (1.5-1.7) | (1.1-1.3) |
| Côte d'Ivoire | 153.6 | 140.4 | 85.5 | 64.5 | 101.2 | 91.4 | 58.4 | 44.8 | 47.6 | 40.7 | 32.2 | 27.4 | 111.3 | 104.0 | 55.0 | 38.0 |
| | (142.5-166.0) | (130.4-152.0) | (77.0-95.0) | (50.6-81.8) | (93.8-109.3) | (84.9-98.9) | (52.6-64.9) | (35.1-56.8) | (42.9-52.8) | (36.2-45.4) | (28.1-36.8) | (20.7-35.9) | (102.1-121.4) | (95.7-113.6) | (48.8-61.8) | (29.0-49.5) |
| Democratic People's Republic of Korea | 40.7 | 100.2 | 21.4 | 16.7 | 32.0 | 59.4 | 17.2 | 13.3 | 21.3 | 23.8 | 11.4 | 8.9 | 19.7 | 78.1 | 9.9 | 7.7 |
| | (31.9-52.2) | (78.5-128.3) | (16.7-27.3) | (13.1-21.3) | (25.0-40.9) | (46.5-76.1) | (13.5-22.0) | (10.4-17.1) | (14.4-29.9) | (15.0-34.8) | (6.4-17.3) | (4.6-13.8) | (13.2-28.1) | (59.6-102.3) | (5.3-15.7) | (3.8-12.5) |
| Democratic Republic of the Congo | 180.0 | 184.6 | 91.9 | 89.7 | 98.5 | 105.0 | 55.1 | 56.4 | 38.7 | 34.1 | 24.0 | 23.6 | 146.7 | 155.7 | 69.6 | 67.6 |
| | (162.1-200.3) | (167.6-203.5) | (79.8-105.2) | (70.4-116.7) | (88.7-109.6) | (95.3-115.8) | (47.9-63.1) | (44.3-73.4) | (32.9-45.9) | (29.5-39.3) | (19.9-28.5) | (17.3-32.4) | (131.3-164.3) | (140.5-172.7) | (60.0-80.4) | (52.4-89.3) |
| Denmark | 8.9 | 5.6 | 4.1 | 4.0 | 7.3 | 4.7 | 3.5 | 3.5 | 4.4 | 3.5 | 2.9 | 2.7 | 4.6 | 2.2 | 1.2 | 1.3 |
| | (8.7-9.3) | (5.4-5.9) | (3.9-4.3) | (3.6-4.4) | (7.1-7.6) | (4.5-4.9) | (3.4-3.7) | (3.2-4.0) | (4.1-4.6) | (3.3-3.7) | (2.7-3.1) | (2.3-3.1) | (4.3-4.8) | (2.0-2.3) | (1.0-1.3) | (1.0-1.6) |
| Djibouti | 115.3 | 98.5 | 65.2 | 48.9 | 87.9 | 80.3 | 56.6 | 43.0 | 48.0 | 42.6 | 33.2 | 27.1 | 70.6 | 58.3 | 32.7 | 21.9 |
| | (98.0-134.1) | (84.2-116.6) | (44.1-95.0) | (25.4-92.6) | (74.7-102.2) | (68.7-95.2) | (38.3-82.4) | (22.3-81.5) | (39.1-58.2) | (34.5-52.2) | (21.6-50.7) | (13.5-53.7) | (58.4-84.0) | (48.5-71.0) | (20.7-50.1) | (10.4-44.5) |
| Dominica | 18.5 | 19.4 | 29.2 | 35.7 | 14.9 | 16.6 | 26.8 | 33.3 | 10.8 | 12.5 | 25.4 | 31.1 | 7.9 | 6.9 | 3.9 | 4.7 |
| | (17.4-19.7) | (18.2-20.7) | (26.8-31.9) | (30.8-41.4) | (14.1-15.9) | (15.6-17.8) | (24.6-29.2) | (28.8-38.8) | (9.7-11.8) | (11.4-13.8) | (23.2-27.9) | (26.5-36.4) | (6.9-8.9) | (6.0-7.9) | (3.2-4.7) | (3.2-6.7) |
| Dominican Republic | 59.3 | 39.6 | 34.9 | 30.6 | 48.0 | 33.5 | 31.0 | 27.7 | 24.3 | 22.9 | 24.4 | 21.2 | 35.8 | 17.0 | 10.7 | 9.4 |
| | (55.3-63.6) | (36.5-42.8) | (29.5-41.1) | (21.0-44.4) | (44.8-51.5) | (30.8-36.2) | (26.3-36.5) | (19.0-40.2) | (21.9-27.0) | (20.7-25.3) | (20.3-28.9) | (14.3-31.7) | (32.8-39.1) | (15.2-19.0) | (8.6-13.4) | (5.7-15.2) |
| Ecuador | 53.7 | 29.6 | 15.3 | 12.9 | 42.7 | 24.1 | 12.9 | 10.9 | 22.3 | 14.1 | 7.3 | 7.0 | 32.0 | 15.6 | 8.1 | 5.9 |
| | (48.2-59.9) | (26.2-33.4) | (14.7-15.9) | (11.3-14.6) | (38.3-47.6) | (21.4-27.3) | (12.4-13.4) | (9.6-12.4) | (18.9-26.0) | (12.1-16.6) | (6.6-7.9) | (5.8-8.3) | (28.0-36.8) | (13.4-18.2) | (7.5-8.9) | (4.8-7.1) |
| Egypt | 86.0 | 47.3 | 26.5 | 22.4 | 67.7 | 40.1 | 24.1 | 20.8 | 33.4 | 22.8 | 15.3 | 13.9 | 54.3 | 25.1 | 11.4 | 8.6 |

| | Under-five mortality rate (U5MR) (per 1,000 live births) | | | | Infant mortality rate (IMR) (per 1,000 live births) | | | | Neonatal mortality rate (NMR) (per 1,000 live births) | | | | Mortality rate age 1–59 months (per 1,000 children aged 28 days) | | | |
|---|---|---|---|---|---|---|---|---|---|---|---|---|---|---|---|---|
| Country | 1990 | 2000 | 2015 | 2024 | 1990 | 2000 | 2015 | 2024 | 1990 | 2000 | 2015 | 2024 | 1990 | 2000 | 2015 | 2024 |
| | (81.5-90.6) | (44.2-50.5) | (23.4-30.1) | (16.3-30.6) | (64.2-71.3) | (37.5-42.8) | (21.3-27.3) | (15.2-28.5) | (30.8-36.1) | (20.9-24.8) | (13.4-17.6) | (9.9-19.2) | (50.8-58.0) | (23.1-27.3) | (9.8-13.2) | (5.9-12.3) |
| El Salvador | 59.5 | 32.2 | 14.6 | 10.0 | 47.2 | 26.4 | 12.6 | 8.8 | 22.4 | 14.5 | 6.4 | 4.3 | 37.9 | 17.9 | 8.2 | 5.7 |
| | (54.3-65.1) | (29.0-35.7) | (11.0-19.3) | (6.1-17.6) | (43.1-51.7) | (23.8-29.3) | (9.5-16.6) | (5.3-15.5) | (19.8-25.5) | (12.8-16.5) | (4.8-8.6) | (2.6-7.8) | (33.9-42.1) | (15.8-20.2) | (6.1-11.0) | (3.4-10.1) |
| Equatorial Guinea | 176.7 | 154.5 | 92.1 | 68.0 | 118.2 | 106.2 | 62.9 | 47.6 | 49.9 | 46.1 | 31.5 | 26.3 | 133.0 | 113.7 | 61.9 | 41.9 |
| | (155.7-198.2) | (137.7-177.9) | (62.1-132.0) | (35.0-128.1) | (104.2-132.6) | (94.7-122.3) | (42.4-90.2) | (24.5-89.7) | (38.1-65.4) | (36.8-58.0) | (18.0-50.9) | (11.4-55.7) | (112.9-153.6) | (98.1-133.5) | (39.6-92.8) | (19.7-83.9) |
| Eritrea | 153.4 | 85.3 | 46.4 | 34.3 | 80.8 | 50.8 | 32.4 | 24.7 | 34.7 | 26.1 | 19.4 | 15.6 | 123.1 | 60.8 | 27.3 | 18.8 |
| | (138.9-169.8) | (76.9-94.8) | (32.5-66.4) | (18.5-65.2) | (73.2-89.4) | (45.8-56.5) | (22.7-46.3) | (13.3-47.0) | (29.4-40.2) | (22.4-30.0) | (13.2-28.7) | (8.1-30.9) | (110.5-137.2) | (54.2-68.3) | (18.5-40.2) | (9.7-36.8) |
| Estonia | 17.7 | 11.0 | 3.2 | 2.0 | 14.0 | 8.8 | 2.4 | 1.5 | 9.9 | 5.3 | 1.4 | 0.9 | 7.9 | 5.7 | 1.7 | 1.1 |
| | (17.1-18.3) | (10.5-11.6) | (2.9-3.4) | (1.7-2.5) | (13.5-14.5) | (8.3-9.2) | (2.3-2.7) | (1.3-1.9) | (9.2-10.5) | (4.8-5.8) | (1.3-1.6) | (0.7-1.2) | (7.3-8.6) | (5.2-6.3) | (1.6-2.0) | (0.8-1.3) |
| Eswatini | 66.3 | 112.5 | 57.5 | 45.1 | 56.7 | 71.7 | 54.1 | 42.2 | 21.0 | 24.9 | 26.6 | 25.0 | 46.2 | 89.8 | 31.7 | 20.3 |
| | (58.6-74.8) | (100.3-126.0) | (46.4-72.3) | (28.5-72.7) | (50.1-63.9) | (63.9-80.4) | (43.7-68.0) | (26.7-68.0) | (17.6-25.0) | (21.7-28.6) | (21.1-33.8) | (15.4-41.3) | (40.2-52.9) | (79.6-101.3) | (25.2-40.6) | (12.1-34.7) |
| Ethiopia | 202.0 | 140.1 | 64.4 | 44.5 | 127.2 | 92.3 | 47.9 | 34.5 | 59.5 | 48.1 | 32.3 | 25.2 | 151.4 | 96.7 | 33.2 | 19.7 |
| | (187.4-217.5) | (130.5-150.6) | (57.1-72.6) | (31.3-63.4) | (118.1-137.0) | (86.0-99.2) | (42.5-54.0) | (24.3-49.1) | (53.5-65.9) | (43.3-53.0) | (28.2-37.0) | (17.5-36.3) | (139.2-164.5) | (89.3-105.0) | (28.9-38.0) | (13.5-28.9) |
| Fiji | 28.8 | 22.6 | 20.9 | 29.1 | 21.5 | 16.6 | 16.1 | 23.5 | 13.2 | 10.0 | 8.5 | 15.0 | 15.8 | 12.8 | 12.5 | 14.2 |
| | (24.9-33.3) | (21.6-23.8) | (20.2-21.6) | (23.6-35.8) | (18.5-24.8) | (15.9-17.5) | (15.6-16.7) | (19.1-28.9) | (10.3-16.5) | (8.9-11.1) | (7.5-9.6) | (11.3-19.5) | (12.7-19.4) | (11.6-14.0) | (11.4-13.6) | (10.6-18.8) |
| Finland | 6.7 | 4.3 | 2.5 | 2.4 | 5.6 | 3.5 | 2.0 | 1.9 | 3.9 | 2.5 | 1.4 | 1.4 | 2.9 | 1.8 | 1.1 | 1.0 |
| | (6.5-7.0) | (4.1-4.5) | (2.4-2.6) | (2.1-2.6) | (5.4-5.8) | (3.4-3.7) | (1.9-2.1) | (1.7-2.1) | (3.7-4.1) | (2.3-2.6) | (1.3-1.5) | (1.2-1.5) | (2.7-3.1) | (1.7-1.9) | (1.0-1.2) | (0.9-1.1) |
| France | 9.0 | 5.4 | 4.2 | 4.3 | 7.5 | 4.1 | 3.2 | 3.4 | 3.6 | 2.7 | 2.4 | 2.8 | 5.4 | 2.6 | 1.7 | 1.5 |
| | (8.8-9.2) | (5.3-5.5) | (4.1-4.3) | (4.1-4.6) | (7.3-7.6) | (4.0-4.2) | (3.1-3.3) | (3.3-3.6) | (3.5-3.8) | (2.6-2.9) | (2.4-2.5) | (2.6-3.0) | (5.2-5.6) | (2.5-2.7) | (1.7-1.8) | (1.4-1.7) |
| Gabon | 84.3 | 73.3 | 44.4 | 32.6 | 58.8 | 50.7 | 33.7 | 25.9 | 27.9 | 26.5 | 21.3 | 16.3 | 57.9 | 48.0 | 23.6 | 16.4 |
| | (72.8-97.0) | (64.8-83.2) | (37.2-53.5) | (22.5-48.9) | (50.9-67.7) | (44.8-57.5) | (28.2-40.7) | (17.9-38.9) | (22.9-34.0) | (22.6-31.2) | (17.4-26.0) | (10.6-25.6) | (49.2-68.0) | (41.7-55.2) | (19.3-29.0) | (10.5-25.7) |
| Gambia | 166.2 | 112.8 | 58.9 | 42.4 | 99.5 | 73.3 | 43.5 | 32.7 | 49.5 | 40.2 | 29.0 | 23.0 | 122.7 | 75.7 | 30.8 | 19.6 |
| | (150.7-183.6) | (102.9-123.5) | (50.8-68.0) | (30.0-59.1) | (90.3-110.0) | (66.9-80.3) | (37.5-50.2) | (23.1-45.6) | (40.9-58.9) | (34.6-45.8) | (24.4-34.2) | (15.6-33.2) | (109.0-138.4) | (67.9-84.5) | (25.9-36.5) | (13.1-28.9) |
| Georgia | 47.3 | 35.8 | 10.6 | 8.6 | 38.5 | 29.2 | 8.9 | 7.5 | 22.1 | 22.3 | 6.7 | 5.0 | 25.7 | 13.8 | 3.8 | 3.7 |
| | (42.2-53.1) | (31.5-41.1) | (9.9-11.2) | (8.1-9.2) | (34.3-43.2) | (25.7-33.5) | (8.4-9.5) | (7.0-7.9) | (19.0-25.6) | (19.2-26.0) | (6.2-7.3) | (4.5-5.4) | (22.4-29.6) | (11.6-16.5) | (3.5-4.2) | (3.3-4.1) |
| Germany | 8.5 | 5.4 | 3.9 | 3.7 | 7.0 | 4.4 | 3.3 | 3.1 | 3.4 | 2.8 | 2.3 | 2.3 | 5.2 | 2.6 | 1.6 | 1.4 |
| | (8.4-8.7) | (5.3-5.5) | (3.9-4.0) | (3.6-3.9) | (6.9-7.1) | (4.3-4.5) | (3.2-3.3) | (3.0-3.2) | (3.2-3.5) | (2.7-2.9) | (2.2-2.4) | (2.2-2.4) | (5.0-5.3) | (2.5-2.7) | (1.6-1.7) | (1.3-1.5) |
| Ghana | 127.6 | 99.9 | 52.5 | 35.9 | 72.7 | 61.7 | 37.9 | 27.5 | 42.0 | 35.2 | 24.3 | 18.1 | 89.3 | 67.1 | 28.9 | 18.0 |
| | (120.4-135.6) | (94.1-105.8) | (48.0-57.3) | (27.6-46.7) | (68.6-77.3) | (58.1-65.4) | (34.7-41.4) | (21.2-35.8) | (38.4-46.0) | (32.0-38.4) | (21.8-27.1) | (13.4-24.0) | (83.5-95.7) | (62.4-71.9) | (26.1-32.2) | (13.5-24.1) |
| Greece | 10.5 | 6.4 | 4.3 | 3.8 | 9.2 | 5.5 | 3.6 | 3.2 | 6.5 | 3.9 | 2.6 | 2.2 | 4.0 | 2.5 | 1.7 | 1.6 |
| | (10.2-10.7) | (6.2-6.6) | (4.1-4.5) | (3.5-4.2) | (9.0-9.5) | (5.4-5.7) | (3.5-3.8) | (2.9-3.6) | (6.3-6.8) | (3.8-4.1) | (2.4-2.7) | (1.9-2.5) | (3.8-4.2) | (2.4-2.6) | (1.6-1.8) | (1.4-1.9) |

| Country | Under-five mortality rate (U5MR) (per 1,000 live births) | | | | Infant mortality rate (IMR) (per 1,000 live births) | | | | Neonatal mortality rate (NMR) (per 1,000 live births) | | | | Mortality rate age 1–59 months (per 1,000 children aged 28 days) | | | |
|---|---|---|---|---|---|---|---|---|---|---|---|---|---|---|---|---|
| | 1990 | 2000 | 2015 | 2024 | 1990 | 2000 | 2015 | 2024 | 1990 | 2000 | 2015 | 2024 | 1990 | 2000 | 2015 | 2024 |
| Grenada | 22.4 | 15.5 | 17.5 | 18.0 | 18.5 | 13.3 | 15.7 | 16.4 | 12.3 | 7.8 | 11.5 | 12.3 | 10.2 | 7.8 | 6.1 | 5.7 |
| | (20.8-24.1) | (14.2-17.0) | (16.0-19.2) | (13.7-23.4) | (17.2-19.8) | (12.2-14.6) | (14.3-17.3) | (12.6-21.5) | (11.0-13.8) | (6.8-8.9) | (10.2-12.9) | (9.2-16.4) | (8.9-11.6) | (6.8-8.9) | (5.2-7.1) | (3.9-8.1) |
| Guatemala | 80.0 | 51.8 | 28.1 | 20.5 | 60.6 | 40.7 | 23.1 | 17.2 | 28.6 | 21.1 | 13.0 | 9.5 | 52.9 | 31.4 | 15.2 | 11.1 |
| | (74.7-85.5) | (47.7-56.2) | (23.8-33.2) | (13.5-30.8) | (56.6-64.8) | (37.5-44.1) | (19.6-27.3) | (11.3-25.8) | (25.8-31.5) | (18.8-23.5) | (10.6-15.8) | (6.0-14.7) | (48.8-57.2) | (28.5-34.5) | (12.5-18.5) | (7.1-17.0) |
| Guinea | 233.4 | 165.7 | 113.7 | 92.1 | 134.5 | 97.3 | 71.2 | 59.8 | 61.6 | 46.1 | 33.7 | 29.8 | 182.8 | 125.3 | 82.8 | 63.8 |
| | (216.2-251.3) | (153.6-178.6) | (101.7-127.3) | (68.8-124.4) | (124.5-144.8) | (90.2-104.9) | (63.7-79.7) | (44.7-80.8) | (54.6-69.5) | (41.0-51.7) | (28.5-39.6) | (20.0-43.8) | (168.2-198.7) | (115.2-136.4) | (73.2-93.9) | (46.1-88.8) |
| Guinea-Bissau | 223.7 | 174.8 | 91.6 | 67.3 | 123.9 | 100.7 | 55.2 | 42.1 | 63.9 | 55.2 | 39.1 | 32.0 | 170.5 | 126.3 | 54.3 | 35.9 |
| | (199.4-249.3) | (156.8-194.6) | (65.7-124.1) | (36.8-117.5) | (110.4-138.0) | (90.3-112.1) | (39.6-74.8) | (23.0-73.4) | (54.3-74.9) | (47.0-63.9) | (27.2-55.3) | (16.7-58.8) | (150.1-192.7) | (111.9-143.0) | (37.3-75.8) | (18.4-66.2) |
| Guyana | 61.4 | 46.7 | 33.3 | 25.2 | 53.3 | 41.5 | 30.4 | 23.2 | 31.2 | 27.2 | 20.3 | 15.3 | 31.1 | 20.1 | 13.2 | 9.9 |
| | (55.4-68.2) | (41.9-52.2) | (23.0-48.3) | (13.9-46.5) | (48.1-59.2) | (37.1-46.3) | (21.0-44.1) | (12.8-42.8) | (27.1-35.8) | (23.7-31.2) | (13.5-30.0) | (8.0-28.8) | (27.1-35.6) | (17.0-23.4) | (8.5-20.1) | (4.9-19.6) |
| Haiti | 144.5 | 102.9 | 69.1 | 52.8 | 98.6 | 71.1 | 49.7 | 39.0 | 38.7 | 29.5 | 26.7 | 22.6 | 110.0 | 75.6 | 43.5 | 30.4 |
| | (134.1-156.0) | (94.3-111.9) | (57.8-84.4) | (34.2-82.6) | (91.5-106.4) | (65.2-77.3) | (41.6-60.7) | (25.2-61.1) | (33.9-43.9) | (25.8-33.5) | (21.1-34.2) | (13.4-38.1) | (101.1-119.9) | (68.6-83.0) | (35.4-54.1) | (18.8-49.7) |
| Honduras | 58.3 | 37.3 | 20.3 | 15.0 | 44.7 | 29.6 | 17.0 | 12.8 | 22.1 | 17.9 | 11.5 | 8.4 | 37.0 | 19.7 | 9.0 | 6.5 |
| | (53.7-63.1) | (34.2-40.7) | (17.3-24.2) | (10.4-21.5) | (41.1-48.4) | (27.1-32.3) | (14.4-20.2) | (8.9-18.3) | (19.2-25.1) | (15.8-20.2) | (9.5-13.9) | (5.6-12.5) | (33.2-41.0) | (17.5-22.2) | (7.2-11.0) | (4.2-9.9) |
| Hungary | 17.2 | 10.1 | 5.1 | 3.7 | 15.2 | 8.7 | 4.2 | 3.1 | 11.1 | 5.7 | 2.7 | 2.1 | 6.1 | 4.4 | 2.4 | 1.6 |
| | (16.8-17.6) | (9.8-10.4) | (4.9-5.2) | (3.3-4.2) | (14.8-15.5) | (8.4-8.9) | (4.1-4.4) | (2.7-3.5) | (10.8-11.5) | (5.5-6.0) | (2.5-2.9) | (1.8-2.4) | (5.8-6.4) | (4.2-4.6) | (2.2-2.5) | (1.4-1.9) |
| Iceland | 6.4 | 3.9 | 2.6 | 2.8 | 5.2 | 3.1 | 2.0 | 2.1 | 3.5 | 2.0 | 1.3 | 1.4 | 3.0 | 1.9 | 1.3 | 1.4 |
| | (5.9-7.1) | (3.5-4.4) | (2.3-3.0) | (2.2-3.7) | (4.7-5.7) | (2.8-3.5) | (1.7-2.3) | (1.6-2.8) | (3.1-3.9) | (1.8-2.4) | (1.1-1.5) | (1.0-1.9) | (2.6-3.4) | (1.6-2.2) | (1.1-1.5) | (1.0-1.9) |
| India | 127.0 | 91.8 | 43.7 | 26.6 | 84.4 | 66.3 | 36.3 | 23.3 | 57.0 | 43.8 | 26.0 | 16.7 | 74.2 | 50.1 | 18.2 | 10.1 |
| | (122.5-131.6) | (88.3-95.4) | (41.7-45.8) | (24.1-29.4) | (81.4-87.4) | (63.8-68.9) | (34.6-38.1) | (21.1-25.8) | (53.9-60.4) | (41.3-46.5) | (24.5-27.6) | (14.9-18.6) | (70.5-78.0) | (47.4-53.1) | (16.9-19.5) | (8.8-11.4) |
| Indonesia | 83.2 | 51.4 | 26.1 | 17.7 | 64.4 | 41.4 | 21.9 | 15.2 | 30.1 | 22.5 | 13.4 | 9.2 | 54.7 | 29.6 | 12.9 | 8.6 |
| | (79.1-87.7) | (48.6-54.2) | (23.9-28.8) | (14.6-21.8) | (61.3-67.9) | (39.2-43.7) | (20.1-24.2) | (12.5-18.7) | (27.9-32.4) | (20.7-24.1) | (12.1-15.0) | (7.4-11.5) | (51.6-58.2) | (27.7-31.7) | (11.6-14.4) | (6.9-10.8) |
| Iran (Islamic Republic of) | 58.0 | 36.4 | 15.5 | 11.3 | 49.0 | 31.7 | 14.0 | 10.3 | 24.7 | 19.5 | 10.0 | 7.2 | 34.0 | 17.2 | 5.3 | 3.8 |
| | (53.0-63.4) | (32.9-40.6) | (11.0-21.7) | (6.1-20.9) | (44.8-53.6) | (28.7-35.4) | (9.9-19.5) | (5.5-19.1) | (16.3-32.6) | (15.9-23.2) | (6.1-15.4) | (3.0-14.6) | (26.2-43.0) | (13.9-21.2) | (2.3-9.4) | (1.1-9.6) |
| Iraq | 81.4 | 44.3 | 29.3 | 21.8 | 55.6 | 38.4 | 26.6 | 20.1 | 26.8 | 23.9 | 16.7 | 12.5 | 56.1 | 21.0 | 12.8 | 9.4 |
| | (74.0-89.4) | (40.3-48.9) | (24.5-35.0) | (14.8-31.9) | (50.6-61.1) | (34.9-42.3) | (22.3-31.8) | (13.6-29.4) | (23.8-30.0) | (21.4-26.8) | (13.8-20.2) | (8.3-18.5) | (50.6-62.1) | (18.5-23.5) | (10.4-15.7) | (6.2-14.3) |
| Ireland | 9.2 | 7.2 | 3.7 | 3.9 | 7.6 | 6.0 | 3.2 | 3.4 | 4.7 | 4.0 | 2.4 | 2.6 | 4.5 | 3.2 | 1.3 | 1.3 |
| | (8.9-9.5) | (6.9-7.5) | (3.5-3.8) | (3.3-4.6) | (7.3-7.9) | (5.8-6.2) | (3.0-3.3) | (2.9-4.1) | (4.5-5.0) | (3.8-4.2) | (2.3-2.5) | (2.2-3.1) | (4.2-4.7) | (3.0-3.4) | (1.2-1.4) | (1.0-1.6) |
| Israel | 11.5 | 6.9 | 3.9 | 3.3 | 9.6 | 5.6 | 3.1 | 2.7 | 6.2 | 3.6 | 2.1 | 1.7 | 5.4 | 3.3 | 1.8 | 1.6 |
| | (11.3-11.8) | (6.7-7.0) | (3.8-4.0) | (3.1-3.6) | (9.4-9.9) | (5.5-5.7) | (3.1-3.2) | (2.5-2.9) | (6.0-6.5) | (3.4-3.7) | (2.0-2.2) | (1.6-1.9) | (5.1-5.6) | (3.2-3.5) | (1.7-1.9) | (1.5-1.8) |
| Italy | 9.7 | 5.6 | 3.5 | 2.7 | 8.4 | 4.7 | 3.0 | 2.3 | 6.4 | 3.5 | 2.1 | 1.8 | 3.3 | 2.1 | 1.4 | 0.9 |

| Country | Under-five mortality rate (U5MR) (per 1,000 live births) | | | | Infant mortality rate (IMR) (per 1,000 live births) | | | | Neonatal mortality rate (NMR) (per 1,000 live births) | | | | Mortality rate age 1–59 months (per 1,000 children aged 28 days) | | | |
|---|---|---|---|---|---|---|---|---|---|---|---|---|---|---|---|---|
| | 1990 | 2000 | 2015 | 2024 | 1990 | 2000 | 2015 | 2024 | 1990 | 2000 | 2015 | 2024 | 1990 | 2000 | 2015 | 2024 |
| | (9.5-9.8) | (5.5-5.7) | (3.4-3.6) | (2.5-2.9) | (8.2-8.5) | (4.6-4.8) | (2.9-3.0) | (2.2-2.4) | (6.3-6.6) | (3.4-3.6) | (2.1-2.2) | (1.7-2.0) | (3.1-3.4) | (2.0-2.2) | (1.3-1.4) | (0.8-1.0) |
| Jamaica | 28.0 | 20.5 | 18.7 | 17.8 | 24.7 | 18.5 | 17.2 | 16.4 | 18.4 | 16.0 | 14.3 | 13.7 | 9.7 | 4.6 | 4.5 | 4.2 |
| | (23.3-33.6) | (17.7-23.9) | (18.0-19.5) | (16.5-19.1) | (20.5-29.6) | (15.9-21.6) | (16.5-17.9) | (15.2-17.6) | (15.1-22.4) | (13.7-18.8) | (13.6-15.1) | (12.5-14.8) | (7.7-12.2) | (3.6-5.7) | (4.0-5.0) | (3.6-4.9) |
| Japan | 6.3 | 4.5 | 2.8 | 2.4 | 4.6 | 3.3 | 2.0 | 1.8 | 2.5 | 1.8 | 0.9 | 0.9 | 3.8 | 2.7 | 1.8 | 1.6 |
| | (6.2-6.4) | (4.4-4.6) | (2.7-2.8) | (2.4-2.5) | (4.5-4.7) | (3.2-3.3) | (2.0-2.1) | (1.8-1.9) | (2.5-2.6) | (1.7-1.8) | (0.9-1.0) | (0.8-0.9) | (3.7-3.9) | (2.7-2.8) | (1.8-1.9) | (1.5-1.6) |
| Jordan | 35.6 | 25.8 | 16.5 | 12.9 | 30.9 | 22.8 | 15.0 | 12.0 | 19.7 | 15.4 | 9.7 | 7.4 | 16.2 | 10.6 | 6.9 | 5.6 |
| | (33.1-38.2) | (23.8-28.0) | (14.1-19.6) | (9.5-18.7) | (28.8-33.2) | (21.1-24.7) | (12.8-17.8) | (8.7-17.2) | (17.9-21.6) | (13.9-16.9) | (8.2-11.7) | (5.3-11.0) | (14.6-18.0) | (9.5-11.9) | (5.7-8.3) | (3.9-8.3) |
| Kazakhstan | 51.1 | 42.1 | 11.9 | 9.4 | 44.3 | 35.8 | 9.5 | 7.4 | 22.3 | 23.6 | 5.5 | 3.9 | 29.3 | 18.9 | 6.4 | 5.5 |
| | (46.3-56.2) | (38.6-46.2) | (11.6-12.1) | (8.9-10.0) | (40.2-48.8) | (32.8-39.3) | (9.3-9.7) | (7.0-7.9) | (19.3-25.6) | (20.6-27.0) | (5.1-5.8) | (3.6-4.4) | (25.8-33.3) | (16.2-22.0) | (6.1-6.8) | (5.1-6.0) |
| Kenya | 101.4 | 95.2 | 46.8 | 38.8 | 67.5 | 63.7 | 38.9 | 34.2 | 26.7 | 26.3 | 23.2 | 20.7 | 76.8 | 70.7 | 24.1 | 18.4 |
| | (94.5-108.5) | (88.5-102.5) | (42.6-51.5) | (31.7-47.6) | (62.9-72.2) | (59.2-68.6) | (35.5-42.9) | (27.8-41.9) | (24.0-29.5) | (23.7-29.1) | (20.7-26.1) | (16.4-26.1) | (71.2-82.6) | (65.4-76.6) | (21.4-27.1) | (14.3-23.6) |
| Kiribati | 91.2 | 65.8 | 58.6 | 53.1 | 69.2 | 49.2 | 42.6 | 38.8 | 34.4 | 27.1 | 23.3 | 21.7 | 58.4 | 39.8 | 35.7 | 31.3 |
| | (80.6-103.1) | (58.3-74.4) | (45.4-74.8) | (33.0-85.3) | (61.2-78.3) | (43.6-55.7) | (32.9-54.3) | (24.1-62.3) | (27.0-45.6) | (21.9-33.0) | (13.1-34.8) | (9.6-40.5) | (47.2-69.3) | (33.5-46.9) | (25.2-50.6) | (17.2-55.3) |
| Kosovo (UNSCR 1244) | 104.4 | 48.4 | 14.3 | 8.7 | 91.5 | 43.6 | 13.1 | 8.0 | 43.4 | 27.3 | 10.3 | 6.6 | 63.9 | 21.5 | 4.1 | 2.2 |
| | (80.1-139.8) | (40.0-58.2) | (12.6-16.2) | (6.8-11.2) | (70.2-122.5) | (36.1-52.4) | (11.5-14.8) | (6.2-10.2) | (32.2-59.7) | (22.1-33.6) | (8.9-11.8) | (5.0-8.5) | (47.5-87.1) | (17.2-26.8) | (3.3-5.0) | (1.3-3.2) |
| Kuwait | 17.2 | 12.7 | 9.1 | 8.1 | 14.5 | 10.6 | 7.8 | 7.0 | 10.1 | 6.6 | 5.0 | 4.4 | 7.2 | 6.2 | 4.2 | 3.7 |
| | (16.7-17.8) | (12.4-13.1) | (8.9-9.4) | (7.6-8.6) | (14.1-14.9) | (10.3-11.0) | (7.6-8.1) | (6.6-7.5) | (9.5-10.9) | (6.2-6.9) | (4.7-5.3) | (4.1-4.8) | (6.5-7.8) | (5.9-6.6) | (3.9-4.4) | (3.4-4.0) |
| Kyrgyzstan | 64.5 | 50.8 | 22.3 | 16.8 | 56.2 | 44.0 | 19.4 | 14.8 | 24.1 | 20.6 | 13.6 | 10.8 | 41.4 | 30.8 | 8.8 | 6.1 |
| | (56.3-73.4) | (45.9-55.6) | (21.7-23.0) | (15.4-18.4) | (49.0-64.0) | (39.7-48.2) | (18.9-20.1) | (13.6-16.2) | (19.8-28.7) | (17.5-23.8) | (12.5-14.7) | (9.0-12.8) | (35.4-48.1) | (27.1-34.7) | (7.8-9.9) | (4.3-7.8) |
| Lao People's Democratic Republic | 148.9 | 100.1 | 44.4 | 29.4 | 111.0 | 82.2 | 39.9 | 27.2 | 47.3 | 35.8 | 18.9 | 13.1 | 106.7 | 66.7 | 26.0 | 16.4 |
| | (136.2-162.8) | (91.7-109.7) | (38.0-51.5) | (21.6-39.4) | (101.6-121.4) | (75.3-90.1) | (34.2-46.3) | (20.0-36.4) | (41.4-54.1) | (31.6-40.7) | (15.7-22.6) | (9.2-18.2) | (96.1-118.3) | (60.2-74.2) | (21.9-30.7) | (11.7-22.6) |
| Latvia | 16.9 | 14.3 | 5.1 | 2.5 | 13.1 | 11.6 | 4.3 | 2.2 | 8.3 | 7.2 | 2.8 | 1.2 | 8.7 | 7.1 | 2.4 | 1.3 |
| | (16.4-17.5) | (13.6-15.0) | (4.8-5.5) | (2.1-3.0) | (12.7-13.5) | (11.0-12.1) | (4.0-4.6) | (1.8-2.6) | (7.8-8.8) | (6.7-7.7) | (2.5-3.0) | (0.9-1.5) | (8.2-9.2) | (6.6-7.7) | (2.1-2.6) | (1.0-1.6) |
| Lebanon | 31.9 | 20.0 | 12.9 | 17.6 | 28.3 | 17.6 | 11.3 | 15.3 | 20.0 | 12.0 | 7.6 | 10.5 | 12.1 | 8.0 | 5.3 | 7.2 |
| | (28.3-35.8) | (16.9-23.6) | (11.4-14.7) | (15.8-19.6) | (25.1-31.8) | (14.9-20.8) | (10.0-12.8) | (13.7-17.0) | (17.5-22.9) | (9.9-14.3) | (6.7-8.8) | (9.2-11.8) | (10.2-14.1) | (6.6-10.0) | (4.6-6.2) | (6.3-8.2) |
| Lesotho | 84.5 | 108.3 | 75.1 | 60.0 | 75.9 | 74.8 | 69.0 | 56.3 | 38.6 | 37.1 | 34.1 | 28.5 | 47.7 | 73.9 | 42.4 | 32.1 |
| | (76.1-94.1) | (99.2-118.5) | (67.4-84.4) | (42.3-82.9) | (68.3-84.5) | (68.5-81.9) | (61.9-77.5) | (39.8-77.9) | (33.7-44.1) | (33.6-41.0) | (29.9-39.1) | (19.4-41.1) | (42.0-54.2) | (67.2-81.4) | (37.2-48.5) | (22.0-46.2) |
| Liberia | 270.0 | 194.9 | 93.2 | 86.4 | 170.9 | 125.5 | 65.1 | 63.2 | 61.1 | 49.6 | 34.0 | 32.9 | 222.3 | 152.9 | 61.3 | 55.0 |
| | (246.2-295.0) | (180.4-210.9) | (83.5-104.6) | (66.1-108.4) | (155.8-186.7) | (116.2-135.8) | (58.3-73.1) | (48.4-79.3) | (52.3-70.9) | (43.6-56.3) | (29.3-39.7) | (24.2-43.8) | (200.9-245.3) | (140.2-166.7) | (54.1-69.6) | (41.1-70.3) |
| Libya | 42.1 | 28.1 | 13.3 | 9.8 | 31.8 | 21.5 | 10.6 | 7.9 | 21.3 | 15.0 | 7.1 | 5.2 | 21.2 | 13.3 | 6.2 | 4.5 |
| | (35.7-49.8) | (26.6-29.8) | (9.7-18.1) | (5.3-17.7) | (26.9-37.5) | (20.3-22.8) | (7.7-14.4) | (4.3-14.3) | (17.6-25.9) | (12.7-17.8) | (4.8-10.3) | (2.6-9.9) | (17.5-25.7) | (10.6-15.7) | (4.1-9.2) | (2.2-8.8) |

| Country | Under-five mortality rate (U5MR) (per 1,000 live births) | | | | Infant mortality rate (IMR) (per 1,000 live births) | | | | Neonatal mortality rate (NMR) (per 1,000 live births) | | | | Mortality rate age 1–59 months (per 1,000 children aged 28 days) | | | |
|---|---|---|---|---|---|---|---|---|---|---|---|---|---|---|---|---|
| | 1990 | 2000 | 2015 | 2024 | 1990 | 2000 | 2015 | 2024 | 1990 | 2000 | 2015 | 2024 | 1990 | 2000 | 2015 | 2024 |
| Lithuania | 15.0 | 10.7 | 5.0 | 3.4 | 11.8 | 8.6 | 4.1 | 2.8 | 7.7 | 4.5 | 2.4 | 1.9 | 7.3 | 6.2 | 2.6 | 1.5 |
| | (14.4-15.5) | (10.2-11.2) | (4.7-5.4) | (2.9-4.0) | (11.3-12.2) | (8.2-9.0) | (3.8-4.4) | (2.4-3.3) | (7.2-8.2) | (4.1-4.9) | (2.1-2.7) | (1.5-2.3) | (6.8-7.8) | (5.8-6.6) | (2.4-2.9) | (1.2-1.8) |
| Luxembourg | 8.8 | 4.7 | 2.5 | 2.2 | 7.3 | 3.9 | 2.1 | 1.9 | 4.2 | 2.4 | 1.5 | 1.5 | 4.6 | 2.3 | 1.0 | 0.7 |
| | (8.1-9.5) | (4.3-5.2) | (2.3-2.9) | (1.7-2.8) | (6.7-7.9) | (3.5-4.3) | (1.9-2.4) | (1.5-2.4) | (3.6-4.8) | (2.0-2.7) | (1.3-1.8) | (1.1-2.0) | (4.0-5.2) | (2.0-2.7) | (0.9-1.2) | (0.5-1.1) |
| Madagascar | 154.8 | 104.4 | 65.3 | 62.4 | 91.2 | 65.4 | 43.6 | 43.0 | 38.4 | 31.0 | 23.7 | 23.1 | 121.0 | 75.8 | 42.5 | 40.2 |
| | (143.6-166.5) | (97.0-112.5) | (58.3-72.7) | (47.9-82.0) | (84.6-98.1) | (60.8-70.5) | (39.0-48.6) | (33.0-56.4) | (34.6-42.6) | (28.1-34.2) | (20.8-27.0) | (17.3-31.1) | (111.6-131.0) | (70.0-82.1) | (37.6-47.8) | (30.5-53.3) |
| Malawi | 243.5 | 171.0 | 57.4 | 48.8 | 134.0 | 98.9 | 39.7 | 38.0 | 49.8 | 37.9 | 24.0 | 23.6 | 203.9 | 138.2 | 34.1 | 25.8 |
| | (229.0-259.2) | (160.4-182.2) | (51.3-63.9) | (36.6-65.5) | (126.0-142.7) | (92.8-105.4) | (35.5-44.3) | (28.5-51.0) | (44.6-55.4) | (33.8-41.9) | (21.1-27.2) | (17.4-32.1) | (190.5-218.2) | (129.0-148.5) | (30.2-38.5) | (18.9-35.3) |
| Malaysia | 16.6 | 9.9 | 8.2 | 8.3 | 12.4 | 7.5 | 6.7 | 7.0 | 7.5 | 4.9 | 4.2 | 4.3 | 9.2 | 5.0 | 4.0 | 4.0 |
| | (16.2-17.1) | (9.7-10.1) | (8.0-8.3) | (7.5-9.2) | (12.1-12.7) | (7.4-7.7) | (6.5-6.8) | (6.3-7.7) | (6.4-8.8) | (4.3-5.6) | (3.8-4.5) | (3.7-4.8) | (7.9-10.3) | (4.3-5.6) | (3.7-4.3) | (3.5-4.6) |
| Maldives | 87.0 | 39.5 | 9.9 | 5.4 | 72.8 | 33.8 | 8.5 | 4.7 | 42.9 | 22.2 | 5.9 | 3.7 | 45.9 | 17.6 | 4.0 | 1.7 |
| | (79.1-95.6) | (35.7-43.7) | (9.2-10.5) | (4.3-6.9) | (66.3-80.1) | (30.6-37.4) | (8.0-9.1) | (3.7-6.0) | (36.0-50.6) | (18.9-25.7) | (5.2-6.5) | (2.8-4.8) | (38.7-53.5) | (14.8-21.1) | (3.5-4.6) | (1.1-2.6) |
| Mali | 233.3 | 189.9 | 109.7 | 73.0 | 124.7 | 103.1 | 66.1 | 47.5 | 67.4 | 51.6 | 35.4 | 28.0 | 177.7 | 145.7 | 77.0 | 46.2 |
| | (218.2-249.2) | (178.1-202.4) | (100.7-119.4) | (61.3-85.8) | (116.7-133.2) | (96.7-109.8) | (60.7-72.0) | (39.9-55.9) | (60.9-74.7) | (46.6-57.0) | (31.4-39.7) | (22.3-34.7) | (164.7-191.6) | (135.8-156.6) | (70.1-84.8) | (37.6-55.7) |
| Malta | 11.4 | 7.6 | 6.6 | 5.3 | 10.0 | 6.7 | 5.8 | 4.7 | 7.6 | 5.1 | 4.4 | 3.6 | 3.8 | 2.5 | 2.2 | 1.8 |
| | (10.6-12.2) | (7.0-8.3) | (6.0-7.3) | (4.2-6.8) | (9.3-10.7) | (6.1-7.3) | (5.3-6.4) | (3.7-6.0) | (7.0-8.2) | (4.6-5.6) | (4.0-4.9) | (2.8-4.6) | (3.4-4.2) | (2.2-2.9) | (1.9-2.5) | (1.3-2.4) |
| Marshall Islands | 47.8 | 42.7 | 35.2 | 26.7 | 37.7 | 34.5 | 29.4 | 22.7 | 18.5 | 19.3 | 16.6 | 12.5 | 29.8 | 23.8 | 18.6 | 14.0 |
| | (40.7-56.0) | (36.1-50.3) | (25.5-48.8) | (14.6-48.4) | (32.1-44.2) | (29.1-40.6) | (21.3-40.8) | (12.4-41.1) | (14.6-23.3) | (15.5-23.7) | (10.5-25.3) | (6.1-25.0) | (24.5-36.1) | (19.4-29.1) | (12.0-28.1) | (6.9-27.6) |
| Mauritania | 116.5 | 98.0 | 49.1 | 36.9 | 73.5 | 67.3 | 38.5 | 30.2 | 44.0 | 38.9 | 25.6 | 20.8 | 75.7 | 61.5 | 24.2 | 16.4 |
| | (105.5-128.6) | (89.6-107.7) | (43.1-56.0) | (25.9-53.5) | (66.5-81.1) | (61.5-74.0) | (33.8-43.8) | (21.2-43.8) | (38.9-49.8) | (34.7-43.6) | (22.0-29.7) | (14.4-30.5) | (67.6-84.6) | (55.6-68.4) | (20.7-28.1) | (11.0-24.5) |
| Mauritius | 23.1 | 18.8 | 14.5 | 15.4 | 19.9 | 16.5 | 12.9 | 13.8 | 14.7 | 12.4 | 9.1 | 9.6 | 8.5 | 6.5 | 5.5 | 5.8 |
| | (22.1-24.2) | (17.9-19.8) | (13.6-15.5) | (13.9-17.0) | (19.0-20.8) | (15.7-17.3) | (12.0-13.7) | (12.5-15.3) | (13.8-15.5) | (11.6-13.2) | (8.4-9.8) | (8.5-10.8) | (7.9-9.2) | (6.0-7.0) | (5.0-6.1) | (5.1-6.7) |
| Mexico | 44.9 | 27.4 | 16.6 | 13.1 | 38.2 | 24.0 | 15.0 | 12.1 | 22.0 | 13.9 | 8.5 | 8.2 | 23.3 | 13.7 | 8.2 | 4.8 |
| | (41.7-48.4) | (25.6-29.5) | (15.7-17.4) | (10.8-15.7) | (35.5-41.2) | (22.4-25.7) | (14.3-15.8) | (10.0-14.6) | (19.4-24.8) | (12.4-15.6) | (7.9-9.0) | (5.9-10.9) | (20.7-26.2) | (12.2-15.3) | (7.7-8.7) | (3.0-7.1) |
| Micronesia (Federated States of) | 49.2 | 38.6 | 29.8 | 22.4 | 41.9 | 33.6 | 26.7 | 20.3 | 24.1 | 20.4 | 15.7 | 11.6 | 25.6 | 18.4 | 13.6 | 10.1 |
| | (41.1-59.4) | (30.7-49.0) | (16.2-54.0) | (9.8-51.4) | (35.0-50.6) | (26.7-42.6) | (14.5-48.4) | (8.9-46.5) | (16.7-32.8) | (12.8-29.7) | (7.0-31.2) | (4.3-29.3) | (18.0-34.6) | (11.1-27.6) | (6.0-28.7) | (3.7-26.2) |
| Monaco | 7.8 | 5.2 | 3.5 | 2.7 | 6.3 | 4.2 | 2.9 | 2.2 | 4.2 | 2.8 | 1.9 | 1.4 | 3.6 | 2.4 | 1.6 | 1.2 |
| | (7.0-8.6) | (4.9-5.5) | (2.8-4.4) | (1.6-4.5) | (5.7-7.0) | (4.0-4.5) | (2.3-3.6) | (1.3-3.7) | (3.1-5.3) | (1.9-3.7) | (1.1-2.8) | (0.7-2.7) | (2.5-4.7) | (1.5-3.3) | (0.9-2.5) | (0.5-2.4) |
| Mongolia | 106.1 | 62.5 | 19.4 | 13.4 | 73.5 | 47.3 | 15.7 | 11.2 | 29.2 | 23.2 | 9.6 | 7.2 | 79.2 | 40.3 | 10.0 | 6.3 |
| | (97.7-115.2) | (57.5-68.2) | (19.0-19.8) | (12.6-14.3) | (67.7-79.8) | (43.5-51.6) | (15.4-16.0) | (10.6-11.9) | (25.3-33.6) | (20.1-26.7) | (9.0-10.1) | (6.5-7.9) | (72.1-87.1) | (36.2-44.8) | (9.4-10.5) | (5.7-7.0) |
| Montenegro | 16.5 | 14.2 | 4.2 | 2.4 | 14.8 | 12.7 | 3.5 | 1.9 | 10.9 | 8.4 | 2.3 | 0.8 | 5.6 | 5.8 | 1.9 | 1.5 |

| Country | Under-five mortality rate (U5MR) (per 1,000 live births) | | | | Infant mortality rate (IMR) (per 1,000 live births) | | | | Neonatal mortality rate (NMR) (per 1,000 live births) | | | | Mortality rate age 1–59 months (per 1,000 children aged 28 days) | | | |
|---|---|---|---|---|---|---|---|---|---|---|---|---|---|---|---|---|
| | 1990 | 2000 | 2015 | 2024 | 1990 | 2000 | 2015 | 2024 | 1990 | 2000 | 2015 | 2024 | 1990 | 2000 | 2015 | 2024 |
| | (15.7-17.3) | (13.5-15.0) | (3.8-4.6) | (2.0-2.9) | (14.1-15.6) | (12.0-13.3) | (3.2-3.9) | (1.6-2.3) | (9.8-12.0) | (7.5-9.3) | (2.0-2.7) | (0.5-1.2) | (4.6-6.7) | (5.0-6.7) | (1.5-2.2) | (1.1-2.0) |
| Montserrat | 20.5 | 14.0 | 8.5 | 6.4 | 18.6 | 13.1 | 8.0 | 6.1 | 11.0 | 7.4 | 4.5 | 3.3 | 9.4 | 6.4 | 3.8 | 2.8 |
| | (15.0-28.0) | (8.9-21.9) | (3.8-18.6) | (2.4-16.6) | (13.6-25.4) | (8.3-20.5) | (3.6-17.7) | (2.3-15.8) | (7.2-16.2) | (4.1-12.8) | (1.8-10.7) | (1.1-9.4) | (6.0-14.3) | (3.4-11.3) | (1.4-9.4) | (0.9-8.4) |
| Morocco | 81.1 | 52.4 | 23.1 | 15.7 | 65.5 | 45.4 | 21.4 | 14.7 | 36.9 | 28.4 | 14.2 | 9.6 | 45.8 | 24.7 | 9.0 | 6.2 |
| | (75.1-87.5) | (47.6-58.1) | (18.5-28.2) | (10.0-24.1) | (60.7-70.7) | (41.2-50.3) | (17.1-26.1) | (9.4-22.6) | (33.3-40.7) | (25.4-32.0) | (11.2-17.6) | (6.0-15.1) | (41.7-50.4) | (21.9-27.8) | (7.0-11.4) | (3.7-9.7) |
| Mozambique | 235.1 | 164.6 | 75.8 | 59.4 | 148.0 | 106.9 | 53.8 | 43.7 | 60.3 | 44.3 | 26.6 | 25.1 | 185.9 | 125.8 | 50.6 | 35.1 |
| | (215.4-256.5) | (152.1-178.0) | (68.1-84.5) | (46.3-76.3) | (135.6-161.5) | (98.8-115.6) | (48.4-60.0) | (34.1-56.1) | (53.2-68.4) | (39.6-49.4) | (23.1-30.4) | (18.9-33.4) | (168.9-204.7) | (115.5-137.2) | (44.9-57.2) | (26.7-46.0) |
| Myanmar | 114.6 | 88.9 | 50.5 | 36.9 | 90.5 | 73.9 | 44.1 | 32.8 | 46.4 | 36.8 | 24.4 | 19.6 | 71.3 | 54.1 | 26.6 | 17.2 |
| | (104.1-126.3) | (81.5-97.4) | (39.0-63.5) | (22.0-60.1) | (82.2-99.8) | (67.7-80.9) | (34.1-55.5) | (19.6-53.4) | (39.7-55.1) | (32.1-42.0) | (17.9-31.9) | (11.2-33.0) | (61.6-80.9) | (48.3-60.5) | (19.8-34.6) | (9.8-29.9) |
| Namibia | 73.6 | 78.2 | 51.8 | 39.5 | 56.2 | 48.0 | 45.3 | 36.4 | 28.4 | 24.0 | 24.6 | 21.4 | 46.6 | 55.5 | 27.6 | 18.6 |
| | (66.1-82.1) | (68.8-89.5) | (40.9-62.6) | (30.5-57.8) | (50.5-62.6) | (42.2-54.9) | (35.7-54.7) | (28.1-53.4) | (24.6-32.6) | (21.0-27.6) | (18.5-31.5) | (14.6-32.8) | (41.2-52.6) | (48.6-63.8) | (20.8-35.3) | (12.4-29.3) |
| Nauru | 66.9 | 41.1 | 17.5 | 8.3 | 58.8 | 37.5 | 16.2 | 7.7 | 32.0 | 24.1 | 9.4 | 4.5 | 35.8 | 17.4 | 8.1 | 3.7 |
| | (52.9-84.3) | (35.8-47.4) | (13.3-23.0) | (4.7-15.4) | (46.5-74.1) | (32.7-43.2) | (12.3-21.3) | (4.4-14.4) | (22.2-44.4) | (20.1-29.0) | (6.5-12.8) | (2.3-8.7) | (25.5-49.2) | (13.7-21.4) | (5.8-11.6) | (1.9-7.5) |
| Nepal | 138.4 | 78.7 | 37.8 | 25.1 | 101.8 | 62.0 | 32.4 | 22.2 | 57.8 | 39.7 | 22.9 | 15.3 | 85.6 | 40.7 | 15.2 | 10.0 |
| | (129.6-148.0) | (73.7-84.2) | (34.2-41.9) | (19.5-32.3) | (95.2-108.8) | (58.0-66.3) | (29.3-36.0) | (17.3-28.6) | (53.1-63.0) | (36.5-43.0) | (20.5-25.6) | (11.7-19.8) | (78.9-92.6) | (37.4-44.3) | (13.5-17.3) | (7.6-13.1) |
| Netherlands (Kingdom of the) | 8.3 | 6.2 | 4.1 | 3.9 | 6.8 | 5.1 | 3.5 | 3.4 | 4.6 | 3.8 | 2.7 | 2.6 | 3.7 | 2.4 | 1.4 | 1.3 |
| | (8.2-8.5) | (6.1-6.3) | (4.0-4.2) | (3.7-4.1) | (6.7-6.9) | (5.0-5.2) | (3.4-3.6) | (3.3-3.6) | (4.5-4.8) | (3.7-3.9) | (2.6-2.7) | (2.5-2.8) | (3.6-3.9) | (2.3-2.5) | (1.4-1.5) | (1.2-1.4) |
| New Zealand | 11.2 | 7.4 | 5.6 | 4.7 | 9.1 | 6.1 | 4.7 | 4.0 | 4.4 | 3.5 | 3.2 | 2.7 | 6.8 | 3.9 | 2.4 | 2.1 |
| | (10.9-11.5) | (7.2-7.6) | (5.4-5.8) | (4.1-5.5) | (8.9-9.4) | (5.9-6.2) | (4.6-4.9) | (3.4-4.7) | (4.1-4.6) | (3.3-3.7) | (3.0-3.4) | (2.1-3.4) | (6.5-7.1) | (3.7-4.1) | (2.3-2.6) | (1.6-2.7) |
| Nicaragua | 68.6 | 37.1 | 17.2 | 11.2 | 56.8 | 31.6 | 15.0 | 9.4 | 23.5 | 16.4 | 10.5 | 5.3 | 46.1 | 21.0 | 6.7 | 6.0 |
| | (62.9-75.0) | (33.0-41.8) | (16.3-18.3) | (10.7-11.8) | (52.1-62.0) | (28.1-35.6) | (14.3-16.0) | (9.0-9.9) | (20.7-26.6) | (14.2-19.0) | (9.8-11.3) | (5.0-5.7) | (41.7-51.0) | (18.3-24.2) | (6.2-7.3) | (5.6-6.4) |
| Niger | 332.4 | 227.9 | 125.4 | 110.7 | 147.7 | 108.3 | 69.1 | 65.6 | 55.4 | 44.1 | 34.5 | 32.8 | 293.3 | 192.4 | 94.0 | 80.2 |
| | (310.0-357.8) | (210.5-246.9) | (111.4-141.2) | (82.9-147.6) | (137.7-159.0) | (100.1-117.4) | (61.4-77.8) | (49.1-87.5) | (48.6-62.8) | (38.3-50.2) | (28.9-40.8) | (23.3-46.5) | (272.0-317.2) | (176.6-209.6) | (82.6-107.1) | (58.6-109.0) |
| Nigeria | 207.6 | 177.3 | 117.8 | 115.6 | 104.7 | 94.9 | 68.0 | 69.8 | 49.5 | 46.1 | 38.0 | 39.0 | 166.2 | 137.4 | 83.0 | 79.6 |
| | (193.5-221.9) | (167.0-188.0) | (109.1-127.3) | (98.8-136.2) | (97.6-111.9) | (89.4-100.7) | (63.0-73.5) | (59.6-82.2) | (44.4-54.9) | (41.9-50.5) | (34.2-42.2) | (31.9-48.1) | (154.0-179.1) | (128.6-146.7) | (76.1-90.5) | (66.6-95.3) |
| Niue | 25.5 | 33.1 | 31.5 | 23.8 | 22.4 | 29.9 | 29.2 | 22.2 | 13.7 | 17.7 | 16.6 | 12.4 | 11.9 | 15.3 | 14.2 | 10.7 |
| | (21.5-30.2) | (25.5-42.5) | (16.0-60.7) | (10.0-56.7) | (18.9-26.5) | (23.0-38.4) | (14.9-56.3) | (9.4-52.9) | (9.9-18.1) | (11.2-25.8) | (7.3-35.1) | (4.5-33.0) | (8.2-16.2) | (9.1-23.3) | (6.1-32.1) | (3.7-29.0) |
| North Macedonia | 36.5 | 16.0 | 11.1 | 2.8 | 32.9 | 14.2 | 9.8 | 2.5 | 17.1 | 9.2 | 8.1 | 1.1 | 19.6 | 6.9 | 3.1 | 1.7 |
| | (35.3-37.6) | (15.3-16.7) | (10.5-11.8) | (2.3-3.4) | (31.8-33.9) | (13.6-14.9) | (9.3-10.4) | (2.0-3.0) | (16.2-18.2) | (8.6-9.8) | (7.5-8.7) | (0.8-1.4) | (18.6-20.7) | (6.3-7.4) | (2.7-3.5) | (1.4-2.2) |
| Norway | 8.7 | 4.9 | 2.7 | 2.5 | 7.0 | 3.9 | 2.2 | 2.0 | 4.0 | 2.7 | 1.6 | 1.4 | 4.7 | 2.2 | 1.1 | 1.1 |
| | (8.4-9.0) | (4.7-5.1) | (2.6-2.8) | (2.3-2.8) | (6.8-7.3) | (3.8-4.1) | (2.1-2.3) | (1.9-2.3) | (3.7-4.2) | (2.5-2.9) | (1.5-1.7) | (1.3-1.6) | (4.5-5.0) | (2.0-2.3) | (1.0-1.2) | (0.9-1.2) |

| Country | Under-five mortality rate (U5MR) (per 1,000 live births) | | | | Infant mortality rate (IMR) (per 1,000 live births) | | | | Neonatal mortality rate (NMR) (per 1,000 live births) | | | | Mortality rate age 1–59 months (per 1,000 children aged 28 days) | | | |
|---|---|---|---|---|---|---|---|---|---|---|---|---|---|---|---|---|
| | 1990 | 2000 | 2015 | 2024 | 1990 | 2000 | 2015 | 2024 | 1990 | 2000 | 2015 | 2024 | 1990 | 2000 | 2015 | 2024 |
| Oman | 39.4 | 16.5 | 11.1 | 10.3 | 31.4 | 13.4 | 9.0 | 8.3 | 17.6 | 7.7 | 5.5 | 5.8 | 22.1 | 8.8 | 5.6 | 4.5 |
| | (34.4-45.5) | (14.4-18.9) | (10.8-11.4) | (9.3-11.4) | (27.5-36.3) | (11.7-15.3) | (8.7-9.3) | (7.5-9.2) | (14.2-21.4) | (5.9-9.7) | (5.0-6.1) | (5.1-6.6) | (18.5-26.8) | (7.0-11.0) | (5.1-6.1) | (3.9-5.2) |
| Pakistan | 140.4 | 107.9 | 74.8 | 56.0 | 113.0 | 88.8 | 63.6 | 48.2 | 65.1 | 57.1 | 44.8 | 36.1 | 80.5 | 53.9 | 31.4 | 20.5 |
| | (135.2-146.0) | (103.2-112.6) | (69.0-81.4) | (44.0-70.4) | (108.8-117.5) | (84.9-92.7) | (58.6-69.2) | (37.9-60.6) | (60.9-69.6) | (53.6-60.7) | (40.8-49.4) | (28.1-45.9) | (75.9-85.5) | (50.4-57.6) | (28.1-35.0) | (15.4-26.8) |
| Palau | 35.5 | 26.5 | 21.3 | 21.4 | 30.0 | 22.5 | 18.2 | 18.6 | 19.1 | 14.3 | 11.2 | 11.2 | 16.6 | 12.3 | 9.6 | 9.6 |
| | (30.5-41.0) | (22.8-30.7) | (12.8-29.1) | (9.0-36.4) | (25.8-34.7) | (19.4-26.1) | (11.0-24.9) | (7.8-31.5) | (13.7-24.8) | (9.3-19.6) | (5.3-18.1) | (3.9-22.1) | (11.7-22.5) | (7.7-17.7) | (4.5-16.4) | (3.2-19.5) |
| Panama | 30.4 | 25.7 | 17.1 | 15.3 | 24.0 | 20.5 | 13.6 | 12.0 | 15.9 | 12.5 | 7.3 | 5.3 | 14.6 | 13.2 | 9.9 | 10.0 |
| | (26.5-34.2) | (22.5-28.9) | (16.6-17.7) | (14.4-16.2) | (20.9-27.0) | (17.9-23.0) | (13.2-14.1) | (11.3-12.7) | (10.1-22.3) | (7.8-17.9) | (5.9-8.8) | (3.9-6.8) | (8.7-20.8) | (8.1-18.4) | (8.4-11.3) | (8.5-11.6) |
| Papua New Guinea | 85.0 | 72.0 | 57.3 | 39.4 | 65.6 | 56.2 | 43.6 | 31.3 | 31.6 | 30.5 | 24.2 | 20.1 | 55.1 | 42.7 | 33.8 | 19.5 |
| | (76.8-94.0) | (64.3-81.0) | (44.8-72.7) | (24.2-63.0) | (59.3-72.6) | (50.2-63.3) | (34.1-55.3) | (19.2-50.0) | (27.3-36.1) | (26.5-35.1) | (18.3-31.4) | (11.8-33.0) | (49.0-62.1) | (37.4-48.9) | (26.0-43.9) | (11.5-32.8) |
| Paraguay | 45.6 | 33.6 | 21.9 | 16.3 | 37.8 | 28.7 | 19.3 | 14.5 | 22.1 | 18.0 | 11.7 | 8.6 | 24.1 | 16.0 | 10.2 | 7.6 |
| | (40.8-50.8) | (27.9-40.5) | (12.7-38.3) | (7.6-36.0) | (33.8-42.2) | (23.8-34.6) | (11.2-33.7) | (6.7-32.1) | (19.0-25.4) | (14.4-22.3) | (6.5-21.1) | (3.8-19.7) | (20.8-27.7) | (12.6-19.9) | (5.5-18.9) | (3.2-17.6) |
| Peru | 80.6 | 38.3 | 16.6 | 12.9 | 60.0 | 30.1 | 13.8 | 11.0 | 28.0 | 16.1 | 8.2 | 6.5 | 54.1 | 22.5 | 8.5 | 6.4 |
| | (76.3-85.3) | (36.0-40.7) | (15.2-18.2) | (9.7-17.6) | (56.8-63.4) | (28.3-31.9) | (12.6-15.1) | (8.3-15.0) | (25.9-30.3) | (14.8-17.5) | (7.4-9.1) | (4.8-9.0) | (50.7-57.8) | (20.9-24.3) | (7.6-9.4) | (4.7-9.0) |
| Philippines | 55.8 | 36.8 | 27.9 | 26.5 | 38.8 | 27.3 | 22.1 | 21.7 | 19.1 | 16.6 | 13.5 | 13.6 | 37.5 | 20.5 | 14.5 | 13.0 |
| | (51.9-60.1) | (34.1-39.7) | (24.8-31.4) | (19.4-36.5) | (36.0-41.8) | (25.2-29.4) | (19.7-24.9) | (15.9-29.9) | (17.1-21.1) | (15.0-18.3) | (11.8-15.5) | (9.7-19.4) | (34.5-40.8) | (18.8-22.5) | (12.7-16.6) | (9.1-18.6) |
| Poland | 17.4 | 9.3 | 4.9 | 4.2 | 15.2 | 8.1 | 4.2 | 3.6 | 11.1 | 5.8 | 2.9 | 2.5 | 6.3 | 3.6 | 2.0 | 1.8 |
| | (17.0-17.8) | (9.1-9.5) | (4.8-5.0) | (4.1-4.4) | (14.9-15.5) | (7.9-8.3) | (4.1-4.3) | (3.5-3.8) | (10.8-11.4) | (5.6-5.9) | (2.8-3.0) | (2.3-2.6) | (6.1-6.6) | (3.4-3.7) | (1.9-2.1) | (1.7-1.9) |
| Portugal | 14.7 | 7.2 | 3.6 | 3.2 | 11.5 | 5.5 | 2.9 | 2.7 | 7.2 | 3.3 | 2.1 | 1.7 | 7.5 | 3.8 | 1.5 | 1.5 |
| | (14.4-15.0) | (7.0-7.4) | (3.5-3.8) | (3.0-3.5) | (11.3-11.8) | (5.4-5.7) | (2.8-3.1) | (2.5-2.9) | (6.9-7.6) | (3.2-3.5) | (2.0-2.2) | (1.5-1.9) | (7.2-7.9) | (3.6-4.0) | (1.4-1.6) | (1.4-1.7) |
| Qatar | 20.8 | 12.5 | 7.8 | 5.9 | 17.4 | 10.4 | 6.4 | 4.8 | 11.4 | 6.6 | 3.8 | 3.8 | 9.5 | 5.9 | 4.0 | 2.1 |
| | (19.5-22.2) | (11.9-13.1) | (7.5-8.1) | (5.4-6.4) | (16.3-18.6) | (9.9-11.0) | (6.2-6.7) | (4.5-5.3) | (9.2-13.7) | (5.9-7.3) | (3.4-4.2) | (3.4-4.2) | (7.3-11.7) | (5.3-6.7) | (3.6-4.4) | (1.8-2.4) |
| Republic of Korea | 15.7 | 7.6 | 3.5 | 2.8 | 12.0 | 5.8 | 2.9 | 2.3 | 7.4 | 3.4 | 1.6 | 1.2 | 8.3 | 4.1 | 1.9 | 1.6 |
| | (14.9-16.6) | (7.3-7.8) | (3.4-3.6) | (2.6-3.0) | (11.4-12.7) | (5.6-6.0) | (2.8-2.9) | (2.2-2.5) | (6.4-8.7) | (3.2-3.7) | (1.5-1.8) | (1.0-1.3) | (7.0-9.4) | (3.9-4.4) | (1.8-2.0) | (1.5-1.8) |
| Republic of Moldova | 33.5 | 31.4 | 15.6 | 16.5 | 29.1 | 27.9 | 14.2 | 15.1 | 18.5 | 20.5 | 11.7 | 12.3 | 15.3 | 11.0 | 3.9 | 4.2 |
| | (28.6-39.2) | (25.8-37.7) | (12.6-19.3) | (13.3-22.2) | (24.8-34.0) | (23.0-33.5) | (11.4-17.5) | (12.2-20.4) | (15.4-22.1) | (16.8-25.0) | (9.2-14.7) | (9.3-17.1) | (12.5-18.5) | (8.7-13.8) | (2.7-5.6) | (2.5-6.8) |
| Romania | 30.9 | 21.4 | 9.2 | 7.3 | 24.2 | 18.0 | 7.7 | 6.0 | 15.1 | 10.2 | 4.3 | 3.6 | 16.1 | 11.3 | 4.9 | 3.7 |
| | (30.1-31.7) | (20.8-22.0) | (8.9-9.4) | (7.0-7.7) | (23.6-24.9) | (17.5-18.4) | (7.5-7.9) | (5.7-6.3) | (12.7-17.8) | (9.2-11.3) | (3.9-4.7) | (3.3-4.0) | (13.3-18.4) | (10.2-12.3) | (4.4-5.3) | (3.3-4.1) |
| Russian Federation | 21.6 | 19.4 | 8.2 | 5.2 | 17.5 | 15.6 | 6.6 | 4.1 | 10.8 | 9.0 | 4.2 | 2.0 | 10.9 | 10.5 | 4.0 | 3.2 |
| | (21.3-22.1) | (19.0-19.7) | (8.1-8.4) | (4.9-5.4) | (17.1-17.8) | (15.3-15.9) | (6.5-6.7) | (3.9-4.3) | (8.1-13.8) | (8.0-9.9) | (3.8-4.6) | (1.7-2.3) | (7.9-13.7) | (9.5-11.4) | (3.6-4.4) | (2.9-3.5) |
| Rwanda | 150.7 | 184.2 | 47.3 | 37.7 | 86.9 | 108.1 | 33.9 | 29.4 | 41.0 | 43.1 | 19.9 | 17.4 | 114.4 | 147.4 | 27.9 | 20.6 |

| Country | Under-five mortality rate (U5MR) (per 1,000 live births) | | | | Infant mortality rate (IMR) (per 1,000 live births) | | | | Neonatal mortality rate (NMR) (per 1,000 live births) | | | | Mortality rate age 1–59 months (per 1,000 children aged 28 days) | | | |
|---|---|---|---|---|---|---|---|---|---|---|---|---|---|---|---|---|
| | 1990 | 2000 | 2015 | 2024 | 1990 | 2000 | 2015 | 2024 | 1990 | 2000 | 2015 | 2024 | 1990 | 2000 | 2015 | 2024 |
| | (140.7-161.3) | (172.1-197.2) | (39.6-56.2) | (26.7-53.2) | (81.1-93.0) | (101.0-115.7) | (28.3-40.2) | (20.8-41.4) | (36.9-45.3) | (38.8-47.7) | (16.4-24.1) | (11.9-25.1) | (106.0-123.6) | (136.9-158.9) | (23.1-33.5) | (14.3-30.0) |
| Saint Kitts and Nevis | 30.3 | 23.9 | 19.4 | 15.9 | 25.2 | 20.2 | 16.9 | 13.9 | 18.7 | 15.2 | 12.3 | 10.0 | 11.7 | 8.8 | 7.2 | 5.8 |
| | (27.6-33.3) | (21.4-26.6) | (15.9-23.7) | (9.7-25.4) | (23.0-27.7) | (18.1-22.5) | (13.8-20.6) | (8.5-22.3) | (16.5-21.2) | (13.2-17.5) | (9.7-15.4) | (5.9-16.6) | (9.9-13.8) | (7.2-10.6) | (5.4-9.4) | (3.2-10.2) |
| Saint Lucia | 20.8 | 18.5 | 18.7 | 17.2 | 17.3 | 16.0 | 16.9 | 15.8 | 12.0 | 11.4 | 13.5 | 11.3 | 8.9 | 7.1 | 5.3 | 6.0 |
| | (19.9-21.8) | (17.5-19.6) | (17.5-20.1) | (14.9-20.0) | (16.5-18.1) | (15.1-16.9) | (15.8-18.1) | (13.7-18.4) | (11.1-13.0) | (10.5-12.4) | (12.4-14.6) | (9.4-13.4) | (8.1-9.8) | (6.3-8.0) | (4.6-6.1) | (4.7-7.6) |
| Saint Vincent and the Grenadines | 23.8 | 22.6 | 17.4 | 11.3 | 19.5 | 19.3 | 15.5 | 10.2 | 12.6 | 13.3 | 11.2 | 9.0 | 11.3 | 9.3 | 6.3 | 2.3 |
| | (22.2-25.5) | (20.9-24.4) | (15.7-19.1) | (9.1-14.0) | (18.2-20.9) | (17.9-20.8) | (14.0-17.1) | (8.2-12.7) | (11.2-14.1) | (12.0-14.8) | (9.9-12.5) | (7.1-11.2) | (9.9-12.8) | (8.2-10.6) | (5.4-7.3) | (1.7-3.2) |
| Samoa | 29.6 | 21.0 | 18.3 | 14.9 | 22.9 | 16.4 | 14.8 | 12.3 | 12.9 | 8.6 | 7.3 | 5.8 | 16.8 | 12.4 | 11.1 | 9.0 |
| | (26.0-33.8) | (18.4-23.6) | (15.2-22.0) | (9.5-23.4) | (20.1-26.1) | (14.4-18.4) | (12.3-17.8) | (7.9-19.3) | (10.1-17.5) | (6.9-10.6) | (5.3-9.5) | (3.3-10.0) | (12.5-20.4) | (10.3-14.6) | (8.7-14.0) | (5.4-14.8) |
| San Marino | 12.7 | 5.2 | 1.9 | 1.3 | 11.0 | 4.9 | 1.8 | 1.2 | 6.7 | 2.5 | 0.8 | 0.5 | 5.9 | 2.7 | 1.0 | 0.7 |
| | (7.5-21.5) | (3.7-7.3) | (1.1-3.2) | (0.6-2.7) | (6.5-18.7) | (3.5-6.8) | (1.1-3.1) | (0.6-2.6) | (3.7-12.2) | (1.6-3.8) | (0.3-1.6) | (0.1-1.4) | (2.9-10.9) | (1.7-4.0) | (0.5-2.0) | (0.2-1.7) |
| Sao Tome and Principe | 108.7 | 82.2 | 23.3 | 13.6 | 65.5 | 50.7 | 15.2 | 9.2 | 27.0 | 22.2 | 11.0 | 6.4 | 83.8 | 61.4 | 12.4 | 7.0 |
| | (95.8-123.0) | (72.4-93.4) | (16.4-33.2) | (7.2-25.5) | (57.7-74.1) | (44.6-57.6) | (10.7-21.7) | (4.9-17.3) | (21.1-34.0) | (17.0-27.5) | (7.4-16.2) | (3.2-12.8) | (72.7-96.5) | (53.0-71.2) | (8.3-18.2) | (3.5-13.9) |
| Saudi Arabia | 44.1 | 21.9 | 9.0 | 6.0 | 40.6 | 19.8 | 7.3 | 4.7 | 22.0 | 11.5 | 4.5 | 2.9 | 22.5 | 10.5 | 4.5 | 3.0 |
| | (37.1-52.7) | (19.6-24.6) | (7.7-10.5) | (4.1-8.9) | (34.1-48.4) | (17.7-22.2) | (6.2-8.5) | (3.2-7.0) | (15.9-29.1) | (9.2-14.3) | (3.4-5.7) | (1.6-4.7) | (16.2-29.8) | (8.0-12.9) | (3.4-5.7) | (1.8-5.0) |
| Senegal | 137.2 | 128.9 | 52.2 | 36.5 | 73.0 | 74.0 | 37.3 | 28.9 | 39.9 | 37.8 | 25.4 | 20.9 | 101.3 | 94.6 | 27.5 | 15.9 |
| | (129.8-144.8) | (121.2-137.0) | (48.3-56.6) | (28.5-46.6) | (69.1-77.1) | (69.5-78.6) | (34.5-40.4) | (22.6-36.9) | (36.4-43.6) | (34.3-41.6) | (23.0-28.1) | (16.1-27.2) | (95.1-107.9) | (88.2-101.4) | (25.0-30.3) | (11.8-20.9) |
| Serbia | 28.0 | 12.7 | 6.3 | 5.4 | 24.2 | 11.0 | 5.4 | 4.6 | 17.0 | 7.7 | 4.0 | 3.0 | 11.2 | 5.0 | 2.3 | 2.4 |
| | (27.2-28.8) | (12.3-13.1) | (6.0-6.6) | (5.0-5.8) | (23.5-24.8) | (10.7-11.3) | (5.2-5.6) | (4.3-4.9) | (13.6-19.6) | (7.1-8.3) | (3.7-4.3) | (2.6-3.3) | (8.5-14.5) | (4.4-5.6) | (2.0-2.6) | (2.1-2.8) |
| Seychelles | 16.3 | 13.8 | 14.6 | 14.0 | 13.2 | 11.8 | 13.2 | 12.9 | 10.9 | 8.7 | 8.8 | 8.3 | 5.4 | 5.1 | 5.7 | 5.6 |
| | (14.9-17.9) | (12.4-15.3) | (13.1-16.2) | (10.4-18.6) | (12.0-14.4) | (10.6-13.1) | (11.9-14.6) | (9.6-17.2) | (9.7-12.3) | (7.6-9.9) | (7.7-10.1) | (6.0-11.5) | (4.5-6.4) | (4.3-6.0) | (4.9-6.8) | (3.9-8.1) |
| Sierra Leone | 258.0 | 222.8 | 136.8 | 90.5 | 135.4 | 123.4 | 76.9 | 54.6 | 52.0 | 48.0 | 34.0 | 28.3 | 217.3 | 183.5 | 106.3 | 63.9 |
| | (238.5-278.5) | (208.0-238.6) | (124.0-151.1) | (68.0-120.2) | (125.1-146.1) | (115.2-132.1) | (69.8-85.0) | (41.0-72.5) | (45.1-60.1) | (42.5-54.2) | (29.2-39.2) | (20.0-39.2) | (199.1-236.1) | (169.9-197.8) | (95.6-118.7) | (47.4-86.8) |
| Singapore | 7.7 | 3.9 | 2.7 | 2.7 | 6.1 | 3.0 | 2.2 | 2.2 | 4.0 | 1.6 | 1.1 | 1.1 | 3.6 | 2.2 | 1.6 | 1.6 |
| | (7.4-8.0) | (3.6-4.1) | (2.5-2.9) | (2.4-3.1) | (5.9-6.4) | (2.9-3.2) | (2.0-2.3) | (1.9-2.5) | (3.8-4.3) | (1.5-1.8) | (1.0-1.2) | (0.9-1.3) | (3.4-3.9) | (2.1-2.4) | (1.5-1.8) | (1.4-1.9) |
| Slovakia | 14.7 | 9.6 | 6.2 | 6.3 | 12.6 | 8.0 | 5.2 | 5.3 | 8.7 | 5.0 | 3.1 | 3.3 | 6.1 | 4.6 | 3.2 | 3.0 |
| | (14.4-15.1) | (9.3-9.9) | (6.0-6.4) | (5.9-6.7) | (12.4-12.9) | (7.8-8.3) | (5.0-5.4) | (5.0-5.7) | (8.3-9.0) | (4.7-5.3) | (2.9-3.2) | (3.0-3.6) | (5.8-6.4) | (4.4-4.9) | (3.0-3.4) | (2.8-3.3) |
| Slovenia | 10.4 | 5.4 | 2.6 | 2.3 | 8.8 | 4.5 | 2.1 | 1.8 | 5.8 | 3.2 | 1.5 | 1.3 | 4.6 | 2.2 | 1.1 | 1.0 |
| | (10.0-10.8) | (5.2-5.7) | (2.4-2.8) | (2.0-2.6) | (8.4-9.1) | (4.3-4.8) | (2.0-2.3) | (1.6-2.1) | (5.4-6.1) | (3.0-3.5) | (1.4-1.7) | (1.1-1.5) | (4.3-5.0) | (2.0-2.4) | (1.0-1.2) | (0.8-1.2) |
| Solomon Islands | 38.1 | 30.5 | 25.0 | 20.0 | 28.8 | 23.5 | 20.0 | 16.4 | 14.8 | 12.8 | 10.1 | 8.0 | 23.6 | 17.9 | 15.0 | 11.9 |
| | (33.3-43.6) | (27.2-34.2) | (20.2-30.8) | (13.1-30.4) | (25.2-33.0) | (21.0-26.4) | (16.2-24.7) | (10.7-24.9) | (11.7-18.3) | (10.8-15.4) | (7.4-13.5) | (4.8-13.2) | (19.7-28.0) | (15.3-20.7) | (11.6-19.2) | (7.4-19.0) |

| Country | Under-five mortality rate (U5MR) (per 1,000 live births) | | | | Infant mortality rate (IMR) (per 1,000 live births) | | | | Neonatal mortality rate (NMR) (per 1,000 live births) | | | | Mortality rate age 1–59 months (per 1,000 children aged 28 days) | | | |
|---|---|---|---|---|---|---|---|---|---|---|---|---|---|---|---|---|
| | 1990 | 2000 | 2015 | 2024 | 1990 | 2000 | 2015 | 2024 | 1990 | 2000 | 2015 | 2024 | 1990 | 2000 | 2015 | 2024 |
| Somalia | 180.4 | 173.2 | 134.3 | 101.1 | 106.3 | 103.9 | 83.7 | 65.4 | 45.5 | 44.7 | 40.3 | 34.2 | 141.3 | 134.1 | 97.5 | 68.1 |
| | (148.4-221.8) | (136.0-226.1) | (79.2-234.6) | (46.4-222.8) | (87.4-130.7) | (81.6-135.6) | (49.4-146.2) | (30.0-144.2) | (34.8-59.8) | (32.5-61.8) | (21.6-76.3) | (14.4-81.9) | (114.5-176.2) | (104.0-178.5) | (54.7-176.8) | (29.5-159.8) |
| South Africa | 58.1 | 71.4 | 38.2 | 35.1 | 52.1 | 46.5 | 29.3 | 24.2 | 20.5 | 13.5 | 11.7 | 12.4 | 38.3 | 58.5 | 26.8 | 22.9 |
| | (50.7-67.0) | (65.9-76.9) | (35.9-40.7) | (30.1-41.3) | (45.4-60.0) | (42.9-50.1) | (27.5-31.2) | (20.8-28.5) | (17.0-24.9) | (11.2-16.6) | (9.9-13.5) | (10.0-15.3) | (32.7-44.8) | (53.3-63.9) | (24.6-29.3) | (19.2-27.5) |
| South Sudan | 301.3 | 180.1 | 240.8 | 96.7 | 157.4 | 118.2 | 151.3 | 71.9 | 64.2 | 55.6 | 41.8 | 39.5 | 252.6 | 131.5 | 206.7 | 57.7 |
| | (246.7-355.1) | (151.7-212.7) | (125.8-409.7) | (24.9-283.2) | (128.9-185.5) | (99.6-139.6) | (79.0-257.3) | (18.5-210.0) | (42.9-87.8) | (40.5-75.2) | (20.8-77.8) | (9.4-124.3) | (202.4-304.8) | (105.8-160.3) | (103.7-367.9) | (13.7-189.8) |
| Spain | 9.2 | 5.4 | 3.3 | 3.2 | 7.4 | 4.4 | 2.7 | 2.6 | 4.9 | 2.8 | 1.9 | 1.7 | 4.3 | 2.7 | 1.4 | 1.5 |
| | (9.0-9.3) | (5.3-5.5) | (3.2-3.4) | (3.0-3.4) | (7.3-7.6) | (4.3-4.4) | (2.7-2.8) | (2.5-2.8) | (4.7-5.1) | (2.7-2.9) | (1.9-2.0) | (1.6-1.9) | (4.1-4.5) | (2.5-2.8) | (1.3-1.4) | (1.3-1.6) |
| Sri Lanka | 23.3 | 16.3 | 8.7 | 5.9 | 17.9 | 13.0 | 7.4 | 5.2 | 14.2 | 9.7 | 5.3 | 4.0 | 9.2 | 6.6 | 3.4 | 1.9 |
| | (22.8-23.8) | (16.0-16.6) | (8.5-9.0) | (4.9-7.0) | (17.5-18.3) | (12.8-13.3) | (7.2-7.6) | (4.3-6.2) | (13.6-14.9) | (9.4-10.0) | (5.1-5.6) | (3.2-4.8) | (8.6-9.8) | (6.3-6.9) | (3.2-3.6) | (1.5-2.5) |
| State of Palestine | 45.1 | 30.3 | 18.2 | 37.9 | 39.2 | 26.3 | 15.8 | 17.0 | 22.5 | 17.1 | 11.5 | 8.9 | 23.1 | 13.4 | 6.7 | 29.2 |
| | (41.4-48.8) | (27.9-32.9) | (15.1-22.1) | (25.5-56.8) | (36.0-42.4) | (24.2-28.6) | (13.1-19.2) | (11.4-25.5) | (19.9-25.2) | (15.3-19.0) | (9.4-14.2) | (5.9-13.4) | (20.5-25.9) | (11.9-15.1) | (5.3-8.5) | (19.4-44.2) |
| Sudan | 130.9 | 102.0 | 63.5 | 61.6 | 84.7 | 70.5 | 48.4 | 42.9 | 42.4 | 36.3 | 28.2 | 23.7 | 92.2 | 68.2 | 36.1 | 38.6 |
| | (120.7-141.9) | (93.2-111.8) | (52.4-76.5) | (39.0-96.3) | (78.1-91.9) | (64.4-77.3) | (40.0-58.4) | (27.2-67.1) | (37.9-47.6) | (32.0-40.9) | (22.6-35.0) | (14.8-37.8) | (84.3-101.2) | (61.4-75.6) | (29.1-44.6) | (23.9-62.0) |
| Suriname | 44.8 | 31.0 | 20.6 | 15.8 | 37.3 | 27.3 | 19.0 | 14.8 | 20.5 | 16.9 | 12.9 | 9.7 | 24.8 | 14.2 | 7.7 | 5.9 |
| | (38.4-52.7) | (26.2-36.5) | (14.8-28.6) | (9.1-28.3) | (32.0-43.9) | (23.0-32.2) | (13.7-26.4) | (8.5-26.4) | (12.3-27.5) | (12.9-21.2) | (8.9-18.6) | (4.8-18.8) | (18.4-33.9) | (10.9-18.6) | (4.8-11.7) | (2.2-12.8) |
| Sweden | 7.0 | 4.1 | 2.9 | 2.4 | 5.9 | 3.4 | 2.3 | 2.0 | 3.5 | 2.3 | 1.6 | 1.4 | 3.5 | 1.8 | 1.3 | 1.0 |
| | (6.8-7.2) | (4.0-4.3) | (2.8-3.0) | (2.2-2.6) | (5.7-6.0) | (3.3-3.5) | (2.3-2.4) | (1.8-2.1) | (3.3-3.7) | (2.2-2.4) | (1.5-1.6) | (1.3-1.6) | (3.3-3.7) | (1.7-1.9) | (1.2-1.4) | (0.9-1.1) |
| Switzerland | 8.2 | 5.6 | 4.3 | 3.9 | 6.6 | 4.7 | 3.7 | 3.4 | 3.9 | 3.5 | 3.0 | 2.8 | 4.3 | 2.2 | 1.3 | 1.1 |
| | (7.9-8.4) | (5.5-5.8) | (4.1-4.4) | (3.6-4.1) | (6.4-6.8) | (4.5-4.8) | (3.6-3.9) | (3.2-3.6) | (3.7-4.1) | (3.3-3.6) | (2.8-3.1) | (2.6-3.0) | (4.1-4.5) | (2.1-2.3) | (1.2-1.4) | (1.0-1.2) |
| Syrian Arab Republic | 36.9 | 23.1 | 37.1 | 18.4 | 31.6 | 20.8 | 25.5 | 17.7 | 16.7 | 12.2 | 10.6 | 9.5 | 20.5 | 11.0 | 26.7 | 8.7 |
| | (33.3-40.8) | (20.8-25.7) | (24.4-45.3) | (9.2-28.8) | (28.6-35.0) | (18.7-23.2) | (16.8-31.1) | (8.8-27.6) | (14.4-19.2) | (10.6-14.1) | (7.0-13.4) | (4.6-15.7) | (17.9-23.3) | (9.5-12.8) | (17.5-32.9) | (4.2-14.6) |
| Tajikistan | 99.2 | 78.4 | 32.2 | 28.1 | 76.7 | 61.5 | 26.3 | 23.5 | 29.7 | 26.2 | 13.1 | 11.1 | 71.5 | 53.5 | 19.4 | 17.2 |
| | (90.4-108.7) | (70.8-86.9) | (27.8-37.3) | (18.7-42.5) | (69.9-84.0) | (55.5-68.1) | (22.7-30.5) | (15.6-35.5) | (24.8-36.0) | (22.6-30.3) | (10.8-15.6) | (7.0-17.6) | (63.4-79.8) | (47.7-60.0) | (16.4-22.8) | (11.1-26.7) |
| Thailand | 36.9 | 21.7 | 11.9 | 9.0 | 30.9 | 18.3 | 10.1 | 7.7 | 20.7 | 12.2 | 6.6 | 5.1 | 16.5 | 9.5 | 5.1 | 4.0 |
| | (34.4-39.4) | (19.0-23.9) | (10.2-13.1) | (7.9-11.5) | (28.8-33.0) | (16.0-20.2) | (8.7-11.1) | (6.8-9.9) | (16.6-23.6) | (8.6-14.9) | (4.1-8.6) | (3.0-7.2) | (13.8-20.6) | (7.1-13.2) | (3.4-7.8) | (2.5-6.4) |
| Timor-Leste | 228.0 | 111.5 | 60.2 | 47.6 | 165.5 | 84.4 | 43.6 | 34.6 | 58.8 | 39.5 | 24.9 | 21.6 | 179.8 | 75.0 | 35.7 | 26.4 |
| | (204.7-254.4) | (100.9-122.7) | (49.0-70.7) | (32.5-72.4) | (148.6-184.7) | (76.4-92.9) | (35.5-51.2) | (23.6-52.6) | (50.2-69.4) | (34.0-45.4) | (17.6-33.1) | (12.8-35.4) | (159.1-203.0) | (66.8-83.8) | (27.1-45.3) | (16.1-42.8) |
| Togo | 147.3 | 119.2 | 75.2 | 56.1 | 85.4 | 70.8 | 45.4 | 34.8 | 43.7 | 36.5 | 26.7 | 22.6 | 108.2 | 85.8 | 49.8 | 34.3 |
| | (135.9-159.5) | (110.7-128.8) | (65.6-86.3) | (38.7-81.0) | (78.8-92.5) | (65.7-76.4) | (39.6-52.0) | (24.0-50.3) | (39.0-49.5) | (32.6-41.1) | (22.2-31.9) | (14.9-33.6) | (98.9-118.2) | (78.8-93.4) | (42.6-58.1) | (23.1-51.0) |
| Tonga | 22.1 | 16.9 | 11.9 | 9.5 | 18.0 | 13.8 | 9.6 | 7.8 | 9.8 | 7.2 | 5.1 | 4.1 | 12.4 | 9.8 | 6.7 | 5.3 |

| | Under-five mortality rate (U5MR) (per 1,000 live births) | | | | Infant mortality rate (IMR) (per 1,000 live births) | | | | Neonatal mortality rate (NMR) (per 1,000 live births) | | | | Mortality rate age 1–59 months (per 1,000 children aged 28 days) | | | |
|---|---|---|---|---|---|---|---|---|---|---|---|---|---|---|---|---|
| Country | 1990 | 2000 | 2015 | 2024 | 1990 | 2000 | 2015 | 2024 | 1990 | 2000 | 2015 | 2024 | 1990 | 2000 | 2015 | 2024 |
| | (18.6-26.3) | (14.6-19.6) | (9.4-14.9) | (6.1-14.8) | (15.1-21.4) | (11.9-16.0) | (7.6-12.1) | (5.0-12.1) | (7.5-12.7) | (5.8-8.7) | (3.8-6.8) | (2.4-7.1) | (9.7-15.4) | (8.1-11.7) | (5.1-8.7) | (3.1-8.7) |
| Trinidad and Tobago | 32.3 | 28.8 | 22.7 | 18.8 | 29.4 | 26.3 | 20.6 | 17.0 | 21.7 | 19.8 | 15.5 | 12.6 | 10.7 | 9.3 | 7.4 | 6.1 |
| | (27.6-37.0) | (24.9-33.9) | (17.2-30.5) | (11.7-30.3) | (25.2-33.8) | (22.6-30.9) | (15.6-27.6) | (10.6-27.4) | (18.3-25.3) | (16.8-23.5) | (11.5-20.9) | (7.8-20.7) | (8.6-13.0) | (7.5-11.4) | (5.3-10.2) | (3.6-10.4) |
| Tunisia | 54.9 | 29.2 | 18.2 | 12.1 | 37.2 | 23.9 | 16.0 | 9.7 | 27.2 | 17.1 | 12.1 | 7.7 | 28.4 | 12.3 | 6.2 | 4.3 |
| | (48.0-62.7) | (25.6-33.1) | (17.7-18.7) | (11.2-13.1) | (32.5-42.5) | (21.0-27.1) | (15.6-16.5) | (9.0-10.6) | (23.3-31.8) | (14.7-19.7) | (11.5-12.7) | (6.7-8.8) | (24.1-33.3) | (10.4-14.4) | (5.7-6.7) | (3.5-5.3) |
| Turkmenistan | 77.9 | 68.7 | 43.2 | 39.0 | 68.1 | 57.5 | 33.9 | 30.4 | 25.9 | 29.4 | 23.7 | 22.3 | 53.4 | 40.5 | 19.8 | 16.9 |
| | (68.7-89.5) | (59.8-79.8) | (33.9-54.3) | (23.1-65.7) | (60.0-78.3) | (50.1-66.7) | (26.6-42.7) | (18.1-51.3) | (21.6-31.2) | (24.7-35.1) | (18.2-30.4) | (12.7-38.9) | (46.2-62.4) | (34.4-48.0) | (15.0-25.9) | (9.3-30.1) |
| Turks and Caicos Islands | 20.8 | 11.7 | 6.2 | 4.7 | 15.4 | 8.2 | 4.1 | 3.0 | 11.1 | 6.3 | 3.2 | 2.4 | 9.6 | 5.4 | 2.8 | 2.0 |
| | (14.0-31.2) | (9.3-14.8) | (2.9-12.7) | (1.4-14.0) | (10.3-23.0) | (6.5-10.3) | (1.9-8.3) | (0.9-9.1) | (6.9-17.8) | (4.0-9.1) | (1.3-7.5) | (0.7-8.0) | (5.7-15.5) | (3.3-8.1) | (1.1-6.5) | (0.6-7.0) |
| Tuvalu | 54.0 | 42.8 | 26.0 | 19.2 | 45.0 | 36.1 | 22.2 | 16.5 | 28.8 | 24.8 | 12.0 | 8.7 | 25.7 | 18.4 | 14.1 | 10.4 |
| | (45.5-64.2) | (38.7-47.4) | (18.0-37.4) | (10.0-36.3) | (38.0-53.5) | (32.7-40.0) | (15.3-31.9) | (8.6-31.2) | (22.8-36.1) | (21.4-28.6) | (8.1-17.7) | (4.2-17.1) | (19.9-32.9) | (15.4-21.8) | (9.3-20.9) | (5.2-20.7) |
| Türkiye | 73.9 | 37.2 | 13.3 | 9.6 | 60.9 | 30.7 | 11.0 | 8.0 | 32.4 | 18.1 | 6.5 | 4.6 | 42.8 | 19.4 | 6.8 | 5.0 |
| | (68.7-79.7) | (34.0-40.9) | (12.3-14.3) | (8.3-11.3) | (56.6-65.7) | (28.1-33.7) | (10.1-11.8) | (6.9-9.4) | (29.3-35.9) | (16.1-20.3) | (6.0-7.1) | (4.0-5.5) | (39.0-47.0) | (17.4-21.8) | (6.3-7.4) | (4.3-5.9) |
| Uganda | 182.2 | 143.9 | 57.9 | 48.7 | 107.1 | 89.3 | 40.3 | 35.9 | 39.2 | 32.3 | 23.3 | 21.3 | 148.8 | 115.2 | 35.4 | 27.9 |
| | (170.3-194.8) | (134.7-153.3) | (52.1-64.2) | (40.0-58.5) | (100.1-114.5) | (83.6-95.1) | (36.3-44.7) | (29.5-43.2) | (34.9-43.7) | (28.9-36.1) | (20.5-26.6) | (16.6-26.7) | (138.3-160.3) | (107.2-123.5) | (31.4-39.7) | (22.1-34.5) |
| Ukraine | 19.2 | 18.2 | 9.5 | 7.9 | 18.3 | 17.6 | 9.2 | 7.6 | 11.8 | 10.9 | 5.6 | 4.5 | 7.5 | 7.4 | 3.9 | 3.3 |
| | (17.3-21.9) | (16.7-20.2) | (9.2-9.8) | (6.9-9.1) | (16.5-20.9) | (16.1-19.5) | (8.9-9.5) | (6.7-8.8) | (9.9-14.0) | (9.1-12.8) | (4.0-6.8) | (3.0-6.0) | (5.8-9.4) | (5.9-9.2) | (2.7-5.5) | (2.1-4.9) |
| United Arab Emirates | 15.1 | 10.7 | 7.6 | 4.8 | 11.8 | 8.5 | 6.1 | 3.8 | 7.9 | 5.9 | 4.2 | 2.4 | 7.3 | 4.9 | 3.5 | 2.4 |
| | (14.0-16.4) | (10.2-11.4) | (7.2-8.1) | (4.1-5.6) | (10.9-12.8) | (8.0-9.0) | (5.7-6.4) | (3.2-4.4) | (7.1-8.7) | (5.4-6.4) | (3.8-4.5) | (1.9-2.9) | (6.6-8.1) | (4.4-5.4) | (3.2-3.8) | (1.9-3.0) |
| United Kingdom | 9.3 | 6.5 | 4.5 | 4.7 | 7.9 | 5.5 | 3.9 | 4.1 | 4.5 | 3.8 | 2.6 | 3.0 | 4.8 | 2.8 | 1.8 | 1.6 |
| | (9.1-9.5) | (6.4-6.7) | (4.4-4.5) | (4.4-4.9) | (7.7-8.0) | (5.4-5.7) | (3.8-3.9) | (3.9-4.4) | (4.3-4.6) | (3.7-3.9) | (2.6-2.7) | (2.9-3.2) | (4.7-5.0) | (2.7-2.9) | (1.8-1.9) | (1.5-1.8) |
| United Republic of Tanzania | 167.9 | 128.0 | 54.3 | 37.0 | 106.2 | 85.2 | 40.8 | 28.7 | 40.0 | 32.6 | 23.4 | 19.9 | 133.1 | 98.6 | 31.5 | 17.3 |
| | (156.9-179.1) | (119.8-136.8) | (49.1-60.1) | (28.5-47.8) | (99.3-113.3) | (79.7-91.0) | (36.8-45.1) | (22.1-37.1) | (35.8-44.3) | (29.3-36.2) | (20.7-26.7) | (14.8-26.6) | (123.7-142.9) | (91.5-106.0) | (28.0-35.5) | (12.5-23.5) |
| United States | 11.2 | 8.4 | 6.8 | 6.5 | 9.4 | 7.1 | 5.8 | 5.5 | 5.8 | 4.6 | 3.9 | 3.7 | 5.5 | 3.8 | 2.9 | 2.8 |
| | (11.0-11.4) | (8.3-8.6) | (6.7-6.9) | (6.2-6.7) | (9.2-9.6) | (7.0-7.2) | (5.7-5.8) | (5.3-5.7) | (5.6-5.9) | (4.5-4.7) | (3.8-4.0) | (3.5-3.8) | (5.4-5.7) | (3.7-3.9) | (2.8-3.0) | (2.7-3.0) |
| Uruguay | 23.6 | 17.0 | 8.9 | 7.4 | 20.7 | 14.7 | 7.5 | 6.2 | 11.8 | 8.5 | 5.0 | 4.0 | 11.9 | 8.6 | 3.9 | 3.4 |
| | (23.1-24.1) | (16.6-17.4) | (8.6-9.2) | (6.8-8.1) | (20.3-21.2) | (14.4-15.1) | (7.3-7.8) | (5.6-6.7) | (10.2-13.4) | (7.9-9.1) | (4.6-5.3) | (3.5-4.7) | (10.3-13.6) | (8.0-9.2) | (3.6-4.2) | (2.9-4.0) |
| Uzbekistan | 69.8 | 60.7 | 19.7 | 13.4 | 60.2 | 54.8 | 18.6 | 12.8 | 30.3 | 28.1 | 11.0 | 7.7 | 40.6 | 33.5 | 8.8 | 5.8 |
| | (61.8-78.4) | (53.4-69.1) | (18.0-20.8) | (12.2-15.4) | (53.3-67.6) | (48.2-62.4) | (17.0-19.6) | (11.6-14.7) | (25.9-35.4) | (24.3-32.8) | (9.8-12.0) | (6.5-9.2) | (35.1-46.8) | (28.9-38.8) | (7.7-9.7) | (4.7-7.1) |
| Vanuatu | 35.1 | 26.6 | 18.6 | 17.1 | 27.6 | 21.3 | 15.4 | 14.4 | 19.5 | 14.3 | 11.0 | 9.0 | 15.8 | 12.4 | 7.7 | 7.9 |
| | (29.5-41.7) | (22.9-30.9) | (15.4-22.4) | (11.5-24.8) | (23.2-32.8) | (18.3-24.7) | (12.8-18.5) | (9.7-20.9) | (11.8-28.3) | (11.1-18.6) | (8.2-15.1) | (3.6-15.3) | (8.2-24.3) | (8.8-15.8) | (4.4-10.6) | (3.8-14.6) |

| Country | Under-five mortality rate (U5MR) (per 1,000 live births) | | | | Infant mortality rate (IMR) (per 1,000 live births) | | | | Neonatal mortality rate (NMR) (per 1,000 live births) | | | | Mortality rate age 1–59 months (per 1,000 children aged 28 days) | | | |
|---|---|---|---|---|---|---|---|---|---|---|---|---|---|---|---|---|
| | 1990 | 2000 | 2015 | 2024 | 1990 | 2000 | 2015 | 2024 | 1990 | 2000 | 2015 | 2024 | 1990 | 2000 | 2015 | 2024 |
| Venezuela (Bolivarian Republic of) | 29.6 | 21.5 | 19.1 | 24.2 | 24.3 | 17.5 | 16.5 | 21.2 | 12.8 | 10.8 | 14.6 | 15.0 | 17.0 | 10.8 | 4.5 | 9.0 |
| | (28.8-30.4) | (21.1-21.9) | (18.2-20.2) | (15.1-38.8) | (23.7-25.0) | (17.2-17.9) | (15.7-17.5) | (13.2-33.9) | (12.1-13.5) | (10.3-11.4) | (14.0-15.4) | (10.0-23.2) | (16.3-17.8) | (10.3-11.4) | (4.0-5.1) | (3.4-18.7) |
| Viet Nam | 51.6 | 30.3 | 21.6 | 17.3 | 37.4 | 22.1 | 15.5 | 12.0 | 23.7 | 15.3 | 11.1 | 8.8 | 28.5 | 15.2 | 10.7 | 8.5 |
| | (47.2-56.4) | (25.5-34.8) | (20.4-23.0) | (15.2-19.7) | (34.3-40.9) | (18.6-25.5) | (14.6-16.4) | (10.6-13.7) | (21.1-26.9) | (12.7-18.0) | (9.4-12.9) | (7.0-11.1) | (25.3-31.9) | (12.5-17.9) | (8.9-12.4) | (6.6-10.6) |
| Yemen | 125.2 | 92.9 | 49.3 | 38.1 | 96.9 | 75.8 | 41.6 | 33.8 | 43.8 | 36.7 | 24.2 | 20.9 | 85.1 | 58.4 | 25.7 | 17.6 |
| | (116.7-134.3) | (85.4-101.0) | (43.3-56.3) | (29.5-49.6) | (90.4-103.9) | (69.6-82.4) | (36.5-47.5) | (26.2-44.0) | (40.0-47.9) | (33.2-40.6) | (21.0-27.9) | (16.0-27.3) | (78.7-92.1) | (53.1-64.0) | (22.3-29.7) | (13.3-23.5) |
| Zambia | 180.7 | 151.8 | 60.2 | 48.4 | 102.3 | 89.3 | 42.4 | 34.6 | 35.5 | 33.0 | 23.4 | 21.4 | 150.5 | 122.7 | 37.6 | 27.5 |
| | (168.7-193.6) | (141.1-163.2) | (54.4-66.3) | (39.0-60.7) | (95.5-109.6) | (83.1-96.0) | (38.3-46.7) | (27.9-43.4) | (31.9-39.4) | (29.8-36.4) | (20.7-26.3) | (16.8-27.4) | (139.9-162.0) | (113.6-132.6) | (33.6-41.9) | (21.7-35.0) |
| Zimbabwe | 84.0 | 99.4 | 59.2 | 64.7 | 54.0 | 58.3 | 54.7 | 62.4 | 20.4 | 23.0 | 27.3 | 33.7 | 64.9 | 78.2 | 32.8 | 31.8 |
| | (75.8-93.1) | (89.8-110.0) | (52.5-66.7) | (44.0-93.2) | (48.7-59.8) | (52.7-64.5) | (48.5-61.6) | (42.5-90.0) | (18.0-23.1) | (20.5-25.8) | (23.8-31.4) | (22.5-49.7) | (58.3-72.3) | (70.5-86.9) | (28.6-37.5) | (20.8-47.9) |

## 3.5. Table A.5: Country deaths, 1990–2024

Number of under-five deaths, infant deaths, neonatal deaths, and deaths age 1–59 months (thousands) for 1990, 2000, 2015, and 2024, by country. Values shown are medians with 90% uncertainty intervals in parentheses.

| Country | Number of under-five deaths (thousands) | | | | Number of infant deaths (thousands) | | | | Number of neonatal deaths (thousands) | | | | Number of deaths age 1 to 59 months (thousands) | | | |
|---|---|---|---|---|---|---|---|---|---|---|---|---|---|---|---|---|
| | 1990 | 2000 | 2015 | 2024 | 1990 | 2000 | 2015 | 2024 | 1990 | 2000 | 2015 | 2024 | 1990 | 2000 | 2015 | 2024 |
| Afghanistan | 110 | 131 | 93 | 78 | 89 | 111 | 83 | 71 | 48 | 64 | 55 | 49 | 62 | 67 | 38 | 28 |
| | (100-120) | (122-142) | (83-104) | (58-104) | (81-98) | (103-120) | (74-93) | (53-95) | (43-54) | (59-71) | (48-63) | (37-67) | (55-68) | (61-74) | (33-43) | (21-39) |
| Albania | 3 | 2 | 0 | 0 | 2 | 1 | 0 | 0 | 1 | 1 | 0 | 0 | 2 | 1 | 0 | 0 |
| | (3-4) | (1-2) | (0-0) | (0-0) | (2-3) | (1-1) | (0-0) | (0-0) | (1-1) | (1-1) | (0-0) | (0-0) | (2-3) | (1-1) | (0-0) | (0-0) |
| Algeria | 40 | 25 | 25 | 19 | 30 | 21 | 22 | 17 | 18 | 14 | 16 | 13 | 21 | 11 | 10 | 6 |
| | (37-43) | (24-27) | (24-27) | (17-22) | (28-33) | (19-22) | (21-24) | (15-20) | (16-21) | (12-15) | (15-17) | (12-15) | (19-24) | (10-13) | (9-10) | (5-7) |
| Andorra | 0 | 0 | 0 | 0 | 0 | 0 | 0 | 0 | 0 | 0 | 0 | 0 | 0 | 0 | 0 | 0 |
| | (0-0) | (0-0) | (0-0) | (0-0) | (0-0) | (0-0) | (0-0) | (0-0) | (0-0) | (0-0) | (0-0) | (0-0) | (0-0) | (0-0) | (0-0) | (0-0) |
| Angola | 117 | 132 | 70 | 67 | 67 | 80 | 45 | 45 | 27 | 30 | 25 | 25 | 90 | 103 | 45 | 42 |
| | (104-132) | (117-150) | (57-86) | (46-104) | (59-75) | (70-90) | (36-55) | (30-69) | (22-36) | (24-35) | (19-31) | (16-41) | (78-103) | (90-117) | (36-57) | (27-65) |
| Anguilla | 0 | 0 | 0 | 0 | 0 | 0 | 0 | 0 | 0 | 0 | 0 | 0 | 0 | 0 | 0 | 0 |
| | (0-0) | (0-0) | (0-0) | (0-0) | (0-0) | (0-0) | (0-0) | (0-0) | (0-0) | (0-0) | (0-0) | (0-0) | (0-0) | (0-0) | (0-0) | (0-0) |
| Antigua and Barbuda | 0 | 0 | 0 | 0 | 0 | 0 | 0 | 0 | 0 | 0 | 0 | 0 | 0 | 0 | 0 | 0 |
| | (0-0) | (0-0) | (0-0) | (0-0) | (0-0) | (0-0) | (0-0) | (0-0) | (0-0) | (0-0) | (0-0) | (0-0) | (0-0) | (0-0) | (0-0) | (0-0) |
| Argentina | 20 | 14 | 9 | 5 | 18 | 12 | 8 | 4 | 11 | 8 | 5 | 3 | 10 | 6 | 4 | 2 |
| | (20-21) | (14-14) | (9-9) | (4-6) | (17-18) | (12-12) | (7-8) | (4-5) | (10-11) | (8-8) | (5-5) | (2-3) | (9-10) | (6-6) | (4-4) | (2-3) |
| Armenia | 4 | 1 | 1 | 0 | 4 | 1 | 1 | 0 | 2 | 1 | 0 | 0 | 2 | 1 | 0 | 0 |
| | (4-5) | (1-1) | (1-1) | (0-0) | (3-4) | (1-1) | (0-1) | (0-0) | (2-2) | (1-1) | (0-0) | (0-0) | (2-2) | (0-1) | (0-0) | (0-0) |

| | Number of under-five deaths (thousands) | | | | Number of infant deaths (thousands) | | | | Number of neonatal deaths (thousands) | | | | Number of deaths age 1 to 59 months (thousands) | | | |
|---|---|---|---|---|---|---|---|---|---|---|---|---|---|---|---|---|
| Country | 1990 | 2000 | 2015 | 2024 | 1990 | 2000 | 2015 | 2024 | 1990 | 2000 | 2015 | 2024 | 1990 | 2000 | 2015 | 2024 |
| Australia | 2 | 2 | 1 | 1 | 2 | 1 | 1 | 1 | 1 | 1 | 1 | 1 | 1 | 1 | 0 | 0 |
| | (2-2) | (2-2) | (1-1) | (1-1) | (2-2) | (1-1) | (1-1) | (1-1) | (1-1) | (1-1) | (1-1) | (1-1) | (1-1) | (1-1) | (0-0) | (0-0) |
| Austria | 1 | 0 | 0 | 0 | 1 | 0 | 0 | 0 | 0 | 0 | 0 | 0 | 0 | 0 | 0 | 0 |
| | (1-1) | (0-0) | (0-0) | (0-0) | (1-1) | (0-0) | (0-0) | (0-0) | (0-0) | (0-0) | (0-0) | (0-0) | (0-0) | (0-0) | (0-0) | (0-0) |
| Azerbaijan | 20 | 9 | 5 | 2 | 16 | 8 | 4 | 2 | 6 | 4 | 3 | 2 | 14 | 5 | 2 | 1 |
| | (18-22) | (8-11) | (4-7) | (1-4) | (14-17) | (7-8) | (3-5) | (1-2) | (5-7) | (4-5) | (2-4) | (1-2) | (12-16) | (4-6) | (2-3) | (0-1) |
| Bahamas | 0 | 0 | 0 | 0 | 0 | 0 | 0 | 0 | 0 | 0 | 0 | 0 | 0 | 0 | 0 | 0 |
| | (0-0) | (0-0) | (0-0) | (0-0) | (0-0) | (0-0) | (0-0) | (0-0) | (0-0) | (0-0) | (0-0) | (0-0) | (0-0) | (0-0) | (0-0) | (0-0) |
| Bahrain | 0 | 0 | 0 | 0 | 0 | 0 | 0 | 0 | 0 | 0 | 0 | 0 | 0 | 0 | 0 | 0 |
| | (0-0) | (0-0) | (0-0) | (0-0) | (0-0) | (0-0) | (0-0) | (0-0) | (0-0) | (0-0) | (0-0) | (0-0) | (0-0) | (0-0) | (0-0) | (0-0) |
| Bangladesh | 572 | 332 | 124 | 105 | 373 | 235 | 95 | 85 | 258 | 173 | 76 | 62 | 314 | 160 | 48 | 43 |
| | (553-592) | (321-343) | (118-129) | (99-114) | (361-387) | (227-242) | (92-100) | (79-92) | (243-272) | (164-181) | (72-80) | (56-69) | (299-330) | (151-168) | (44-51) | (39-49) |
| Barbados | 0 | 0 | 0 | 0 | 0 | 0 | 0 | 0 | 0 | 0 | 0 | 0 | 0 | 0 | 0 | 0 |
| | (0-0) | (0-0) | (0-0) | (0-0) | (0-0) | (0-0) | (0-0) | (0-0) | (0-0) | (0-0) | (0-0) | (0-0) | (0-0) | (0-0) | (0-0) | (0-0) |
| Belarus | 2 | 1 | 0 | 0 | 2 | 1 | 0 | 0 | 1 | 0 | 0 | 0 | 1 | 1 | 0 | 0 |
| | (2-2) | (1-1) | (0-0) | (0-0) | (2-2) | (1-1) | (0-0) | (0-0) | (1-2) | (0-1) | (0-0) | (0-0) | (1-1) | (1-1) | (0-0) | (0-0) |
| Belgium | 1 | 1 | 1 | 0 | 1 | 1 | 0 | 0 | 1 | 0 | 0 | 0 | 1 | 0 | 0 | 0 |
| | (1-1) | (1-1) | (0-1) | (0-0) | (1-1) | (1-1) | (0-0) | (0-0) | (1-1) | (0-0) | (0-0) | (0-0) | (1-1) | (0-0) | (0-0) | (0-0) |
| Belize | 0 | 0 | 0 | 0 | 0 | 0 | 0 | 0 | 0 | 0 | 0 | 0 | 0 | 0 | 0 | 0 |
| | (0-0) | (0-0) | (0-0) | (0-0) | (0-0) | (0-0) | (0-0) | (0-0) | (0-0) | (0-0) | (0-0) | (0-0) | (0-0) | (0-0) | (0-0) | (0-0) |
| Benin | 39 | 39 | 41 | 35 | 23 | 24 | 25 | 22 | 11 | 12 | 14 | 13 | 29 | 28 | 27 | 22 |
| | (37-42) | (37-42) | (37-46) | (26-48) | (22-25) | (22-25) | (22-28) | (16-29) | (10-12) | (10-13) | (12-16) | (9-18) | (27-31) | (26-30) | (24-30) | (16-31) |
| Bhutan | 3 | 1 | 0 | 0 | 2 | 1 | 0 | 0 | 1 | 0 | 0 | 0 | 2 | 1 | 0 | 0 |
| | (2-3) | (1-1) | (0-0) | (0-0) | (2-2) | (1-1) | (0-0) | (0-0) | (1-1) | (0-1) | (0-0) | (0-0) | (1-2) | (1-1) | (0-0) | (0-0) |
| Bolivia (Plurinational State of) | 30 | 18 | 7 | 4 | 22 | 15 | 6 | 4 | 10 | 7 | 3 | 2 | 19 | 11 | 4 | 2 |
| | (28-31) | (17-19) | (6-8) | (3-6) | (21-24) | (14-16) | (5-7) | (3-5) | (9-11) | (6-8) | (3-4) | (1-3) | (18-21) | (10-12) | (3-4) | (2-3) |
| Bosnia and Herzegovina | 1 | 0 | 0 | 0 | 1 | 0 | 0 | 0 | 1 | 0 | 0 | 0 | 0 | 0 | 0 | 0 |
| | (1-1) | (0-0) | (0-0) | (0-0) | (1-1) | (0-0) | (0-0) | (0-0) | (1-1) | (0-0) | (0-0) | (0-0) | (0-1) | (0-0) | (0-0) | (0-0) |
| Botswana | 2 | 4 | 3 | 2 | 2 | 2 | 3 | 2 | 1 | 0 | 1 | 1 | 1 | 3 | 1 | 1 |
| | (2-3) | (3-5) | (1-6) | (1-7) | (1-2) | (1-3) | (1-5) | (1-6) | (1-1) | (0-1) | (1-3) | (0-4) | (1-2) | (2-5) | (1-3) | (0-3) |
| Brazil | 233 | 120 | 47 | 37 | 192 | 101 | 41 | 32 | 93 | 62 | 28 | 18 | 141 | 58 | 19 | 19 |
| | (218-251) | (112-129) | (46-49) | (32-43) | (179-206) | (95-108) | (39-42) | (27-37) | (81-105) | (54-71) | (27-30) | (15-22) | (128-156) | (50-66) | (18-20) | (16-22) |
| British Virgin Islands | 0 | 0 | 0 | 0 | 0 | 0 | 0 | 0 | 0 | 0 | 0 | 0 | 0 | 0 | 0 | 0 |
| | (0-0) | (0-0) | (0-0) | (0-0) | (0-0) | (0-0) | (0-0) | (0-0) | (0-0) | (0-0) | (0-0) | (0-0) | (0-0) | (0-0) | (0-0) | (0-0) |
| Brunei Darussalam | 0 | 0 | 0 | 0 | 0 | 0 | 0 | 0 | 0 | 0 | 0 | 0 | 0 | 0 | 0 | 0 |
| | (0-0) | (0-0) | (0-0) | (0-0) | (0-0) | (0-0) | (0-0) | (0-0) | (0-0) | (0-0) | (0-0) | (0-0) | (0-0) | (0-0) | (0-0) | (0-0) |

| | Number of under-five deaths (thousands) | | | | Number of infant deaths (thousands) | | | | Number of neonatal deaths (thousands) | | | | Number of deaths age 1 to 59 months (thousands) | | | |
|---|---|---|---|---|---|---|---|---|---|---|---|---|---|---|---|---|
| Country | 1990 | 2000 | 2015 | 2024 | 1990 | 2000 | 2015 | 2024 | 1990 | 2000 | 2015 | 2024 | 1990 | 2000 | 2015 | 2024 |
| Bulgaria | 2 | 1 | 1 | 0 | 2 | 1 | 0 | 0 | 1 | 1 | 0 | 0 | 1 | 1 | 0 | 0 |
| | (2-2) | (1-1) | (1-1) | (0-0) | (2-2) | (1-1) | (0-0) | (0-0) | (1-1) | (1-1) | (0-0) | (0-0) | (1-1) | (1-1) | (0-0) | (0-0) |
| Burkina Faso | 81 | 90 | 74 | 54 | 43 | 48 | 42 | 32 | 19 | 22 | 21 | 18 | 62 | 68 | 53 | 36 |
| | (75-87) | (84-97) | (61-90) | (34-86) | (40-46) | (45-52) | (35-51) | (20-50) | (17-22) | (19-25) | (15-28) | (10-30) | (57-66) | (63-74) | (42-65) | (22-58) |
| Burundi | 43 | 40 | 31 | 22 | 24 | 24 | 19 | 14 | 10 | 10 | 11 | 9 | 33 | 30 | 20 | 13 |
| | (38-48) | (37-45) | (23-40) | (12-37) | (21-26) | (22-26) | (15-25) | (8-24) | (9-12) | (9-11) | (8-15) | (5-16) | (29-37) | (27-34) | (15-26) | (7-22) |
| Cabo Verde | 1 | 0 | 0 | 0 | 1 | 0 | 0 | 0 | 0 | 0 | 0 | 0 | 1 | 0 | 0 | 0 |
| | (1-1) | (0-0) | (0-0) | (0-0) | (1-1) | (0-0) | (0-0) | (0-0) | (0-0) | (0-0) | (0-0) | (0-0) | (1-1) | (0-0) | (0-0) | (0-0) |
| Cambodia | 38 | 37 | 11 | 7 | 29 | 30 | 10 | 6 | 14 | 12 | 6 | 3 | 25 | 25 | 5 | 3 |
| | (35-42) | (34-41) | (8-14) | (3-14) | (27-32) | (27-33) | (7-13) | (3-12) | (12-15) | (11-14) | (4-8) | (2-7) | (22-27) | (22-27) | (4-7) | (1-7) |
| Cameroon | 65 | 84 | 73 | 62 | 39 | 49 | 45 | 39 | 20 | 22 | 25 | 24 | 45 | 62 | 47 | 37 |
| | (60-70) | (77-91) | (65-81) | (44-85) | (36-42) | (46-54) | (40-50) | (27-53) | (18-22) | (19-24) | (22-29) | (17-35) | (41-49) | (57-68) | (42-54) | (26-53) |
| Canada | 3 | 2 | 2 | 2 | 3 | 2 | 2 | 2 | 2 | 1 | 1 | 1 | 1 | 1 | 1 | 1 |
| | (3-3) | (2-2) | (2-2) | (2-2) | (3-3) | (2-2) | (2-2) | (2-2) | (2-2) | (1-1) | (1-1) | (1-1) | (1-2) | (1-1) | (1-1) | (1-1) |
| Central African Republic | 23 | 26 | 23 | 21 | 14 | 16 | 14 | 14 | 7 | 7 | 7 | 7 | 16 | 19 | 16 | 13 |
| | (21-26) | (23-28) | (20-27) | (15-28) | (13-16) | (14-17) | (12-17) | (10-19) | (6-8) | (6-8) | (5-8) | (5-11) | (14-18) | (17-21) | (14-19) | (9-18) |
| Chad | 61 | 75 | 84 | 79 | 35 | 43 | 48 | 47 | 16 | 19 | 24 | 27 | 45 | 56 | 60 | 52 |
| | (57-66) | (70-82) | (70-99) | (51-120) | (33-38) | (40-47) | (40-56) | (31-72) | (14-18) | (17-22) | (19-30) | (16-43) | (41-49) | (51-62) | (49-71) | (33-81) |
| Chile | 6 | 3 | 2 | 1 | 5 | 2 | 2 | 1 | 3 | 1 | 1 | 1 | 3 | 1 | 1 | 0 |
| | (5-6) | (3-3) | (2-2) | (1-1) | (5-5) | (2-2) | (2-2) | (1-1) | (2-3) | (1-2) | (1-1) | (1-1) | (3-3) | (1-1) | (1-1) | (0-0) |
| China | 1,461 | 631 | 195 | 54 | 1,189 | 512 | 149 | 36 | 830 | 374 | 93 | 23 | 631 | 257 | 101 | 31 |
| | (1,351-1,583) | (597-670) | (184-207) | (49-60) | (1,100-1,288) | (485-544) | (140-158) | (33-40) | (741-928) | (345-406) | (85-102) | (20-26) | (549-720) | (232-284) | (93-110) | (27-35) |
| Colombia | 31 | 22 | 11 | 8 | 26 | 19 | 10 | 7 | 16 | 12 | 6 | 4 | 15 | 10 | 5 | 4 |
| | (28-33) | (20-24) | (9-14) | (5-13) | (24-28) | (17-20) | (8-12) | (5-12) | (14-17) | (11-13) | (5-8) | (3-7) | (13-16) | (9-11) | (4-6) | (2-6) |
| Comoros | 2 | 1 | 1 | 1 | 2 | 1 | 1 | 1 | 1 | 1 | 1 | 1 | 1 | 1 | 1 | 0 |
| | (2-3) | (1-2) | (1-1) | (1-1) | (2-2) | (1-1) | (1-1) | (1-1) | (1-1) | (1-1) | (0-1) | (0-1) | (1-2) | (1-1) | (0-1) | (0-1) |
| Congo | 8 | 12 | 9 | 7 | 5 | 8 | 6 | 5 | 3 | 4 | 4 | 3 | 6 | 9 | 6 | 4 |
| | (7-9) | (11-14) | (6-13) | (4-14) | (5-6) | (7-9) | (4-9) | (3-10) | (2-3) | (3-4) | (2-5) | (2-6) | (5-6) | (8-10) | (4-8) | (2-8) |
| Cook Islands | 0 | 0 | 0 | 0 | 0 | 0 | 0 | 0 | 0 | 0 | 0 | 0 | 0 | 0 | 0 | 0 |
| | (0-0) | (0-0) | (0-0) | (0-0) | (0-0) | (0-0) | (0-0) | (0-0) | (0-0) | (0-0) | (0-0) | (0-0) | (0-0) | (0-0) | (0-0) | (0-0) |
| Costa Rica | 1 | 1 | 1 | 1 | 1 | 1 | 1 | 0 | 1 | 1 | 0 | 0 | 1 | 0 | 0 | 0 |
| | (1-1) | (1-1) | (1-1) | (1-1) | (1-1) | (1-1) | (1-1) | (0-0) | (1-1) | (1-1) | (0-0) | (0-0) | (1-1) | (0-0) | (0-0) | (0-0) |
| Croatia | 1 | 0 | 0 | 0 | 1 | 0 | 0 | 0 | 0 | 0 | 0 | 0 | 0 | 0 | 0 | 0 |
| | (1-1) | (0-0) | (0-0) | (0-0) | (1-1) | (0-0) | (0-0) | (0-0) | (0-0) | (0-0) | (0-0) | (0-0) | (0-0) | (0-0) | (0-0) | (0-0) |
| Cuba | 2 | 1 | 1 | 1 | 2 | 1 | 1 | 1 | 1 | 1 | 0 | 0 | 1 | 1 | 0 | 0 |
| | (2-3) | (1-1) | (1-1) | (1-1) | (2-2) | (1-1) | (1-1) | (1-1) | (1-1) | (1-1) | (0-0) | (0-0) | (1-1) | (1-1) | (0-1) | (0-0) |

| Country | Number of under-five deaths (thousands) | | | | Number of infant deaths (thousands) | | | | Number of neonatal deaths (thousands) | | | | Number of deaths age 1 to 59 months (thousands) | | | |
|---|---|---|---|---|---|---|---|---|---|---|---|---|---|---|---|---|
| | 1990 | 2000 | 2015 | 2024 | 1990 | 2000 | 2015 | 2024 | 1990 | 2000 | 2015 | 2024 | 1990 | 2000 | 2015 | 2024 |
| Cyprus | 0 | 0 | 0 | 0 | 0 | 0 | 0 | 0 | 0 | 0 | 0 | 0 | 0 | 0 | 0 | 0 |
| | (0-0) | (0-0) | (0-0) | (0-0) | (0-0) | (0-0) | (0-0) | (0-0) | (0-0) | (0-0) | (0-0) | (0-0) | (0-0) | (0-0) | (0-0) | (0-0) |
| Czechia | 2 | 0 | 0 | 0 | 1 | 0 | 0 | 0 | 1 | 0 | 0 | 0 | 1 | 0 | 0 | 0 |
| | (2-2) | (0-1) | (0-0) | (0-0) | (1-1) | (0-0) | (0-0) | (0-0) | (1-1) | (0-0) | (0-0) | (0-0) | (1-1) | (0-0) | (0-0) | (0-0) |
| Côte d'Ivoire | 83 | 104 | 82 | 64 | 55 | 70 | 57 | 45 | 27 | 32 | 32 | 28 | 56 | 72 | 51 | 36 |
| | (77-89) | (97-113) | (74-91) | (50-81) | (51-60) | (65-75) | (51-63) | (35-56) | (24-30) | (28-36) | (28-36) | (21-36) | (51-60) | (67-79) | (45-57) | (28-47) |
| Democratic People's Republic of Korea | 17 | 43 | 7 | 6 | 14 | 25 | 6 | 5 | 9 | 10 | 4 | 3 | 8 | 33 | 3 | 3 |
| | (13-22) | (34-55) | (6-9) | (4-7) | (11-17) | (19-32) | (5-8) | (4-6) | (6-13) | (6-14) | (2-6) | (2-5) | (5-11) | (25-43) | (2-5) | (1-4) |
| Democratic Republic of the Congo | 286 | 400 | 307 | 385 | 161 | 232 | 189 | 247 | 65 | 78 | 84 | 105 | 221 | 322 | 223 | 280 |
| | (258-318) | (363-440) | (267-351) | (302-502) | (145-179) | (211-256) | (164-216) | (194-321) | (55-77) | (68-90) | (70-100) | (77-145) | (198-246) | (291-356) | (193-257) | (217-367) |
| Denmark | 1 | 0 | 0 | 0 | 0 | 0 | 0 | 0 | 0 | 0 | 0 | 0 | 0 | 0 | 0 | 0 |
| | (1-1) | (0-0) | (0-0) | (0-0) | (0-0) | (0-0) | (0-0) | (0-0) | (0-0) | (0-0) | (0-0) | (0-0) | (0-0) | (0-0) | (0-0) | (0-0) |
| Djibouti | 2 | 2 | 2 | 1 | 2 | 2 | 1 | 1 | 1 | 1 | 1 | 1 | 1 | 1 | 1 | 1 |
| | (2-3) | (2-3) | (1-2) | (1-2) | (2-2) | (2-2) | (1-2) | (1-2) | (1-1) | (1-1) | (1-1) | (0-1) | (1-1) | (1-2) | (0-1) | (0-1) |
| Dominica | 0 | 0 | 0 | 0 | 0 | 0 | 0 | 0 | 0 | 0 | 0 | 0 | 0 | 0 | 0 | 0 |
| | (0-0) | (0-0) | (0-0) | (0-0) | (0-0) | (0-0) | (0-0) | (0-0) | (0-0) | (0-0) | (0-0) | (0-0) | (0-0) | (0-0) | (0-0) | (0-0) |
| Dominican Republic | 13 | 9 | 7 | 6 | 10 | 7 | 7 | 6 | 5 | 5 | 5 | 4 | 7 | 4 | 2 | 2 |
| | (12-14) | (8-9) | (6-9) | (4-9) | (10-11) | (7-8) | (6-8) | (4-8) | (5-6) | (5-6) | (4-6) | (3-6) | (7-8) | (3-4) | (2-3) | (1-3) |
| Ecuador | 17 | 10 | 5 | 3 | 13 | 8 | 4 | 3 | 7 | 5 | 2 | 2 | 10 | 5 | 2 | 2 |
| | (15-18) | (9-11) | (4-5) | (3-4) | (12-15) | (7-9) | (4-4) | (3-3) | (6-8) | (4-5) | (2-2) | (2-2) | (8-11) | (4-6) | (2-3) | (1-2) |
| Egypt | 167 | 93 | 73 | 54 | 132 | 80 | 67 | 50 | 65 | 46 | 43 | 34 | 102 | 48 | 30 | 20 |
| | (159-176) | (87-100) | (65-83) | (39-74) | (125-139) | (74-85) | (59-76) | (37-69) | (60-71) | (42-50) | (37-49) | (24-47) | (95-109) | (44-52) | (26-35) | (14-29) |
| El Salvador | 11 | 5 | 2 | 1 | 9 | 4 | 1 | 1 | 4 | 2 | 1 | 0 | 7 | 3 | 1 | 1 |
| | (10-12) | (5-6) | (1-2) | (1-2) | (8-9) | (4-5) | (1-2) | (1-2) | (4-5) | (2-3) | (1-1) | (0-1) | (6-7) | (3-3) | (1-1) | (0-1) |
| Equatorial Guinea | 4 | 4 | 4 | 4 | 2 | 3 | 3 | 3 | 1 | 1 | 2 | 1 | 3 | 3 | 3 | 2 |
| | (3-4) | (4-5) | (3-6) | (2-7) | (2-3) | (3-3) | (2-4) | (1-5) | (1-1) | (1-2) | (1-3) | (1-3) | (2-3) | (2-3) | (2-4) | (1-4) |
| Eritrea | 13 | 7 | 4 | 3 | 7 | 4 | 3 | 2 | 3 | 2 | 2 | 2 | 10 | 5 | 3 | 2 |
| | (12-14) | (7-8) | (3-6) | (2-6) | (6-8) | (4-5) | (2-4) | (1-5) | (3-4) | (2-3) | (1-3) | (1-3) | (9-11) | (5-6) | (2-4) | (1-3) |
| Estonia | 0 | 0 | 0 | 0 | 0 | 0 | 0 | 0 | 0 | 0 | 0 | 0 | 0 | 0 | 0 | 0 |
| | (0-0) | (0-0) | (0-0) | (0-0) | (0-0) | (0-0) | (0-0) | (0-0) | (0-0) | (0-0) | (0-0) | (0-0) | (0-0) | (0-0) | (0-0) | (0-0) |
| Eswatini | 2 | 4 | 2 | 1 | 2 | 2 | 2 | 1 | 1 | 1 | 1 | 1 | 2 | 3 | 1 | 1 |
| | (2-3) | (3-4) | (2-2) | (1-2) | (2-2) | (2-3) | (1-2) | (1-2) | (1-1) | (1-1) | (1-1) | (0-1) | (1-2) | (3-3) | (1-1) | (0-1) |
| Ethiopia | 456 | 416 | 217 | 181 | 296 | 277 | 163 | 142 | 144 | 148 | 112 | 105 | 312 | 268 | 105 | 76 |
| | (424-490) | (388-447) | (192-245) | (128-258) | (275-318) | (259-298) | (144-183) | (100-201) | (129-159) | (133-163) | (97-128) | (73-151) | (288-338) | (248-291) | (92-120) | (53-111) |
| Fiji | 1 | 0 | 0 | 0 | 0 | 0 | 0 | 0 | 0 | 0 | 0 | 0 | 0 | 0 | 0 | 0 |

| | Number of under-five deaths (thousands) | | | | Number of infant deaths (thousands) | | | | Number of neonatal deaths (thousands) | | | | Number of deaths age 1 to 59 months (thousands) | | | |
|---|---|---|---|---|---|---|---|---|---|---|---|---|---|---|---|---|
| Country | 1990 | 2000 | 2015 | 2024 | 1990 | 2000 | 2015 | 2024 | 1990 | 2000 | 2015 | 2024 | 1990 | 2000 | 2015 | 2024 |
| | (1-1) | (0-0) | (0-0) | (0-1) | (0-1) | (0-0) | (0-0) | (0-0) | (0-0) | (0-0) | (0-0) | (0-0) | (0-0) | (0-0) | (0-0) | (0-0) |
| Finland | 0 | 0 | 0 | 0 | 0 | 0 | 0 | 0 | 0 | 0 | 0 | 0 | 0 | 0 | 0 | 0 |
| | (0-0) | (0-0) | (0-0) | (0-0) | (0-0) | (0-0) | (0-0) | (0-0) | (0-0) | (0-0) | (0-0) | (0-0) | (0-0) | (0-0) | (0-0) | (0-0) |
| France | 7 | 4 | 3 | 3 | 6 | 3 | 2 | 2 | 3 | 2 | 2 | 2 | 4 | 2 | 1 | 1 |
| | (7-7) | (4-4) | (3-3) | (3-3) | (6-6) | (3-3) | (2-3) | (2-2) | (3-3) | (2-2) | (2-2) | (2-2) | (4-4) | (2-2) | (1-1) | (1-1) |
| Gabon | 3 | 3 | 3 | 2 | 2 | 2 | 2 | 2 | 1 | 1 | 1 | 1 | 2 | 2 | 1 | 1 |
| | (3-3) | (3-3) | (2-3) | (2-3) | (2-2) | (2-2) | (2-3) | (1-3) | (1-1) | (1-1) | (1-2) | (1-2) | (2-2) | (2-2) | (1-2) | (1-2) |
| Gambia | 8 | 7 | 5 | 3 | 5 | 4 | 3 | 3 | 2 | 2 | 2 | 2 | 5 | 4 | 2 | 2 |
| | (7-9) | (6-7) | (4-5) | (2-5) | (4-5) | (4-5) | (3-4) | (2-4) | (2-3) | (2-3) | (2-3) | (1-3) | (5-6) | (4-5) | (2-3) | (1-2) |
| Georgia | 5 | 2 | 1 | 0 | 4 | 2 | 1 | 0 | 2 | 1 | 0 | 0 | 3 | 1 | 0 | 0 |
| | (4-5) | (2-2) | (1-1) | (0-0) | (4-4) | (1-2) | (1-1) | (0-0) | (2-3) | (1-1) | (0-0) | (0-0) | (2-3) | (1-1) | (0-0) | (0-0) |
| Germany | 8 | 4 | 3 | 3 | 6 | 3 | 2 | 2 | 3 | 2 | 2 | 2 | 5 | 2 | 1 | 1 |
| | (7-8) | (4-4) | (3-3) | (3-3) | (6-6) | (3-3) | (2-2) | (2-2) | (3-3) | (2-2) | (2-2) | (2-2) | (4-5) | (2-2) | (1-1) | (1-1) |
| Ghana | 77 | 67 | 46 | 32 | 44 | 42 | 33 | 24 | 26 | 25 | 22 | 16 | 51 | 42 | 24 | 16 |
| | (73-82) | (63-71) | (42-50) | (24-41) | (42-47) | (40-45) | (31-37) | (19-32) | (24-29) | (23-27) | (19-24) | (12-21) | (48-55) | (39-45) | (22-27) | (12-21) |
| Greece | 1 | 1 | 0 | 0 | 1 | 1 | 0 | 0 | 1 | 0 | 0 | 0 | 0 | 0 | 0 | 0 |
| | (1-1) | (1-1) | (0-0) | (0-0) | (1-1) | (1-1) | (0-0) | (0-0) | (1-1) | (0-0) | (0-0) | (0-0) | (0-0) | (0-0) | (0-0) | (0-0) |
| Grenada | 0 | 0 | 0 | 0 | 0 | 0 | 0 | 0 | 0 | 0 | 0 | 0 | 0 | 0 | 0 | 0 |
| | (0-0) | (0-0) | (0-0) | (0-0) | (0-0) | (0-0) | (0-0) | (0-0) | (0-0) | (0-0) | (0-0) | (0-0) | (0-0) | (0-0) | (0-0) | (0-0) |
| Guatemala | 28 | 21 | 12 | 8 | 21 | 16 | 10 | 6 | 10 | 9 | 5 | 4 | 18 | 12 | 6 | 4 |
| | (26-30) | (19-22) | (10-14) | (5-12) | (20-23) | (15-18) | (8-11) | (4-10) | (9-11) | (8-10) | (4-7) | (2-6) | (16-19) | (11-13) | (5-7) | (3-6) |
| Guinea | 65 | 57 | 49 | 44 | 38 | 34 | 31 | 29 | 18 | 16 | 15 | 15 | 47 | 41 | 34 | 30 |
| | (60-70) | (53-61) | (44-55) | (33-60) | (35-41) | (31-36) | (28-35) | (22-39) | (16-20) | (14-18) | (13-18) | (10-22) | (43-51) | (37-44) | (30-38) | (21-41) |
| Guinea-Bissau | 10 | 9 | 6 | 4 | 5 | 5 | 3 | 3 | 3 | 3 | 2 | 2 | 7 | 6 | 3 | 2 |
| | (9-11) | (8-10) | (4-8) | (2-8) | (5-6) | (5-6) | (2-5) | (1-5) | (2-3) | (2-3) | (2-3) | (1-4) | (6-8) | (5-7) | (2-4) | (1-4) |
| Guyana | 1 | 1 | 1 | 0 | 1 | 1 | 1 | 0 | 1 | 1 | 0 | 0 | 1 | 0 | 0 | 0 |
| | (1-2) | (1-1) | (0-1) | (0-1) | (1-1) | (1-1) | (0-1) | (0-1) | (1-1) | (0-1) | (0-1) | (0-0) | (1-1) | (0-0) | (0-0) | (0-0) |
| Haiti | 37 | 27 | 18 | 14 | 25 | 19 | 13 | 10 | 10 | 8 | 7 | 6 | 27 | 19 | 11 | 8 |
| | (34-40) | (25-29) | (15-22) | (9-21) | (23-27) | (17-20) | (11-16) | (6-16) | (9-12) | (7-9) | (6-9) | (3-10) | (24-29) | (17-21) | (9-14) | (5-12) |
| Honduras | 11 | 8 | 4 | 3 | 8 | 7 | 4 | 3 | 4 | 4 | 3 | 2 | 7 | 4 | 2 | 2 |
| | (10-12) | (8-9) | (4-5) | (2-5) | (8-9) | (6-7) | (3-4) | (2-4) | (4-5) | (4-5) | (2-3) | (1-3) | (6-7) | (4-5) | (2-2) | (1-2) |
| Hungary | 2 | 1 | 0 | 0 | 2 | 1 | 0 | 0 | 1 | 1 | 0 | 0 | 1 | 0 | 0 | 0 |
| | (2-2) | (1-1) | (0-0) | (0-0) | (2-2) | (1-1) | (0-0) | (0-0) | (1-1) | (1-1) | (0-0) | (0-0) | (1-1) | (0-0) | (0-0) | (0-0) |
| Iceland | 0 | 0 | 0 | 0 | 0 | 0 | 0 | 0 | 0 | 0 | 0 | 0 | 0 | 0 | 0 | 0 |
| | (0-0) | (0-0) | (0-0) | (0-0) | (0-0) | (0-0) | (0-0) | (0-0) | (0-0) | (0-0) | (0-0) | (0-0) | (0-0) | (0-0) | (0-0) | (0-0) |
| India | 3,523 | 2,624 | 1,100 | 615 | 2,343 | 1,898 | 906 | 540 | 1,605 | 1,275 | 654 | 386 | 1,918 | 1,349 | 446 | 229 |
| | (3,399-3,647) | (2,526-2,726) | (1,048-1,152) | (557-680) | (2,261-2,425) | (1,828-1,972) | (863-949) | (489-596) | (1,517-1,700) | (1,200-1,351) | (615-694) | (346-431) | (1,820-2,016) | (1,274-1,429) | (415-477) | (201-259) |

| | Number of under-five deaths (thousands) | | | | Number of infant deaths (thousands) | | | | Number of neonatal deaths (thousands) | | | | Number of deaths age 1 to 59 months (thousands) | | | |
|---|---|---|---|---|---|---|---|---|---|---|---|---|---|---|---|---|
| Country | 1990 | 2000 | 2015 | 2024 | 1990 | 2000 | 2015 | 2024 | 1990 | 2000 | 2015 | 2024 | 1990 | 2000 | 2015 | 2024 |
| Indonesia | 393 | 241 | 128 | 79 | 304 | 194 | 107 | 68 | 143 | 105 | 65 | 41 | 251 | 136 | 63 | 38 |
| | (374-414) | (229-255) | (118-141) | (65-97) | (289-320) | (183-204) | (98-118) | (56-83) | (132-153) | (97-113) | (59-73) | (33-52) | (237-266) | (128-146) | (57-70) | (31-48) |
| Iran (Islamic Republic of) | 114 | 38 | 24 | 13 | 96 | 33 | 22 | 12 | 48 | 21 | 16 | 8 | 66 | 18 | 8 | 5 |
| | (104-125) | (35-43) | (17-33) | (7-24) | (88-105) | (30-37) | (15-30) | (6-22) | (32-64) | (17-25) | (10-24) | (3-17) | (51-83) | (14-22) | (3-14) | (1-11) |
| Iraq | 55 | 38 | 35 | 25 | 39 | 33 | 32 | 23 | 19 | 21 | 20 | 15 | 36 | 17 | 15 | 11 |
| | (50-61) | (35-42) | (29-42) | (17-37) | (35-42) | (30-37) | (27-38) | (16-34) | (17-21) | (19-24) | (16-24) | (10-22) | (33-40) | (15-19) | (12-18) | (7-16) |
| Ireland | 0 | 0 | 0 | 0 | 0 | 0 | 0 | 0 | 0 | 0 | 0 | 0 | 0 | 0 | 0 | 0 |
| | (0-1) | (0-0) | (0-0) | (0-0) | (0-0) | (0-0) | (0-0) | (0-0) | (0-0) | (0-0) | (0-0) | (0-0) | (0-0) | (0-0) | (0-0) | (0-0) |
| Israel | 1 | 1 | 1 | 1 | 1 | 1 | 1 | 0 | 1 | 0 | 0 | 0 | 0 | 0 | 0 | 0 |
| | (1-1) | (1-1) | (1-1) | (1-1) | (1-1) | (1-1) | (1-1) | (0-0) | (1-1) | (0-0) | (0-0) | (0-0) | (0-1) | (0-0) | (0-0) | (0-0) |
| Italy | 5 | 3 | 2 | 1 | 5 | 3 | 1 | 1 | 4 | 2 | 1 | 1 | 2 | 1 | 1 | 0 |
| | (5-6) | (3-3) | (2-2) | (1-1) | (5-5) | (2-3) | (1-1) | (1-1) | (4-4) | (2-2) | (1-1) | (1-1) | (2-2) | (1-1) | (1-1) | (0-0) |
| Jamaica | 2 | 1 | 1 | 1 | 2 | 1 | 1 | 1 | 1 | 1 | 1 | 0 | 1 | 0 | 0 | 0 |
| | (1-2) | (1-1) | (1-1) | (1-1) | (1-2) | (1-1) | (1-1) | (0-1) | (1-1) | (1-1) | (1-1) | (0-0) | (0-1) | (0-0) | (0-0) | (0-0) |
| Japan | 8 | 5 | 3 | 2 | 5 | 4 | 2 | 1 | 3 | 2 | 1 | 1 | 5 | 3 | 2 | 1 |
| | (8-8) | (5-5) | (3-3) | (2-2) | (5-6) | (4-4) | (2-2) | (1-1) | (3-3) | (2-2) | (1-1) | (1-1) | (5-5) | (3-3) | (2-2) | (1-1) |
| Jordan | 5 | 4 | 4 | 3 | 4 | 4 | 3 | 3 | 3 | 2 | 2 | 2 | 2 | 2 | 1 | 1 |
| | (4-5) | (4-4) | (3-4) | (2-4) | (4-4) | (3-4) | (3-4) | (2-4) | (2-3) | (2-3) | (2-3) | (1-3) | (2-2) | (1-2) | (1-2) | (1-2) |
| Kazakhstan | 21 | 10 | 5 | 4 | 18 | 9 | 4 | 3 | 9 | 6 | 2 | 2 | 12 | 5 | 3 | 2 |
| | (19-23) | (10-11) | (5-5) | (4-4) | (16-19) | (8-10) | (4-4) | (3-3) | (8-10) | (5-7) | (2-2) | (1-2) | (10-13) | (4-5) | (2-3) | (2-2) |
| Kenya | 99 | 112 | 68 | 58 | 67 | 77 | 56 | 51 | 27 | 32 | 34 | 31 | 72 | 80 | 34 | 27 |
| | (92-106) | (105-121) | (62-75) | (47-71) | (62-71) | (71-83) | (51-62) | (42-63) | (24-30) | (29-36) | (30-38) | (25-40) | (67-78) | (74-86) | (30-38) | (21-34) |
| Kiribati | 0 | 0 | 0 | 0 | 0 | 0 | 0 | 0 | 0 | 0 | 0 | 0 | 0 | 0 | 0 | 0 |
| | (0-0) | (0-0) | (0-0) | (0-0) | (0-0) | (0-0) | (0-0) | (0-0) | (0-0) | (0-0) | (0-0) | (0-0) | (0-0) | (0-0) | (0-0) | (0-0) |
| Kosovo (UNSCR 1244) | 6 | 2 | 0 | 0 | 5 | 2 | 0 | 0 | 2 | 1 | 0 | 0 | 3 | 1 | 0 | 0 |
| | (5-8) | (1-2) | (0-0) | (0-0) | (4-7) | (1-2) | (0-0) | (0-0) | (2-3) | (1-1) | (0-0) | (0-0) | (3-5) | (1-1) | (0-0) | (0-0) |
| Kuwait | 1 | 1 | 1 | 0 | 1 | 0 | 0 | 0 | 1 | 0 | 0 | 0 | 0 | 0 | 0 | 0 |
| | (1-1) | (1-1) | (1-1) | (0-0) | (1-1) | (0-0) | (0-0) | (0-0) | (1-1) | (0-0) | (0-0) | (0-0) | (0-0) | (0-0) | (0-0) | (0-0) |
| Kyrgyzstan | 9 | 6 | 3 | 3 | 8 | 5 | 3 | 2 | 3 | 2 | 2 | 2 | 6 | 3 | 1 | 1 |
| | (8-10) | (5-6) | (3-4) | (2-3) | (7-9) | (4-5) | (3-3) | (2-2) | (3-4) | (2-3) | (2-2) | (1-2) | (5-7) | (3-4) | (1-2) | (1-1) |
| Lao People's Democratic Republic | 26 | 18 | 7 | 5 | 20 | 15 | 7 | 4 | 9 | 6 | 3 | 2 | 17 | 12 | 4 | 3 |
| | (24-29) | (16-20) | (6-9) | (4-6) | (18-22) | (13-16) | (6-8) | (3-6) | (8-10) | (6-7) | (3-4) | (1-3) | (16-19) | (10-13) | (4-5) | (2-4) |
| Latvia | 1 | 0 | 0 | 0 | 0 | 0 | 0 | 0 | 0 | 0 | 0 | 0 | 0 | 0 | 0 | 0 |
| | (1-1) | (0-0) | (0-0) | (0-0) | (0-1) | (0-0) | (0-0) | (0-0) | (0-0) | (0-0) | (0-0) | (0-0) | (0-0) | (0-0) | (0-0) | (0-0) |
| Lebanon | 3 | 2 | 2 | 2 | 3 | 2 | 1 | 1 | 2 | 1 | 1 | 1 | 1 | 1 | 1 | 1 |
| | (3-4) | (2-2) | (1-2) | (1-2) | (3-3) | (1-2) | (1-2) | (1-2) | (2-2) | (1-1) | (1-1) | (1-1) | (1-1) | (1-1) | (1-1) | (1-1) |

| Country | Number of under-five deaths (thousands) 1990 | 2000 | 2015 | 2024 | Number of infant deaths (thousands) 1990 | 2000 | 2015 | 2024 | Number of neonatal deaths (thousands) 1990 | 2000 | 2015 | 2024 | Number of deaths age 1 to 59 months (thousands) 1990 | 2000 | 2015 | 2024 |
|---|---|---|---|---|---|---|---|---|---|---|---|---|---|---|---|---|
| Lesotho | 5 | 6 | 5 | 3 | 5 | 4 | 4 | 3 | 2 | 2 | 2 | 2 | 3 | 4 | 2 | 2 |
| | (5-6) | (6-7) | (4-5) | (2-5) | (4-5) | (4-5) | (4-5) | (2-4) | (2-3) | (2-2) | (2-2) | (1-2) | (3-3) | (4-4) | (2-3) | (1-2) |
| Liberia | 31 | 22 | 15 | 15 | 19 | 15 | 10 | 11 | 7 | 6 | 5 | 6 | 23 | 16 | 9 | 9 |
| | (28-33) | (21-24) | (13-17) | (11-18) | (18-21) | (14-17) | (9-12) | (8-13) | (6-8) | (6-7) | (5-6) | (4-8) | (21-26) | (15-18) | (8-11) | (7-11) |
| Libya | 5 | 3 | 2 | 1 | 4 | 2 | 2 | 1 | 3 | 2 | 1 | 1 | 3 | 1 | 1 | 1 |
| | (4-6) | (3-3) | (1-3) | (1-2) | (3-5) | (2-3) | (1-2) | (1-2) | (2-3) | (1-2) | (1-1) | (0-1) | (2-3) | (1-2) | (1-1) | (0-1) |
| Lithuania | 1 | 0 | 0 | 0 | 1 | 0 | 0 | 0 | 0 | 0 | 0 | 0 | 0 | 0 | 0 | 0 |
| | (1-1) | (0-0) | (0-0) | (0-0) | (1-1) | (0-0) | (0-0) | (0-0) | (0-0) | (0-0) | (0-0) | (0-0) | (0-0) | (0-0) | (0-0) | (0-0) |
| Luxembourg | 0 | 0 | 0 | 0 | 0 | 0 | 0 | 0 | 0 | 0 | 0 | 0 | 0 | 0 | 0 | 0 |
| | (0-0) | (0-0) | (0-0) | (0-0) | (0-0) | (0-0) | (0-0) | (0-0) | (0-0) | (0-0) | (0-0) | (0-0) | (0-0) | (0-0) | (0-0) | (0-0) |
| Madagascar | 78 | 70 | 55 | 62 | 47 | 45 | 37 | 43 | 20 | 21 | 20 | 23 | 57 | 49 | 34 | 39 |
| | (72-83) | (65-76) | (49-61) | (48-81) | (44-50) | (42-48) | (33-41) | (33-56) | (18-23) | (19-24) | (18-23) | (18-32) | (53-62) | (45-53) | (30-39) | (29-51) |
| Malawi | 104 | 83 | 35 | 32 | 59 | 49 | 24 | 25 | 23 | 19 | 15 | 16 | 81 | 64 | 20 | 16 |
| | (98-111) | (78-89) | (31-38) | (24-43) | (56-63) | (46-52) | (21-27) | (19-34) | (21-26) | (17-21) | (13-16) | (12-22) | (76-87) | (60-69) | (18-22) | (12-22) |
| Malaysia | 8 | 5 | 4 | 4 | 6 | 4 | 3 | 3 | 4 | 3 | 2 | 2 | 4 | 3 | 2 | 2 |
| | (7-8) | (5-5) | (4-4) | (3-4) | (6-6) | (4-4) | (3-3) | (3-3) | (3-4) | (2-3) | (2-2) | (2-2) | (4-5) | (2-3) | (2-2) | (2-2) |
| Maldives | 1 | 0 | 0 | 0 | 1 | 0 | 0 | 0 | 0 | 0 | 0 | 0 | 0 | 0 | 0 | 0 |
| | (1-1) | (0-0) | (0-0) | (0-0) | (1-1) | (0-0) | (0-0) | (0-0) | (0-0) | (0-0) | (0-0) | (0-0) | (0-0) | (0-0) | (0-0) | (0-0) |
| Mali | 98 | 97 | 87 | 68 | 53 | 54 | 54 | 45 | 29 | 28 | 29 | 27 | 69 | 69 | 58 | 41 |
| | (92-104) | (91-103) | (80-95) | (57-80) | (49-56) | (51-58) | (49-58) | (38-53) | (26-32) | (25-31) | (26-33) | (22-34) | (64-74) | (64-74) | (53-64) | (33-50) |
| Malta | 0 | 0 | 0 | 0 | 0 | 0 | 0 | 0 | 0 | 0 | 0 | 0 | 0 | 0 | 0 | 0 |
| | (0-0) | (0-0) | (0-0) | (0-0) | (0-0) | (0-0) | (0-0) | (0-0) | (0-0) | (0-0) | (0-0) | (0-0) | (0-0) | (0-0) | (0-0) | (0-0) |
| Marshall Islands | 0 | 0 | 0 | 0 | 0 | 0 | 0 | 0 | 0 | 0 | 0 | 0 | 0 | 0 | 0 | 0 |
| | (0-0) | (0-0) | (0-0) | (0-0) | (0-0) | (0-0) | (0-0) | (0-0) | (0-0) | (0-0) | (0-0) | (0-0) | (0-0) | (0-0) | (0-0) | (0-0) |
| Mauritania | 9 | 9 | 7 | 6 | 6 | 7 | 6 | 5 | 4 | 4 | 4 | 4 | 6 | 6 | 3 | 3 |
| | (8-10) | (9-10) | (6-8) | (4-9) | (5-6) | (6-7) | (5-6) | (4-8) | (3-4) | (3-4) | (3-4) | (3-5) | (5-6) | (5-6) | (3-4) | (2-4) |
| Mauritius | 1 | 0 | 0 | 0 | 0 | 0 | 0 | 0 | 0 | 0 | 0 | 0 | 0 | 0 | 0 | 0 |
| | (0-1) | (0-0) | (0-0) | (0-0) | (0-0) | (0-0) | (0-0) | (0-0) | (0-0) | (0-0) | (0-0) | (0-0) | (0-0) | (0-0) | (0-0) | (0-0) |
| Mexico | 108 | 65 | 37 | 27 | 92 | 57 | 34 | 24 | 54 | 33 | 19 | 17 | 55 | 32 | 18 | 10 |
| | (101-117) | (61-70) | (35-39) | (22-32) | (86-99) | (53-61) | (32-35) | (20-30) | (47-60) | (30-37) | (17-20) | (12-22) | (49-62) | (29-36) | (17-20) | (6-14) |
| Micronesia (Federated States of) | 0 | 0 | 0 | 0 | 0 | 0 | 0 | 0 | 0 | 0 | 0 | 0 | 0 | 0 | 0 | 0 |
| | (0-0) | (0-0) | (0-0) | (0-0) | (0-0) | (0-0) | (0-0) | (0-0) | (0-0) | (0-0) | (0-0) | (0-0) | (0-0) | (0-0) | (0-0) | (0-0) |
| Monaco | 0 | 0 | 0 | 0 | 0 | 0 | 0 | 0 | 0 | 0 | 0 | 0 | 0 | 0 | 0 | 0 |
| | (0-0) | (0-0) | (0-0) | (0-0) | (0-0) | (0-0) | (0-0) | (0-0) | (0-0) | (0-0) | (0-0) | (0-0) | (0-0) | (0-0) | (0-0) | (0-0) |
| Mongolia | 8 | 3 | 1 | 1 | 5 | 2 | 1 | 1 | 2 | 1 | 1 | 0 | 6 | 2 | 1 | 0 |
| | (7-8) | (3-3) | (1-2) | (1-1) | (5-6) | (2-3) | (1-1) | (1-1) | (2-2) | (1-1) | (1-1) | (0-0) | (5-6) | (2-2) | (1-1) | (0-0) |

| | Number of under-five deaths (thousands) | | | | Number of infant deaths (thousands) | | | | Number of neonatal deaths (thousands) | | | | Number of deaths age 1 to 59 months (thousands) | | | |
|---|---|---|---|---|---|---|---|---|---|---|---|---|---|---|---|---|
| Country | 1990 | 2000 | 2015 | 2024 | 1990 | 2000 | 2015 | 2024 | 1990 | 2000 | 2015 | 2024 | 1990 | 2000 | 2015 | 2024 |
| Montenegro | 0 | 0 | 0 | 0 | 0 | 0 | 0 | 0 | 0 | 0 | 0 | 0 | 0 | 0 | 0 | 0 |
| | (0-0) | (0-0) | (0-0) | (0-0) | (0-0) | (0-0) | (0-0) | (0-0) | (0-0) | (0-0) | (0-0) | (0-0) | (0-0) | (0-0) | (0-0) | (0-0) |
| Montserrat | 0 | 0 | 0 | 0 | 0 | 0 | 0 | 0 | 0 | 0 | 0 | 0 | 0 | 0 | 0 | 0 |
| | (0-0) | (0-0) | (0-0) | (0-0) | (0-0) | (0-0) | (0-0) | (0-0) | (0-0) | (0-0) | (0-0) | (0-0) | (0-0) | (0-0) | (0-0) | (0-0) |
| Morocco | 59 | 34 | 16 | 10 | 47 | 29 | 14 | 9 | 27 | 18 | 10 | 6 | 32 | 16 | 6 | 4 |
| | (54-63) | (31-38) | (12-19) | (6-15) | (44-51) | (27-32) | (12-18) | (6-14) | (24-30) | (16-21) | (8-12) | (4-9) | (29-35) | (14-18) | (5-8) | (2-6) |
| Mozambique | 144 | 125 | 79 | 74 | 89 | 82 | 57 | 55 | 37 | 35 | 28 | 32 | 106 | 90 | 51 | 42 |
| | (132-157) | (115-135) | (71-88) | (58-95) | (82-97) | (76-89) | (51-63) | (43-71) | (33-42) | (31-39) | (25-33) | (24-43) | (97-116) | (83-97) | (45-57) | (32-55) |
| Myanmar | 123 | 92 | 47 | 33 | 97 | 76 | 41 | 29 | 50 | 39 | 23 | 18 | 73 | 54 | 24 | 16 |
| | (112-135) | (85-101) | (37-60) | (20-54) | (88-106) | (70-84) | (32-52) | (18-48) | (43-60) | (34-44) | (17-30) | (10-30) | (63-82) | (48-60) | (18-31) | (9-26) |
| Namibia | 4 | 4 | 4 | 3 | 3 | 3 | 3 | 3 | 2 | 1 | 2 | 2 | 2 | 3 | 2 | 1 |
| | (3-4) | (4-5) | (3-4) | (2-4) | (3-3) | (2-3) | (3-4) | (2-4) | (1-2) | (1-2) | (1-2) | (1-3) | (2-2) | (3-3) | (1-2) | (1-2) |
| Nauru | 0 | 0 | 0 | 0 | 0 | 0 | 0 | 0 | 0 | 0 | 0 | 0 | 0 | 0 | 0 | 0 |
| | (0-0) | (0-0) | (0-0) | (0-0) | (0-0) | (0-0) | (0-0) | (0-0) | (0-0) | (0-0) | (0-0) | (0-0) | (0-0) | (0-0) | (0-0) | (0-0) |
| Nepal | 99 | 60 | 23 | 14 | 74 | 47 | 20 | 13 | 43 | 30 | 14 | 9 | 56 | 30 | 9 | 6 |
| | (93-106) | (56-64) | (21-26) | (11-18) | (69-79) | (44-50) | (18-22) | (10-16) | (39-47) | (28-33) | (13-16) | (7-11) | (52-61) | (27-32) | (8-10) | (4-7) |
| Netherlands (Kingdom of the) | 2 | 1 | 1 | 1 | 1 | 1 | 1 | 1 | 1 | 1 | 0 | 0 | 1 | 0 | 0 | 0 |
| | (2-2) | (1-1) | (1-1) | (1-1) | (1-1) | (1-1) | (1-1) | (1-1) | (1-1) | (1-1) | (0-0) | (0-0) | (1-1) | (0-0) | (0-0) | (0-0) |
| New Zealand | 1 | 0 | 0 | 0 | 1 | 0 | 0 | 0 | 0 | 0 | 0 | 0 | 0 | 0 | 0 | 0 |
| | (1-1) | (0-0) | (0-0) | (0-0) | (1-1) | (0-0) | (0-0) | (0-0) | (0-0) | (0-0) | (0-0) | (0-0) | (0-0) | (0-0) | (0-0) | (0-0) |
| Nicaragua | 10 | 5 | 2 | 1 | 9 | 4 | 2 | 1 | 4 | 2 | 1 | 1 | 7 | 3 | 1 | 1 |
| | (10-11) | (5-6) | (2-3) | (1-2) | (8-9) | (4-5) | (2-2) | (1-1) | (3-4) | (2-3) | (1-2) | (1-1) | (6-7) | (3-3) | (1-1) | (1-1) |
| Niger | 142 | 130 | 111 | 118 | 65 | 64 | 63 | 72 | 25 | 27 | 32 | 37 | 117 | 103 | 79 | 82 |
| | (133-153) | (121-141) | (99-125) | (89-158) | (61-70) | (60-70) | (56-71) | (54-96) | (22-29) | (24-31) | (27-38) | (26-52) | (109-126) | (95-113) | (69-90) | (60-110) |
| Nigeria | 855 | 924 | 859 | 856 | 440 | 509 | 499 | 520 | 214 | 255 | 282 | 295 | 641 | 669 | 577 | 561 |
| | (798-914) | (871-979) | (795-928) | (731-1,009) | (411-470) | (480-539) | (462-539) | (445-611) | (192-237) | (232-280) | (254-313) | (241-363) | (595-689) | (626-713) | (530-627) | (469-669) |
| Niue | 0 | 0 | 0 | 0 | 0 | 0 | 0 | 0 | 0 | 0 | 0 | 0 | 0 | 0 | 0 | 0 |
| | (0-0) | (0-0) | (0-0) | (0-0) | (0-0) | (0-0) | (0-0) | (0-0) | (0-0) | (0-0) | (0-0) | (0-0) | (0-0) | (0-0) | (0-0) | (0-0) |
| North Macedonia | 1 | 0 | 0 | 0 | 1 | 0 | 0 | 0 | 1 | 0 | 0 | 0 | 1 | 0 | 0 | 0 |
| | (1-1) | (0-0) | (0-0) | (0-0) | (1-1) | (0-0) | (0-0) | (0-0) | (1-1) | (0-0) | (0-0) | (0-0) | (1-1) | (0-0) | (0-0) | (0-0) |
| Norway | 1 | 0 | 0 | 0 | 0 | 0 | 0 | 0 | 0 | 0 | 0 | 0 | 0 | 0 | 0 | 0 |
| | (0-1) | (0-0) | (0-0) | (0-0) | (0-0) | (0-0) | (0-0) | (0-0) | (0-0) | (0-0) | (0-0) | (0-0) | (0-0) | (0-0) | (0-0) | (0-0) |
| Oman | 3 | 1 | 1 | 1 | 2 | 1 | 1 | 1 | 1 | 0 | 0 | 1 | 1 | 0 | 0 | 0 |
| | (2-3) | (1-1) | (1-1) | (1-1) | (2-2) | (1-1) | (1-1) | (1-1) | (1-1) | (0-1) | (0-1) | (0-1) | (1-2) | (0-1) | (0-0) | (0-0) |
| Pakistan | 673 | 596 | 496 | 382 | 546 | 492 | 420 | 329 | 324 | 323 | 299 | 249 | 349 | 273 | 197 | 133 |

| Country | Number of under-five deaths (thousands) | | | | Number of infant deaths (thousands) | | | | Number of neonatal deaths (thousands) | | | | Number of deaths age 1 to 59 months (thousands) | | | |
|---|---|---|---|---|---|---|---|---|---|---|---|---|---|---|---|---|
| | 1990 | 2000 | 2015 | 2024 | 1990 | 2000 | 2015 | 2024 | 1990 | 2000 | 2015 | 2024 | 1990 | 2000 | 2015 | 2024 |
| | (648-699) | (570-621) | (457-539) | (300-479) | (527-568) | (471-514) | (388-457) | (259-413) | (303-346) | (303-343) | (272-329) | (194-316) | (328-372) | (255-292) | (176-219) | (100-171) |
| Palau | 0 | 0 | 0 | 0 | 0 | 0 | 0 | 0 | 0 | 0 | 0 | 0 | 0 | 0 | 0 | 0 |
| | (0-0) | (0-0) | (0-0) | (0-0) | (0-0) | (0-0) | (0-0) | (0-0) | (0-0) | (0-0) | (0-0) | (0-0) | (0-0) | (0-0) | (0-0) | (0-0) |
| Panama | 2 | 2 | 1 | 1 | 2 | 1 | 1 | 1 | 1 | 1 | 1 | 0 | 1 | 1 | 1 | 1 |
| | (2-2) | (2-2) | (1-1) | (1-1) | (1-2) | (1-2) | (1-1) | (1-1) | (1-1) | (1-1) | (0-1) | (0-0) | (1-1) | (1-1) | (1-1) | (1-1) |
| Papua New Guinea | 12 | 13 | 14 | 10 | 9 | 10 | 11 | 8 | 5 | 6 | 6 | 5 | 7 | 7 | 8 | 5 |
| | (11-13) | (12-15) | (11-18) | (6-16) | (8-10) | (9-12) | (8-14) | (5-13) | (4-5) | (5-7) | (5-8) | (3-8) | (7-8) | (6-8) | (6-10) | (3-8) |
| Paraguay | 6 | 5 | 3 | 2 | 5 | 4 | 3 | 2 | 3 | 2 | 2 | 1 | 3 | 2 | 1 | 1 |
| | (6-7) | (4-6) | (2-5) | (1-5) | (5-6) | (3-5) | (2-5) | (1-4) | (3-4) | (2-3) | (1-3) | (1-3) | (3-4) | (2-3) | (1-3) | (0-2) |
| Peru | 55 | 24 | 9 | 7 | 41 | 19 | 8 | 6 | 19 | 10 | 4 | 4 | 35 | 14 | 5 | 3 |
| | (52-58) | (23-26) | (8-10) | (5-10) | (38-43) | (18-20) | (7-8) | (4-8) | (18-21) | (9-11) | (4-5) | (3-5) | (33-38) | (13-15) | (4-5) | (3-5) |
| Philippines | 118 | 85 | 68 | 49 | 83 | 63 | 53 | 40 | 41 | 39 | 33 | 25 | 77 | 46 | 35 | 24 |
| | (110-128) | (79-92) | (60-76) | (36-67) | (77-90) | (58-68) | (47-60) | (29-55) | (37-46) | (35-43) | (28-37) | (18-36) | (71-84) | (42-51) | (31-40) | (17-34) |
| Poland | 10 | 4 | 2 | 1 | 8 | 3 | 2 | 1 | 6 | 2 | 1 | 1 | 4 | 1 | 1 | 1 |
| | (10-10) | (3-4) | (2-2) | (1-1) | (8-9) | (3-3) | (2-2) | (1-1) | (6-6) | (2-2) | (1-1) | (1-1) | (4-4) | (1-1) | (1-1) | (1-1) |
| Portugal | 2 | 1 | 0 | 0 | 1 | 1 | 0 | 0 | 1 | 0 | 0 | 0 | 1 | 0 | 0 | 0 |
| | (2-2) | (1-1) | (0-0) | (0-0) | (1-1) | (1-1) | (0-0) | (0-0) | (1-1) | (0-0) | (0-0) | (0-0) | (1-1) | (0-0) | (0-0) | (0-0) |
| Qatar | 0 | 0 | 0 | 0 | 0 | 0 | 0 | 0 | 0 | 0 | 0 | 0 | 0 | 0 | 0 | 0 |
| | (0-0) | (0-0) | (0-0) | (0-0) | (0-0) | (0-0) | (0-0) | (0-0) | (0-0) | (0-0) | (0-0) | (0-0) | (0-0) | (0-0) | (0-0) | (0-0) |
| Republic of Korea | 11 | 5 | 2 | 1 | 8 | 4 | 1 | 1 | 5 | 2 | 1 | 0 | 5 | 3 | 1 | 0 |
| | (10-11) | (4-5) | (1-2) | (1-1) | (8-9) | (3-4) | (1-1) | (1-1) | (4-6) | (2-2) | (1-1) | (0-0) | (5-6) | (2-3) | (1-1) | (0-0) |
| Republic of Moldova | 3 | 2 | 1 | 1 | 2 | 1 | 1 | 0 | 2 | 1 | 1 | 0 | 1 | 1 | 0 | 0 |
| | (2-3) | (1-2) | (1-1) | (0-1) | (2-3) | (1-2) | (1-1) | (0-1) | (1-2) | (1-1) | (0-1) | (0-1) | (1-2) | (0-1) | (0-0) | (0-0) |
| Romania | 11 | 5 | 2 | 1 | 8 | 4 | 2 | 1 | 5 | 2 | 1 | 1 | 6 | 3 | 1 | 1 |
| | (10-11) | (5-5) | (2-2) | (1-1) | (8-8) | (4-4) | (2-2) | (1-1) | (4-6) | (2-3) | (1-1) | (1-1) | (5-7) | (2-3) | (1-1) | (1-1) |
| Russian Federation | 47 | 25 | 16 | 7 | 37 | 20 | 13 | 5 | 22 | 11 | 8 | 3 | 25 | 13 | 8 | 4 |
| | (46-48) | (24-25) | (16-16) | (6-7) | (36-37) | (19-20) | (13-13) | (5-6) | (16-28) | (10-13) | (7-9) | (2-3) | (19-31) | (12-14) | (7-8) | (4-5) |
| Rwanda | 48 | 59 | 17 | 15 | 28 | 37 | 12 | 12 | 13 | 15 | 7 | 7 | 35 | 44 | 10 | 8 |
| | (45-52) | (55-63) | (14-21) | (11-21) | (26-30) | (34-39) | (10-15) | (8-16) | (12-15) | (13-16) | (6-9) | (5-10) | (33-38) | (41-47) | (8-12) | (6-11) |
| Saint Kitts and Nevis | 0 | 0 | 0 | 0 | 0 | 0 | 0 | 0 | 0 | 0 | 0 | 0 | 0 | 0 | 0 | 0 |
| | (0-0) | (0-0) | (0-0) | (0-0) | (0-0) | (0-0) | (0-0) | (0-0) | (0-0) | (0-0) | (0-0) | (0-0) | (0-0) | (0-0) | (0-0) | (0-0) |
| Saint Lucia | 0 | 0 | 0 | 0 | 0 | 0 | 0 | 0 | 0 | 0 | 0 | 0 | 0 | 0 | 0 | 0 |
| | (0-0) | (0-0) | (0-0) | (0-0) | (0-0) | (0-0) | (0-0) | (0-0) | (0-0) | (0-0) | (0-0) | (0-0) | (0-0) | (0-0) | (0-0) | (0-0) |
| Saint Vincent and the Grenadines | 0 | 0 | 0 | 0 | 0 | 0 | 0 | 0 | 0 | 0 | 0 | 0 | 0 | 0 | 0 | 0 |
| | (0-0) | (0-0) | (0-0) | (0-0) | (0-0) | (0-0) | (0-0) | (0-0) | (0-0) | (0-0) | (0-0) | (0-0) | (0-0) | (0-0) | (0-0) | (0-0) |
| Samoa | 0 | 0 | 0 | 0 | 0 | 0 | 0 | 0 | 0 | 0 | 0 | 0 | 0 | 0 | 0 | 0 |

| Country | Number of under-five deaths (thousands) | | | | Number of infant deaths (thousands) | | | | Number of neonatal deaths (thousands) | | | | Number of deaths age 1 to 59 months (thousands) | | | |
|---|---|---|---|---|---|---|---|---|---|---|---|---|---|---|---|---|
| | 1990 | 2000 | 2015 | 2024 | 1990 | 2000 | 2015 | 2024 | 1990 | 2000 | 2015 | 2024 | 1990 | 2000 | 2015 | 2024 |
| | (0-0) | (0-0) | (0-0) | (0-0) | (0-0) | (0-0) | (0-0) | (0-0) | (0-0) | (0-0) | (0-0) | (0-0) | (0-0) | (0-0) | (0-0) | (0-0) |
| San Marino | 0 | 0 | 0 | 0 | 0 | 0 | 0 | 0 | 0 | 0 | 0 | 0 | 0 | 0 | 0 | 0 |
| | (0-0) | (0-0) | (0-0) | (0-0) | (0-0) | (0-0) | (0-0) | (0-0) | (0-0) | (0-0) | (0-0) | (0-0) | (0-0) | (0-0) | (0-0) | (0-0) |
| Sao Tome and Principe | 1 | 0 | 0 | 0 | 0 | 0 | 0 | 0 | 0 | 0 | 0 | 0 | 0 | 0 | 0 | 0 |
| | (0-1) | (0-1) | (0-0) | (0-0) | (0-0) | (0-0) | (0-0) | (0-0) | (0-0) | (0-0) | (0-0) | (0-0) | (0-0) | (0-0) | (0-0) | (0-0) |
| Saudi Arabia | 18 | 10 | 5 | 3 | 16 | 9 | 4 | 3 | 9 | 5 | 3 | 2 | 9 | 5 | 3 | 2 |
| | (15-21) | (9-11) | (4-6) | (2-5) | (14-19) | (8-10) | (4-5) | (2-4) | (6-12) | (4-6) | (2-3) | (1-3) | (6-11) | (3-6) | (2-3) | (1-3) |
| Senegal | 43 | 48 | 26 | 19 | 23 | 28 | 19 | 15 | 13 | 15 | 13 | 11 | 30 | 34 | 13 | 8 |
| | (40-45) | (45-51) | (24-28) | (15-25) | (22-24) | (27-30) | (17-20) | (12-20) | (12-14) | (13-16) | (12-14) | (9-15) | (28-32) | (31-36) | (12-15) | (6-11) |
| Serbia | 3 | 1 | 0 | 0 | 2 | 1 | 0 | 0 | 2 | 1 | 0 | 0 | 1 | 0 | 0 | 0 |
| | (3-3) | (1-1) | (0-0) | (0-0) | (2-2) | (1-1) | (0-0) | (0-0) | (1-2) | (1-1) | (0-0) | (0-0) | (1-1) | (0-0) | (0-0) | (0-0) |
| Seychelles | 0 | 0 | 0 | 0 | 0 | 0 | 0 | 0 | 0 | 0 | 0 | 0 | 0 | 0 | 0 | 0 |
| | (0-0) | (0-0) | (0-0) | (0-0) | (0-0) | (0-0) | (0-0) | (0-0) | (0-0) | (0-0) | (0-0) | (0-0) | (0-0) | (0-0) | (0-0) | (0-0) |
| Sierra Leone | 47 | 43 | 33 | 23 | 25 | 24 | 19 | 14 | 10 | 10 | 8 | 7 | 37 | 34 | 25 | 16 |
| | (43-50) | (41-47) | (30-37) | (17-31) | (23-27) | (22-26) | (17-20) | (11-19) | (9-11) | (8-11) | (7-10) | (5-10) | (34-40) | (31-36) | (22-28) | (12-21) |
| Singapore | 0 | 0 | 0 | 0 | 0 | 0 | 0 | 0 | 0 | 0 | 0 | 0 | 0 | 0 | 0 | 0 |
| | (0-0) | (0-0) | (0-0) | (0-0) | (0-0) | (0-0) | (0-0) | (0-0) | (0-0) | (0-0) | (0-0) | (0-0) | (0-0) | (0-0) | (0-0) | (0-0) |
| Slovakia | 1 | 1 | 0 | 0 | 1 | 0 | 0 | 0 | 1 | 0 | 0 | 0 | 0 | 0 | 0 | 0 |
| | (1-1) | (1-1) | (0-0) | (0-0) | (1-1) | (0-0) | (0-0) | (0-0) | (1-1) | (0-0) | (0-0) | (0-0) | (0-1) | (0-0) | (0-0) | (0-0) |
| Slovenia | 0 | 0 | 0 | 0 | 0 | 0 | 0 | 0 | 0 | 0 | 0 | 0 | 0 | 0 | 0 | 0 |
| | (0-0) | (0-0) | (0-0) | (0-0) | (0-0) | (0-0) | (0-0) | (0-0) | (0-0) | (0-0) | (0-0) | (0-0) | (0-0) | (0-0) | (0-0) | (0-0) |
| Solomon Islands | 0 | 0 | 0 | 0 | 0 | 0 | 0 | 0 | 0 | 0 | 0 | 0 | 0 | 0 | 0 | 0 |
| | (0-1) | (0-1) | (0-1) | (0-1) | (0-0) | (0-0) | (0-0) | (0-1) | (0-0) | (0-0) | (0-0) | (0-0) | (0-0) | (0-0) | (0-0) | (0-0) |
| Somalia | 63 | 72 | 82 | 78 | 37 | 45 | 53 | 52 | 16 | 20 | 26 | 28 | 47 | 53 | 56 | 51 |
| | (52-78) | (57-94) | (49-142) | (36-171) | (31-46) | (35-58) | (31-92) | (24-112) | (12-21) | (14-28) | (14-50) | (12-66) | (38-58) | (41-69) | (32-98) | (22-111) |
| South Africa | 69 | 75 | 46 | 41 | 61 | 48 | 35 | 29 | 24 | 14 | 14 | 15 | 45 | 61 | 32 | 27 |
| | (60-79) | (69-81) | (43-49) | (36-49) | (54-71) | (44-52) | (33-37) | (25-34) | (20-29) | (11-17) | (12-16) | (12-18) | (38-52) | (56-67) | (29-34) | (22-32) |
| South Sudan | 68 | 51 | 102 | 32 | 38 | 34 | 61 | 24 | 16 | 17 | 16 | 14 | 52 | 34 | 85 | 18 |
| | (57-76) | (43-59) | (52-180) | (8-94) | (31-45) | (29-40) | (32-102) | (6-69) | (11-22) | (12-23) | (8-31) | (3-43) | (43-59) | (27-41) | (42-153) | (4-54) |
| Spain | 4 | 2 | 1 | 1 | 3 | 2 | 1 | 1 | 2 | 1 | 1 | 1 | 2 | 1 | 1 | 0 |
| | (4-4) | (2-2) | (1-1) | (1-1) | (3-3) | (2-2) | (1-1) | (1-1) | (2-2) | (1-1) | (1-1) | (1-1) | (2-2) | (1-1) | (1-1) | (0-1) |
| Sri Lanka | 8 | 6 | 3 | 2 | 6 | 5 | 3 | 2 | 5 | 3 | 2 | 1 | 3 | 2 | 1 | 1 |
| | (8-8) | (5-6) | (3-3) | (2-2) | (6-6) | (4-5) | (3-3) | (1-2) | (5-5) | (3-4) | (2-2) | (1-2) | (3-3) | (2-2) | (1-1) | (0-1) |
| State of Palestine | 4 | 4 | 3 | 6 | 4 | 3 | 2 | 2 | 2 | 2 | 2 | 1 | 2 | 2 | 1 | 4 |
| | (4-5) | (3-4) | (2-3) | (4-8) | (3-4) | (3-3) | (2-3) | (2-4) | (2-2) | (2-2) | (1-2) | (1-2) | (2-2) | (1-2) | (1-1) | (3-6) |
| Sudan | 121 | 112 | 88 | 102 | 80 | 78 | 67 | 71 | 41 | 41 | 40 | 39 | 80 | 71 | 48 | 62 |

| Country | Number of under-five deaths (thousands) 1990 | 2000 | 2015 | 2024 | Number of infant deaths (thousands) 1990 | 2000 | 2015 | 2024 | Number of neonatal deaths (thousands) 1990 | 2000 | 2015 | 2024 | Number of deaths age 1 to 59 months (thousands) 1990 | 2000 | 2015 | 2024 |
|---|---|---|---|---|---|---|---|---|---|---|---|---|---|---|---|---|
| | (111-130) | (102-122) | (73-106) | (65-160) | (74-87) | (72-86) | (56-81) | (45-111) | (37-46) | (36-46) | (32-50) | (25-63) | (73-87) | (64-78) | (38-58) | (39-98) |
| Suriname | 1 | 0 | 0 | 0 | 0 | 0 | 0 | 0 | 0 | 0 | 0 | 0 | 0 | 0 | 0 | 0 |
| | (0-1) | (0-0) | (0-0) | (0-0) | (0-1) | (0-0) | (0-0) | (0-0) | (0-0) | (0-0) | (0-0) | (0-0) | (0-0) | (0-0) | (0-0) | (0-0) |
| Sweden | 1 | 0 | 0 | 0 | 1 | 0 | 0 | 0 | 0 | 0 | 0 | 0 | 0 | 0 | 0 | 0 |
| | (1-1) | (0-0) | (0-0) | (0-0) | (1-1) | (0-0) | (0-0) | (0-0) | (0-0) | (0-0) | (0-0) | (0-0) | (0-0) | (0-0) | (0-0) | (0-0) |
| Switzerland | 1 | 0 | 0 | 0 | 1 | 0 | 0 | 0 | 0 | 0 | 0 | 0 | 0 | 0 | 0 | 0 |
| | (1-1) | (0-0) | (0-0) | (0-0) | (1-1) | (0-0) | (0-0) | (0-0) | (0-0) | (0-0) | (0-0) | (0-0) | (0-0) | (0-0) | (0-0) | (0-0) |
| Syrian Arab Republic | 17 | 12 | 18 | 10 | 15 | 11 | 11 | 10 | 8 | 6 | 4 | 5 | 9 | 6 | 14 | 5 |
| | (15-19) | (11-13) | (12-22) | (5-16) | (13-16) | (10-12) | (7-14) | (5-15) | (7-9) | (5-7) | (3-6) | (3-9) | (8-11) | (5-6) | (9-17) | (2-7) |
| Tajikistan | 22 | 15 | 9 | 8 | 18 | 12 | 7 | 6 | 7 | 5 | 4 | 3 | 15 | 10 | 5 | 5 |
| | (20-25) | (13-16) | (8-10) | (5-11) | (16-19) | (10-13) | (6-8) | (4-10) | (6-8) | (4-6) | (3-4) | (2-5) | (14-17) | (9-11) | (4-6) | (3-7) |
| Thailand | 40 | 21 | 9 | 5 | 33 | 17 | 8 | 5 | 22 | 12 | 5 | 3 | 17 | 9 | 4 | 2 |
| | (37-42) | (18-23) | (8-10) | (5-7) | (31-35) | (15-19) | (7-8) | (4-6) | (18-25) | (8-14) | (3-6) | (2-4) | (15-22) | (7-13) | (3-6) | (1-4) |
| Timor-Leste | 7 | 4 | 2 | 1 | 5 | 3 | 1 | 1 | 2 | 1 | 1 | 1 | 5 | 3 | 1 | 1 |
| | (6-8) | (3-4) | (2-2) | (1-2) | (5-6) | (3-3) | (1-2) | (1-2) | (2-2) | (1-1) | (1-1) | (0-1) | (5-6) | (2-3) | (1-1) | (0-1) |
| Togo | 24 | 22 | 20 | 16 | 14 | 13 | 12 | 10 | 7 | 7 | 7 | 7 | 16 | 15 | 12 | 10 |
| | (22-25) | (21-24) | (17-22) | (11-23) | (13-15) | (13-15) | (10-14) | (7-15) | (6-8) | (6-8) | (6-8) | (4-10) | (15-18) | (14-16) | (11-14) | (6-14) |
| Tonga | 0 | 0 | 0 | 0 | 0 | 0 | 0 | 0 | 0 | 0 | 0 | 0 | 0 | 0 | 0 | 0 |
| | (0-0) | (0-0) | (0-0) | (0-0) | (0-0) | (0-0) | (0-0) | (0-0) | (0-0) | (0-0) | (0-0) | (0-0) | (0-0) | (0-0) | (0-0) | (0-0) |
| Trinidad and Tobago | 1 | 1 | 0 | 0 | 1 | 1 | 0 | 0 | 1 | 0 | 0 | 0 | 0 | 0 | 0 | 0 |
| | (1-1) | (0-1) | (0-1) | (0-0) | (1-1) | (0-1) | (0-1) | (0-0) | (0-1) | (0-0) | (0-0) | (0-0) | (0-0) | (0-0) | (0-0) | (0-0) |
| Tunisia | 12 | 5 | 4 | 2 | 8 | 4 | 4 | 2 | 6 | 3 | 3 | 1 | 6 | 2 | 1 | 1 |
| | (11-14) | (4-5) | (4-4) | (2-2) | (7-9) | (3-4) | (3-4) | (1-2) | (5-7) | (2-3) | (3-3) | (1-1) | (5-7) | (2-2) | (1-1) | (1-1) |
| Turkmenistan | 10 | 7 | 7 | 6 | 9 | 6 | 6 | 5 | 3 | 3 | 4 | 4 | 7 | 4 | 3 | 3 |
| | (9-12) | (6-9) | (6-9) | (4-10) | (8-10) | (5-7) | (4-7) | (3-8) | (3-4) | (3-4) | (3-5) | (2-6) | (6-8) | (4-5) | (2-4) | (2-5) |
| Turks and Caicos Islands | 0 | 0 | 0 | 0 | 0 | 0 | 0 | 0 | 0 | 0 | 0 | 0 | 0 | 0 | 0 | 0 |
| | (0-0) | (0-0) | (0-0) | (0-0) | (0-0) | (0-0) | (0-0) | (0-0) | (0-0) | (0-0) | (0-0) | (0-0) | (0-0) | (0-0) | (0-0) | (0-0) |
| Tuvalu | 0 | 0 | 0 | 0 | 0 | 0 | 0 | 0 | 0 | 0 | 0 | 0 | 0 | 0 | 0 | 0 |
| | (0-0) | (0-0) | (0-0) | (0-0) | (0-0) | (0-0) | (0-0) | (0-0) | (0-0) | (0-0) | (0-0) | (0-0) | (0-0) | (0-0) | (0-0) | (0-0) |
| Türkiye | 107 | 53 | 18 | 10 | 87 | 44 | 15 | 9 | 46 | 25 | 9 | 5 | 60 | 28 | 9 | 5 |
| | (99-115) | (49-58) | (17-19) | (9-12) | (81-94) | (40-48) | (14-16) | (7-10) | (42-51) | (23-28) | (8-10) | (4-6) | (55-66) | (25-31) | (8-10) | (5-6) |
| Uganda | 153 | 159 | 83 | 83 | 93 | 101 | 58 | 61 | 35 | 37 | 34 | 37 | 118 | 122 | 49 | 46 |
| | (143-164) | (149-170) | (75-92) | (68-99) | (87-99) | (94-107) | (52-65) | (50-74) | (31-39) | (33-42) | (30-39) | (28-46) | (110-127) | (113-130) | (43-55) | (36-57) |
| Ukraine | 13 | 7 | 4 | 2 | 13 | 7 | 4 | 2 | 8 | 4 | 3 | 1 | 5 | 3 | 2 | 1 |
| | (12-15) | (7-8) | (4-5) | (1-2) | (12-15) | (6-8) | (4-4) | (1-2) | (7-10) | (4-5) | (2-3) | (1-1) | (4-7) | (2-4) | (1-3) | (0-1) |
| United Arab Emirates | 1 | 1 | 1 | 0 | 1 | 0 | 1 | 0 | 0 | 0 | 0 | 0 | 0 | 0 | 0 | 0 |

| Country | Number of under-five deaths (thousands) | | | | Number of infant deaths (thousands) | | | | Number of neonatal deaths (thousands) | | | | Number of deaths age 1 to 59 months (thousands) | | | |
|---|---|---|---|---|---|---|---|---|---|---|---|---|---|---|---|---|
| | 1990 | 2000 | 2015 | 2024 | 1990 | 2000 | 2015 | 2024 | 1990 | 2000 | 2015 | 2024 | 1990 | 2000 | 2015 | 2024 |
| | (1-1) | (1-1) | (1-1) | (0-1) | (1-1) | (0-0) | (1-1) | (0-0) | (0-0) | (0-0) | (0-0) | (0-0) | (0-0) | (0-0) | (0-0) | (0-0) |
| United Kingdom | 7 | 5 | 3 | 3 | 6 | 4 | 3 | 3 | 4 | 3 | 2 | 2 | 4 | 2 | 1 | 1 |
| | (7-7) | (4-5) | (3-4) | (3-3) | (6-6) | (4-4) | (3-3) | (3-3) | (3-4) | (3-3) | (2-2) | (2-2) | (4-4) | (2-2) | (1-1) | (1-1) |
| United Republic of Tanzania | 182 | 175 | 103 | 86 | 116 | 118 | 79 | 68 | 45 | 46 | 46 | 47 | 137 | 128 | 57 | 39 |
| | (170-194) | (163-186) | (93-114) | (67-111) | (109-124) | (111-126) | (71-87) | (52-87) | (40-50) | (42-51) | (40-52) | (35-64) | (127-147) | (119-138) | (51-64) | (28-52) |
| United States | 46 | 33 | 27 | 24 | 39 | 28 | 23 | 20 | 24 | 18 | 15 | 13 | 22 | 15 | 11 | 10 |
| | (45-47) | (32-33) | (26-27) | (23-25) | (38-39) | (27-28) | (23-23) | (19-21) | (23-25) | (18-19) | (15-16) | (13-14) | (21-23) | (14-15) | (11-12) | (10-11) |
| Uruguay | 1 | 1 | 0 | 0 | 1 | 1 | 0 | 0 | 1 | 0 | 0 | 0 | 1 | 0 | 0 | 0 |
| | (1-1) | (1-1) | (0-0) | (0-0) | (1-1) | (1-1) | (0-0) | (0-0) | (1-1) | (0-0) | (0-0) | (0-0) | (1-1) | (0-0) | (0-0) | (0-0) |
| Uzbekistan | 47 | 33 | 14 | 12 | 40 | 30 | 14 | 12 | 21 | 15 | 8 | 7 | 26 | 18 | 6 | 5 |
| | (42-53) | (29-38) | (13-15) | (11-14) | (36-45) | (26-34) | (12-14) | (11-14) | (18-24) | (13-18) | (7-9) | (6-9) | (22-30) | (16-21) | (5-7) | (4-7) |
| Vanuatu | 0 | 0 | 0 | 0 | 0 | 0 | 0 | 0 | 0 | 0 | 0 | 0 | 0 | 0 | 0 | 0 |
| | (0-0) | (0-0) | (0-0) | (0-0) | (0-0) | (0-0) | (0-0) | (0-0) | (0-0) | (0-0) | (0-0) | (0-0) | (0-0) | (0-0) | (0-0) | (0-0) |
| Venezuela (Bolivarian Republic of) | 17 | 12 | 11 | 10 | 14 | 10 | 9 | 9 | 7 | 6 | 8 | 6 | 9 | 6 | 3 | 4 |
| | (16-17) | (12-13) | (10-12) | (6-17) | (13-14) | (10-10) | (9-10) | (6-14) | (7-8) | (6-7) | (8-9) | (4-10) | (9-10) | (6-7) | (2-3) | (1-8) |
| Viet Nam | 96 | 42 | 36 | 24 | 70 | 30 | 26 | 16 | 45 | 21 | 19 | 12 | 51 | 21 | 17 | 12 |
| | (88-105) | (35-48) | (34-39) | (21-28) | (64-76) | (25-35) | (25-28) | (14-19) | (40-51) | (18-25) | (16-22) | (9-15) | (45-57) | (17-24) | (14-20) | (9-15) |
| Yemen | 85 | 73 | 53 | 52 | 66 | 59 | 45 | 47 | 31 | 29 | 27 | 29 | 54 | 43 | 26 | 23 |
| | (79-91) | (67-79) | (47-61) | (41-68) | (62-71) | (55-65) | (40-52) | (36-61) | (28-34) | (27-33) | (24-31) | (22-38) | (50-58) | (39-47) | (23-30) | (18-31) |
| Zambia | 63 | 66 | 36 | 33 | 36 | 40 | 26 | 24 | 13 | 15 | 14 | 15 | 50 | 51 | 22 | 18 |
| | (59-68) | (62-71) | (33-40) | (27-41) | (34-39) | (37-43) | (23-28) | (19-30) | (12-14) | (14-17) | (13-16) | (12-19) | (47-54) | (47-55) | (20-24) | (14-23) |
| Zimbabwe | 30 | 40 | 29 | 32 | 19 | 25 | 27 | 31 | 7 | 10 | 13 | 17 | 23 | 30 | 16 | 15 |
| | (27-33) | (36-44) | (26-33) | (22-46) | (17-21) | (22-27) | (24-30) | (21-44) | (6-8) | (9-11) | (12-15) | (11-25) | (21-25) | (27-34) | (14-18) | (10-22) |

## 3.6. Table A.6: Country annual rate of reduction

Annual rate of reduction (ARR, %) for under-five mortality rate (U5MR), infant mortality rate (IMR), neonatal mortality rate (NMR), and mortality rate age 1–59 months for the periods 1990-2000, 2000-2015, and 2015-2024, by country. Values shown are medians with 90% uncertainty intervals in parentheses.

| Country | Annual rate of reduction in under-five mortality rate (%) | | | Annual rate of reduction in infant mortality rate (%) | | | Annual rate of reduction in neonatal mortality rate (%) | | | Annual rate of reduction in mortality rate age 1–59 months (%) | | |
|---|---|---|---|---|---|---|---|---|---|---|---|---|
| | 1990-2000 | 2000-2015 | 2015-2024 | 1990-2000 | 2000-2015 | 2015-2024 | 1990-2000 | 2000-2015 | 2015-2024 | 1990-2000 | 2000-2015 | 2015-2024 |
| Afghanistan | 3.2 | 4.0 | 3.4 | 2.8 | 3.6 | 3.1 | 2.0 | 2.7 | 2.5 | 4.3 | 5.7 | 4.8 |
| | (2.4-4) | (3.2-4.9) | (0.5-5.9) | (1.9-3.5) | (2.8-4.5) | (0.2-5.6) | (0.8-3.1) | (1.7-3.7) | (-0.4-5.2) | (3.3-5.3) | (4.7-6.8) | (1.5-7.6) |
| Albania | 4.1 | 6.9 | 0.4 | 4.3 | 5.6 | -0.9 | 0.4 | 4.6 | -1.2 | 6.3 | 9.6 | 3.9 |
| | (2.8-5.3) | (6.2-7.6) | (-1.5-2.4) | (3-5.5) | (5-6.3) | (-2.8-1.1) | (-2.6-3.3) | (3.2-6) | (-3.7-1.6) | (4.3-8.5) | (8.2-10.9) | (-0.7-9.1) |
| Algeria | 2.1 | 3.4 | 1.6 | 1.3 | 3.1 | 1.3 | 0.7 | 2.7 | 0.2 | 3.6 | 4.5 | 4.2 |
| | (1.2-3) | (2.9-4) | (-0.1-3.2) | (0.4-2.2) | (2.5-3.6) | (-0.3-3) | (-0.8-2.2) | (1.8-3.5) | (-1.5-1.9) | (2.1-5.2) | (3.6-5.4) | (2.4-6) |

| | Annual rate of reduction in under-five mortality rate (%) | | | Annual rate of reduction in infant mortality rate (%) | | | Annual rate of reduction in neonatal mortality rate (%) | | | Annual rate of reduction in mortality rate age 1–59 months (%) | | |
|---|---|---|---|---|---|---|---|---|---|---|---|---|
| Country | 1990-2000 | 2000-2015 | 2015-2024 | 1990-2000 | 2000-2015 | 2015-2024 | 1990-2000 | 2000-2015 | 2015-2024 | 1990-2000 | 2000-2015 | 2015-2024 |
| Andorra | 5.4 | 5.1 | 3.7 | 3.3 | 4.5 | 3.6 | 6.0 | 4.5 | 4.0 | 5.0 | 5.8 | 3.5 |
| | (-4.1-14.6) | (-3.6-13.9) | (-1.4-9.2) | (-6.3-12.5) | (-4.2-13.3) | (-1.5-9.1) | (-4.4-16.5) | (-4.7-13.8) | (-2.7-11.2) | (-6.2-14.7) | (-3.6-15.2) | (-2.6-12.1) |
| Angola | 1.4 | 7.3 | 2.6 | 0.9 | 6.9 | 2.1 | 1.6 | 4.2 | 1.6 | 1.4 | 8.6 | 3.1 |
| | (0.1-2.7) | (5.7-8.9) | (-2.7-6.8) | (-0.4-2.2) | (5.3-8.5) | (-3.1-6.3) | (-0.9-5.3) | (2.3-6.2) | (-4-6.5) | (-0.1-2.9) | (6.9-10.3) | (-2.3-7.7) |
| Anguilla | 4.7 | 3.2 | 3.2 | 4.2 | 3.0 | 3.0 | 4.7 | 3.2 | 3.2 | 4.8 | 3.1 | 3.1 |
| | (1.3-8.2) | (-0.6-6.8) | (-1.3-7.6) | (0.7-7.7) | (-0.8-6.7) | (-1.5-7.4) | (0.8-8.9) | (-0.8-7.4) | (-1.7-8.3) | (0.7-9.1) | (-1-7.4) | (-1.9-8.4) |
| Antigua and Barbuda | -0.9 | 2.0 | 2.2 | -0.9 | 2.3 | 2.2 | -2.6 | 3.5 | 2.5 | 2.6 | -0.9 | 1.8 |
| | (-2.5-0.6) | (0.8-3.2) | (-1.4-5.8) | (-2.5-0.7) | (1.1-3.5) | (-1.5-5.8) | (-4.5--0.7) | (2-5) | (-1.5-6.6) | (-0.1-5.2) | (-2.9-1.1) | (-2.6-6.4) |
| Argentina | 4.0 | 3.3 | 2.3 | 4.0 | 3.4 | 2.5 | 3.0 | 3.2 | 3.3 | 5.1 | 3.5 | 1.2 |
| | (3.7-4.2) | (3.1-3.6) | (0.8-3.8) | (3.7-4.2) | (3.2-3.7) | (1-4) | (2.7-3.3) | (3-3.5) | (1.7-4.8) | (4.8-5.5) | (3.2-3.8) | (-0.4-2.9) |
| Armenia | 4.7 | 4.9 | 4.7 | 4.9 | 5.2 | 4.6 | 3.5 | 4.8 | 4.7 | 5.9 | 5.0 | 4.8 |
| | (3.9-5.5) | (3.9-6) | (2.5-6.5) | (4.2-5.8) | (4.2-6.2) | (2.3-6.4) | (1.9-5.2) | (3.1-6.7) | (2-7.2) | (4.3-7.6) | (3.3-7.2) | (1.9-7.5) |
| Australia | 4.0 | 3.1 | 0.8 | 3.9 | 2.9 | 0.7 | 2.8 | 2.7 | 0.4 | 5.4 | 3.7 | 1.6 |
| | (3.7-4.2) | (2.9-3.3) | (0.1-1.5) | (3.7-4.2) | (2.8-3.1) | (0-1.4) | (2.4-3.1) | (2.4-2.9) | (-0.4-1.2) | (4.9-5.8) | (3.4-4) | (0.6-2.6) |
| Austria | 5.5 | 2.6 | 1.2 | 5.6 | 2.6 | 1.1 | 4.0 | 2.1 | 0.4 | 7.1 | 3.5 | 2.6 |
| | (5-5.9) | (2.3-3) | (0.3-2.2) | (5.1-6) | (2.2-2.9) | (0.2-2.1) | (3.4-4.6) | (1.6-2.5) | (-0.7-1.6) | (6.4-7.8) | (2.9-4) | (1.2-4.1) |
| Azerbaijan | 2.5 | 6.5 | 5.0 | 2.1 | 7.1 | 5.8 | -1.6 | 5.2 | 2.8 | 5.0 | 8.1 | 8.8 |
| | (1.3-3.6) | (4.9-8.2) | (0.1-9.4) | (0.9-3.2) | (5.4-8.8) | (1-10.3) | (-3.8-0.5) | (3-7.5) | (-2.6-7.5) | (3.4-6.7) | (5.7-10.4) | (3-14.4) |
| Bahamas | 3.7 | 1.0 | 1.3 | 3.6 | 0.7 | 1.1 | 5.4 | -0.6 | -0.4 | 1.8 | 3.0 | 5.0 |
| | (2.9-4.5) | (0.3-1.6) | (-1.5-4.2) | (2.8-4.4) | (0.1-1.4) | (-1.8-3.9) | (4.1-6.7) | (-1.6-0.3) | (-3.5-2.7) | (0.3-3.2) | (1.9-4.1) | (1-9.3) |
| Bahrain | 6.1 | 3.2 | -1.5 | 6.4 | 3.4 | -1.6 | 11.3 | 2.6 | -3.1 | 0.2 | 3.7 | -0.1 |
| | (5.5-6.7) | (2.8-3.7) | (-2.9-0) | (5.8-7) | (2.9-3.8) | (-3--0.2) | (10.2-12.4) | (1.8-3.4) | (-5--1.2) | (-1.2-1.5) | (3.1-4.4) | (-1.9-1.9) |
| Bangladesh | 5.4 | 5.3 | 2.6 | 4.8 | 4.6 | 2.2 | 4.2 | 4.1 | 3.0 | 6.7 | 7.0 | 2.0 |
| | (5-5.8) | (5-5.6) | (1.5-3.5) | (4.4-5.2) | (4.3-5) | (1.2-3.1) | (3.5-4.8) | (3.6-4.5) | (1.8-4.3) | (6.2-7.3) | (6.5-7.5) | (0.5-3.4) |
| Barbados | 2.3 | 0.8 | 3.0 | 2.2 | 0.7 | 2.9 | 2.8 | 0.2 | 3.1 | 1.6 | 2.0 | 2.8 |
| | (1.4-3.3) | (-0.6-2.3) | (-1.5-7.4) | (1.3-3.2) | (-0.8-2.1) | (-1.6-7.3) | (1.5-4) | (-1.5-1.8) | (-1.6-7.7) | (-0.1-3.4) | (-0.1-4.2) | (-2.2-8.4) |
| Belarus | 1.8 | 7.6 | 6.2 | 2.0 | 7.7 | 6.1 | 6.4 | 8.0 | 8.4 | -3.5 | 7.4 | 5.1 |
| | (1.4-2.1) | (7.3-7.9) | (3.3-9.4) | (1.7-2.3) | (7.4-8) | (3.2-9.2) | (2.3-10.6) | (6.6-9.4) | (2.6-15.9) | (-10.7-1.3) | (6.4-8.3) | (1.3-9.6) |
| Belgium | 5.3 | 2.4 | 1.1 | 5.4 | 2.4 | 0.9 | 4.3 | 1.8 | 0.4 | 6.3 | 3.2 | 2.0 |
| | (5-5.7) | (2.1-2.7) | (0.4-1.8) | (5.1-5.8) | (2.1-2.6) | (0.2-1.6) | (3.7-4.9) | (1.3-2.2) | (-0.6-1.3) | (5.7-6.8) | (2.7-3.6) | (0.9-3.2) |
| Belize | 4.8 | 3.0 | 2.4 | 4.6 | 2.7 | 2.1 | 4.4 | 1.0 | 1.5 | 5.2 | 5.8 | 4.5 |
| | (3.5-6) | (2.4-3.6) | (0.7-4) | (3.4-5.8) | (2.1-3.3) | (0.4-3.8) | (2.5-6.3) | (0.1-1.8) | (-0.5-3.3) | (3.3-7) | (4.8-6.9) | (1.7-7.3) |
| Benin | 2.4 | 2.3 | 2.8 | 2.1 | 2.2 | 2.6 | 1.5 | 1.3 | 1.6 | 2.8 | 2.7 | 3.5 |
| | (1.9-2.9) | (1.7-2.9) | (-0.3-5.8) | (1.6-2.6) | (1.6-2.8) | (-0.5-5.6) | (0.5-2.5) | (0.4-2.3) | (-1.7-4.8) | (2.2-3.3) | (2-3.4) | (0.2-6.6) |
| Bhutan | 5.0 | 6.5 | 5.9 | 4.7 | 6.3 | 5.7 | 3.1 | 5.3 | 7.3 | 6.2 | 7.6 | 4.8 |
| | (3.5-6.6) | (4.9-8) | (2.4-9.5) | (3.3-6.3) | (4.7-7.9) | (2.2-9.4) | (-1.3-7.9) | (2.8-8) | (2.4-13.5) | (3.2-8.7) | (5.3-9.9) | (0-9.7) |
| Bolivia (Plurinational State of) | 5.1 | 6.5 | 5.9 | 4.4 | 5.9 | 5.6 | 3.6 | 5.2 | 6.2 | 6.1 | 7.6 | 5.7 |

| Country | Annual rate of reduction in under-five mortality rate (%) | | | Annual rate of reduction in infant mortality rate (%) | | | Annual rate of reduction in neonatal mortality rate (%) | | | Annual rate of reduction in mortality rate age 1–59 months (%) | | |
|---|---|---|---|---|---|---|---|---|---|---|---|---|
| | 1990-2000 | 2000-2015 | 2015-2024 | 1990-2000 | 2000-2015 | 2015-2024 | 1990-2000 | 2000-2015 | 2015-2024 | 1990-2000 | 2000-2015 | 2015-2024 |
| | (4.6-5.6) | (5.6-7.4) | (3.5-8.3) | (3.9-5) | (5-6.9) | (3.1-8) | (2.7-4.6) | (4.1-6.3) | (3.5-9) | (5.4-6.8) | (6.6-8.7) | (3-8.3) |
| Bosnia and Herzegovina | 6.4 | 2.3 | -0.3 | 6.6 | 2.3 | 0.8 | 5.5 | 2.1 | 0.8 | 8.3 | 2.8 | -2.5 |
| | (6-6.9) | (1.9-2.7) | (-1.7-1.1) | (6.2-7) | (1.9-2.7) | (-0.6-2.2) | (4.9-6.1) | (1.6-2.6) | (-0.8-2.4) | (7.4-9.3) | (1.9-3.6) | (-4.6--0.4) |
| Botswana | -4.4 | 3.6 | 3.8 | 0.1 | -0.8 | 3.5 | 8.6 | -5.9 | 2.4 | -8.5 | 6.9 | 5.1 |
| | (-9--0.2) | (-1.8-7.8) | (-3.2-12.8) | (-4.5-4.3) | (-6.1-3.4) | (-3.5-12.6) | (3.8-13) | (-11.5--1.2) | (-4.9-11.6) | (-13.3--4.2) | (1.2-11.6) | (-2.2-14.9) |
| Brazil | 6.0 | 5.1 | 1.3 | 5.8 | 5.0 | 1.3 | 3.3 | 4.3 | 3.2 | 8.4 | 6.3 | -1.1 |
| | (5.2-6.8) | (4.6-5.6) | (-0.5-3.1) | (5-6.5) | (4.5-5.5) | (-0.5-3) | (1.6-5) | (3.2-5.3) | (1.3-5.1) | (6.9-10.1) | (5.1-7.4) | (-3-0.9) |
| British Virgin Islands | 3.1 | 0.6 | 2.5 | 2.7 | 0.2 | 2.3 | 3.1 | 0.7 | 2.6 | 3.1 | 0.5 | 2.3 |
| | (1.1-5.2) | (-2.5-3.5) | (-1.9-7) | (0.7-4.9) | (-2.8-3.2) | (-2-6.8) | (0.4-6.2) | (-2.7-4.1) | (-2.3-7.6) | (0.3-6.5) | (-3-4.2) | (-2.5-7.6) |
| Brunei Darussalam | 2.6 | -0.2 | 0.8 | 2.1 | -0.6 | 0.5 | 1.8 | -0.2 | -1.1 | 3.2 | -0.3 | 2.9 |
| | (1.7-3.4) | (-0.9-0.4) | (-0.8-2.5) | (1.3-3) | (-1.2-0.1) | (-1.1-2.2) | (0.1-3.5) | (-1.2-0.8) | (-3.2-1) | (1.6-4.7) | (-1.2-0.7) | (0.7-5.2) |
| Bulgaria | 0.5 | 5.1 | 3.9 | 0.2 | 5.0 | 4.2 | 0.2 | 4.4 | 5.0 | 0.8 | 5.7 | 2.9 |
| | (0.2-0.9) | (4.8-5.4) | (3-4.8) | (-0.1-0.6) | (4.7-5.3) | (3.4-5.1) | (-0.3-0.7) | (4-4.8) | (3.8-6.2) | (0.3-1.2) | (5.3-6.1) | (1.8-4) |
| Burkina Faso | 1.1 | 3.7 | 3.3 | 1.1 | 3.3 | 2.8 | 1.2 | 2.4 | 1.9 | 1.1 | 4.3 | 4.0 |
| | (0.5-1.7) | (2.5-4.9) | (-0.9-7.5) | (0.4-1.7) | (2.1-4.5) | (-1.3-7) | (-0.2-2.6) | (0.3-4.6) | (-2.9-6.7) | (0.4-1.8) | (2.9-5.6) | (-0.4-8.5) |
| Burundi | 1.0 | 5.5 | 3.9 | 0.4 | 5.0 | 3.5 | 0.7 | 2.9 | 2.3 | 1.1 | 6.6 | 4.9 |
| | (-0.3-2.2) | (3.6-7.4) | (-0.6-8.6) | (-0.9-1.6) | (3.1-6.9) | (-1-8.2) | (-1-2.6) | (0.8-5.1) | (-2.4-7.2) | (-0.3-2.4) | (4.7-8.7) | (0.3-9.7) |
| Cabo Verde | 4.5 | 4.2 | 6.0 | 3.6 | 3.4 | 5.6 | 0.3 | 2.6 | 4.7 | 7.4 | 6.4 | 8.8 |
| | (4-5) | (3.8-4.6) | (4.2-7.9) | (3.1-4.1) | (3-3.8) | (3.8-7.5) | (-1.7-2.4) | (1.5-3.6) | (2.5-7) | (6-9.1) | (5.2-7.7) | (5.5-12.6) |
| Cambodia | 1.0 | 8.5 | 5.3 | 0.3 | 8.0 | 5.1 | 1.3 | 5.5 | 5.3 | 0.9 | 10.8 | 5.3 |
| | (0.2-1.8) | (6.6-10.3) | (-1.7-12.3) | (-0.4-1.1) | (6.2-9.9) | (-1.9-12.1) | (0.1-2.7) | (3.4-7.6) | (-1.7-12.5) | (-0.1-1.8) | (8.8-12.8) | (-1.8-12.7) |
| Cameroon | -0.5 | 3.2 | 3.5 | -0.2 | 3.0 | 3.1 | 1.3 | 1.2 | 1.9 | -1.1 | 4.1 | 4.4 |
| | (-1.2-0.3) | (2.5-4) | (0.1-7) | (-1-0.6) | (2.3-3.7) | (-0.2-6.7) | (0.1-2.6) | (0.1-2.3) | (-1.7-5.7) | (-1.9--0.2) | (3.3-4.9) | (0.8-8.2) |
| Canada | 2.8 | 1.0 | -0.2 | 2.6 | 0.8 | -0.2 | 1.5 | 0.4 | 0.5 | 4.6 | 2.1 | -1.4 |
| | (2.6-3.1) | (0.8-1.2) | (-0.8-0.4) | (2.3-2.8) | (0.7-1) | (-0.7-0.4) | (1.2-1.9) | (0.2-0.6) | (-0.2-1.2) | (4.2-5) | (1.8-2.4) | (-2.4--0.4) |
| Central African Republic | 0.8 | 2.5 | 2.6 | 0.9 | 2.2 | 2.2 | 1.7 | 1.6 | 1.4 | 0.5 | 2.9 | 3.3 |
| | (-0.3-1.7) | (1.5-3.5) | (-0.2-5.5) | (-0.2-1.9) | (1.2-3.2) | (-0.6-5.1) | (0-3.7) | (0.1-3.2) | (-1.9-4.7) | (-0.8-1.6) | (1.8-4.1) | (0.3-6.4) |
| Chad | 1.5 | 2.3 | 3.1 | 1.4 | 2.3 | 2.7 | 1.7 | 1.4 | 1.7 | 1.5 | 2.7 | 3.8 |
| | (0.8-2.1) | (1.3-3.5) | (-0.9-7.1) | (0.8-2) | (1.3-3.4) | (-1.2-6.7) | (0.3-3.1) | (-0.2-3.1) | (-2.8-6.2) | (0.8-2.2) | (1.6-4) | (-0.4-8) |
| Chile | 5.6 | 2.2 | 1.7 | 5.5 | 2.0 | 1.8 | 4.0 | 0.6 | 1.2 | 7.1 | 4.4 | 2.8 |
| | (5.2-5.9) | (1.9-2.4) | (1.2-2.3) | (5.2-5.9) | (1.8-2.2) | (1.2-2.3) | (3.6-4.4) | (0.3-0.9) | (0.6-1.9) | (6.7-7.5) | (4.1-4.8) | (1.8-3.7) |
| China | 3.8 | 8.2 | 7.0 | 3.7 | 8.6 | 7.8 | 3.3 | 9.3 | 8.0 | 4.6 | 7.0 | 6.1 |
| | (2.8-4.8) | (7.6-8.8) | (5.6-8.4) | (2.7-4.7) | (8.1-9.2) | (6.4-9.2) | (1.8-4.7) | (8.5-10.1) | (6.1-9.9) | (2.8-6.3) | (6.2-7.9) | (4.3-7.9) |
| Colombia | 3.6 | 3.2 | 3.2 | 3.2 | 2.9 | 3.1 | 3.0 | 3.1 | 3.3 | 4.3 | 3.4 | 3.2 |
| | (2.8-4.3) | (1.8-4.7) | (-1.2-7.5) | (2.4-3.9) | (1.5-4.4) | (-1.3-7.4) | (1.8-4) | (1.5-4.8) | (-1.2-7.7) | (3.2-5.5) | (1.6-5.2) | (-1.3-7.8) |
| Comoros | 4.1 | 3.3 | 2.5 | 3.6 | 2.8 | 2.2 | 3.4 | 1.9 | 1.9 | 4.8 | 4.7 | 3.3 |
| | (2.8-5.9) | (2.1-4.4) | (-0.6-5.2) | (2.3-5.4) | (1.7-3.9) | (-1-4.9) | (1-6.1) | (-0.1-3.6) | (-1.8-5.5) | (2.8-7) | (3-6.5) | (-0.9-7.4) |

| | Annual rate of reduction in under-five mortality rate (%) | | | Annual rate of reduction in infant mortality rate (%) | | | Annual rate of reduction in neonatal mortality rate (%) | | | Annual rate of reduction in mortality rate age 1–59 months (%) | | |
|---|---|---|---|---|---|---|---|---|---|---|---|---|
| **Country** | **1990-2000** | **2000-2015** | **2015-2024** | **1990-2000** | **2000-2015** | **2015-2024** | **1990-2000** | **2000-2015** | **2015-2024** | **1990-2000** | **2000-2015** | **2015-2024** |
| Congo | -2.1 | 5.1 | 3.3 | -2.4 | 4.8 | 3.0 | -0.8 | 3.0 | 2.0 | -2.6 | 6.1 | 4.2 |
| | (-3.4--0.8) | (2.8-7.6) | (-1.7-8) | (-3.7--1.1) | (2.6-7.4) | (-2-7.7) | (-2.8-1.1) | (0.4-5.8) | (-3.2-6.8) | (-4.1--1.2) | (3.7-8.8) | (-1-9.1) |
| Cook Islands | 2.4 | 2.2 | 0.1 | 2.4 | 2.2 | -0.1 | 2.5 | 2.2 | 0.2 | 2.4 | 2.2 | -0.1 |
| | (1.1-3.8) | (0.6-3.8) | (-3.7-3.8) | (1.1-3.7) | (0.6-3.8) | (-3.8-3.7) | (0.2-5) | (0.1-4.7) | (-4.2-4.5) | (0-5.4) | (-0.1-4.9) | (-4.4-4.6) |
| Costa Rica | 2.6 | 2.5 | -1.6 | 2.5 | 2.4 | -1.7 | 1.6 | 1.4 | -1.8 | 3.8 | 4.4 | -1.1 |
| | (2.3-2.9) | (2.2-2.7) | (-2.3--0.9) | (2.2-2.8) | (2.2-2.7) | (-2.4--1) | (1.1-2.1) | (1-1.7) | (-2.6--1) | (3.2-4.4) | (3.9-4.9) | (-2.3-0.2) |
| Croatia | 4.4 | 3.5 | -1.5 | 4.4 | 3.6 | 0.2 | 3.9 | 3.9 | 1.3 | 5.3 | 2.8 | -5.1 |
| | (3.8-4.8) | (3.2-3.9) | (-3.2-0) | (3.9-4.9) | (3.2-4) | (-1.5-1.8) | (3.2-4.6) | (3.4-4.5) | (-0.8-3.3) | (4.2-6.3) | (2-3.6) | (-7.3--3) |
| Cuba | 4.5 | 2.2 | -3.7 | 4.7 | 2.3 | -3.8 | 5.0 | 3.2 | -5.6 | 4.1 | 1.5 | -2.1 |
| | (3-6) | (1.1-3.5) | (-6.2--1.5) | (3.1-6.2) | (1.1-3.5) | (-6.3--1.6) | (3.4-6.5) | (2-4.4) | (-8.2--3.2) | (2.5-5.6) | (0.3-2.7) | (-4.7-0.2) |
| Cyprus | 5.3 | 6.4 | -7.6 | 5.6 | 6.7 | -7.4 | 5.3 | 6.3 | -7.3 | 5.4 | 6.6 | -8.1 |
| | (4.4-6.4) | (5.4-7.5) | (-11--4.4) | (4.6-6.6) | (5.6-7.7) | (-10.8--4.2) | (3.1-7.3) | (4.7-7.8) | (-11--3.6) | (3-7.8) | (4.9-8.4) | (-12--4.1) |
| Czechia | 8.0 | 3.6 | 2.2 | 8.4 | 3.7 | 2.2 | 9.9 | 3.5 | 1.4 | 5.8 | 3.8 | 3.1 |
| | (7.6-8.4) | (3.3-4) | (1.3-3.2) | (8-8.8) | (3.4-4.1) | (1.2-3.2) | (9.3-10.6) | (2.9-4) | (0-2.8) | (5.1-6.5) | (3.2-4.3) | (1.7-4.6) |
| Côte d'Ivoire | 0.9 | 3.3 | 3.1 | 1.0 | 3.0 | 2.9 | 1.6 | 1.6 | 1.8 | 0.7 | 4.2 | 4.1 |
| | (0.2-1.6) | (2.7-3.9) | (0.9-5.4) | (0.3-1.7) | (2.4-3.6) | (0.7-5.2) | (0.4-2.8) | (0.5-2.5) | (-0.8-4.5) | (-0.2-1.5) | (3.5-5) | (1.6-6.6) |
| Democratic People's Republic of Korea | -9.0 | 10.3 | 2.8 | -6.2 | 8.3 | 2.8 | -1.1 | 4.9 | 2.7 | -13.7 | 13.7 | 2.8 |
| | (-9--9) | (10.3-10.3) | (2.8-2.8) | (-6.2--6.2) | (8.3-8.3) | (2.8-2.8) | (-3-1.2) | (3.5-6.9) | (0.9-5.2) | (-16.2--11.9) | (11.6-17.1) | (0.6-5.4) |
| Democratic Republic of the Congo | -0.3 | 4.6 | 0.3 | -0.6 | 4.3 | -0.3 | 1.3 | 2.3 | 0.2 | -0.6 | 5.4 | 0.3 |
| | (-1.4-0.8) | (3.7-5.7) | (-2.7-2.8) | (-1.8-0.4) | (3.3-5.3) | (-3.2-2.3) | (-0.6-3.3) | (1-3.8) | (-3.4-3.4) | (-1.8-0.6) | (4.3-6.4) | (-2.7-3) |
| Denmark | 4.6 | 2.2 | 0.3 | 4.5 | 1.9 | 0.0 | 2.3 | 1.2 | 0.8 | 7.5 | 4.1 | -0.8 |
| | (4.1-5.1) | (1.8-2.6) | (-1.1-1.5) | (4-5) | (1.5-2.3) | (-1.4-1.2) | (1.5-3) | (0.7-1.8) | (-1-2.5) | (6.6-8.4) | (3.2-5.1) | (-3.6-2) |
| Djibouti | 1.6 | 2.8 | 3.2 | 0.9 | 2.3 | 3.0 | 1.2 | 1.7 | 2.3 | 1.9 | 3.8 | 4.3 |
| | (-0.3-3.1) | (0.3-5.6) | (-1.6-7.9) | (-1-2.4) | (-0.1-5.2) | (-1.7-7.8) | (-1.3-3.6) | (-1.3-4.8) | (-2.8-7.2) | (-0.4-3.9) | (1-7.1) | (-0.9-9.8) |
| Dominica | -0.4 | -2.7 | -2.2 | -1.1 | -3.2 | -2.4 | -1.5 | -4.7 | -2.2 | 1.2 | 3.9 | -2.2 |
| | (-1-0.1) | (-3.4--2.1) | (-3.3--1.2) | (-1.6--0.6) | (-3.8--2.6) | (-3.5--1.4) | (-2.7--0.3) | (-5.5--3.9) | (-3.4--1) | (-0.5-3) | (2.2-5.6) | (-6-1.8) |
| Dominican Republic | 4.0 | 0.8 | 1.4 | 3.6 | 0.5 | 1.3 | 0.6 | -0.4 | 1.5 | 7.4 | 3.1 | 1.3 |
| | (3.3-4.9) | (-0.3-2) | (-1.9-4.7) | (2.8-4.4) | (-0.6-1.6) | (-2.1-4.6) | (-0.6-1.8) | (-1.7-0.9) | (-2-5.1) | (6.3-8.6) | (1.4-4.7) | (-2.9-6.2) |
| Ecuador | 6.0 | 4.4 | 1.9 | 5.7 | 4.2 | 1.8 | 4.6 | 4.4 | 0.4 | 7.2 | 4.4 | 3.5 |
| | (4.9-7.1) | (3.6-5.2) | (0.5-3.5) | (4.6-6.8) | (3.4-5) | (0.4-3.4) | (2.6-6.4) | (3.3-5.6) | (-1.8-2.6) | (5.6-8.9) | (3.2-5.5) | (1.4-6.1) |
| Egypt | 6.0 | 3.9 | 1.9 | 5.2 | 3.4 | 1.6 | 3.8 | 2.6 | 1.1 | 7.7 | 5.3 | 3.0 |
| | (5.4-6.6) | (3-4.7) | (-1.1-4.8) | (4.6-5.8) | (2.6-4.2) | (-1.4-4.5) | (2.9-4.7) | (1.6-3.6) | (-2.1-4.2) | (6.9-8.5) | (4.2-6.4) | (-0.4-6.9) |
| El Salvador | 6.2 | 5.3 | 4.1 | 5.8 | 5.0 | 3.9 | 4.4 | 5.5 | 4.3 | 7.5 | 5.2 | 4.0 |
| | (5.2-7.1) | (3.4-7.1) | (0.1-7.5) | (4.8-6.8) | (3.1-6.8) | (-0.1-7.2) | (2.9-5.8) | (3.4-7.5) | (0.1-8.1) | (6.2-8.8) | (3.2-7.1) | (-0.1-7.6) |
| Equatorial Guinea | 1.3 | 3.4 | 3.4 | 1.1 | 3.5 | 3.1 | 0.8 | 2.5 | 2.0 | 1.6 | 4.0 | 4.2 |
| | (-0.4-2.7) | (1.2-6.1) | (-1.4-8.1) | (-0.7-2.5) | (1.2-6.2) | (-1.7-7.8) | (-3-4.7) | (-0.8-6.5) | (-3.7-7.8) | (-0.7-3.5) | (1.4-7.1) | (-0.9-9.7) |
| Eritrea | 5.9 | 4.1 | 3.4 | 4.6 | 3.0 | 3.0 | 2.9 | 2.0 | 2.4 | 7.0 | 5.3 | 4.1 |

| | Annual rate of reduction in under-five mortality rate (%) | | | Annual rate of reduction in infant mortality rate (%) | | | Annual rate of reduction in neonatal mortality rate (%) | | | Annual rate of reduction in mortality rate age 1–59 months (%) | | |
|---|---|---|---|---|---|---|---|---|---|---|---|---|
| Country | 1990-2000 | 2000-2015 | 2015-2024 | 1990-2000 | 2000-2015 | 2015-2024 | 1990-2000 | 2000-2015 | 2015-2024 | 1990-2000 | 2000-2015 | 2015-2024 |
| | (5.1-6.7) | (1.8-6.3) | (-1.7-8.1) | (3.8-5.5) | (0.7-5.3) | (-2-7.7) | (1.2-4.4) | (-0.7-4.6) | (-2.8-7.4) | (6.1-8) | (2.8-7.8) | (-1-9.2) |
| Estonia | 4.7 | 8.3 | 5.0 | 4.7 | 8.5 | 5.2 | 6.2 | 8.8 | 4.6 | 3.2 | 7.9 | 5.4 |
| | (4.1-5.4) | (7.7-9) | (2.7-7.4) | (4.1-5.3) | (7.9-9.2) | (2.9-7.6) | (5.2-7.2) | (7.8-9.8) | (1.4-7.7) | (2.1-4.2) | (7-8.9) | (2.6-8.6) |
| Eswatini | -5.3 | 4.5 | 2.7 | -2.4 | 1.9 | 2.8 | -1.7 | -0.4 | 0.7 | -6.6 | 6.9 | 4.8 |
| | (-6.4--4.1) | (2.7-6.1) | (-1-6.5) | (-3.5--1.2) | (0.2-3.5) | (-0.9-6.6) | (-3.6-0.2) | (-2.4-1.4) | (-3.5-4.9) | (-8--5.3) | (5.1-8.7) | (0.6-9.4) |
| Ethiopia | 3.7 | 5.2 | 4.1 | 3.2 | 4.4 | 3.7 | 2.1 | 2.7 | 2.7 | 4.5 | 7.1 | 5.8 |
| | (3.1-4.2) | (4.4-6) | (0.7-7.5) | (2.7-3.7) | (3.6-5.1) | (0.2-7) | (1.2-3.1) | (1.6-3.7) | (-0.8-6.2) | (3.8-5.2) | (6.2-8.1) | (2.1-9.4) |
| Fiji | 2.4 | 0.5 | -3.7 | 2.6 | 0.2 | -4.2 | 2.8 | 1.0 | -6.3 | 2.1 | 0.2 | -1.5 |
| | (1.1-3.7) | (0.1-0.9) | (-6.1--1.3) | (1.2-3.9) | (-0.2-0.6) | (-6.6--1.8) | (0-5.5) | (0.1-2) | (-9.4--3.1) | (-0.3-4.4) | (-0.6-0.9) | (-4.6-1.8) |
| Finland | 4.5 | 3.6 | 0.6 | 4.6 | 3.8 | 0.7 | 4.4 | 3.9 | 0.2 | 4.7 | 3.3 | 1.2 |
| | (4-5.1) | (3.2-4.1) | (-0.8-1.9) | (4.1-5.2) | (3.3-4.2) | (-0.7-2) | (3.7-5.2) | (3.3-4.5) | (-1.4-1.8) | (3.8-5.6) | (2.6-4) | (-0.7-3) |
| France | 5.1 | 1.7 | -0.5 | 6.0 | 1.7 | -0.8 | 2.7 | 0.8 | -1.7 | 7.1 | 2.8 | 1.5 |
| | (4.9-5.4) | (1.5-1.9) | (-1.1-0.1) | (5.7-6.3) | (1.5-1.8) | (-1.4--0.2) | (2.4-3.1) | (0.6-1.1) | (-2.5--0.8) | (6.8-7.5) | (2.5-3.1) | (0.4-2.8) |
| Gabon | 1.4 | 3.3 | 3.4 | 1.5 | 2.7 | 2.9 | 0.5 | 1.5 | 2.9 | 1.9 | 4.7 | 3.9 |
| | (0.3-2.4) | (2.1-4.5) | (-0.1-6.7) | (0.4-2.5) | (1.4-3.9) | (-0.6-6.2) | (-1.6-2.5) | (-0.1-3) | (-1.2-6.9) | (0.5-3.2) | (3.2-6.1) | (0-8.3) |
| Gambia | 3.9 | 4.3 | 3.7 | 3.1 | 3.5 | 3.2 | 2.1 | 2.2 | 2.6 | 4.8 | 6.0 | 4.9 |
| | (3-4.8) | (3.5-5.3) | (0.5-6.9) | (2.2-4) | (2.6-4.4) | (0-6.4) | (-0.1-4.3) | (0.9-3.4) | (-1-6.3) | (3.5-6.1) | (4.9-7.2) | (1.2-8.9) |
| Georgia | 2.8 | 8.1 | 2.3 | 2.8 | 7.9 | 2.0 | -0.1 | 8.0 | 3.4 | 6.2 | 8.5 | 0.5 |
| | (1.3-4.1) | (7.2-9.2) | (1.2-3.3) | (1.3-4.1) | (6.9-9) | (0.9-3) | (-1.9-1.7) | (6.8-9.2) | (2.2-4.6) | (4.2-8.2) | (7.2-9.9) | (-0.9-2) |
| Germany | 4.7 | 2.1 | 0.6 | 4.6 | 1.9 | 0.6 | 1.9 | 1.3 | -0.1 | 6.9 | 3.1 | 1.6 |
| | (4.4-4.9) | (1.9-2.2) | (0.1-1.1) | (4.4-4.9) | (1.7-2.1) | (0.1-1.1) | (1.6-2.3) | (1-1.5) | (-0.7-0.6) | (6.6-7.3) | (2.8-3.3) | (0.8-2.5) |
| Ghana | 2.4 | 4.3 | 4.2 | 1.6 | 3.3 | 3.6 | 1.8 | 2.5 | 3.3 | 2.9 | 5.6 | 5.2 |
| | (1.8-3.1) | (3.7-4.9) | (1.5-7) | (1-2.3) | (2.7-3.8) | (0.8-6.3) | (0.8-2.8) | (1.6-3.3) | (0.3-6.4) | (2.1-3.7) | (4.9-6.3) | (2.2-8.3) |
| Greece | 4.9 | 2.7 | 1.3 | 5.2 | 2.8 | 1.2 | 5.1 | 2.8 | 1.9 | 4.6 | 2.5 | 0.3 |
| | (4.5-5.3) | (2.4-3) | (0.1-2.5) | (4.8-5.5) | (2.5-3.1) | (0-2.4) | (4.5-5.6) | (2.4-3.2) | (0.4-3.5) | (4-5.3) | (2-3.1) | (-1.4-2.2) |
| Grenada | 3.7 | -0.8 | -0.3 | 3.3 | -1.1 | -0.5 | 4.6 | -2.6 | -0.7 | 2.7 | 1.6 | 0.6 |
| | (2.6-4.8) | (-1.7-0.1) | (-3.3-2.8) | (2.1-4.4) | (-2--0.2) | (-3.5-2.6) | (2.8-6.3) | (-3.9--1.4) | (-4.1-2.6) | (0.8-4.6) | (0.1-3.1) | (-3.5-5.1) |
| Guatemala | 4.3 | 4.1 | 3.5 | 4.0 | 3.8 | 3.3 | 3.0 | 3.2 | 3.5 | 5.2 | 4.8 | 3.5 |
| | (3.7-5.1) | (3.1-5.1) | (-0.4-7.4) | (3.3-4.7) | (2.8-4.8) | (-0.6-7.2) | (1.9-4.3) | (1.9-4.6) | (-0.5-7.6) | (4.3-6.1) | (3.6-6.1) | (-0.5-7.7) |
| Guinea | 3.4 | 2.5 | 2.3 | 3.2 | 2.1 | 1.9 | 2.9 | 2.1 | 1.4 | 3.8 | 2.8 | 2.8 |
| | (2.9-4) | (1.8-3.2) | (-0.6-5.2) | (2.7-3.8) | (1.4-2.8) | (-1-4.9) | (1.7-4.1) | (0.9-3.3) | (-2.4-5.1) | (3.1-4.5) | (1.9-3.6) | (-0.3-6.1) |
| Guinea-Bissau | 2.5 | 4.3 | 3.4 | 2.1 | 4.0 | 3.0 | 1.5 | 2.3 | 2.2 | 3.0 | 5.6 | 4.5 |
| | (1.4-3.5) | (2.3-6.6) | (-0.9-8) | (1-3.1) | (2-6.3) | (-1.3-7.6) | (-0.3-3.4) | (-0.2-4.8) | (-2.5-7.1) | (1.7-4.2) | (3.4-8.1) | (-0.3-9.7) |
| Guyana | 2.7 | 2.3 | 3.1 | 2.5 | 2.1 | 3.0 | 1.4 | 1.9 | 3.2 | 4.4 | 2.8 | 3.1 |
| | (1.6-3.9) | (-0.2-4.7) | (-1.4-7.6) | (1.4-3.7) | (-0.4-4.5) | (-1.5-7.5) | (-0.3-3) | (-0.7-4.7) | (-1.5-7.9) | (2.7-6.3) | (-0.1-5.7) | (-1.9-8.2) |
| Haiti | 3.4 | 2.7 | 3.0 | 3.3 | 2.4 | 2.7 | 2.7 | 0.7 | 1.8 | 3.8 | 3.7 | 3.9 |
| | (2.7-4.2) | (1.3-3.8) | (-1.1-7.2) | (2.6-4) | (1-3.5) | (-1.4-6.8) | (1.3-4.1) | (-1.2-2.3) | (-2.8-6.6) | (3-4.7) | (2.2-5.1) | (-0.5-8.5) |

| Country | Annual rate of reduction in under-five mortality rate (%) 1990-2000 | 2000-2015 | 2015-2024 | Annual rate of reduction in infant mortality rate (%) 1990-2000 | 2000-2015 | 2015-2024 | Annual rate of reduction in neonatal mortality rate (%) 1990-2000 | 2000-2015 | 2015-2024 | Annual rate of reduction in mortality rate age 1–59 months (%) 1990-2000 | 2000-2015 | 2015-2024 |
|---|---|---|---|---|---|---|---|---|---|---|---|---|
| Honduras | 4.5 | 4.0 | 3.4 | 4.1 | 3.7 | 3.2 | 2.1 | 3.0 | 3.5 | 6.3 | 5.2 | 3.4 |
| | (3.8-5.1) | (3-5) | (0.2-6.6) | (3.4-4.8) | (2.6-4.7) | (-0.1-6.4) | (0.7-3.4) | (1.6-4.3) | (-0.1-7.1) | (5.2-7.4) | (3.8-6.7) | (-0.3-7.4) |
| Hungary | 5.3 | 4.6 | 3.4 | 5.6 | 4.8 | 3.6 | 6.6 | 5.0 | 3.0 | 3.3 | 4.1 | 4.0 |
| | (4.9-5.7) | (4.3-4.9) | (2.1-4.8) | (5.3-6) | (4.5-5.1) | (2.2-4.9) | (6.2-7.1) | (4.6-5.5) | (1.3-4.7) | (2.7-3.9) | (3.7-4.6) | (2.2-5.8) |
| Iceland | 4.9 | 2.7 | -0.8 | 5.1 | 2.9 | -0.7 | 5.3 | 3.0 | -1.0 | 4.6 | 2.4 | -0.7 |
| | (3.6-6.3) | (1.5-3.8) | (-3.9-2.3) | (3.8-6.5) | (1.8-4.1) | (-3.8-2.4) | (3.5-7.1) | (1.4-4.5) | (-4.5-2.6) | (2.7-6.5) | (0.9-3.9) | (-4.2-3) |
| India | 3.3 | 4.9 | 5.5 | 2.4 | 4.0 | 4.9 | 2.6 | 3.5 | 5.0 | 3.9 | 6.8 | 6.6 |
| | (2.8-3.7) | (4.5-5.3) | (4.3-6.7) | (2-2.8) | (3.6-4.4) | (3.7-6.1) | (1.9-3.3) | (2.9-4) | (3.6-6.3) | (3.3-4.5) | (6.2-7.4) | (5-8.1) |
| Indonesia | 4.8 | 4.5 | 4.3 | 4.4 | 4.2 | 4.1 | 2.9 | 3.4 | 4.2 | 6.1 | 5.5 | 4.5 |
| | (4.4-5.3) | (3.9-5.1) | (2.6-6.1) | (4-4.9) | (3.6-4.8) | (2.3-5.8) | (2.2-3.7) | (2.6-4.2) | (2.2-6.2) | (5.5-6.8) | (4.8-6.3) | (2.5-6.6) |
| Iran (Islamic Republic of) | 4.7 | 5.7 | 3.5 | 4.3 | 5.5 | 3.4 | 2.4 | 4.4 | 3.7 | 6.8 | 7.6 | 3.2 |
| | (3.6-5.6) | (3.3-8.1) | (-1.4-8.2) | (3.3-5.3) | (3.1-7.9) | (-1.5-8.1) | (-2.7-6.3) | (1.3-7.8) | (-1.9-10.3) | (3.1-10.5) | (3.8-13.5) | (-4-12.4) |
| Iraq | 6.1 | 2.8 | 3.3 | 3.7 | 2.4 | 3.1 | 1.2 | 2.4 | 3.3 | 9.8 | 3.3 | 3.3 |
| | (5.3-6.8) | (1.6-4) | (-0.1-6.6) | (2.9-4.4) | (1.3-3.7) | (-0.2-6.5) | (-0.1-2.3) | (1-3.8) | (-0.2-6.8) | (8.8-10.9) | (1.9-4.8) | (-0.3-7) |
| Ireland | 2.5 | 4.5 | -0.6 | 2.4 | 4.2 | -0.9 | 1.6 | 3.4 | -0.8 | 3.5 | 6.1 | -0.1 |
| | (2-3) | (4.1-4.9) | (-2.6-1.3) | (1.9-2.9) | (3.8-4.6) | (-2.9-1) | (0.9-2.3) | (2.9-4) | (-3-1.3) | (2.7-4.2) | (5.4-6.8) | (-2.9-2.5) |
| Israel | 5.2 | 3.8 | 1.8 | 5.4 | 3.8 | 1.9 | 5.5 | 3.6 | 2.2 | 4.8 | 3.9 | 1.3 |
| | (4.9-5.5) | (3.5-4) | (0.9-2.6) | (5.1-5.8) | (3.6-4.1) | (1-2.7) | (5-6) | (3.3-4) | (1.1-3.3) | (4.3-5.3) | (3.6-4.3) | (0.2-2.5) |
| Italy | 5.5 | 3.1 | 2.9 | 5.7 | 3.1 | 2.9 | 6.2 | 3.2 | 1.9 | 4.3 | 2.8 | 4.8 |
| | (5.2-5.8) | (2.9-3.3) | (2.2-3.6) | (5.4-6) | (2.9-3.2) | (2.2-3.6) | (5.9-6.5) | (3-3.5) | (0.8-2.9) | (3.9-4.7) | (2.5-3.1) | (3.2-6.5) |
| Jamaica | 3.1 | 0.6 | 0.6 | 2.9 | 0.5 | 0.5 | 1.4 | 0.7 | 0.5 | 7.5 | 0.2 | 0.7 |
| | (1.3-4.9) | (-0.4-1.6) | (-0.4-1.5) | (1.1-4.7) | (-0.5-1.5) | (-0.4-1.5) | (-0.6-3.4) | (-0.3-1.8) | (-0.6-1.6) | (4.7-10.3) | (-1.5-1.8) | (-1.2-2.8) |
| Japan | 3.4 | 3.3 | 1.4 | 3.4 | 3.2 | 1.2 | 3.7 | 4.3 | 0.7 | 3.2 | 2.7 | 1.8 |
| | (3.1-3.7) | (3.1-3.4) | (1-1.8) | (3.1-3.6) | (3.1-3.4) | (0.7-1.6) | (3.4-4.1) | (4-4.6) | (0-1.3) | (2.9-3.5) | (2.5-2.9) | (1.3-2.3) |
| Jordan | 3.2 | 3.0 | 2.7 | 3.0 | 2.8 | 2.5 | 2.5 | 3.1 | 3.0 | 4.2 | 2.9 | 2.4 |
| | (2.4-3.9) | (1.8-4) | (0-5) | (2.2-3.8) | (1.6-3.9) | (-0.2-4.8) | (1.4-3.5) | (1.8-4.2) | (0-5.7) | (3-5.5) | (1.5-4.2) | (-0.9-5.2) |
| Kazakhstan | 1.9 | 8.4 | 2.5 | 2.1 | 8.9 | 2.7 | -0.6 | 9.7 | 3.7 | 4.4 | 7.2 | 1.7 |
| | (1-2.8) | (7.9-9.1) | (1.8-3.2) | (1.2-3) | (8.3-9.5) | (2-3.4) | (-2.3-1.1) | (8.7-10.8) | (2.6-4.7) | (2.7-6.2) | (6.1-8.3) | (0.8-2.6) |
| Kenya | 0.6 | 4.7 | 2.1 | 0.6 | 3.3 | 1.5 | 0.1 | 0.8 | 1.3 | 0.8 | 7.2 | 2.9 |
| | (-0.1-1.3) | (4.1-5.3) | (-0.1-4.3) | (-0.2-1.3) | (2.7-3.9) | (-0.7-3.7) | (-1-1.2) | (0-1.7) | (-1.2-3.8) | (0-1.6) | (6.4-8) | (0.4-5.7) |
| Kiribati | 3.3 | 0.8 | 1.1 | 3.4 | 1.0 | 1.0 | 2.4 | 1.0 | 0.8 | 3.9 | 0.6 | 1.3 |
| | (2-4.5) | (-0.9-2.5) | (-2.7-4.8) | (2.1-4.7) | (-0.7-2.7) | (-2.8-4.7) | (-0.7-6.2) | (-1.8-4.9) | (-4.2-6.3) | (1.2-6.1) | (-1.7-3.2) | (-2.9-6.1) |
| Kosovo (UNSCR 1244) | 7.7 | 8.1 | 5.5 | 7.4 | 8.0 | 5.4 | 4.6 | 6.5 | 5.0 | 10.8 | 11.1 | 7.0 |
| | (5.2-10.6) | (6.9-9.3) | (2.1-8.9) | (4.9-10.3) | (6.8-9.2) | (2-8.9) | (1.6-8) | (5.1-8) | (1.2-8.7) | (7.8-14.2) | (9.3-12.9) | (1.9-13) |
| Kuwait | 3.0 | 2.2 | 1.3 | 3.1 | 2.1 | 1.2 | 4.3 | 1.9 | 1.3 | 1.5 | 2.6 | 1.4 |
| | (2.6-3.5) | (1.9-2.5) | (0.6-2.1) | (2.6-3.5) | (1.8-2.3) | (0.4-2) | (3.5-5.2) | (1.4-2.3) | (0.3-2.4) | (0.3-2.5) | (2.1-3.2) | (0.2-2.5) |
| Kyrgyzstan | 2.4 | 5.5 | 3.2 | 2.5 | 5.4 | 3.1 | 1.6 | 2.8 | 2.6 | 3.0 | 8.3 | 4.2 |

| Country | Annual rate of reduction in under-five mortality rate (%) | | | Annual rate of reduction in infant mortality rate (%) | | | Annual rate of reduction in neonatal mortality rate (%) | | | Annual rate of reduction in mortality rate age 1–59 months (%) | | |
|---|---|---|---|---|---|---|---|---|---|---|---|---|
| | 1990-2000 | 2000-2015 | 2015-2024 | 1990-2000 | 2000-2015 | 2015-2024 | 1990-2000 | 2000-2015 | 2015-2024 | 1990-2000 | 2000-2015 | 2015-2024 |
| | (1-3.7) | (4.8-6.1) | (2.1-4.2) | (1.1-3.7) | (4.7-6.1) | (2-4.1) | (-0.6-3.7) | (1.5-3.9) | (0.5-4.7) | (1.2-4.6) | (7.1-9.5) | (1.1-8.1) |
| Lao People's Democratic Republic | 4.0 | 5.4 | 4.6 | 3.0 | 4.8 | 4.3 | 2.8 | 4.3 | 4.0 | 4.7 | 6.3 | 5.1 |
| | (3.1-4.7) | (4.4-6.5) | (1.9-7.1) | (2.2-3.8) | (3.9-5.9) | (1.6-6.8) | (1.4-4.3) | (2.9-5.7) | (0.9-7.3) | (3.7-5.7) | (5.1-7.5) | (2.1-8) |
| Latvia | 1.7 | 6.8 | 8.0 | 1.2 | 6.5 | 7.8 | 1.4 | 6.4 | 9.3 | 2.0 | 7.3 | 6.7 |
| | (1.1-2.2) | (6.3-7.4) | (5.8-10.3) | (0.7-1.8) | (6-7.1) | (5.6-10.1) | (0.5-2.3) | (5.6-7.2) | (6.5-12.3) | (1.1-2.8) | (6.5-8.2) | (3.9-9.6) |
| Lebanon | 4.7 | 2.9 | -3.4 | 4.7 | 3.0 | -3.3 | 5.1 | 3.0 | -3.5 | 4.1 | 2.8 | -3.4 |
| | (3-6.3) | (1.7-4.1) | (-5.5--1.4) | (3.1-6.4) | (1.8-4.1) | (-5.4--1.3) | (3.2-7.2) | (1.6-4.3) | (-5.6--1.3) | (1.5-6.3) | (1.2-4.4) | (-5.7--1) |
| Lesotho | -2.5 | 2.4 | 2.5 | 0.1 | 0.5 | 2.2 | 0.4 | 0.6 | 2.0 | -4.4 | 3.7 | 3.0 |
| | (-3.6--1.3) | (1.5-3.3) | (-0.9-6.3) | (-0.9-1.3) | (-0.4-1.4) | (-1.2-6) | (-1-1.8) | (-0.5-1.6) | (-1.9-6.2) | (-5.7--3) | (2.7-4.7) | (-0.7-7.2) |
| Liberia | 3.3 | 4.9 | 0.8 | 3.1 | 4.4 | 0.3 | 2.1 | 2.5 | 0.4 | 3.8 | 6.1 | 1.1 |
| | (2.5-4.1) | (4.1-5.7) | (-1.8-3.9) | (2.3-3.9) | (3.6-5.1) | (-2.4-3.4) | (0.5-3.6) | (1.3-3.7) | (-3-3.9) | (2.8-4.7) | (5.2-7) | (-1.7-4.5) |
| Libya | 4.1 | 5.0 | 3.4 | 3.9 | 4.7 | 3.2 | 3.5 | 5.0 | 3.4 | 4.7 | 5.0 | 3.4 |
| | (2.5-5.6) | (2.9-7.1) | (-1.4-8.3) | (2.4-5.4) | (2.7-6.9) | (-1.6-8.1) | (1.3-5.7) | (2.7-7.4) | (-1.6-8.5) | (2.5-7.2) | (2.7-7.5) | (-1.6-8.6) |
| Lithuania | 3.4 | 5.0 | 4.4 | 3.2 | 4.9 | 4.2 | 5.4 | 4.3 | 2.7 | 1.6 | 5.7 | 6.2 |
| | (2.7-3.9) | (4.5-5.6) | (2.5-6.3) | (2.6-3.8) | (4.3-5.4) | (2.3-6.1) | (4.4-6.3) | (3.4-5.1) | (0.2-5.2) | (0.7-2.5) | (4.9-6.5) | (3.7-8.9) |
| Luxembourg | 6.2 | 4.1 | 1.7 | 6.3 | 4.0 | 1.5 | 5.6 | 3.1 | 0.3 | 6.9 | 5.4 | 4.0 |
| | (5.1-7.4) | (3.1-5.1) | (-1.4-4.6) | (5.1-7.5) | (3-5) | (-1.5-4.4) | (3.7-7.6) | (1.6-4.6) | (-3.3-4) | (4.9-8.8) | (3.7-7) | (-0.5-9.3) |
| Madagascar | 3.9 | 3.1 | 0.5 | 3.3 | 2.7 | 0.2 | 2.1 | 1.8 | 0.3 | 4.7 | 3.8 | 0.6 |
| | (3.1-4.7) | (2.4-3.9) | (-2.5-3.2) | (2.5-4.1) | (2-3.4) | (-2.8-2.9) | (1-3.3) | (0.8-2.7) | (-2.9-3.2) | (3.8-5.5) | (3.1-4.7) | (-2.4-3.5) |
| Malawi | 3.5 | 7.3 | 1.8 | 3.0 | 6.1 | 0.5 | 2.7 | 3.0 | 0.2 | 3.9 | 9.3 | 3.1 |
| | (3-4) | (6.5-8.1) | (-1.5-5) | (2.5-3.5) | (5.3-6.9) | (-2.8-3.7) | (1.7-3.8) | (1.9-4.1) | (-3.3-3.6) | (3.3-4.5) | (8.5-10.2) | (-0.4-6.6) |
| Malaysia | 5.2 | 1.3 | -0.2 | 5.0 | 0.8 | -0.5 | 4.3 | 1.1 | -0.2 | 6.0 | 1.5 | -0.2 |
| | (4.9-5.5) | (1.1-1.5) | (-1.4-1) | (4.6-5.3) | (0.6-1) | (-1.7-0.7) | (2.5-5.9) | (0-2.2) | (-1.6-1.1) | (4.5-7.6) | (0.3-2.6) | (-1.6-1.2) |
| Maldives | 7.9 | 9.2 | 6.6 | 7.7 | 9.2 | 6.5 | 6.6 | 8.9 | 5.2 | 9.5 | 9.9 | 9.1 |
| | (6.9-8.9) | (8.4-10.1) | (3.9-9.3) | (6.7-8.6) | (8.4-10) | (3.8-9.3) | (4.5-8.8) | (7.6-10.1) | (1.8-8.6) | (7.1-11.8) | (8.4-11.4) | (4.7-14.4) |
| Mali | 2.1 | 3.7 | 4.5 | 1.9 | 3.0 | 3.7 | 2.7 | 2.5 | 2.6 | 2.0 | 4.3 | 5.7 |
| | (1.5-2.6) | (3.1-4.2) | (3-6.2) | (1.4-2.4) | (2.4-3.5) | (2.2-5.4) | (1.7-3.7) | (1.6-3.4) | (0.4-4.9) | (1.4-2.6) | (3.6-4.9) | (3.9-7.7) |
| Malta | 4.0 | 1.0 | 2.4 | 4.0 | 1.0 | 2.3 | 4.0 | 0.9 | 2.4 | 3.9 | 1.0 | 2.3 |
| | (2.9-5.1) | (0.1-1.9) | (-0.4-5.3) | (2.9-5.1) | (0.1-1.9) | (-0.5-5.2) | (2.8-5.3) | (-0.1-1.9) | (-0.5-5.4) | (2.3-5.6) | (-0.2-2.3) | (-1-5.7) |
| Marshall Islands | 1.1 | 1.3 | 3.0 | 0.9 | 1.1 | 2.9 | -0.4 | 1.0 | 3.2 | 2.2 | 1.6 | 3.0 |
| | (-0.6-2.9) | (-1.2-3.8) | (-1.5-7.8) | (-0.8-2.6) | (-1.5-3.6) | (-1.7-7.6) | (-3.5-2.7) | (-2.1-4.3) | (-2-8.4) | (-0.3-4.7) | (-1.5-4.8) | (-2-8.4) |
| Mauritania | 1.7 | 4.6 | 3.2 | 0.9 | 3.7 | 2.7 | 1.2 | 2.8 | 2.3 | 2.1 | 6.2 | 4.3 |
| | (0.5-2.9) | (3.7-5.5) | (-0.5-6.8) | (-0.3-2.1) | (2.9-4.6) | (-1-6.3) | (-0.2-2.7) | (1.7-3.9) | (-1.5-6) | (0.7-3.4) | (5.2-7.3) | (0.4-8.2) |
| Mauritius | 2.1 | 1.7 | -0.6 | 1.9 | 1.7 | -0.8 | 1.7 | 2.1 | -0.6 | 2.8 | 1.1 | -0.6 |
| | (1.4-2.7) | (1.2-2.3) | (-2-0.7) | (1.2-2.5) | (1.1-2.2) | (-2.2-0.5) | (0.9-2.5) | (1.4-2.7) | (-2.3-1) | (1.7-3.8) | (0.2-1.9) | (-2.5-1.3) |
| Mexico | 4.9 | 3.4 | 2.7 | 4.7 | 3.1 | 2.4 | 4.6 | 3.3 | 0.3 | 5.3 | 3.4 | 5.8 |
| | (4.2-5.6) | (2.8-3.9) | (0.7-4.7) | (4-5.4) | (2.6-3.7) | (0.4-4.5) | (3.1-6.1) | (2.5-4.1) | (-2.7-4.1) | (3.9-6.8) | (2.6-4.2) | (1.5-11.2) |

| | Annual rate of reduction in under-five mortality rate (%) | | | Annual rate of reduction in infant mortality rate (%) | | | Annual rate of reduction in neonatal mortality rate (%) | | | Annual rate of reduction in mortality rate age 1–59 months (%) | | |
|---|---|---|---|---|---|---|---|---|---|---|---|---|
| **Country** | **1990-2000** | **2000-2015** | **2015-2024** | **1990-2000** | **2000-2015** | **2015-2024** | **1990-2000** | **2000-2015** | **2015-2024** | **1990-2000** | **2000-2015** | **2015-2024** |
| Micronesia (Federated States of) | 2.4 | 1.7 | 3.2 | 2.2 | 1.5 | 3.0 | 1.7 | 1.8 | 3.3 | 3.3 | 1.7 | 3.1 |
| | (0.5-4.4) | (-1.7-5.2) | (-1.8-8) | (0.3-4.1) | (-1.9-5) | (-1.9-7.9) | (-1-4.6) | (-2.1-5.7) | (-2.2-8.6) | (0.5-6.7) | (-2-6) | (-2.2-8.9) |
| Monaco | 4.1 | 2.5 | 3.1 | 4.1 | 2.5 | 2.9 | 4.1 | 2.6 | 3.3 | 4.0 | 2.5 | 2.9 |
| | (2.8-5.3) | (1-4.1) | (-1.4-7.7) | (2.8-5.3) | (0.9-4.1) | (-1.6-7.5) | (1.9-6.6) | (0.4-5) | (-1.9-8.3) | (1.6-6.9) | (0.2-5.3) | (-2-8.4) |
| Mongolia | 5.3 | 7.8 | 4.1 | 4.4 | 7.3 | 3.7 | 2.3 | 5.9 | 3.2 | 6.8 | 9.3 | 5.1 |
| | (4.4-6.2) | (7.2-8.4) | (3.4-4.8) | (3.5-5.4) | (6.8-7.9) | (3-4.5) | (0.3-4.4) | (4.9-7) | (2.1-4.3) | (5.5-8) | (8.5-10.1) | (3.9-6.2) |
| Montenegro | 1.5 | 8.1 | 6.3 | 1.6 | 8.5 | 6.8 | 2.6 | 8.6 | 11.3 | -0.3 | 7.6 | 2.1 |
| | (0.8-2.2) | (7.4-8.9) | (3.9-8.6) | (0.8-2.3) | (7.8-9.2) | (4.4-9.1) | (1.1-4.2) | (7.3-9.9) | (6.5-16.8) | (-2.8-2) | (6-9.2) | (-1.6-6.1) |
| Montserrat | 3.8 | 3.3 | 3.2 | 3.5 | 3.2 | 3.1 | 4.0 | 3.4 | 3.3 | 3.7 | 3.4 | 3.0 |
| | (-0.1-7.7) | (-0.6-7.2) | (-1.6-8.3) | (-0.4-7.4) | (-0.7-7.1) | (-1.7-8.2) | (-0.5-8.4) | (-0.8-7.7) | (-1.9-8.8) | (-0.6-8.7) | (-0.9-7.8) | (-2.2-9) |
| Morocco | 4.4 | 5.5 | 4.3 | 3.7 | 5.0 | 4.1 | 2.6 | 4.6 | 4.3 | 6.2 | 6.7 | 4.3 |
| | (3.4-5.2) | (4-7.1) | (0.6-8.1) | (2.7-4.5) | (3.6-6.7) | (0.4-7.9) | (1.4-3.8) | (3.1-6.4) | (0.5-8.2) | (5-7.4) | (5-8.6) | (0.4-8.4) |
| Mozambique | 3.6 | 5.2 | 2.7 | 3.3 | 4.6 | 2.3 | 3.1 | 3.4 | 0.6 | 3.9 | 6.1 | 4.0 |
| | (2.9-4.2) | (4.5-5.9) | (0.2-5.2) | (2.6-3.9) | (3.9-5.3) | (-0.2-4.8) | (1.9-4.3) | (2.4-4.5) | (-2.5-3.7) | (3.2-4.6) | (5.3-6.9) | (1.2-6.9) |
| Myanmar | 2.5 | 3.8 | 3.5 | 2.0 | 3.4 | 3.3 | 2.3 | 2.7 | 2.4 | 2.8 | 4.7 | 4.6 |
| | (1.6-3.5) | (2.3-5.5) | (-0.8-7.8) | (1.1-3) | (2-5.1) | (-1-7.7) | (0.5-4.5) | (0.9-4.9) | (-2.1-7.1) | (1.1-4.2) | (2.9-6.8) | (0-9.4) |
| Namibia | -0.6 | 2.7 | 3.0 | 1.6 | 0.4 | 2.4 | 1.7 | -0.2 | 1.6 | -1.8 | 4.6 | 4.6 |
| | (-2-0.8) | (1.2-4.6) | (-1.9-6.9) | (0.2-3) | (-1.1-2.2) | (-2.5-6.4) | (0-3.3) | (-2.1-2) | (-3.5-6) | (-3.3--0.2) | (2.8-6.8) | (-0.9-9.2) |
| Nauru | 4.9 | 5.7 | 8.3 | 4.5 | 5.6 | 8.2 | 2.8 | 6.3 | 8.2 | 7.2 | 5.0 | 8.5 |
| | (2.6-7) | (3.7-7.7) | (3.4-12.8) | (2.3-6.7) | (3.6-7.6) | (3.3-12.7) | (-1.2-6.2) | (3.9-9.1) | (2.1-14) | (3.5-11.1) | (2-7.8) | (2.7-14.9) |
| Nepal | 5.6 | 4.9 | 4.5 | 5.0 | 4.3 | 4.2 | 3.8 | 3.7 | 4.5 | 7.4 | 6.5 | 4.7 |
| | (5-6.2) | (4.2-5.5) | (2.1-7) | (4.4-5.6) | (3.7-5) | (1.8-6.6) | (3-4.6) | (2.9-4.4) | (2-7) | (6.7-8.2) | (5.7-7.4) | (2.1-7.3) |
| Netherlands (Kingdom of the) | 3.0 | 2.8 | 0.5 | 2.8 | 2.5 | 0.3 | 1.9 | 2.4 | 0.1 | 4.5 | 3.4 | 1.4 |
| | (2.7-3.2) | (2.6-3) | (0-1.1) | (2.5-3.1) | (2.3-2.8) | (-0.3-0.9) | (1.5-2.3) | (2.1-2.7) | (-0.6-0.8) | (4-5) | (3-3.8) | (0.3-2.4) |
| New Zealand | 4.1 | 1.8 | 1.9 | 4.1 | 1.7 | 1.8 | 2.2 | 0.5 | 2.1 | 5.6 | 3.2 | 1.7 |
| | (3.7-4.5) | (1.5-2.1) | (0.1-3.7) | (3.7-4.5) | (1.4-2) | (-0.1-3.6) | (1.4-3) | (0-1) | (-0.5-4.9) | (5-6.2) | (2.7-3.7) | (-1.4-5) |
| Nicaragua | 6.1 | 5.1 | 4.7 | 5.9 | 4.9 | 5.2 | 3.6 | 3.0 | 7.6 | 7.8 | 7.6 | 1.4 |
| | (5-7.3) | (4.3-6) | (3.9-5.6) | (4.7-7) | (4.1-5.8) | (4.4-6.1) | (1.8-5.4) | (1.9-4) | (6.6-8.6) | (6.4-9.3) | (6.5-8.7) | (0.3-2.4) |
| Niger | 3.8 | 4.0 | 1.4 | 3.1 | 3.0 | 0.6 | 2.3 | 1.6 | 0.6 | 4.2 | 4.8 | 1.7 |
| | (3.1-4.4) | (3.2-4.8) | (-1.5-4.3) | (2.4-3.8) | (2.2-3.8) | (-2.3-3.5) | (1-3.6) | (0.2-3) | (-2.8-4) | (3.5-4.9) | (3.9-5.7) | (-1.3-4.9) |
| Nigeria | 1.6 | 2.7 | 0.2 | 1.0 | 2.2 | -0.3 | 0.7 | 1.3 | -0.3 | 1.9 | 3.4 | 0.4 |
| | (1-2.1) | (2.3-3.2) | (-1.4-1.8) | (0.5-1.5) | (1.8-2.7) | (-1.9-1.3) | (-0.3-1.6) | (0.6-2) | (-2.5-1.8) | (1.3-2.5) | (2.8-3.9) | (-1.3-2.3) |
| Niue | -2.6 | 0.3 | 3.1 | -2.9 | 0.2 | 3.0 | -2.5 | 0.4 | 3.2 | -2.7 | 0.2 | 3.1 |
| | (-5--0.1) | (-3.5-4) | (-1.8-8) | (-5.3--0.4) | (-3.6-3.9) | (-1.8-7.9) | (-5.6-0.7) | (-3.7-4.5) | (-2.1-8.6) | (-5.7-1) | (-3.9-4.7) | (-2.2-8.8) |
| North Macedonia | 8.2 | 2.4 | 15.3 | 8.4 | 2.5 | 15.4 | 6.2 | 0.9 | 22.6 | 10.5 | 5.3 | 6.3 |
| | (7.7-8.8) | (1.9-2.9) | (13.2-17.5) | (7.8-8.9) | (2-2.9) | (13.3-17.6) | (5.5-7) | (0.3-1.5) | (19.6-25.9) | (9.6-11.3) | (4.5-6.2) | (3.4-9.3) |
| Norway | 5.8 | 3.9 | 0.9 | 5.8 | 3.9 | 0.8 | 3.9 | 3.5 | 1.2 | 7.7 | 4.5 | 0.4 |

| Country | Annual rate of reduction in under-five mortality rate (%) | | | Annual rate of reduction in infant mortality rate (%) | | | Annual rate of reduction in neonatal mortality rate (%) | | | Annual rate of reduction in mortality rate age 1–59 months (%) | | |
|---|---|---|---|---|---|---|---|---|---|---|---|---|
| | 1990-2000 | 2000-2015 | 2015-2024 | 1990-2000 | 2000-2015 | 2015-2024 | 1990-2000 | 2000-2015 | 2015-2024 | 1990-2000 | 2000-2015 | 2015-2024 |
| | (5.2-6.3) | (3.5-4.4) | (-0.4-2.2) | (5.2-6.3) | (3.4-4.3) | (-0.5-2.1) | (3.1-4.7) | (2.9-4.1) | (-0.3-2.8) | (6.9-8.5) | (3.8-5.2) | (-1.4-2.2) |
| Oman | 8.7 | 2.7 | 0.8 | 8.5 | 2.6 | 0.9 | 8.2 | 2.2 | -0.5 | 9.2 | 3.1 | 2.3 |
| | (7.2-10.1) | (1.7-3.6) | (-0.4-2.1) | (7.1-9.9) | (1.7-3.6) | (-0.3-2.1) | (5.3-11.4) | (0.2-4) | (-2.1-1.1) | (6.6-11.9) | (1.2-4.8) | (0.6-4.2) |
| Pakistan | 2.6 | 2.4 | 3.2 | 2.4 | 2.2 | 3.1 | 1.3 | 1.6 | 2.4 | 4.0 | 3.6 | 4.7 |
| | (2.2-3.1) | (1.9-3) | (1-5.6) | (1.9-2.9) | (1.7-2.8) | (0.9-5.5) | (0.5-2.1) | (0.9-2.3) | (0.1-4.8) | (3.3-4.8) | (2.8-4.4) | (2.2-7.5) |
| Palau | 2.9 | 1.5 | -0.1 | 2.9 | 1.4 | -0.2 | 2.9 | 1.6 | 0.0 | 3.0 | 1.3 | -0.2 |
| | (1-4.8) | (-0.7-4.9) | (-3.9-4.9) | (1-4.8) | (-0.8-4.8) | (-4-4.8) | (0.4-5.8) | (-1.1-5.7) | (-4.4-5.6) | (0.2-6.2) | (-1.3-5.8) | (-4.5-5.6) |
| Panama | 1.7 | 2.7 | 1.3 | 1.6 | 2.7 | 1.4 | 2.4 | 3.6 | 3.5 | 1.0 | 2.0 | -0.1 |
| | (0.4-2.9) | (1.8-3.5) | (0.6-2) | (0.3-2.8) | (1.8-3.5) | (0.7-2.2) | (-2.8-7.6) | (-0.2-6.8) | (0.9-6.4) | (-4.6-6.4) | (-1.8-4.7) | (-1.8-1.6) |
| Papua New Guinea | 1.7 | 1.5 | 4.2 | 1.5 | 1.7 | 3.7 | 0.4 | 1.5 | 2.1 | 2.5 | 1.5 | 6.0 |
| | (0.6-2.6) | (0.1-3.1) | (0.1-8.3) | (0.5-2.5) | (0.3-3.3) | (-0.4-7.8) | (-1.4-1.9) | (-0.2-3.5) | (-2.2-6.4) | (1.2-3.8) | (-0.1-3.3) | (1.6-10.7) |
| Paraguay | 3.0 | 2.9 | 3.3 | 2.8 | 2.6 | 3.2 | 2.1 | 2.9 | 3.5 | 4.1 | 2.9 | 3.1 |
| | (1.4-4.6) | (-0.3-5.9) | (-1.6-8.2) | (1.1-4.4) | (-0.5-5.7) | (-1.8-8.1) | (-0.2-4.2) | (-0.6-6.3) | (-1.8-8.4) | (2-6.4) | (-0.6-6.5) | (-2-8.7) |
| Peru | 7.4 | 5.6 | 2.8 | 6.9 | 5.2 | 2.5 | 5.5 | 4.5 | 2.5 | 8.8 | 6.5 | 3.1 |
| | (6.8-8.1) | (4.9-6.2) | (-0.3-5.6) | (6.3-7.5) | (4.5-5.9) | (-0.6-5.3) | (4.6-6.5) | (3.7-5.4) | (-0.8-5.6) | (8-9.5) | (5.7-7.3) | (-0.3-6.3) |
| Philippines | 4.2 | 1.9 | 0.6 | 3.5 | 1.4 | 0.2 | 1.4 | 1.4 | -0.1 | 6.0 | 2.3 | 1.2 |
| | (3.4-4.9) | (1-2.7) | (-2.7-3.6) | (2.8-4.3) | (0.6-2.2) | (-3-3.3) | (0.2-2.5) | (0.4-2.4) | (-3.7-3.4) | (5.1-7) | (1.4-3.3) | (-2.4-4.8) |
| Poland | 6.2 | 4.3 | 1.6 | 6.3 | 4.4 | 1.7 | 6.6 | 4.6 | 1.8 | 5.7 | 3.9 | 1.4 |
| | (5.9-6.5) | (4.1-4.5) | (1.1-2.1) | (6-6.6) | (4.2-4.6) | (1.2-2.2) | (6.2-6.9) | (4.3-4.8) | (1.2-2.4) | (5.3-6.1) | (3.6-4.2) | (0.6-2.1) |
| Portugal | 7.2 | 4.5 | 1.3 | 7.4 | 4.2 | 1.1 | 7.7 | 3.1 | 2.4 | 6.8 | 6.1 | 0.0 |
| | (6.9-7.6) | (4.2-4.9) | (0.4-2.2) | (7.1-7.8) | (3.8-4.5) | (0.2-2) | (7.1-8.3) | (2.6-3.6) | (1.1-3.6) | (6.2-7.3) | (5.6-6.7) | (-1.4-1.4) |
| Qatar | 5.1 | 3.1 | 3.1 | 5.1 | 3.2 | 3.2 | 5.6 | 3.6 | 0.0 | 4.7 | 2.7 | 7.2 |
| | (4.3-6) | (2.7-3.6) | (2-4.2) | (4.3-6) | (2.8-3.7) | (2.1-4.2) | (2.8-8.1) | (2.8-4.5) | (-1.6-1.6) | (1.4-7.5) | (1.8-3.5) | (5.4-9.1) |
| Republic of Korea | 7.3 | 5.1 | 2.5 | 7.3 | 4.7 | 2.3 | 7.8 | 4.9 | 3.7 | 7.0 | 5.3 | 1.6 |
| | (6.7-7.9) | (4.9-5.4) | (1.8-3.3) | (6.6-7.9) | (4.5-5) | (1.5-3) | (6-9.6) | (4.4-5.5) | (2.5-5.1) | (5.1-8.5) | (4.8-5.7) | (0.5-2.7) |
| Republic of Moldova | 0.7 | 4.7 | -0.6 | 0.4 | 4.5 | -0.7 | -1.0 | 3.8 | -0.6 | 3.2 | 6.9 | -0.6 |
| | (-1.2-2.6) | (2.7-6.6) | (-4.9-2.8) | (-1.5-2.4) | (2.6-6.4) | (-5-2.7) | (-3.2-1.2) | (1.7-5.8) | (-5-3.1) | (0.7-5.9) | (4-9.8) | (-5.8-4.2) |
| Romania | 3.7 | 5.7 | 2.5 | 3.0 | 5.7 | 2.7 | 3.9 | 5.8 | 1.9 | 3.5 | 5.6 | 3.1 |
| | (3.3-4) | (5.4-5.9) | (1.9-3.1) | (2.6-3.4) | (5.4-5.9) | (2.1-3.3) | (1.9-5.9) | (4.8-6.8) | (0.7-3) | (1.4-5.2) | (4.7-6.5) | (2-4.1) |
| Russian Federation | 1.1 | 5.7 | 5.1 | 1.1 | 5.7 | 5.2 | 1.9 | 5.0 | 8.3 | 0.4 | 6.4 | 2.5 |
| | (0.8-1.4) | (5.5-5.9) | (4.5-5.7) | (0.9-1.4) | (5.5-5.9) | (4.6-5.8) | (-1.8-5.2) | (4.5-5.6) | (7-9.7) | (-3.5-3.4) | (5.9-7) | (1.4-3.7) |
| Rwanda | -2.0 | 9.1 | 2.5 | -2.2 | 7.7 | 1.6 | -0.5 | 5.2 | 1.5 | -2.5 | 11.1 | 3.3 |
| | (-2.6--1.4) | (7.9-10.3) | (-1.9-7) | (-2.8--1.6) | (6.6-8.9) | (-2.9-6) | (-1.6-0.6) | (3.7-6.6) | (-3-6.2) | (-3.2--1.8) | (9.8-12.4) | (-1.3-8.1) |
| Saint Kitts and Nevis | 2.4 | 1.4 | 2.3 | 2.2 | 1.2 | 2.1 | 2.1 | 1.4 | 2.3 | 2.9 | 1.3 | 2.3 |
| | (1-3.7) | (-0.2-3) | (-2-6.6) | (0.9-3.6) | (-0.4-2.8) | (-2.1-6.4) | (0.3-3.9) | (-0.4-3.3) | (-2.3-6.9) | (0.5-5.4) | (-1.1-3.8) | (-2.7-7.8) |
| Saint Lucia | 1.2 | -0.1 | 0.9 | 0.8 | -0.4 | 0.7 | 0.5 | -1.1 | 2.0 | 2.2 | 2.0 | -1.4 |
| | (0.6-1.8) | (-0.6-0.5) | (-0.6-2.4) | (0.2-1.4) | (-0.9-0.2) | (-0.8-2.2) | (-0.6-1.6) | (-1.9--0.3) | (0.1-3.9) | (0.8-3.8) | (0.7-3.2) | (-4.2-1.6) |

| | Annual rate of reduction in under-five mortality rate (%) | | | Annual rate of reduction in infant mortality rate (%) | | | Annual rate of reduction in neonatal mortality rate (%) | | | Annual rate of reduction in mortality rate age 1–59 months (%) | | |
|---|---|---|---|---|---|---|---|---|---|---|---|---|
| Country | 1990-2000 | 2000-2015 | 2015-2024 | 1990-2000 | 2000-2015 | 2015-2024 | 1990-2000 | 2000-2015 | 2015-2024 | 1990-2000 | 2000-2015 | 2015-2024 |
| Saint Vincent and the Grenadines | 0.5 | 1.7 | 4.8 | 0.1 | 1.5 | 4.6 | -0.6 | 1.2 | 2.5 | 1.9 | 2.6 | 10.9 |
| | (-0.5-1.5) | (0.9-2.6) | (2.2-7.3) | (-0.9-1.1) | (0.6-2.3) | (2-7.1) | (-2.1-0.9) | (0.1-2.3) | (-0.3-5.2) | (0.2-3.7) | (1.3-4.1) | (7.2-14.8) |
| Samoa | 3.5 | 0.9 | 2.3 | 3.4 | 0.7 | 2.1 | 4.0 | 1.2 | 2.4 | 3.1 | 0.8 | 2.2 |
| | (2.1-4.8) | (-0.5-2.4) | (-1.8-6.2) | (2-4.7) | (-0.8-2.1) | (-2.1-6) | (1-8) | (-1.1-3.7) | (-2.6-7.4) | (-0.4-5.5) | (-1.3-2.7) | (-2.2-6.7) |
| San Marino | 8.8 | 6.8 | 4.6 | 8.1 | 6.6 | 4.5 | 9.7 | 7.4 | 4.9 | 8.0 | 6.2 | 4.4 |
| | (3.6-13.9) | (3.2-10.3) | (-0.1-9.3) | (2.9-13.2) | (3-10.2) | (-0.2-9.2) | (3.1-16.9) | (2.6-14.5) | (-1.8-12.5) | (-0.1-14.5) | (1.5-11.2) | (-0.8-11.2) |
| Sao Tome and Principe | 2.8 | 8.4 | 6.0 | 2.6 | 8.0 | 5.7 | 2.0 | 4.7 | 6.0 | 3.1 | 10.6 | 6.1 |
| | (1.4-4.2) | (6-10.9) | (1.7-10.4) | (1.1-4) | (5.6-10.5) | (1.3-10) | (-1-5.5) | (1.4-7.7) | (0.9-11.2) | (1.3-4.8) | (7.9-13.6) | (1.3-11.4) |
| Saudi Arabia | 7.0 | 5.9 | 4.6 | 7.2 | 6.6 | 5.0 | 6.4 | 6.2 | 5.1 | 7.7 | 5.6 | 4.2 |
| | (5.4-8.8) | (4.6-7.1) | (0.9-8.3) | (5.6-9) | (5.4-7.9) | (1.3-8.7) | (2.6-10.3) | (3.9-8.7) | (0.4-10.5) | (3.7-11.5) | (3-8) | (-0.6-9) |
| Senegal | 0.6 | 6.0 | 4.0 | -0.1 | 4.6 | 2.8 | 0.6 | 2.6 | 2.1 | 0.7 | 8.2 | 6.1 |
| | (0-1.2) | (5.4-6.6) | (1.4-6.7) | (-0.8-0.5) | (4-5.1) | (0.2-5.5) | (-0.5-1.6) | (1.8-3.4) | (-0.7-5) | (-0.1-1.4) | (7.5-8.9) | (3.1-9.4) |
| Serbia | 7.9 | 4.7 | 1.7 | 7.9 | 4.7 | 1.8 | 7.9 | 4.4 | 3.2 | 8.1 | 5.1 | -0.5 |
| | (7.5-8.3) | (4.3-5) | (0.7-2.6) | (7.4-8.3) | (4.4-5.1) | (0.9-2.8) | (5.1-9.9) | (3.8-5) | (1.8-4.7) | (4.6-11.6) | (4.3-5.9) | (-2.3-1.2) |
| Seychelles | 1.7 | -0.4 | 0.5 | 1.1 | -0.7 | 0.3 | 2.3 | -0.1 | 0.7 | 0.6 | -0.8 | 0.2 |
| | (0.4-3) | (-1.4-0.6) | (-2.7-3.7) | (-0.2-2.4) | (-1.7-0.3) | (-2.9-3.5) | (0.6-4) | (-1.3-1.2) | (-2.9-4.4) | (-2-2.8) | (-2.5-0.8) | (-3.7-4.3) |
| Sierra Leone | 1.5 | 3.3 | 4.6 | 0.9 | 3.1 | 3.8 | 0.8 | 2.3 | 2.1 | 1.7 | 3.6 | 5.6 |
| | (0.7-2.2) | (2.6-3.9) | (1.7-7.3) | (0.1-1.7) | (2.5-3.8) | (1-6.6) | (-0.8-2.4) | (1.2-3.6) | (-1.2-5.4) | (0.8-2.5) | (2.9-4.4) | (2.6-8.6) |
| Singapore | 6.9 | 2.4 | 0.0 | 7.1 | 2.2 | -0.3 | 9.2 | 2.7 | 0.0 | 4.8 | 2.2 | 0.0 |
| | (6.2-7.6) | (1.8-3) | (-1.7-1.6) | (6.4-7.8) | (1.6-2.8) | (-2-1.4) | (8.2-10.3) | (1.8-3.6) | (-2.4-2.2) | (3.8-5.8) | (1.5-3) | (-1.9-2) |
| Slovakia | 4.3 | 2.9 | -0.1 | 4.5 | 3.0 | -0.3 | 5.5 | 3.3 | -0.8 | 2.7 | 2.6 | 0.5 |
| | (3.9-4.6) | (2.6-3.2) | (-1-0.7) | (4.1-4.9) | (2.6-3.3) | (-1.2-0.5) | (4.9-6.1) | (2.8-3.8) | (-2-0.4) | (2.1-3.4) | (2.1-3.1) | (-0.7-1.7) |
| Slovenia | 6.4 | 4.9 | 1.6 | 6.6 | 5.0 | 1.7 | 5.8 | 5.0 | 1.9 | 7.3 | 4.7 | 1.3 |
| | (5.8-7.1) | (4.3-5.5) | (-0.5-3.5) | (6-7.3) | (4.4-5.7) | (-0.4-3.5) | (4.8-6.8) | (4.2-5.9) | (-0.6-4.1) | (6.1-8.6) | (3.7-5.7) | (-1.3-3.8) |
| Solomon Islands | 2.2 | 1.3 | 2.5 | 2.0 | 1.1 | 2.2 | 1.5 | 1.6 | 2.6 | 2.7 | 1.2 | 2.4 |
| | (1-3.4) | (-0.1-2.8) | (-1.1-6) | (0.8-3.2) | (-0.3-2.5) | (-1.4-5.8) | (-1.6-3.9) | (-0.5-4) | (-1.6-6.6) | (0.8-4.9) | (-0.7-3.1) | (-1.3-6.4) |
| Somalia | 0.4 | 1.7 | 3.2 | 0.2 | 1.4 | 2.7 | 0.2 | 0.7 | 1.8 | 0.5 | 2.1 | 3.9 |
| | (-0.7-1.4) | (-1.2-4.5) | (-1.8-8.2) | (-0.8-1.2) | (-1.4-4.2) | (-2.2-7.8) | (-2.8-3.2) | (-3-4.4) | (-3.5-7.3) | (-0.9-1.9) | (-1.1-5.2) | (-1.4-9.3) |
| South Africa | -2.1 | 4.2 | 1.0 | 1.1 | 3.1 | 2.1 | 4.2 | 1.0 | -0.7 | -4.3 | 5.2 | 1.8 |
| | (-3.5--0.5) | (3.5-4.8) | (-1.1-2.9) | (-0.3-2.7) | (2.4-3.7) | (0.1-4) | (1.2-6.9) | (-0.7-3) | (-3.7-2.1) | (-6--2.5) | (4.3-6) | (-0.6-4) |
| South Sudan | 5.1 | -1.9 | 10.1 | 2.9 | -1.6 | 8.3 | 1.4 | 1.9 | 0.6 | 6.5 | -3.0 | 13.9 |
| | (3.4-6.8) | (-5.5-2.6) | (3-18.7) | (1.1-4.5) | (-5.2-2.9) | (1.3-16.8) | (-3.9-5.9) | (-2.8-6.9) | (-6.9-10.1) | (4.3-8.8) | (-7-1.9) | (6.1-23.4) |
| Spain | 5.2 | 3.3 | 0.4 | 5.4 | 3.1 | 0.5 | 5.6 | 2.4 | 1.3 | 4.9 | 4.4 | -0.8 |
| | (5-5.5) | (3.1-3.5) | (-0.3-1.1) | (5.1-5.6) | (2.9-3.3) | (-0.2-1.2) | (5.2-6) | (2.1-2.7) | (0.2-2.4) | (4.4-5.3) | (4.1-4.7) | (-1.9-0.5) |
| Sri Lanka | 3.6 | 4.1 | 4.4 | 3.2 | 3.8 | 3.9 | 3.8 | 4.0 | 3.3 | 3.3 | 4.4 | 6.3 |
| | (3.3-3.9) | (3.9-4.4) | (2.4-6.4) | (2.9-3.5) | (3.6-4) | (1.9-6) | (3.4-4.3) | (3.7-4.2) | (1.1-5.6) | (2.6-3.9) | (4.1-4.8) | (3.4-9.5) |
| State of Palestine | 4.0 | 3.4 | -8.2 | 4.0 | 3.4 | -0.8 | 2.7 | 2.6 | 2.9 | 5.4 | 4.6 | -16.3 |

| Country | Annual rate of reduction in under-five mortality rate (%) | | | Annual rate of reduction in infant mortality rate (%) | | | Annual rate of reduction in neonatal mortality rate (%) | | | Annual rate of reduction in mortality rate age 1–59 months (%) | | |
|---|---|---|---|---|---|---|---|---|---|---|---|---|
| | 1990-2000 | 2000-2015 | 2015-2024 | 1990-2000 | 2000-2015 | 2015-2024 | 1990-2000 | 2000-2015 | 2015-2024 | 1990-2000 | 2000-2015 | 2015-2024 |
| | (3.1-4.7) | (2.1-4.6) | (-11.5--4.7) | (3.1-4.7) | (2.1-4.6) | (-4.2-2.6) | (1.4-4) | (1.1-4) | (-0.5-6.3) | (4-6.8) | (2.9-6.4) | (-20.1--12.6) |
| Sudan | 2.5 | 3.2 | 0.3 | 1.8 | 2.5 | 1.3 | 1.5 | 1.7 | 1.9 | 3.0 | 4.2 | -0.7 |
| | (1.8-3.2) | (2-4.3) | (-3.8-4.5) | (1.1-2.6) | (1.4-3.6) | (-2.8-5.6) | (0.3-2.9) | (0.2-3.1) | (-2.3-6.2) | (2.1-3.9) | (2.9-5.6) | (-5.1-3.6) |
| Suriname | 3.7 | 2.7 | 2.9 | 3.1 | 2.4 | 2.8 | 1.9 | 1.8 | 3.2 | 5.5 | 4.1 | 2.5 |
| | (2-5.5) | (0.4-4.9) | (-1.2-6.9) | (1.5-4.9) | (0.1-4.6) | (-1.4-6.8) | (-3.5-5.7) | (-1.2-4.5) | (-2.1-9.2) | (1.7-9.6) | (0.8-7.8) | (-3.8-11) |
| Sweden | 5.3 | 2.4 | 1.9 | 5.5 | 2.5 | 2.0 | 4.2 | 2.6 | 1.1 | 6.6 | 2.1 | 3.1 |
| | (4.8-5.8) | (2-2.7) | (1-2.9) | (5-5.9) | (2.1-2.8) | (1-2.9) | (3.5-4.8) | (2.1-3.1) | (-0.1-2.3) | (5.9-7.4) | (1.5-2.7) | (1.7-4.5) |
| Switzerland | 3.7 | 1.8 | 1.1 | 3.5 | 1.5 | 0.9 | 1.2 | 1.0 | 0.7 | 6.8 | 3.4 | 2.0 |
| | (3.3-4.2) | (1.5-2.1) | (0.3-1.9) | (3-3.9) | (1.2-1.8) | (0.1-1.8) | (0.6-1.8) | (0.6-1.4) | (-0.2-1.7) | (6-7.5) | (2.8-4) | (0.6-3.6) |
| Syrian Arab Republic | 4.7 | -3.2 | 7.8 | 4.2 | -1.3 | 4.1 | 3.1 | 1.0 | 1.2 | 6.2 | -5.9 | 12.1 |
| | (3.6-5.8) | (-4.7--0.2) | (3.9-11.7) | (3.1-5.3) | (-2.9-1.6) | (0.2-8) | (1.4-4.9) | (-0.9-3.9) | (-3.2-5.8) | (4.5-7.9) | (-7.7--2.9) | (7.8-16.9) |
| Tajikistan | 2.4 | 5.9 | 1.5 | 2.2 | 5.7 | 1.3 | 1.2 | 4.6 | 1.8 | 2.9 | 6.8 | 1.3 |
| | (1.3-3.4) | (4.9-7) | (-3.2-6) | (1.2-3.2) | (4.6-6.7) | (-3.4-5.8) | (-1-3.6) | (3.2-6.2) | (-3.6-7) | (1.5-4.2) | (5.5-8) | (-3.6-6.3) |
| Thailand | 5.3 | 4.0 | 3.1 | 5.2 | 4.0 | 3.0 | 5.3 | 4.1 | 2.9 | 5.4 | 4.0 | 3.4 |
| | (4.4-6.6) | (3.1-4.9) | (0.2-4.6) | (4.3-6.5) | (3-4.8) | (0.1-4.4) | (3.5-7.7) | (2.5-5.9) | (-0.2-5.5) | (3.3-8.1) | (2.3-6.1) | (-0.4-5.7) |
| Timor-Leste | 7.2 | 4.1 | 2.6 | 6.7 | 4.4 | 2.6 | 4.0 | 3.1 | 1.6 | 8.7 | 4.9 | 3.4 |
| | (6.2-8.1) | (3-5.4) | (-1.2-5.5) | (5.8-7.7) | (3.3-5.7) | (-1.3-5.5) | (2.2-5.9) | (1-5.5) | (-2.6-5.3) | (7.6-9.9) | (3.3-6.8) | (-0.8-6.9) |
| Togo | 2.1 | 3.1 | 3.2 | 1.9 | 3.0 | 2.9 | 1.8 | 2.1 | 1.9 | 2.3 | 3.6 | 4.1 |
| | (1.4-2.8) | (2.2-3.9) | (-0.3-6.8) | (1.2-2.5) | (2.1-3.8) | (-0.6-6.5) | (0.6-3.1) | (0.8-3.4) | (-1.9-5.7) | (1.4-3.1) | (2.6-4.7) | (0.4-7.9) |
| Tonga | 2.7 | 2.4 | 2.5 | 2.6 | 2.4 | 2.4 | 3.1 | 2.2 | 2.4 | 2.4 | 2.5 | 2.5 |
| | (1-4.3) | (0.7-4.1) | (-1.1-6) | (1-4.3) | (0.7-4.1) | (-1.2-5.9) | (0.2-6.4) | (-0.1-4.4) | (-2.2-7.1) | (-0.5-4.7) | (0.5-4.7) | (-1.5-7.1) |
| Trinidad and Tobago | 1.1 | 1.6 | 2.1 | 1.1 | 1.6 | 2.1 | 0.9 | 1.6 | 2.2 | 1.5 | 1.5 | 1.9 |
| | (-0.8-2.5) | (-0.2-3.4) | (-1-5.4) | (-0.8-2.5) | (-0.2-3.5) | (-1-5.4) | (-1.1-2.6) | (-0.3-3.6) | (-1-5.7) | (-1.1-3.7) | (-0.8-3.8) | (-1.9-6) |
| Tunisia | 6.3 | 3.1 | 4.6 | 4.4 | 2.7 | 5.5 | 4.6 | 2.3 | 5.0 | 8.4 | 4.6 | 3.9 |
| | (5-7.7) | (2.2-4) | (3.7-5.5) | (3.1-5.8) | (1.8-3.5) | (4.6-6.4) | (3-6.4) | (1.2-3.3) | (3.5-6.6) | (6.5-10.3) | (3.3-5.8) | (1.4-6.3) |
| Turkmenistan | 1.2 | 3.1 | 1.1 | 1.7 | 3.5 | 1.2 | -1.3 | 1.4 | 0.7 | 2.7 | 4.7 | 1.7 |
| | (-0.2-2.6) | (1.6-4.8) | (-4.1-5.8) | (0.3-3.1) | (2-5.2) | (-4.1-5.9) | (-3.5-0.9) | (-0.5-3.4) | (-4.9-5.9) | (1-4.6) | (2.8-6.8) | (-4.1-7.4) |
| Turks and Caicos Islands | 5.7 | 4.2 | 3.2 | 6.3 | 4.7 | 3.2 | 5.7 | 4.4 | 3.3 | 5.8 | 4.1 | 3.2 |
| | (3-8.5) | (-1.4-10.2) | (-2.1-8.7) | (3.6-9.1) | (-1-10.6) | (-2.1-8.6) | (2.5-9.4) | (-1.5-10.6) | (-2.4-9.2) | (2.3-9.6) | (-1.6-10.7) | (-2.6-9.2) |
| Tuvalu | 2.3 | 3.3 | 3.4 | 2.2 | 3.2 | 3.3 | 1.5 | 4.8 | 3.6 | 3.4 | 1.8 | 3.3 |
| | (0.5-4.2) | (0.9-5.8) | (-1-7.8) | (0.3-4.1) | (0.8-5.8) | (-1.1-7.7) | (-1.2-4.1) | (2.1-7.7) | (-1.3-9) | (0.3-6.4) | (-1-4.8) | (-1.6-8.4) |
| Türkiye | 6.9 | 6.9 | 3.6 | 6.8 | 6.9 | 3.5 | 5.8 | 6.8 | 3.8 | 7.9 | 7.0 | 3.5 |
| | (6-7.8) | (6.1-7.7) | (1.5-5.5) | (5.9-7.8) | (6.1-7.7) | (1.3-5.3) | (4.5-7.1) | (5.9-7.8) | (1.6-5.7) | (6.7-9.1) | (6.1-7.9) | (1.3-5.4) |
| Uganda | 2.4 | 6.1 | 1.9 | 1.8 | 5.3 | 1.3 | 1.9 | 2.2 | 1.0 | 2.6 | 7.9 | 2.6 |
| | (1.8-2.9) | (5.4-6.8) | (-0.3-4.2) | (1.3-2.4) | (4.6-6) | (-1-3.6) | (0.7-3.1) | (1.1-3.3) | (-1.7-3.9) | (1.9-3.2) | (7-8.7) | (0.1-5.4) |
| Ukraine | 0.5 | 4.4 | 2.0 | 0.4 | 4.3 | 2.1 | 0.7 | 4.5 | 2.2 | 0.2 | 4.2 | 1.7 |
| | (-0.5-1.6) | (3.7-5.1) | (0.3-3.6) | (-0.6-1.5) | (3.7-5) | (0.4-3.7) | (-1.4-3.2) | (2.9-6.8) | (0-4.5) | (-3.3-3.3) | (1.7-7) | (-0.8-4.5) |

| Country | Annual rate of reduction in under-five mortality rate (%) | | | Annual rate of reduction in infant mortality rate (%) | | | Annual rate of reduction in neonatal mortality rate (%) | | | Annual rate of reduction in mortality rate age 1–59 months (%) | | |
|---|---|---|---|---|---|---|---|---|---|---|---|---|
| | 1990-2000 | 2000-2015 | 2015-2024 | 1990-2000 | 2000-2015 | 2015-2024 | 1990-2000 | 2000-2015 | 2015-2024 | 1990-2000 | 2000-2015 | 2015-2024 |
| United Arab Emirates | 3.4 | 2.3 | 5.2 | 3.3 | 2.2 | 5.3 | 2.9 | 2.3 | 6.3 | 4.0 | 2.3 | 4.0 |
| | (2.4-4.4) | (1.7-2.8) | (3.3-7.2) | (2.3-4.3) | (1.7-2.8) | (3.3-7.2) | (1.7-4.1) | (1.6-3) | (3.7-9.1) | (2.7-5.3) | (1.5-3) | (1.4-6.8) |
| United Kingdom | 3.5 | 2.6 | -0.5 | 3.5 | 2.4 | -0.8 | 1.7 | 2.4 | -1.5 | 5.6 | 2.8 | 1.1 |
| | (3.2-3.8) | (2.4-2.7) | (-1.2-0.1) | (3.2-3.8) | (2.2-2.6) | (-1.4--0.1) | (1.4-2) | (2.2-2.6) | (-2.3--0.8) | (5.2-5.9) | (2.6-3.1) | (0.2-2) |
| United Republic of Tanzania | 2.7 | 5.7 | 4.3 | 2.2 | 4.9 | 3.9 | 2.0 | 2.2 | 1.8 | 3.0 | 7.6 | 6.6 |
| | (2.1-3.3) | (5-6.4) | (1.6-7) | (1.6-2.8) | (4.2-5.6) | (1.3-6.6) | (0.9-3.2) | (1.1-3.2) | (-1.2-4.9) | (2.3-3.7) | (6.8-8.4) | (3.6-10.1) |
| United States | 2.9 | 1.5 | 0.5 | 2.8 | 1.4 | 0.5 | 2.2 | 1.2 | 0.7 | 3.6 | 1.9 | 0.2 |
| | (2.6-3.1) | (1.3-1.6) | (0-1) | (2.5-3) | (1.3-1.6) | (0-1) | (1.9-2.5) | (1-1.3) | (0.1-1.2) | (3.3-3.9) | (1.7-2.1) | (-0.4-0.8) |
| Uruguay | 3.3 | 4.3 | 1.9 | 3.4 | 4.5 | 2.2 | 3.3 | 3.5 | 2.3 | 3.3 | 5.3 | 1.5 |
| | (3-3.6) | (4.1-4.6) | (0.8-3.1) | (3.1-3.7) | (4.2-4.8) | (1.1-3.3) | (1.5-5) | (3-4) | (0.6-4.1) | (1.5-4.9) | (4.7-5.8) | (-0.4-3.5) |
| Uzbekistan | 1.4 | 7.5 | 4.3 | 0.9 | 7.2 | 4.2 | 0.8 | 6.3 | 4.0 | 1.9 | 8.9 | 4.8 |
| | (-0.1-2.8) | (6.6-8.5) | (2.4-5.6) | (-0.5-2.3) | (6.3-8.2) | (2.2-5.4) | (-1.1-2.6) | (5.1-7.6) | (1.7-5.9) | (0.1-3.7) | (7.7-10.2) | (2.1-7) |
| Vanuatu | 2.8 | 2.4 | 0.9 | 2.6 | 2.2 | 0.7 | 3.1 | 1.8 | 2.2 | 2.4 | 3.2 | -0.7 |
| | (1.4-4.2) | (1-3.8) | (-2.5-4.8) | (1.2-4) | (0.8-3.6) | (-2.7-4.6) | (-3-7.6) | (-0.6-4) | (-3.4-13) | (-5-8.5) | (0.6-6.7) | (-9.9-7.2) |
| Venezuela (Bolivarian Republic of) | 3.2 | 0.8 | -2.6 | 3.3 | 0.4 | -2.7 | 1.7 | -2.0 | -0.3 | 4.5 | 5.8 | -8.0 |
| | (2.8-3.5) | (0.4-1.2) | (-7.5-2.3) | (2.9-3.6) | (0-0.7) | (-7.6-2.2) | (1.2-2.2) | (-2.4--1.7) | (-4.9-4) | (4.1-5) | (5.1-6.6) | (-15.3-2.6) |
| Viet Nam | 5.3 | 2.2 | 2.5 | 5.3 | 2.4 | 2.8 | 4.4 | 2.2 | 2.5 | 6.3 | 2.4 | 2.5 |
| | (3.8-7.2) | (1-3.2) | (0.9-4.1) | (3.7-7.1) | (1.1-3.4) | (1.2-4.4) | (2.5-6.7) | (0.5-3.7) | (0.5-4.6) | (4.4-8.5) | (0.7-4) | (0.5-4.7) |
| Yemen | 3.0 | 4.2 | 2.8 | 2.5 | 4.0 | 2.3 | 1.8 | 2.8 | 1.6 | 3.8 | 5.5 | 4.2 |
| | (2.3-3.7) | (3.4-5.1) | (0.3-5.2) | (1.8-3.1) | (3.1-4.9) | (-0.2-4.7) | (0.8-2.7) | (1.8-3.8) | (-1-4.1) | (3-4.6) | (4.5-6.5) | (1.5-6.8) |
| Zambia | 1.7 | 6.2 | 2.4 | 1.4 | 5.0 | 2.3 | 0.7 | 2.3 | 1.0 | 2.0 | 7.9 | 3.5 |
| | (1.1-2.4) | (5.5-6.8) | (-0.1-4.8) | (0.7-2) | (4.3-5.6) | (-0.2-4.6) | (-0.4-1.8) | (1.4-3.2) | (-1.7-3.6) | (1.4-2.7) | (7.2-8.6) | (0.8-6.1) |
| Zimbabwe | -1.7 | 3.5 | -1.0 | -0.8 | 0.4 | -1.5 | -1.2 | -1.1 | -2.3 | -1.9 | 5.8 | 0.3 |
| | (-3--0.4) | (2.5-4.4) | (-5.2-3.4) | (-2.1-0.5) | (-0.5-1.4) | (-5.7-2.9) | (-2.8-0.4) | (-2.2--0.1) | (-6.9-2.2) | (-3.2--0.5) | (4.8-6.8) | (-4.3-5.3) |

## 3.7. Table A.7: Data series in U5MR database

Data series included in the under-five mortality rate (U5MR) database, by country.

| Country | Data series | Inclusion |
|---|---|---|
| Afghanistan | National Demographic and Family Guidance Survey 1972 (Household Deaths) | 0 |
| | National Demographic and Family Guidance Survey 1972 (Indirect) | 1 |
| | Census 1979 (Household Deaths) | 1 |
| | Multiple Indicator Cluster Survey 1997 (Indirect) | 1 |
| | Multiple Indicator Cluster Survey 2000 (Indirect) | 0 |
| | Multiple Indicator Cluster Survey 2003 (Direct) | 0 |
| | Multiple Indicator Cluster Survey 2003 (Indirect) | 0 |

| Country | Data series | Inclusion |
| --- | --- | --- |
| | Afghanistan Health Survey 2006-2007 (Indirect) | 1 |
| | National Risk and Vulnerability Assessment Survey 2007-2008 (Indirect) | 1 |
| | Afghanistan Mortality Survey (AMS) Excluding South Zone 2010 (Direct) | 0 |
| | Afghanistan Mortality Survey (AMS) Excluding South Zone (Household data (5 year)) 2010 (Household Deaths) | 1 |
| | Afghanistan Mortality Survey (AMS) Excluding South Zone (NN adjusted) 2010 (Direct) | 1 |
| | Multiple Indicator Cluster Survey 2010-2011 (Indirect) | 1 |
| | Multiple Indicator Cluster Survey Excluding South Zone 2010-2011 (Indirect) | 0 |
| | Afghanistan Living Conditions Survey 2011-2012 (Indirect) | 0 |
| | Afghanistan Health Survey 2015 2015 (Direct) | 0 |
| | Demographic and Health Survey 2015 (Direct) | 0 |
| | Demographic and Health Survey (NN adjusted) 2015 (Direct) | 1 |
| | Afghanistan Health Survey 2018 (Direct) | 0 |
| | Afghanistan Health Survey (NN adjusted) 2018 (Direct) | 1 |
| | Multiple Indicator Cluster Survey 2022-2023 (Direct) | 0 |
| | Multiple Indicator Cluster Survey (NN adjusted) 2022-2023 (Direct) | 1 |
| Angola | Census 1940 (Indirect) | 0 |
| | Multiple Indicator Cluster Survey 1996 (Indirect) | 1 |
| | Multiple Indicator Cluster Survey 2001 (Indirect) | 1 |
| | Malaria Indicator Survey 2006-2007 (Indirect) | 1 |
| | Household Incomes and Expenditures Survey IBEP 2008-2009 (Indirect) | 1 |
| | Malaria Indicator Survey 2011 (Direct) | 0 |
| | Census 2014 (Household Deaths) | 0 |
| | Census 2014 (Indirect) | 0 |
| | Demographic and Health Survey 2015-2016 (Direct) | 1 |
| | Demographic and Health Survey 2023-2024 (Direct) | 1 |
| Anguilla | UNPD Vital Registration Data 2022 version 2022 (VR) | 0 |
| | Recalculated UNPD Vital Registration Data 2022 version 2023 (VR) | 1 |
| Albania | Multiple Indicator Cluster Survey 2000 (Indirect) | 1 |
| | Reproductive Health Survey 2002 (Direct) | 1 |
| | Multiple Indicator Cluster Survey 2005 (Indirect) | 1 |
| | Demographic and Health Survey 2008-2009 (Direct) | 1 |
| | Demographic and Health Survey 2008-2009 (Indirect) | 0 |
| | Census 2011 (Indirect) | 0 |

| Country | Data series | Inclusion |
|---|---|---|
| | Demographic and Health Survey 2017-2018 (Direct) | 0 |
| | UNPD Vital Registration Data 2022 version 2022 (VR) | 0 |
| | Census 2023 (Indirect) | 0 |
| | WHO Vital Registration Data 2025 version 2025 (VR) | 1 |
| Andorra | Recalculated WHO Vital Registration Data 2021 version 2025 (VR) | 1 |
| | WHO Vital Registration Data 2025 version 2025 (VR) | 0 |
| United Arab Emirates | Census 1975 (Indirect) | 1 |
| | Census 1980 (Indirect) | 1 |
| | Child Health Survey 1987 (Indirect) | 1 |
| | Family Health Survey 1995 (Direct) | 1 |
| | Family Health Survey 1995 (Indirect) | 0 |
| | Vital Registration by Federal Competitiveness and Statistics Center 2023 (VR) | 1 |
| | WHO Vital Registration Data 2025 version 2025 (VR) | 0 |
| Argentina | TABLAS DE VIDA NACIONALES (Life Table) | 0 |
| | Census 1970 (Indirect) | 0 |
| | Census 1980 (Indirect) | 0 |
| | Census 1991 (Indirect) | 0 |
| | Census 2001 (Indirect) | 0 |
| | Census 2010 (Indirect) | 0 |
| | Multiple Indicator Cluster Survey 2019 (Indirect) | 0 |
| | Census 2022 (Indirect) | 0 |
| | VR from DEIS 2023 (VR) | 1 |
| | WHO Vital Registration Data 2025 version 2025 (VR) | 1 |
| Armenia | Census 1989 (Indirect) | 0 |
| | Demographic and Health Survey 2000 (Direct) | 1 |
| | Demographic and Health Survey 2000 (Indirect) | 0 |
| | Census 2001 (Indirect) | 1 |
| | Demographic and Health Survey 2005 (Direct) | 1 |
| | Demographic and Health Survey 2005 (Indirect) | 0 |
| | Transmonee Vital Registration Data 2008 version 2008 (VR) | 0 |
| | Demographic and Health Survey 2010 (Direct) | 1 |
| | Census 2011 (Indirect) | 0 |
| | Vital Registration Data from The Demographic Handbook of Armenia 2011 2011 (VR) | 0 |

| Country | Data series | Inclusion |
|---|---|---|
| | Census 2011 (Household Deaths) | 0 |
| | Vital Registration Data from The Demographic Handbook of Armenia 2014 2014 (VR) | 0 |
| | Vital Registration Data from The Demographic Handbook of Armenia 2016 2016 (VR) | 0 |
| | Demographic and Health Survey 2015-2016 (Direct) | 0 |
| | Census 2022 (Indirect) | 0 |
| | WHO Vital Registration Data 2025 version 2025 (VR) | 1 |
| Antigua and Barbuda | UNPD Vital Registration Data 2022 version 2022 (VR) | 1 |
| | Recalculated WHO Vital Registration Data 2025 version 2025 (VR) | 1 |
| | WHO Vital Registration Data 2025 version 2025 (VR) | 0 |
| Australia | WHO Vital Registration Data 2025 version 2025 (VR) | 1 |
| Austria | UNPD Vital Registration Data 2022 version 2022 (VR) | 1 |
| | WHO Vital Registration Data 2025 version 2025 (VR) | 1 |
| Azerbaijan | Census 1989 (Indirect) | 0 |
| | Census 1999 (Indirect) | 0 |
| | Multiple Indicator Cluster Survey 2000 (Indirect) | 1 |
| | Reproductive Health Survey 2001 (Direct) | 1 |
| | Demographic and Health Survey 2006 (Direct) | 1 |
| | Demographic and Health Survey 2006 (Indirect) | 0 |
| | Transmonee Vital Registration Data 2008 version 2008 (VR) | 0 |
| | Census 2009 (Indirect) | 0 |
| | Azerbaijan National DHS 2011 (Direct) | 1 |
| | VR from Children in Azerbaijan Report 2013 2013 (VR) | 0 |
| | VR from Children in Azerbaijan Report 2016 2016 (VR) | 0 |
| | Adjusted National Vital Registration Data 2018 (VR) | 1 |
| | Multiple Indicator Cluster Survey 2023 (Direct) | 0 |
| | WHO Vital Registration Data 2025 version 2025 (VR) | 1 |
| Burundi | Population Survey 1965 (Household Deaths) | 0 |
| | Demographic Survey 1970 (Direct) | 1 |
| | Demographic Survey 1970 (Indirect) | 0 |
| | Post Census Survey 1979 (Indirect) | 1 |
| | Demographic and Health Survey 1987 (Direct) | 1 |
| | Demographic and Health Survey 1987 (Indirect) | 0 |
| | Census 1990 (Indirect) | 1 |

| Country | Data series | Inclusion |
|---|---|---|
| | Multiple Indicator Cluster Survey 2000 (Indirect) | 1 |
| | Multiple Indicator Cluster Survey 2005 (Indirect) | 1 |
| | Census 2008 (Household Deaths) | 0 |
| | Census 2008 (Indirect) | 1 |
| | Demographic and Health Survey 2010-2011 (Direct) | 1 |
| | Enquete menages pour le suivi et l'evaluation de l'impact de l'appui au systeme de remboursement du Paquet Minimum des Services de sante 2012 (Indirect) | 1 |
| | Malaria Indicator Survey 2012 (Indirect) | 1 |
| | Demographic and Health Survey 2016-2017 (Direct) | 1 |
| Belgium | UNPD Vital Registration Data 2022 version 2022 (VR) | 1 |
| | WHO Vital Registration Data 2025 version 2025 (VR) | 1 |
| Benin | Survey 1961 (Indirect) | 1 |
| | World Fertility Survey 1981-1982 (Direct) | 1 |
| | World Fertility Survey 1981-1982 (Indirect) | 0 |
| | Enquete a passages repetes 1981-1983 (Household Deaths) | 1 |
| | Census 1992 (Indirect) | 1 |
| | Demographic and Health Survey 1996 (Direct) | 1 |
| | Demographic and Health Survey 1996 (Indirect) | 0 |
| | Demographic and Health Survey 2001 (Direct) | 1 |
| | Demographic and Health Survey 2001 (Indirect) | 0 |
| | Census 2002 (Indirect) | 1 |
| | Demographic and Health Survey 2006 (Direct) | 1 |
| | Demographic and Health Survey 2006 (Indirect) | 0 |
| | Demographic and Health Survey 2011-2012 (Direct) | 0 |
| | Census 2013 (Indirect) | 1 |
| | Multiple Indicator Cluster Survey 2014 (Direct) | 1 |
| | Demographic and Health Survey 2017-2018 (Direct) | 1 |
| | Multiple Indicator Cluster Survey 2021-2022 (Direct) | 0 |
| Burkina Faso | Survey 1960-1961 (Indirect) | 1 |
| | Post-Enumeration Survey 1976 (Indirect) | 1 |
| | Recensement General de la Population et de l'Habitat (RGPH) 1985 (Indirect) | 1 |
| | Recensement General de la Population et de l'Habitat (RGPH) 1985 (Household Deaths) | 0 |
| | Survey 1991 (Indirect) | 1 |
| | Demographic and Health Survey 1993 (Direct) | 1 |

| Country | Data series | Inclusion |
| --- | --- | --- |
| | Demographic and Health Survey 1993 (Indirect) | 0 |
| | Recensement General de la Population et de l'Habitat (RGPH) 1996 (Household Deaths) | 0 |
| | Demographic and Health Survey 1998-1999 (Direct) | 1 |
| | Demographic and Health Survey 1998-1999 (Indirect) | 0 |
| | Demographic and Health Survey 2003 (Direct) | 1 |
| | Demographic and Health Survey 2003 (Indirect) | 0 |
| | Multiple Indicator Cluster Survey 2006 (Indirect) | 1 |
| | Recensement General de la Population et de l'Habitat (RGPH) 2006 (Household Deaths) | 0 |
| | Recensement General de la Population et de l'Habitat (RGPH) 2006 (Indirect) | 0 |
| | Global Fund Evaluation Survey 2008 (Household Deaths) | 0 |
| | Demographic and Health Survey 2010 (Direct) | 1 |
| | Malaria Indicator Survey 2014 (Indirect) | 1 |
| | L Enquete sur le Module Demographie et Sante 2015 (Direct) | 0 |
| | Malaria Indicator Survey 2017-2018 (Indirect) | 1 |
| | Recensement General de la Population et de l'Habitat (RGPH) 2019 (Household Deaths) | 0 |
| | Enquete Demographique et de Sante 2021 (Direct) | 0 |
| Bangladesh | Population Growth Estimation Experiment 1962-1965 (Direct) | 1 |
| | Population Growth Estimation Experiment 1962-1965 (Household Deaths) | 0 |
| | Retrospective Fertility and Mortality Survey (UN SA) 1974 (Indirect) | 1 |
| | World Fertility Survey 1975-1976 (Direct) | 1 |
| | World Fertility Survey 1975-1976 (Indirect) | 0 |
| | Contraceptive Prevalence Survey 1979-1980 (Indirect) | 0 |
| | Contraceptive Prevalence Survey 1981 (Indirect) | 1 |
| | Contraceptive Prevalence Survey 1983-1984 (Indirect) | 1 |
| | Contraceptive Prevalence Survey 1985-1986 (Indirect) | 1 |
| | National Life Tables 1987 (Life Table) | 1 |
| | Fertility Survey 1988-1989 (Direct) | 1 |
| | Health and Demographic Survey 1994 (Indirect) | 1 |
| | Demographic and Health Survey 1993-1994 (Direct) | 1 |
| | Demographic and Health Survey 1993-1994 (Indirect) | 0 |
| | Demographic and Health Survey 1996-1997 (Direct) | 1 |
| | Demographic and Health Survey 1996-1997 (Indirect) | 0 |
| | Demographic and Health Survey 1999-2000 (Direct) | 1 |

| Country | Data series | Inclusion |
|---|---|---|
| | Demographic and Health Survey 1999-2000 (Indirect) | 0 |
| | Maternal Health Services and Maternal Mortality Survey 2001 (Direct) | 1 |
| | Demographic and Health Survey 2004 (Direct) | 1 |
| | Demographic and Health Survey 2004 (Indirect) | 0 |
| | Demographic and Health Survey 2007 (Direct) | 1 |
| | Demographic and Health Survey 2007 (Indirect) | 0 |
| | Multiple Indicator Cluster Survey 2009 (Indirect) | 1 |
| | Maternal Mortality and Health Care Survey 2010 (Direct) | 1 |
| | Census 2011 (Household Deaths) | 1 |
| | Demographic and Health Survey 2011 (Direct) | 1 |
| | Multiple Indicator Cluster Survey 2012-2013 (Indirect) | 1 |
| | Demographic and Health Survey 2014 (Direct) | 1 |
| | Maternal Mortality and Health Care Survey 2016 (Direct) | 1 |
| | Demographic and Health Survey 2017-2018 (Direct) | 1 |
| | Multiple Indicator Cluster Survey 2019 (Direct) | 1 |
| | Demographic and Health Survey 2022 (Direct) | 1 |
| | Report on Sample Vital Registration System 2023 (VR) | 1 |
| Bulgaria | UNPD Vital Registration Data 2022 version 2022 (VR) | 1 |
| | WHO Vital Registration Data 2025 version 2025 (VR) | 1 |
| Bahrain | Census 1965 (Indirect) | 0 |
| | Census 1971 (Indirect) | 1 |
| | Census 1981 (Indirect) | 1 |
| | Child Health Survey 1989 (Direct) | 1 |
| | Child Health Survey 1989 (Indirect) | 0 |
| | Census 1991 (Indirect) | 1 |
| | Gulf Family Health Survey 1995 (Direct) | 1 |
| | Gulf Family Health Survey 1995 (Indirect) | 0 |
| | Census 2001 (Indirect) | 1 |
| | WHO Vital Registration Data 2025 version 2025 (VR) | 1 |
| Bahamas | Recalculated WHO Vital Registration Data 2025 version 2025 (VR) | 1 |
| | WHO Vital Registration Data 2025 version 2025 (VR) | 0 |
| Bosnia and Herzegovina | WHO Vital Registration Data 2025 version 2025 (VR) | 1 |
| Belarus | Census 1989 (Indirect) | 0 |

| Country | Data series | Inclusion |
|---|---|---|
| | Census 1999 (Indirect) | 0 |
| | Multiple Indicator Cluster Survey 2005 (Indirect) | 0 |
| | Transmonee Vital Registration Data 2008 version 2008 (VR) | 0 |
| | Demographic Yearbook of the Republic of Belarus 2012 (VR) | 0 |
| | HMD Vital Registration Data 2025 (VR) | 1 |
| | WHO Vital Registration Data 2025 version 2025 (VR) | 1 |
| Belize | Family Health Survey 1991 (Direct) | 1 |
| | Family Health Survey 1999 (Indirect) | 1 |
| | Census 2000 (Indirect) | 1 |
| | Multiple Indicator Cluster Survey 2006 (Indirect) | 1 |
| | Vital Registration Data from MOH (Abstract of Statistics 2008) 2008 (VR) | 0 |
| | Multiple Indicator Cluster Survey 2011 (Indirect) | 1 |
| | Multiple Indicator Cluster Survey 2015-2016 (Direct) | 0 |
| | UNPD Vital Registration Data 2022 version 2022 (VR) | 0 |
| | Multiple Indicator Cluster Survey 2024 (Direct) | 0 |
| | Recalculated WHO Vital Registration Data 2025 version 2025 (VR) | 1 |
| | WHO Vital Registration Data 2025 version 2025 (VR) | 0 |
| Bolivia (Plurinational State of) | EDEN 1975 (Indirect) | 1 |
| | Census 1976 (Indirect) | 1 |
| | Encuesta Demografica Nacional 1980 (Household Deaths) | 1 |
| | Encuesta Demografica Nacional 1980 (Indirect) | 1 |
| | ENPV 1988 (Indirect) | 1 |
| | Demographic and Health Survey 1989 (Direct) | 1 |
| | Demographic and Health Survey 1989 (Indirect) | 0 |
| | Census 1992 (Household Deaths) | 0 |
| | Census 1992 (Indirect) | 0 |
| | Demographic and Health Survey 1993-1994 (Direct) | 1 |
| | Demographic and Health Survey 1993-1994 (Indirect) | 0 |
| | Demographic and Health Survey 1998 (Direct) | 1 |
| | Demographic and Health Survey 1998 (Indirect) | 0 |
| | Multiple Indicator Cluster Survey 2000 (Indirect) | 1 |
| | Census 2001 (Household Deaths) | 0 |
| | Census 2001 (Indirect) | 1 |

| Country | Data series | Inclusion |
| --- | --- | --- |
| | Demographic and Health Survey 2003 (Direct) | 1 |
| | Demographic and Health Survey 2003 (Indirect) | 0 |
| | Demographic and Health Survey 2008 (Direct) | 1 |
| | Census 2012 (Household Deaths) | 1 |
| | Census 2012 (Indirect) | 1 |
| | Demographic and Health Survey 2016 (Direct) | 1 |
| | Demographic and Health Survey (EDSA) 2023 (Direct) | 1 |
| Brazil | Census 1940 (Indirect) | 1 |
| | Census 1950 (Indirect) | 1 |
| | Census 1960 (Indirect) | 1 |
| | Census 1970 (Indirect) | 1 |
| | National Health Survey 1972 (Indirect) | 1 |
| | National Health Survey 1973 (Indirect) | 1 |
| | National Health Survey 1976 (Indirect) | 1 |
| | National Health Survey 1977 (Indirect) | 1 |
| | National Health Survey 1978 (Indirect) | 1 |
| | Census 1980 (Indirect) | 1 |
| | National Health Survey 1984 (Indirect) | 1 |
| | Pesquisa Nacional por Amostra de Domicilios 1986 (Direct) | 1 |
| | Pesquisa Nacional por Amostra de Domicilios 1986 (Indirect) | 1 |
| | Demographic and Health Survey 1986 (Direct) | 1 |
| | Demographic and Health Survey 1986 (Indirect) | 0 |
| | Census 1991 (Indirect) | 1 |
| | Demographic and Health Survey 1996 (Direct) | 1 |
| | Demographic and Health Survey 1996 (Indirect) | 0 |
| | Census 2000 (Indirect) | 1 |
| | Pesquisa Nacional por Amostra de Domicilios 2002 (Indirect) | 1 |
| | Pesquisa Nacional por Amostra de Domicilios 2003 (Indirect) | 1 |
| | Pesquisa Nacional por Amostra de Domicilios 2004 (Indirect) | 1 |
| | Pesquisa Nacional por Amostra de Domicilios 2005 (Indirect) | 1 |
| | Pesquisa Nacional por Amostra de Domicilios 2006 (Indirect) | 1 |
| | Pesquisa Nacional por Amostra de Domicilios 2007 (Indirect) | 1 |
| | Pesquisa Nacional por Amostra de Domicilios 2008 (Indirect) | 1 |

| Country | Data series | Inclusion |
|---|---|---|
| | Pesquisa Nacional por Amostra de Domicilios 2009 (Indirect) | 1 |
| | Census 2010 (Household Deaths) | 0 |
| | Census 2010 (Indirect) | 0 |
| | Pesquisa Nacional por Amostra de Domicilios 2011 (Indirect) | 1 |
| | Pesquisa Nacional por Amostra de Domicilios 2012 (Indirect) | 1 |
| | Pesquisa Nacional por Amostra de Domicilios 2013 (Indirect) | 1 |
| | Pesquisa Nacional por Amostra de Domicilios 2014 (Indirect) | 1 |
| | Pesquisa Nacional por Amostra de Domicilios 2015 (Indirect) | 1 |
| | Data from Information System of Ministry of Health (Busca Ativa) 2023 (VR) | 1 |
| | WHO Vital Registration Data 2025 version 2025 (VR) | 0 |
| Barbados | UNPD Vital Registration Data 2022 version 2022 (VR) | 1 |
| | Recalculated WHO Vital Registration Data 2025 version 2025 (VR) | 1 |
| | WHO Vital Registration Data 2025 version 2025 (VR) | 0 |
| Brunei Darussalam | Census 1960 (Indirect) | 1 |
| | UNPD Vital Registration Data 2022 version 2022 (VR) | 1 |
| | Recalculated WHO Vital Registration Data 2025 version 2025 (VR) | 1 |
| | WHO Vital Registration Data 2025 version 2025 (VR) | 0 |
| Bhutan | Demographic Sample Survey 1984 (Direct) | 1 |
| | Demographic Sample Survey 1984 (Indirect) | 1 |
| | National Health Survey 1994 (Household Deaths) | 1 |
| | National Health Survey 2000 (Direct) | 1 |
| | Census 2005 (Household Deaths) | 1 |
| | Census 2005 (Indirect) | 1 |
| | Multiple Indicator Cluster Survey 2010 (Indirect) | 1 |
| | National Health Survey 2012 (Direct) | 1 |
| | Census 2017 (Household Deaths) | 0 |
| | National Health Survey 2023 (Direct) | 1 |
| Botswana | Census 1971 (Indirect) | 1 |
| | Census 1981 (Household Deaths) | 0 |
| | Census 1981 (Indirect) | 1 |
| | Family Health Survey 1984 (Indirect) | 1 |
| | Family Health Survey 1988 (Direct) | 0 |
| | Family Health Survey 1988 (Indirect) | 0 |

| Country | Data series | Inclusion |
| --- | --- | --- |
| | Family Health Survey (MM adjusted) 1988 (Direct) | 1 |
| | Census 1991 (Household Deaths) | 0 |
| | Census 1991 (Indirect) | 1 |
| | Family Health Survey 1996 (Direct) | 0 |
| | Family Health Survey (MM adjusted) 1996 (Direct) | 0 |
| | Demographic Survey 1998 (Household Deaths) | 0 |
| | Multiple Indicator Cluster Survey 2000 (Indirect) | 1 |
| | Census 2001 (Household Deaths) | 0 |
| | Census 2001 (Indirect) | 1 |
| | Demographic Survey 2006 (Household Deaths) | 0 |
| | Demographic Survey 2006 (Indirect) | 1 |
| | Family Health Survey 2007 (Direct) | 0 |
| | Family Health Survey (MM adjusted) 2007 (Direct) | 1 |
| | Census 2011 (Household Deaths) | 0 |
| | Census 2011 (Indirect) | 1 |
| | Demographic Survey 2017 (Indirect) | 1 |
| | Census 2022 (Household Deaths) | 0 |
| | Census 2022 (Indirect) | 0 |
| | Vital Statistics Report from Statistics Botswana 2023 (VR) | 0 |
| Central African Republic | Survey 1959-1960 (Household Deaths) | 0 |
| | Census 1975 (Indirect) | 1 |
| | Census 1988 (Household Deaths) | 0 |
| | Census 1988 (Indirect) | 1 |
| | Demographic and Health Survey 1994-1995 (Direct) | 0 |
| | Demographic and Health Survey 1994-1995 (Indirect) | 0 |
| | Demographic and Health Survey (MM adjusted) 1994-1995 (Direct) | 1 |
| | Multiple Indicator Cluster Survey 2000 (Indirect) | 1 |
| | Census 2003 (Household Deaths) | 0 |
| | Multiple Indicator Cluster Survey 2006 (Indirect) | 1 |
| | Multiple Indicator Cluster Survey 2010 (Indirect) | 1 |
| | Enquete Nationale Sur la Situation Nutritionnelle et la Mortalite 2012 (Household Deaths) | 0 |
| | Multiple Indicator Cluster Survey 2018-2019 (Direct) | 0 |
| | Multiple Indicator Cluster Survey (MM adjusted) 2018-2019 (Direct) | 1 |

| Country | Data series | Inclusion |
|---|---|---|
| Canada | WHO Vital Registration Data 2025 version 2025 (VR) | 1 |
| Switzerland | WHO Vital Registration Data 2025 version 2025 (VR) | 1 |
| Chile | TABLAS DE VIDA NACIONALES (Life Table) | 0 |
| | Vital Registration from Ministerio de Salud. Departamento de Estadisticas de Salud.Anexo Salud1 (VR) | 0 |
| | Census 1970 (Indirect) | 0 |
| | Census 1982 (Indirect) | 0 |
| | Census 1992 (Indirect) | 0 |
| | Census 2002 (Indirect) | 0 |
| | Census 2017 (Indirect) | 0 |
| | WHO Vital Registration Data 2025 version 2025 (VR) | 1 |
| China | Adjusted Census Deaths 1964-2000 (Household Deaths) | 1 |
| | National Life Tables (Life Table) | 0 |
| | Census 1982 (Indirect) | 1 |
| | Population Sample Survey 1982 (Indirect) | 1 |
| | Intercensal Population Sample Survey of One-Percent 1987 (Household Deaths) | 0 |
| | Intercensal Population Sample Survey of One-Percent 1987 (Indirect) | 1 |
| | National Survey on Fertility and Birth Control 1988 (Direct) | 1 |
| | Census 1990 (Indirect) | 0 |
| | Fertility Sampling Survey 1992 (Direct) | 1 |
| | Intercensal Population Sample Survey of One-Percent 1995 (Indirect) | 0 |
| | Census 2000 (Indirect) | 0 |
| | Intercensal Population Sample Survey of One-Percent 2005 (Direct) | 0 |
| | Intercensal Population Sample Survey of One-Percent 2005 (Indirect) | 0 |
| | National Survey on Causes of Death 2004-2005 (Household Deaths) | 0 |
| | Census 2010 (Household Deaths) | 0 |
| | Census 2010 (Indirect) | 0 |
| | Intercensal Population Sample Survey of One-Percent 2015 (Indirect) | 0 |
| | Census 2020 (Household Deaths) | 0 |
| | NHFPC, Death Registration Data 2024 (VR) | 1 |
| Côte d'Ivoire | Survey 1957-1958 (Indirect) | 0 |
| | Demographic Survey Repeated Passages 1978-1979 (Direct) | 1 |
| | Demographic Survey Repeated Passages 1978-1979 (Indirect) | 0 |
| | Demographic Survey Repeated Passages 1978-1979 (Household Deaths) | 0 |

| Country | Data series | Inclusion |
|---|---|---|
| | World Fertility Survey 1980-1981 (Direct) | 1 |
| | World Fertility Survey 1980-1981 (Indirect) | 0 |
| | Census 1988 (Indirect) | 0 |
| | Demographic and Health Survey 1994 (Direct) | 0 |
| | Demographic and Health Survey 1994 (Indirect) | 0 |
| | Demographic and Health Survey (MM adjusted) 1994 (Direct) | 1 |
| | Census 1998 (Household Deaths) | 0 |
| | Demographic and Health Survey 1998-1999 (Direct) | 0 |
| | Demographic and Health Survey 1998-1999 (Indirect) | 0 |
| | Demographic and Health Survey (MM adjusted) 1998-1999 (Direct) | 1 |
| | AIDS Indicator Survey 2005 (Household Deaths) | 0 |
| | AIDS Indicator Survey 2005 (Direct) | 0 |
| | AIDS Indicator Survey 2005 (Indirect) | 0 |
| | AIDS Indicator Survey (MM adjusted) 2005 (Direct) | 1 |
| | Demographic and Health Survey 2011-2012 (Direct) | 0 |
| | Demographic and Health Survey (MM adjusted) 2011-2012 (Direct) | 1 |
| | Multiple Indicator Cluster Survey 2016 (Direct) | 0 |
| | Multiple Indicator Cluster Survey (MM adjusted) 2016 (Direct) | 1 |
| | Enquete Demographique et de Sante 2021 (Direct) | 0 |
| | Enquete Demographique et de Sante (MM adjusted) 2021 (Direct) | 1 |
| Cameroon | Census 1976 (Household Deaths) | 0 |
| | World Fertility Survey 1978 (Direct) | 1 |
| | World Fertility Survey 1978 (Indirect) | 0 |
| | Census 1987 (Household Deaths) | 0 |
| | Demographic and Health Survey 1991 (Direct) | 0 |
| | Demographic and Health Survey 1991 (Indirect) | 0 |
| | Demographic and Health Survey (MM adjusted) 1991 (Direct) | 1 |
| | Demographic and Health Survey 1998 (Direct) | 0 |
| | Demographic and Health Survey 1998 (Indirect) | 0 |
| | Demographic and Health Survey (MM adjusted) 1998 (Direct) | 1 |
| | Multiple Indicator Cluster Survey 2000 (Indirect) | 1 |
| | Demographic and Health Survey 2004 (Direct) | 0 |
| | Demographic and Health Survey 2004 (Indirect) | 0 |

| Country | Data series | Inclusion |
|---|---|---|
| | Demographic and Health Survey (MM adjusted) 2004 (Direct) | 1 |
| | Census 2005 (Household Deaths) | 0 |
| | Census 2005 (Indirect) | 0 |
| | Demographic and Health Survey 2011 (Direct) | 0 |
| | Demographic and Health Survey (MM adjusted) 2011 (Direct) | 1 |
| | Multiple Indicator Cluster Survey 2014 (Direct) | 0 |
| | Multiple Indicator Cluster Survey (MM adjusted) 2014 (Direct) | 1 |
| | Demographic and Health Survey 2018 (Direct) | 0 |
| | Demographic and Health Survey (MM adjusted) 2018 (Direct) | 1 |
| | Malaria Indicator Survey 2022 (Indirect) | 1 |
| Democratic Republic of the Congo | Census 1984 (Indirect) | 1 |
| | Multiple Indicator Cluster Survey 1995 (Indirect) | 1 |
| | Multiple Indicator Cluster Survey 2001 (Indirect) | 1 |
| | Demographic and Health Survey 2007 (Direct) | 1 |
| | Demographic and Health Survey 2007 (Indirect) | 0 |
| | Multiple Indicator Cluster Survey 2010 (Indirect) | 1 |
| | Demographic and Health Survey 2013-2014 (Direct) | 1 |
| | Multiple Indicator Cluster Survey 2017-2018 (Direct) | 0 |
| | Demographic and Health Survey 2023-2024 (Direct) | 1 |
| Congo | Demographic Survey 1960-1961 (Indirect) | 1 |
| | Census 1974 (Household Deaths) | 0 |
| | Census 1974 (Indirect) | 1 |
| | Census 1984 (Household Deaths) | 0 |
| | Demographic and Health Survey 2005 (Direct) | 1 |
| | Demographic and Health Survey 2005 (Indirect) | 0 |
| | AIDS Indicator Survey 2009 (Indirect) | 1 |
| | Demographic and Health Survey 2011-2012 (Direct) | 1 |
| | Multiple Indicator Cluster Survey 2014-2015 (Direct) | 1 |
| Cook Islands | Census 1966 (Indirect) | 1 |
| | Census 1976 (Indirect) | 0 |
| | Census 1981 (Indirect) | 1 |
| | Census 1996 (Indirect) | 1 |
| | Census 2001 (Indirect) | 1 |

| Country | Data series | Inclusion |
| --- | --- | --- |
| | Census 2006 (Indirect) | 1 |
| | Census 2011 (Indirect) | 1 |
| | Vital Statistics Report 2009-2013 (VR) | 0 |
| | Census 2021 (Indirect) | 1 |
| | Recalculated WHO Vital Registration Data 2025 version 2025 (VR) | 1 |
| | WHO Vital Registration Data 2025 version 2025 (VR) | 0 |
| Colombia | Census 1973 (Indirect) | 1 |
| | World Fertility Survey 1976 (Indirect) | 0 |
| | World Fertility Survey 1976 (Direct) | 1 |
| | Contraceptive Prevalence Survey 1978 (Indirect) | 1 |
| | Household Survey (HOG) 1978 (Indirect) | 1 |
| | Household Survey (HOG) 1980 (Indirect) | 1 |
| | Census 1985 (Indirect) | 1 |
| | Demographic and Health Survey 1986 (Direct) | 1 |
| | Demographic and Health Survey 1986 (Indirect) | 0 |
| | Demographic and Health Survey 1990 (Direct) | 1 |
| | Demographic and Health Survey 1990 (Indirect) | 0 |
| | Census 1993 (Indirect) | 1 |
| | Demographic and Health Survey 1995 (Direct) | 1 |
| | Demographic and Health Survey 1995 (Indirect) | 0 |
| | Demographic and Health Survey 2000 (Direct) | 1 |
| | Demographic and Health Survey 2000 (Indirect) | 0 |
| | Census 2005 (Household Deaths) | 0 |
| | Census 2005 (Indirect) | 0 |
| | Demographic and Health Survey 2005 (Direct) | 1 |
| | Demographic and Health Survey 2005 (Indirect) | 0 |
| | Vital Registration (Mexico March 2009) 2009 (VR) | 0 |
| | Demographic and Health Survey 2010 (Direct) | 1 |
| | Demographic and Health Survey 2010 (Indirect) | 0 |
| | Demographic and Health Survey 2015 (Direct) | 1 |
| | Census 2018 (Household Deaths) | 0 |
| | UNPD Vital Registration Data 2022 version 2022 (VR) | 0 |
| | WHO Vital Registration Data 2025 version 2025 (VR) | 0 |

| Country | Data series | Inclusion |
| --- | --- | --- |
| Comoros | Census 1958 (Household Deaths) | 0 |
| | Census 1980 (Household Deaths) | 1 |
| | Census 1980 (Indirect) | 0 |
| | Census 1991 (Household Deaths) | 1 |
| | Demographic and Health Survey 1996 (Direct) | 1 |
| | Demographic and Health Survey 1996 (Indirect) | 0 |
| | Multiple Indicator Cluster Survey 2000 (Indirect) | 0 |
| | Census 2003 (Household Deaths) | 1 |
| | Demographic and Health Survey 2012 (Direct) | 0 |
| | Census 2017 (Household Deaths) | 1 |
| | Multiple Indicator Cluster Survey 2022 (Direct) | 1 |
| Cabo Verde | Census 1960 (Indirect) | 1 |
| | Census 1970 (Direct) | 1 |
| | Census 1980 (Direct) | 1 |
| | Demographic and Reproductive Health Survey 1998 (Direct) | 1 |
| | Census 2000 (Direct) | 1 |
| | Census 2000 (Household Deaths) | 0 |
| | Demographic and Health Survey 2005 (Direct) | 1 |
| | Demographic and Health Survey 2005 (Indirect) | 0 |
| | Vital Registration Data from Relatorio Estatistico 2007 2009 Ministry of Health 2007-2009 (VR) | 0 |
| | Census 2010 (Indirect) | 1 |
| | VR from Relatorio Estatistico 2012 (VR) | 0 |
| | Demographic and Reproductive Health Survey 2018 (Direct) | 1 |
| | UNPD Vital Registration Data 2022 version 2022 (VR) | 0 |
| | WHO Vital Registration Data 2025 version 2025 (VR) | 1 |
| Costa Rica | TABLAS DE VIDA NACIONALES (Life Table) | 0 |
| | Vital Registration (Mexico March 26, Roberto) (VR) | 0 |
| | Census 1973 (Indirect) | 1 |
| | World Fertility Survey 1976 (Direct) | 1 |
| | World Fertility Survey 1976 (Indirect) | 0 |
| | Contraceptive Prevalence Survey 1978 (Indirect) | 0 |
| | Contraceptive Prevalence Survey 1981 (Indirect) | 0 |
| | Census 1984 (Indirect) | 1 |

| Country | Data series | Inclusion |
|---|---|---|
| | National Survey of Fertility and Health 1986 (Indirect) | 0 |
| | Census 2000 (Indirect) | 0 |
| | Census 2011 (Indirect) | 0 |
| | Multiple Indicator Cluster Survey 2018 (Indirect) | 0 |
| | UNPD Vital Registration Data 2022 version 2022 (VR) | 1 |
| | WHO Vital Registration Data 2025 version 2025 (VR) | 1 |
| Cuba | TABLAS DE VIDA NACIONALES (Life Table) | 0 |
| | National Population Survey on Income and Expenditures 1974 (Indirect) | 0 |
| | National Demographic Survey 1979 (Indirect) | 0 |
| | Census 1981 (Indirect) | 0 |
| | National Fertility Survey 1987 (Indirect) | 0 |
| | Multiple Indicator Cluster Survey 2010-2011 (Indirect) | 0 |
| | UNPD Vital Registration Data 2022 version 2022 (VR) | 1 |
| | Vital Registration Data from Ministerio de Salud Publica de Cuba 2024 (VR) | 0 |
| | WHO Vital Registration Data 2025 version 2025 (VR) | 1 |
| Cyprus | UNPD Vital Registration Data 2022 version 2022 (VR) | 1 |
| | Recalculated WHO Vital Registration Data 2025 version 2025 (VR) | 1 |
| | WHO Vital Registration Data 2025 version 2025 (VR) | 0 |
| Czechia | HMD Vital Registration Data 2025 (VR) | 1 |
| | WHO Vital Registration Data 2025 version 2025 (VR) | 1 |
| Germany | WHO Vital Registration Data 2025 version 2025 (VR) | 1 |
| Djibouti | Demographic Survey 1991 (Indirect) | 1 |
| | PAPFAM Family Health Survey 2002 (Direct) | 1 |
| | Multiple Indicator Cluster Survey 2006 (Indirect) | 1 |
| | PAPFAM Family Health Survey 2012 (Direct) | 1 |
| Dominica | Recalculated WHO Vital Registration Data 2025 version 2025 (VR) | 1 |
| | WHO Vital Registration Data 2025 version 2025 (VR) | 0 |
| Denmark | HMD Vital Registration Data 2025 (VR) | 1 |
| | WHO Vital Registration Data 2025 version 2025 (VR) | 1 |
| Dominican Republic | Census 1970 (Indirect) | 1 |
| | World Fertility Survey 1975 (Direct) | 1 |
| | World Fertility Survey 1975 (Indirect) | 0 |
| | World Fertility Survey 1980 (Indirect) | 0 |

| Country | Data series | Inclusion |
|---|---|---|
| | World Fertility Survey 1980 (Direct) | 1 |
| | Census 1981 (Indirect) | 1 |
| | Contraceptive Prevalence Survey 1983 (Indirect) | 1 |
| | Demographic and Health Survey 1986 (Direct) | 1 |
| | Demographic and Health Survey 1986 (Indirect) | 0 |
| | Demographic and Health Survey 1991 (Direct) | 1 |
| | Demographic and Health Survey 1991 (Indirect) | 0 |
| | Demographic and Health Survey 1996 (Direct) | 1 |
| | Demographic and Health Survey 1996 (Indirect) | 0 |
| | Demographic and Health Survey 1999 (Direct) | 0 |
| | Multiple Indicator Cluster Survey 2000 (Indirect) | 1 |
| | Census 2002 (Indirect) | 0 |
| | Demographic and Health Survey 2002 (Direct) | 1 |
| | Demographic and Health Survey 2002 (Indirect) | 0 |
| | Encuesta Nacional de Hogares de Propositos Multiples 2006 (Direct) | 1 |
| | Demographic and Health Survey 2007 (Direct) | 1 |
| | Demographic and Health Survey 2007 (Indirect) | 0 |
| | Census 2010 (Household Deaths) | 0 |
| | Census 2010 (Indirect) | 0 |
| | Demographic and Health Survey 2013 (Direct) | 1 |
| | Multiple Indicator Cluster Survey 2014 (Direct) | 1 |
| | Multiple Indicator Cluster Survey 2019 (Direct) | 1 |
| | VR from Ministerio de Salud Publica 2021 (VR) | 0 |
| | VR from Oficina Nacional de Estadistica 2021 (VR) | 0 |
| | WHO Vital Registration Data 2025 version 2025 (VR) | 0 |
| Algeria | Life Tables  (Life Table) | 1 |
| | Algeria Fertility Survey 1970 (Indirect) | 1 |
| | Demographic Survey 1970 (Direct) | 1 |
| | National Fertility Survey 1986 (Direct) | 1 |
| | PAPCHILD Maternal and Child Health Survey 1992 (Direct) | 1 |
| | Multiple Indicator Cluster Survey 1995 (Direct) | 1 |
| | Multiple Indicator Cluster Survey 2000 (Indirect) | 1 |
| | PAPFAM Family Health Survey 2002 (Direct) | 1 |

| Country | Data series | Inclusion |
| --- | --- | --- |
| | Multiple Indicator Cluster Survey 2006 (Indirect) | 1 |
| | Multiple Indicator Cluster Survey 2012-2013 (Direct) | 0 |
| | Multiple Indicator Cluster Survey 2018-2019 (Direct) | 0 |
| | VR from Demographie Algerienne 2023 (VR) | 1 |
| | WHO Vital Registration Data 2025 version 2025 (VR) | 1 |
| Ecuador | Census 1974 (Indirect) | 1 |
| | World Fertility Survey 1979-1980 (Direct) | 1 |
| | World Fertility Survey 1979-1980 (Indirect) | 0 |
| | Census 1982 (Indirect) | 1 |
| | ESMID 1982 (Indirect) | 1 |
| | Demographic and Family Health Survey 1987 (Direct) | 1 |
| | Demographic and Family Health Survey 1987 (Indirect) | 0 |
| | Demographic and Maternal and Child Health Survey 1989 (Direct) | 1 |
| | Census 1990 (Indirect) | 1 |
| | Demographic and Maternal and Child Health Survey 1994 (Direct) | 0 |
| | Demographic and Maternal and Child Health Survey 1999 (Direct) | 1 |
| | Census 2001 (Indirect) | 1 |
| | Demographic and Maternal and Child Health Survey 2004 (Direct) | 1 |
| | Census 2010 (Indirect) | 1 |
| | Encuesta Nacional de Salud y Nutricion (ENSANUT) 2012 (Direct) | 0 |
| | Encuesta Nacional de Salud y Nutricion (ENSANUT), Imputed birth 2012 (Direct) | 0 |
| | Encuesta Nacional de Salud y Nutricion (ENSANUT), Imputed birth and deaths 2012 (Direct) | 1 |
| | Adjusted Vital Registration (Live Births) 2018 (VR) | 1 |
| | Vital Registration (INEC Population projection) 2018 (VR) | 0 |
| | Encuesta Nacional de Salud y Nutricion (ENSANUT) 2018 (Direct) | 0 |
| | Encuesta Nacional de Salud y Nutricion (ENSANUT) 2018 (Indirect) | 0 |
| | Vital Registration (Live Births) 2021 (VR) | 1 |
| | Census 2022 (Indirect) | 1 |
| | UNPD Vital Registration Data 2022 version 2022 (VR) | 0 |
| | Census 2022 (Household Deaths) | 0 |
| | Vital Registration (Live Births) 2024 (VR) | 1 |
| | WHO Vital Registration Data 2025 version 2025 (VR) | 0 |
| Egypt | Census 1947 (Household Deaths) | 0 |

| Country | Data series | Inclusion |
|---|---|---|
| | Census 1960 (Household Deaths) | 0 |
| | Census 1976 (Indirect) | 0 |
| | World Fertility Survey 1980 (Direct) | 1 |
| | World Fertility Survey 1980 (Indirect) | 0 |
| | Contraceptive Prevalence Survey 1984 (Indirect) | 1 |
| | Census 1986 (Indirect) | 0 |
| | Demographic and Health Survey 1988 (Direct) | 1 |
| | Demographic and Health Survey 1988 (Indirect) | 0 |
| | PAPCHILD Maternal and Child Health Survey 1991 (Direct) | 1 |
| | Demographic and Health Survey 1992 (Direct) | 1 |
| | Demographic and Health Survey 1992 (Indirect) | 0 |
| | Demographic and Health Survey 1995 (Direct) | 1 |
| | Demographic and Health Survey 1995 (Indirect) | 0 |
| | Demographic and Health Survey 1997 (Direct) | 1 |
| | Demographic and Health Survey 1998 (Direct) | 1 |
| | Demographic and Health Survey 2000 (Direct) | 1 |
| | Demographic and Health Survey 2000 (Indirect) | 0 |
| | Demographic and Health Survey 2003 (Direct) | 1 |
| | Demographic and Health Survey 2003 (Indirect) | 0 |
| | Demographic and Health Survey 2005 (Direct) | 1 |
| | Demographic and Health Survey 2005 (Indirect) | 0 |
| | Demographic and Health Survey 2008 (Direct) | 1 |
| | Demographic and Health Survey 2014 (Direct) | 1 |
| | Health Indicator Survey 2015 (Indirect) | 1 |
| | UNPD Vital Registration Data 2022 version 2022 (VR) | 0 |
| | Family Health Survey 2021 (Direct) | 1 |
| | WHO Vital Registration Data 2025 version 2025 (VR) | 0 |
| Eritrea | Demographic and Health Survey 1995-1996 (Direct) | 1 |
| | Demographic and Health Survey 1995-1996 (Indirect) | 0 |
| | Demographic and Health Survey 2002 (Direct) | 1 |
| | Demographic and Health Survey 2002 (Indirect) | 0 |
| | Population and Health Survey 2010 (Direct) | 1 |
| Spain | WHO Vital Registration Data 2025 version 2025 (VR) | 1 |

| Country | Data series | Inclusion |
|---|---|---|
| Estonia | Census 2011-2012 (Household Deaths) | 0 |
| | HMD Vital Registration Data 2025 (VR) | 1 |
| | Recalculated WHO Vital Registration Data 2025 version 2025 (VR) | 1 |
| | WHO Vital Registration Data 2025 version 2025 (VR) | 0 |
| Ethiopia | Demographic Survey 1981 (Household Deaths) | 0 |
| | Demographic Survey 1981 (Indirect) | 1 |
| | Census 1984 (Household Deaths) | 0 |
| | Census 1984 (Indirect) | 0 |
| | National Family and Fertility Survey 1990 (Direct) | 1 |
| | National Family and Fertility Survey 1990 (Indirect) | 0 |
| | Census 1994 (Indirect) | 0 |
| | Demographic and Health Survey 2000 (Direct) | 1 |
| | Demographic and Health Survey 2000 (Indirect) | 0 |
| | Demographic and Health Survey 2005 (Direct) | 1 |
| | Demographic and Health Survey 2005 (Indirect) | 0 |
| | Census 2007 (Household Deaths) | 0 |
| | Census 2007 (Indirect) | 0 |
| | Demographic and Health Survey 2011 (Direct) | 1 |
| | Mini Demographic and Health Survey 2014 (Indirect) | 0 |
| | Mini Demographic and Health Survey 2014 (Direct) | 1 |
| | Demographic and Health Survey 2016 (Direct) | 1 |
| | Mini Demographic and Health Survey 2019 (Direct) | 1 |
| | Demographic and Health Survey 2024-2025 (Direct) | 0 |
| Finland | UNPD Vital Registration Data 2022 version 2022 (VR) | 1 |
| | WHO Vital Registration Data 2025 version 2025 (VR) | 1 |
| Fiji | Census 1956 (Indirect) | 1 |
| | Census 1966 (Indirect) | 1 |
| | World Fertility Survey 1974 (Direct) | 1 |
| | Census 1976 (Indirect) | 1 |
| | Census 1986 (Indirect) | 1 |
| | Census 1996 (Indirect) | 1 |
| | Census 2007 (Indirect) | 1 |
| | Vital Statistics Report 2016 (VR) | 0 |

| Country | Data series | Inclusion |
|---|---|---|
| | Multiple Indicator Cluster Survey 2021 (Direct) | 0 |
| | WHO Vital Registration Data 2025 version 2025 (VR) | 1 |
| France | WHO Vital Registration Data 2025 version 2025 (VR) | 1 |
| Micronesia (Federated States of) | Census 1973 (Indirect) | 1 |
| | Census 1994 (Indirect) | 1 |
| | Census 2000 (Indirect) | 1 |
| | Census 2010 (Indirect) | 1 |
| Gabon | Census 1960-1961 (Household Deaths) | 0 |
| | Census 1960-1961 (Indirect) | 1 |
| | Demographic and Health Survey 2000 (Direct) | 0 |
| | Demographic and Health Survey 2000 (Indirect) | 0 |
| | Demographic and Health Survey (MM adjusted) 2000 (Direct) | 1 |
| | Demographic and Health Survey 2012 (Direct) | 0 |
| | Demographic and Health Survey (MM adjusted) 2012 (Direct) | 1 |
| | Demographic and Health Survey 2019-2021 (Direct) | 0 |
| | Demographic and Health Survey (MM adjusted) 2019-2021 (Direct) | 1 |
| United Kingdom | WHO Vital Registration Data 2025 version 2025 (VR) | 1 |
| Georgia | Census 1989 (Indirect) | 0 |
| | Reproductive Health Survey 1999-2000 (Direct) | 1 |
| | Census 2002 (Indirect) | 1 |
| | Multiple Indicator Cluster Survey 2005 (Indirect) | 1 |
| | Reproductive Health Survey 2005 (Direct) | 1 |
| | Reproductive Health Survey 2010 (Direct) | 1 |
| | Census 2014 (Indirect) | 0 |
| | Multiple Indicator Cluster Survey 2018 (Indirect) | 0 |
| | Vital Statistics of the National Center for Disease Control and Public Health 2021 (VR) | 1 |
| | WHO Vital Registration Data 2025 version 2025 (VR) | 1 |
| Ghana | Census 1948 (Indirect) | 1 |
| | Census 1960 (Indirect) | 1 |
| | Dual Registration 1969 (VR) | 1 |
| | Census 1971 (Indirect) | 1 |
| | CCP Registration 1975 (VR) | 1 |
| | World Fertility Survey 1979-1980 (Direct) | 0 |

| Country | Data series | Inclusion |
|---|---|---|
| | World Fertility Survey 1979-1980 (Indirect) | 0 |
| | Demographic and Health Survey 1988 (Direct) | 1 |
| | Demographic and Health Survey 1988 (Indirect) | 0 |
| | Demographic and Health Survey 1993-1994 (Direct) | 1 |
| | Demographic and Health Survey 1993-1994 (Indirect) | 0 |
| | Demographic and Health Survey 1998-1999 (Direct) | 1 |
| | Demographic and Health Survey 1998-1999 (Indirect) | 0 |
| | Census 2000 (Indirect) | 0 |
| | Demographic and Health Survey 2003 (Direct) | 1 |
| | Demographic and Health Survey 2003 (Indirect) | 0 |
| | Multiple Indicator Cluster Survey 2006 (Indirect) | 1 |
| | Maternal Health Survey 2007 (Direct) | 1 |
| | Demographic and Health Survey 2008 (Direct) | 1 |
| | Demographic and Health Survey 2008 (Indirect) | 0 |
| | Census 2010 (Indirect) | 1 |
| | Census 2010 (Household Deaths) | 0 |
| | Multiple Indicator Cluster Survey 2011 (Direct) | 1 |
| | Demographic and Health Survey 2014 (Direct) | 1 |
| | Malaria Indicator Survey 2016 (Indirect) | 1 |
| | Maternal Health Survey 2017 (Direct) | 1 |
| | Multiple Indicator Cluster Survey 2017-2018 (Direct) | 1 |
| | Malaria Indicator Survey 2019 (Indirect) | 1 |
| | Census 2021 (Indirect) | 0 |
| | Census 2021 (Household Deaths) | 0 |
| | Demographic and Health Survey 2022 (Direct) | 1 |
| Guinea | Survey 1954-1955 (Household Deaths) | 1 |
| | Survey 1954-1955 (Indirect) | 1 |
| | Census 1983 (Household Deaths) | 0 |
| | Demographic and Health Survey 1992 (Direct) | 1 |
| | Demographic and Health Survey 1992 (Indirect) | 0 |
| | Census 1996 (Indirect) | 1 |
| | Census 1996 (Household Deaths) | 0 |
| | Demographic and Health Survey 1999 (Direct) | 1 |

| Country | Data series | Inclusion |
|---|---|---|
| | Demographic and Health Survey 1999 (Indirect) | 0 |
| | Multiple Indicator Cluster Survey 2003 (Indirect) | 1 |
| | Demographic and Health Survey 2005 (Direct) | 1 |
| | Demographic and Health Survey 2005 (Indirect) | 0 |
| | Demographic and Health Survey 2012 (Direct) | 1 |
| | Census 2014 (Household Deaths) | 0 |
| | Census 2014 (Indirect) | 1 |
| | Multiple Indicator Cluster Survey 2016 (Direct) | 0 |
| | Demographic and Health Survey 2018 (Direct) | 1 |
| | Malaria Indicator Survey 2021 (Indirect) | 1 |
| Gambia | Census 1973 (Indirect) | 1 |
| | Census 1983 (Indirect) | 1 |
| | Census 1993 (Indirect) | 1 |
| | Multiple Indicator Cluster Survey 2000 (Indirect) | 1 |
| | Census 2003 (Indirect) | 1 |
| | Multiple Indicator Cluster Survey 2005-2006 (Indirect) | 1 |
| | Multiple Indicator Cluster Survey 2010 (Indirect) | 1 |
| | Census 2013 (Indirect) | 0 |
| | Demographic and Health Survey 2013 (Direct) | 0 |
| | Multiple Indicator Cluster Survey 2018 (Direct) | 1 |
| | Demographic and Health Survey 2019-2020 (Direct) | 1 |
| Guinea-Bissau | Census 1950 (Indirect) | 0 |
| | Multiple Indicator Cluster Survey 2000 (Indirect) | 1 |
| | Multiple Indicator Cluster Survey 2006 (Indirect) | 1 |
| | Census 2009 (Household Deaths) | 0 |
| | Multiple Indicator Cluster Survey 2010 (Indirect) | 0 |
| | Multiple Indicator Cluster Survey 2010 (Direct) | 1 |
| | Multiple Indicator Cluster Survey 2014 (Direct) | 1 |
| | Multiple Indicator Cluster Survey 2018-2019 (Direct) | 0 |
| Equatorial Guinea | Census 1983 (Household Deaths) | 1 |
| | Census 1994 (Household Deaths) | 1 |
| | Multiple Indicator Cluster Survey 2000 (Indirect) | 1 |
| | Census 2001 (Household Deaths) | 1 |

| Country | Data series | Inclusion |
| --- | --- | --- |
| | Demographic and Health Survey 2011 (Direct) | 1 |
| Greece | UNPD Vital Registration Data 2022 version 2022 (VR) | 1 |
| | WHO Vital Registration Data 2025 version 2025 (VR) | 1 |
| Grenada | UNPD Vital Registration Data 2022 version 2022 (VR) | 1 |
| | Recalculated WHO Vital Registration Data 2025 version 2025 (VR) | 1 |
| | WHO Vital Registration Data 2025 version 2025 (VR) | 0 |
| Guatemala | Census 1973 (Indirect) | 1 |
| | Encuesta Nacional de Fecundidad, Planificacion Familiar y Comunicacion 1978-1979 (Indirect) | 1 |
| | Census 1981 (Indirect) | 1 |
| | Enc. Nacional SocioDemografica 1987 (Indirect) | 1 |
| | Demographic and Health Survey 1987 (Direct) | 1 |
| | Demographic and Health Survey 1987 (Indirect) | 0 |
| | Enc. Nacional Sociodemografica 1989 (Indirect) | 1 |
| | Census 1994 (Indirect) | 1 |
| | Demographic and Health Survey 1995 (Direct) | 1 |
| | Demographic and Health Survey 1995 (Indirect) | 0 |
| | Demographic and Health Survey 1998-1999 (Direct) | 1 |
| | Demographic and Health Survey 1998-1999 (Indirect) | 0 |
| | Census 2002 (Indirect) | 1 |
| | Encuesta Nacional de Salud Materno Infantil (ENSMI) 2002 (Direct) | 1 |
| | Encuesta Nacional de Salud Materno Infantil (ENSMI) 2002 (Indirect) | 0 |
| | Encuesta Nacional de Salud Materno Infantil (ENSMI) 2008-2009 (Direct) | 1 |
| | Demographic and Health Survey 2014-2015 (Direct) | 1 |
| | Census 2018 (Indirect) | 1 |
| | UNPD Vital Registration Data 2022 version 2022 (VR) | 0 |
| | WHO Vital Registration Data 2025 version 2025 (VR) | 0 |
| Guyana | World Fertility Survey 1975 (Direct) | 1 |
| | World Fertility Survey 1975 (Indirect) | 0 |
| | Multiple Indicator Cluster Survey 2000-2001 (Indirect) | 1 |
| | Census 2002 (Indirect) | 1 |
| | AIDS Indicator Survey 2005 (Direct) | 1 |
| | AIDS Indicator Survey 2005 (Indirect) | 0 |
| | Multiple Indicator Cluster Survey 2006 (Indirect) | 1 |

| Country | Data series | Inclusion |
|---|---|---|
| | Demographic and Health Survey 2009 (Direct) | 1 |
| | Census 2012 (Household Deaths) | 0 |
| | Multiple Indicator Cluster Survey 2014 (Direct) | 1 |
| | Multiple Indicator Cluster Survey 2019-2020 (Direct) | 0 |
| | WHO Vital Registration Data 2025 version 2025 (VR) | 0 |
| Honduras | Encuesta Demografica Nacional 1972 (Direct) | 1 |
| | Encuesta Demografica Nacional 1972 (Household Deaths) | 0 |
| | Encuesta Demografica Nacional 1972 (Indirect) | 1 |
| | Census 1974 (Indirect) | 1 |
| | National Demographic Survey 1983 (Indirect) | 1 |
| | National Survey of Maternal and Child Health 1984 (Indirect) | 1 |
| | National Survey of Epidemiology and Family Health 1987 (Direct) | 1 |
| | National Survey of Epidemiology and Family Health 1987 (Indirect) | 0 |
| | Census 1988 (Indirect) | 1 |
| | National Survey of Epidemiology and Family Health 1991-1992 (Direct) | 1 |
| | National Survey of Epidemiology and Family Health 1991-1992 (Indirect) | 0 |
| | National Survey of Epidemiology and Family Health 1996 (Direct) | 1 |
| | National Survey of Epidemiology and Family Health 1996 (Indirect) | 0 |
| | Census 2001 (Household Deaths) | 0 |
| | Census 2001 (Indirect) | 1 |
| | National Survey of Epidemiology and Family Health 2001 (Direct) | 1 |
| | Demographic and Health Survey 2005-2006 (Direct) | 1 |
| | Demographic and Health Survey 2005-2006 (Indirect) | 0 |
| | Demographic and Health Survey 2011-2012 (Direct) | 1 |
| | Census 2013 (Household Deaths) | 0 |
| | Census 2013 (Indirect) | 1 |
| | Multiple Indicator Cluster Survey 2019 (Direct) | 1 |
| | WHO Vital Registration Data 2025 version 2025 (VR) | 0 |
| Croatia | Census 2011 (Household Deaths) | 0 |
| | WHO Vital Registration Data 2025 version 2025 (VR) | 1 |
| Haiti | Census 1971 (Direct) | 0 |
| | Census 1971 (Household Deaths) | 0 |
| | World Fertility Survey 1977 (Direct) | 1 |

| Country | Data series | Inclusion |
|---|---|---|
| | World Fertility Survey 1977 (Indirect) | 0 |
| | Census 1982 (Indirect) | 1 |
| | Contraceptive Prevalence Survey 1983 (Indirect) | 1 |
| | Mortality, morbidity and service utilization survey 1987 (Direct) | 1 |
| | Mortality, morbidity and service utilization survey 1987 (Indirect) | 0 |
| | Demographic and Health Survey 1994-1995 (Direct) | 1 |
| | Demographic and Health Survey 1994-1995 (Indirect) | 0 |
| | Demographic and Health Survey 2000 (Direct) | 1 |
| | Demographic and Health Survey 2000 (Indirect) | 0 |
| | Census 2003 (Household Deaths) | 0 |
| | Census 2003 (Indirect) | 0 |
| | Demographic and Health Survey 2005-2006 (Direct) | 1 |
| | Demographic and Health Survey 2005-2006 (Indirect) | 0 |
| | Demographic and Health Survey 2012 (Direct) | 1 |
| | Demographic and Health Survey 2016-2017 (Direct) | 1 |
| | WHO Vital Registration Data 2025 version 2025 (VR) | 0 |
| Hungary | UNPD Vital Registration Data 2022 version 2022 (VR) | 1 |
| | WHO Vital Registration Data 2025 version 2025 (VR) | 1 |
| Indonesia | National Socio-economic Survey 1964-1965 (Household Deaths) | 0 |
| | Census 1971 (Indirect) | 1 |
| | Intercensal Survey 1976 (Indirect) | 0 |
| | World Fertility Survey 1976 (Direct) | 1 |
| | World Fertility Survey 1976 (Indirect) | 0 |
| | Census 1980 (Indirect) | 1 |
| | Demographic and Health Survey 1987 (Direct) | 1 |
| | Demographic and Health Survey 1987 (Indirect) | 0 |
| | Census 1990 (Indirect) | 1 |
| | Demographic and Health Survey 1991 (Direct) | 1 |
| | Demographic and Health Survey 1991 (Indirect) | 0 |
| | Demographic and Health Survey 1994 (Direct) | 1 |
| | Demographic and Health Survey 1994 (Indirect) | 0 |
| | Intercensal Survey 1995 (Indirect) | 0 |
| | Demographic and Health Survey 1997 (Direct) | 1 |

| Country | Data series | Inclusion |
|---|---|---|
| | Demographic and Health Survey 1997 (Indirect) | 0 |
| | Census 2000 (Indirect) | 1 |
| | National Socio-Economic Survey (SUSENAS) 2003 (Indirect) | 1 |
| | Demographic and Health Survey 2002-2003 (Direct) | 1 |
| | Demographic and Health Survey 2002-2003 (Indirect) | 0 |
| | National Socio-Economic Survey (SUSENAS) 2004 (Indirect) | 1 |
| | National Socio-Economic Survey (SUSENAS) 2005 (Indirect) | 1 |
| | National Socio-Economic Survey (SUSENAS) 2006 (Indirect) | 1 |
| | National Socio-Economic Survey (SUSENAS) 2007 (Indirect) | 1 |
| | Demographic and Health Survey 2007 (Direct) | 1 |
| | Demographic and Health Survey 2007 (Indirect) | 0 |
| | National Socio-Economic Survey (SUSENAS) 2008 (Indirect) | 1 |
| | National Socio-Economic Survey (SUSENAS) 2009 (Indirect) | 1 |
| | Census 2010 (Household Deaths) | 0 |
| | Census 2010 (Indirect) | 1 |
| | National Socio-Economic Survey (SUSENAS) 2010 (Indirect) | 1 |
| | Demographic and Health Survey 2012 (Direct) | 1 |
| | Report on Sample Vital Registration System 2014 2014 (VR) | 0 |
| | Demographic and Health Survey 2017 (Direct) | 1 |
| | Population Census Long Form 2020 (Household Deaths) | 1 |
| India | Provincial Annual Sanitary-Health Reports 1947 (VR) | 0 |
| | National Sample Survey 1966 (Indirect) | 1 |
| | National Family Planning Survey 1970 (Indirect) | 1 |
| | National Fertility Survey 1972 (Indirect) | 1 |
| | Survey on Infant and Child Mortality 1979 (Indirect) | 0 |
| | Second All-India Family Planning Survey 1981 (Indirect) | 1 |
| | Census 1981 (Indirect) | 0 |
| | Census 1991 (Indirect) | 0 |
| | National Family Health Survey 1992-1993 (Direct) | 1 |
| | National Family Health Survey 1992-1993 (Indirect) | 0 |
| | District Level Household Survey 1999 (Indirect) | 1 |
| | National Family Health Survey 1998-1999 (Direct) | 1 |
| | National Family Health Survey 1998-1999 (Indirect) | 0 |

| Country | Data series | Inclusion |
|---|---|---|
| | Census 2001 (Indirect) | 0 |
| | District Level Household Survey 2004 (Indirect) | 1 |
| | National Family Health Survey 2005-2006 (Direct) | 1 |
| | National Family Health Survey 2005-2006 (Indirect) | 0 |
| | District Level Household Survey 2008 (Indirect) | 1 |
| | Census 2011 (Indirect) | 1 |
| | National Family Health Survey 2015-2016 (Direct) | 0 |
| | National Family Health Survey 2019-2021 (Direct) | 0 |
| | Sample Registration System 2023 (VR) | 1 |
| Ireland | Census 2011 (Household Deaths) | 0 |
| | WHO Vital Registration Data 2025 version 2025 (VR) | 1 |
| Iran (Islamic Republic of) | Population Growth Survey 1973-1976 (Direct) | 1 |
| | Population Growth Survey 1973-1976 (Indirect) | 0 |
| | Census 1986 (Indirect) | 1 |
| | Infant and Child Mortality Survey 1989 (Direct) | 1 |
| | Infant and Child Mortality Survey 1989 (Indirect) | 0 |
| | Intercensal Population Survey 1991 (Indirect) | 1 |
| | Iran National MICS 1995 (Household Deaths) | 1 |
| | Census 1996 (Indirect) | 1 |
| | Demographic Survey 1998 (Indirect) | 1 |
| | Iran National MICS 1998 (Household Deaths) | 1 |
| | Demographic and Health Survey 2000 (Direct) | 1 |
| | Demographic and Health Survey 2000 (Indirect) | 0 |
| | Census 2006 (Indirect) | 1 |
| | Iran National Child Mortality Surveillance System 2008 (VR) | 1 |
| | National Multiple-Indicator Demographic and Health Survey 2010 (Direct) | 1 |
| | Census 2011 (Indirect) | 1 |
| | Census 2016 (Indirect) | 0 |
| | UNPD Demographic Yearbook Data 2022 version 2022 (VR) | 0 |
| | WHO Vital Registration Data 2025 version 2025 (VR) | 0 |
| Iraq | Census 1957 (Indirect) | 1 |
| | Census 1965 (Household Deaths) | 0 |
| | Demographic Sample Survey and Sample Registration System 1973 (Direct) | 1 |

| Country | Data series | Inclusion |
|---|---|---|
| | Fertility Survey 1974 (Indirect) | 1 |
| | Census 1987 (Indirect) | 1 |
| | Gulf Child Health Survey 1989 (Direct) | 0 |
| | Gulf Child Health Survey 1989 (Indirect) | 1 |
| | Immunization, Diarrhoeal Disease, Maternal and Child Mortality Survey 1990 (Indirect) | 1 |
| | Infant and Child Mortality and Nutrition Survey 1991 (Direct) | 0 |
| | Census 1997 (Indirect) | 0 |
| | Child and Maternal Mortality Survey 1999 (Direct) | 0 |
| | Living Conditions Survey 2004 (Direct) | 1 |
| | Iraq Family Health Survey 2006 (Indirect) | 1 |
| | Multiple Indicator Cluster Survey 2006 (Direct) | 1 |
| | Multiple Indicator Cluster Survey 2006 (Indirect) | 0 |
| | Multiple Indicator Cluster Survey 2011 (Direct) | 1 |
| | Multiple Indicator Cluster Survey 2018 (Direct) | 1 |
| | WHO Vital Registration Data 2025 version 2025 (VR) | 0 |
| Iceland | UNPD Vital Registration Data 2022 version 2022 (VR) | 1 |
| | Recalculated WHO Vital Registration Data 2025 version 2025 (VR) | 1 |
| | WHO Vital Registration Data 2025 version 2025 (VR) | 0 |
| Israel | National Life table 1969-1988 (Life Table) | 0 |
| | UNPD Vital Registration Data 2022 version 2022 (VR) | 1 |
| | WHO Vital Registration Data 2025 version 2025 (VR) | 1 |
| Italy | WHO Vital Registration Data 2025 version 2025 (VR) | 1 |
| Jamaica | National Life Tables (Life Table) | 1 |
| | World Fertility Survey 1975-1976 (Direct) | 1 |
| | World Fertility Survey 1975-1976 (Indirect) | 0 |
| | Census 1982 (Indirect) | 1 |
| | Contraceptive Prevalence Survey 1989 (Direct) | 0 |
| | Multiple Indicator Cluster Survey 2000 (Indirect) | 0 |
| | Census 2001 (Indirect) | 0 |
| | Multiple Indicator Cluster Survey 2005 (Indirect) | 1 |
| | Reproductive Health Survey 2008-2009 (Direct) | 1 |
| | Census 2011 (Indirect) | 1 |
| | UNPD Vital Registration Data 2022 version 2022 (VR) | 0 |

| Country | Data series | Inclusion |
|---|---|---|
| | Reproductive Health Survey 2021-2022 (Direct) | 0 |
| | WHO Vital Registration Data 2025 version 2025 (VR) | 1 |
| Jordan | Census 1961 (Indirect) | 1 |
| | Jordan Fertility Survey 1972 (Indirect) | 1 |
| | World Fertility Survey 1976 (Direct) | 1 |
| | World Fertility Survey 1976 (Indirect) | 0 |
| | Census 1979 (Indirect) | 1 |
| | Jordan Demographic Survey 1981 (Indirect) | 1 |
| | EPI/CDD and Child Mortality Survey 1988 (Indirect) | 1 |
| | EPI/CDD and Child Mortality Survey 1990 (Indirect) | 1 |
| | Demographic and Health Survey 1990 (Direct) | 1 |
| | Demographic and Health Survey 1990 (Indirect) | 0 |
| | Demographic and Health Survey 1997 (Direct) | 1 |
| | Demographic and Health Survey 1997 (Indirect) | 0 |
| | Annual Fertility Survey 1999 (Indirect) | 1 |
| | Demographic and Health Survey 2002 (Direct) | 1 |
| | Demographic and Health Survey 2002 (Indirect) | 0 |
| | Demographic and Health Survey 2007 (Direct) | 1 |
| | Demographic and Health Survey 2007 (Indirect) | 0 |
| | Demographic and Health Survey 2009 (Direct) | 1 |
| | Demographic and Health Survey 2009 (Indirect) | 0 |
| | Demographic and Health Survey 2012 (Direct) | 1 |
| | Census 2015 (Household Deaths) | 0 |
| | Demographic and Health Survey 2017 (Direct) | 1 |
| | Demographic and Health Survey 2023 (Direct) | 1 |
| | WHO Vital Registration Data 2025 version 2025 (VR) | 0 |
| Japan | WHO Vital Registration Data 2025 version 2025 (VR) | 1 |
| Kazakhstan | Vital Registration Data from The Agency of Statistics of Kazakhstan  (VR) | 0 |
| | Census 1989 (Indirect) | 1 |
| | Demographic and Health Survey 1995 (Direct) | 1 |
| | Demographic and Health Survey 1995 (Indirect) | 0 |
| | Isms 1996 (Indirect) | 1 |
| | Census 1999 (Indirect) | 0 |

| Country | Data series | Inclusion |
| --- | --- | --- |
| | Demographic and Health Survey 1999 (Direct) | 1 |
| | Demographic and Health Survey 1999 (Indirect) | 0 |
| | Multiple Indicator Cluster Survey 2006 (Indirect) | 1 |
| | Transmonee Vital Registration Data 2008 version 2008 (VR) | 0 |
| | Census 2009 (Indirect) | 1 |
| | Multiple Indicator Cluster Survey 2010-2011 (Indirect) | 1 |
| | Multiple Indicator Cluster Survey 2015 (Indirect) | 1 |
| | Multiple Indicator Cluster Survey 2024 (Direct) | 0 |
| | WHO Vital Registration Data 2025 version 2025 (VR) | 1 |
| Kenya | Census 1969 (Indirect) | 1 |
| | Demographic Survey 1977 (Indirect) | 1 |
| | World Fertility Survey 1977-1978 (Direct) | 1 |
| | World Fertility Survey 1977-1978 (Indirect) | 0 |
| | Census 1979 (Indirect) | 1 |
| | Demographic Survey 1983 (Indirect) | 1 |
| | Census 1989 (Indirect) | 1 |
| | Demographic and Health Survey 1989 (Direct) | 0 |
| | Demographic and Health Survey 1989 (Indirect) | 0 |
| | Demographic and Health Survey (MM adjusted) 1989 (Direct) | 1 |
| | Demographic and Health Survey 1993 (Direct) | 0 |
| | Demographic and Health Survey 1993 (Indirect) | 0 |
| | Demographic and Health Survey (MM adjusted) 1993 (Direct) | 1 |
| | Demographic and Health Survey 1998 (Direct) | 0 |
| | Demographic and Health Survey 1998 (Indirect) | 0 |
| | Demographic and Health Survey (MM adjusted) 1998 (Direct) | 1 |
| | Census 1999 (Indirect) | 1 |
| | Multiple Indicator Cluster Survey 2000 (Indirect) | 1 |
| | Demographic and Health Survey 2003 (Direct) | 0 |
| | Demographic and Health Survey 2003 (Indirect) | 0 |
| | Demographic and Health Survey (MM adjusted) 2003 (Direct) | 1 |
| | Demographic and Health Survey 2008-2009 (Direct) | 0 |
| | Demographic and Health Survey 2008-2009 (Indirect) | 0 |
| | Demographic and Health Survey (MM adjusted) 2008-2009 (Direct) | 1 |

| Country | Data series | Inclusion |
|---|---|---|
| | Census 2009 (Household Deaths) | 0 |
| | Demographic and Health Survey 2014 (Direct) | 0 |
| | Demographic and Health Survey (MM adjusted) 2014 (Direct) | 1 |
| | Malaria Indicator Survey 2015 (Indirect) | 1 |
| | Census 2019 (Indirect) | 0 |
| | Census 2019 (Household Deaths) | 1 |
| | Malaria Indicator Survey 2020 (Indirect) | 1 |
| | Demographic and Health Survey 2022 (Direct) | 0 |
| | Demographic and Health Survey (MM adjusted) 2022 (Direct) | 1 |
| Kyrgyzstan | Census 1989 (Indirect) | 0 |
| | Demographic and Health Survey 1997 (Direct) | 1 |
| | Demographic and Health Survey 1997 (Indirect) | 0 |
| | Census 1999 (Indirect) | 0 |
| | Multiple Indicator Cluster Survey 2005-2006 (Indirect) | 1 |
| | Census 2009 (Indirect) | 0 |
| | Demographic and Health Survey 2012 (Direct) | 1 |
| | Multiple Indicator Cluster Survey 2014 (Direct) | 1 |
| | Multiple Indicator Cluster Survey 2018 (Direct) | 1 |
| | Multiple Indicator Cluster Survey 2023 (Direct) | 1 |
| | WHO Vital Registration Data 2025 version 2025 (VR) | 1 |
| Cambodia | Survey 1959 (Household Deaths) | 0 |
| | Census extracted from IPUMS 1962 (Indirect) | 0 |
| | Census 1998 (Indirect) | 1 |
| | National Health Survey 1998 (Direct) | 0 |
| | National Health Survey 1998 (Indirect) | 1 |
| | Demographic and Health Survey 2000 (Direct) | 1 |
| | Demographic and Health Survey 2000 (Indirect) | 0 |
| | Inter-censal Population Survey 2004 (Indirect) | 1 |
| | Demographic and Health Survey 2005 (Direct) | 1 |
| | Demographic and Health Survey 2005 (Indirect) | 0 |
| | Census extracted from IPUMS 2008 (Household Deaths) | 0 |
| | Census 2008 (Indirect) | 0 |
| | Demographic and Health Survey 2010 (Direct) | 1 |

| Country | Data series | Inclusion |
|---|---|---|
| | Intercensal Population Survey 2013 (Indirect) | 0 |
| | Demographic and Health Survey 2014 (Direct) | 1 |
| | Census 2019 (Indirect) | 1 |
| | Demographic and Health Survey 2021-2022 (Direct) | 0 |
| Kiribati | Census 1963 (Indirect) | 1 |
| | Census 1968 (Indirect) | 1 |
| | Census 1973 (Indirect) | 1 |
| | Census 1978 (Indirect) | 1 |
| | Census 1985 (Indirect) | 1 |
| | Census 1990 (Indirect) | 1 |
| | Census 1995 (Indirect) | 1 |
| | Census 2000 (Indirect) | 1 |
| | Census 2005 (Indirect) | 1 |
| | SPC Demographic and Health Survey 2009 (Direct) | 1 |
| | Census 2010 (Household Deaths) | 0 |
| | Census 2010 (Indirect) | 1 |
| | Multiple Indicator Cluster Survey 2018-2019 (Direct) | 0 |
| | Census 2020 (Indirect) | 1 |
| | WHO Vital Registration Data 2025 version 2025 (VR) | 0 |
| Saint Kitts and Nevis | Recalculated WHO Vital Registration Data 2025 version 2025 (VR) | 1 |
| | WHO Vital Registration Data 2025 version 2025 (VR) | 0 |
| Republic of Korea | National Life Table  (Life Table) | 0 |
| | National Life Table 1970-2015  (Life Table) | 1 |
| | Census 1970 (Indirect) | 1 |
| | World Fertility Survey 1974 (Direct) | 1 |
| | World Fertility Survey 1974 (Indirect) | 0 |
| | Census 1975 (Indirect) | 1 |
| | Census 1980 (Indirect) | 1 |
| | Census 1985 (Indirect) | 1 |
| | Census 1990 (Indirect) | 1 |
| | WHO Vital Registration Data 2025 version 2025 (VR) | 1 |
| Kuwait | National Life Tables  (Life Table) | 1 |
| | Census 1975 (Indirect) | 1 |

| Country | Data series | Inclusion |
| --- | --- | --- |
| | Census 1980 (Indirect) | 1 |
| | Child Health Survey 1987 (Indirect) | 1 |
| | Family Health Survey 1996 (Direct) | 0 |
| | Family Health Survey 1996 (Indirect) | 0 |
| | UNPD Vital Registration Data 2022 version 2022 (VR) | 0 |
| | WHO Vital Registration Data 2025 version 2025 (VR) | 1 |
| Lao People's Democratic Republic | Social Indicator Survey 1993 (Indirect) | 1 |
| | Fertility and Birth Spacing Survey 1994 (Direct) | 1 |
| | Census 1995 (Indirect) | 1 |
| | Reproductive Health Survey 2000 (Direct) | 1 |
| | Census 2005 (Indirect) | 1 |
| | Reproductive Health Survey 2005 (Indirect) | 0 |
| | Reproductive Health Survey 2005 (Direct) | 1 |
| | Lao Social Indicator Survey (combined MICS4/DHS) 2012 (Direct) | 1 |
| | Census 2015 (Direct) | 0 |
| | Lao Social Indicator Survey II 2017 (Direct) | 1 |
| | Lao Social Indicator Survey III 2023 (Direct) | 1 |
| Lebanon | National Fertility and Family Planning Survey 1971 (Indirect) | 1 |
| | National EPI CDD IMR Survey 1990 (Indirect) | 1 |
| | Maternal and Child Health Survey 1996 (Direct) | 1 |
| | Maternal and Child Health Survey 1996 (Indirect) | 1 |
| | Population and Housing Survey 1996 (Indirect) | 1 |
| | Multiple Indicator Cluster Survey 2000 (Indirect) | 1 |
| | PAPFAM Family Health Survey 2004 (Direct) | 1 |
| | Multiple Indicator Cluster Survey 2009 (Indirect) | 1 |
| | Health Facility Data from Ministry of Public Health 2025 (VR) | 1 |
| | WHO Vital Registration Data 2025 version 2025 (VR) | 0 |
| Liberia | Population Growth Survey 1969-1970 (Direct) | 0 |
| | Population Growth Survey 1969-1970 (Indirect) | 0 |
| | Population Growth Survey 1970-1971 (Direct) | 1 |
| | Population Growth Survey 1970-1971 (Indirect) | 1 |
| | Census 1974 (Indirect) | 0 |
| | Demographic and Health Survey 1986 (Direct) | 1 |

| Country | Data series | Inclusion |
|---|---|---|
| | Demographic and Health Survey 1986 (Indirect) | 0 |
| | LDHS 1999-2000 (Direct) | 0 |
| | Demographic and Health Survey 2006-2007 (Direct) | 1 |
| | Demographic and Health Survey 2006-2007 (Indirect) | 0 |
| | Population and Housing Census 2008 (Household Deaths) | 0 |
| | Population and Housing Census 2008 (Indirect) | 0 |
| | Malaria Indicator Survey 2008-2009 (Direct) | 1 |
| | Malaria Indicator Survey 2008-2009 (Indirect) | 0 |
| | Malaria Indicator Survey 2011 (Indirect) | 1 |
| | Demographic and Health Survey 2013 (Direct) | 1 |
| | Malaria Indicator Survey 2016 (Indirect) | 1 |
| | Demographic and Health Survey 2019-2020 (Direct) | 1 |
| | Malaria Indicator Survey 2022 (Indirect) | 1 |
| | Census 2022 (Household Deaths) | 1 |
| Libya | Census 1973 (Indirect) | 1 |
| | PAPCHILD Maternal and Child Health Survey 1995 (Direct) | 1 |
| | Multiple Indicator Cluster Survey 2003 (Indirect) | 1 |
| | PAPFAM Family Health Survey 2007 (Direct) | 1 |
| | VR Data from Health and Environment Report 2009 2009 (VR) | 1 |
| | PAPFAM Family Health Survey 2014 (Direct) | 0 |
| | Multiple Indicator Cluster Survey 2024-2025 (Direct) | 0 |
| Saint Lucia | UNPD Vital Registration Data 2022 version 2022 (VR) | 1 |
| | Recalculated WHO Vital Registration Data 2025 version 2025 (VR) | 1 |
| | WHO Vital Registration Data 2025 version 2025 (VR) | 0 |
| Sri Lanka | National life table (Life Table) | 1 |
| | Census 1971 (Indirect) | 1 |
| | World Fertility Survey 1975 (Direct) | 1 |
| | World Fertility Survey 1975 (Indirect) | 0 |
| | Demographic and Health Survey 1987 (Direct) | 1 |
| | Demographic and Health Survey 1987 (Indirect) | 0 |
| | Demographic and Health Survey 1993 (Direct) | 1 |
| | Demographic and Health Survey 2000 (Direct) | 1 |
| | Census 2001 (Indirect) | 1 |

| Country | Data series | Inclusion |
|---|---|---|
| | Demographic and Health Survey 2006 (Indirect) | 0 |
| | Demographic and Health Survey 2006 (Direct) | 1 |
| | Census 2012 (Indirect) | 1 |
| | Demographic and Health Survey 2016 (Direct) | 1 |
| | Civil Registration Data from Department of Census and Statistics 2023 (VR) | 1 |
| | WHO Vital Registration Data 2025 version 2025 (VR) | 1 |
| Lesotho | ces 1968 (Indirect) | 1 |
| | Demographic Survey 1971-1973 (Indirect) | 1 |
| | Demographic Survey 1971-1973 (Household Deaths) | 0 |
| | Census 1976 (Indirect) | 1 |
| | World Fertility Survey 1977 (Household Deaths) | 0 |
| | World Fertility Survey 1977 (Direct) | 1 |
| | World Fertility Survey 1977 (Indirect) | 0 |
| | Census 1986 (Indirect) | 1 |
| | Census 1986 (Household Deaths) | 0 |
| | Census 1996 (Indirect) | 1 |
| | Census 1996 (Household Deaths) | 0 |
| | Multiple Indicator Cluster Survey 2000 (Indirect) | 1 |
| | Demographic Survey 2001 (Household Deaths) | 0 |
| | Demographic Survey 2001 (Indirect) | 1 |
| | Demographic and Health Survey 2004 (Direct) | 0 |
| | Demographic and Health Survey 2004 (Indirect) | 0 |
| | Demographic and Health Survey (MM adjusted) 2004 (Direct) | 1 |
| | Census 2006 (Household Deaths) | 0 |
| | Census 2006 (Indirect) | 1 |
| | Demographic and Health Survey 2009 (Direct) | 0 |
| | Demographic and Health Survey 2009 (Indirect) | 0 |
| | Demographic and Health Survey (MM adjusted) 2009 (Direct) | 1 |
| | Demographic Survey 2011 (Indirect) | 0 |
| | Demographic and Health Survey 2014 (Direct) | 0 |
| | Demographic and Health Survey (MM adjusted) 2014 (Direct) | 1 |
| | Census 2016 (Household Deaths) | 1 |
| | Multiple Indicator Cluster Survey 2018 (Direct) | 0 |

| Country | Data series | Inclusion |
|---|---|---|
| | Multiple Indicator Cluster Survey (MM adjusted) 2018 (Direct) | 1 |
| | Demographic Survey 2021 (Indirect) | 0 |
| | Demographic and Health Survey 2023-2024 (Direct) | 0 |
| | Demographic and Health Survey (MM adjusted) 2023-2024 (Direct) | 1 |
| Lithuania | Census 2011 (Household Deaths) | 0 |
| | HMD Vital Registration Data 2025 (VR) | 1 |
| | Recalculated WHO Vital Registration Data 2025 version 2025 (VR) | 1 |
| | WHO Vital Registration Data 2025 version 2025 (VR) | 0 |
| Luxembourg | UNPD Vital Registration Data 2022 version 2022 (VR) | 1 |
| | Recalculated WHO Vital Registration Data 2025 version 2025 (VR) | 1 |
| | WHO Vital Registration Data 2025 version 2025 (VR) | 0 |
| Latvia | HMD Vital Registration Data 2025 (VR) | 1 |
| | Recalculated WHO Vital Registration Data 2025 version 2025 (VR) | 1 |
| | WHO Vital Registration Data 2025 version 2025 (VR) | 0 |
| Morocco | World Fertility Survey 1980 (Direct) | 1 |
| | World Fertility Survey 1980 (Indirect) | 0 |
| | Census 1982 (Indirect) | 1 |
| | Contraceptive Prevalence Survey 1983 (Indirect) | 1 |
| | Demographic and Health Survey 1987 (Direct) | 1 |
| | Demographic and Health Survey 1987 (Indirect) | 0 |
| | Demographic and Health Survey 1992 (Direct) | 1 |
| | Demographic and Health Survey 1992 (Indirect) | 0 |
| | Census 1994 (Indirect) | 1 |
| | Demographic and Health Survey 1995 (Direct) | 1 |
| | Demographic and Health Survey 1995 (Indirect) | 0 |
| | PAPG ENSME grand-echantillon 1996 (Direct) | 0 |
| | PAPCHILD Maternal and Child Health Survey 1997 (Direct) | 1 |
| | Census 2004 (Indirect) | 1 |
| | Demographic and Health Survey 2003-2004 (Direct) | 1 |
| | Demographic and Health Survey 2003-2004 (Indirect) | 0 |
| | National Demographic Survey with repeated passages 2009-2010 (Household Deaths) | 1 |
| | National Survey on Population and Family Health 2011 (Direct) | 1 |
| | Census 2014 (Indirect) | 1 |

| Country | Data series | Inclusion |
| --- | --- | --- |
| | National Survey on Population and Family Health 2018 (Direct) | 1 |
| | WHO Vital Registration Data 2025 version 2025 (VR) | 0 |
| Monaco | WHO Vital Registration Data 2018 version neighbouring 2025 (VR) | 1 |
| | WHO Vital Registration Data 2025 version 2025 (VR) | 0 |
| Republic of Moldova | Census 1989 (Indirect) | 1 |
| | Census 2004 (Indirect) | 0 |
| | Demographic and Health Survey 2005 (Direct) | 1 |
| | Demographic and Health Survey 2005 (Indirect) | 0 |
| | Multiple Indicator Cluster Survey 2012 (Direct) | 1 |
| | WHO Vital Registration Data 2025 version 2025 (VR) | 1 |
| Madagascar | Enquete demographique 1966 (Household Deaths) | 0 |
| | Enquete demographique 1966 (Indirect) | 0 |
| | Census 1975 (Indirect) | 0 |
| | Demographic and Health Survey 1992 (Direct) | 1 |
| | Demographic and Health Survey 1992 (Indirect) | 0 |
| | Census 1993 (Household Deaths) | 0 |
| | Multiple Indicator Cluster Survey 1995 (Indirect) | 1 |
| | Demographic and Health Survey 1997 (Direct) | 1 |
| | Demographic and Health Survey 1997 (Indirect) | 0 |
| | Multiple Indicator Cluster Survey 2000 (Indirect) | 1 |
| | Demographic and Health Survey 2003-2004 (Direct) | 1 |
| | Demographic and Health Survey 2003-2004 (Indirect) | 0 |
| | Demographic and Health Survey 2008-2009 (Direct) | 1 |
| | Demographic and Health Survey 2008-2009 (Indirect) | 0 |
| | Malaria Indicator Survey 2011 (Indirect) | 1 |
| | National Survey on Monitoring the MDGs (ENSOMD) 2012-2013 (Direct) | 0 |
| | Malaria Indicator Survey 2013 (Indirect) | 1 |
| | Malaria Indicator Survey 2016 (Indirect) | 1 |
| | Multiple Indicator Cluster Survey 2018 (Direct) | 1 |
| | Enquete Demographique et de Sante (EDSMDV) 2021 (Direct) | 1 |
| Maldives | Census 1977 (Indirect) | 1 |
| | Census 1985 (Indirect) | 1 |
| | Census 1990 (Indirect) | 1 |

| Country | Data series | Inclusion |
| --- | --- | --- |
| | Census 1995 (Indirect) | 1 |
| | Poverty and Vulnerability Survey 1997 (Indirect) | 1 |
| | Census 2000 (Indirect) | 1 |
| | Vulnerability and Poverty Assessment 2004 (Indirect) | 1 |
| | Vital Registration Data from Statistical Yearbook of Maldives 2005 version 2005 (VR) | 0 |
| | Census 2006 (Indirect) | 1 |
| | Demographic and Health Survey 2009 (Direct) | 1 |
| | Demographic and Health Survey 2009 (Indirect) | 0 |
| | Vital Statistics 2012 (VR) | 0 |
| | Demographic and Health Survey 2016-2017 (Direct) | 1 |
| | Vital Statistics Report of Maldives 2024 (VR) | 0 |
| | Recalculated WHO Vital Registration Data 2025 version 2025 (VR) | 1 |
| | WHO Vital Registration Data 2025 version 2025 (VR) | 0 |
| Mexico | World Fertility Survey 1976 (Indirect) | 0 |
| | World Fertility Survey 1976 (Direct) | 1 |
| | Contraceptive Prevalence Survey 1979 (Indirect) | 1 |
| | Census 1980 (Indirect) | 1 |
| | Demographic and Health Survey 1987 (Direct) | 1 |
| | Demographic and Health Survey 1987 (Indirect) | 0 |
| | Census 1990 (Indirect) | 1 |
| | Encuesta Nacional de la Dinamica Demografica (ENADID) 1992 (Direct) | 1 |
| | Encuesta Nacional de la Dinamica Demografica (ENADID) 1992 (Indirect) | 0 |
| | Encuesta Nacional de la Dinamica Demografica (ENADID) 1997 (Direct) | 1 |
| | Census 2000 (Indirect) | 1 |
| | Conteo 2005 (Indirect) | 1 |
| | Encuesta Nacional de la Dinamica Demografica (ENADID) 2006 (Indirect) | 1 |
| | Encuesta Nacional de la Dinamica Demografica (ENADID) 2009 (Direct) | 0 |
| | Census 2010 (Indirect) | 1 |
| | Encuesta Nacional de la Dinamica Demografica (ENADID) 2014 (Indirect) | 0 |
| | Encuesta Nacional de la Dinamica Demografica (ENADID) 2014 (Direct) | 1 |
| | Encuesta Intercensal 2015 (Indirect) | 1 |
| | Vital Registration Data from Mexico Ministry of Health 2018 (VR) | 1 |
| | Encuesta Nacional de la Dinamica Demografica (ENADID) 2018 (Direct) | 1 |

| Country | Data series | Inclusion |
| --- | --- | --- |
| | Encuesta Nacional de la Dinamica Demografica (ENADID) 2023 (Direct) | 1 |
| | WHO Vital Registration Data 2025 version 2025 (VR) | 0 |
| Marshall Islands | Census 1973 (Indirect) | 1 |
| | Census 1980 (Indirect) | 1 |
| | Women's Health Survey 1985 (Indirect) | 1 |
| | Census 1988 (Indirect) | 1 |
| | Census 1999 (Indirect) | 1 |
| | Demographic and Health Survey 2007 (Direct) | 1 |
| | Demographic and Health Survey 2007 (Indirect) | 0 |
| | Census 2011 (Indirect) | 0 |
| | Census 2011 (Household Deaths) | 1 |
| | WHO Vital Registration Data 2025 version 2025 (VR) | 0 |
| North Macedonia | Multiple Indicator Cluster Survey 2005 (Indirect) | 0 |
| | Multiple Indicator Cluster Survey 2018-2019 (Direct) | 0 |
| | Recalculated WHO Vital Registration Data 2025 version 2025 (VR) | 1 |
| | WHO Vital Registration Data 2025 version 2025 (VR) | 0 |
| Mali | Survey 1957-1958 (Indirect) | 0 |
| | Survey 1960-1961 (Indirect) | 0 |
| | Census 1976 (Household Deaths) | 0 |
| | Census 1987 (Indirect) | 0 |
| | Census 1987 (Household Deaths) | 0 |
| | Demographic and Health Survey 1987 (Direct) | 1 |
| | Demographic and Health Survey 1987 (Indirect) | 0 |
| | Demographic and Health Survey 1995-1996 (Direct) | 1 |
| | Demographic and Health Survey 1995-1996 (Indirect) | 0 |
| | Census 1998 (Indirect) | 0 |
| | Census 1998 (Household Deaths) | 0 |
| | Demographic and Health Survey 2001 (Direct) | 1 |
| | Demographic and Health Survey 2001 (Indirect) | 0 |
| | Demographic and Health Survey 2006 (Direct) | 1 |
| | Demographic and Health Survey 2006 (Indirect) | 0 |
| | Census 2009 (Household Deaths) | 0 |
| | Census 2009 (Indirect) | 1 |

| Country | Data series | Inclusion |
| --- | --- | --- |
| | Demographic and Health Survey 2012-2013 (Direct) | 1 |
| | Malaria Indicator Survey 2015 (Indirect) | 1 |
| | Multiple Indicator Cluster Survey 2015 (Direct) | 1 |
| | Demographic and Health Survey 2018 (Direct) | 1 |
| | Malaria Indicator Survey 2021 (Indirect) | 1 |
| | Demographic and Health Survey 2023-2024 (Direct) | 1 |
| Malta | UNPD Vital Registration Data 2022 version 2022 (VR) | 1 |
| | Recalculated WHO Vital Registration Data 2025 version 2025 (VR) | 1 |
| | WHO Vital Registration Data 2025 version 2025 (VR) | 0 |
| Myanmar | Census 1983 (Indirect) | 1 |
| | Population Change and Fertility Survey 1991 (Direct) | 1 |
| | Fertility and Reproductive Health Survey 1997 (Direct) | 1 |
| | Fertility and Reproductive Health Survey 1997 (Indirect) | 0 |
| | National Mortality Survey 1999 (Household Deaths) | 1 |
| | Fertility and Reproductive Health Survey 2001 (Direct) | 1 |
| | Fertility and Reproductive Health Survey 2001 (Indirect) | 0 |
| | Under-Five Mortality Survey 2002 (Direct) | 1 |
| | Fertility and Reproductive Health Survey 2007 (Direct) | 1 |
| | Fertility and Reproductive Health Survey 2007 (Indirect) | 0 |
| | Multiple Indicator Cluster Survey 2009-2010 (Direct) | 1 |
| | Multiple Indicator Cluster Survey 2009-2010 (Indirect) | 0 |
| | VR from Central Statistical Organization 2011 (VR) | 0 |
| | Census 2014 (Indirect) | 1 |
| | Demographic and Health Survey 2015-2016 (Direct) | 1 |
| | Inter-censal Survey 2019 (Indirect) | 0 |
| | UNPD Demographic Yearbook Data 2022 version 2022 (VR) | 0 |
| Montenegro | Vital Registration from Statistical Yearbook 2012 (VR) | 0 |
| | Recalculated WHO Vital Registration Data 2025 version 2025 (VR) | 1 |
| | WHO Vital Registration Data 2025 version 2025 (VR) | 0 |
| Mongolia | Census 1989 (Indirect) | 0 |
| | Demographic Survey 1994 (Indirect) | 1 |
| | Demographic Survey 1996 (Direct) | 1 |
| | Reproductive Health Survey 1998 (Direct) | 1 |

| Country | Data series | Inclusion |
|---|---|---|
| | Reproductive Health Survey 1998 (Indirect) | 0 |
| | Multiple Indicator Cluster Survey 2000 (Indirect) | 1 |
| | Reproductive Health Survey 2003 (Direct) | 0 |
| | Reproductive Health Survey 2003 (Indirect) | 1 |
| | Multiple Indicator Cluster Survey 2005 (Indirect) | 1 |
| | Reproductive Health Survey 2008 (Direct) | 0 |
| | Reproductive Health Survey 2008 (Indirect) | 1 |
| | Multiple Indicator Cluster Survey 2010 (Indirect) | 1 |
| | Social Indicator Sample Survey (SISS) 2013-2014 (Direct) | 1 |
| | Multiple Indicator Cluster Survey 2018 (Direct) | 0 |
| | Multiple Indicator Cluster Survey 2023 (Direct) | 0 |
| | WHO Vital Registration Data 2025 version 2025 (VR) | 1 |
| Mozambique | Census 1940 (Indirect) | 0 |
| | Census 1950 (Indirect) | 0 |
| | Census 1970 (Indirect) | 0 |
| | Census 1980 (Indirect) | 1 |
| | Mozambique National Demographic Survey 1991 (Indirect) | 1 |
| | Multiple Indicator Cluster Survey 1995 (Indirect) | 1 |
| | Census 1997 (Indirect) | 1 |
| | Demographic and Health Survey 1997 (Direct) | 0 |
| | Demographic and Health Survey 1997 (Indirect) | 0 |
| | Demographic and Health Survey (MM adjusted) 1997 (Direct) | 0 |
| | Census 1997 (Household Deaths) | 0 |
| | Demographic and Health Survey 2003-2004 (Direct) | 0 |
| | Demographic and Health Survey 2003-2004 (Indirect) | 0 |
| | Demographic and Health Survey (MM adjusted) 2003-2004 (Direct) | 1 |
| | Census 2007 (Indirect) | 1 |
| | Census 2007 (Household Deaths) | 0 |
| | Multiple Indicator Cluster Survey 2008 (Direct) | 0 |
| | Multiple Indicator Cluster Survey (MM adjusted) 2008 (Direct) | 1 |
| | Post-census Mortality Survey 2007-2008 (Direct) | 0 |
| | AIDS Indicator Survey 2009 (Indirect) | 1 |
| | Demographic and Health Survey 2011 (Direct) | 0 |

| Country | Data series | Inclusion |
|---|---|---|
| | Demographic and Health Survey (MM adjusted) 2011 (Direct) | 1 |
| | AIDS Indicator Survey 2015 (Indirect) | 1 |
| | Census 2017 (Household Deaths) | 0 |
| | Census 2017 (Indirect) | 1 |
| | Malaria Indicator Survey 2018 (Indirect) | 1 |
| | Countrywide Mortality Surveillance for Action (COMSA) 2020 (VR) | 0 |
| | Demographic and Health Survey 2022-2023 (Direct) | 0 |
| | Demographic and Health Survey (MM adjusted) 2022-2023 (Direct) | 1 |
| Mauritania | Fouta-Toro-Survey 1957 (Indirect) | 1 |
| | Survey 1964-1965 (Indirect) | 0 |
| | Census 1977 (Indirect) | 1 |
| | World Fertility Survey 1981-1982 (Direct) | 1 |
| | World Fertility Survey 1981-1982 (Indirect) | 0 |
| | Census 1988 (Indirect) | 1 |
| | Census 1988 (Household Deaths) | 0 |
| | Maternal and Child Health Survey 1990 (Direct) | 1 |
| | Maternal and Child Health Survey 1990 (Indirect) | 0 |
| | Multiple Indicator Cluster Survey 1995 (Indirect) | 0 |
| | Demographic and Health Survey 2000-2001 (Direct) | 1 |
| | Demographic and Health Survey 2000-2001 (Indirect) | 0 |
| | EMIP survey 2003-2004 (Direct) | 1 |
| | EMIP survey 2003-2004 (Indirect) | 0 |
| | Multiple Indicator Cluster Survey 2007 (Indirect) | 1 |
| | Multiple Indicator Cluster Survey 2011 (Direct) | 1 |
| | Census 2013 (Household Deaths) | 0 |
| | Census 2013 (Indirect) | 0 |
| | Multiple Indicator Cluster Survey 2015 (Direct) | 1 |
| | Demographic and Health Survey 2019-2021 (Direct) | 1 |
| Montserrat | Recalculated UNPD Vital Registration Data 2022 version 2022 (VR) | 1 |
| | UNPD Vital Registration Data 2022 version 2022 (VR) | 0 |
| Mauritius | WHO Vital Registration Data 2025 version 2025 (VR) | 1 |
| Malawi | Population Change Survey 1970-1972 (Household Deaths) | 0 |
| | Population Change Survey 1970-1972 (Direct) | 1 |

| Country | Data series | Inclusion |
|---|---|---|
| | Population Change Survey 1970-1972 (Indirect) | 0 |
| | Census 1977 (Indirect) | 1 |
| | Census 1977 (Household Deaths) | 0 |
| | Demographic Survey 1982 (Indirect) | 1 |
| | Family Formation Survey 1984 (Direct) | 1 |
| | Family Formation Survey 1984 (Household Deaths) | 0 |
| | Family Formation Survey 1984 (Indirect) | 0 |
| | Census 1987 (Indirect) | 1 |
| | Census 1987 (Household Deaths) | 0 |
| | Demographic and Health Survey 1992 (Direct) | 0 |
| | Demographic and Health Survey 1992 (Indirect) | 0 |
| | Demographic and Health Survey (MM adjusted) 1992 (Direct) | 1 |
| | Multiple Indicator Cluster Survey 1995 (Direct) | 0 |
| | Multiple Indicator Cluster Survey (MM adjusted) 1995 (Direct) | 1 |
| | Census 1998 (Indirect) | 1 |
| | Census 1998 (Household Deaths) | 0 |
| | Demographic and Health Survey 2000 (Direct) | 0 |
| | Demographic and Health Survey 2000 (Indirect) | 0 |
| | Demographic and Health Survey (MM adjusted) 2000 (Direct) | 1 |
| | Second Integrated Household Survey 2004-2005 (Household Deaths) | 0 |
| | Demographic and Health Survey 2004 (Direct) | 0 |
| | Demographic and Health Survey 2004 (Indirect) | 0 |
| | Demographic and Health Survey (MM adjusted) 2004 (Direct) | 1 |
| | Multiple Indicator Cluster Survey 2006 (Direct) | 0 |
| | Multiple Indicator Cluster Survey 2006 (Indirect) | 0 |
| | Multiple Indicator Cluster Survey (MM adjusted) 2006 (Direct) | 1 |
| | Census 2008 (Indirect) | 1 |
| | Census 2008 (Household Deaths) | 0 |
| | Demographic and Health Survey 2010 (Household Deaths) | 0 |
| | Demographic and Health Survey 2010 (Direct) | 0 |
| | Demographic and Health Survey (MM adjusted) 2010 (Direct) | 1 |
| | Third Integrated Household Survey 2010-2011 (Household Deaths) | 0 |
| | Malaria Indicator Survey 2012 (Indirect) | 0 |

| Country | Data series | Inclusion |
|---|---|---|
| | MDG Endline Survey 2013-2014 (Direct) | 0 |
| | MDG Endline Survey (MM adjusted) 2013-2014 (Direct) | 1 |
| | Malaria Indicator Survey 2014 (Indirect) | 1 |
| | Demographic and Health Survey 2015-2016 (Direct) | 0 |
| | Demographic and Health Survey (MM adjusted) 2015-2016 (Direct) | 1 |
| | Malaria Indicator Survey 2017 (Indirect) | 1 |
| | Census 2018 (Indirect) | 1 |
| | Multiple Indicator Cluster Survey 2019-2020 (Direct) | 0 |
| | Multiple Indicator Cluster Survey (MM adjusted) 2019-2020 (Direct) | 1 |
| | Demographic and Health Survey 2024 (Direct) | 0 |
| | Demographic and Health Survey (MM adjusted) 2024 (Direct) | 1 |
| Malaysia | National Life Table (Life Table) | 1 |
| | Census 1970 (Indirect) | 1 |
| | World Fertility Survey 1974 (Direct) | 1 |
| | Census 1980 (Indirect) | 1 |
| | Vital Registration Data from Department of Statistics 2025 (VR) | 1 |
| | WHO Vital Registration Data 2025 version 2025 (VR) | 0 |
| Namibia | Census 1991 (Indirect) | 1 |
| | Demographic and Health Survey 1992 (Direct) | 0 |
| | Demographic and Health Survey 1992 (Indirect) | 0 |
| | Demographic and Health Survey (MM adjusted) 1992 (Direct) | 1 |
| | Demographic and Health Survey 2000 (Direct) | 0 |
| | Demographic and Health Survey 2000 (Indirect) | 0 |
| | Demographic and Health Survey (MM adjusted) 2000 (Direct) | 1 |
| | Census 2001 (Household Deaths) | 1 |
| | Demographic and Health Survey 2006-2007 (Household Deaths) | 0 |
| | Demographic and Health Survey 2006-2007 (Direct) | 0 |
| | Demographic and Health Survey 2006-2007 (Indirect) | 0 |
| | Demographic and Health Survey (MM adjusted) 2006-2007 (Direct) | 1 |
| | Census 2011 (Household Deaths) | 0 |
| | Census 2011 (Indirect) | 1 |
| | Demographic and Health Survey 2013 (Direct) | 0 |
| | Demographic and Health Survey (MM adjusted) 2013 (Direct) | 1 |

| Country | Data series | Inclusion |
| --- | --- | --- |
| | Inter-censal Demographic Survey 2016 (Household Deaths) | 1 |
| | Inter-censal Demographic Survey 2016 (Indirect) | 0 |
| | National Population Registration System 2017 (VR) | 0 |
| | Census 2023 (Indirect) | 0 |
| | Census 2023 (Household Deaths) | 1 |
| Niger | Survey 1960 (Indirect) | 0 |
| | Census 1988 (Indirect) | 0 |
| | Demographic and Health Survey 1992 (Direct) | 1 |
| | Demographic and Health Survey 1992 (Indirect) | 0 |
| | Multiple Indicator Cluster Survey 1996 (Direct) | 1 |
| | Multiple Indicator Cluster Survey 1996 (Indirect) | 0 |
| | Demographic and Health Survey 1998 (Direct) | 1 |
| | Demographic and Health Survey 1998 (Indirect) | 0 |
| | Multiple Indicator Cluster Survey 2000 (Indirect) | 1 |
| | Census 2001 (Household Deaths) | 0 |
| | Census 2001 (Indirect) | 0 |
| | Demographic and Health Survey 2006 (Direct) | 1 |
| | Demographic and Health Survey 2006 (Indirect) | 0 |
| | Child Survival and Mortality Survey New 2010 (Direct) | 1 |
| | Demographic and Health Survey 2012 (Direct) | 1 |
| | Etude Nationale d'Evaluation des Indicateurs Socioeconomiques et Demographiques 2015 (Direct) | 0 |
| | Malaria Indicator Survey 2021 (Indirect) | 1 |
| | Enquete Nationale sur la Fecondite et la Mortalite des Enfants de moins de 5 ans (ENAFEME) 2021 (Direct) | 1 |
| Nigeria | Rural Demographic Sample Survey 1965-1966 (Household Deaths) | 1 |
| | Survey 1971-1973 (Indirect) | 0 |
| | Malumfashi DSS 1962-1977 (Household Deaths) | 0 |
| | World Fertility Survey 1981-1982 (Direct) | 0 |
| | World Fertility Survey 1981-1982 (Indirect) | 0 |
| | Demographic and Health Survey 1986 (Direct) | 0 |
| | Demographic and Health Survey 1990 (Direct) | 1 |
| | Demographic and Health Survey 1990 (Indirect) | 0 |
| | Multiple Indicator Cluster Survey 1995 (Indirect) | 0 |
| | Multiple Indicator Cluster Survey 1999 (Indirect) | 0 |

| Country | Data series | Inclusion |
|---|---|---|
| | Demographic and Health Survey 1999 (Direct) | 0 |
| | Demographic and Health Survey 1999 (Indirect) | 0 |
| | Demographic and Health Survey 2003 (Direct) | 1 |
| | Demographic and Health Survey 2003 (Indirect) | 0 |
| | Multiple Indicator Cluster Survey 2007 (Indirect) | 0 |
| | Demographic and Health Survey 2008 (Household Deaths) | 0 |
| | Demographic and Health Survey 2008 (Direct) | 1 |
| | Demographic and Health Survey 2008 (Indirect) | 0 |
| | Malaria Indicator Survey 2010 (Direct) | 1 |
| | GHS Panel Survey 2010 (Household Deaths) | 0 |
| | Multiple Indicator Cluster Survey 2011 (Indirect) | 1 |
| | GHS Panel Survey 2012-2013 (Household Deaths) | 0 |
| | Demographic and Health Survey 2013 (Household Deaths) | 0 |
| | Demographic and Health Survey 2013 (Direct) | 1 |
| | Malaria Indicator Survey 2015 (Indirect) | 0 |
| | Multiple Indicator Cluster Survey 2016-2017 (Direct) | 1 |
| | Demographic and Health Survey 2018 (Direct) | 1 |
| | Malaria Indicator Survey 2021 (Indirect) | 0 |
| | Multiple Indicator Cluster Survey 2021 (Direct) | 1 |
| | Demographic and Health Survey 2024 (Direct) | 1 |
| Nicaragua | Census 1971 (Indirect) | 1 |
| | Enc.Demografica Nacional 1978 (Indirect) | 1 |
| | Enc.Socio-Demografica Nicaraguense 1985 (Indirect) | 1 |
| | Family Health Survey 1992-1993 (Direct) | 1 |
| | Family Health Survey 1992-1993 (Indirect) | 0 |
| | Census 1995 (Household Deaths) | 0 |
| | Census 1995 (Indirect) | 1 |
| | Demographic and Health Survey 1998 (Direct) | 1 |
| | Demographic and Health Survey 1998 (Indirect) | 0 |
| | Living Standards Measurement Survey 2001 (Household Deaths) | 0 |
| | Demographic and Health Survey 2001 (Direct) | 1 |
| | Demographic and Health Survey 2001 (Indirect) | 0 |
| | Census 2005 (Household Deaths) | 0 |

| Country | Data series | Inclusion |
| --- | --- | --- |
| | Census 2005 (Indirect) | 1 |
| | Nicaraguense de Demografia y Salud (ENDESA) 2006-2007 (Direct) | 1 |
| | Encuesta Nicaraguense de Demografia y Salud 2011-2012 (Direct) | 1 |
| | UNPD Vital Registration Data 2022 version 2022 (VR) | 0 |
| | VR from Ministra de Salud 2022 (VR) | 0 |
| | VR from Ministra de Salud (live births) 2025 (VR) | 1 |
| | VR submitted by government to UN IGME 2025 (VR) | 0 |
| | VR submitted by government to UN IGME (live births) 2025 (VR) | 1 |
| Niue | Census 1961 (Indirect) | 1 |
| | Census 1986 (Indirect) | 1 |
| | Census 1991 (Indirect) | 1 |
| | Census 1997 (Indirect) | 1 |
| | Census 2001 (Indirect) | 1 |
| | Vital Statistics Report 2012-2016 (VR) | 0 |
| | Recalculated WHO Vital Registration Data 2025 version 2025 (VR) | 1 |
| | WHO Vital Registration Data 2025 version 2025 (VR) | 0 |
| Netherlands (Kingdom of the) | WHO Vital Registration Data 2025 version 2025 (VR) | 1 |
| Norway | WHO Vital Registration Data 2025 version 2025 (VR) | 1 |
| Nepal | Census 1971 (Indirect) | 0 |
| | World Fertility Survey 1976 (Direct) | 1 |
| | World Fertility Survey 1976 (Indirect) | 0 |
| | Census 1981 (Indirect) | 0 |
| | Contraceptive Prevalence Survey 1981 (Indirect) | 0 |
| | Fertility and Family Planning Survey 1986 (Indirect) | 0 |
| | Census 1991 (Indirect) | 0 |
| | Fertility and Family Planning Survey 1991 (Direct) | 1 |
| | Fertility and Family Planning Survey 1991 (Indirect) | 0 |
| | Demographic and Health Survey 1996 (Direct) | 1 |
| | Demographic and Health Survey 1996 (Indirect) | 0 |
| | Census 2001 (Household Deaths) | 0 |
| | Census 2001 (Indirect) | 0 |
| | Demographic and Health Survey 2001 (Direct) | 1 |
| | Demographic and Health Survey 2001 (Indirect) | 0 |

| Country | Data series | Inclusion |
| --- | --- | --- |
| | Demographic and Health Survey 2006 (Direct) | 1 |
| | Demographic and Health Survey 2006 (Indirect) | 0 |
| | Demographic and Health Survey 2011 (Direct) | 1 |
| | Census 2011 (Household Deaths) | 0 |
| | Multiple Indicator Cluster Survey 2014 (Direct) | 1 |
| | Demographic and Health Survey 2016 (Direct) | 1 |
| | Multiple Indicator Cluster Survey 2019 (Direct) | 1 |
| | Demographic and Health Survey 2022 (Direct) | 1 |
| Nauru | Vital Registration Data and Census 2000 (VR) | 1 |
| | Census 2002 (Indirect) | 1 |
| | Demographic and Health Survey 2007 (Direct) | 1 |
| | Census 2011 (Indirect) | 1 |
| | Vital Statistics Report 2008-2013 (VR) | 0 |
| | Vital Statistics Report 2015-2017 (VR) | 0 |
| | Census 2021 (Indirect) | 1 |
| | Multiple Indicator Cluster Survey 2023 (Direct) | 1 |
| | WHO Vital Registration Data 2025 version 2025 (VR) | 0 |
| New Zealand | Census 2006 (Household Deaths) | 0 |
| | HMD Vital Registration Data 2023 (VR) | 1 |
| | WHO Vital Registration Data 2025 version 2025 (VR) | 1 |
| Oman | Socio-Demographic Survey in 5 towns 1975 (Indirect) | 1 |
| | Socio-Demographic Survey in 11 towns 1977-1979 (Indirect) | 1 |
| | Child Health Survey 1988 (Direct) | 1 |
| | Child Health Survey 1988 (Indirect) | 0 |
| | Census 1993 (Indirect) | 1 |
| | Family Health Survey 1995 (Direct) | 1 |
| | Family Health Survey 1995 (Indirect) | 0 |
| | Comprehensive Health Survey for Evaluation and Reproductive Health 2000 (Indirect) | 1 |
| | Census 2003 (Household Deaths) | 1 |
| | Census 2003 (Indirect) | 1 |
| | Multiple Indicator Cluster Survey 2014 (Direct) | 0 |
| | Data from Ministry of Health Annual Health Report 2024 (VR) | 1 |
| | WHO Vital Registration Data 2025 version 2025 (VR) | 0 |

| Country | Data series | Inclusion |
|---|---|---|
| Pakistan | Integrated Household Survey 1957-1958 (Indirect) | 1 |
| | Population Growth Estimation Experiment 1962-1965 (Household Deaths) | 0 |
| | Population Growth Estimation Experiment 1962-1965 (Direct) | 1 |
| | Population Growth Survey I 1971 (Direct) | 1 |
| | Housing, Economic, Demographic Characteristics Survey 1973 (Indirect) | 0 |
| | World Fertility Survey 1975 (Direct) | 1 |
| | Population Growth Survey II 1976-1978 (Direct) | 1 |
| | Labour Force and Migration Survey 1980 (Direct) | 1 |
| | Census 1981 (Indirect) | 1 |
| | Contraceptive Prevalence Survey 1984 (Indirect) | 1 |
| | Demographic Survey 1984 (Indirect) | 0 |
| | Demographic Survey 1988 (Direct) | 1 |
| | Demographic Survey 1988 (Indirect) | 0 |
| | Living Standards Survey 1991 (Direct) | 1 |
| | Living Standards Survey 1991 (Indirect) | 0 |
| | Demographic and Health Survey 1990-1991 (Direct) | 1 |
| | Demographic and Health Survey 1990-1991 (Indirect) | 0 |
| | Contraceptive Prevalence Survey 1993 (Indirect) | 0 |
| | Contraceptive Prevalence Survey 1994-1995 (Indirect) | 1 |
| | Pakistan Fertility and Family Planning Survey 1996 (Direct) | 1 |
| | Pakistan Fertility and Family Planning Survey 1996 (Indirect) | 0 |
| | Integrated Household Survey 1998 (Direct) | 1 |
| | Integrated Household Survey 1998 (Indirect) | 0 |
| | Census 1998 (Indirect) | 1 |
| | Pakistan Demographic Survey 1984-1999 (VR) | 1 |
| | Integrated Household Survey 2001 (Direct) | 1 |
| | Integrated Household Survey 2001 (Indirect) | 0 |
| | Reproductive Health and Family Planning Survey 2000-2001 (Direct) | 1 |
| | Reproductive Health and Family Planning Survey 2000-2001 (Indirect) | 0 |
| | Demographic Survey 2000-2007 (Household Deaths) | 1 |
| | Demographic and Health Survey 2006-2007 (Direct) | 1 |
| | Demographic and Health Survey 2006-2007 (Indirect) | 0 |
| | Demographic and Health Survey 2012-2013 (Direct) | 1 |

| Country | Data series | Inclusion |
|---|---|---|
| | Social & Living Standards Measurement Survey 2013-2014 (Direct) | 1 |
| | Demographic and Health Survey 2017-2018 (Direct) | 1 |
| | Social & Living Standards Measurement Survey 2018-2019 (Direct) | 1 |
| | Pakistan Demographic Survey 2020 (Household Deaths) | 1 |
| | Household Integrated Economic Survey 2024-2025 (Direct) | 0 |
| Panama | TABLAS DE VIDA NACIONALES (Life Table) | 1 |
| | Vital Registration and Census (VR) | 0 |
| | Vital Registration from National Statistical Institute (VR) | 0 |
| | World Fertility Survey 1975-1976 (Direct) | 1 |
| | Encuesta Demografica Nacional 1975-1977 (Direct) | 0 |
| | Encuesta Demografica Nacional 1975-1977 (Indirect) | 0 |
| | Census 1980 (Indirect) | 1 |
| | Census 1990 (Indirect) | 1 |
| | Census 2000 (Household Deaths) | 0 |
| | Census 2000 (Indirect) | 1 |
| | Census 2010 (Indirect) | 1 |
| | UNPD Vital Registration Data 2022 version 2022 (VR) | 0 |
| | Census 2023 (Indirect) | 0 |
| | Census 2023 (Household Deaths) | 0 |
| | WHO Vital Registration Data 2025 version 2025 (VR) | 1 |
| Peru | Census 1972 (Indirect) | 1 |
| | Demographic Survey 1974-1976 (Direct) | 1 |
| | Demographic Survey 1974-1976 (Indirect) | 0 |
| | World Fertility Survey 1977-1978 (Direct) | 1 |
| | World Fertility Survey 1977-1978 (Indirect) | 0 |
| | Census 1981 (Indirect) | 1 |
| | Contraceptive Prevalence Survey 1981 (Indirect) | 1 |
| | Demographic and Health Survey 1986 (Direct) | 1 |
| | Demographic and Health Survey 1986 (Indirect) | 0 |
| | Demographic and Health Survey 1991-1992 (Direct) | 1 |
| | Demographic and Health Survey 1991-1992 (Indirect) | 0 |
| | Census 1993 (Indirect) | 1 |
| | Demographic and Health Survey 1996 (Direct) | 1 |

| Country | Data series | Inclusion |
| --- | --- | --- |
| | Demographic and Health Survey 1996 (Indirect) | 0 |
| | Demographic and Health Survey 2000 (Direct) | 1 |
| | Demographic and Health Survey 2000 (Indirect) | 0 |
| | Census 2007 (Indirect) | 1 |
| | Demographic and Health Survey 2004-2008 (Direct) | 1 |
| | Demographic and Health Survey 2004-2008 (Indirect) | 0 |
| | Vital Registration (26 March 2009) 2009 (VR) | 0 |
| | Encuesta Demografica y de Salud Familiar (ENDES) 2009 (Direct) | 1 |
| | Encuesta Demografica y de Salud Familiar (ENDES) 2010 (Direct) | 1 |
| | Encuesta Demografica y de Salud Familiar (ENDES) 2011 (Direct) | 1 |
| | Demographic and Health Survey 2012 (Direct) | 1 |
| | Demographic and Health Survey 2013 (Direct) | 1 |
| | Encuesta Demografica y de Salud Familiar 2014-2015 (Direct) | 1 |
| | Census 2017 (Indirect) | 1 |
| | Encuesta Demografica y de Salud Familiar 2016-2017 (Direct) | 1 |
| | Encuesta Demografica y de Salud Familiar (ENDES) 2018 (Household Deaths) | 0 |
| | Encuesta Demografica y de Salud Familiar 2019 (Household Deaths) | 0 |
| | Encuesta Demografica y de Salud Familiar 2018-2019 (Direct) | 1 |
| | Vital Registration from RENIEC using live births 2021 (VR) | 0 |
| | Vital Registration from RENIEC using population 2021 (VR) | 0 |
| | Encuesta Demografica y de Salud Familiar 2020 (Household Deaths) | 0 |
| | Encuesta Demografica y de Salud Familiar 2020-2021 (Direct) | 1 |
| | Encuesta Demografica y de Salud Familiar 2022-2023 (Direct) | 1 |
| | WHO Vital Registration Data 2025 version 2025 (VR) | 0 |
| Philippines | Census 1970 (Indirect) | 0 |
| | World Fertility Survey 1978 (Direct) | 1 |
| | Census 1980 (Indirect) | 0 |
| | Demographic Survey 1988 (Indirect) | 1 |
| | Census 1990 (Indirect) | 0 |
| | Demographic and Health Survey 1993 (Direct) | 1 |
| | Demographic and Health Survey 1993 (Indirect) | 0 |
| | Demographic and Health Survey 1998 (Direct) | 1 |
| | Demographic and Health Survey 1998 (Indirect) | 0 |

| Country | Data series | Inclusion |
| --- | --- | --- |
| | Demographic and Health Survey 2003 (Direct) | 1 |
| | Demographic and Health Survey 2003 (Indirect) | 0 |
| | Family Planning Survey 2006 (Direct) | 0 |
| | Family Planning Survey 2006 (Indirect) | 1 |
| | Demographic and Health Survey 2008 (Direct) | 1 |
| | Demographic and Health Survey 2008 (Indirect) | 0 |
| | Census 2010 (Indirect) | 0 |
| | Family Health Survey 2011 (Direct) | 1 |
| | National Demographic and Health Survey 2013 (Direct) | 1 |
| | Demographic and Health Survey 2017 (Direct) | 1 |
| | Census 2020 (Indirect) | 0 |
| | Demographic and Health Survey 2022 (Direct) | 1 |
| | WHO Vital Registration Data 2025 version 2025 (VR) | 0 |
| Palau | Census 1973 (Indirect) | 0 |
| | Census 1980 (Indirect) | 1 |
| | Census 1995 (Indirect) | 0 |
| | Census 2000 (Indirect) | 0 |
| | Census 2005 (Indirect) | 0 |
| | Census 2015 (Indirect) | 0 |
| | Census 2020 (Indirect) | 1 |
| | Recalculated WHO Vital Registration Data 2025 version 2025 (VR) | 1 |
| | WHO Vital Registration Data 2025 version 2025 (VR) | 0 |
| Papua New Guinea | Census 1966 (Indirect) | 1 |
| | Census 1971 (Indirect) | 1 |
| | Census 1980 (Indirect) | 0 |
| | Demographic and Health Survey 1991 (Indirect) | 1 |
| | Demographic and Health Survey 1996 (Direct) | 1 |
| | Demographic and Health Survey 1996 (Indirect) | 0 |
| | Census 2000 (Indirect) | 1 |
| | Demographic and Health Survey 2006 (Direct) | 1 |
| | Census 2011 (Indirect) | 0 |
| | Demographic and Health Survey 2016-2018 (Direct) | 1 |
| | WHO Vital Registration Data 2025 version 2025 (VR) | 0 |

| Country | Data series | Inclusion |
|---|---|---|
| Poland | Census 2011 (Household Deaths) | 0 |
| | UNPD Vital Registration Data 2022 version 2022 (VR) | 1 |
| | WHO Vital Registration Data 2025 version 2025 (VR) | 1 |
| Democratic People's Republic of Korea | Census (adjusted from 1q0 and 5q0 based on 4q0 and south model life table) 1993 (Household Deaths) | 1 |
| | Civil Registration Data from Central Bureau of Statistics 2000 (VR) | 1 |
| | Census (adjusted 1q0 and thus 5q0 based on 4q1 and north model life table) 2008 (Household Deaths) | 1 |
| | Socio-economic, Demographic and Health Survey 2014 (Life Table) | 0 |
| | Multiple Indicator Cluster Survey 2017 (Direct) | 0 |
| Portugal | World Fertility Survey 1979-1980 (Direct) | 0 |
| | Census 2011 (Household Deaths) | 0 |
| | UNPD Vital Registration Data 2022 version 2022 (VR) | 1 |
| | WHO Vital Registration Data 2025 version 2025 (VR) | 1 |
| Paraguay | Census 1972 (Indirect) | 1 |
| | National Survey of Contraceptive Use 1977 (Indirect) | 1 |
| | World Fertility Survey 1979 (Direct) | 1 |
| | World Fertility Survey 1979 (Indirect) | 0 |
| | Census 1982 (Indirect) | 1 |
| | Demographic and Health Survey 1990 (Direct) | 1 |
| | Demographic and Health Survey 1990 (Indirect) | 0 |
| | Census 1992 (Indirect) | 1 |
| | National Survey of Demography and Reproductive Health 1995-1996 (Direct) | 1 |
| | National Survey of Demography and Reproductive Health 1995-1996 (Indirect) | 0 |
| | Census 2002 (Household Deaths) | 0 |
| | Census 2002 (Indirect) | 1 |
| | Reproductive Health Survey 2004 (Direct) | 1 |
| | Encuesta Nacional de Demografia y Salud Sexual y Reproductiva 2008 (Direct) | 1 |
| | Multiple Indicator Cluster Survey 2016 (Direct) | 0 |
| | VR Ministerio de Salud Pulbica y Bienestar Social 2018 (VR) | 0 |
| | WHO Vital Registration Data 2025 version 2025 (VR) | 0 |
| State of Palestine | FALCOT-survey 1992 (Indirect) | 1 |
| | Demographic Survey 1995 (Direct) | 1 |
| | Demographic Survey 1995 (Indirect) | 0 |
| | Census 1997 (Indirect) | 1 |

| Country | Data series | Inclusion |
|---|---|---|
| | Health Survey 2000 (Direct) | 1 |
| | Health Survey 2000 (Indirect) | 0 |
| | Demographic and Health Survey 2004 (Direct) | 1 |
| | Demographic and Health Survey 2004 (Indirect) | 0 |
| | PAPFAM Family Health Survey 2006 (Direct) | 1 |
| | PAPFAM Family Health Survey 2006 (Indirect) | 0 |
| | Census 2007 (Indirect) | 1 |
| | Multiple Indicator Cluster Survey-Family Health Survey 2010 (Direct) | 1 |
| | Multiple Indicator Cluster Survey 2014 (Direct) | 1 |
| | Census 2017 (Indirect) | 1 |
| | Multiple Indicator Cluster Survey 2019-2020 (Direct) | 1 |
| | WHO Vital Registration Data 2025 version 2025 (VR) | 0 |
| Qatar | Child Health Survey 1987 (Direct) | 1 |
| | Child Health Survey 1987 (Indirect) | 0 |
| | Gulf Family Health Survey 1998 (Direct) | 0 |
| | Gulf Family Health Survey 1998 (Indirect) | 0 |
| | Census 2004 (Indirect) | 0 |
| | Vital Statistics, Annual Bulletin 2015 (VR) | 0 |
| | WHO Vital Registration Data 2025 version 2025 (VR) | 1 |
| Kosovo (UNSCR 1244) | Demographic, Social and Reproductive Health Survey 2003 (Indirect) | 0 |
| | Demographic, Social and Reproductive Health Survey 2009 (Direct) | 0 |
| | Census 2011 (Household Deaths) | 1 |
| | Multiple Indicator Cluster Survey 2013-2014 (Direct) | 1 |
| | Vital Registration Data from Kosovo Agency of Statistics 2020 (VR) | 1 |
| | Multiple Indicator Cluster Survey 2019-2020 (Direct) | 1 |
| | Vital Registration Data from Kosovo Agency of Statistics 2023 (VR) | 0 |
| Romania | Reproductive Health Survey 1999 (Direct) | 0 |
| | UNPD Vital Registration Data 2022 version 2022 (VR) | 1 |
| | WHO Vital Registration Data 2025 version 2025 (VR) | 1 |
| Russian Federation | Trans MONEE Vital Registration  (VR) | 0 |
| | Census 1989 (Indirect) | 1 |
| | HMD Vital Registration Data 2025 (VR) | 1 |
| | WHO Vital Registration Data 2025 version 2025 (VR) | 1 |

| Country | Data series | Inclusion |
|---|---|---|
| Rwanda | Demographic Survey 1970 (Indirect) | 1 |
| | Census 1978 (Indirect) | 1 |
| | World Fertility Survey 1983 (Direct) | 1 |
| | World Fertility Survey 1983 (Indirect) | 0 |
| | Census 1991 (Indirect) | 1 |
| | Demographic and Health Survey 1992 (Direct) | 0 |
| | Demographic and Health Survey 1992 (Indirect) | 0 |
| | Demographic and Health Survey (MM adjusted) 1992 (Direct) | 1 |
| | Socio-demographic Survey 1996 (Indirect) | 1 |
| | Demographic and Health Survey 2000 (Direct) | 0 |
| | Demographic and Health Survey 2000 (Indirect) | 0 |
| | Demographic and Health Survey (MM adjusted) 2000 (Direct) | 1 |
| | Census 2002 (Indirect) | 1 |
| | Census 2002 (Household Deaths) | 0 |
| | Demographic and Health Survey 2005 (Direct) | 0 |
| | Demographic and Health Survey 2005 (Indirect) | 0 |
| | Demographic and Health Survey (MM adjusted) 2005 (Direct) | 1 |
| | Demographic and Health Survey Adjusted by adding 20 points 2007-2008 (Indirect) | 0 |
| | Interim Demographic and Health Survey 2007-2008 (Direct) | 0 |
| | Interim Demographic and Health Survey (MM adjusted) 2007-2008 (Direct) | 1 |
| | Demographic and Health Survey 2010 (Direct) | 0 |
| | Demographic and Health Survey 2010 (Indirect) | 0 |
| | Demographic and Health Survey (MM adjusted) 2010 (Direct) | 1 |
| | Census 2012 (Household Deaths) | 0 |
| | Census 2012 (Indirect) | 1 |
| | Malaria Indicator Survey 2013 (Indirect) | 1 |
| | Demographic and Health Survey 2014-2015 (Direct) | 0 |
| | Demographic and Health Survey (MM adjusted) 2014-2015 (Direct) | 1 |
| | Malaria Indicator Survey 2017 (Indirect) | 1 |
| | Demographic and Health Survey 2019-2020 (Direct) | 0 |
| | Demographic and Health Survey (MM adjusted) 2019-2020 (Direct) | 1 |
| | Census 2022 (Household Deaths) | 1 |
| Saudi Arabia | Child Health Survey 1987 (Indirect) | 1 |

| Country | Data series | Inclusion |
| --- | --- | --- |
| | Family Health Survey 1996 (Direct) | 0 |
| | Family Health Survey 1996 (Indirect) | 1 |
| | Demographic Survey 1999 (Household Deaths) | 1 |
| | Demographic Survey 2000 (Household Deaths) | 1 |
| | Census 2004 (Household Deaths) | 1 |
| | Demographic Survey 2007 (Household Deaths) | 1 |
| | Demographic Survey 2016 (Direct) | 1 |
| | Household Health Survey 2017 (Direct) | 1 |
| | Household Health Survey 2018 (Direct) | 1 |
| | Vital Registration Data from General Authority for Statistics Report 2011-2022 2023 (VR) | 0 |
| | Vital Registration Data from the Ministry of Health Statistical Yearbook 2024 (VR) | 0 |
| | WHO Vital Registration Data 2025 version 2025 (VR) | 0 |
| Sudan | Census 1955-1956 (Household Deaths) | 1 |
| | Census 1973 (Indirect) | 1 |
| | World Fertility Survey 1978-1979 (Direct) | 1 |
| | World Fertility Survey (Household Survey) 1978-1979 (Indirect) | 0 |
| | World Fertility Survey (Individual Survey) 1978-1979 (Indirect) | 0 |
| | Census 1983 (Indirect) | 1 |
| | Demographic and Health Survey 1989-1990 (Direct) | 1 |
| | Demographic and Health Survey 1989-1990 (Indirect) | 0 |
| | Maternal and Child Health Survey 1992 (Direct) | 1 |
| | Census 1993 (Household Deaths) | 1 |
| | Census 1993 (Indirect) | 1 |
| | Maternal and Child Health Survey 1992 (Indirect) | 1 |
| | Safe Motherhood Survey 1999 (Direct) | 1 |
| | Safe Motherhood Survey 1999 (Indirect) | 0 |
| | Sudan Household Health Survey 2006 (Household Deaths) | 1 |
| | Sudan Household Health Survey 2006 (Indirect) | 1 |
| | Census 2008 (Household Deaths) | 0 |
| | Census 2008 (Indirect) | 1 |
| | Multiple Indicator Cluster Survey 2010 (Direct) | 1 |
| | Multiple Indicator Cluster Survey 2014 (Direct) | 1 |
| Senegal | Survey 1960-1961 (Indirect) | 1 |

| Country | Data series | Inclusion |
|---|---|---|
| | Multiround Survey 1970-1971 (Household Deaths) | 1 |
| | World Fertility Survey 1978 (Direct) | 1 |
| | World Fertility Survey 1978 (Indirect) | 0 |
| | Multiround Survey 1978-1979 (Household Deaths) | 1 |
| | Demographic and Health Survey 1986 (Direct) | 1 |
| | Demographic and Health Survey 1986 (Indirect) | 0 |
| | Demographic and Health Survey 1992-1993 (Direct) | 1 |
| | Demographic and Health Survey 1992-1993 (Indirect) | 0 |
| | Multiple Indicator Cluster Survey 1996 (Indirect) | 0 |
| | Demographic and Health Survey 1997 (Direct) | 1 |
| | Demographic and Health Survey 1997 (Indirect) | 0 |
| | Demographic and Health Survey 1999-2000 (Direct) | 1 |
| | Demographic and Health Survey 1999-2000 (Indirect) | 0 |
| | Census 2002 (Indirect) | 0 |
| | Census 2002 (Household Deaths) | 0 |
| | Demographic and Health Survey 2005 (Direct) | 1 |
| | Demographic and Health Survey 2005 (Indirect) | 0 |
| | Malaria Indicator Survey 2006 (Indirect) | 1 |
| | Malaria Indicator Survey 2008-2009 (Direct) | 1 |
| | Malaria Indicator Survey 2008-2009 (Indirect) | 0 |
| | Demographic and Health Survey 2010-2011 (Direct) | 1 |
| | Census 2013 (Household Deaths) | 0 |
| | Census 2013 (Indirect) | 0 |
| | Demographic and Health Survey 2012-2013 (Direct) | 1 |
| | Demographic and Health Survey 2014 (Direct) | 1 |
| | Demographic and Health Survey 2015 (Direct) | 1 |
| | Demographic and Health Survey 2016 (Direct) | 1 |
| | Demographic and Health Survey 2017 (Direct) | 1 |
| | Demographic and Health Survey 2018 (Direct) | 1 |
| | Demographic and Health Survey 2019 (Direct) | 1 |
| | Malaria Indicator Survey 2020-2021 (Indirect) | 1 |
| | Demographic and Health Survey 2023 (Direct) | 1 |
| Singapore | Recalculated WHO Vital Registration Data 2025 version 2025 (VR) | 1 |

| Country | Data series | Inclusion |
| --- | --- | --- |
| | WHO Vital Registration Data 2025 version 2025 (VR) | 0 |
| Solomon Islands | Census 1959 (Indirect) | 1 |
| | Census 1970 (Indirect) | 1 |
| | Census 1976 (Indirect) | 1 |
| | Census 1986 (Indirect) | 1 |
| | Census 1999 (Indirect) | 1 |
| | Demographic and Health Survey 2007 (Direct) | 1 |
| | Demographic and Health Survey 2007 (Indirect) | 0 |
| | Census 2009 (Household Deaths) | 0 |
| | Census 2009 (Indirect) | 1 |
| | Demographic and Health Survey 2015 (Direct) | 1 |
| | Census 2019 (Indirect) | 1 |
| Sierra Leone | National Fertility Survey 1969 (Indirect) | 0 |
| | Pilot Census 1973 (Indirect) | 1 |
| | Census 1974 (Indirect) | 1 |
| | National Survey 1977 (Household Deaths) | 1 |
| | Census 1985 (Indirect) | 1 |
| | DSMS 1992 (Household Deaths) | 1 |
| | DSMS 1992 (Indirect) | 1 |
| | Multiple Indicator Cluster Survey 2000 (Indirect) | 1 |
| | Pilot Census 2003 (Indirect) | 0 |
| | Census 2004 (Household Deaths) | 1 |
| | Census 2004 (Indirect) | 1 |
| | Multiple Indicator Cluster Survey 2005-2006 (Indirect) | 1 |
| | Demographic and Health Survey 2008 (Direct) | 0 |
| | Demographic and Health Survey 2008 (Indirect) | 0 |
| | Multiple Indicator Cluster Survey 2010 (Indirect) | 1 |
| | VR from Health Management Information System 2012 (VR) | 0 |
| | Demographic and Health Survey 2013 (Direct) | 1 |
| | Census 2015 (Household Deaths) | 1 |
| | Census 2015 (Indirect) | 1 |
| | Malaria Indicator Survey 2016 (Indirect) | 1 |
| | Multiple Indicator Cluster Survey 2017 (Direct) | 0 |

| Country | Data series | Inclusion |
| --- | --- | --- |
| | Demographic and Health Survey 2019 (Direct) | 1 |
| | Countrywide Mortality Surveillance for Action (COMSA) 2023 (VR) | 0 |
| El Salvador | Vital Registration Data from Informe de Labores Ministerio de Salud 2010-2011 (VR) | 0 |
| | Census 1971 (Indirect) | 1 |
| | National Fertility Survey 1973 (Indirect) | 0 |
| | National Family Health Survey 1985 (Direct) | 0 |
| | National Family Health Survey 1985 (Indirect) | 1 |
| | National Family Health Survey 1988 (Direct) | 1 |
| | Census 1992 (Household Deaths) | 0 |
| | Census 1992 (Indirect) | 1 |
| | Household Survey of Multiple Purposes 1992 (Indirect) | 1 |
| | Household Survey of Multiple Purposes 1993 (Indirect) | 1 |
| | National Family Health Survey 1993 (Direct) | 1 |
| | National Family Health Survey 1993 (Indirect) | 0 |
| | National Family Health Survey 1998 (Direct) | 1 |
| | National Family Health Survey 1998 (Indirect) | 0 |
| | National Family Health Survey 2002-2003 (Direct) | 1 |
| | Census 2007 (Household Deaths) | 0 |
| | Census 2007 (Indirect) | 1 |
| | National Family Health Survey 2008 (Direct) | 1 |
| | Vital Registration Data from Informe de Labores Ministerio de Salud 2013-2014 2013-2014 (VR) | 0 |
| | Multiple Indicator Cluster Survey 2014 (Direct) | 1 |
| | Vital Registration Data from Informe de Labores Ministerio de Salud 2015-2016 2015-2016 (VR) | 0 |
| | Encuesta Nacional de Salud (ENS) 2021 (Direct) | 1 |
| | UNPD Vital Registration Data 2022 version 2022 (VR) | 0 |
| | WHO Vital Registration Data 2025 version 2025 (VR) | 0 |
| San Marino | Recalculated WHO Vital Registration Data 2025 version 2025 (VR) | 1 |
| | WHO Vital Registration Data 2025 version 2025 (VR) | 0 |
| Somalia | Multiple Indicator Cluster Survey 1999 (Indirect) | 1 |
| | Multiple Indicator Cluster Survey 2006 (Direct) | 1 |
| | Multiple Indicator Cluster Survey 2006 (Indirect) | 0 |
| | Health and Demographic Survey 2018-2019 (Indirect) | 0 |
| | Census 1955-1956 (Household Deaths) | 1 |

| Country | Data series | Inclusion |
|---|---|---|
| | Census 1973 (Indirect) | 1 |
| | SDN_1979WFS_HH 1978-1979 (Indirect) | 0 |
| | Census 1983 (Indirect) | 1 |
| | Demographic and Health Survey 1989-1990 (Direct) | 1 |
| | Demographic and Health Survey 1989-1990 (Indirect) | 0 |
| | Census 1993 (Indirect) | 1 |
| | Pan Arab for Child Development Survey 1993 (Direct) | 1 |
| | Multiple Indicator Cluster Survey 2006 (Direct) | 1 |
| | Multiple Indicator Cluster Survey 2006 (Indirect) | 1 |
| | Census 2008 (Indirect) | 1 |
| Serbia | Vital Registration Data from Statistical Office of the Republic of Serbia and Institute of Public Health of Serbia (VR) | 0 |
| | Multiple Indicator Cluster Survey 2010 (Indirect) | 0 |
| | Multiple Indicator Cluster Survey 2019 (Indirect) | 0 |
| | UNPD Vital Registration Data 2022 version 2022 (VR) | 0 |
| | WHO Vital Registration Data 2025 version 2025 (VR) | 1 |
| South Sudan | Census 1955-1956 (Household Deaths) | 1 |
| | Census 1973 (Indirect) | 0 |
| | Census 1983 (Indirect) | 0 |
| | UNICEF Survey 1985 (Indirect) | 0 |
| | Census 1993 (Household Deaths) | 1 |
| | Census 1993 (Indirect) | 1 |
| | Demography of Forced Migration Project DFMP survey 1999 (Indirect) | 0 |
| | Sudan Household Health Survey 2006 (Household Deaths) | 1 |
| | Sudan Household Health Survey 2006 (Indirect) | 0 |
| | Census 2008 (Household Deaths) | 0 |
| | Census 2008 (Indirect) | 1 |
| | Household Health Survey 2010 (Direct) | 0 |
| Sao Tome and Principe | Census 1980 (Indirect) | 1 |
| | Census 1991 (Indirect) | 1 |
| | Multiple Indicator Cluster Survey 2000 (Indirect) | 1 |
| | Multiple Indicator Cluster Survey 2006 (Indirect) | 0 |
| | Demographic and Health Survey 2008-2009 (Direct) | 1 |
| | Demographic and Health Survey 2008-2009 (Indirect) | 0 |

| Country | Data series | Inclusion |
|---|---|---|
| | Census 2012 (Household Deaths) | 0 |
| | Multiple Indicator Cluster Survey 2014 (Direct) | 1 |
| | Multiple Indicator Cluster Survey 2019 (Direct) | 1 |
| | WHO Vital Registration Data 2025 version 2025 (VR) | 0 |
| Suriname | Multiple Indicator Cluster Survey 1999-2000 (Indirect) | 1 |
| | Census 2004 (Indirect) | 0 |
| | Multiple Indicator Cluster Survey 2006 (Indirect) | 1 |
| | Census 2012 (Indirect) | 0 |
| | Multiple Indicator Cluster Survey 2018 (Direct) | 1 |
| | WHO Vital Registration Data 2025 version 2025 (VR) | 0 |
| Slovakia | HMD Vital Registration Data 2025 (VR) | 1 |
| | WHO Vital Registration Data 2025 version 2025 (VR) | 1 |
| Slovenia | Recalculated WHO Vital Registration Data 2025 version 2025 (VR) | 1 |
| | WHO Vital Registration Data 2025 version 2025 (VR) | 0 |
| Sweden | WHO Vital Registration Data 2025 version 2025 (VR) | 1 |
| Eswatini | Census 1966 (Indirect) | 1 |
| | Census 1976 (Indirect) | 1 |
| | Census 1986 (Indirect) | 1 |
| | Census 1997 (Indirect) | 1 |
| | Census 1997 (Household Deaths) | 0 |
| | Multiple Indicator Cluster Survey 1999-2000 (Indirect) | 1 |
| | Census 2007 (Indirect) | 1 |
| | Demographic and Health Survey 2006-2007 (Household Deaths) | 0 |
| | Demographic and Health Survey 2006-2007 (Direct) | 0 |
| | Demographic and Health Survey 2006-2007 (Indirect) | 0 |
| | Demographic and Health Survey (MM adjusted) 2006-2007 (Direct) | 1 |
| | Census 2007 (Household Deaths) | 0 |
| | Multiple Indicator Cluster Survey 2010 (Direct) | 0 |
| | Multiple Indicator Cluster Survey (MM adjusted) 2010 (Direct) | 1 |
| | Multiple Indicator Cluster Survey 2014 (Direct) | 0 |
| | Multiple Indicator Cluster Survey (MM adjusted) 2014 (Direct) | 1 |
| | Multiple Indicator Cluster Survey 2021-2022 (Direct) | 0 |
| | Multiple Indicator Cluster Survey (MM adjusted) 2021-2022 (Direct) | 1 |

| Country | Data series | Inclusion |
|---|---|---|
| Seychelles | Census 1960 (Household Deaths) | 0 |
| | Census 1960 (Indirect) | 1 |
| | Census 1971 (Indirect) | 1 |
| | Recalculated WHO Vital Registration Data 2025 version 2025 (VR) | 1 |
| | WHO Vital Registration Data 2025 version 2025 (VR) | 0 |
| Syrian Arab Republic | Census 1970 (Indirect) | 1 |
| | Census 1976 (Indirect) | 1 |
| | World Fertility Survey 1978 (Direct) | 1 |
| | World Fertility Survey 1978 (Indirect) | 0 |
| | Census 1981 (Indirect) | 1 |
| | EPI/CDD and Child Mortality Survey 1990 (Indirect) | 1 |
| | PAPCHILD Maternal and Child Health Survey 1993 (Direct) | 1 |
| | Census 1994 (Indirect) | 1 |
| | PAPFAM Family Health Survey 2001 (Direct) | 1 |
| | Population and Housing Census 2004 (Household Deaths) | 1 |
| | Multiple Indicator Cluster Survey 2006 (Indirect) | 1 |
| | Health survey for causes of child deaths 2007-2008 (Household Deaths) | 1 |
| | PAPFAM Family Health Survey 2009 (Direct) | 1 |
| | Vital Registration/hospitals/funeral offices Data 2009 (VR) | 0 |
| | Health survey for causes of child deaths 2017-2018 (Household Deaths) | 1 |
| | WHO Vital Registration Data 2025 version 2025 (VR) | 0 |
| Turks and Caicos Islands | Census 2001 (Household Deaths) | 1 |
| | Multiple Indicator Cluster Survey 2019-2020 (Direct) | 1 |
| | Recalculated UNPD Vital Registration Data 2022 version 2022 (VR) | 1 |
| | UNPD Vital Registration Data 2022 version 2022 (VR) | 0 |
| Chad | Population Survey 1963 (Indirect) | 1 |
| | Census 1993 (Indirect) | 0 |
| | Demographic and Health Survey 1996-1997 (Direct) | 1 |
| | Demographic and Health Survey 1996-1997 (Indirect) | 0 |
| | Multiple Indicator Cluster Survey 2000 (Indirect) | 1 |
| | Demographic and Health Survey 2004 (Direct) | 1 |
| | Demographic and Health Survey 2004 (Indirect) | 0 |
| | Census 2009 (Household Deaths) | 0 |

| Country | Data series | Inclusion |
|---|---|---|
| | Census 2009 (Indirect) | 0 |
| | Multiple Indicator Cluster Survey 2010 (Indirect) | 1 |
| | Demographic and Health Survey 2014-2015 (Direct) | 1 |
| | Multiple Indicator Cluster Survey 2019 (Direct) | 0 |
| Togo | Census 1958 (Indirect) | 1 |
| | Demographic survey 1961 (Household Deaths) | 0 |
| | Demographic survey 1961 (Indirect) | 1 |
| | Demographic Survey 1971 (Indirect) | 1 |
| | Census 1981 (Household Deaths) | 0 |
| | Demographic and Health Survey 1988 (Direct) | 1 |
| | Demographic and Health Survey 1988 (Indirect) | 0 |
| | Multiple Indicator Cluster Survey 1996 (Direct) | 1 |
| | Demographic and Health Survey 1998 (Direct) | 1 |
| | Demographic and Health Survey 1998 (Indirect) | 0 |
| | Multiple Indicator Cluster Survey 2006 (Indirect) | 1 |
| | Census 2010 (Indirect) | 1 |
| | Multiple Indicator Cluster Survey 2010 (Indirect) | 1 |
| | Census 2010 (Household Deaths) | 0 |
| | Demographic and Health Survey 2013-2014 (Direct) | 1 |
| | Malaria Indicator Survey 2017 (Indirect) | 1 |
| | Multiple Indicator Cluster Survey 2017 (Direct) | 1 |
| Thailand | Life Table by Hill 1980-1990 Intercensal Death (Life Table) | 1 |
| | Life Table by Hill 1990-2000 Intercensal Death (Life Table) | 1 |
| | Life Table in 2002 by Hill (Life Table) | 1 |
| | National Life Tables (Life Table) | 1 |
| | Vital Registration (VR) | 0 |
| | Census 1970 (Indirect) | 1 |
| | Survey of Population Change 1974-1975 (Direct) | 1 |
| | Survey of Population Change 1974-1975 (Indirect) | 0 |
| | World Fertility Survey 1975 (Direct) | 1 |
| | World Fertility Survey 1975 (Indirect) | 0 |
| | National Fertility Survey 1979 (Indirect) | 1 |
| | Census 1980 (Indirect) | 1 |

| Country | Data series | Inclusion |
|---|---|---|
| | Contraceptive Prevalence Survey 1981 (Indirect) | 1 |
| | Contraceptive Prevalence Survey 1984 (Indirect) | 1 |
| | Survey of Population Change 1985-1986 (Direct) | 1 |
| | Survey of Population Change 1985-1986 (Indirect) | 0 |
| | Demographic and Health Survey 1987 (Direct) | 1 |
| | Demographic and Health Survey 1987 (Indirect) | 0 |
| | Survey of Population Change 1989 (Direct) | 1 |
| | Survey of Population Change 1989 (Indirect) | 0 |
| | Census 1990 (Indirect) | 0 |
| | Contraceptive Prevalence Survey 1996 (Indirect) | 1 |
| | Survey of Population Change 1995-1996 (Direct) | 1 |
| | Survey of Population Change 1995-1996 (Indirect) | 0 |
| | Census 2000 (Indirect) | 1 |
| | Multiple Indicator Cluster Survey 2005-2006 (Indirect) | 1 |
| | Survey of Population Change 2005-2006 (Direct) | 1 |
| | Survey of Population Change 2005-2006 (Indirect) | 0 |
| | Census 2010 (Indirect) | 0 |
| | Multiple Indicator Cluster Survey 2012-2013 (Indirect) | 1 |
| | Vital Registration from Ministry of Public Health 2015 (VR) | 0 |
| | Multiple Indicator Cluster Survey 2015-2016 (Indirect) | 0 |
| | Survey of Population Change 2015-2016 (Indirect) | 0 |
| | Multiple Indicator Cluster Survey 2019 (Indirect) | 0 |
| | Multiple Indicator Cluster Survey 2022 (Indirect) | 0 |
| | WHO Vital Registration Data 2025 version 2025 (VR) | 1 |
| Tajikistan | Census 1989 (Indirect) | 1 |
| | Tajikistan Living Standards Survey 1999 (Direct) | 1 |
| | Tajikistan Living Standards Survey 1999 (Indirect) | 0 |
| | Multiple Indicator Cluster Survey 2000 (Indirect) | 1 |
| | Demographic Survey 2002 (Direct) | 1 |
| | Demographic Survey 2002 (Indirect) | 0 |
| | Tajikistan Living Standards Survey 2003 (Direct) | 0 |
| | Tajikistan Living Standards Survey 2003 (Indirect) | 0 |
| | Multiple Indicator Cluster Survey 2005 (Indirect) | 1 |

| Country | Data series | Inclusion |
|---|---|---|
| | Tajikistan Living Standards Survey 2007 (Direct) | 0 |
| | Tajikistan Living Standards Survey 2007 (Indirect) | 0 |
| | Census 2010 (Indirect) | 0 |
| | Survey on Infant, Child and Maternal Mortality 2010 (Direct) | 0 |
| | Survey on Infant, Child and Maternal Mortality 2010 (Indirect) | 0 |
| | Vital Registration Data from Statistical Yearbook Health Status and Performance of Health Facilities 2010 2010 (VR) | 0 |
| | Vital Registration Data from State Agency for Statistics 2012 2012 (VR) | 0 |
| | Demographic and Health Survey 2012 (Direct) | 1 |
| | Demographic and Health Survey 2017 (Direct) | 1 |
| | Demographic and Health Survey 2023 (Direct) | 1 |
| | WHO Vital Registration Data 2025 version 2025 (VR) | 1 |
| Turkmenistan | Vital Registration  (VR) | 0 |
| | Demographic and Health Survey 2000 (Direct) | 1 |
| | Demographic and Health Survey 2000 (Indirect) | 0 |
| | Multiple Indicator Cluster Survey 2006 (Indirect) | 1 |
| | Transmonee Vital Registration Data 2008 version 2008 (VR) | 0 |
| | Multiple Indicator Cluster Survey 2015-2016 (Direct) | 0 |
| | Multiple Indicator Cluster Survey 2019 (Direct) | 1 |
| | Vital Registration Data (Live Births) 2020 (VR) | 0 |
| | Multiple Indicator Cluster Survey 2025 (Direct) | 0 |
| | WHO Vital Registration Data 2025 version 2025 (VR) | 1 |
| Timor-Leste | Census 1990 (Indirect) | 0 |
| | ICS 1995 (Indirect) | 0 |
| | Living Standards Survey 2001 (Indirect) | 1 |
| | Multiple Indicator Cluster Survey 2002 (Indirect) | 1 |
| | Demographic and Health Survey 2003 (Direct) | 1 |
| | Census 2004 (Indirect) | 1 |
| | Census 2010 (Indirect) | 1 |
| | Demographic and Health Survey 2009-2010 (Direct) | 1 |
| | Census 2015 (Household Deaths) | 1 |
| | Demographic and Health Survey 2016 (Direct) | 0 |
| | Census 2022 (Indirect) | 1 |
| Tonga | Census 1976 (Indirect) | 1 |

| Country | Data series | Inclusion |
| --- | --- | --- |
| | Census 1986 (Indirect) | 1 |
| | Census 1996 (Indirect) | 1 |
| | Census 2006 (Household Deaths) | 0 |
| | Census 2006 (Indirect) | 1 |
| | National Demographic and Health Survey 2012 (Direct) | 1 |
| | Census 2016 (Indirect) | 1 |
| | Multiple Indicator Cluster Survey 2019 (Direct) | 1 |
| | Census 2021 (Indirect) | 1 |
| | WHO Vital Registration Data 2025 version 2025 (VR) | 0 |
| Trinidad and Tobago | National Life Tables (Life Table) | 0 |
| | World Fertility Survey 1977 (Direct) | 1 |
| | World Fertility Survey 1977 (Indirect) | 0 |
| | Demographic and Health Survey 1987 (Direct) | 1 |
| | Demographic and Health Survey 1987 (Indirect) | 0 |
| | Multiple Indicator Cluster Survey 2000 (Indirect) | 1 |
| | Multiple Indicator Cluster Survey 2006 (Indirect) | 1 |
| | Multiple Indicator Cluster Survey 2011 (Indirect) | 1 |
| | Population and Housing Census 2011 (Indirect) | 0 |
| | Preliminary MoH Vital Registration Data 2019 (VR) | 0 |
| | UNPD Vital Registration Data 2022 version 2022 (VR) | 0 |
| | Multiple Indicator Cluster Survey 2022 (Direct) | 1 |
| | WHO Vital Registration Data 2025 version 2025 (VR) | 0 |
| Tunisia | Vital Registration from Institut National de la Statistique (VR) | 1 |
| | National Demographic Survey 1968-1969 (Household Deaths) | 1 |
| | Census 1975 (Indirect) | 1 |
| | World Fertility Survey 1978 (Direct) | 0 |
| | World Fertility Survey 1978 (Indirect) | 0 |
| | Contraceptive Prevalence Survey 1983 (Indirect) | 1 |
| | Census 1984 (Indirect) | 1 |
| | Demographic and Health Survey 1988 (Direct) | 1 |
| | Demographic and Health Survey 1988 (Indirect) | 0 |
| | PAPCHILD Maternal and Child Health Survey 1994 (Direct) | 1 |
| | PAPFAM Family Health Survey 2001 (Direct) | 1 |

| Country | Data series | Inclusion |
|---|---|---|
| | Census 2004 (Indirect) | 1 |
| | Multiple Indicator Cluster Survey 2011-2012 (Direct) | 1 |
| | Census 2014 (Indirect) | 0 |
| | Multiple Indicator Cluster Survey 2018 (Direct) | 0 |
| | Multiple Indicator Cluster Survey 2023 (Direct) | 1 |
| | WHO Vital Registration Data 2025 version 2025 (VR) | 1 |
| Türkiye | Turkey Demographic Survey 1967 (Direct) | 1 |
| | Turkey Demographic Survey 1967 (Indirect) | 0 |
| | Census 1970 (Indirect) | 1 |
| | Census 1975 (Indirect) | 1 |
| | World Fertility Survey 1978 (Direct) | 1 |
| | World Fertility Survey 1978 (Indirect) | 0 |
| | Census 1980 (Indirect) | 1 |
| | Turkey Population and Health Survey 1983 (Indirect) | 1 |
| | Census 1985 (Indirect) | 1 |
| | Turkey Population and Health Survey 1988 (Direct) | 1 |
| | Turkey Population and Health Survey 1988 (Indirect) | 0 |
| | Demographic Survey 1989 (Indirect) | 1 |
| | Census 1990 (Indirect) | 1 |
| | Demographic and Health Survey 1993 (Direct) | 1 |
| | Demographic and Health Survey 1993 (Indirect) | 0 |
| | Demographic and Health Survey 1998 (Direct) | 1 |
| | Demographic and Health Survey 1998 (Indirect) | 0 |
| | Census 2000 (Indirect) | 1 |
| | National Verbal Autopsy Survey 2003 (Household Deaths) | 0 |
| | Demographic and Health Survey 2003-2004 (Direct) | 1 |
| | Demographic and Health Survey 2003-2004 (Indirect) | 0 |
| | Demographic and Health Survey 2008 (Direct) | 1 |
| | Child Mortality Survey 2010 (Direct) | 0 |
| | Child Mortality Survey 2012 (Direct) | 0 |
| | Demographic and Health Survey 2013 (Direct) | 1 |
| | Demographic and Health Survey 2018 (Indirect) | 0 |
| | WHO Vital Registration Data 2025 version 2025 (VR) | 1 |

| Country | Data series | Inclusion |
|---|---|---|
| Tuvalu | Census 1968 (Indirect) | 1 |
| | Census 1973 (Indirect) | 1 |
| | Census 1979 (Indirect) | 1 |
| | Census 1991 (Indirect) | 1 |
| | Census 2002 (Direct) | 1 |
| | Demographic and Health Survey 2007 (Direct) | 1 |
| | Census 2012 (Indirect) | 1 |
| | Multiple Indicator Cluster Survey 2019-2020 (Direct) | 1 |
| | Recalculated WHO Vital Registration Data 2025 version 2025 (VR) | 1 |
| | WHO Vital Registration Data 2025 version 2025 (VR) | 0 |
| United Republic of Tanzania | Census 1967 (Household Deaths) | 0 |
| | Census 1967 (Indirect) | 1 |
| | National Demographic Survey 1973 (Household Deaths) | 0 |
| | Census 1978 (Indirect) | 1 |
| | Census 1988 (Indirect) | 1 |
| | Census 1988 (Household Deaths) | 0 |
| | Demographic and Health Survey 1991-1992 (Direct) | 0 |
| | Demographic and Health Survey 1991-1992 (Indirect) | 0 |
| | Demographic and Health Survey (MM adjusted) 1991-1992 (Direct) | 1 |
| | Demographic and Health Survey 1996 (Direct) | 0 |
| | Demographic and Health Survey 1996 (Indirect) | 0 |
| | Demographic and Health Survey (MM adjusted) 1996 (Direct) | 1 |
| | Demographic and Health Survey 1999 (Direct) | 0 |
| | Demographic and Health Survey 1999 (Indirect) | 0 |
| | Demographic and Health Survey (MM adjusted) 1999 (Direct) | 1 |
| | Census 2002 (Indirect) | 1 |
| | Census 2002 (Household Deaths) | 0 |
| | Demographic and Health Survey 2004-2005 (Direct) | 0 |
| | Demographic and Health Survey 2004-2005 (Indirect) | 0 |
| | Demographic and Health Survey (MM adjusted) 2004-2005 (Direct) | 1 |
| | AIDS Indicator Survey 2007-2008 (Household Deaths) | 0 |
| | AIDS Indicator Survey 2007-2008 (Direct) | 0 |
| | AIDS Indicator Survey 2007-2008 (Indirect) | 0 |

| Country | Data series | Inclusion |
| --- | --- | --- |
| | AIDS Indicator Survey (MM adjusted) 2007-2008 (Direct) | 1 |
| | National Panel Survey 2008-2009 (Household Deaths) | 0 |
| | Demographic and Health Survey 2010 (Direct) | 0 |
| | Demographic and Health Survey (MM adjusted) 2010 (Direct) | 1 |
| | National Panel Survey 2010-2011 (Household Deaths) | 0 |
| | HIV/AIDS and Malaria Indicator Survey 2011 (Indirect) | 0 |
| | Census 2012 (Indirect) | 1 |
| | National Panel Survey 2012-2013 (Household Deaths) | 0 |
| | Demographic and Health Survey 2015-2016 (Direct) | 0 |
| | Demographic and Health Survey (MM adjusted) 2015-2016 (Direct) | 1 |
| | Malaria Indicator Survey 2017 (Indirect) | 1 |
| | Demographic and Health Survey 2022 (Direct) | 0 |
| | Demographic and Health Survey (MM adjusted) 2022 (Direct) | 1 |
| | Census 2022 (Indirect) | 1 |
| Uganda | Census 1969 (Indirect) | 1 |
| | Demographic and Health Survey 1988-1989 (Direct) | 0 |
| | Demographic and Health Survey 1988-1989 (Indirect) | 0 |
| | Demographic and Health Survey (MM adjusted) 1988-1989 (Direct) | 1 |
| | Census 1991 (Direct) | 1 |
| | Demographic and Health Survey 1995 (Direct) | 0 |
| | Demographic and Health Survey 1995 (Indirect) | 0 |
| | Demographic and Health Survey (MM adjusted) 1995 (Direct) | 1 |
| | Demographic and Health Survey 2000-2001 (Direct) | 0 |
| | Demographic and Health Survey 2000-2001 (Indirect) | 0 |
| | Demographic and Health Survey (MM adjusted) 2000-2001 (Direct) | 1 |
| | Census 2002 (Direct) | 1 |
| | Census 2002 (Indirect) | 1 |
| | Census 2002 (Household Deaths) | 0 |
| | Demographic and Health Survey 2006 (Household Deaths) | 0 |
| | Demographic and Health Survey 2006 (Direct) | 0 |
| | Demographic and Health Survey 2006 (Indirect) | 0 |
| | Demographic and Health Survey (MM adjusted) 2006 (Direct) | 1 |
| | Malaria Indicator Survey 2009-2010 (Direct) | 0 |

| Country | Data series | Inclusion |
| --- | --- | --- |
| | Malaria Indicator Survey 2009-2010 (Indirect) | 0 |
| | Malaria Indicator Survey (MM adjusted) 2009-2010 (Direct) | 1 |
| | AIDS Indicator Survey 2011 (Household Deaths) | 0 |
| | Demographic and Health Survey 2011 (Direct) | 0 |
| | Demographic and Health Survey (MM adjusted) 2011 (Direct) | 1 |
| | Census 2014 (Indirect) | 1 |
| | Malaria Indicator Survey 2014-2015 (Indirect) | 1 |
| | Demographic and Health Survey 2016 (Direct) | 0 |
| | Demographic and Health Survey (MM adjusted) 2016 (Direct) | 1 |
| | Malaria Indicator Survey 2018-2019 (Indirect) | 1 |
| | Uganda Demographic and Health Survey 2022 (Direct) | 0 |
| | Uganda Demographic and Health Survey (MM adjusted) 2022 (Direct) | 1 |
| | Census 2024 (Household Deaths) | 1 |
| | Census 2024 (Indirect) | 0 |
| Ukraine | Vital Registration Data from Human Mortality Database (VR) | 0 |
| | Vital Registration Data from State Statistics Committee of Ukraine (VR) | 0 |
| | Census 1989 (Indirect) | 1 |
| | Reproductive Health Survey 1999 (Direct) | 1 |
| | Census 2001 (Indirect) | 1 |
| | Multiple Indicator Cluster Survey 2005 (Indirect) | 0 |
| | Demographic and Health Survey 2007 (Direct) | 1 |
| | Demographic and Health Survey 2007 (Indirect) | 0 |
| | Multiple Indicator Cluster Survey 2012 (Indirect) | 0 |
| | Multiple Indicator Cluster Survey 2012 (Direct) | 0 |
| | WHO Vital Registration Data 2025 version 2025 (VR) | 1 |
| Uruguay | TABLAS DE VIDA NACIONALES (Life Table) | 0 |
| | Census 1975 (Indirect) | 1 |
| | Census 1985 (Indirect) | 1 |
| | Census 1996 (Indirect) | 1 |
| | Census 2010 (Indirect) | 1 |
| | UNPD Vital Registration Data 2022 version 2022 (VR) | 1 |
| | WHO Vital Registration Data 2025 version 2025 (VR) | 1 |
| United States | WHO Vital Registration Data 2025 version 2025 (VR) | 1 |

| Country | Data series | Inclusion |
|---|---|---|
| Uzbekistan | Demographic and Health Survey 1996 (Direct) | 0 |
| | Demographic and Health Survey 1996 (Indirect) | 0 |
| | Multiple Indicator Cluster Survey 2000 (Indirect) | 1 |
| | Demographic and Health Survey 2002 (Direct) | 1 |
| | Demographic and Health Survey 2002 (Indirect) | 0 |
| | Multiple Indicator Cluster Survey 2006 (Indirect) | 1 |
| | Vital registration from National Statistical Office 2017 (VR) | 0 |
| | Multiple Indicator Cluster Survey 2021-2022 (Direct) | 1 |
| | WHO Vital Registration Data 2025 version 2025 (VR) | 1 |
| Saint Vincent and the Grenadines | Recalculated WHO Vital Registration Data 2025 version 2025 (VR) | 1 |
| | WHO Vital Registration Data 2025 version 2025 (VR) | 0 |
| Venezuela (Bolivarian Republic of) | TABLAS DE VIDA NACIONALES (Life Table) | 1 |
| | World Fertility Survey 1977 (Direct) | 1 |
| | World Fertility Survey 1977 (Indirect) | 0 |
| | Census 1981 (Indirect) | 1 |
| | Census 1990 (Indirect) | 1 |
| | National Population and Family Survey 1998 (Direct) | 1 |
| | Census 2001 (Indirect) | 1 |
| | Vital Registration Data from Ministerio del Poder Popular para la Planificacion y Desarrollo (May 2009) 2009 (VR) | 0 |
| | Census 2011 (Indirect) | 1 |
| | Vital Registration Data from Instituto Nacional de Estadistica 2014 (VR) | 0 |
| | Vital Registration Data from Ministerio del Poder Popular para la Salud 2017 (VR) | 0 |
| | Provisional Adjusted Data from Ministerio del Poder Popular para la Salud and Instituto Nacional de Estadistica 2019 (VR) | 0 |
| | Encuesta Nacional sobre Condiciones de Vida (ENCOVI) 2019-2020 (Indirect) | 1 |
| | Encuesta Nacional sobre Condiciones de Vida (ENCOVI) 2021 (Indirect) | 0 |
| | UNPD Demographic Yearbook Data 2023 version 2023 (VR) | 1 |
| | WHO Vital Registration Data 2025 version 2025 (VR) | 1 |
| British Virgin Islands | Recalculated UNPD Vital Registration Data 2022 version 2022 (VR) | 1 |
| | UNPD Vital Registration Data 2022 version 2022 (VR) | 0 |
| Viet Nam | National Demographic and Health Survey 1988 (Indirect) | 0 |
| | Census 1989 (Indirect) | 1 |
| | National Demographic and Health Survey 1988 (Direct) | 1 |

| Country | Data series | Inclusion |
|---|---|---|
| | Intercensal Demographic Survey 1994 (Indirect) | 0 |
| | Intercensal Demographic Survey 1994 (Direct) | 1 |
| | Demographic and Health Survey 1997 (Direct) | 1 |
| | Demographic and Health Survey 1997 (Indirect) | 0 |
| | Vietnam Longitudinal Survey (VLSS) 1998 (Direct) | 1 |
| | Population and Housing Census 1999 (Household Deaths) | 0 |
| | Population and Housing Census 1999 (Indirect) | 0 |
| | Multiple Indicator Cluster Survey 2000 (Indirect) | 1 |
| | Demographic and Health Survey 2002 (Direct) | 1 |
| | Demographic and Health Survey 2002 (Indirect) | 0 |
| | Annual Population Change Survey 2005 (Indirect) | 0 |
| | Population and AIDS Indicator Survey 2005 (Indirect) | 0 |
| | Multiple Indicator Cluster Survey 2006 (Indirect) | 0 |
| | Population Change and Family Planning Survey 2006 (Indirect) | 0 |
| | Population Change and Family Planning Survey 2007 (Household Deaths) | 0 |
| | Population Change and Family Planning Survey 2007 (Indirect) | 0 |
| | Population Change and Family Planning Survey 2008 (Indirect) | 0 |
| | Annual Population Change Survey 2009 (Indirect) | 0 |
| | Census 2009 (Indirect) | 0 |
| | Population Change and Family Planning Survey 2010 (Indirect) | 0 |
| | Population Change and Family Planning Survey 2011 (Indirect) | 0 |
| | Multiple Indicator Cluster Survey 2010-2011 (Indirect) | 0 |
| | Population Change and Family Planning Survey 2011 (Household Deaths) | 1 |
| | Population Change and Family Planning Survey 2012 (Household Deaths) | 1 |
| | Population Change and Family Planning Survey 2012 (Indirect) | 0 |
| | Population Change and Family Planning Survey 2013 (Household Deaths) | 1 |
| | Population Change and Family Planning Survey 2013 (Indirect) | 0 |
| | Intercensal Population and Housing Survey 2014 (Household Deaths) | 1 |
| | Multiple Indicator Cluster Survey 2013-2014 (Direct) | 1 |
| | Population Change and Family Planning Survey 2015 (Household Deaths) | 1 |
| | Population Change and Family Planning Survey 2015 (Indirect) | 0 |
| | Population Change and Family Planning Survey 2016 (Household Deaths) | 1 |
| | Population Change and Family Planning Survey 2016 (Indirect) | 0 |

| Country | Data series | Inclusion |
|---|---|---|
| | Population Change and Family Planning Survey 2017 (Household Deaths) | 1 |
| | Population Change and Family Planning Survey 2017 (Indirect) | 0 |
| | Population Change and Family Planning Survey 2018 (Household Deaths) | 1 |
| | Population Change and Family Planning Survey 2018 (Indirect) | 0 |
| | Census 2019 (Household Deaths) | 1 |
| | Census 2019 (Indirect) | 0 |
| | Population Change and Family Planning Survey 2020 (Household Deaths) | 1 |
| | Multiple Indicator Cluster Survey 2020-2021 (Direct) | 0 |
| | Population Change and Family Planning Survey 2021 (Household Deaths) | 1 |
| | Population Change and Family Planning Survey 2022 (Household Deaths) | 1 |
| | Population Change and Family Planning Survey 2023 (Household Deaths) | 1 |
| | Population Change and Family Planning Survey 2024 (Household Deaths) | 1 |
| Vanuatu | Census 1967 (Indirect) | 1 |
| | Census 1989 (Indirect) | 1 |
| | Census 1999 (Indirect) | 1 |
| | Multiple Indicator Cluster Survey 2007-2008 (Indirect) | 0 |
| | Census 2009 (Household Deaths) | 0 |
| | Census 2009 (Indirect) | 1 |
| | Demographic and Health Survey 2013 (Direct) | 1 |
| | Census 2020 (Indirect) | 1 |
| | Multiple Indicator Cluster Survey 2023 (Direct) | 1 |
| Samoa | Census 1956 (Indirect) | 1 |
| | Census 1961 (Indirect) | 1 |
| | Census 1966 (Indirect) | 1 |
| | Census 1971 (Indirect) | 0 |
| | Census 1976 (Indirect) | 1 |
| | Census 1981 (Indirect) | 1 |
| | Demographic and Health Survey 1999 (Household Deaths) | 1 |
| | Demographic and Health Survey 1999 (Indirect) | 1 |
| | Demographic and Vital Statistics Survey 2000 (Indirect) | 1 |
| | Population and Housing Census 2001 (Indirect) | 1 |
| | Population and Housing Census 2006 (Household Deaths) | 0 |
| | Population and Housing Census 2006 (Indirect) | 1 |

| Country | Data series | Inclusion |
| --- | --- | --- |
| | Demographic and Health Survey 2009 (Direct) | 0 |
| | Demographic and Health Survey 2009 (Indirect) | 0 |
| | Population and Housing Census 2011 (Household Deaths) | 1 |
| | Demographic and Health Survey 2014 (Direct) | 0 |
| | Population and Housing Census 2016 (Household Deaths) | 0 |
| | Population and Housing Census 2016 (Indirect) | 1 |
| | Multiple Indicator Cluster Survey 2019-2020 (Direct) | 1 |
| | WHO Vital Registration Data 2025 version 2025 (VR) | 0 |
| Yemen | World Fertility Survey 1979 (Direct) | 1 |
| | World Fertility Survey 1979 (Indirect) | 0 |
| | Demographic and Health Survey 1991-1992 (Direct) | 1 |
| | Demographic and Health Survey 1991-1992 (Indirect) | 0 |
| | Census 1994 (Indirect) | 1 |
| | Demographic and Health Survey 1997 (Direct) | 1 |
| | Demographic and Health Survey 1997 (Indirect) | 0 |
| | PAPFAM Family Health Survey 2003 (Direct) | 1 |
| | Census 2004 (Household Deaths) | 1 |
| | Census 2004 (Indirect) | 1 |
| | Multiple Indicator Cluster Survey 2006 (Direct) | 1 |
| | Multiple Indicator Cluster Survey 2006 (Indirect) | 0 |
| | Demographic and Health Survey 2013 (Direct) | 1 |
| | Multiple Indicator Cluster Survey 2022-2023 (Direct) | 1 |
| South Africa | Demographic and Health Survey 1987-1989 (Direct) | 0 |
| | HSRC 1990 (Direct) | 1 |
| | Household Survey 1993 (Household Deaths) | 0 |
| | Household Survey 1995 (Household Deaths) | 0 |
| | Household Survey 1996 (Household Deaths) | 0 |
| | Census 1996 (Indirect) | 1 |
| | Household Survey 1997 (Household Deaths) | 0 |
| | Household Survey 1998 (Household Deaths) | 0 |
| | Demographic and Health Survey 1998 (Direct) | 0 |
| | Demographic and Health Survey 1998 (Indirect) | 0 |
| | Demographic and Health Survey (MM adjusted) 1998 (Direct) | 1 |

| Country | Data series | Inclusion |
| --- | --- | --- |
| | Census 2001 (Household Deaths) | 1 |
| | Census 2001 (Indirect) | 0 |
| | Demographic and Health Survey 2004 (Direct) | 0 |
| | Demographic and Health Survey (MM adjusted) 2004 (Direct) | 0 |
| | Community Survey 2007 (Household Deaths) | 1 |
| | Census 2011 (Household Deaths) | 0 |
| | Community Survey 2016 (Indirect) | 0 |
| | Demographic and Health Survey 2016 (Direct) | 0 |
| | Demographic and Health Survey (MM adjusted) 2016 (Direct) | 1 |
| | Rapid Mortality Surveillance 2024 (VR) | 1 |
| | WHO Vital Registration Data 2025 version 2025 (VR) | 0 |
| Zambia | Census 1969 (Indirect) | 1 |
| | Sample Census of Population 1974 (Indirect) | 1 |
| | Census 1980 (Indirect) | 1 |
| | Census 1990 (Indirect) | 1 |
| | Demographic and Health Survey 1992 (Direct) | 0 |
| | Demographic and Health Survey 1992 (Indirect) | 0 |
| | Demographic and Health Survey (MM adjusted) 1992 (Direct) | 1 |
| | Demographic and Health Survey 1996-1997 (Direct) | 0 |
| | Demographic and Health Survey 1996-1997 (Indirect) | 0 |
| | Demographic and Health Survey (MM adjusted) 1996-1997 (Direct) | 1 |
| | Census 2000 (Indirect) | 1 |
| | Demographic and Health Survey 2001-2002 (Direct) | 0 |
| | Demographic and Health Survey 2001-2002 (Indirect) | 0 |
| | Demographic and Health Survey (MM adjusted) 2001-2002 (Direct) | 1 |
| | Demographic and Health Survey 2007 (Indirect) | 1 |
| | Demographic and Health Survey 2007 (Household Deaths) | 0 |
| | Demographic and Health Survey 2007 (Direct) | 0 |
| | Demographic and Health Survey (MM adjusted) 2007 (Direct) | 1 |
| | Global Fund Evaluation Survey 2008 (Household Deaths) | 0 |
| | Census 2010 (Household Deaths) | 0 |
| | Census 2010 (Indirect) | 1 |
| | Demographic and Health Survey 2013-2014 (Direct) | 0 |

| Country | Data series | Inclusion |
| --- | --- | --- |
| | Demographic and Health Survey (MM adjusted) 2013-2014 (Direct) | 1 |
| | Sample Registration System with Verbal Autopsy 2015-2016 (VR) | 0 |
| | Demographic and Health Survey 2018 (Direct) | 0 |
| | Demographic and Health Survey (MM adjusted) 2018 (Direct) | 1 |
| | Demographic and Health Survey 2024 (Direct) | 0 |
| | Demographic and Health Survey (MM adjusted) 2024 (Direct) | 1 |
| Zimbabwe | Census 1969 (Indirect) | 1 |
| | Census 1982 (Indirect) | 1 |
| | Reproductive Health Survey 1984 (Indirect) | 1 |
| | Inter-censal Demographic Survey 1987 (Indirect) | 1 |
| | Demographic and Health Survey 1988-1989 (Direct) | 0 |
| | Demographic and Health Survey 1988-1989 (Indirect) | 0 |
| | Demographic and Health Survey (MM adjusted) 1988-1989 (Direct) | 1 |
| | Census 1992 (Indirect) | 1 |
| | Census 1992 (Household Deaths) | 0 |
| | Demographic and Health Survey 1994 (Direct) | 0 |
| | Demographic and Health Survey 1994 (Indirect) | 0 |
| | Demographic and Health Survey (MM adjusted) 1994 (Direct) | 1 |
| | Inter-censal Demographic Survey 1997 (Household Deaths) | 0 |
| | Inter-censal Demographic Survey 1997 (Indirect) | 1 |
| | Demographic and Health Survey 1999 (Direct) | 0 |
| | Demographic and Health Survey 1999 (Indirect) | 0 |
| | Demographic and Health Survey (MM adjusted) 1999 (Direct) | 1 |
| | Census 2002 (Indirect) | 1 |
| | Census 2002 (Household Deaths) | 0 |
| | Demographic and Health Survey 2005-2006 (Household Deaths) | 0 |
| | Demographic and Health Survey 2005-2006 (Direct) | 0 |
| | Demographic and Health Survey 2005-2006 (Indirect) | 0 |
| | Demographic and Health Survey (MM adjusted) 2005-2006 (Direct) | 0 |
| | Multiple Indicator Cluster Survey 2009 (Direct) | 0 |
| | Multiple Indicator Cluster Survey (MM adjusted) 2009 (Direct) | 1 |
| | Demographic and Health Survey 2010-2011 (Direct) | 0 |
| | Demographic and Health Survey (MM adjusted) 2010-2011 (Direct) | 1 |

| Country | Data series | Inclusion |
|---|---|---|
| | Census 2012 (Household Deaths) | 0 |
| | Multiple Indicator Cluster Survey 2014 (Direct) | 0 |
| | Multiple Indicator Cluster Survey (MM adjusted) 2014 (Direct) | 1 |
| | Demographic and Health Survey 2015 (Direct) | 0 |
| | Demographic and Health Survey (MM adjusted) 2015 (Direct) | 1 |
| | Inter-censal Demographic Survey 2017 (Household Deaths) | 0 |
| | Multiple Indicator Cluster Survey 2019 (Direct) | 0 |
| | Multiple Indicator Cluster Survey (MM adjusted) 2019 (Direct) | 1 |
| | Census 2022 (Household Deaths) | 0 |
| | Demographic and Health Survey 2023-2024 (Direct) | 0 |
| | Demographic and Health Survey (MM adjusted) 2023-2024 (Direct) | 1 |

# 4. Supplementary figures: Estimates, with underlying data, by country

## Afghanistan (AFG)

### Under-five mortality rate

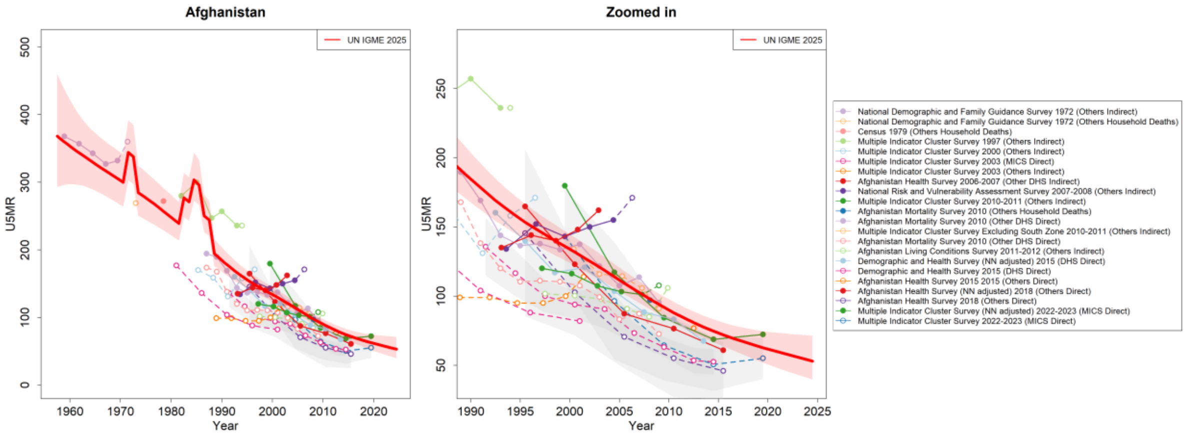


### Infant mortality rate

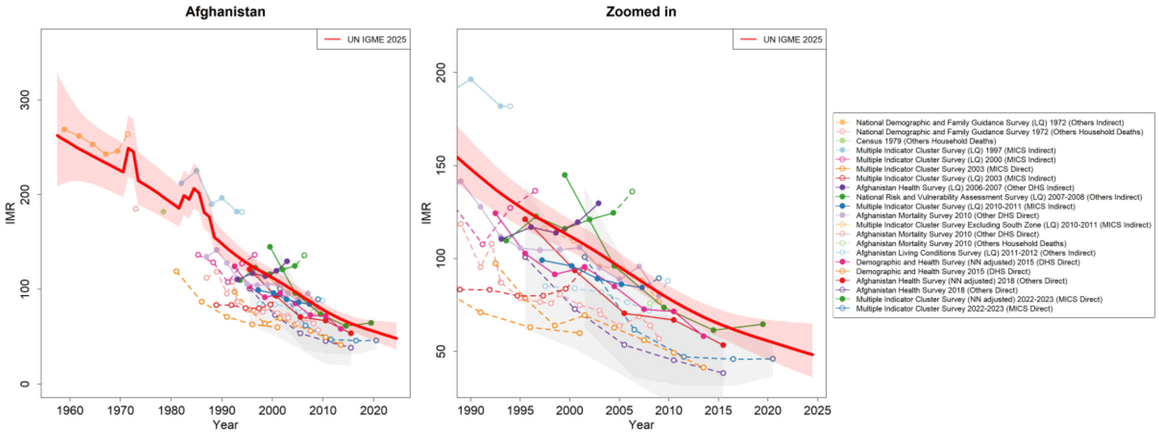


### Neonatal mortality rate

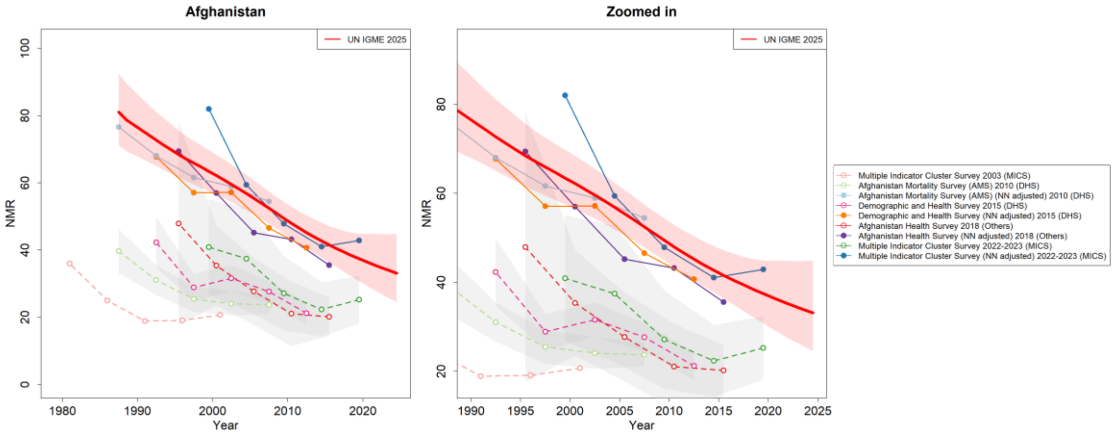

## Albania (ALB)

### Under-five mortality rate

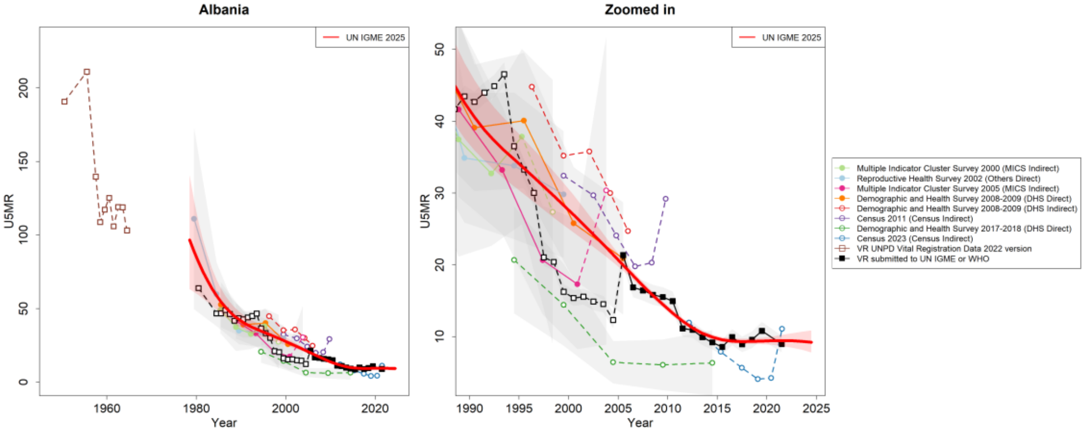


### Infant mortality rate

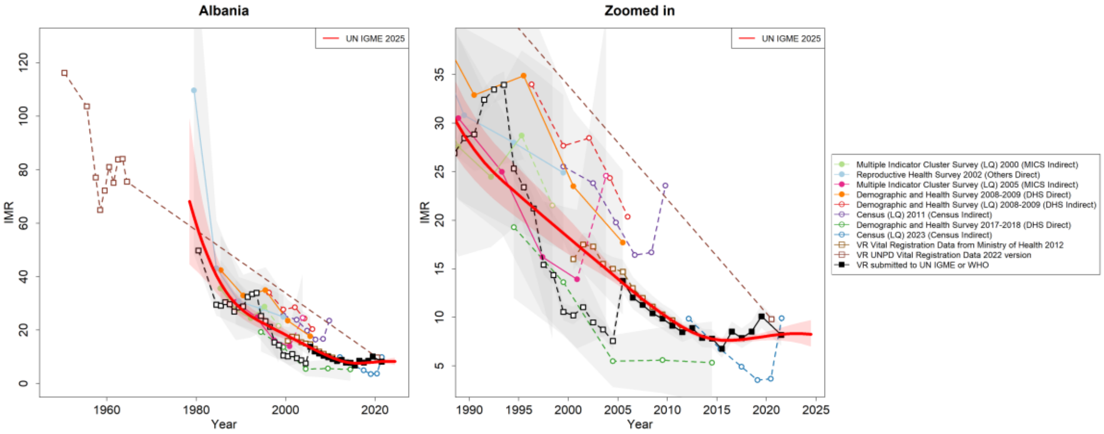


### Neonatal mortality rate

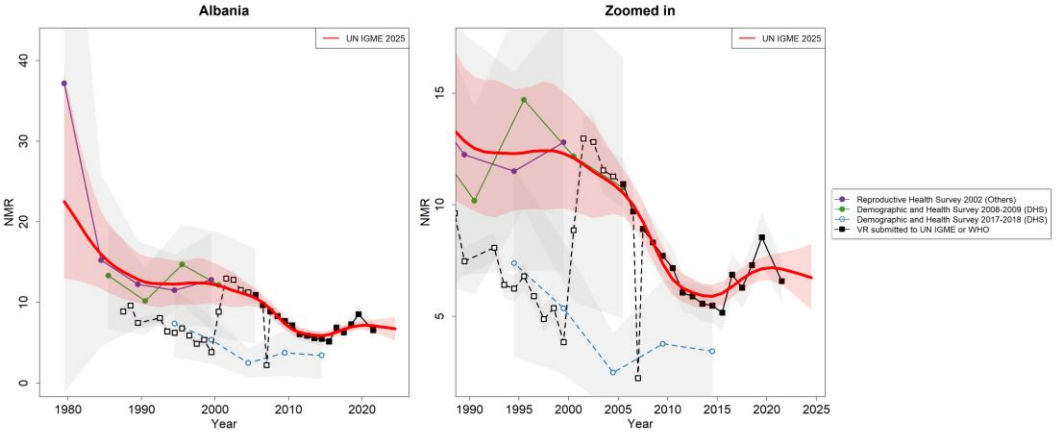

## Algeria (DZA)

### Under-five mortality rate

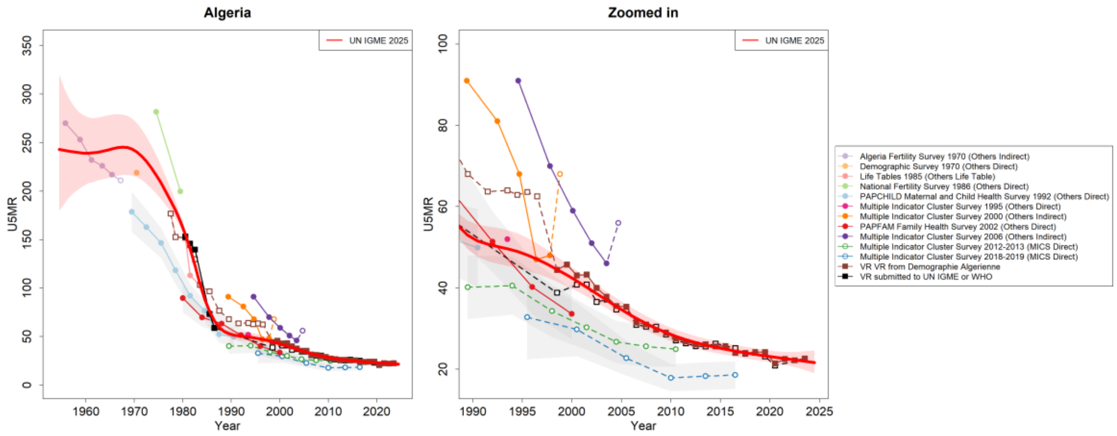


### Infant mortality rate

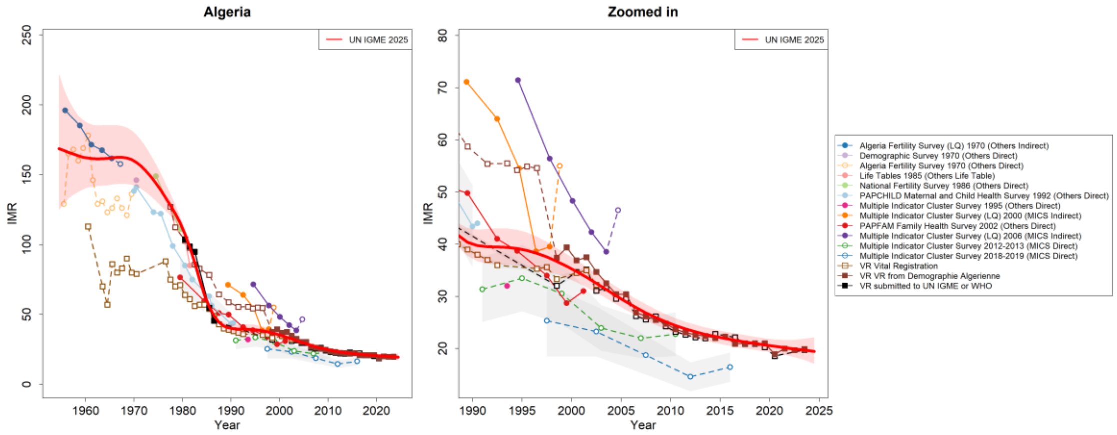


### Neonatal mortality rate

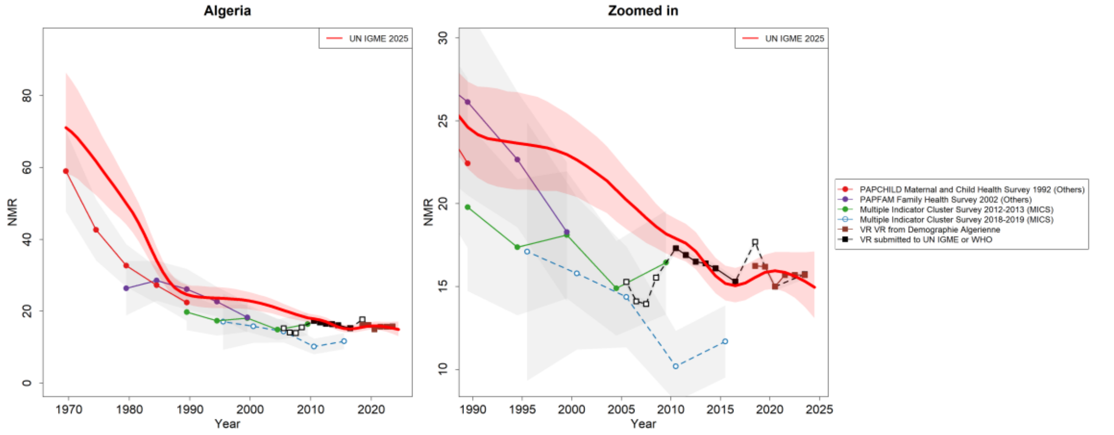

## Andorra (AND)

### Under-five mortality rate

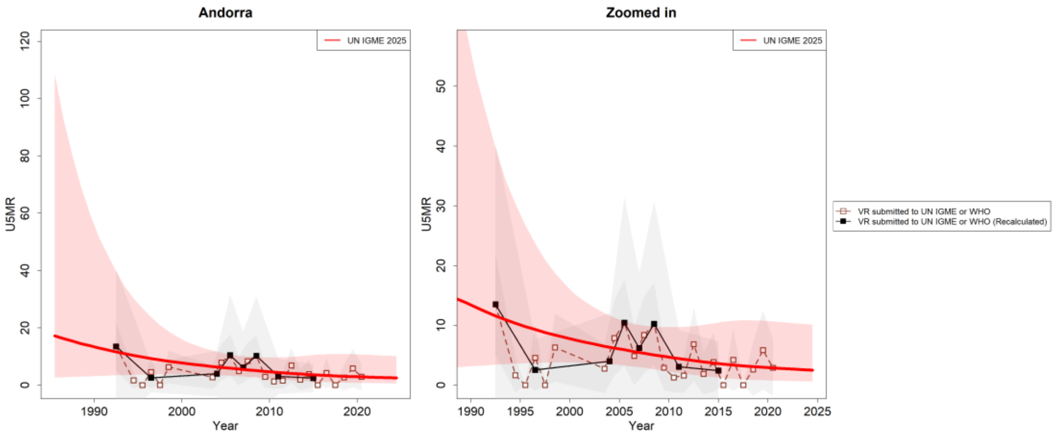


### Infant mortality rate

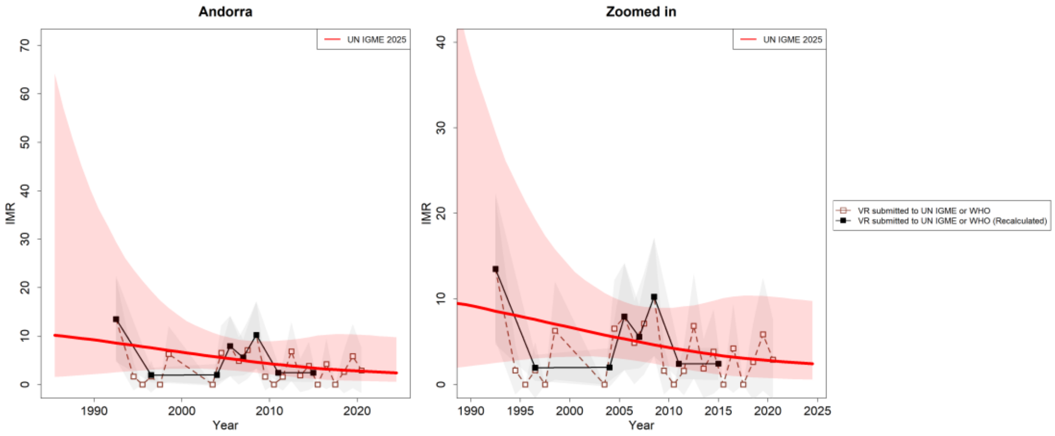


### Neonatal mortality rate

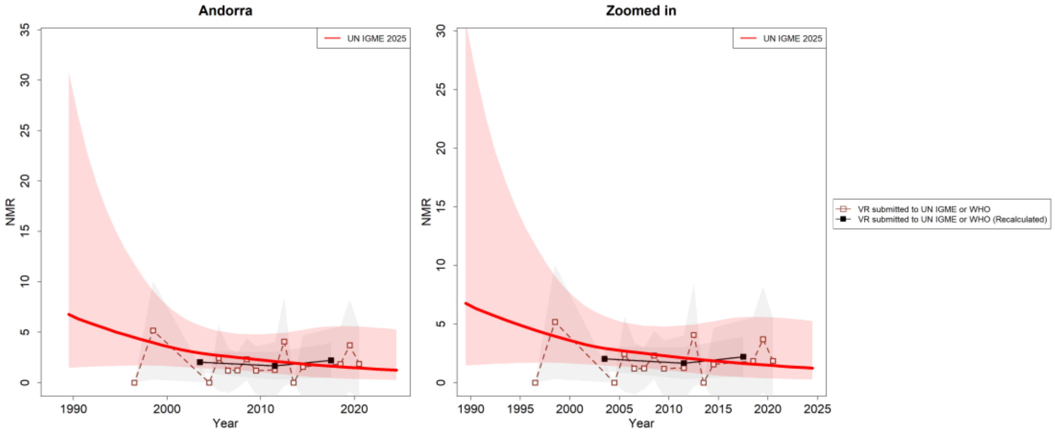

## Angola (AGO)

### Under-five mortality rate

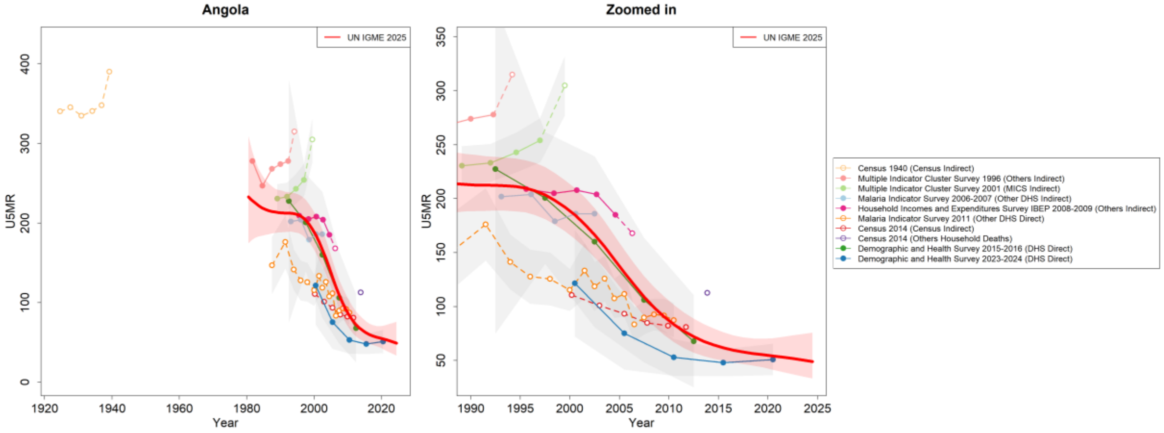


### Infant mortality rate

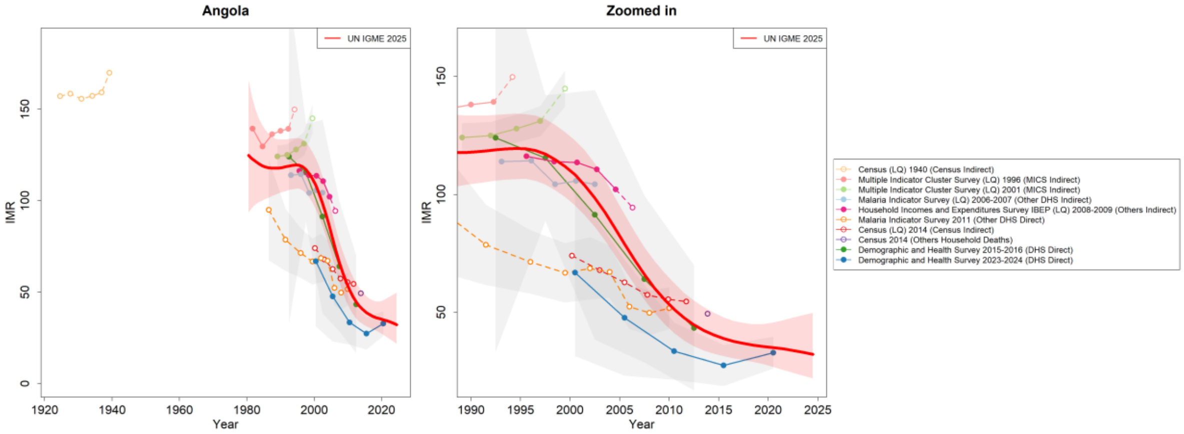


### Neonatal mortality rate

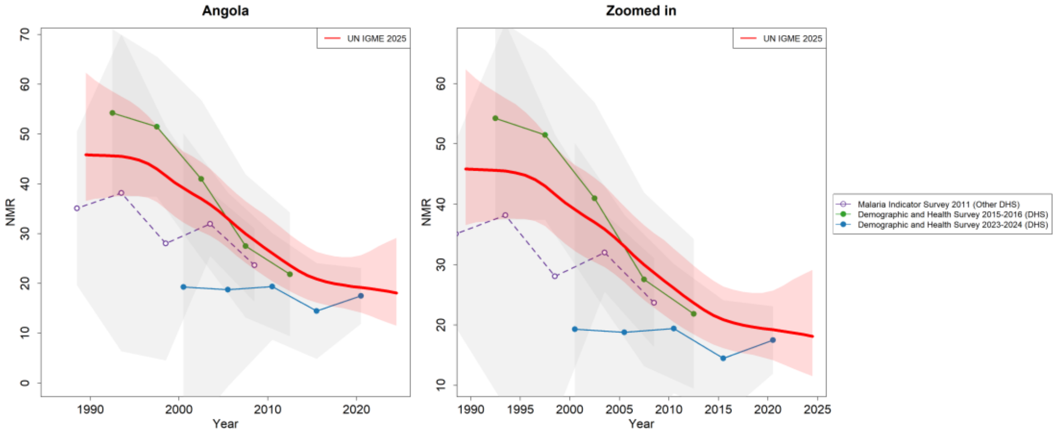

## Anguilla (AIA)

### Under-five mortality rate

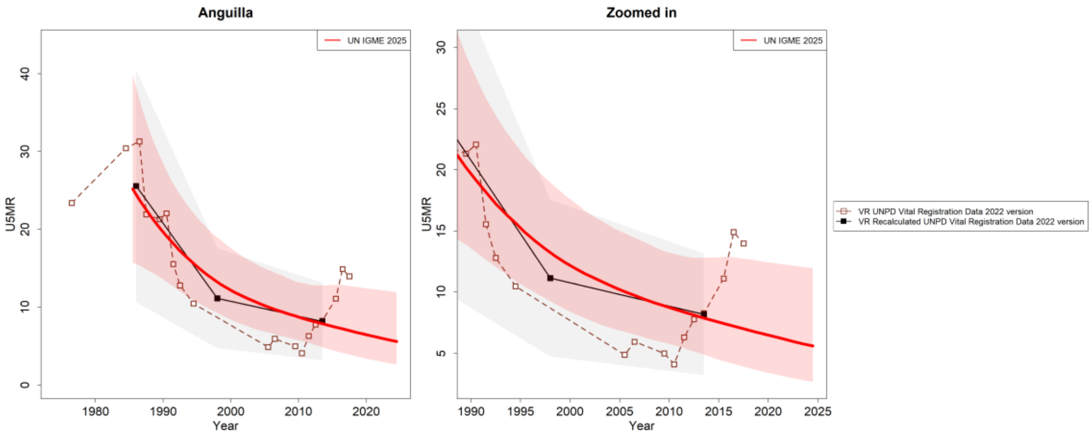


### Infant mortality rate

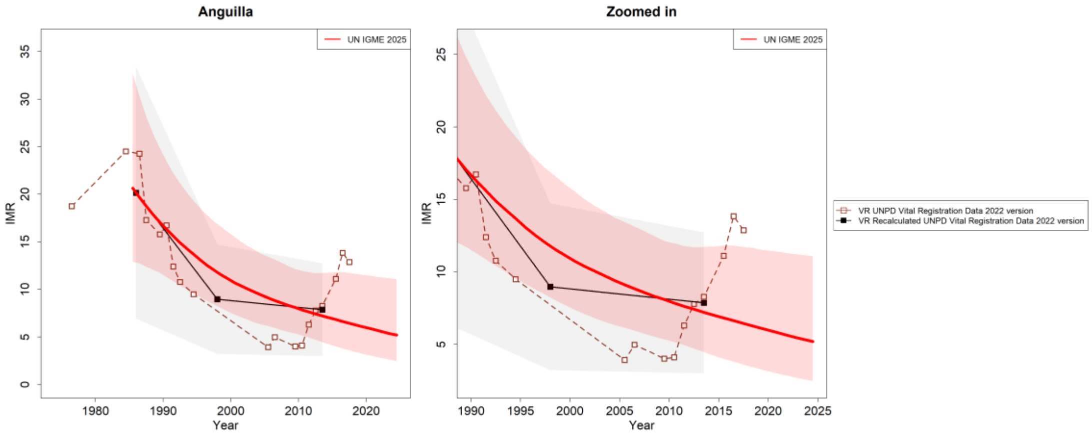


### Neonatal mortality rate

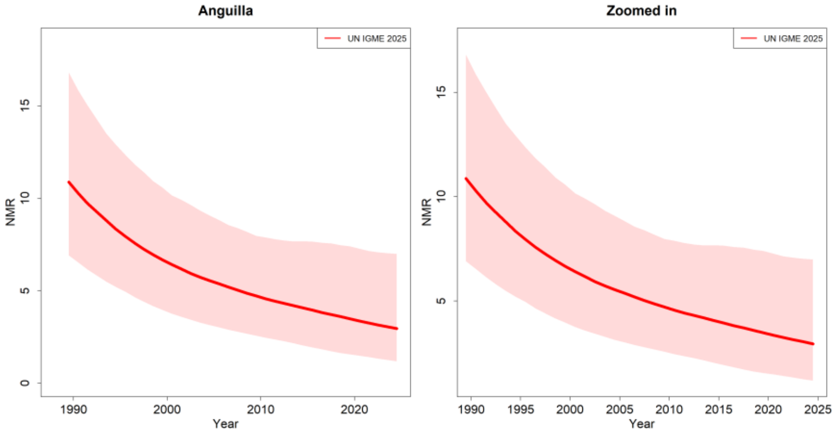

## Antigua and Barbuda (ATG)

### Under-five mortality rate

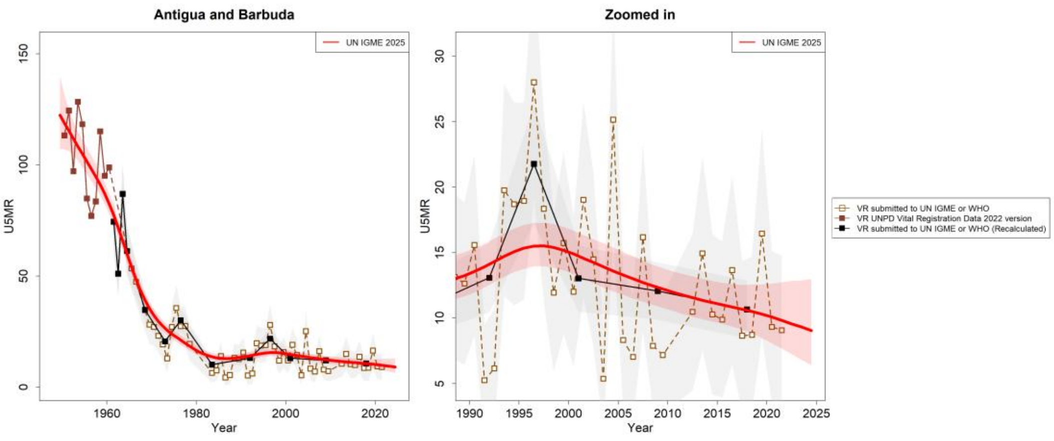


### Infant mortality rate

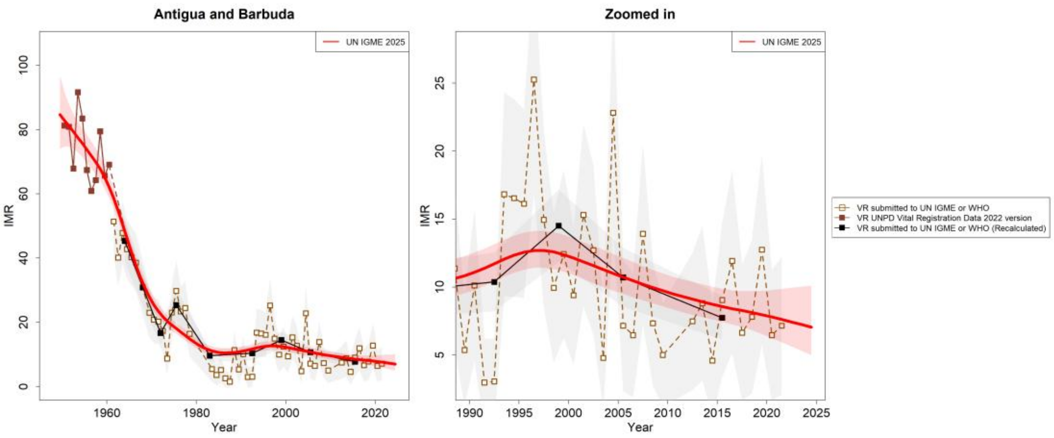


### Neonatal mortality rate

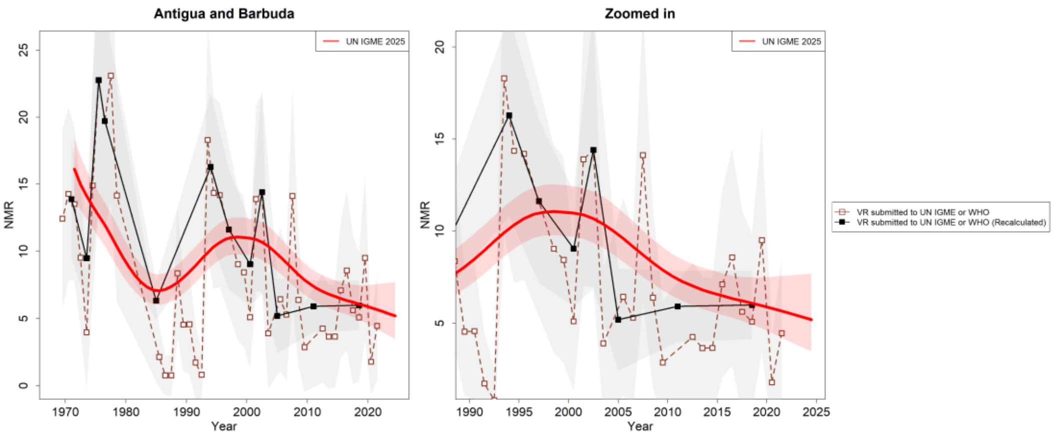

## Argentina (ARG)

### Under-five mortality rate

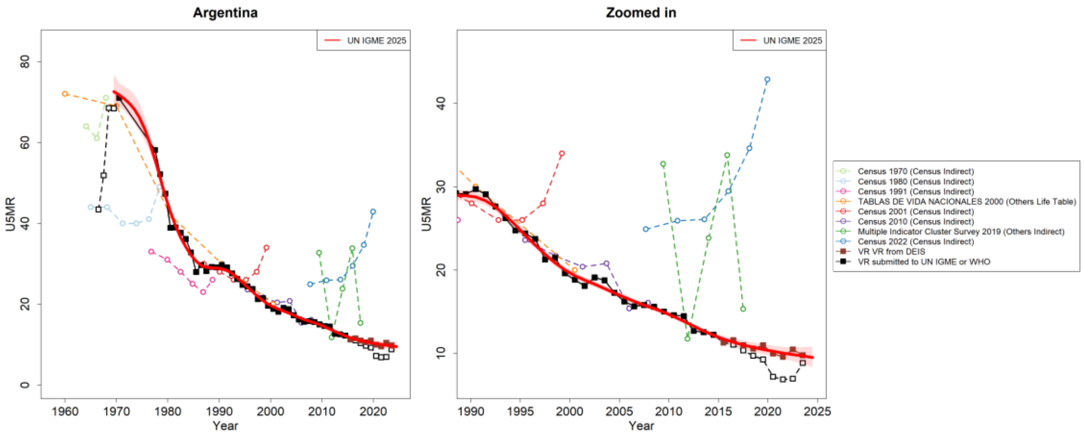


### Infant mortality rate

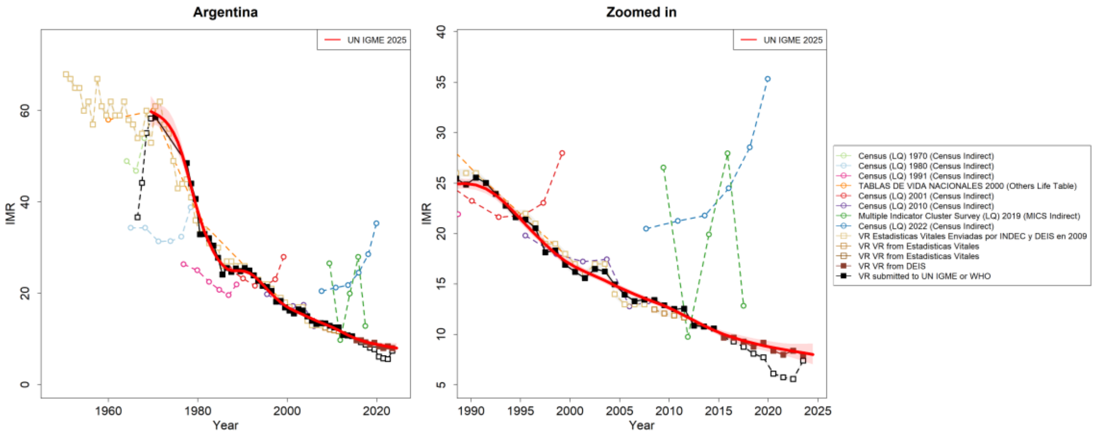


### Neonatal mortality rate

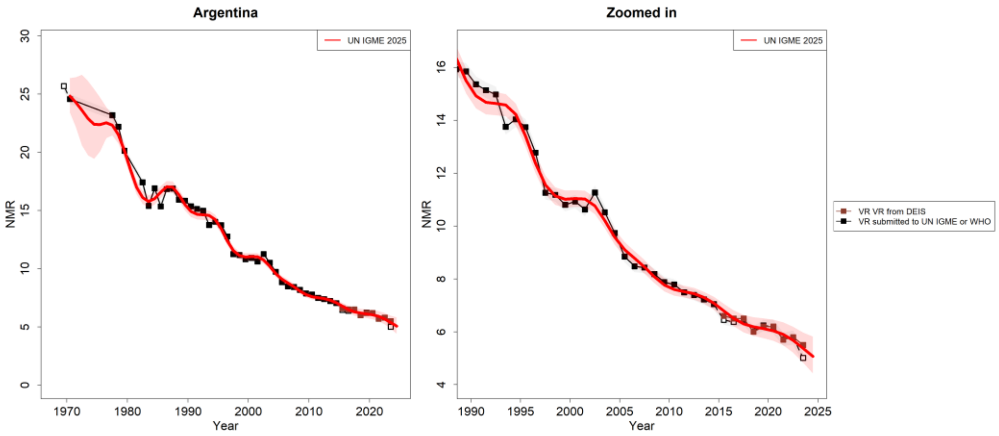

## Armenia (ARM)

### Under-five mortality rate

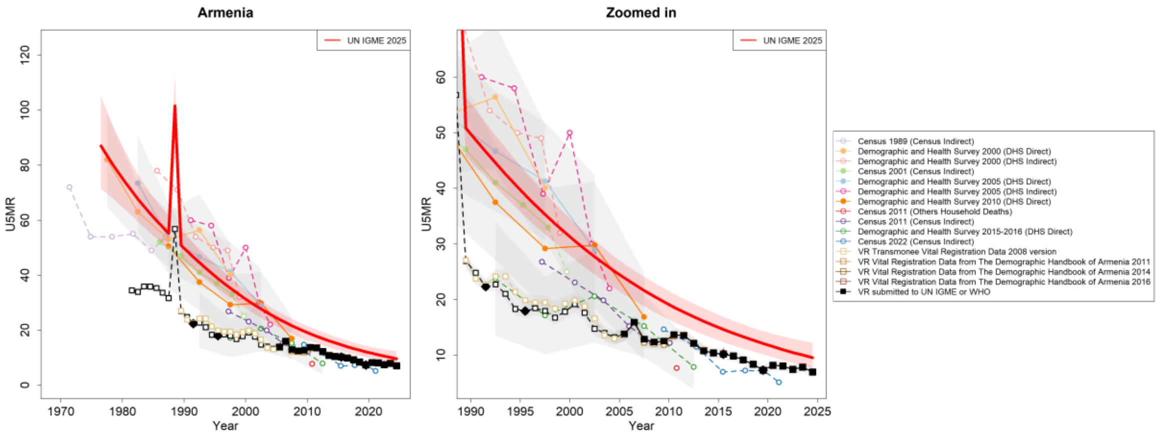


### Infant mortality rate

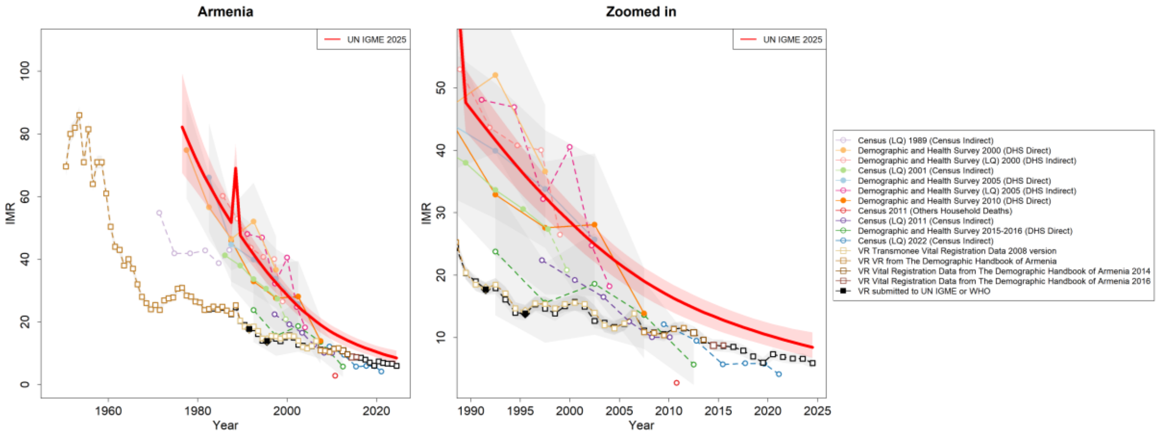


### Neonatal mortality rate

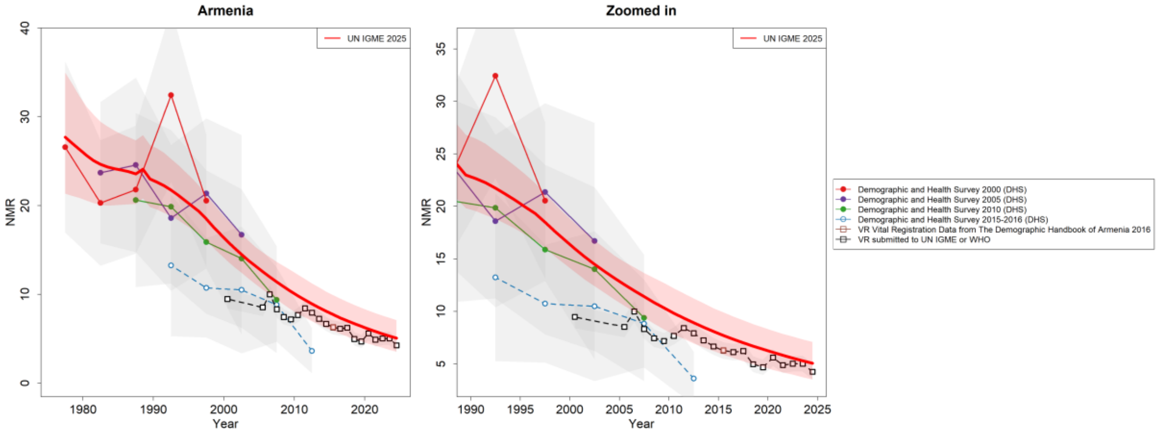

## Australia (AUS)

### Under-five mortality rate

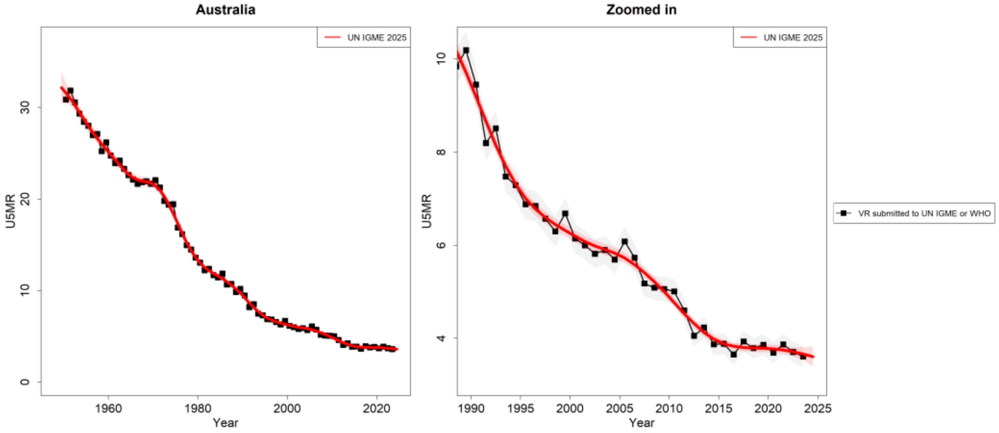


### Infant mortality rate

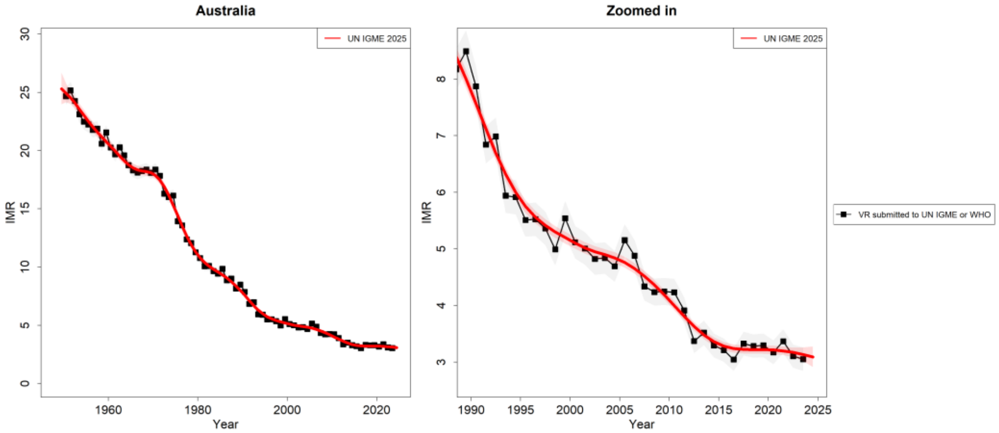


### Neonatal mortality rate

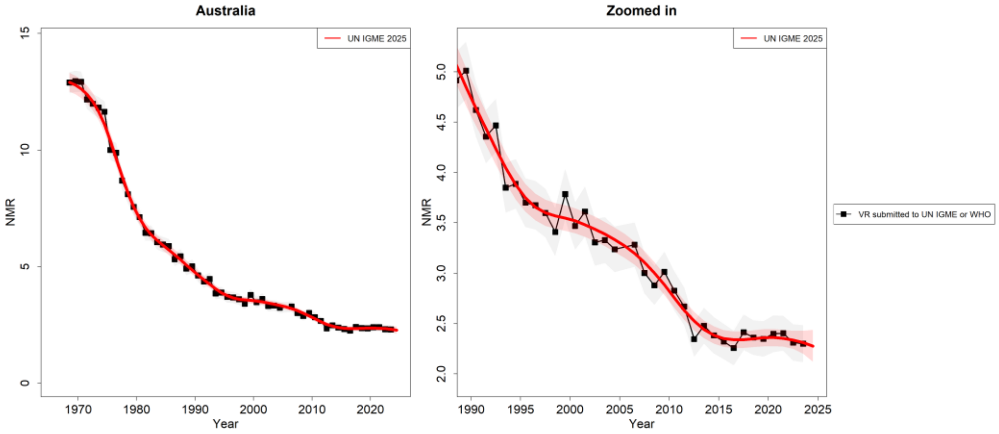

## Austria (AUT)

### Under-five mortality rate

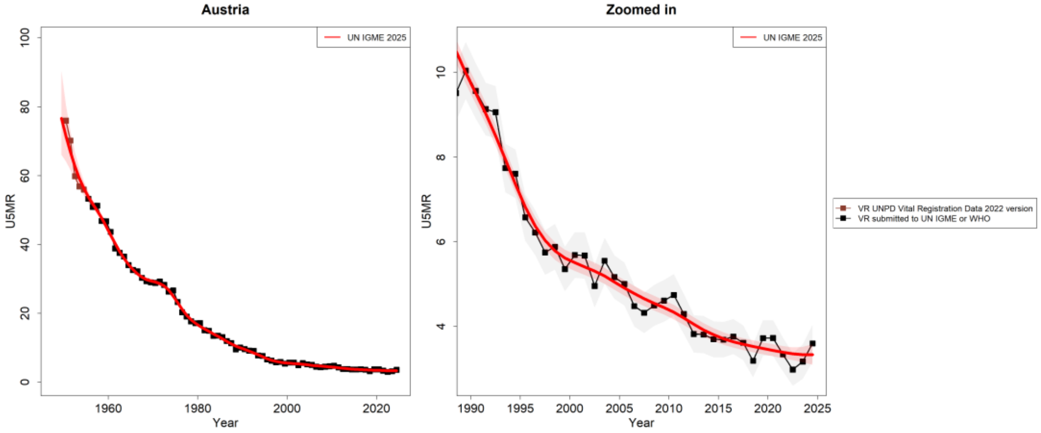


### Infant mortality rate

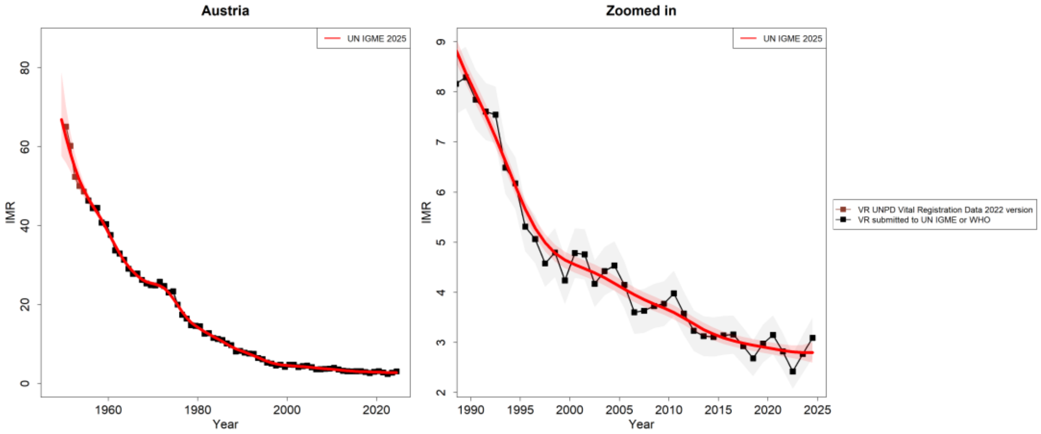


### Neonatal mortality rate

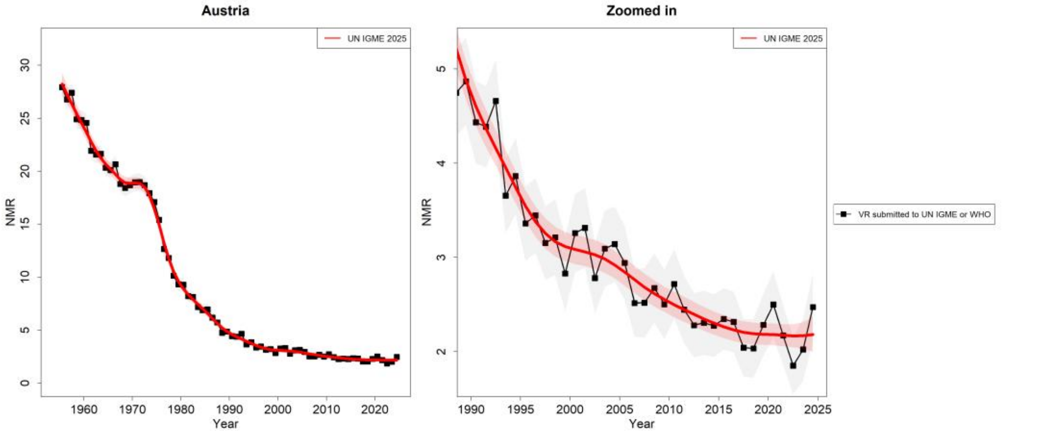

## Azerbaijan (AZE)

### Under-five mortality rate

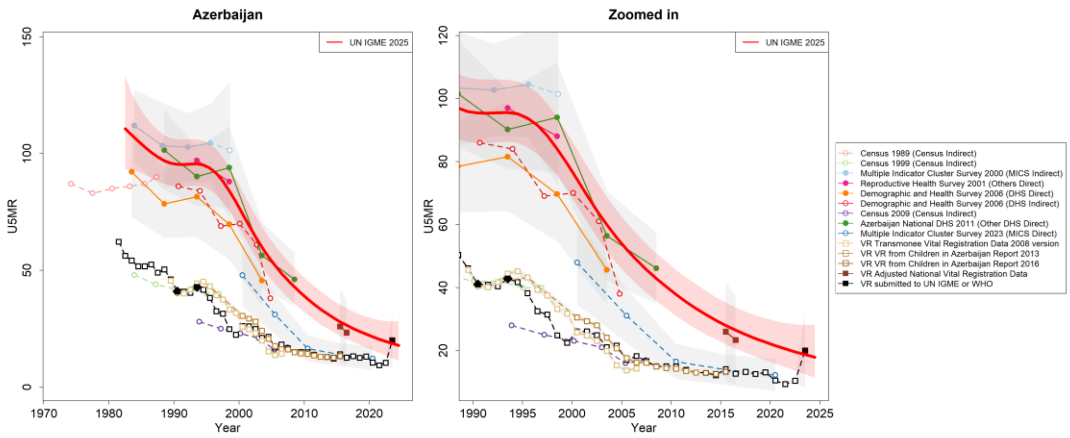


### Infant mortality rate

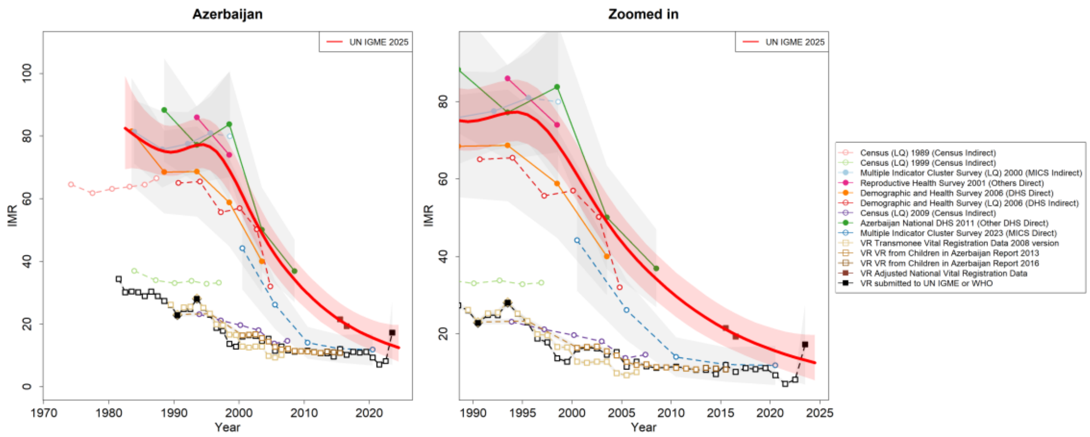


### Neonatal mortality rate

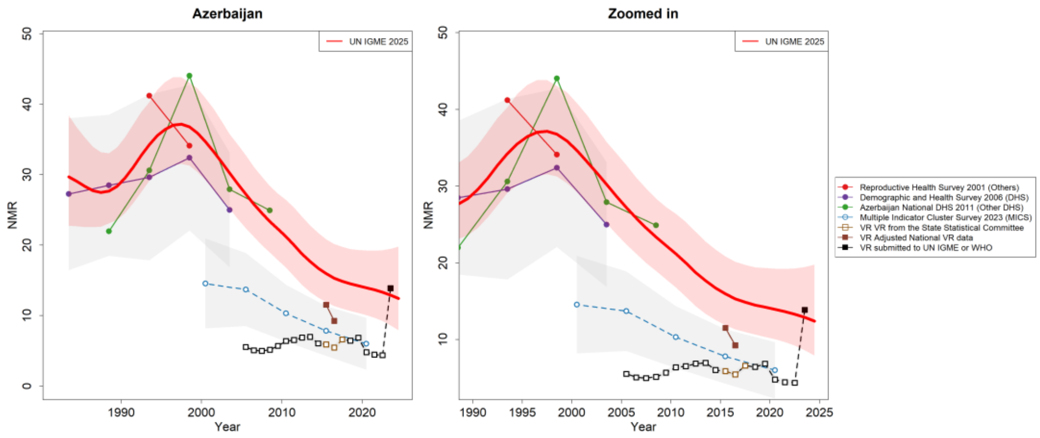

## Bahamas (BHS)

### Under-five mortality rate

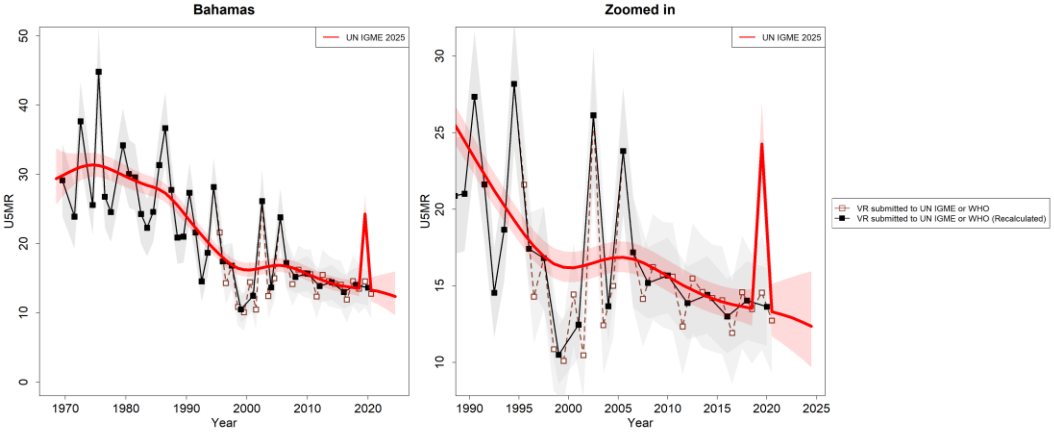


### Infant mortality rate

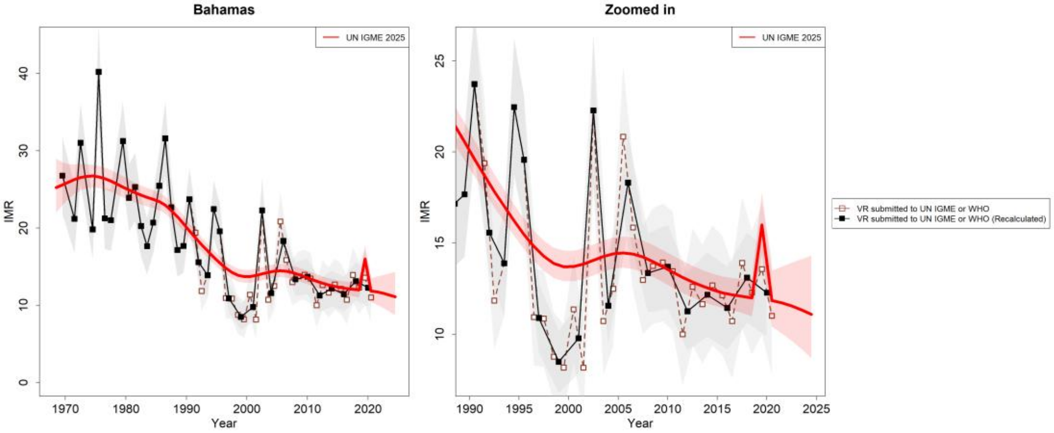


### Neonatal mortality rate

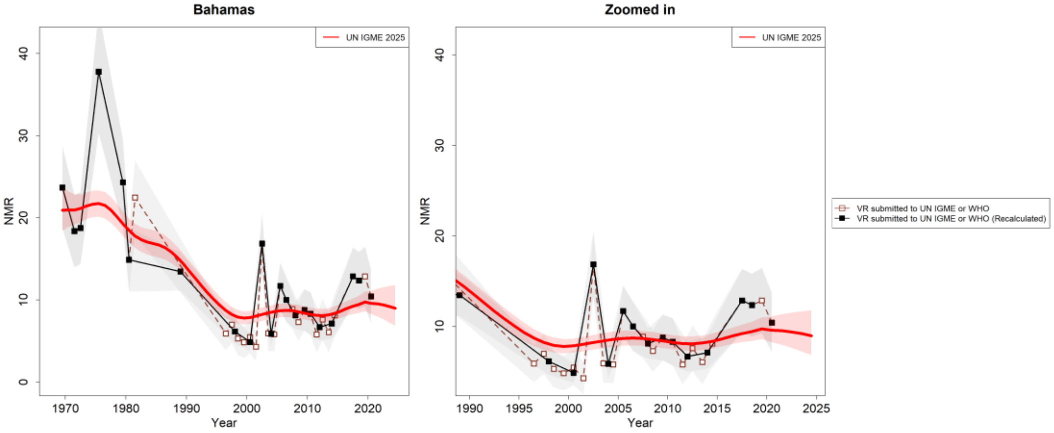

## Bahrain (BHR)

### Under-five mortality rate

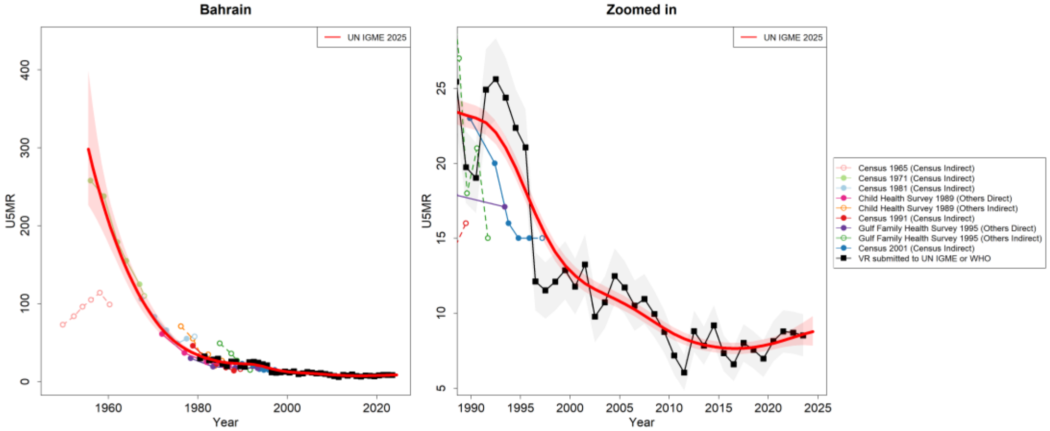


### Infant mortality rate

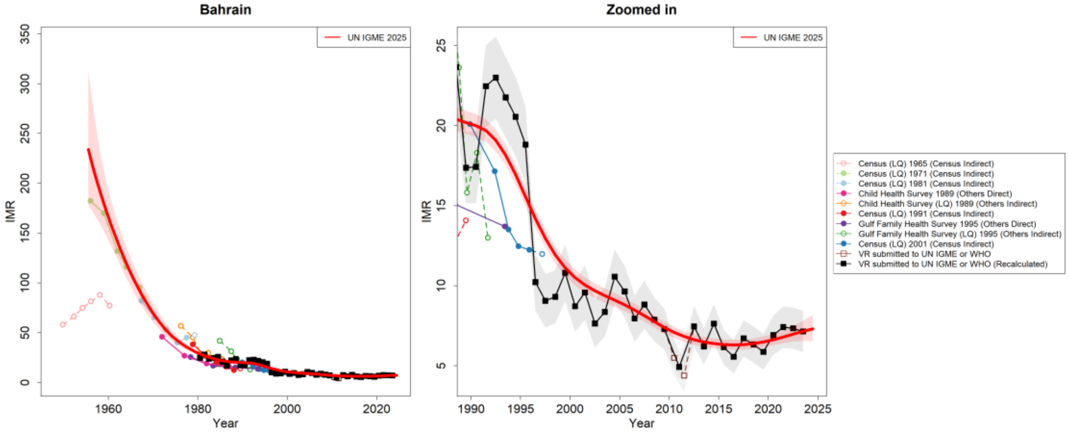


### Neonatal mortality rate

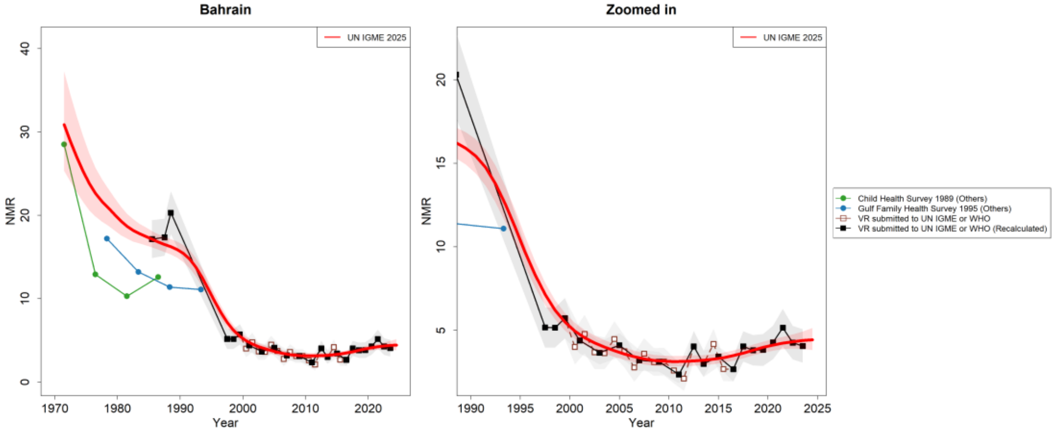

## Bangladesh (BGD)

### Under-five mortality rate

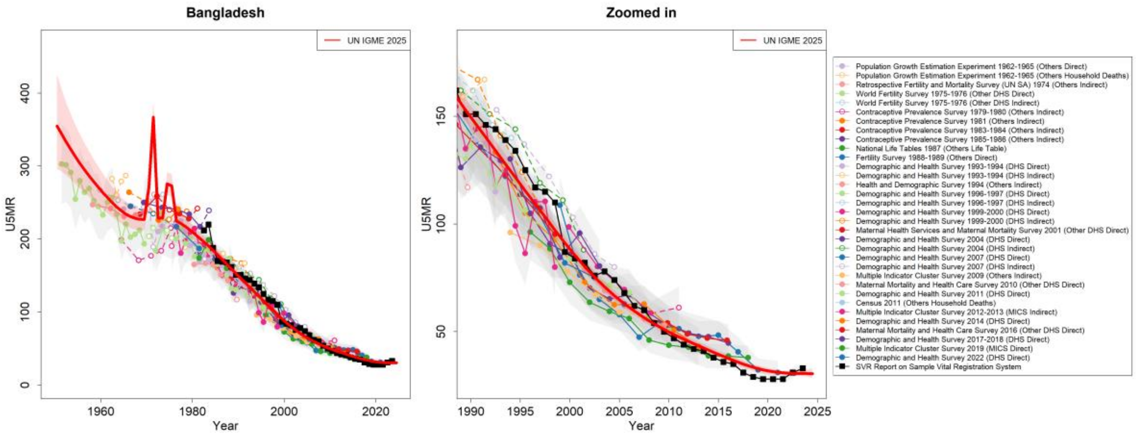


### Infant mortality rate

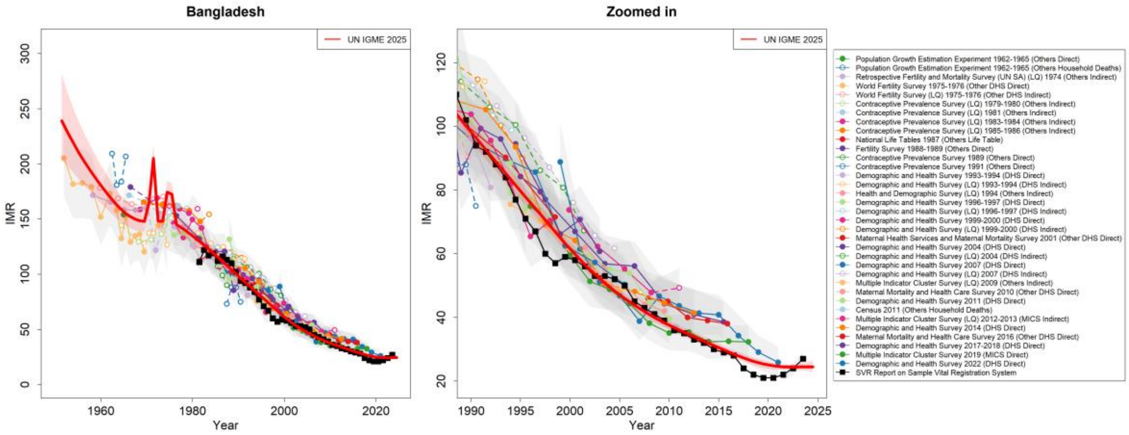


### Neonatal mortality rate

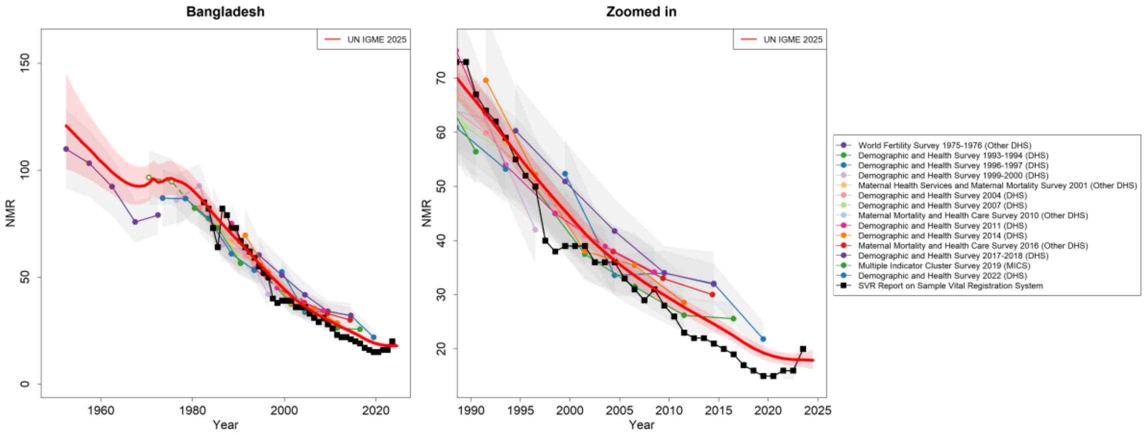

## Barbados (BRB)

### Under-five mortality rate

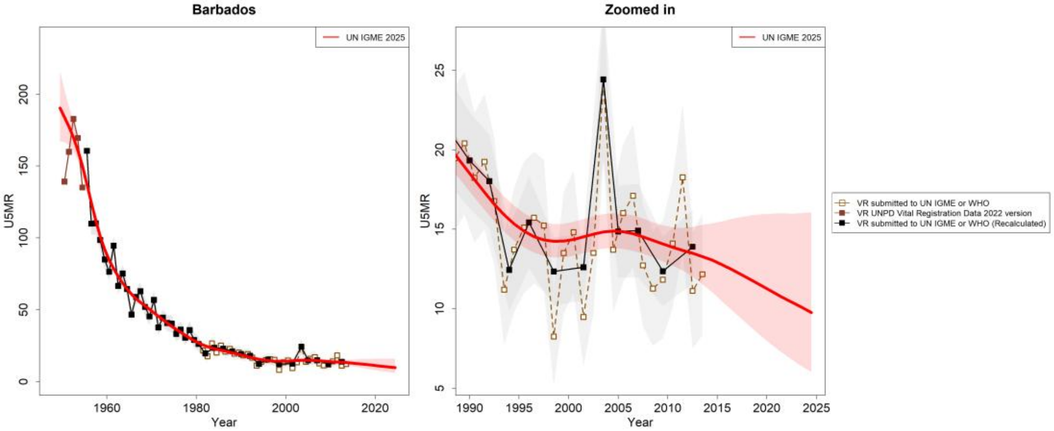


### Infant mortality rate

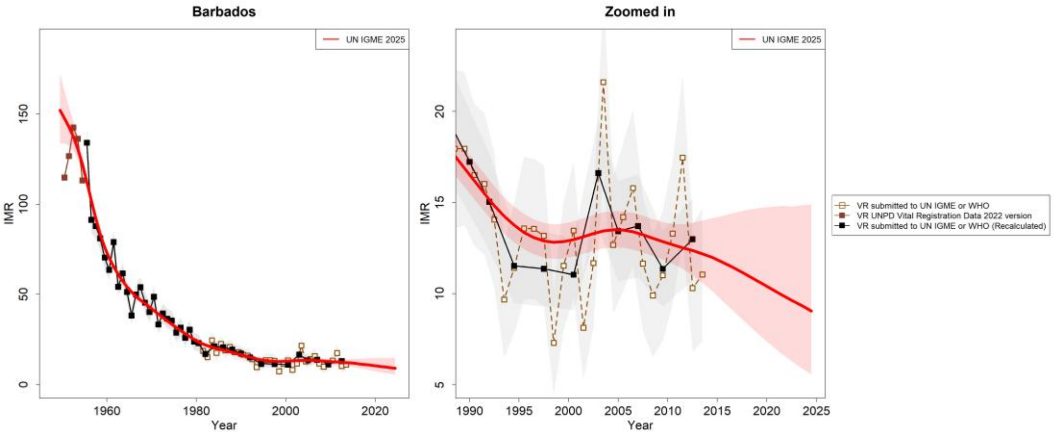


### Neonatal mortality rate

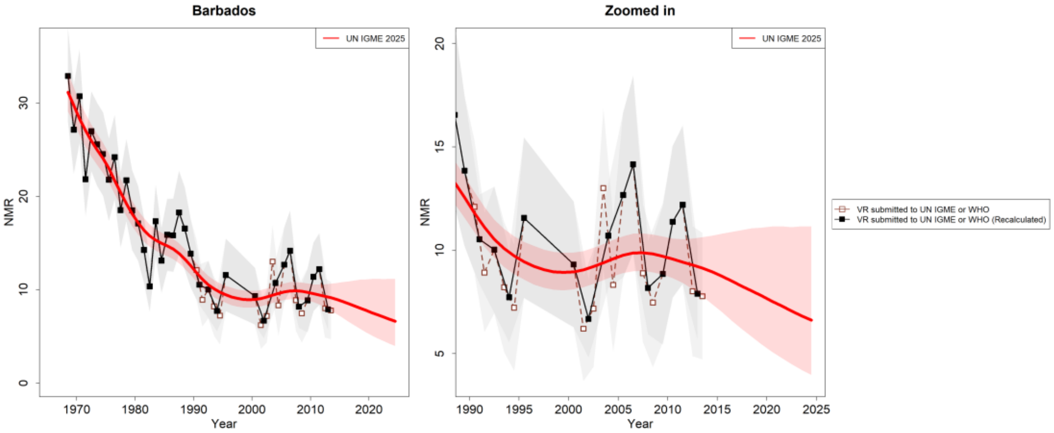

## Belarus (BLR)

### Under-five mortality rate

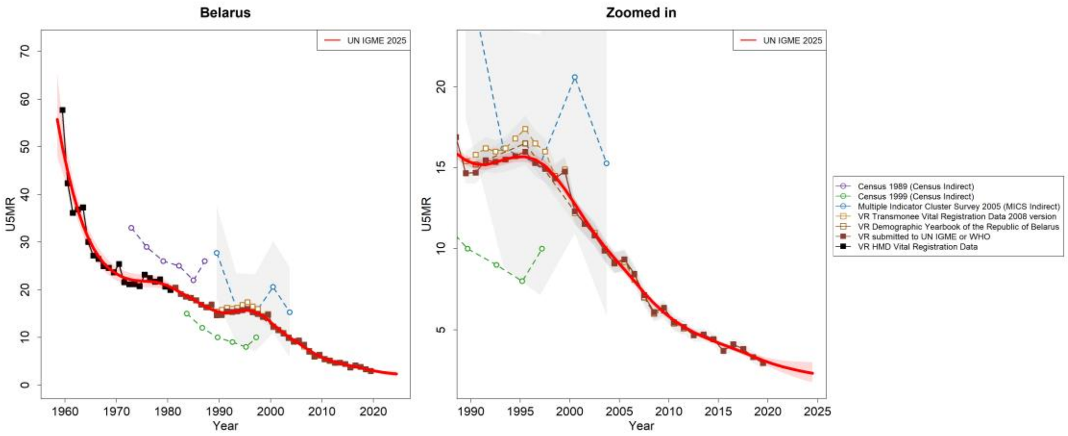


### Infant mortality rate

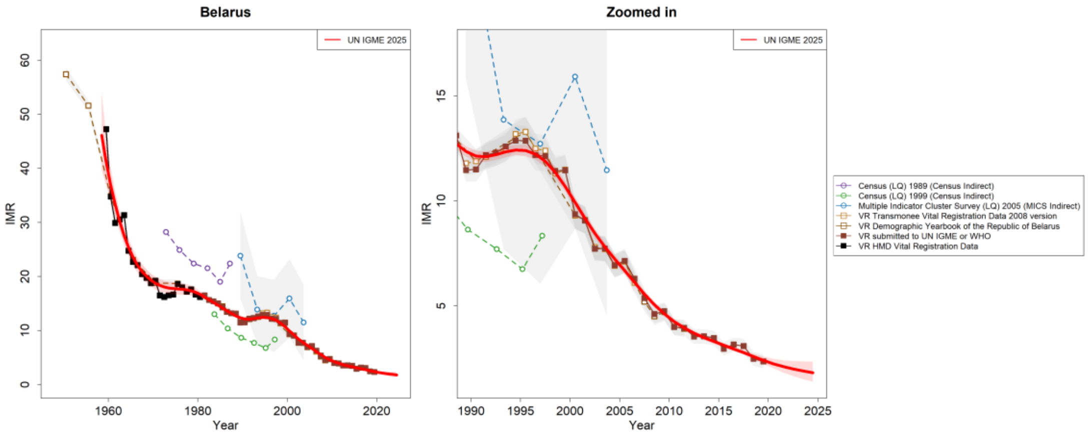


### Neonatal mortality rate

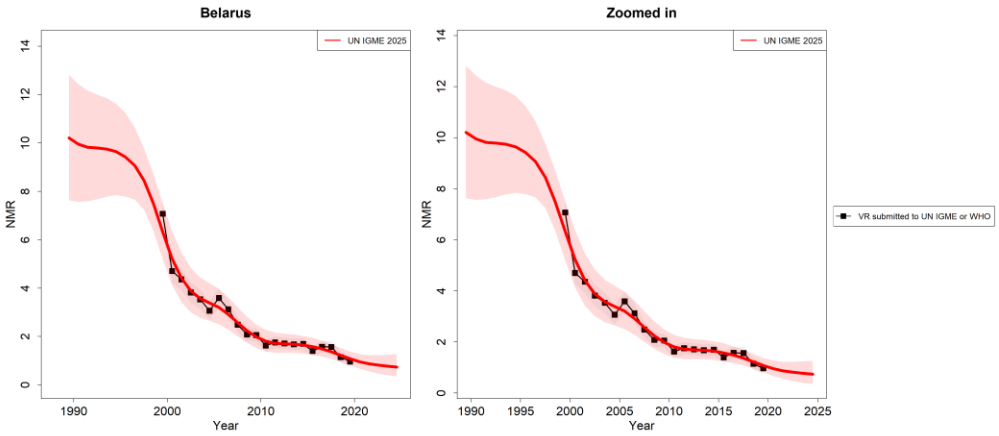

## Belgium (BEL)

### Under-five mortality rate

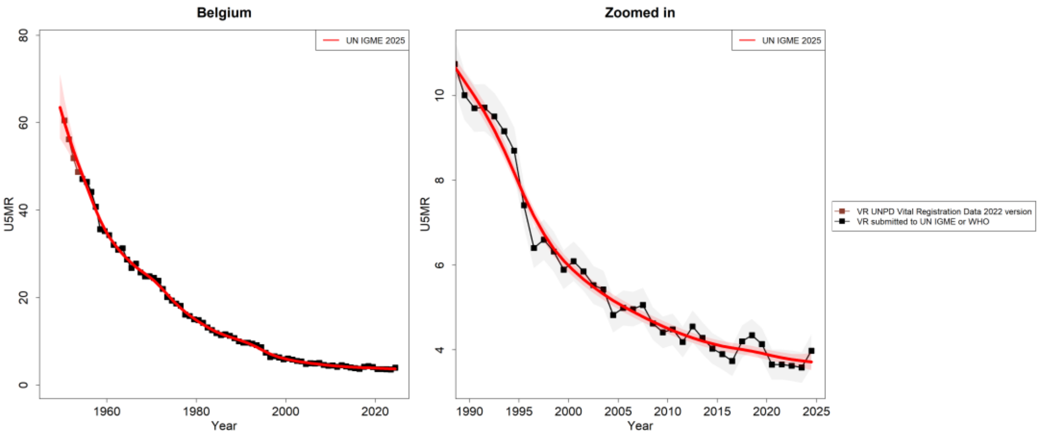


### Infant mortality rate

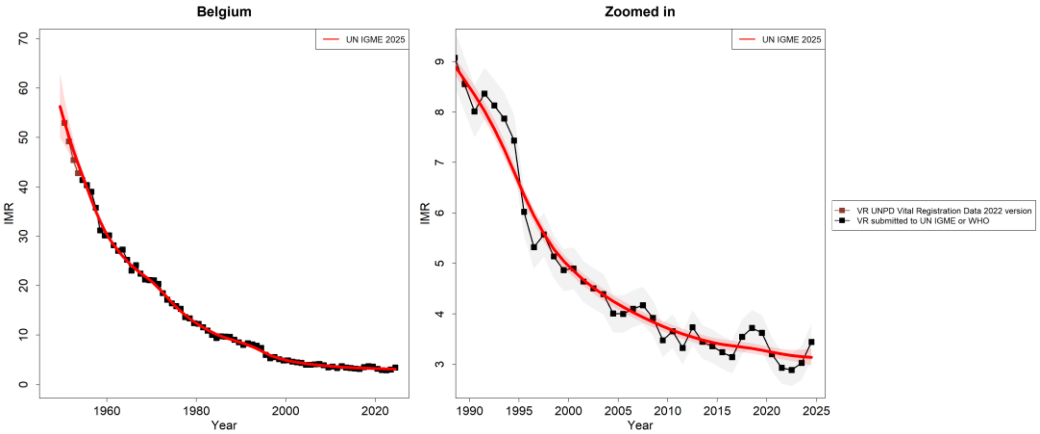


### Neonatal mortality rate

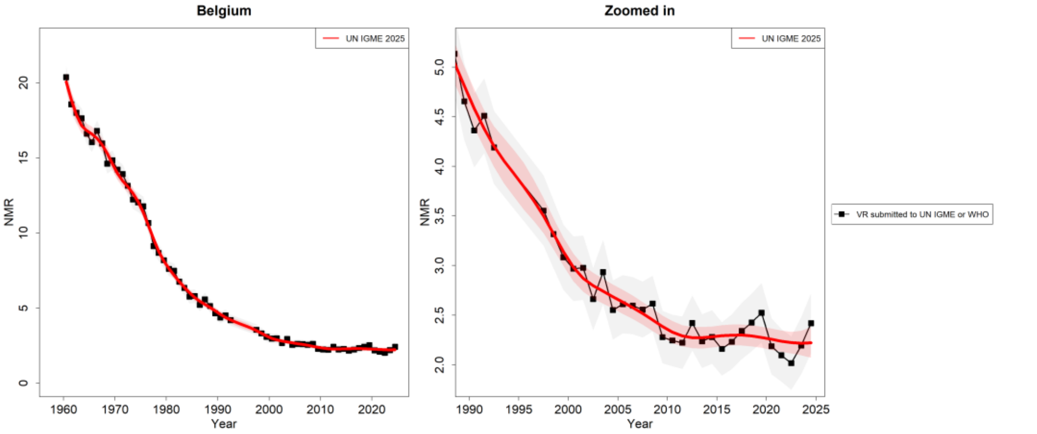

## Belize (BLZ)

### Under-five mortality rate

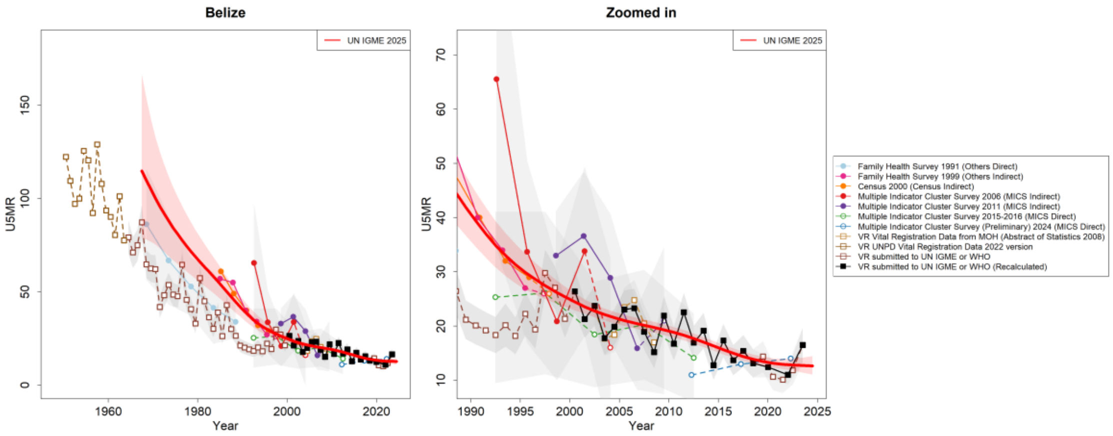


### Infant mortality rate

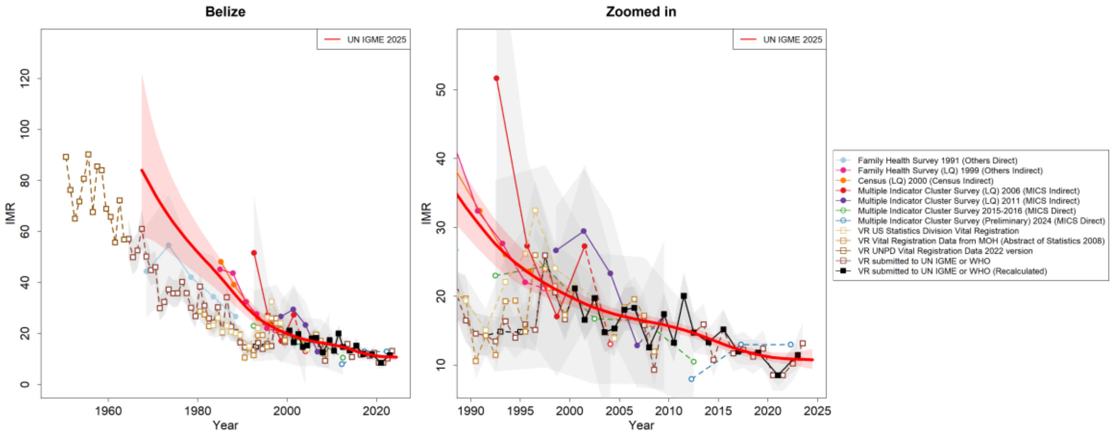


### Neonatal mortality rate

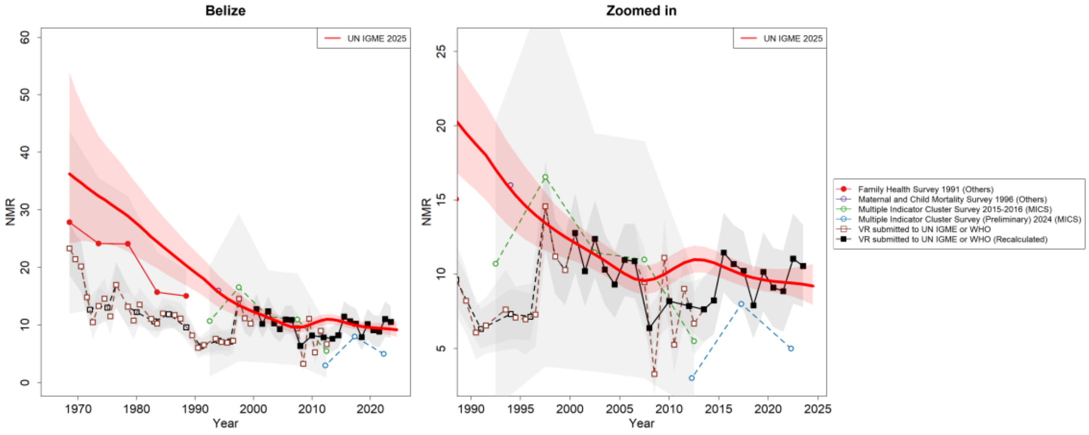

## Benin (BEN)

### Under-five mortality rate

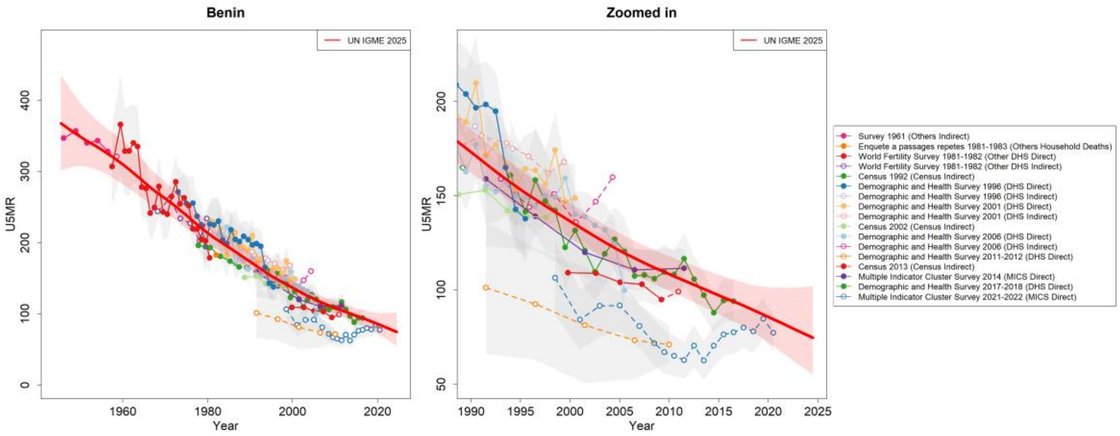


### Infant mortality rate

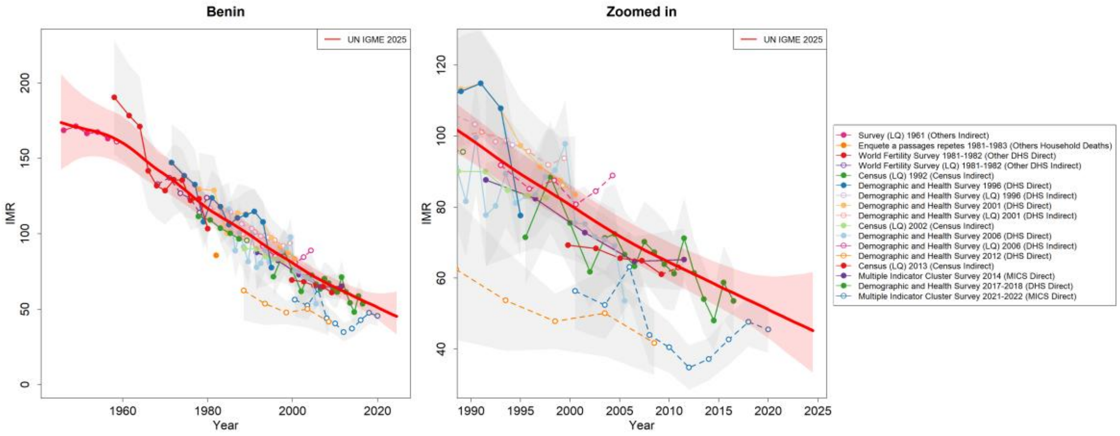


### Neonatal mortality rate

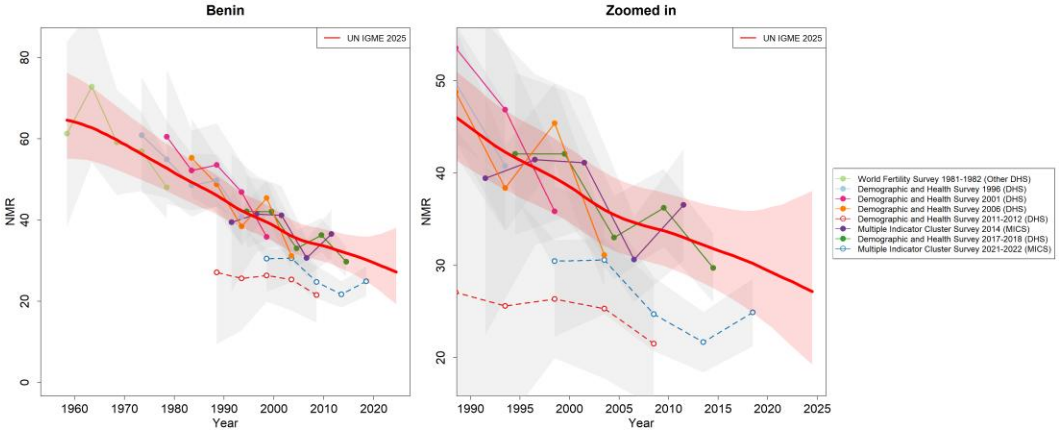

## Bhutan (BTN)

### Under-five mortality rate

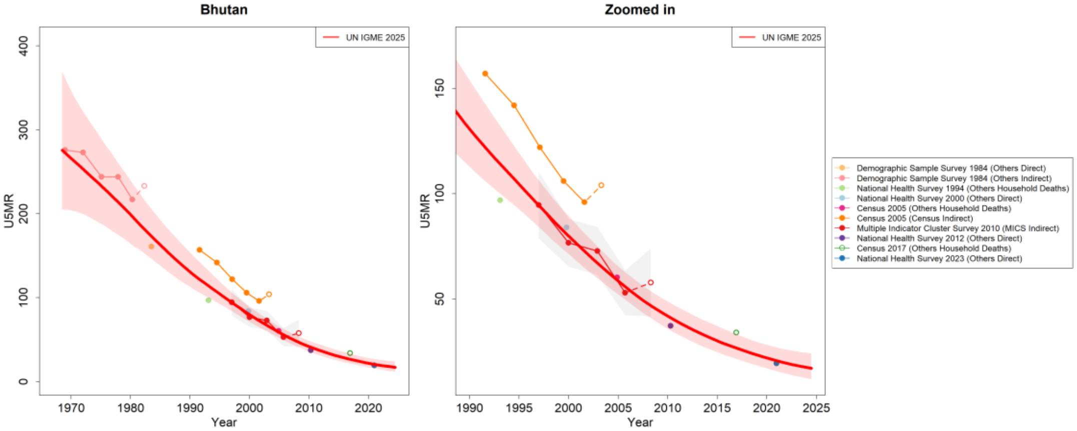


### Infant mortality rate

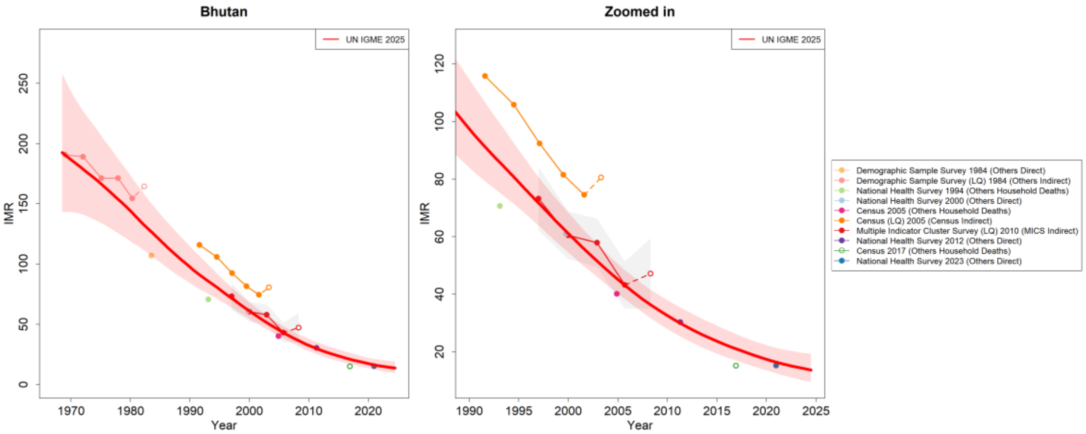


### Neonatal mortality rate

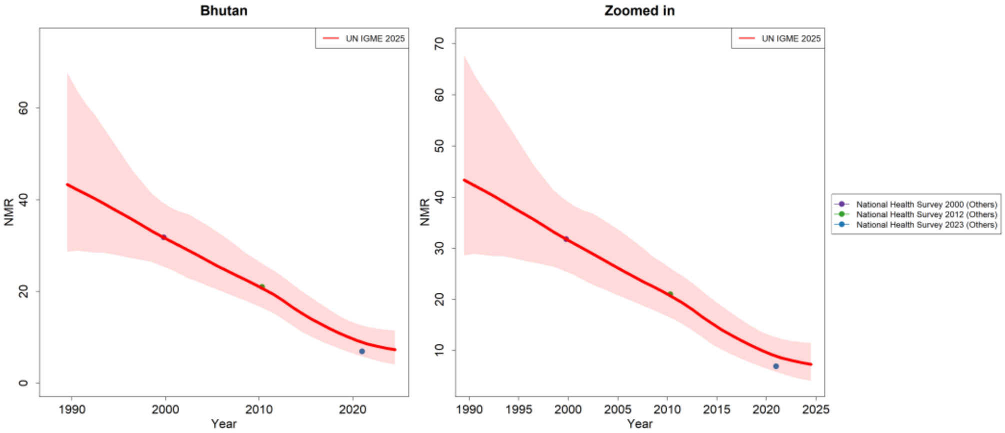

## Bolivia (Plurinational State of) (BOL)

### Under-five mortality rate

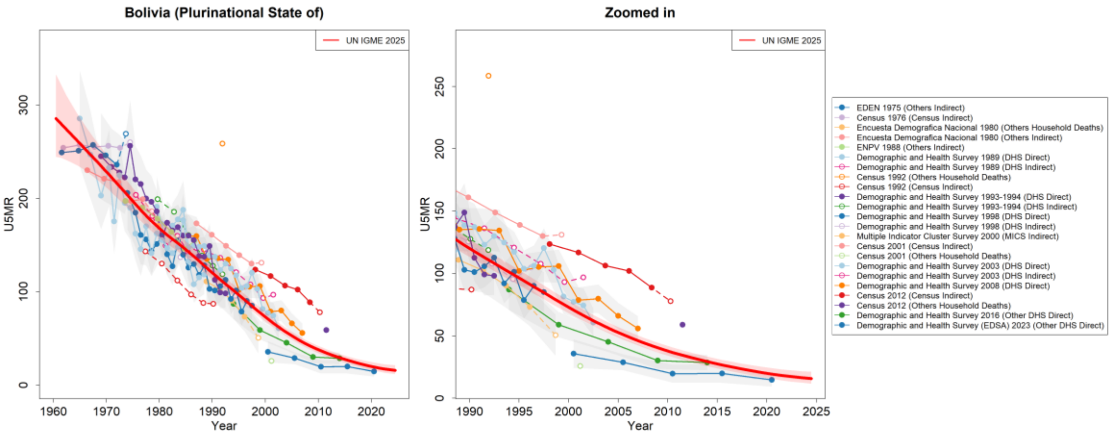


### Infant mortality rate

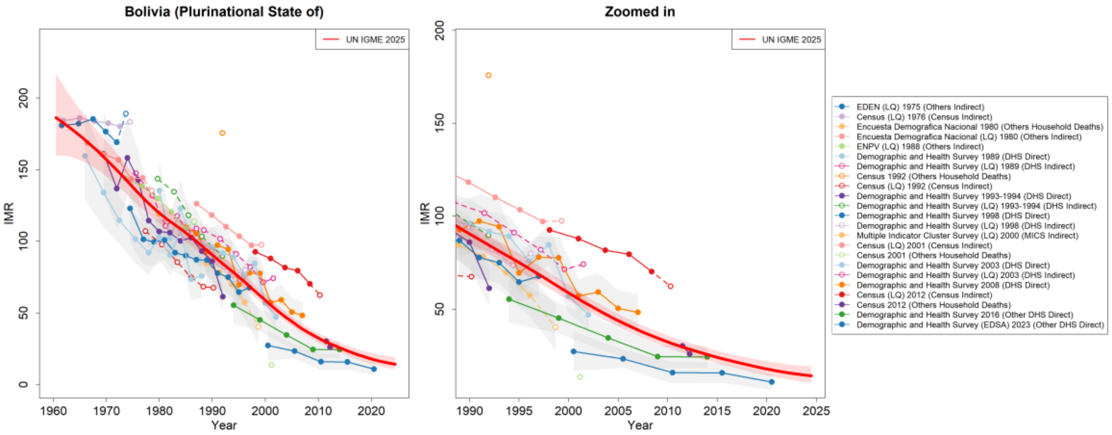


### Neonatal mortality rate

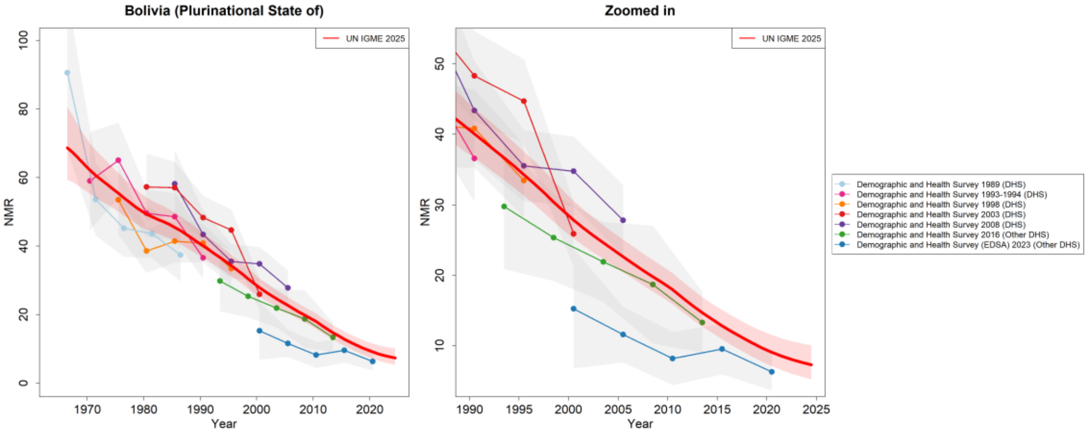

## Bosnia and Herzegovina (BIH)

### Under-five mortality rate

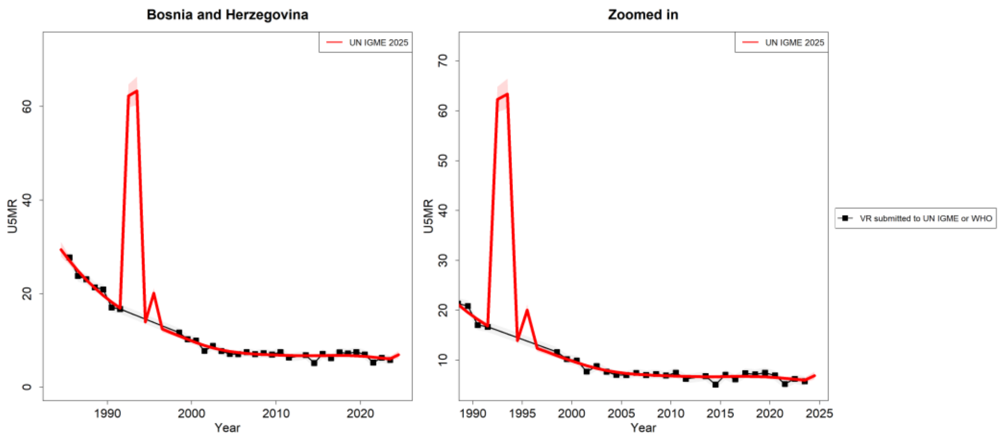


### Infant mortality rate

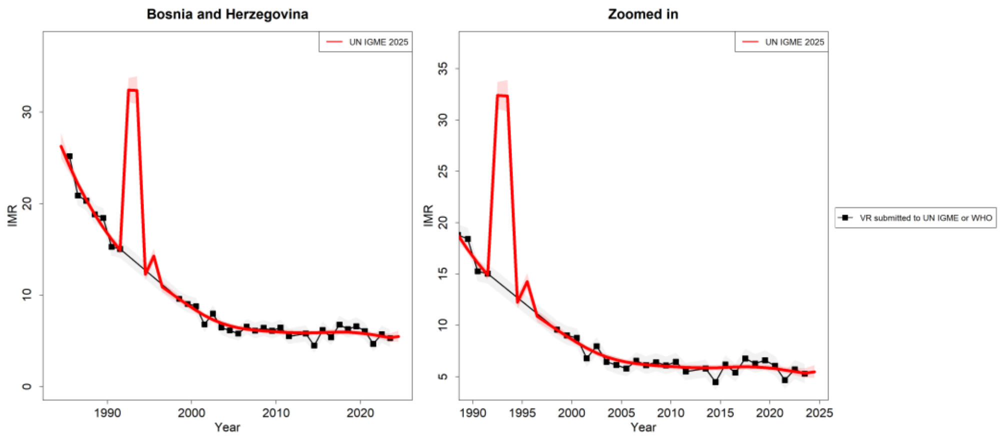


### Neonatal mortality rate

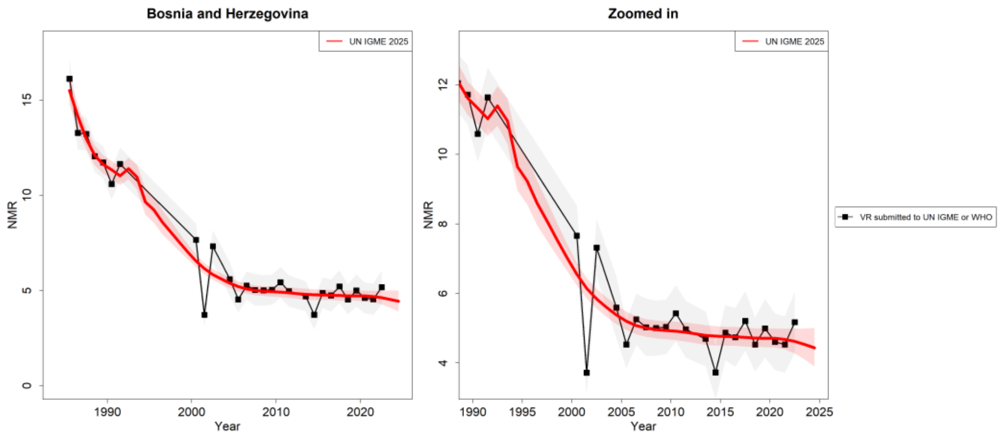

## Botswana (BWA)

### Under-five mortality rate

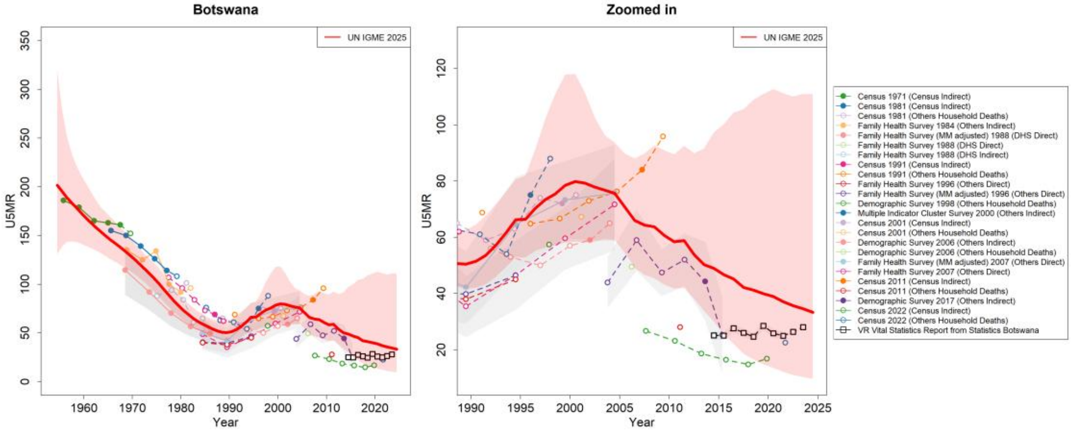


### Infant mortality rate

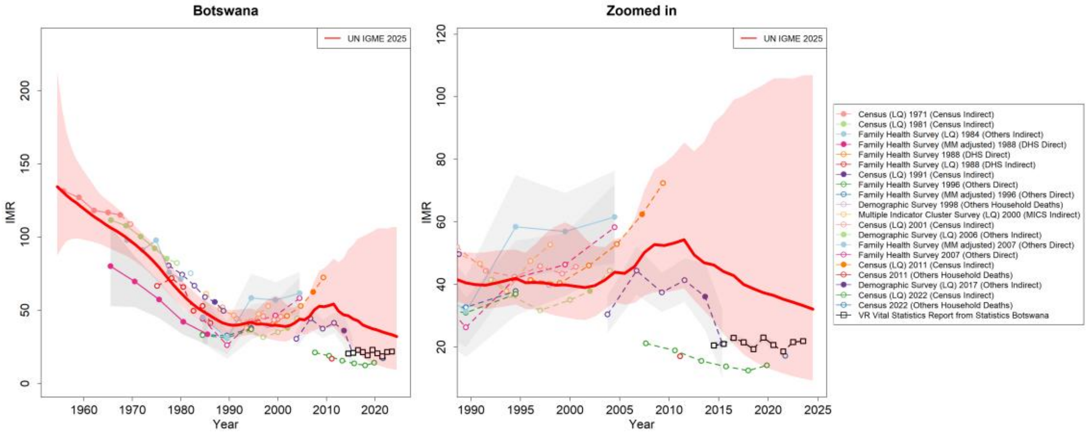


### Neonatal mortality rate

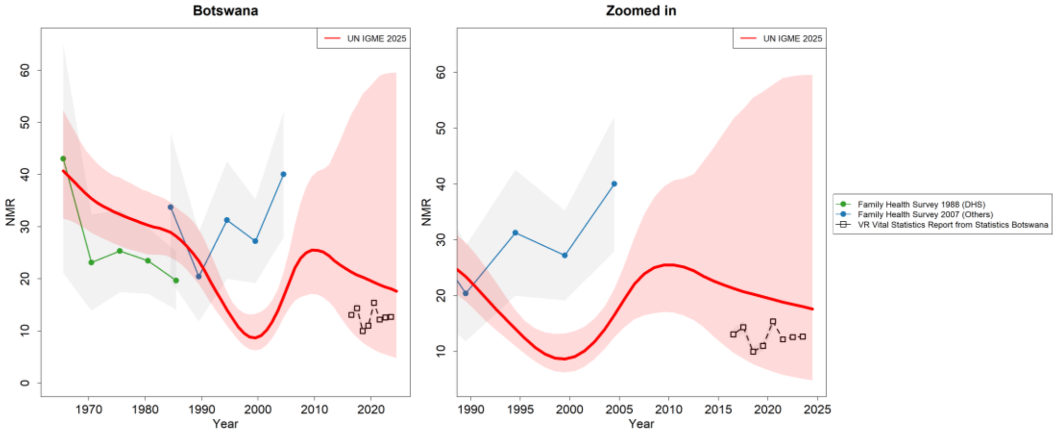

## Brazil (BRA)

### Under-five mortality rate

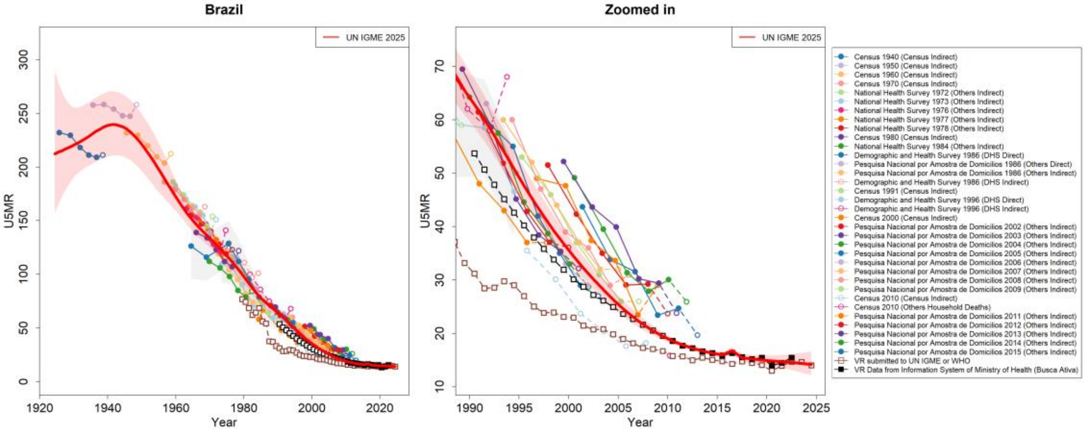


### Infant mortality rate

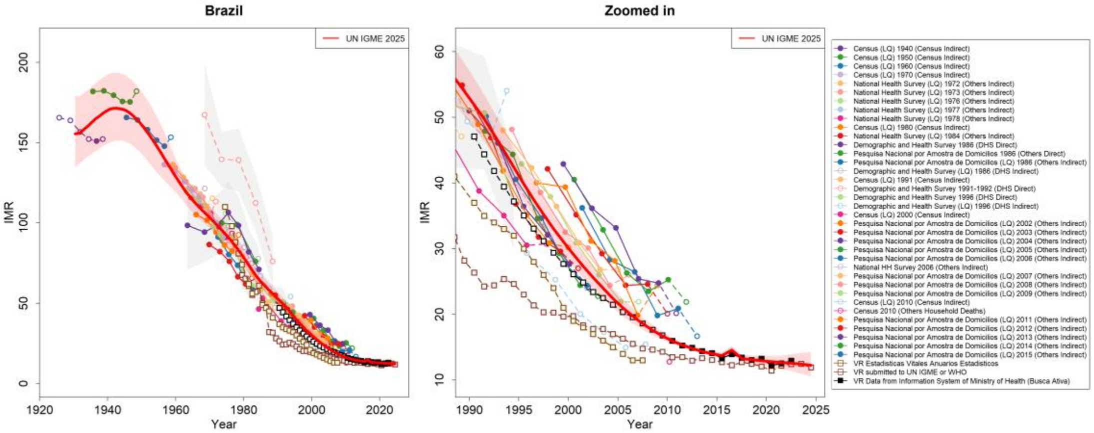


### Neonatal mortality rate

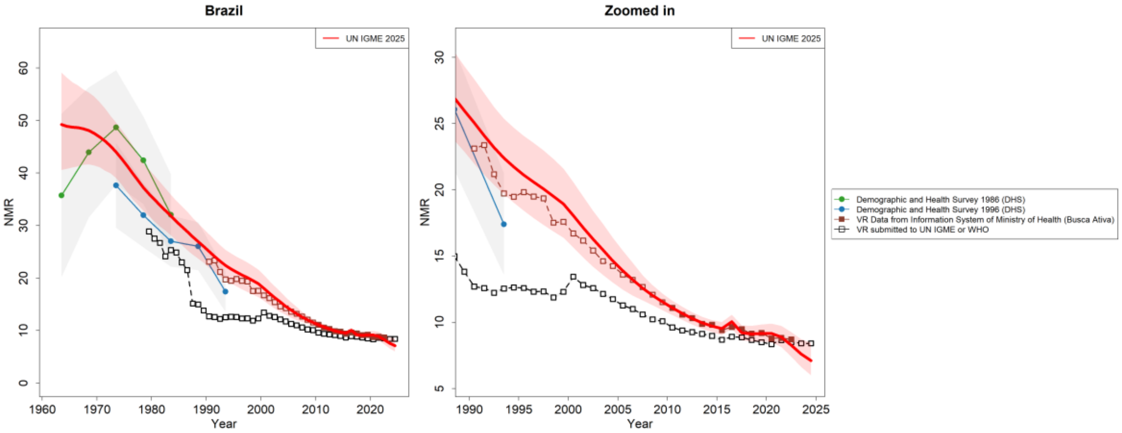

## British Virgin Islands (VGB)

### Under-five mortality rate

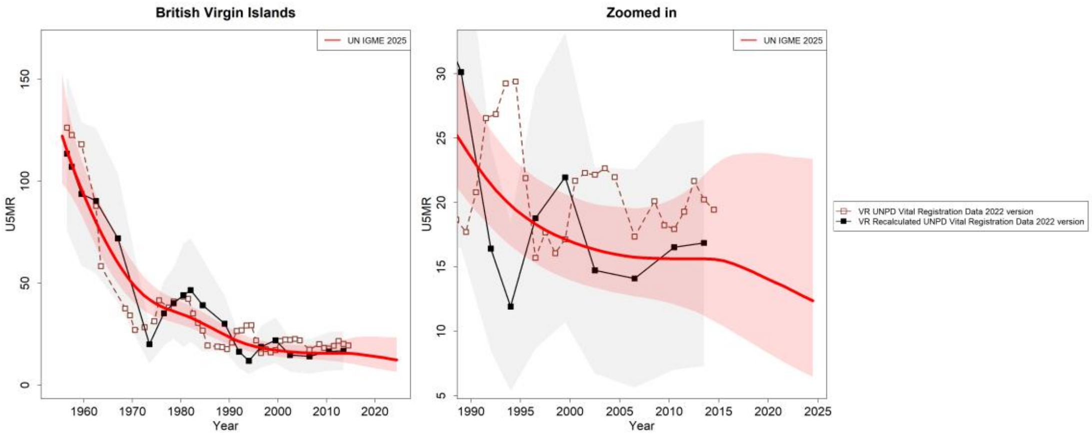


### Infant mortality rate

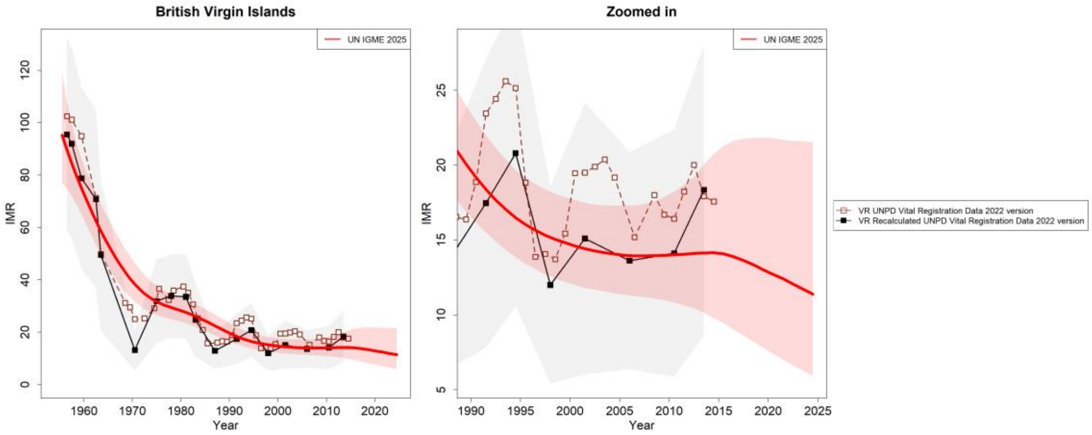


### Neonatal mortality rate

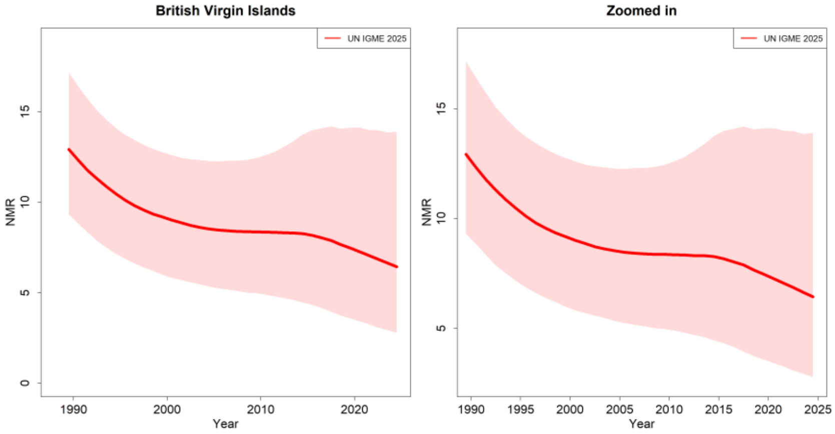

## Brunei Darussalam (BRN)

### Under-five mortality rate

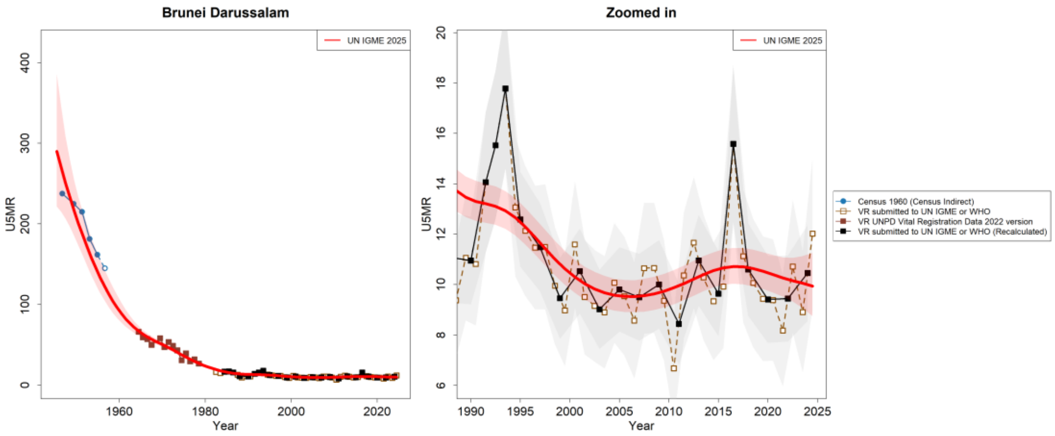


### Infant mortality rate

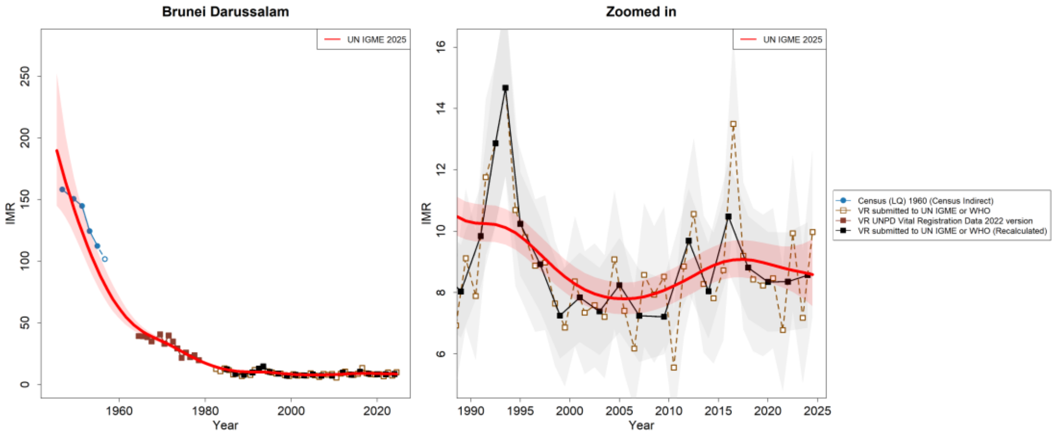


### Neonatal mortality rate

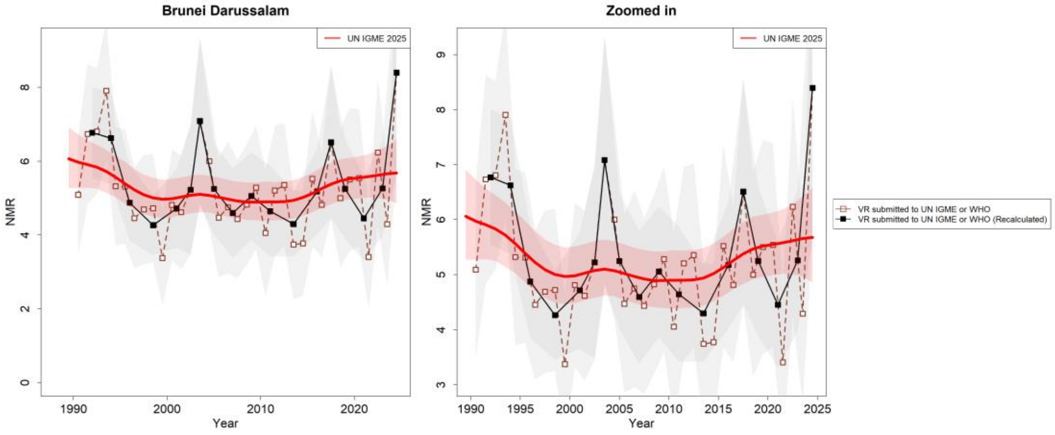

## Bulgaria (BGR)

### Under-five mortality rate

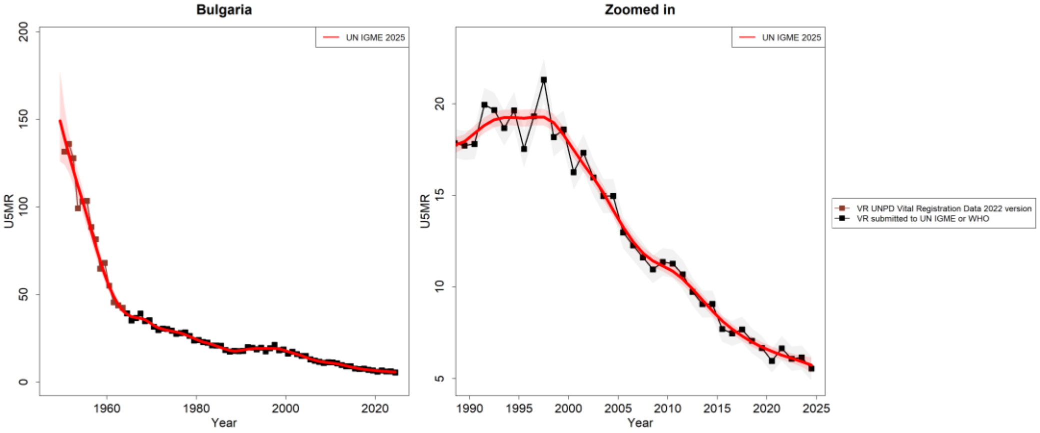


### Infant mortality rate

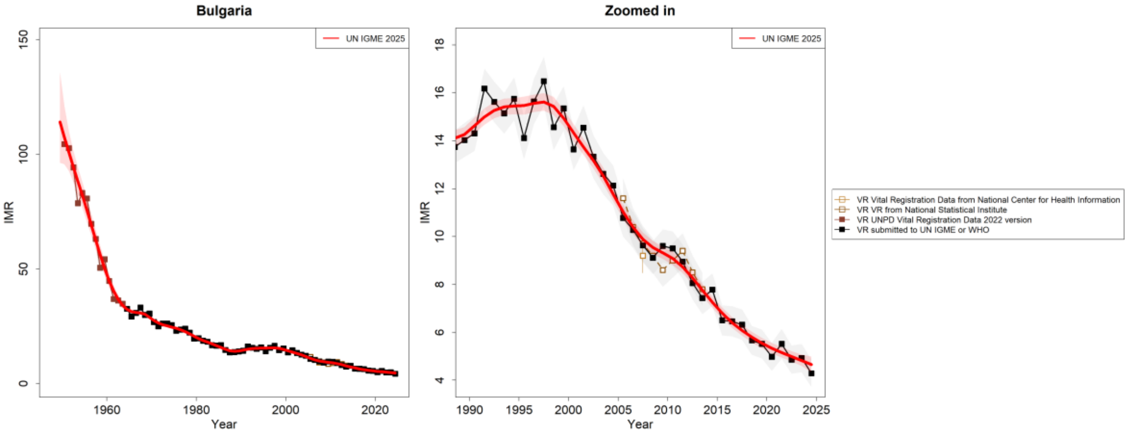


### Neonatal mortality rate

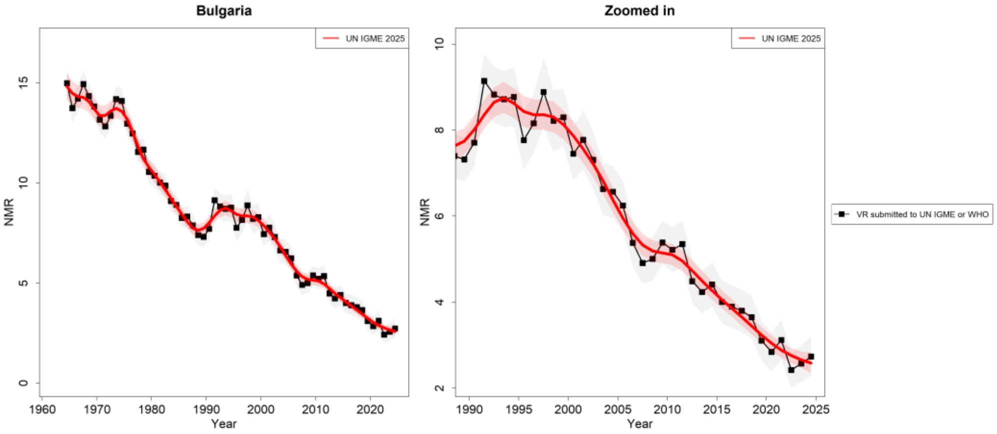

## Burkina Faso (BFA)

### Under-five mortality rate

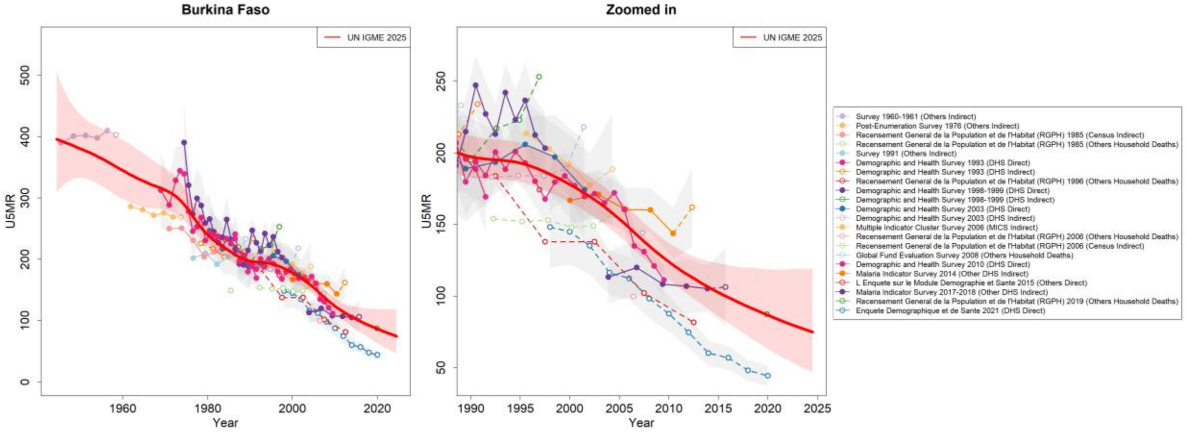


### Infant mortality rate

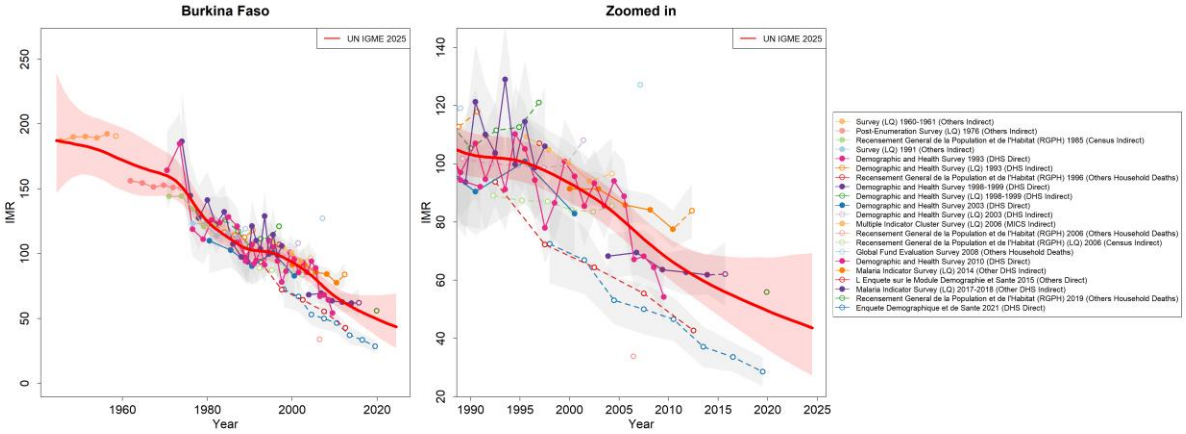


### Neonatal mortality rate

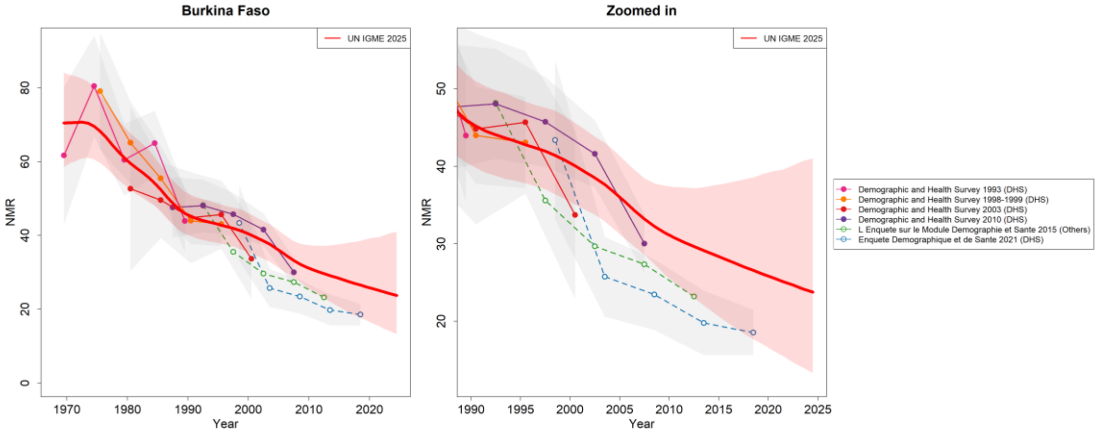

## Burundi (BDI)

### Under-five mortality rate

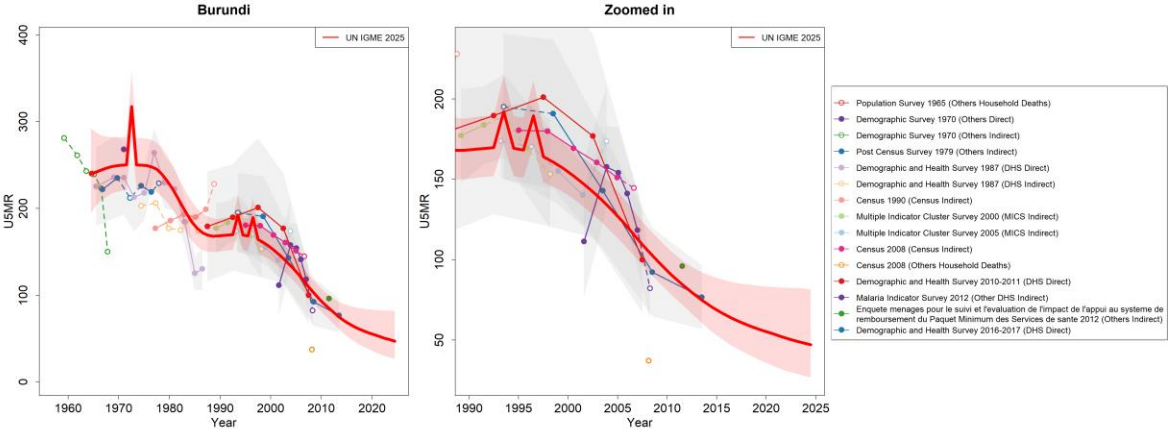


### Infant mortality rate

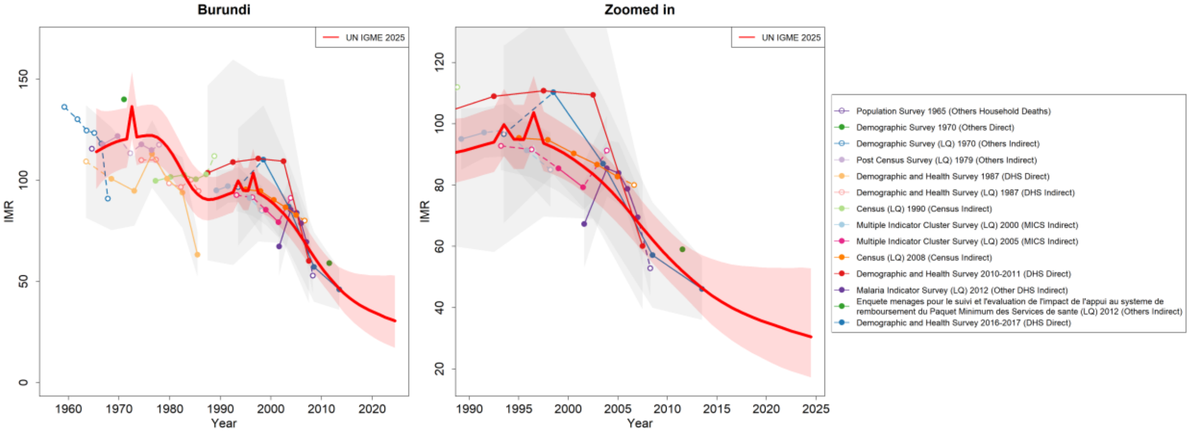


### Neonatal mortality rate

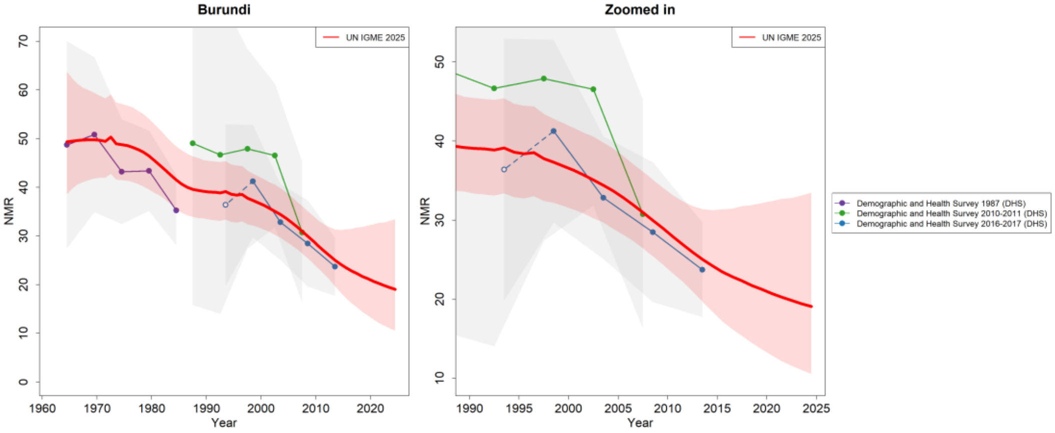

## Cabo Verde (CPV)

### Under-five mortality rate

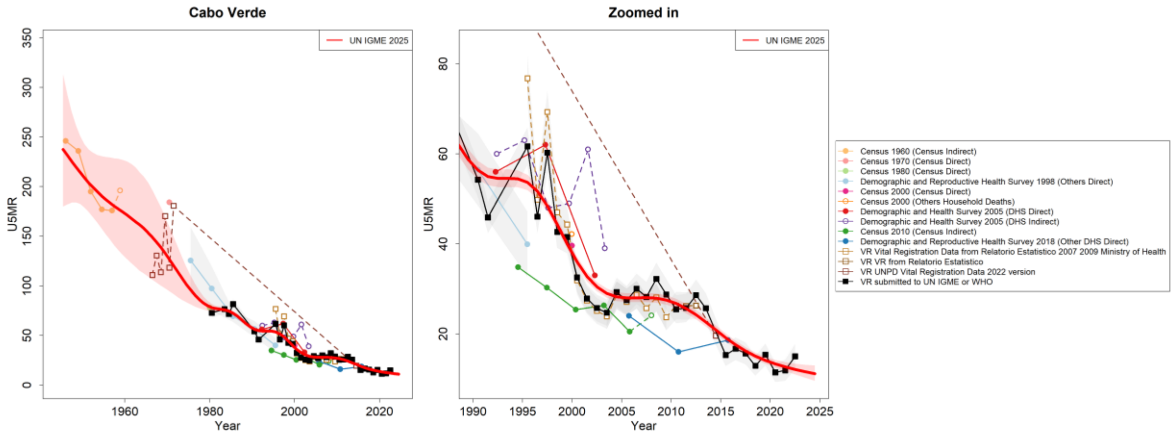


### Infant mortality rate

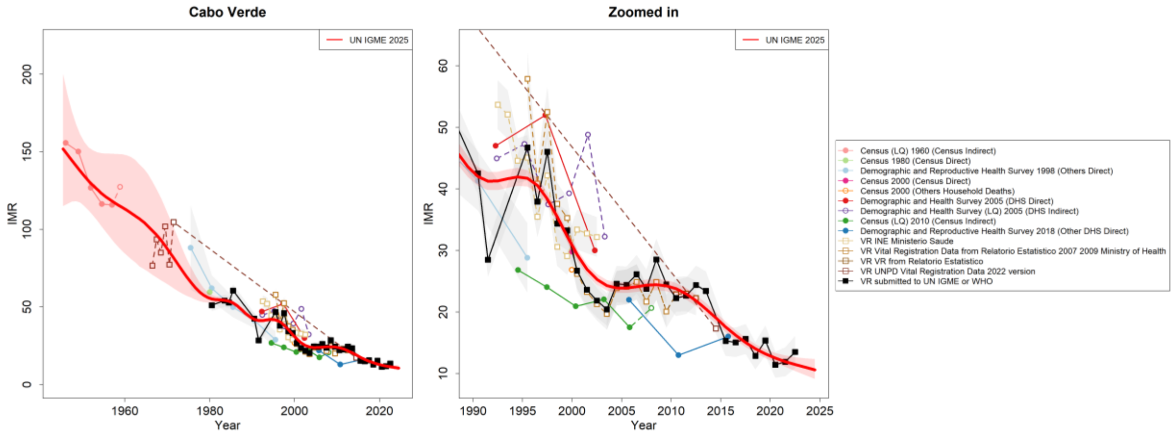


### Neonatal mortality rate

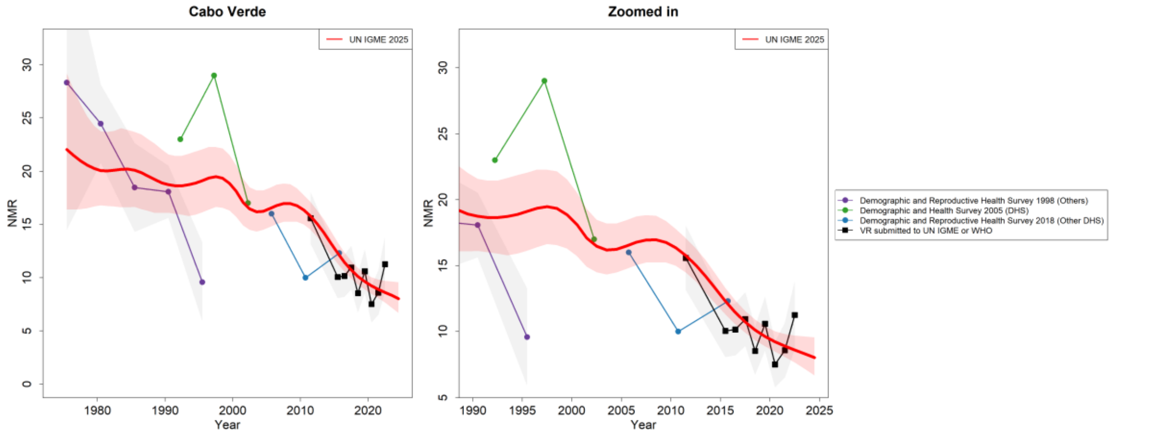

## Cambodia (KHM)

### Under-five mortality rate

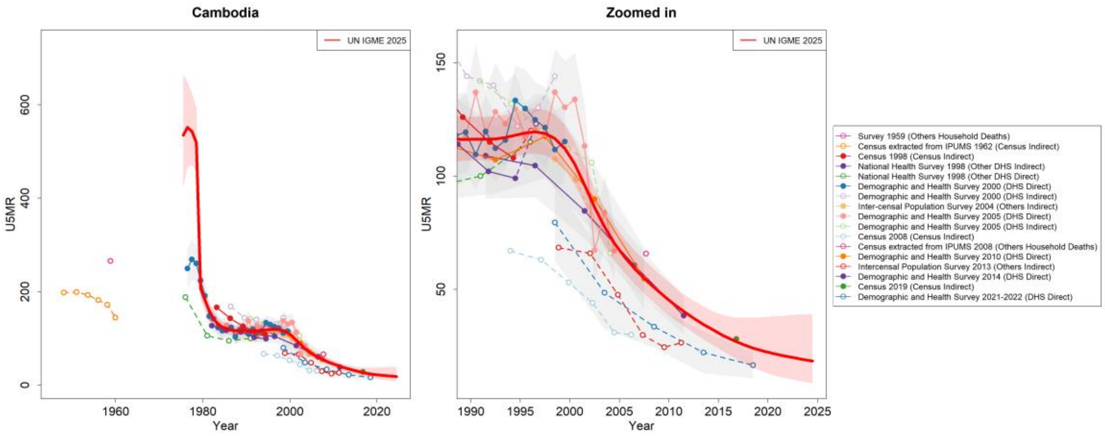


### Infant mortality rate

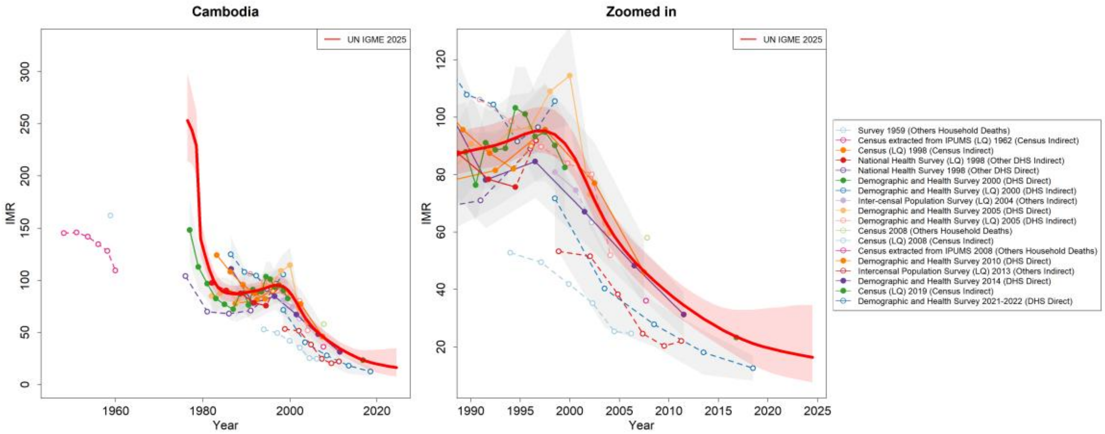


### Neonatal mortality rate

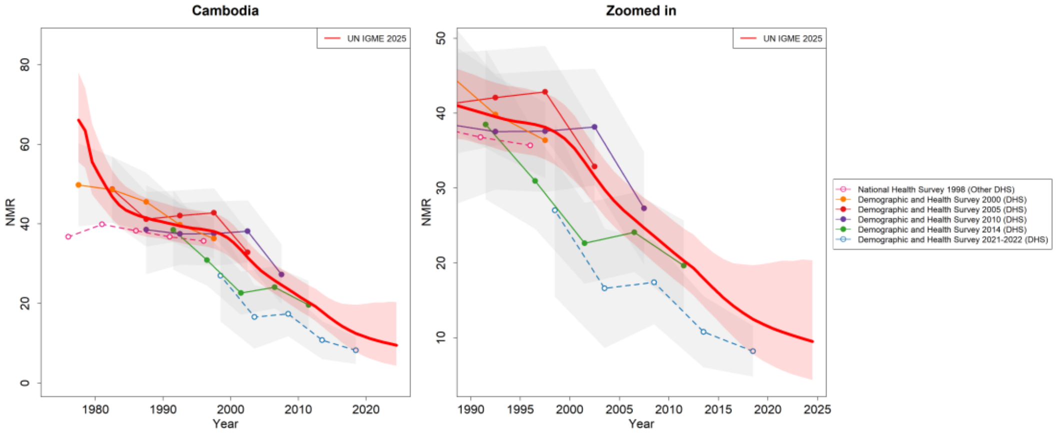

## Cameroon (CMR)

### Under-five mortality rate

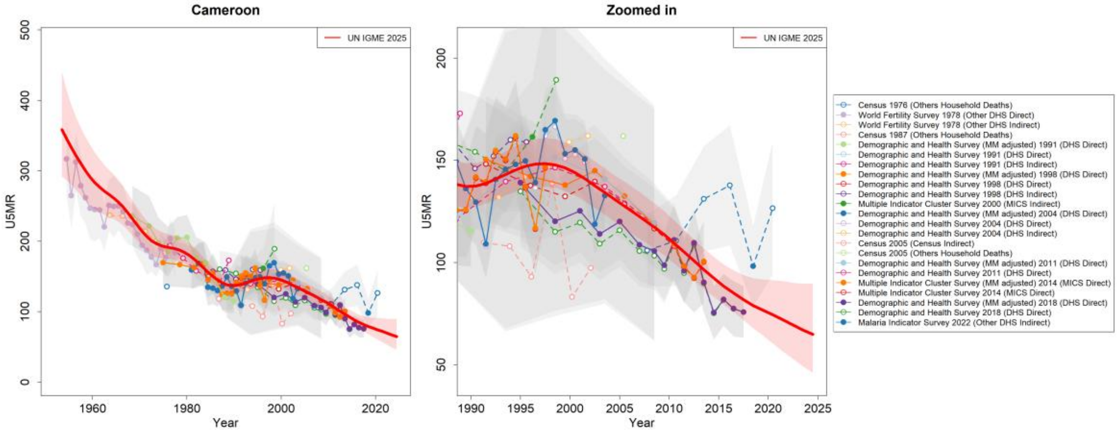


### Infant mortality rate

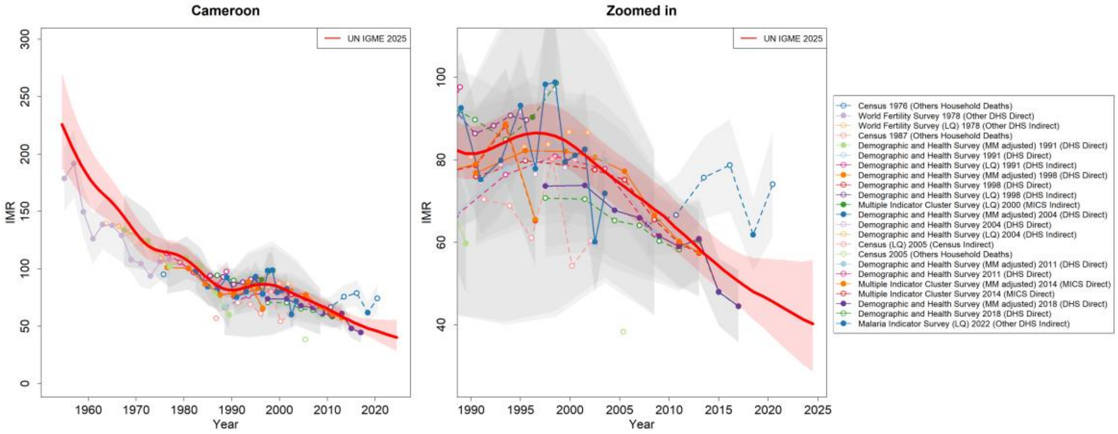


### Neonatal mortality rate

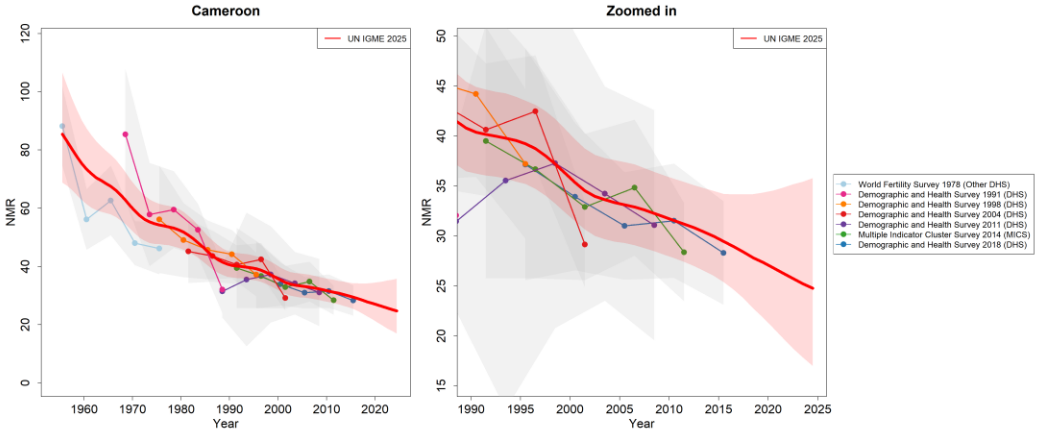

## Canada (CAN)

### Under-five mortality rate

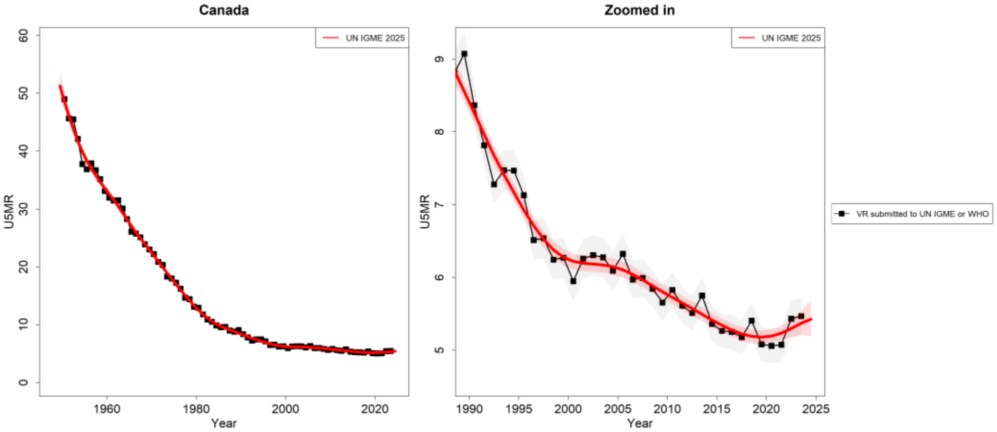


### Infant mortality rate

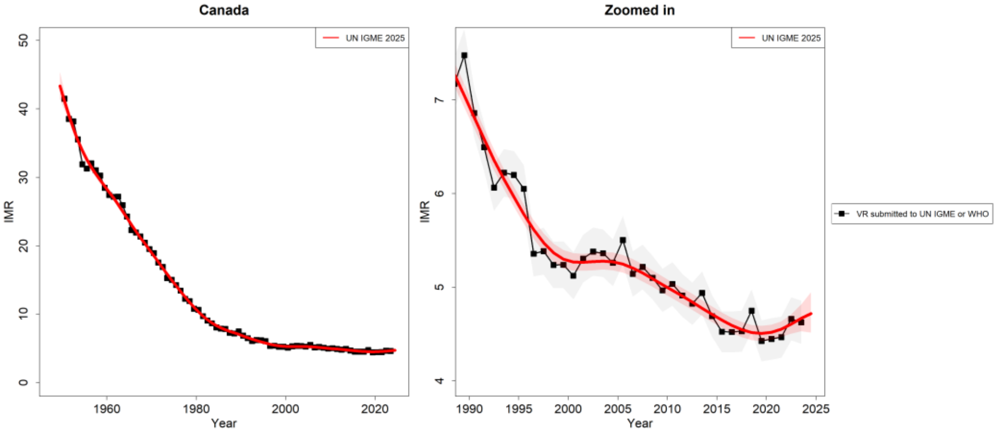


### Neonatal mortality rate

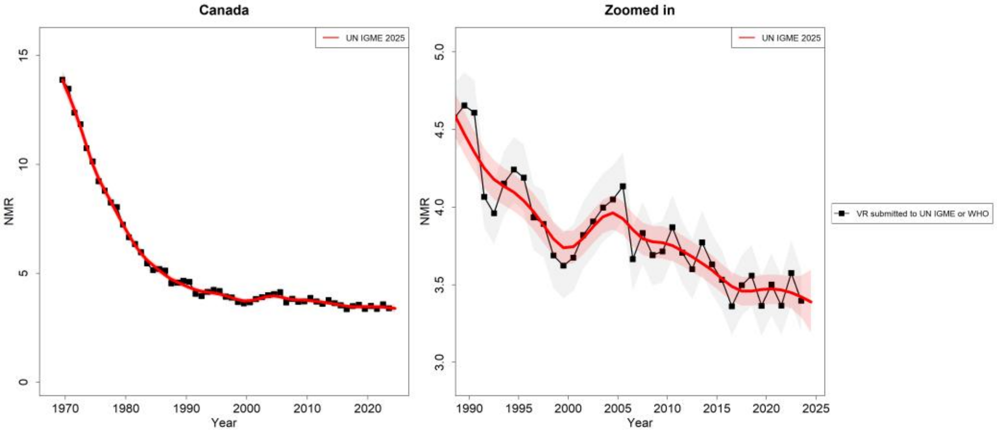

## Central African Republic (CAF)

### Under-five mortality rate

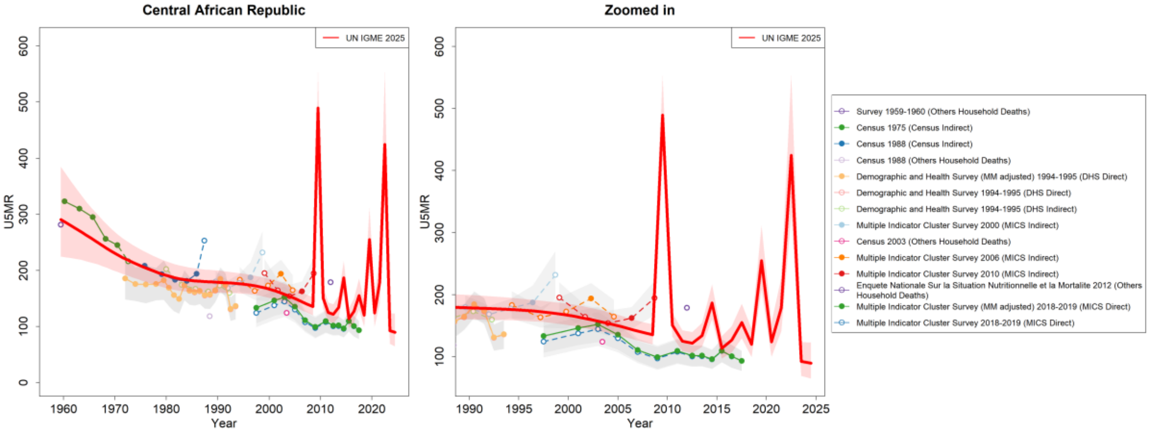


### Infant mortality rate

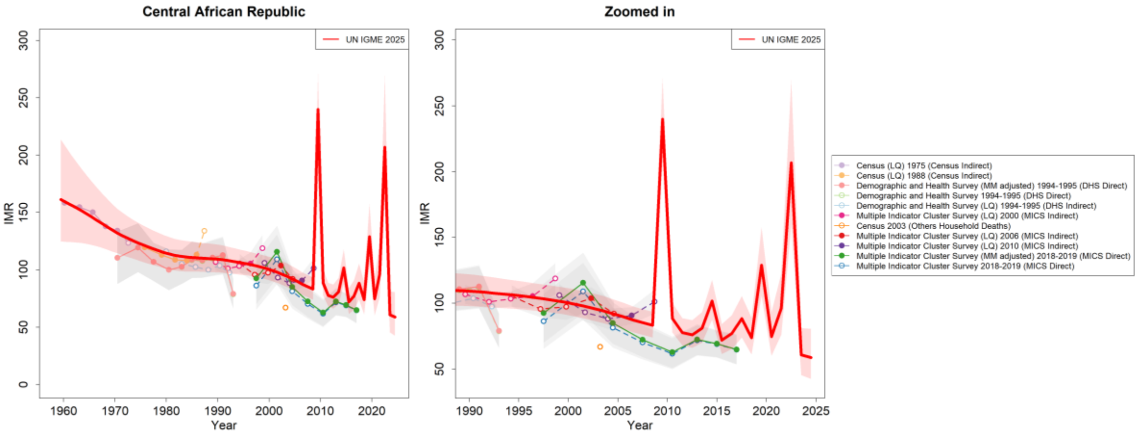


### Neonatal mortality rate

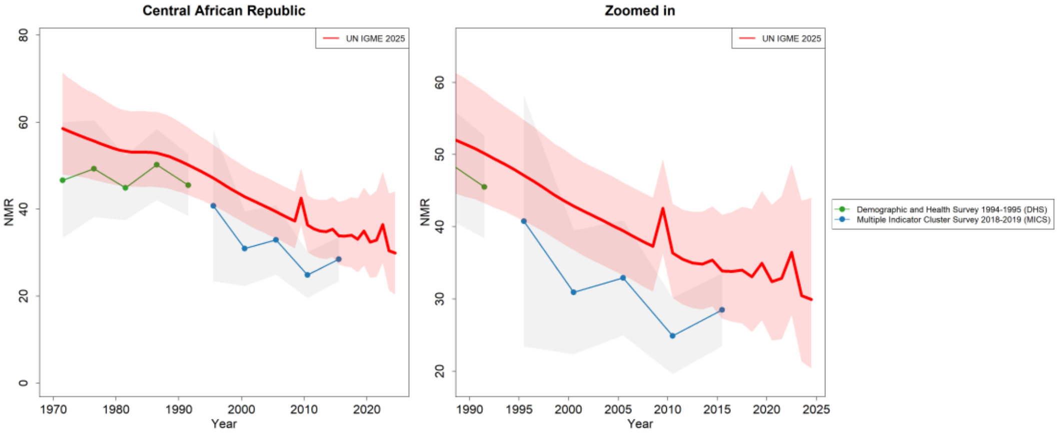

## Chad (TCD)

### Under-five mortality rate

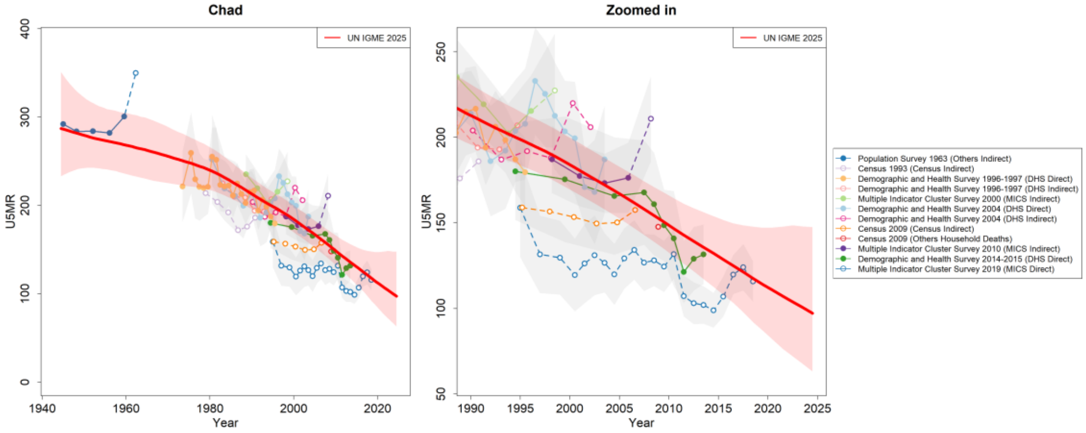


### Infant mortality rate

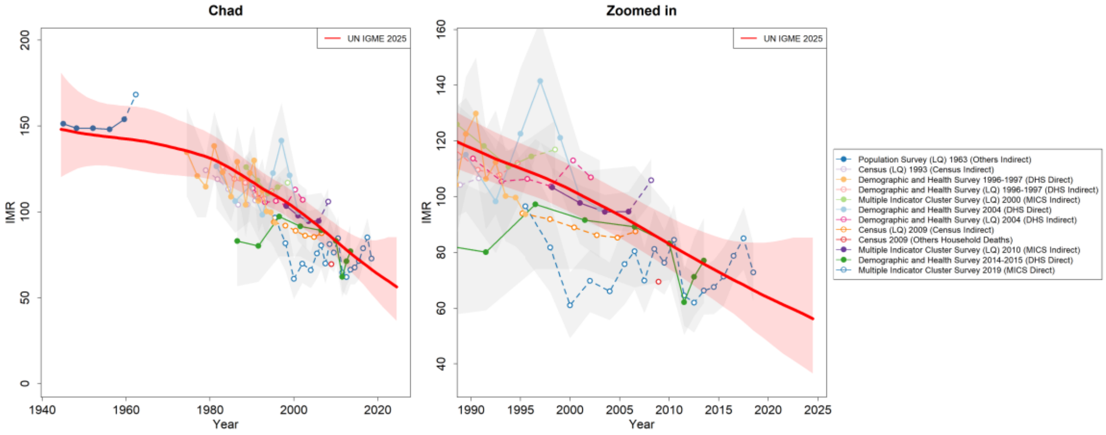


### Neonatal mortality rate

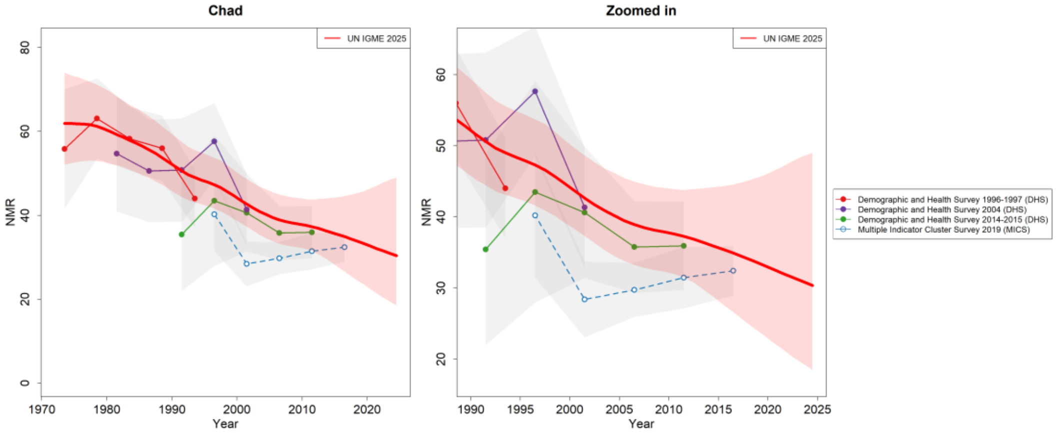

## Chile (CHL)

### Under-five mortality rate

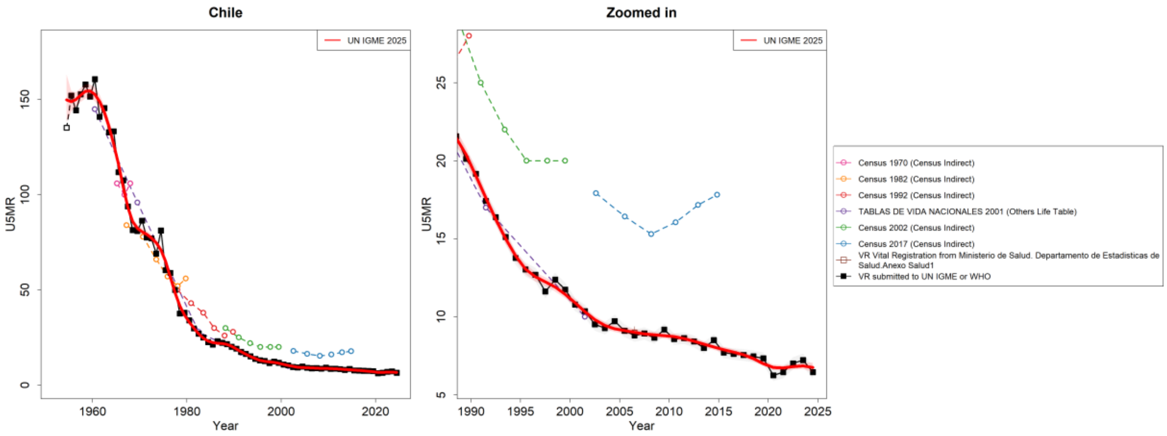


### Infant mortality rate

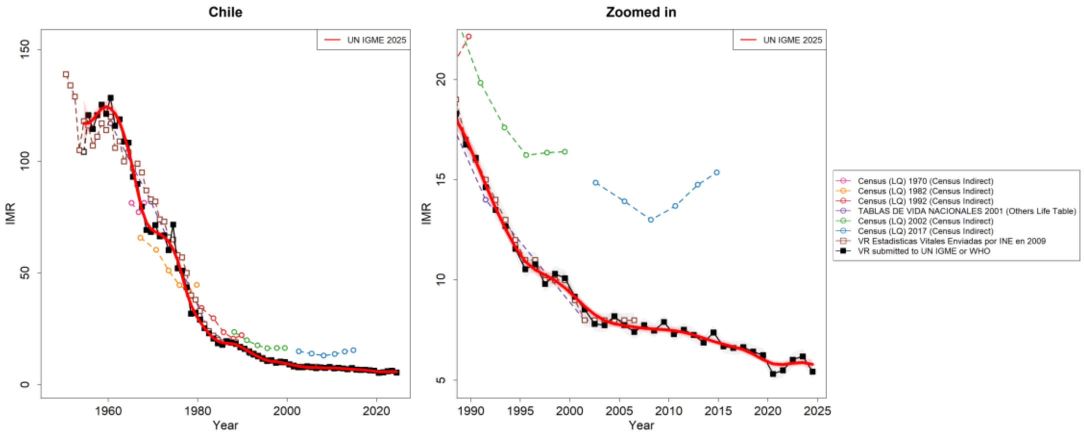


### Neonatal mortality rate

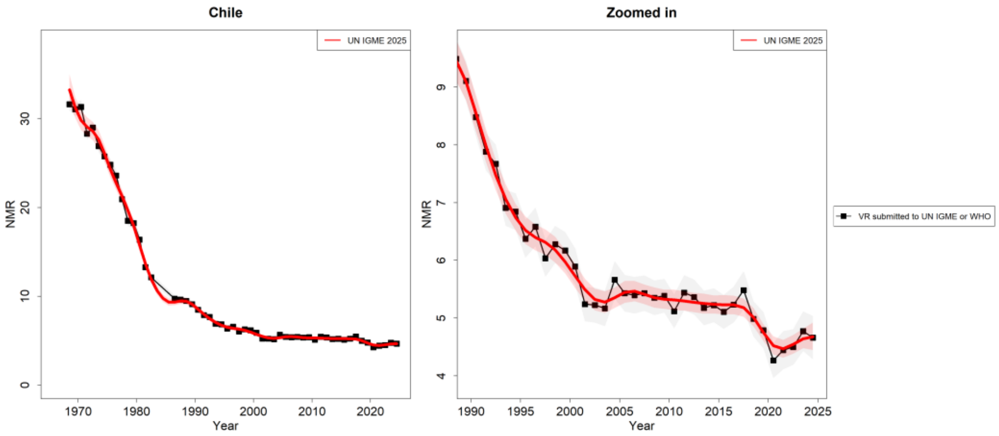

# China (CHN)

## Under-five mortality rate

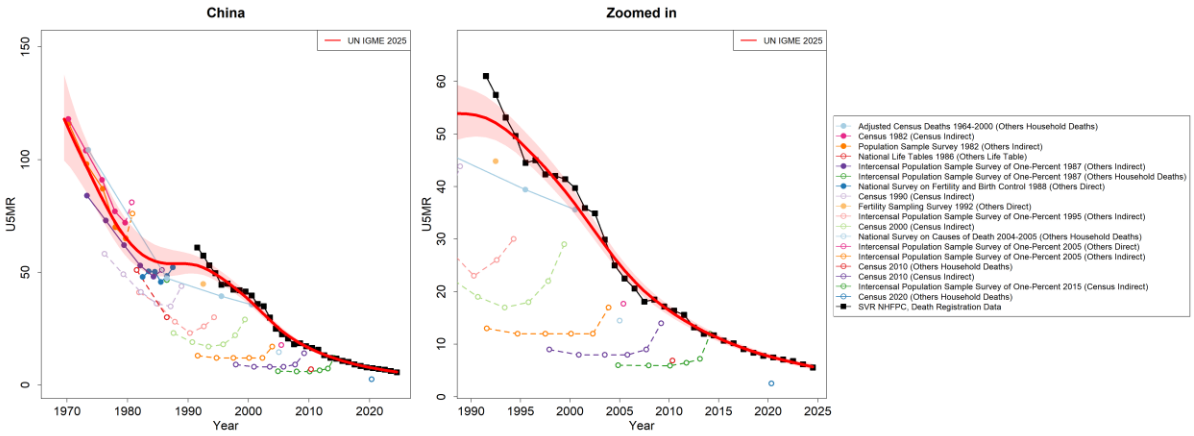


## Infant mortality rate

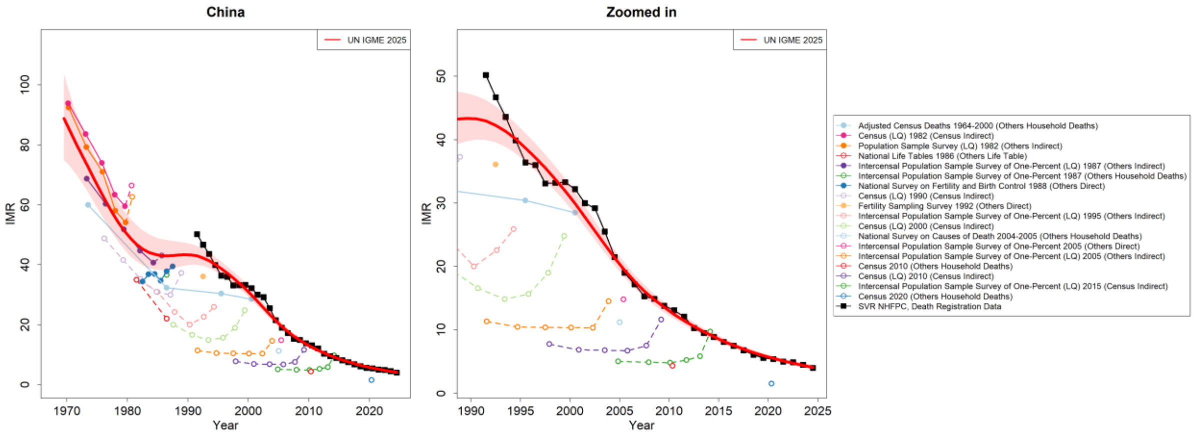


## Neonatal mortality rate

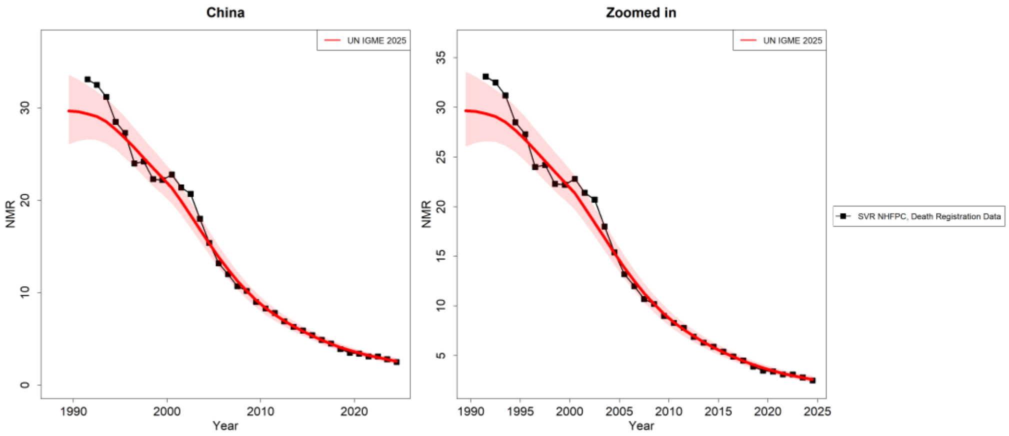

## Colombia (COL)

### Under-five mortality rate

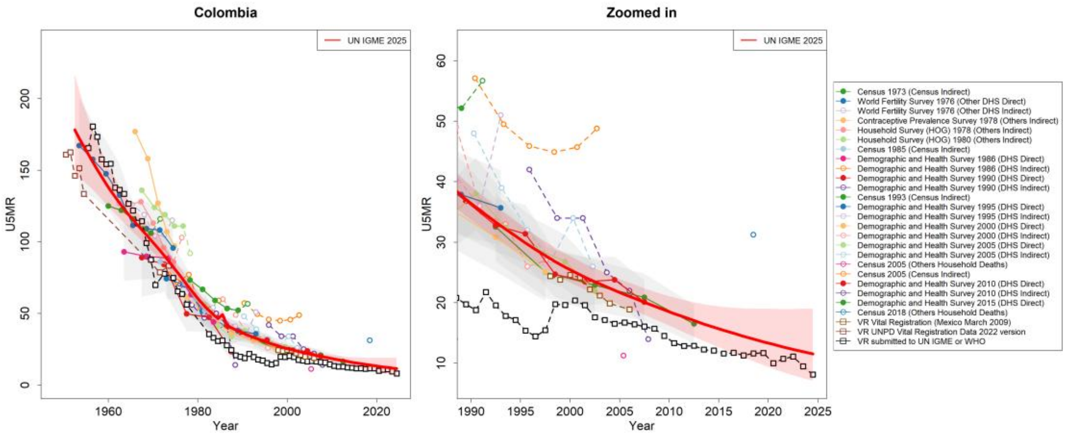


### Infant mortality rate

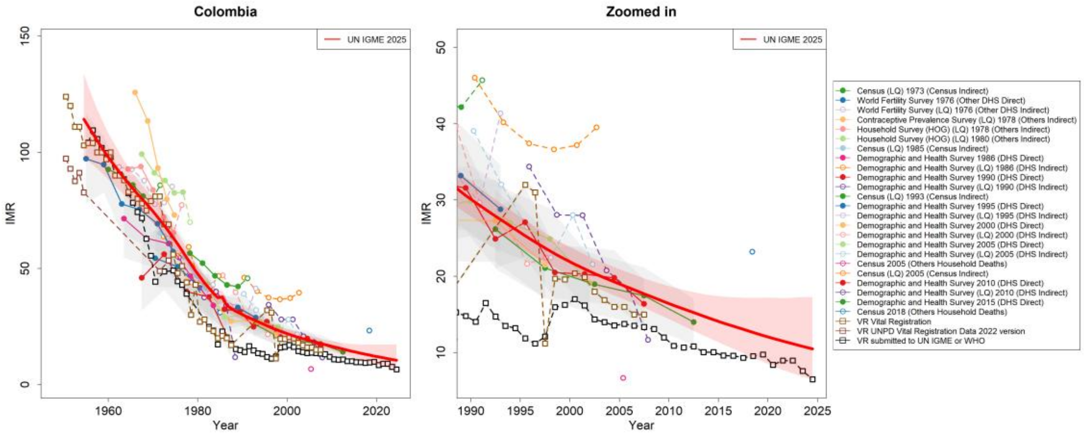


### Neonatal mortality rate

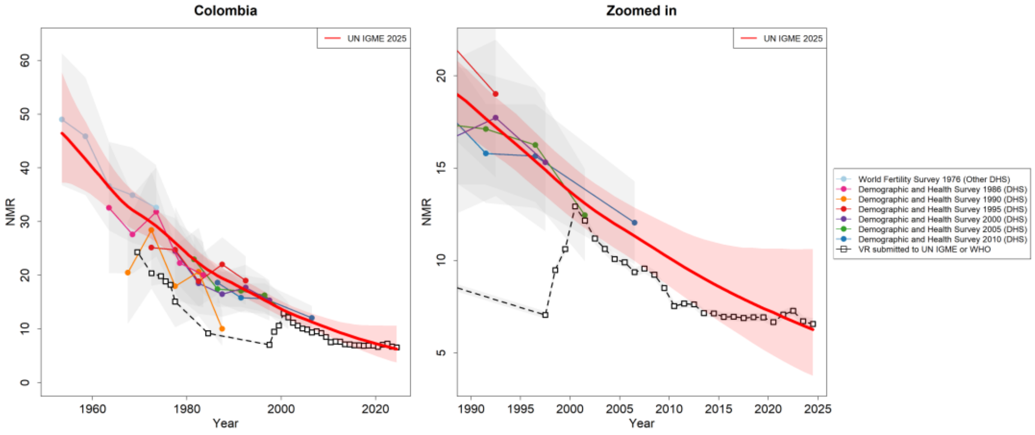

# Comoros (COM)

## Under-five mortality rate

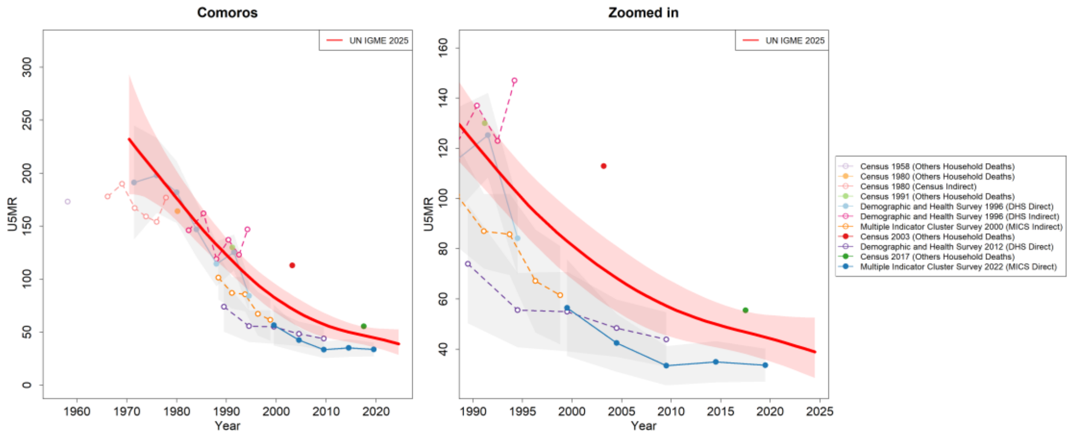


## Infant mortality rate

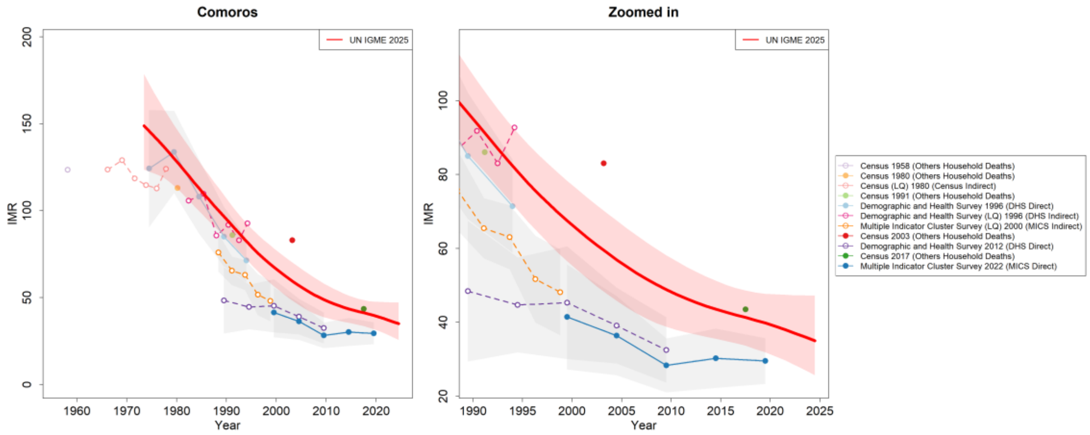


## Neonatal mortality rate

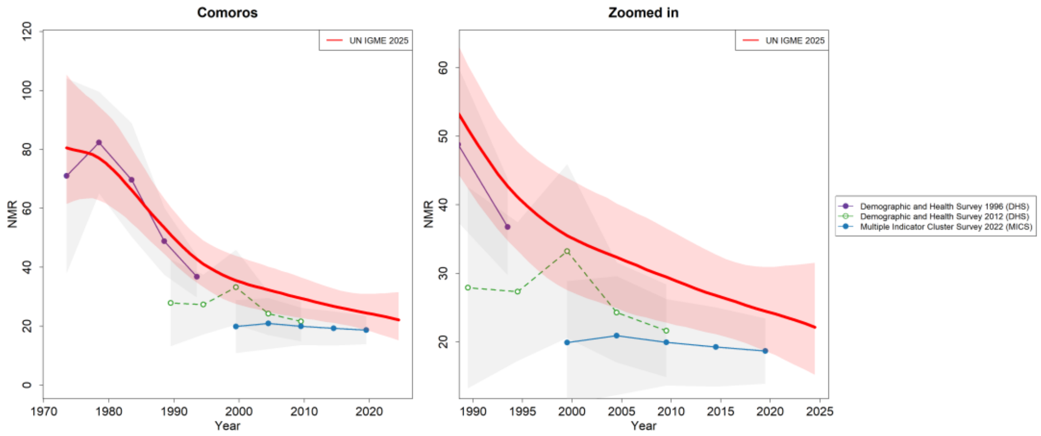

## Congo (COG)

### Under-five mortality rate

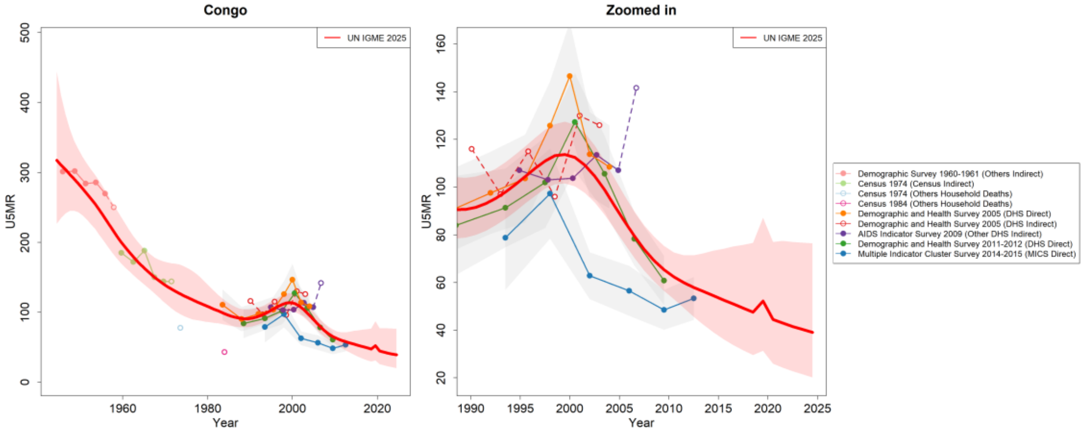


### Infant mortality rate

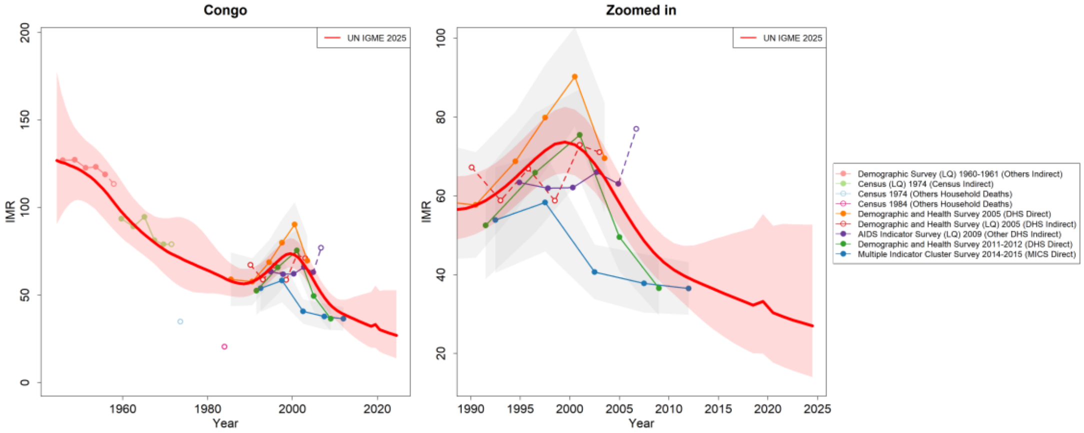


### Neonatal mortality rate

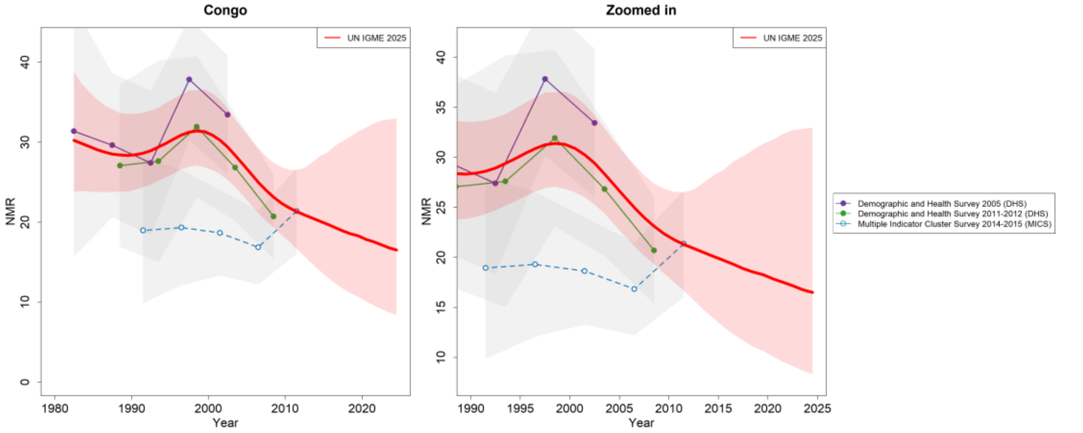

## Cook Islands (COK)

### Under-five mortality rate

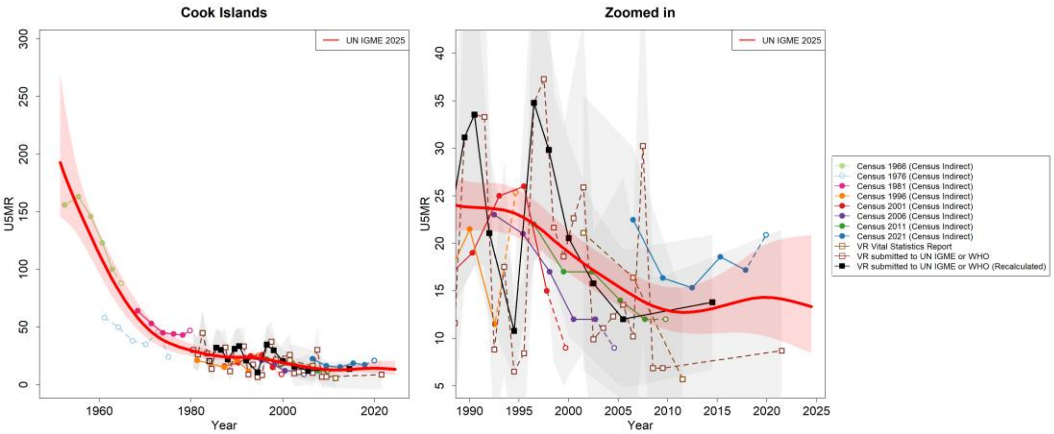


### Infant mortality rate

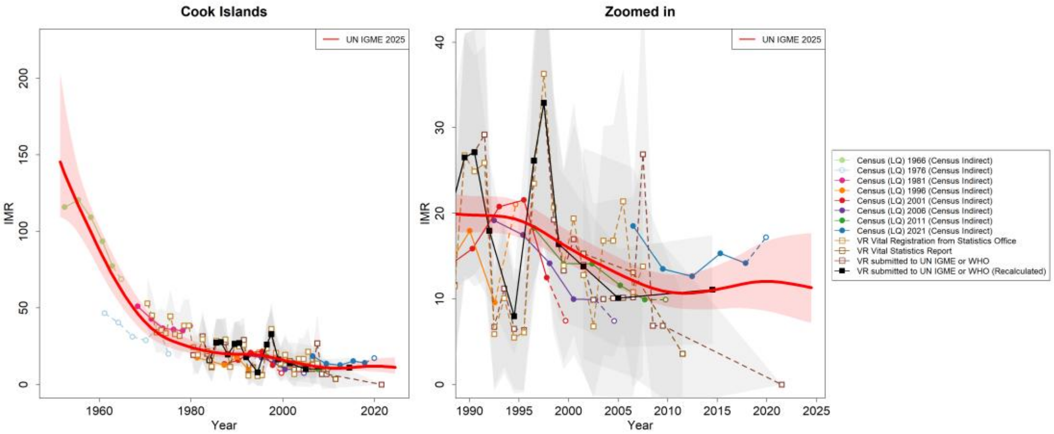


### Neonatal mortality rate

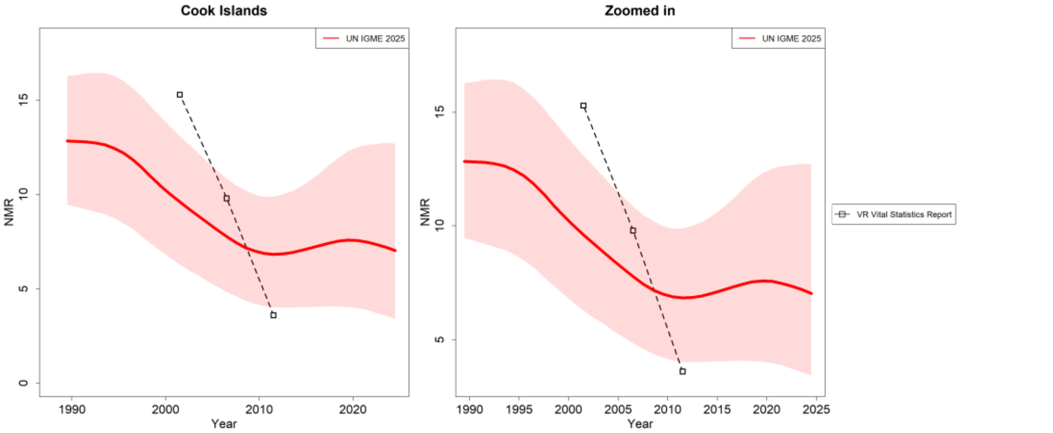

## Costa Rica (CRI)

### Under-five mortality rate

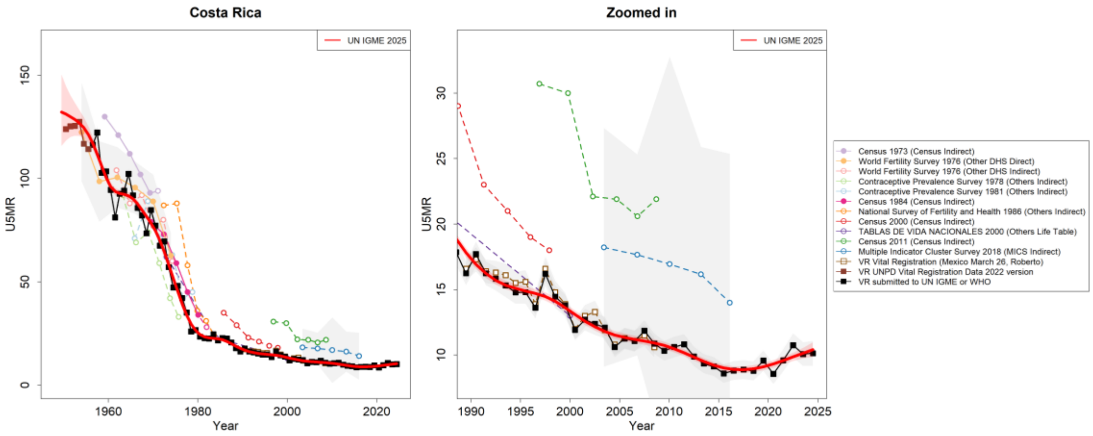


### Infant mortality rate

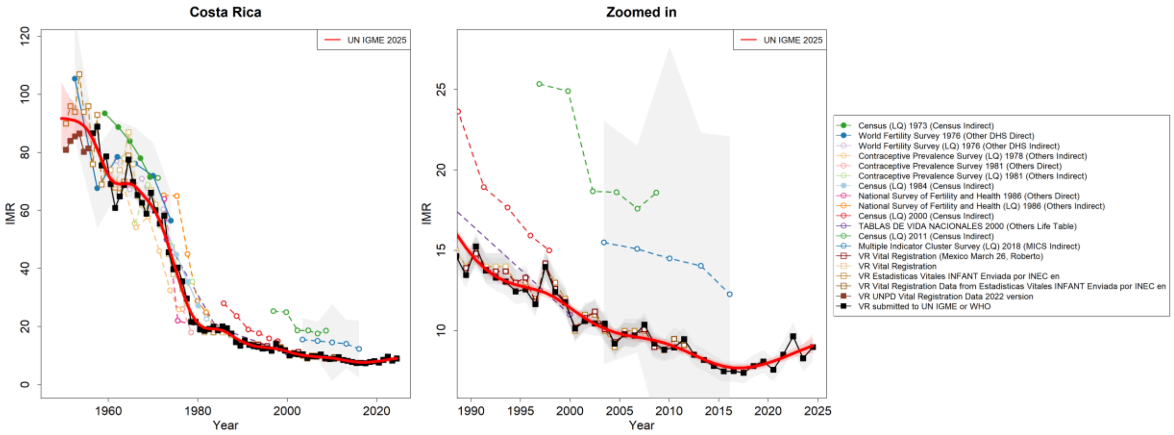


### Neonatal mortality rate

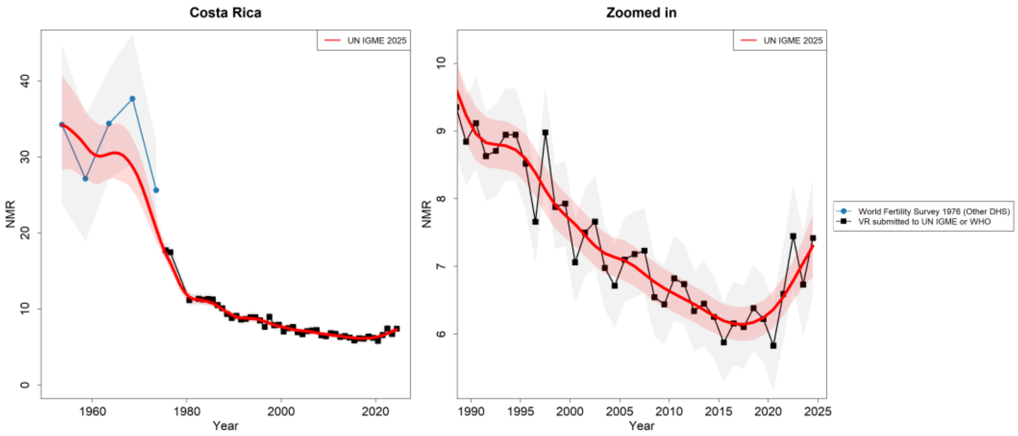

## Croatia (HRV)

### Under-five mortality rate

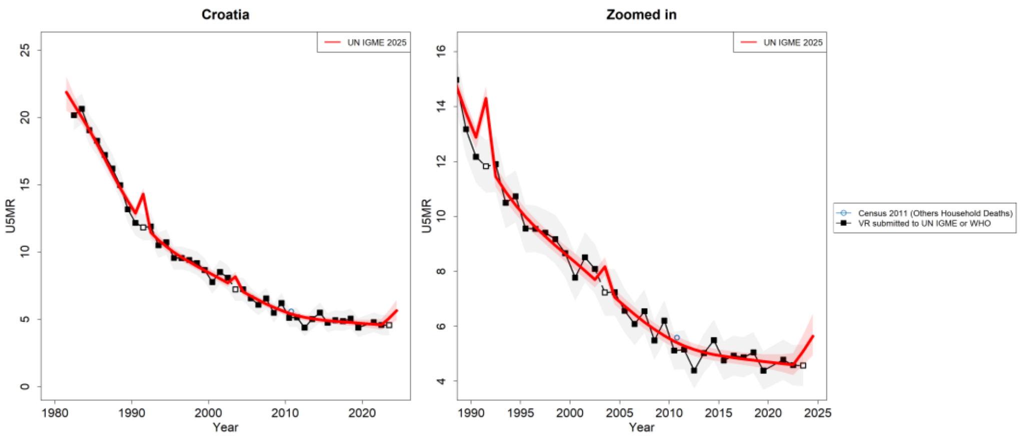


### Infant mortality rate

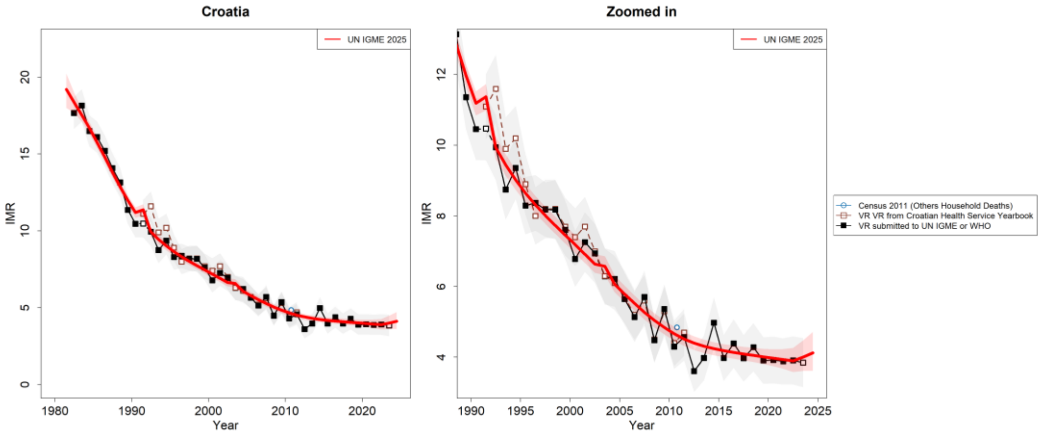


### Neonatal mortality rate

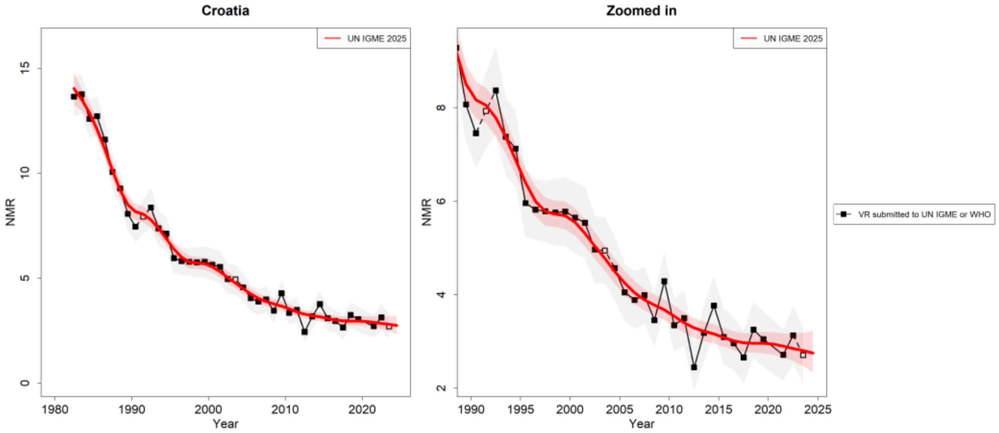

## Cuba (CUB)

### Under-five mortality rate

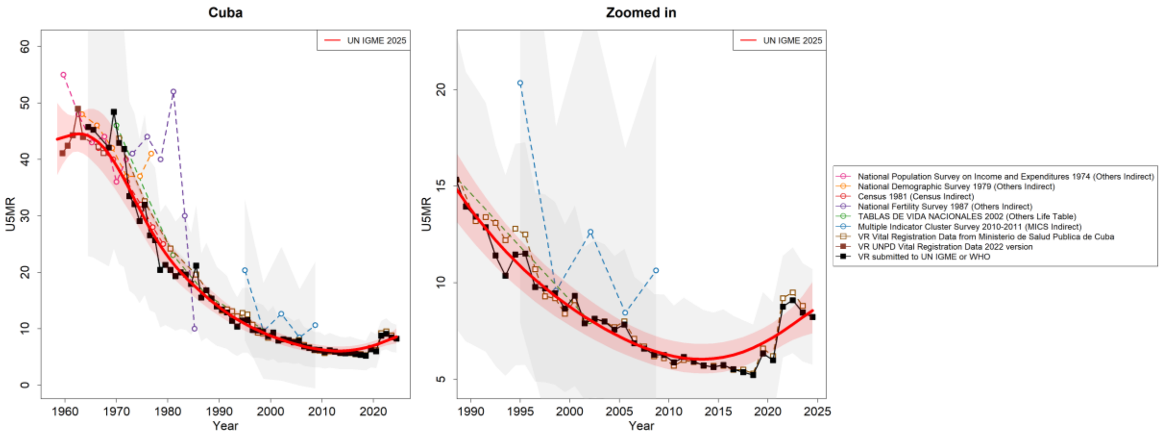


### Infant mortality rate

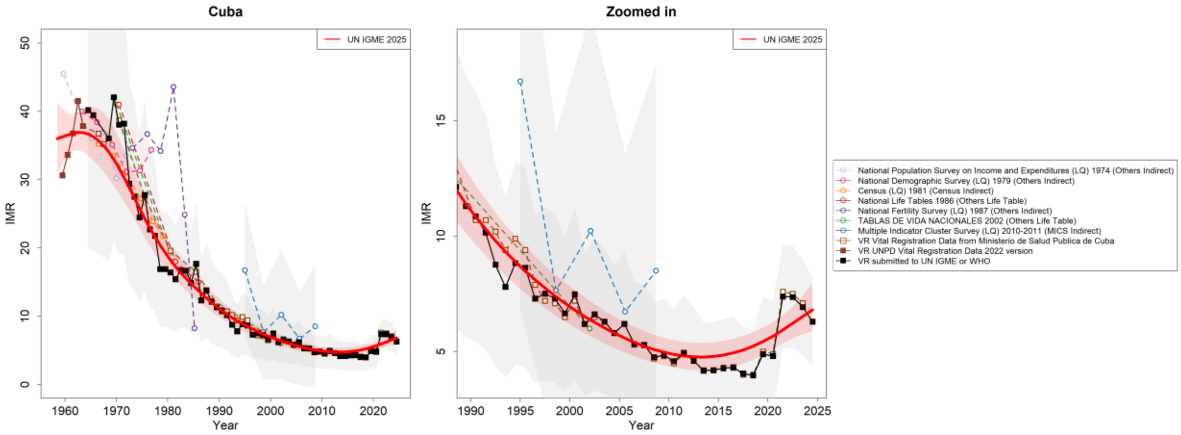


### Neonatal mortality rate

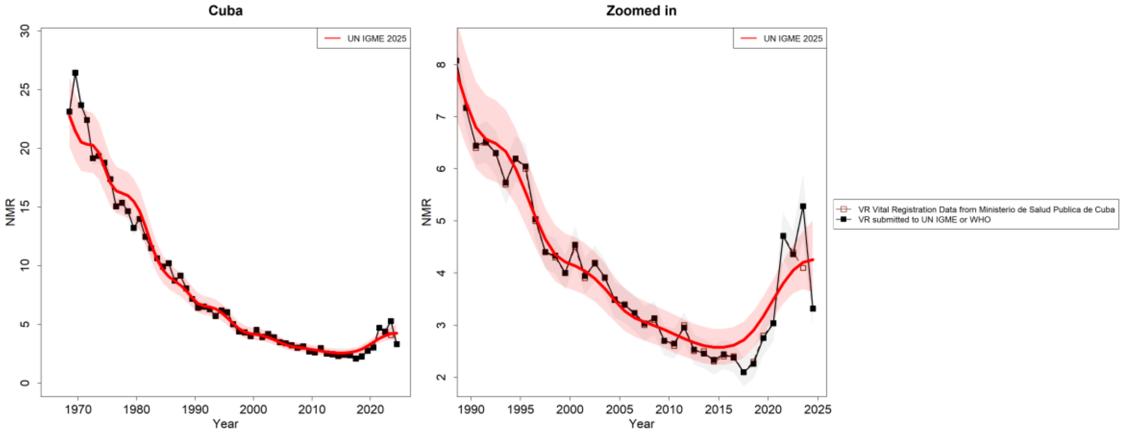

# Cyprus (CYP)

## Under-five mortality rate

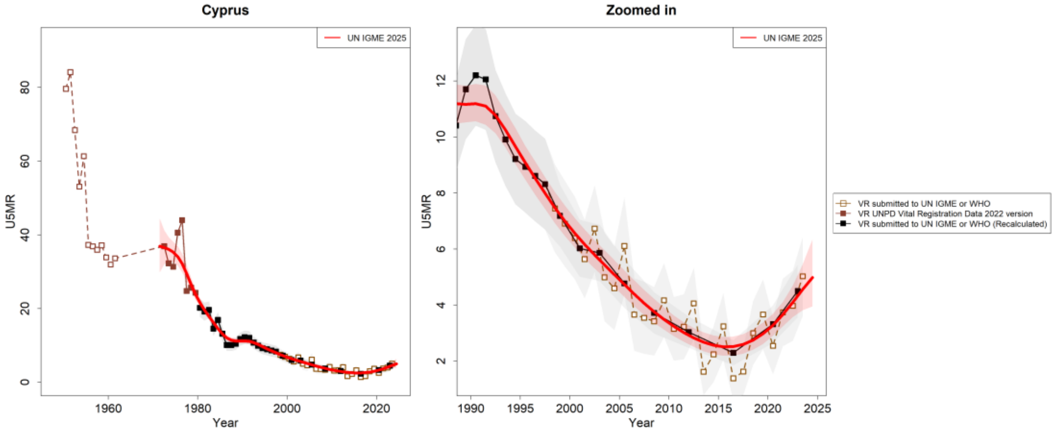


## Infant mortality rate

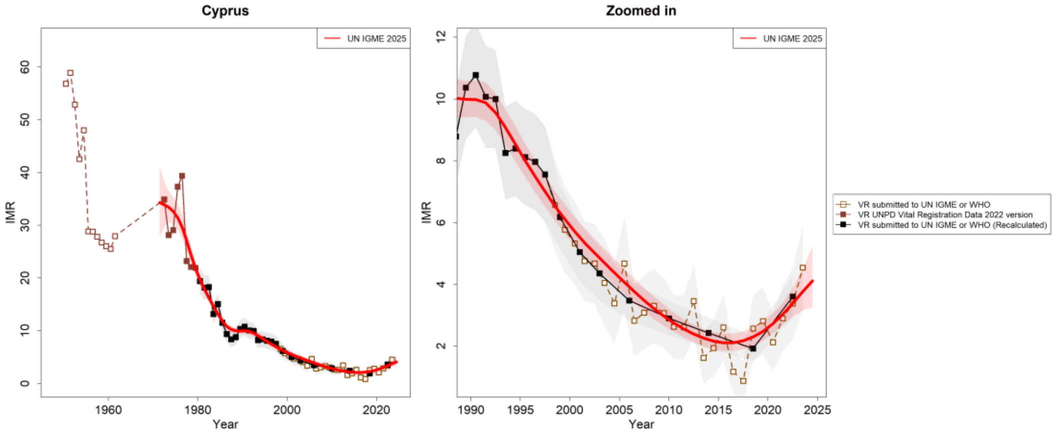


## Neonatal mortality rate

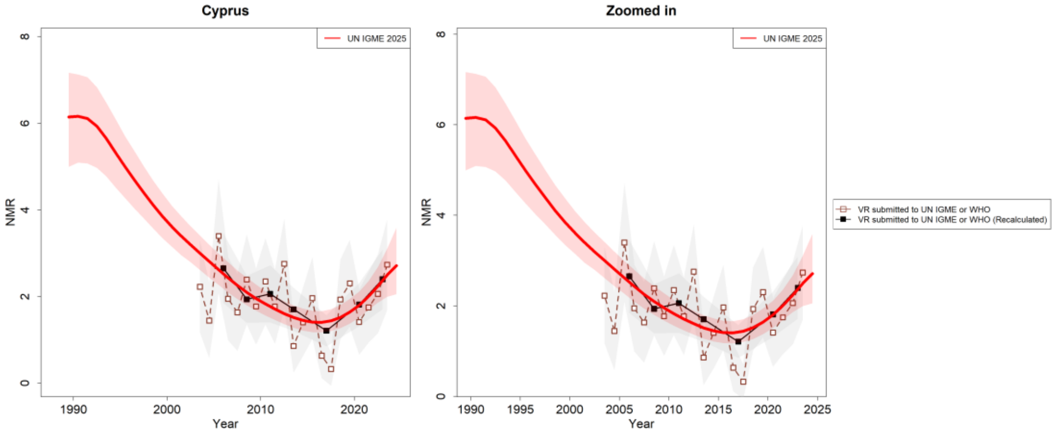

## Czechia (CZE)

### Under-five mortality rate

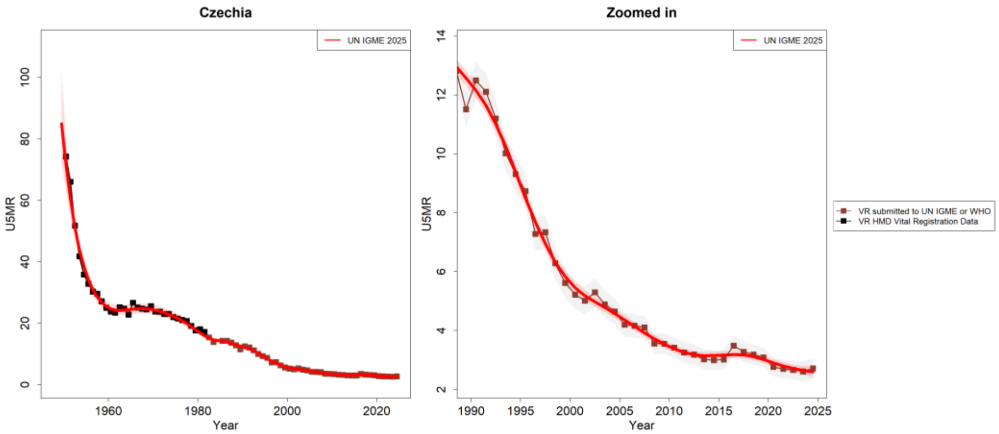


### Infant mortality rate

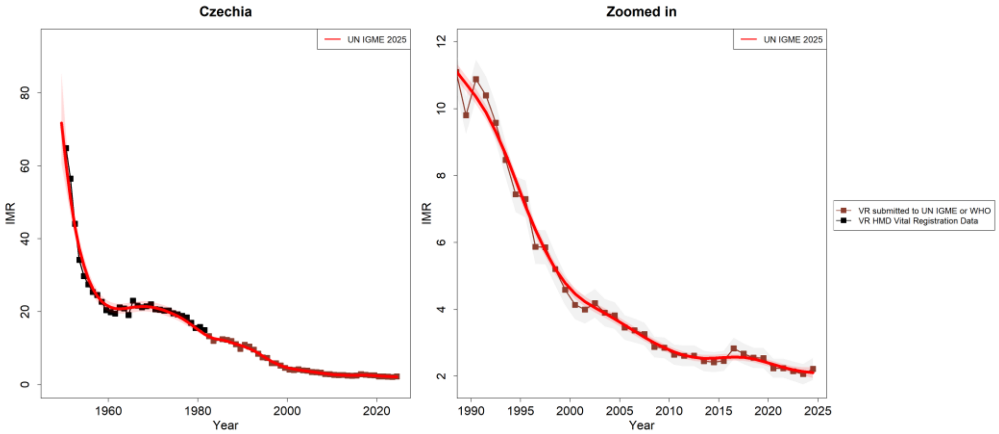


### Neonatal mortality rate

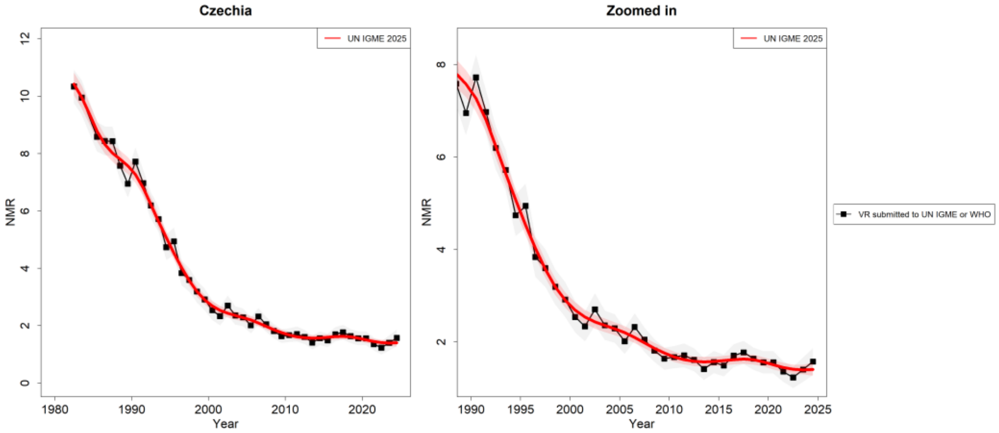

## Côte d'Ivoire (CIV)

### Under-five mortality rate

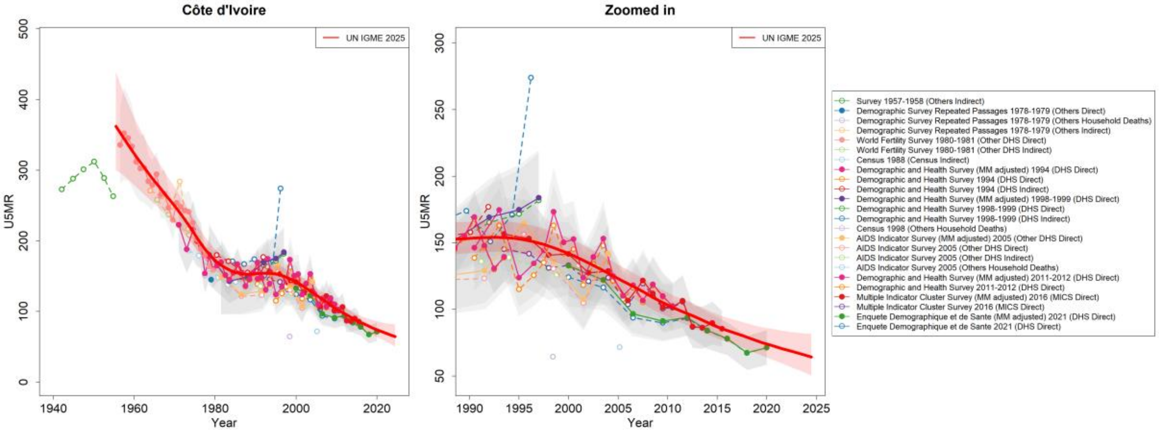


### Infant mortality rate

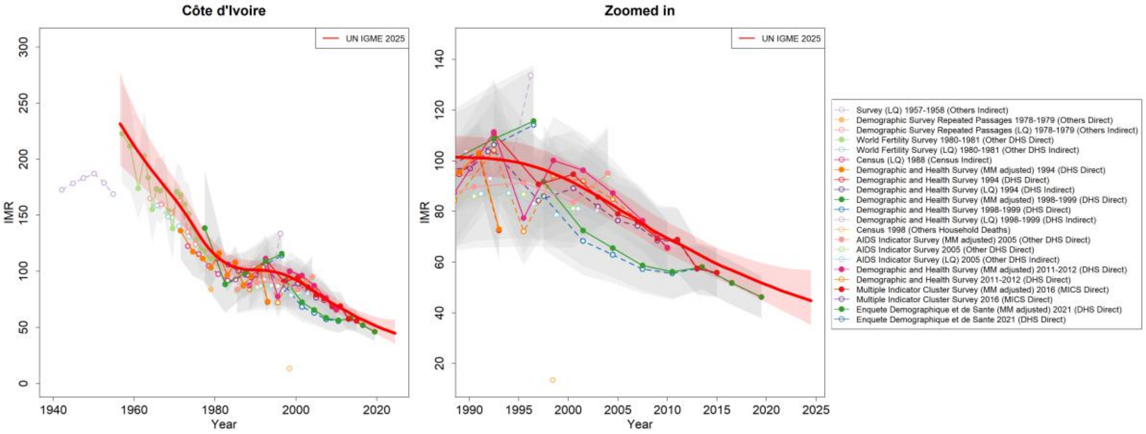


### Neonatal mortality rate

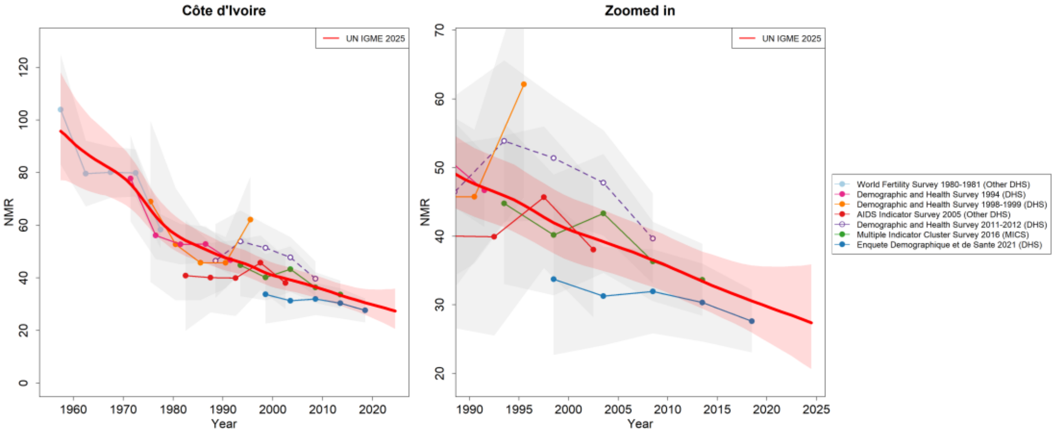

## Democratic People’s Republic of Korea (PRK)

### Under-five mortality rate

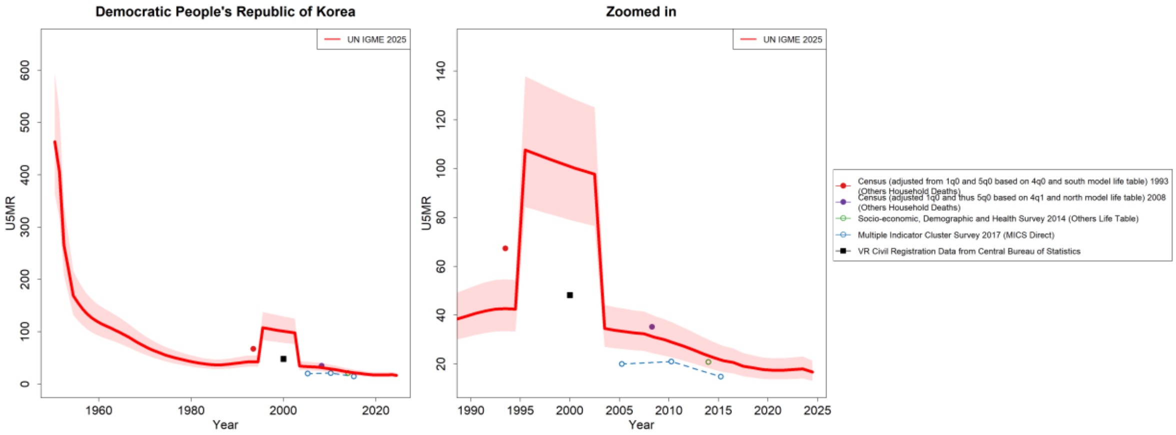


### Infant mortality rate

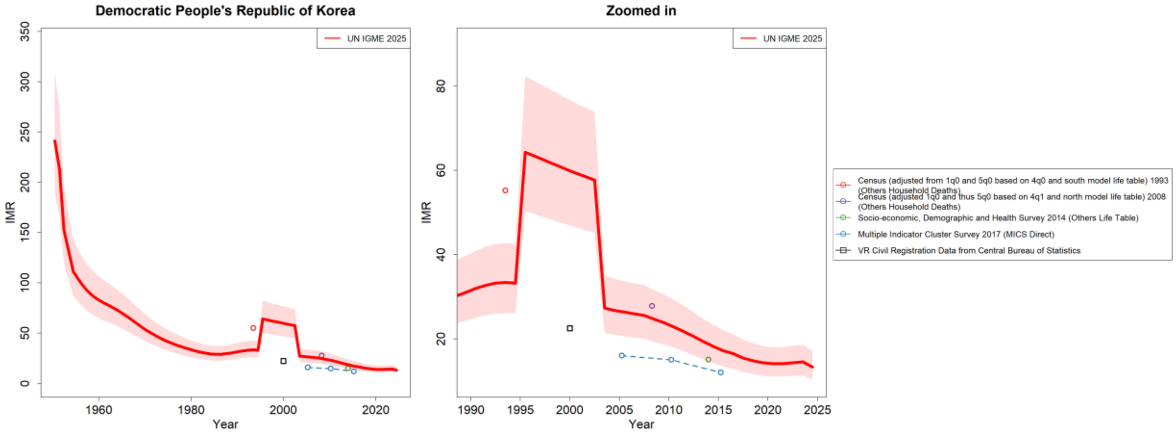


### Neonatal mortality rate

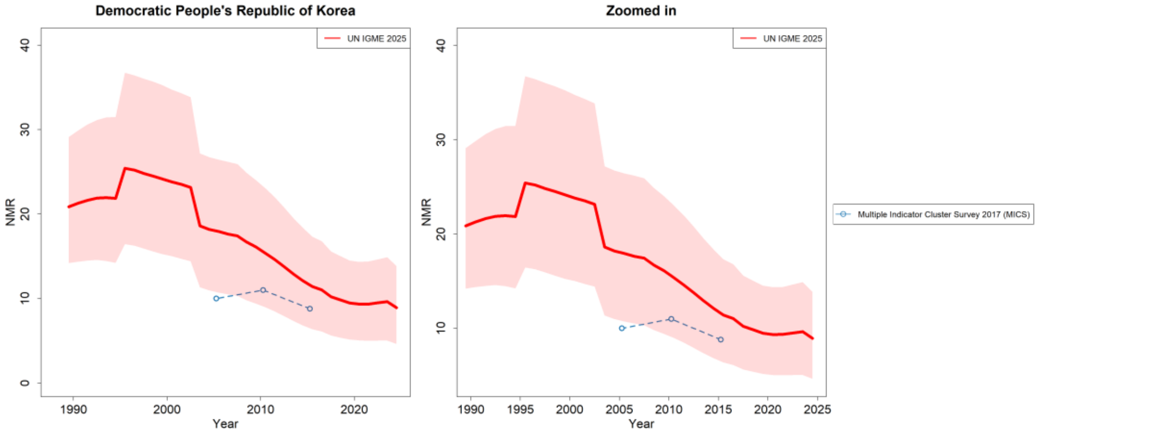

## Democratic Republic of the Congo (COD)

### Under-five mortality rate

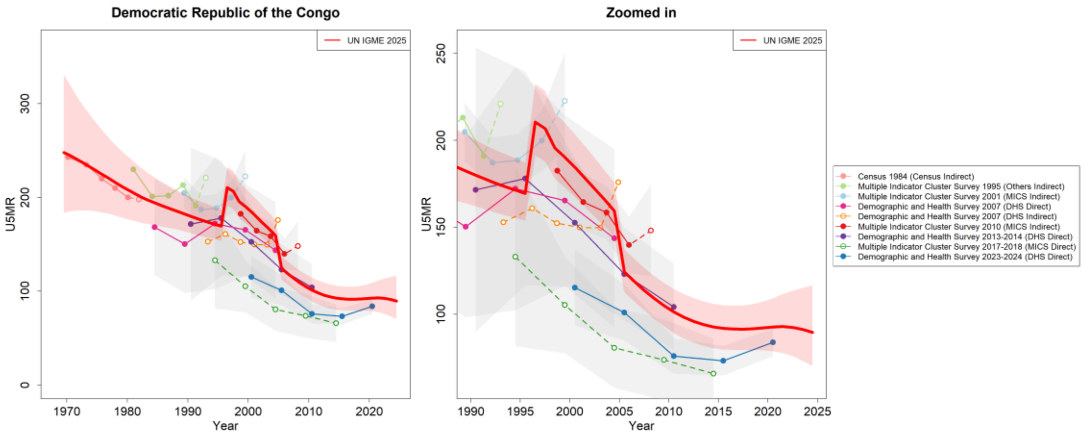


### Infant mortality rate

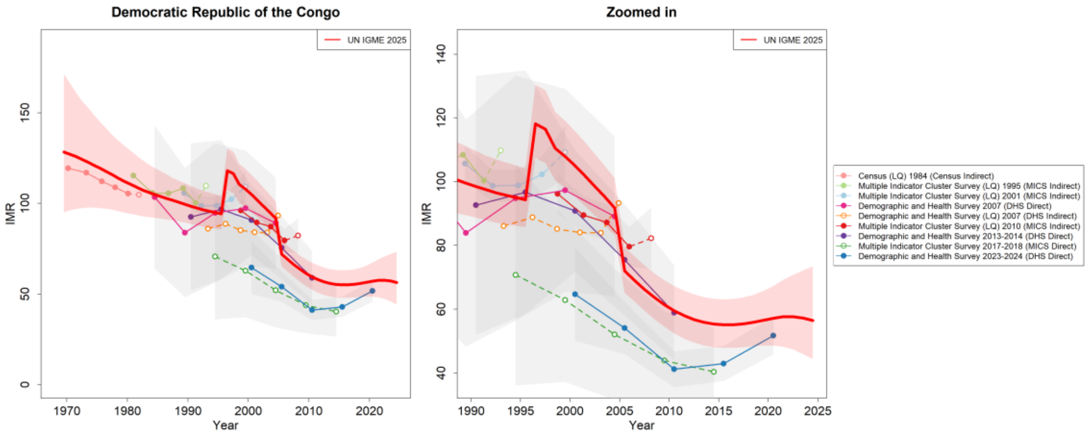


### Neonatal mortality rate

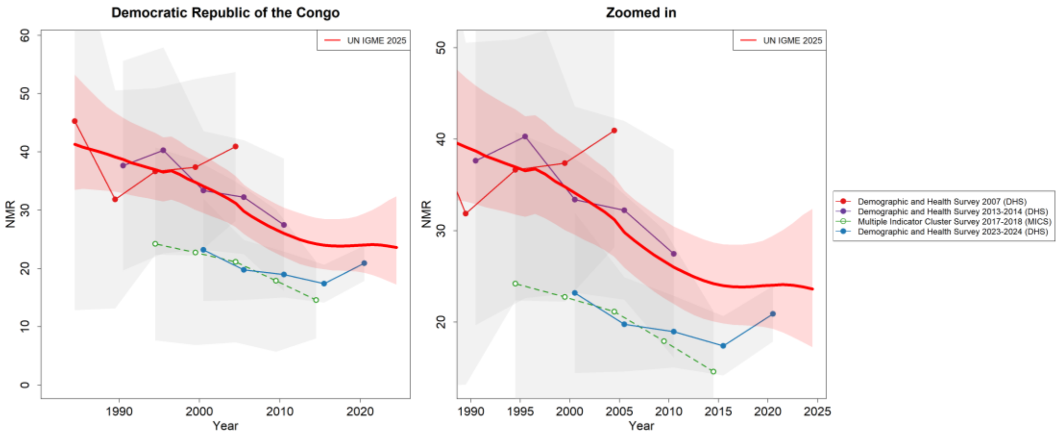

## Denmark (DNK)

### Under-five mortality rate

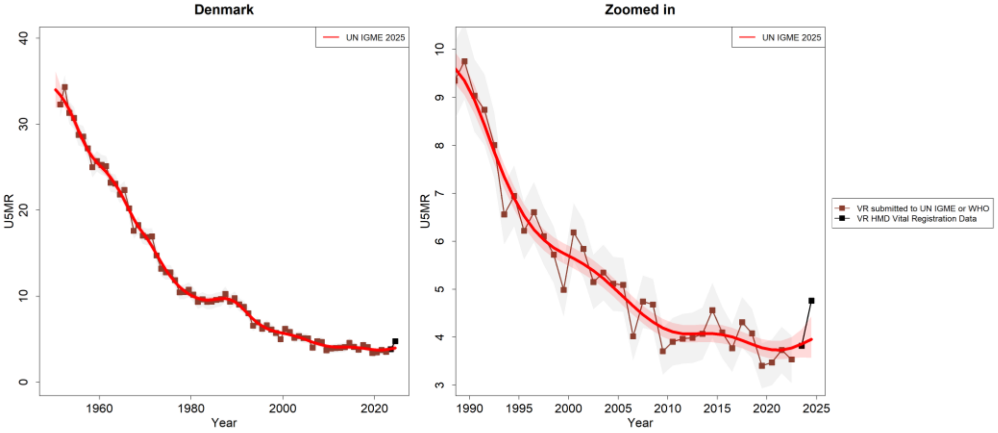


### Infant mortality rate

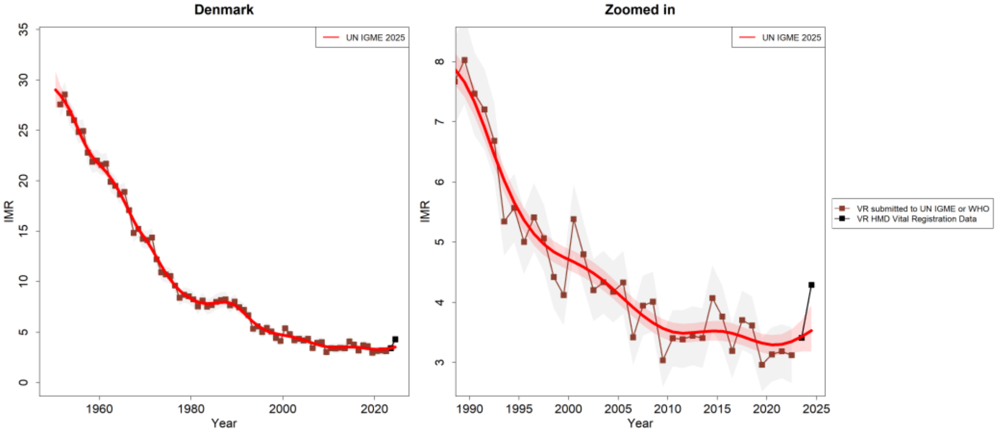


### Neonatal mortality rate

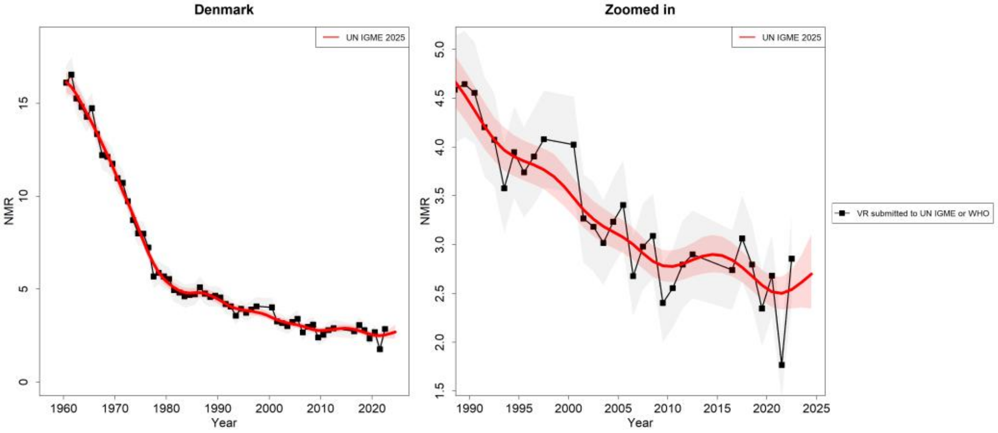

## Djibouti (DJI)

### Under-five mortality rate

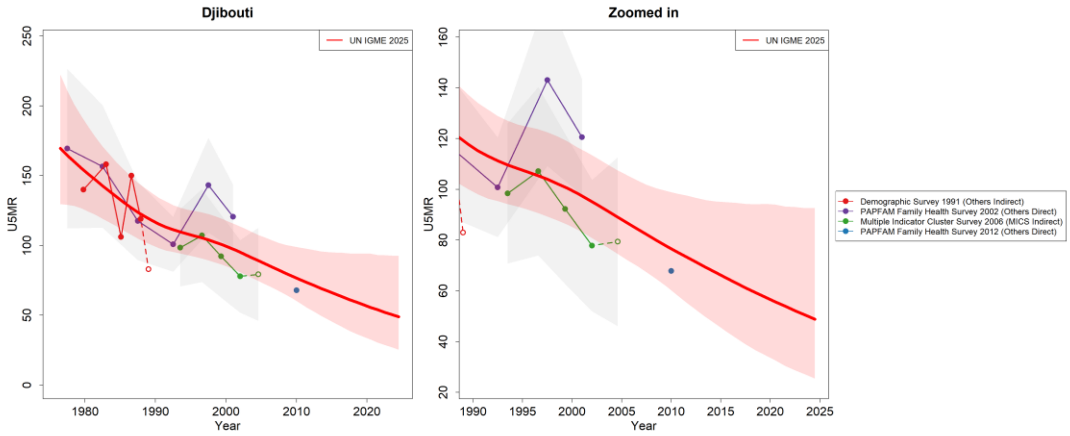


### Infant mortality rate

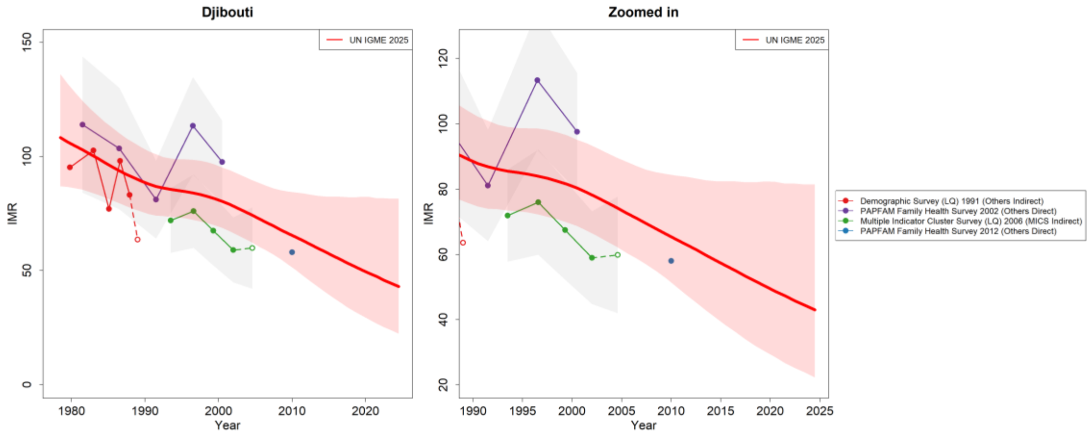


### Neonatal mortality rate

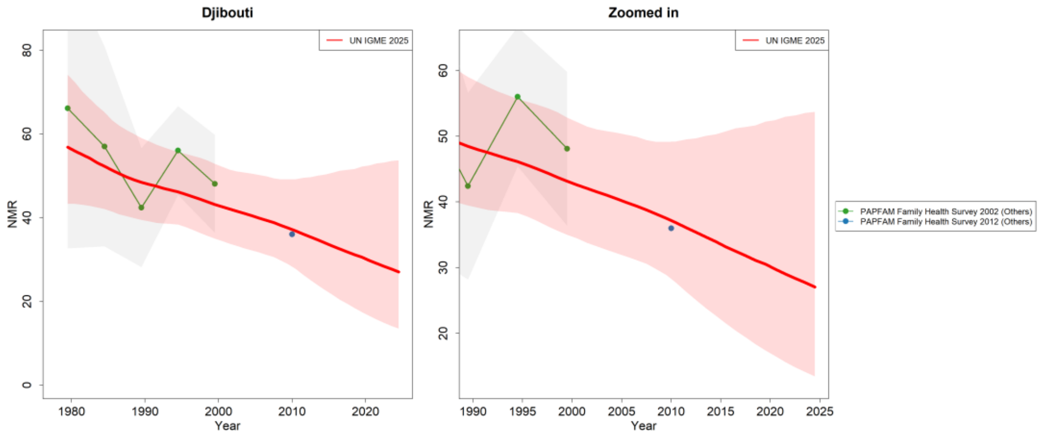

## Dominica (DMA)

### Under-five mortality rate

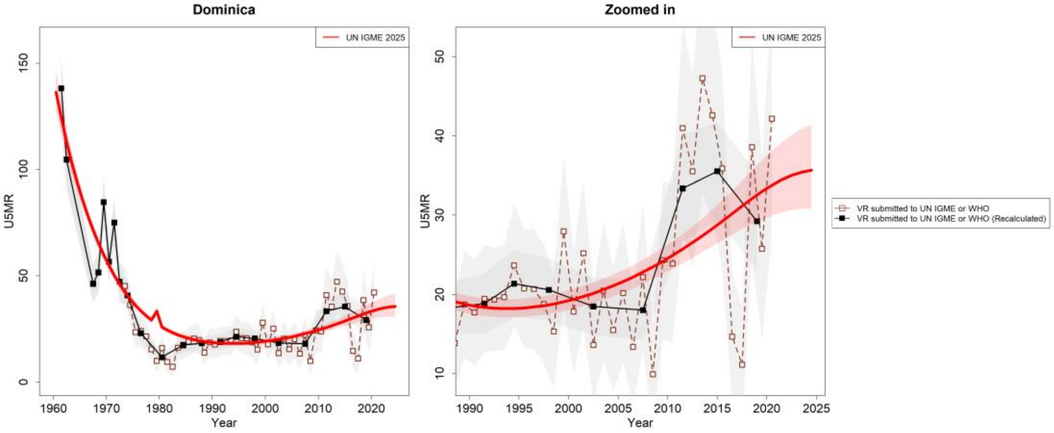


### Infant mortality rate

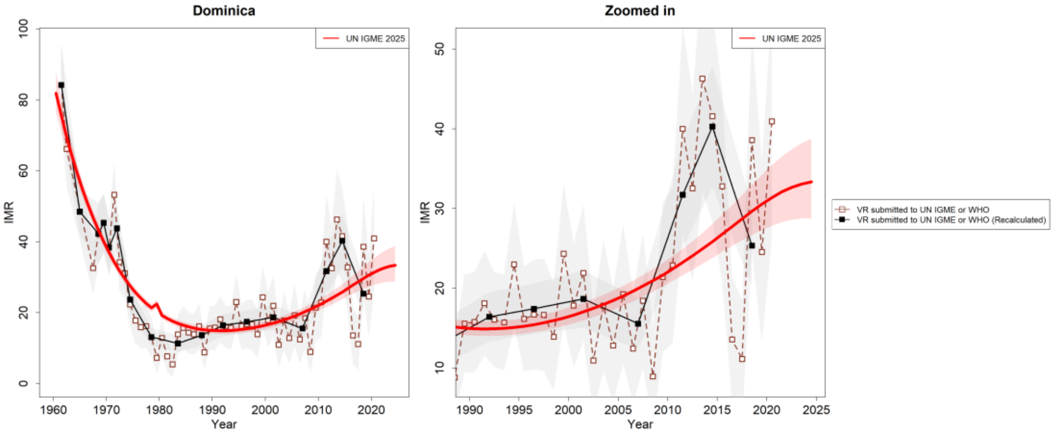


### Neonatal mortality rate

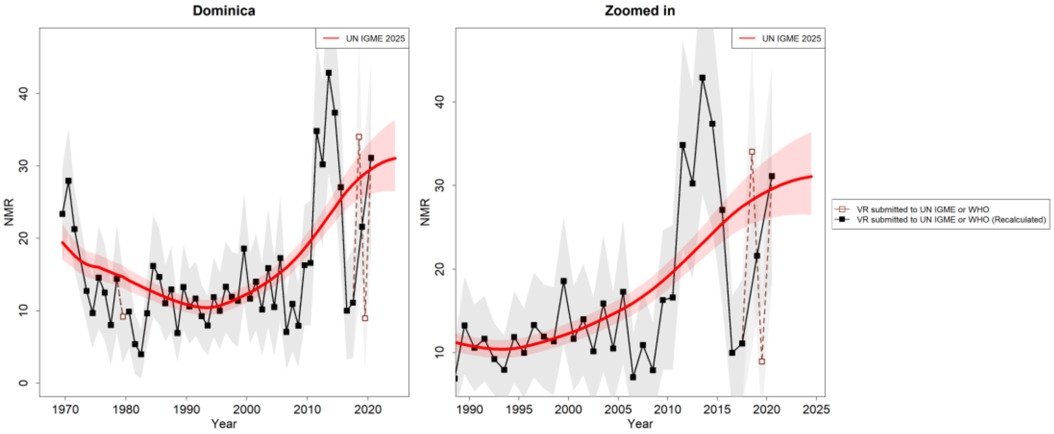

## Dominican Republic (DOM)

### Under-five mortality rate

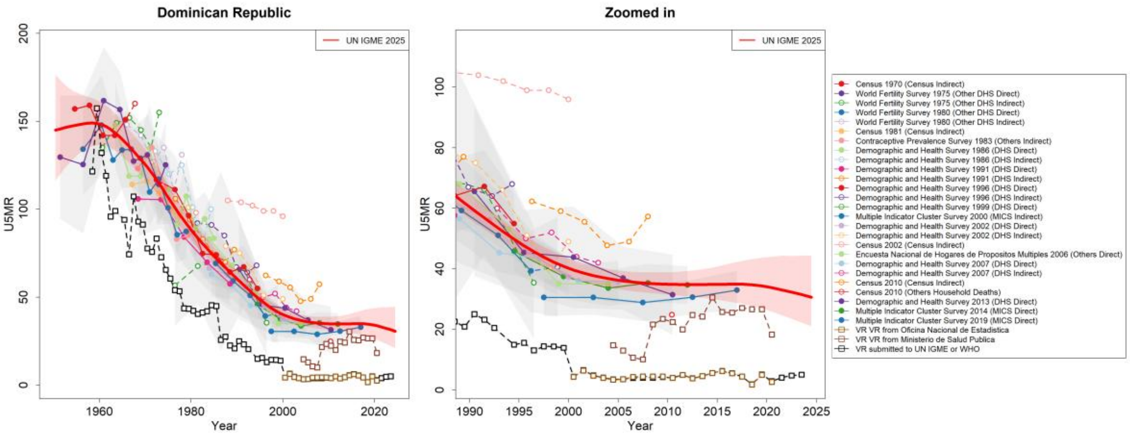


### Infant mortality rate

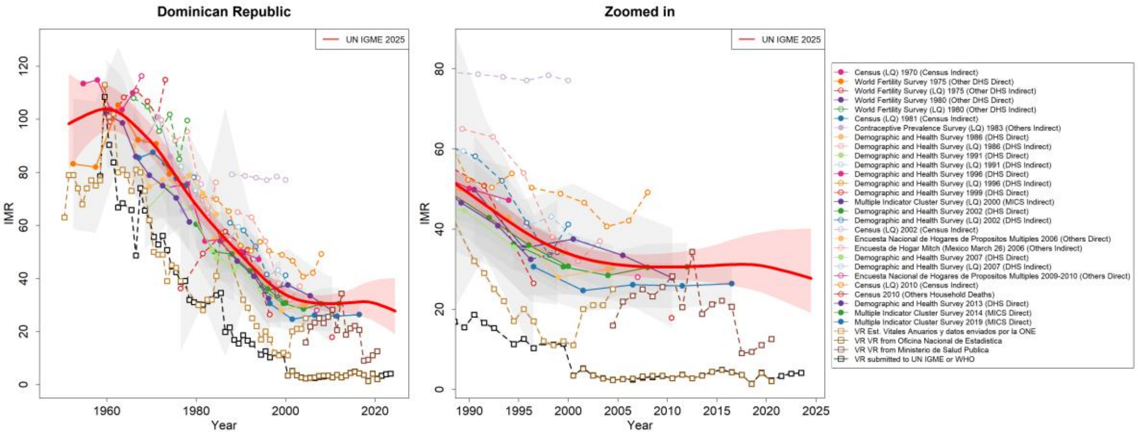


### Neonatal mortality rate

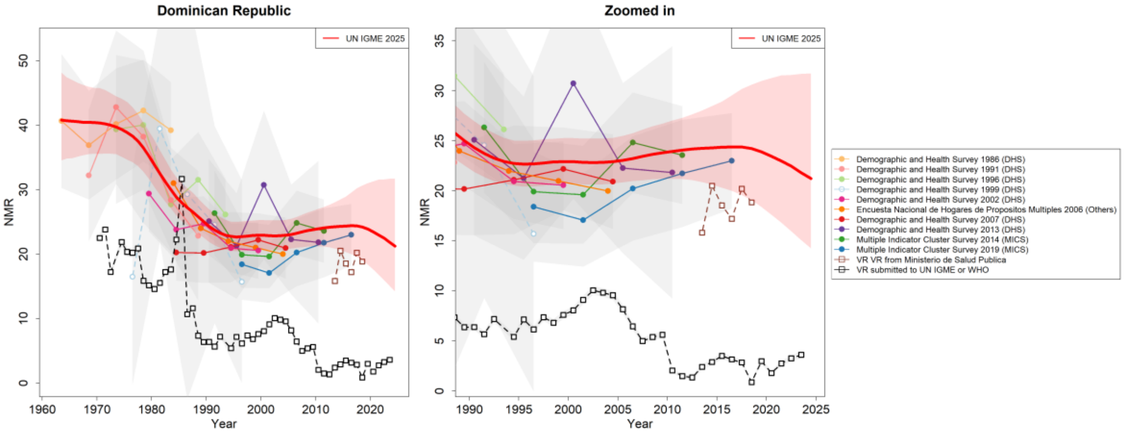

## Ecuador (ECU)

### Under-five mortality rate

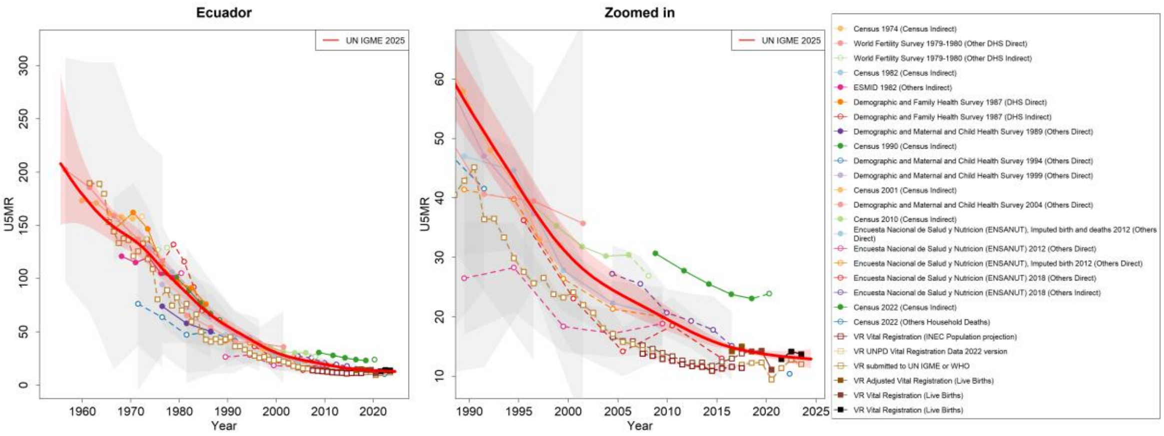


### Infant mortality rate

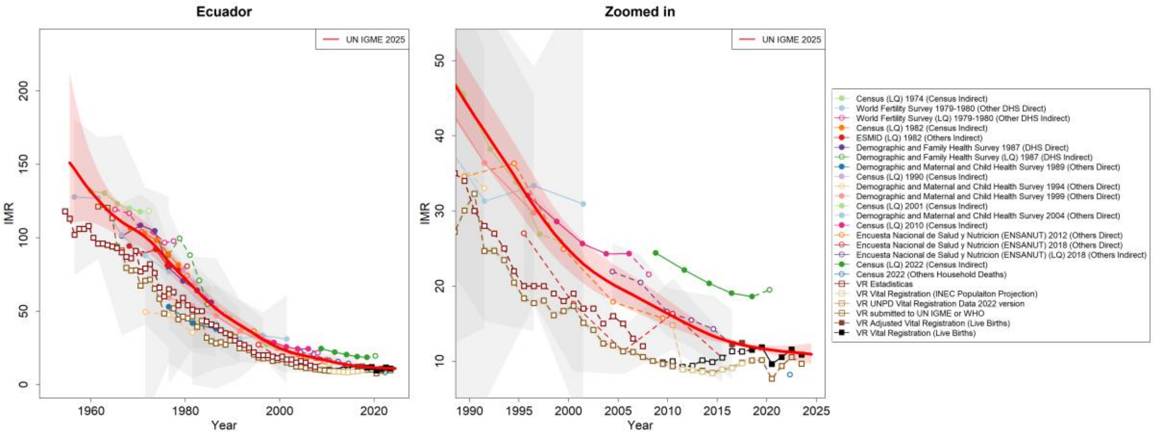


### Neonatal mortality rate

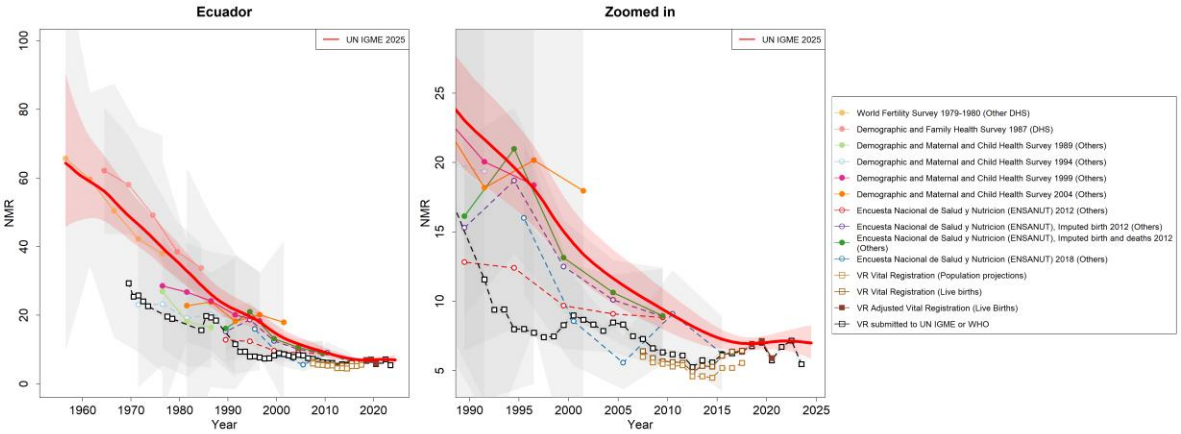

## Egypt (EGY)

### Under-five mortality rate

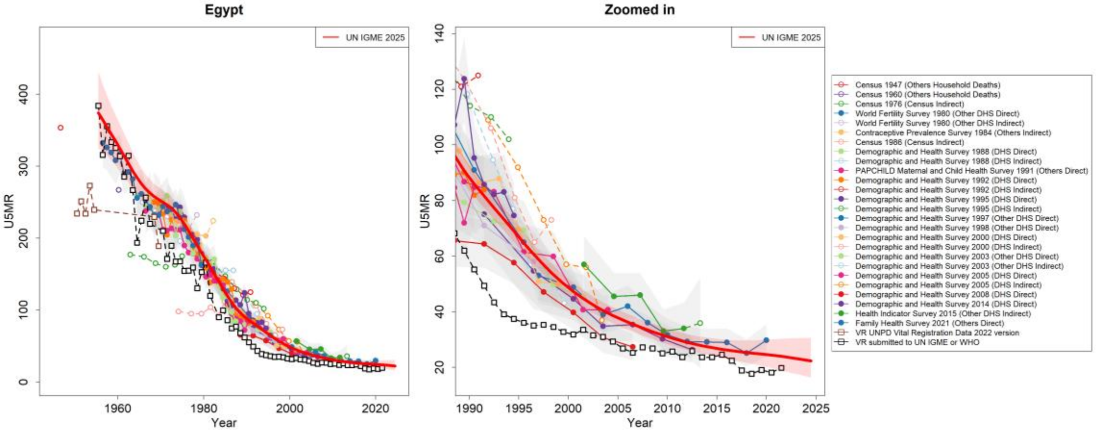


### Infant mortality rate

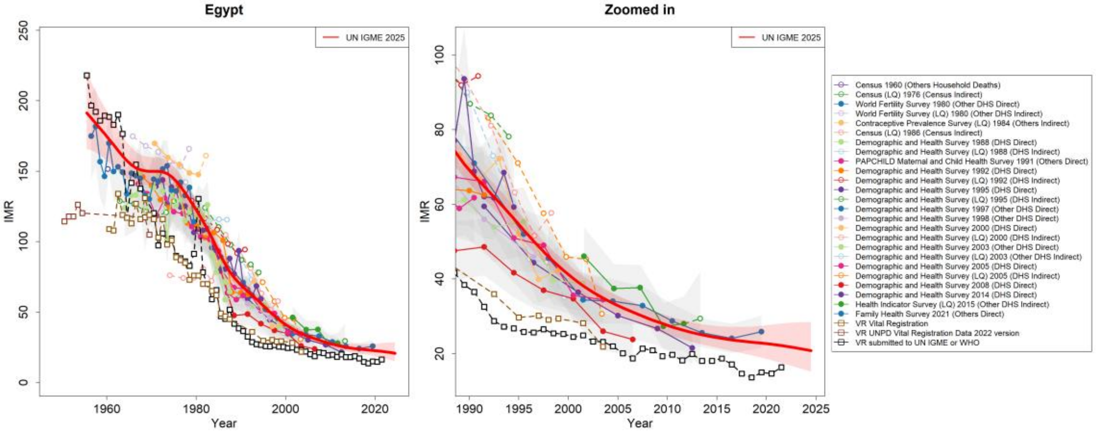


### Neonatal mortality rate

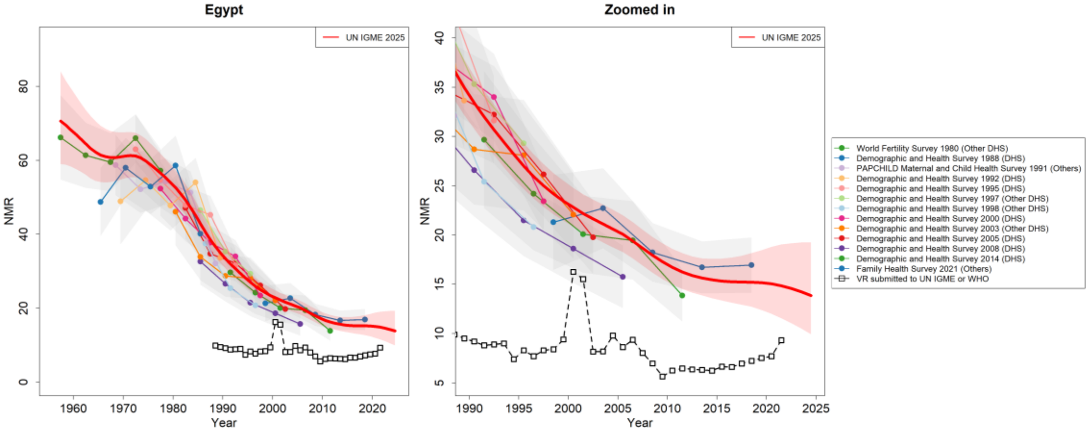

## El Salvador (SLV)

### Under-five mortality rate

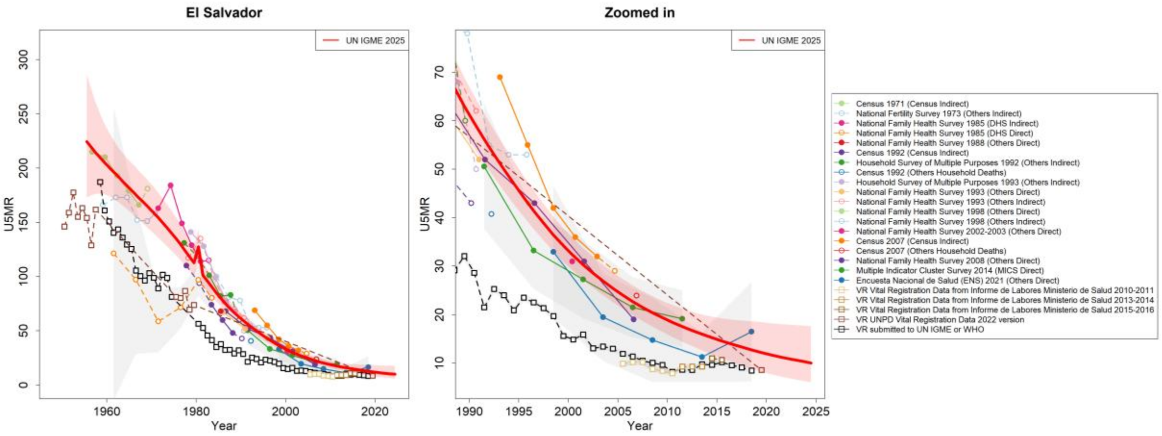


### Infant mortality rate

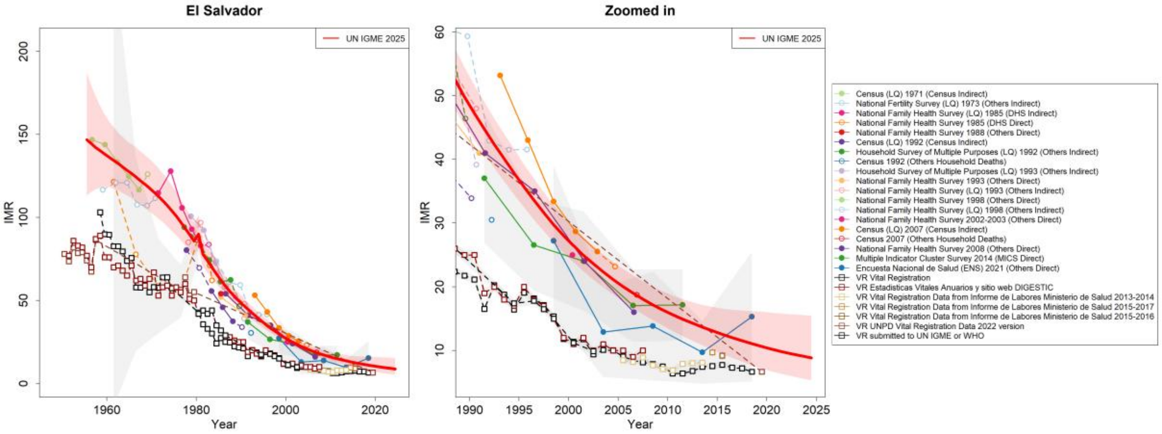


### Neonatal mortality rate

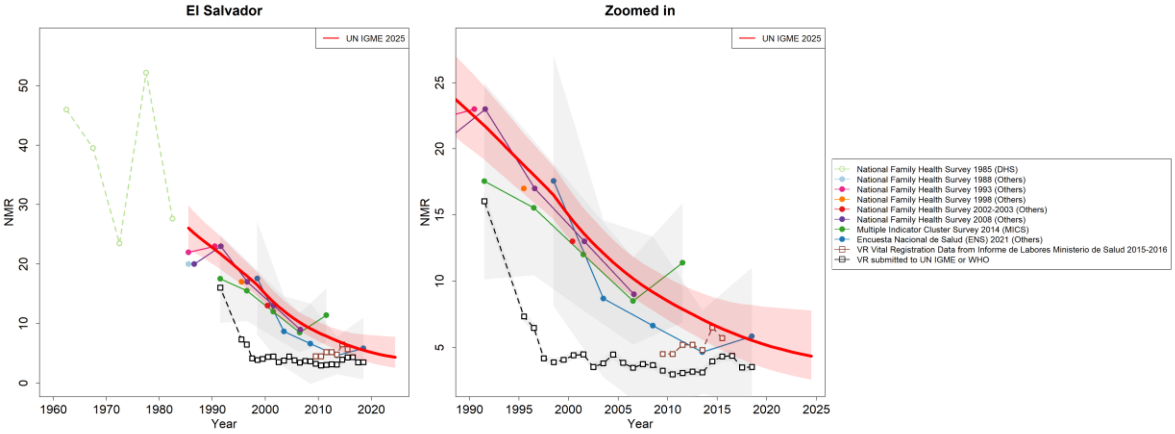

## Equatorial Guinea (GNQ)

### Under-five mortality rate

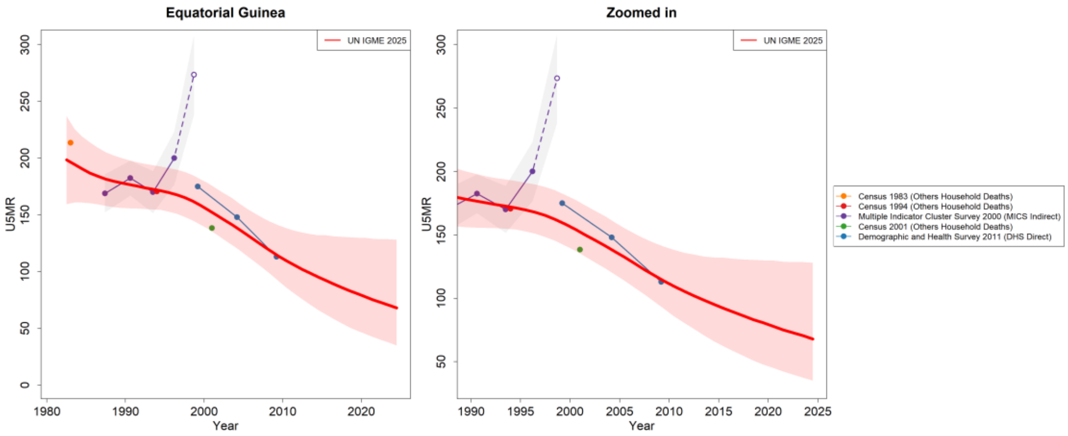


### Infant mortality rate

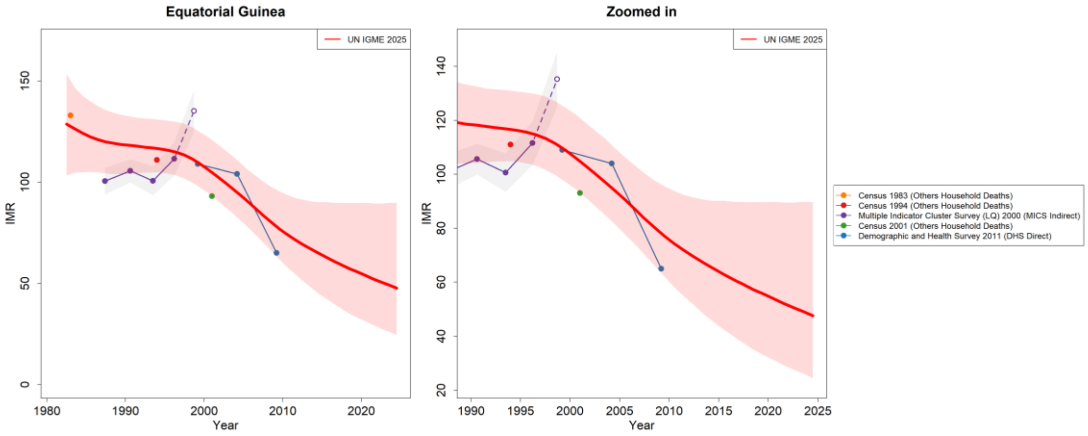


### Neonatal mortality rate

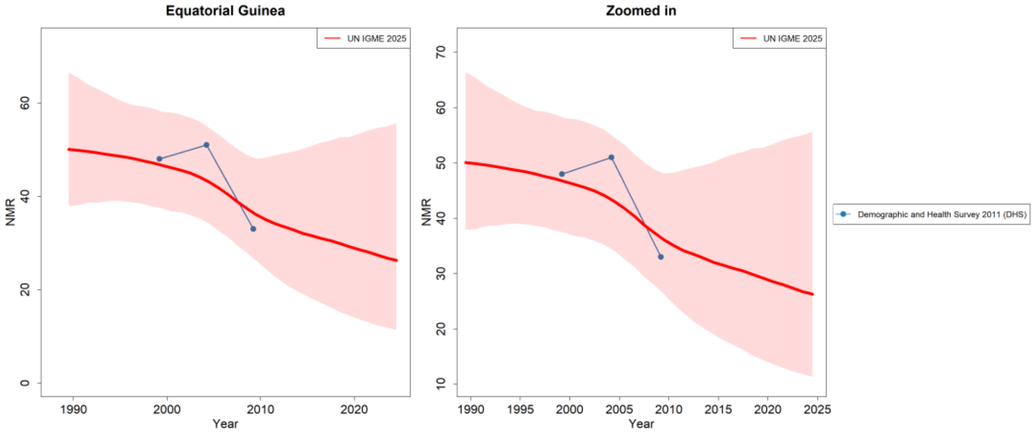

## Eritrea (ERI)

### Under-five mortality rate

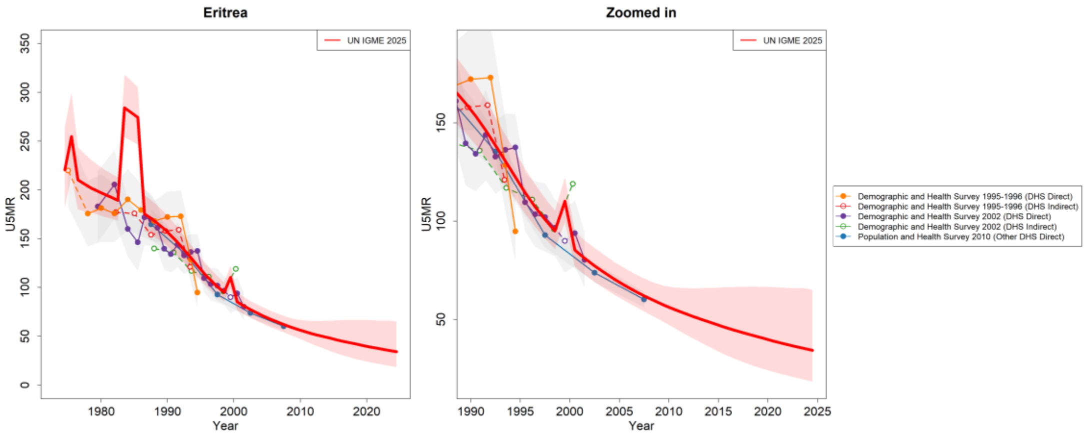


### Infant mortality rate

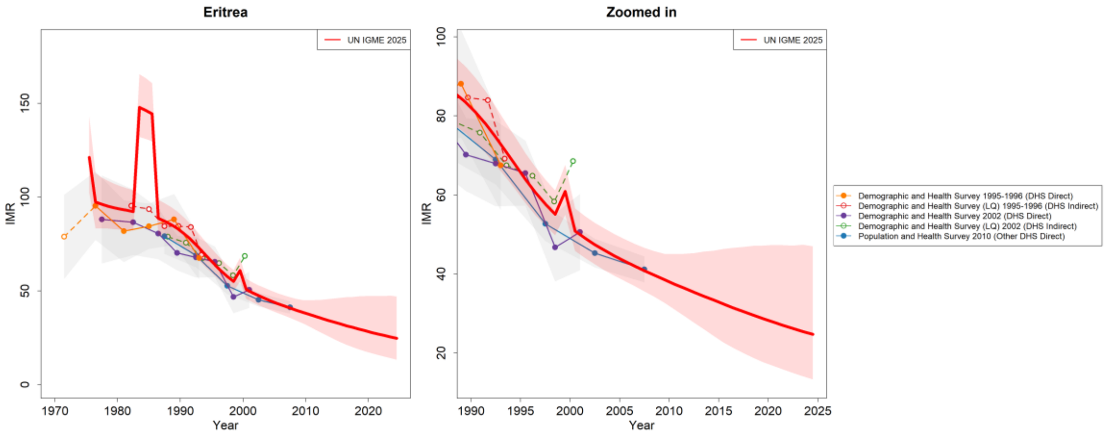


### Neonatal mortality rate

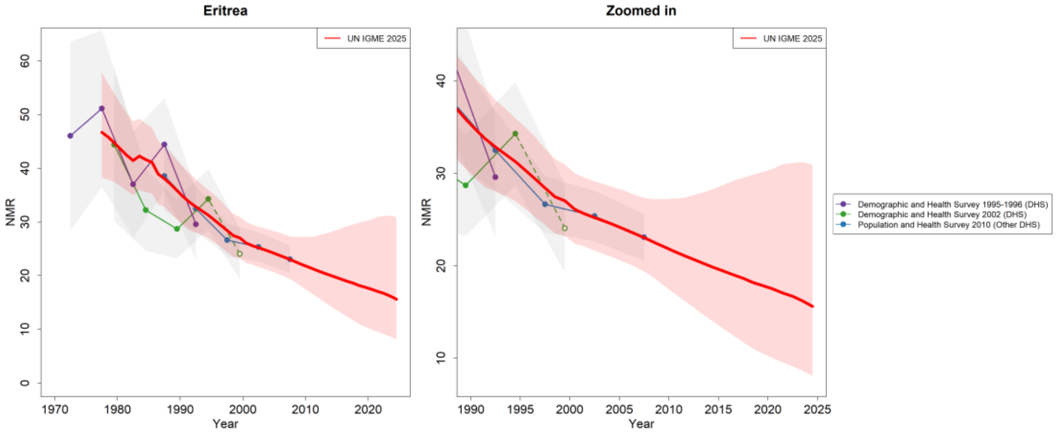

## Estonia (EST)

### Under-five mortality rate

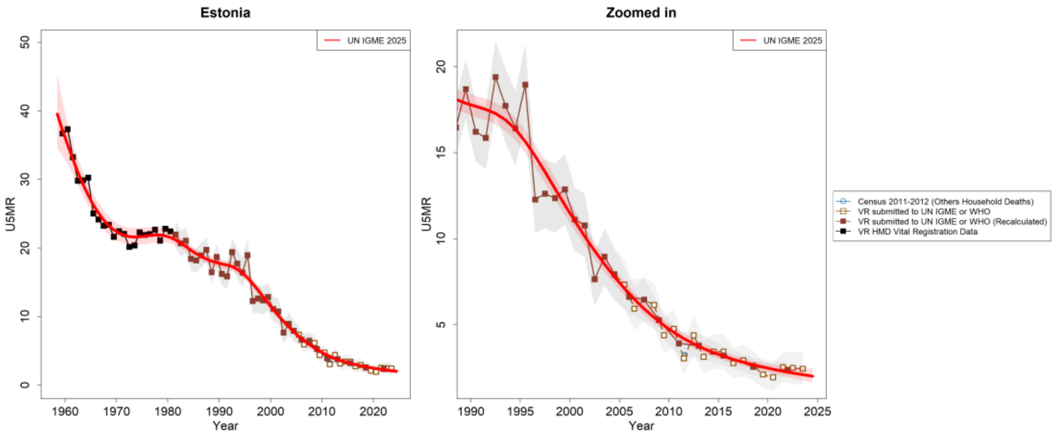


### Infant mortality rate

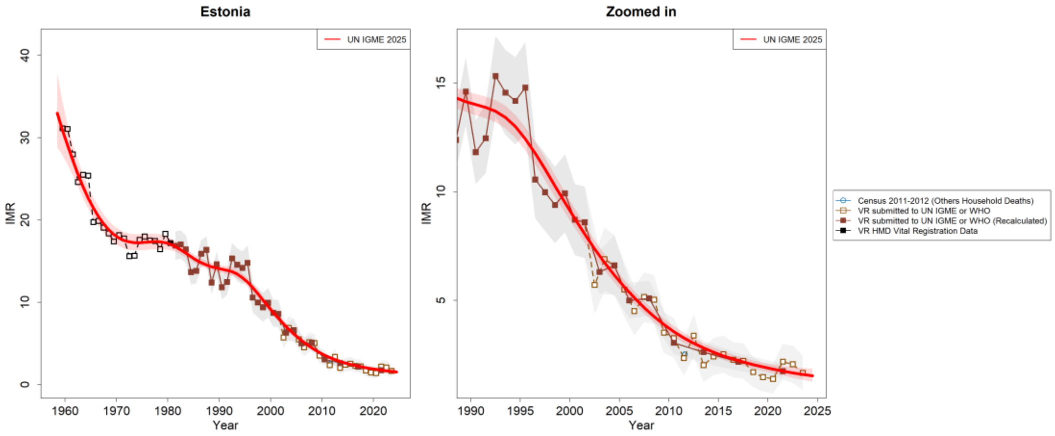


### Neonatal mortality rate

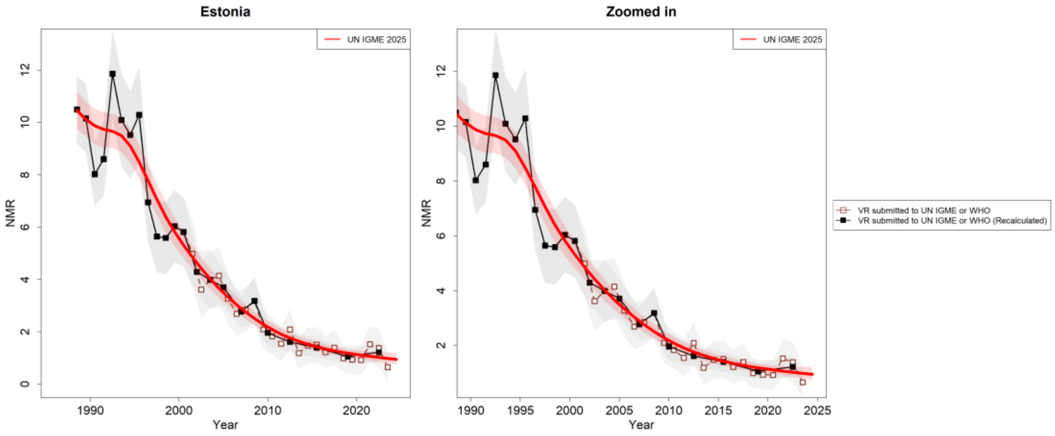

## Eswatini (SWZ)

### Under-five mortality rate

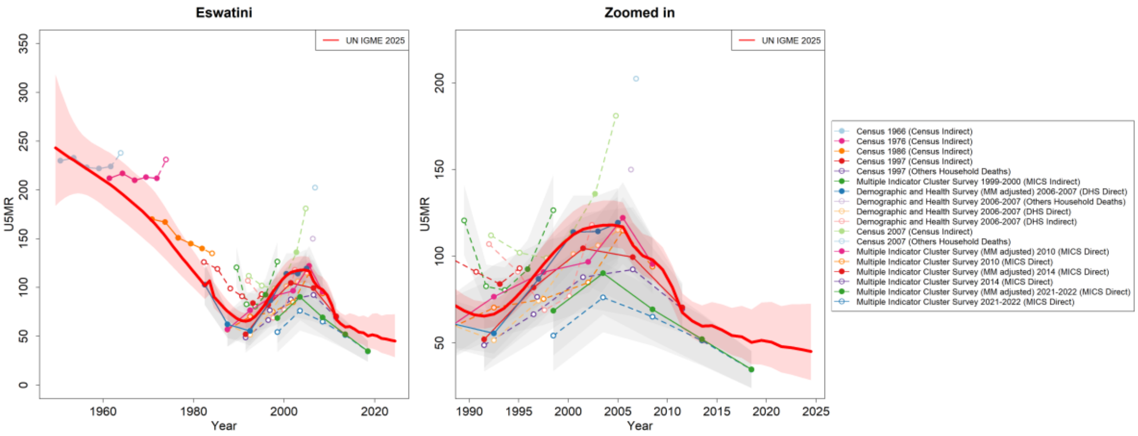


### Infant mortality rate

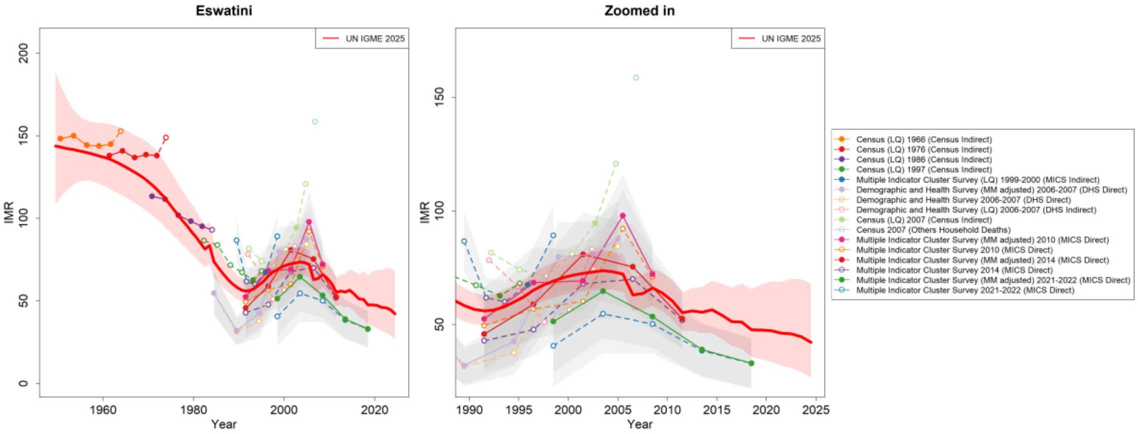


### Neonatal mortality rate

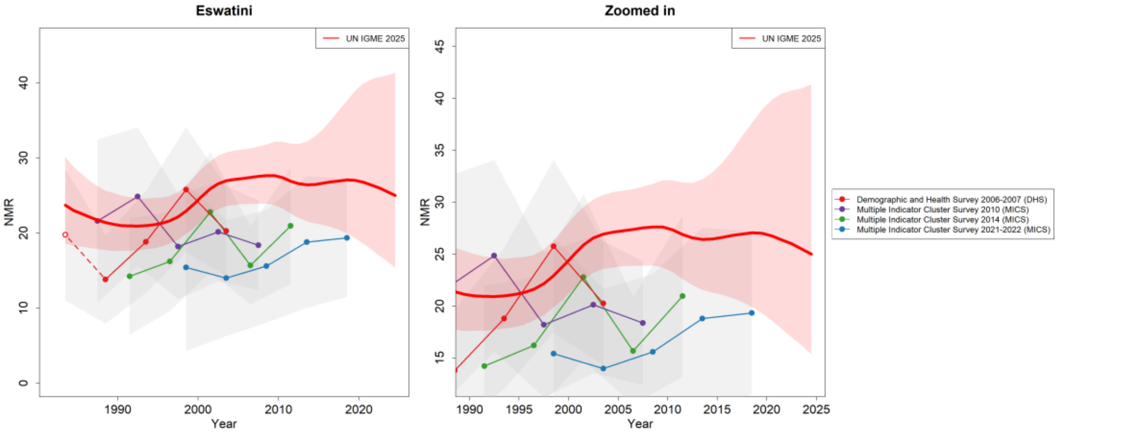

## Ethiopia (ETH)

### Under-five mortality rate

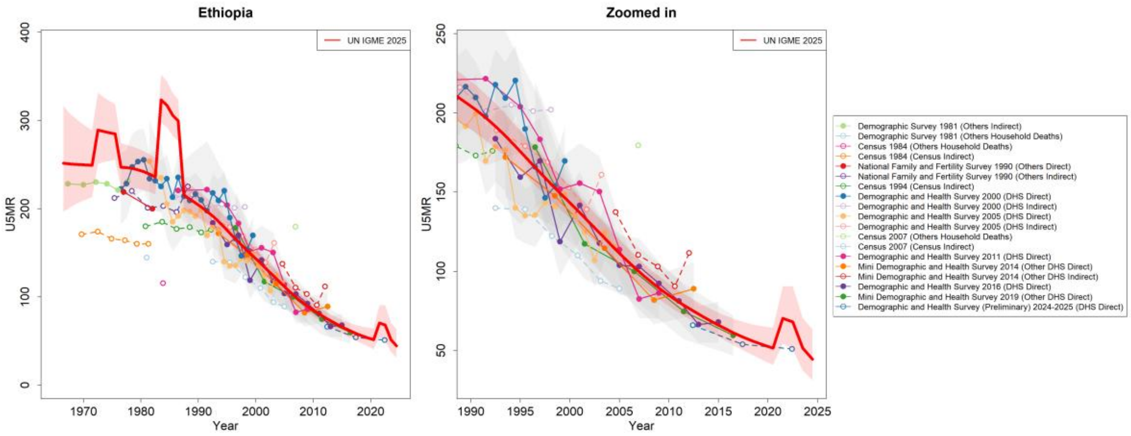


### Infant mortality rate

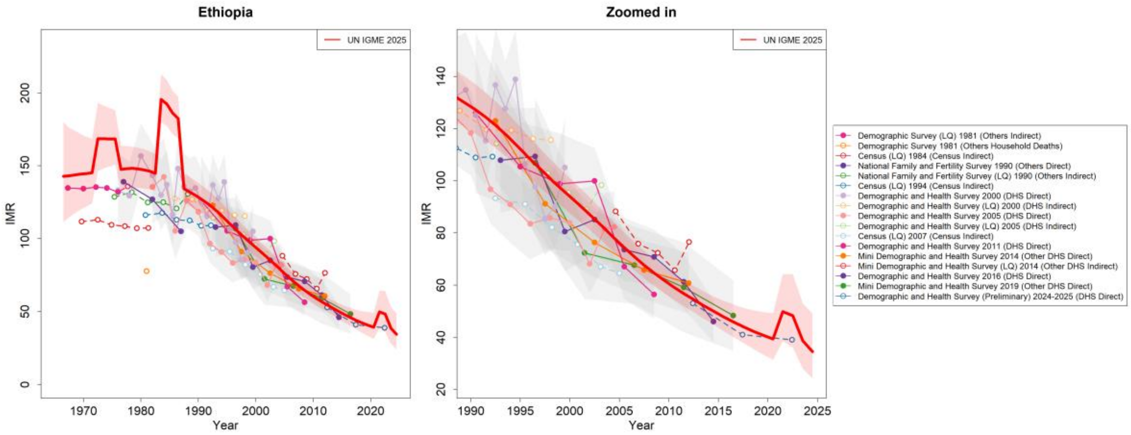


### Neonatal mortality rate

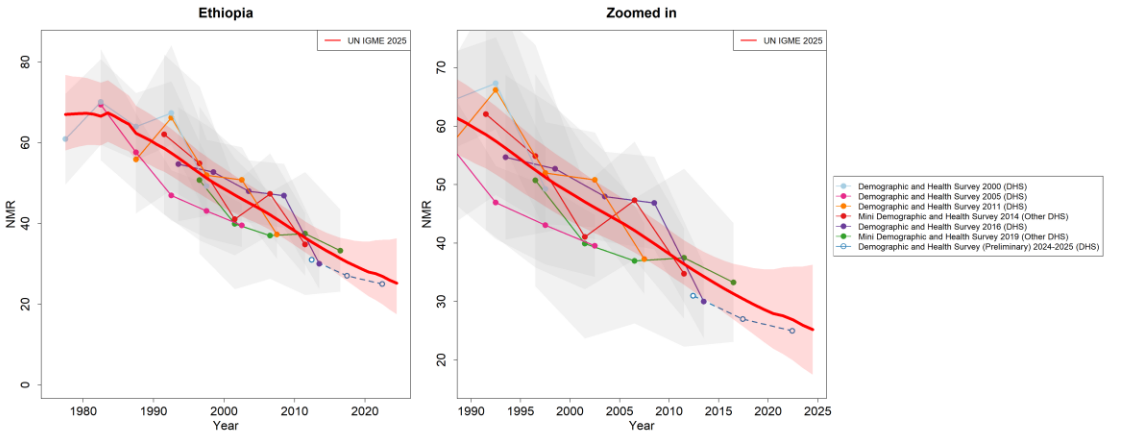

## Fiji (FJI)

### Under-five mortality rate

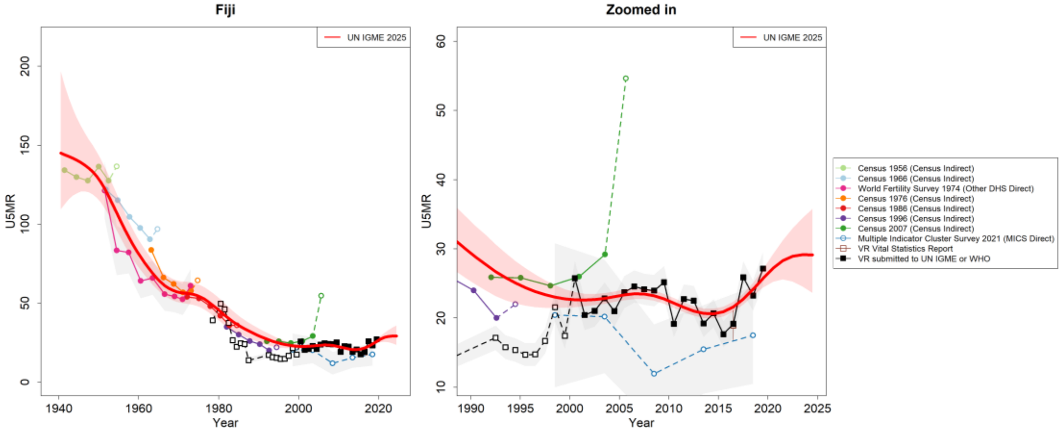


### Infant mortality rate

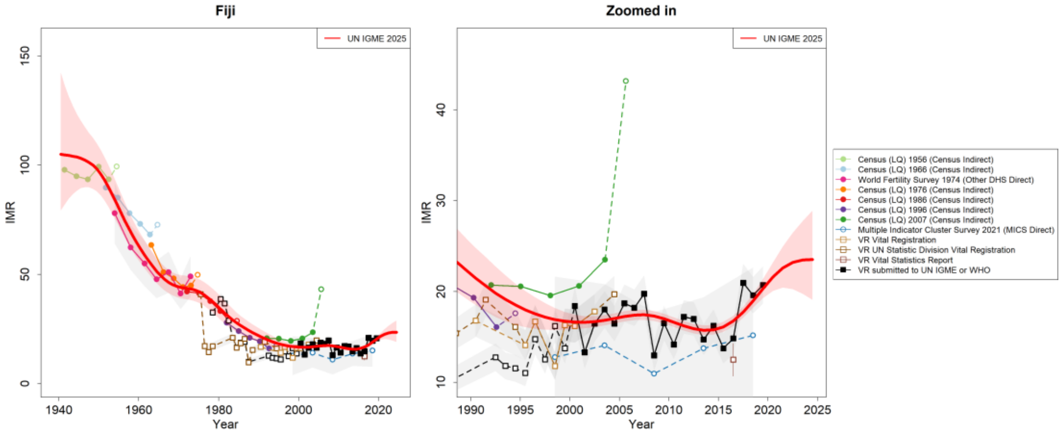


### Neonatal mortality rate

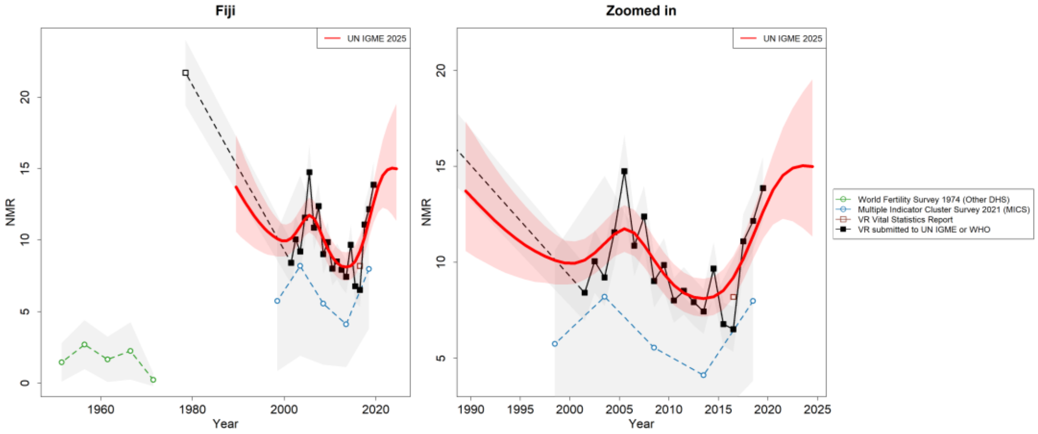

## Finland (FIN)

### Under-five mortality rate

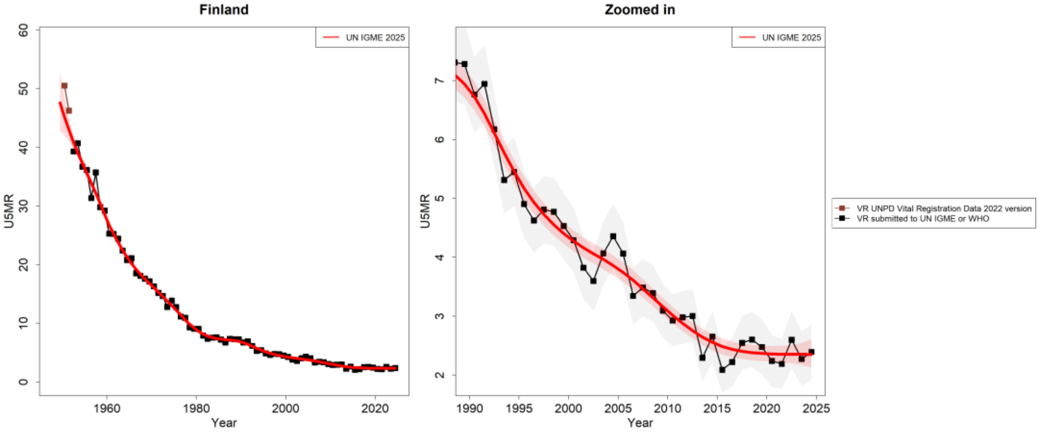


### Infant mortality rate

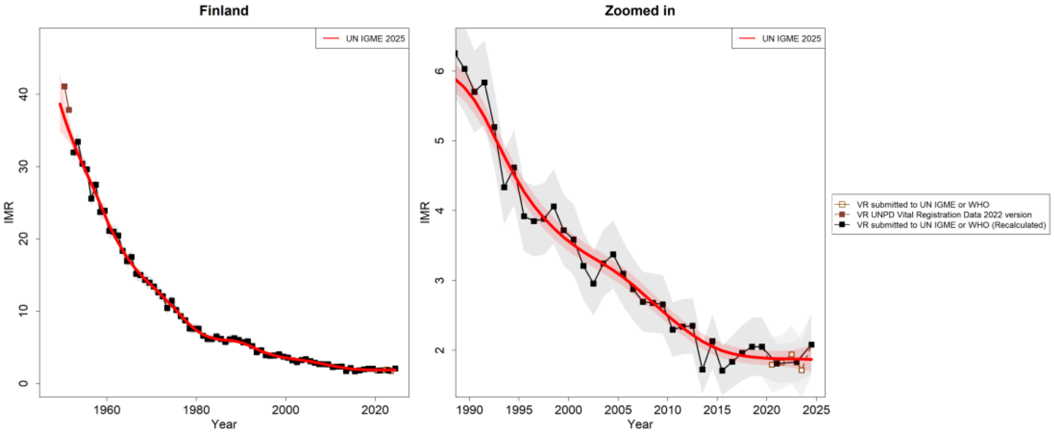


### Neonatal mortality rate

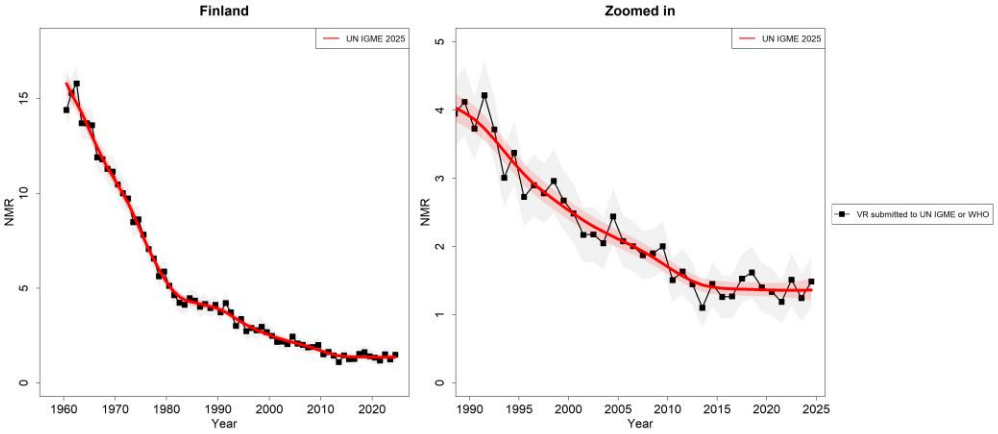

## France (FRA)

### Under-five mortality rate

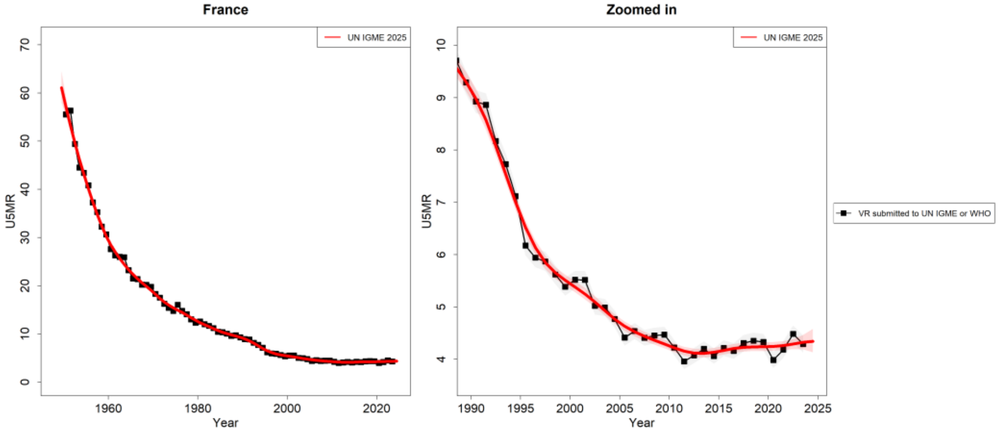


### Infant mortality rate

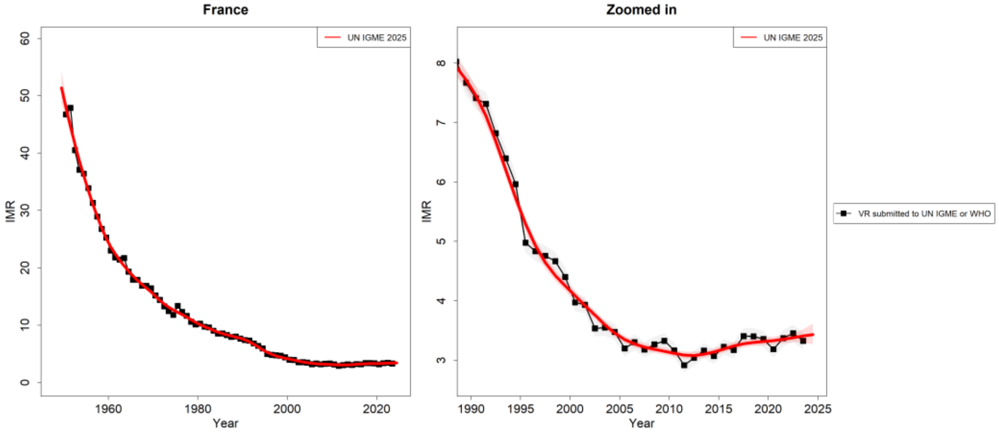


### Neonatal mortality rate

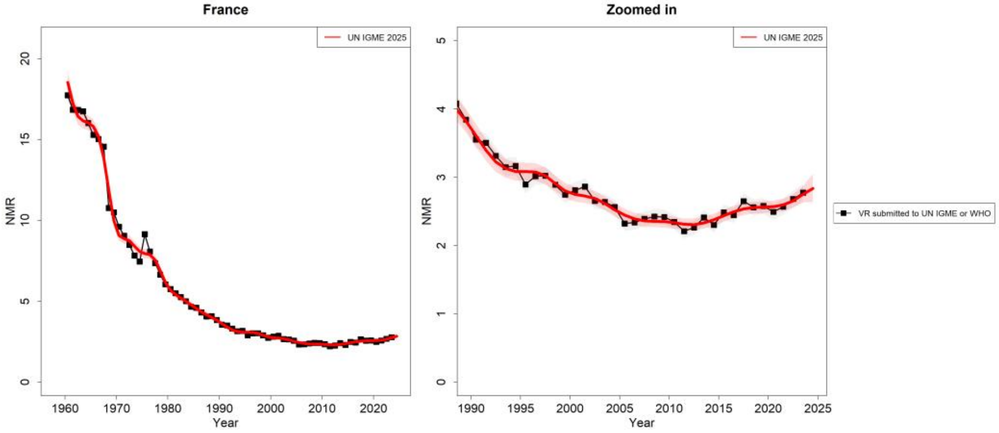

## Gabon (GAB)

### Under-five mortality rate

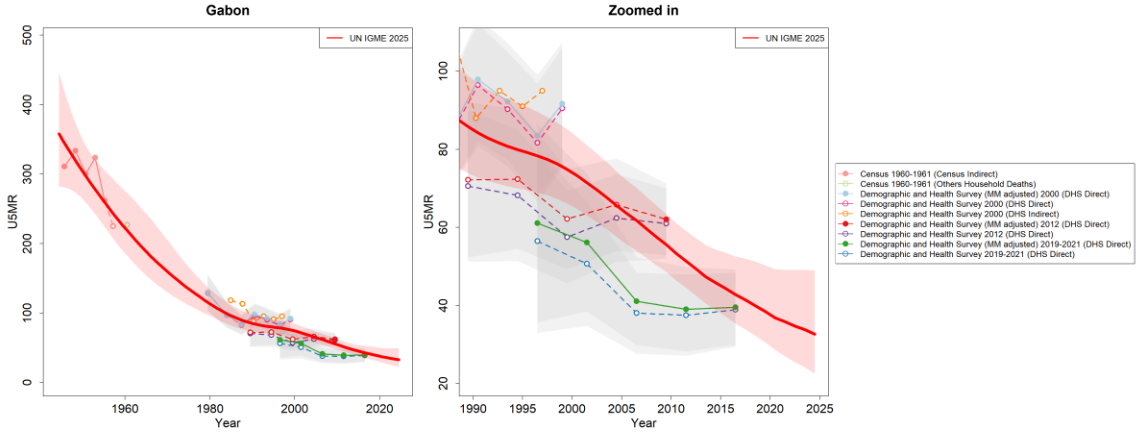


### Infant mortality rate

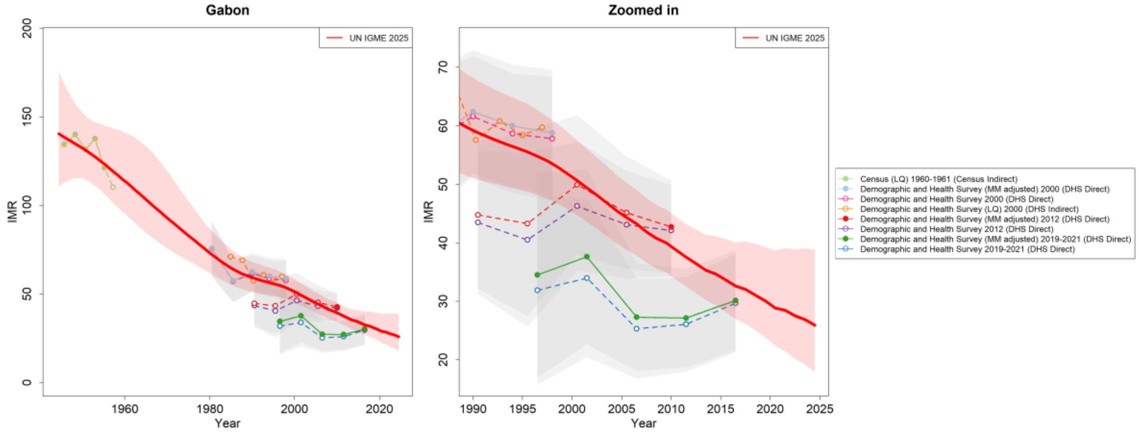


### Neonatal mortality rate

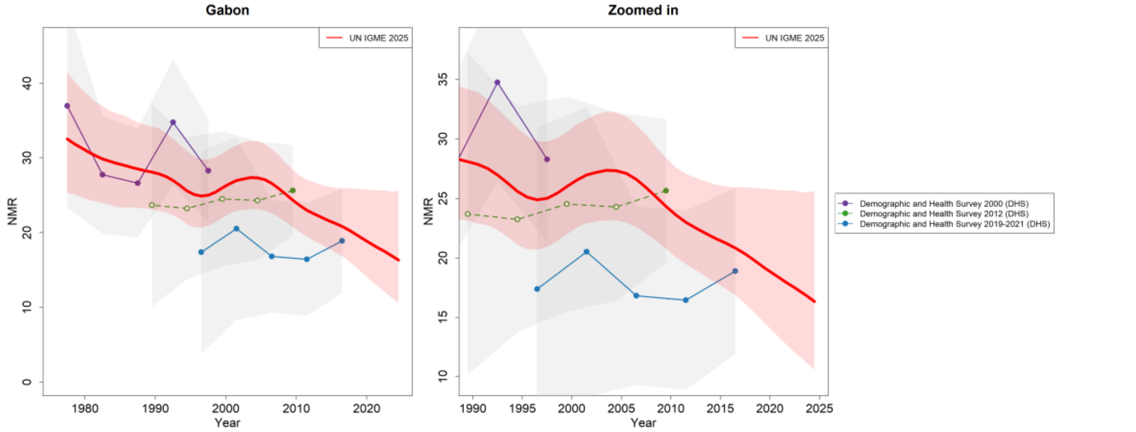

## Gambia (GMB)

### Under-five mortality rate

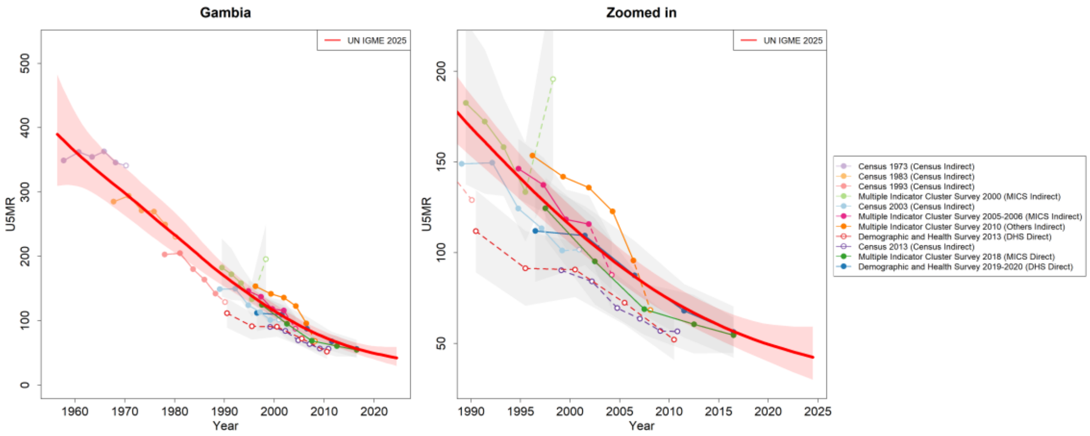


### Infant mortality rate

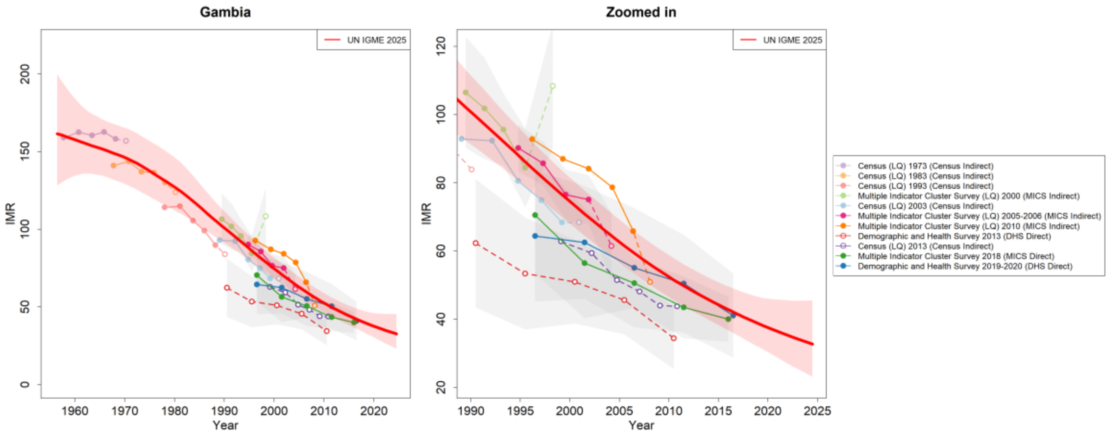


### Neonatal mortality rate

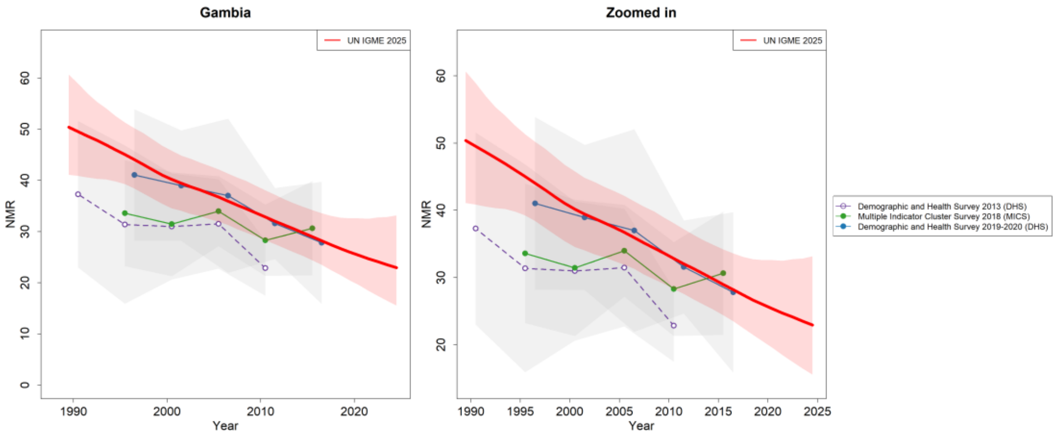

## Georgia (GEO)

### Under-five mortality rate

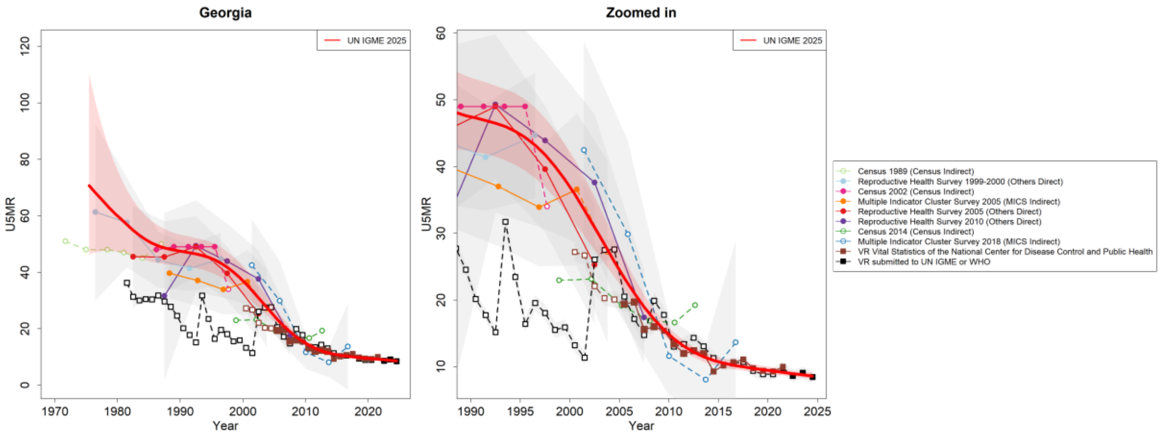


### Infant mortality rate

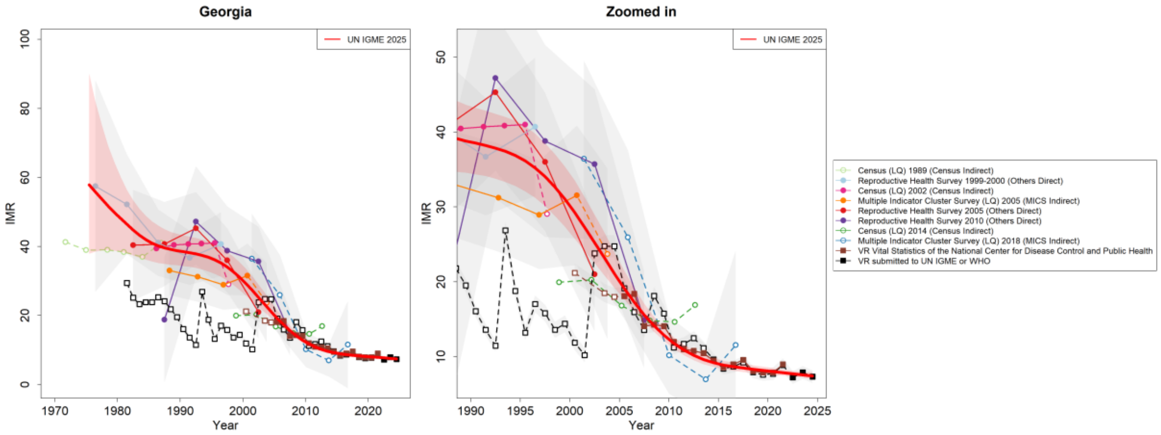


### Neonatal mortality rate

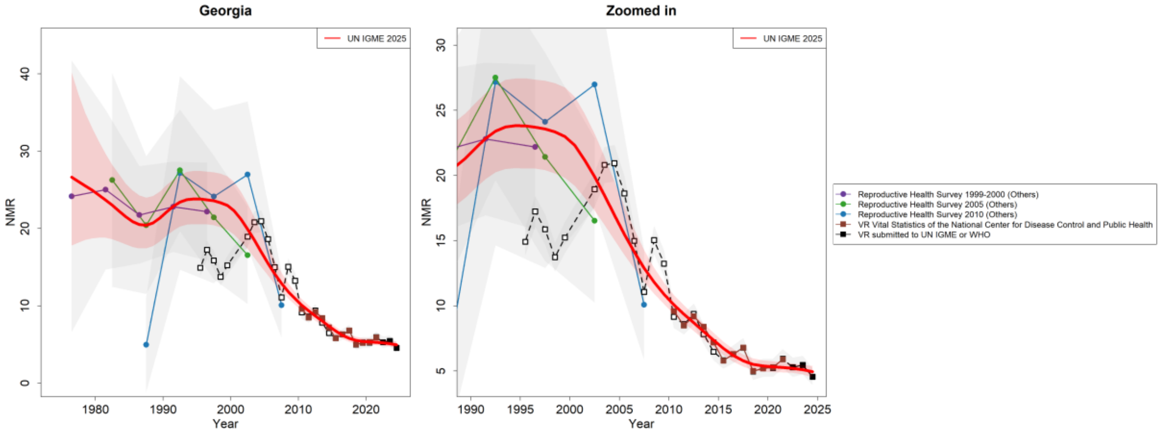

## Germany (DEU)

### Under-five mortality rate

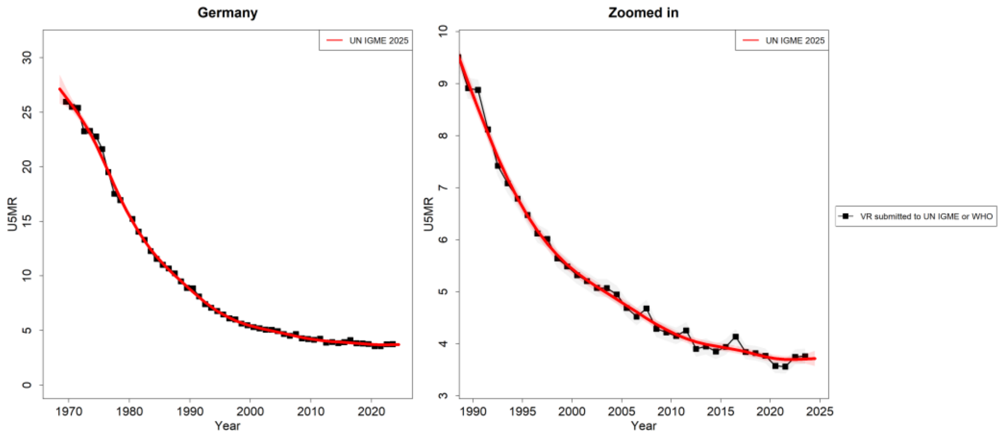


### Infant mortality rate

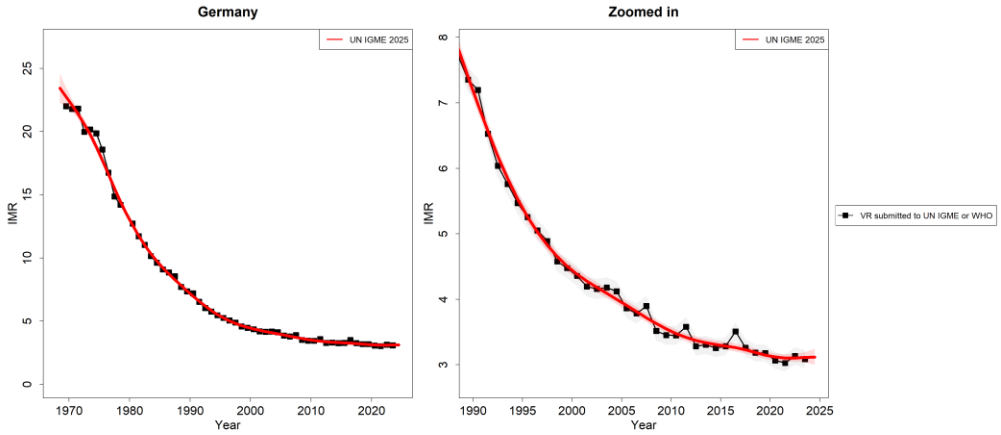


### Neonatal mortality rate

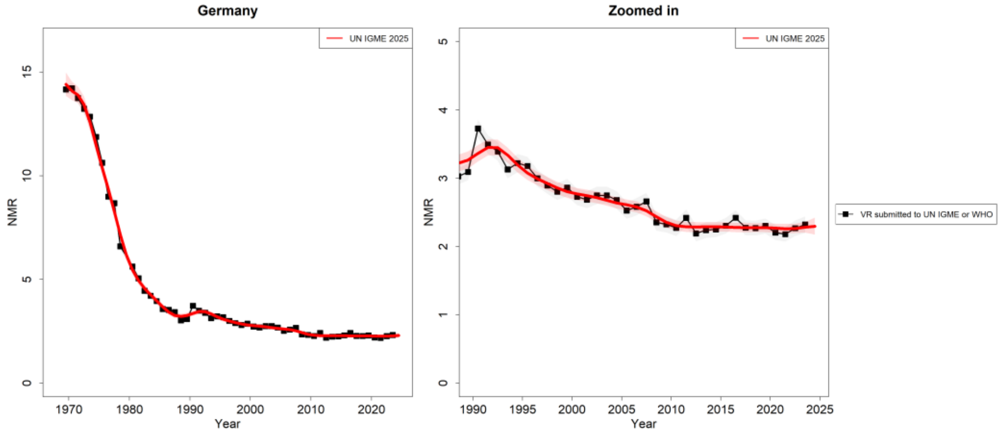

## Ghana (GHA)

### Under-five mortality rate

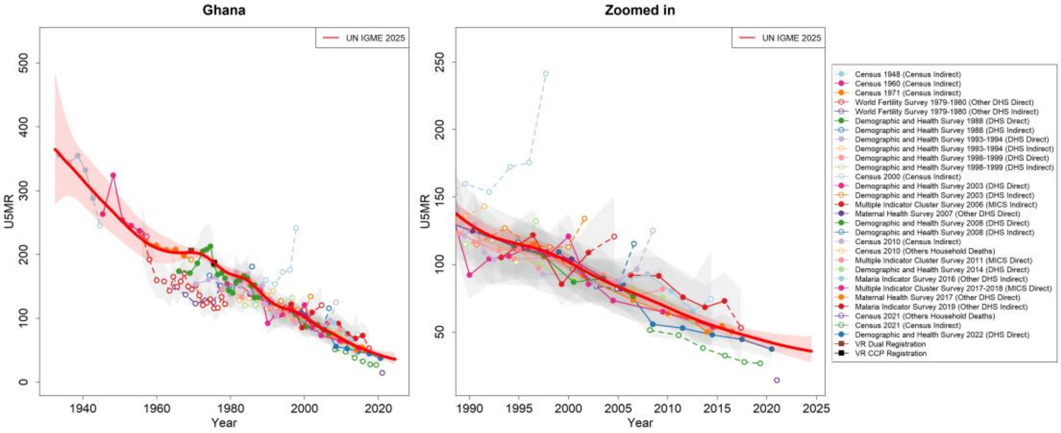


### Infant mortality rate

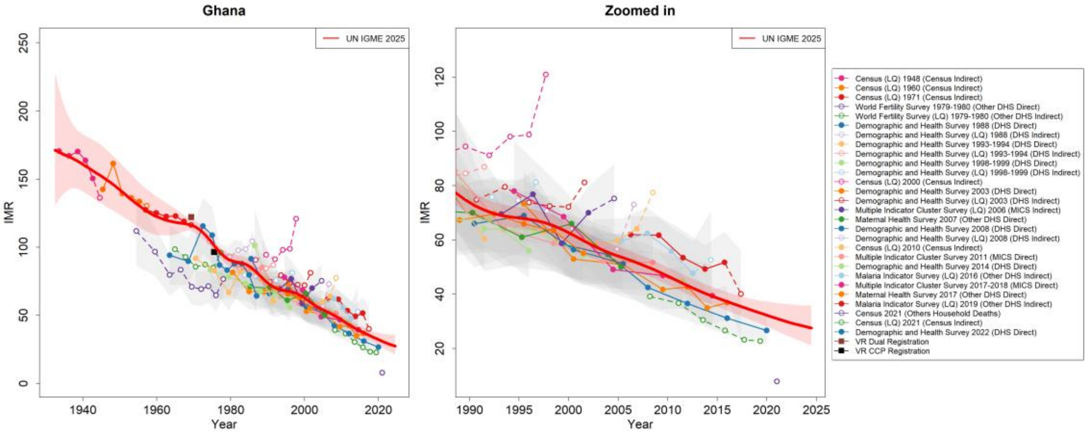


### Neonatal mortality rate

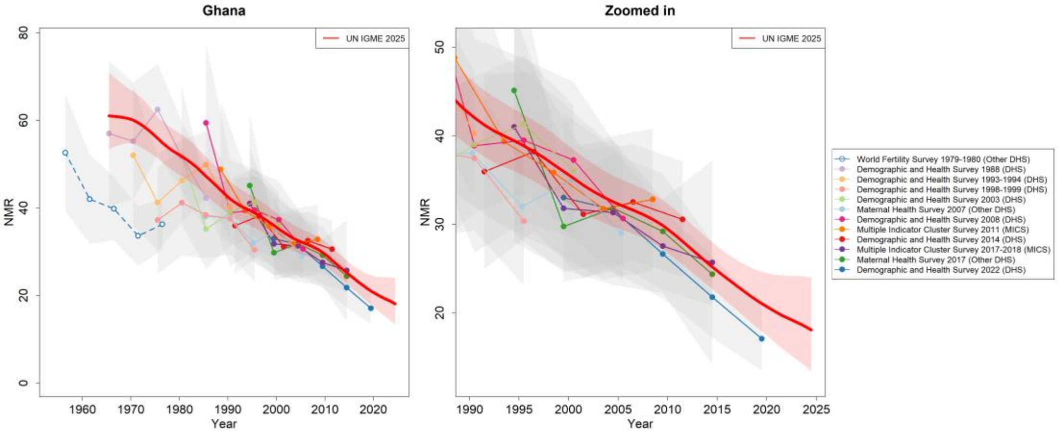

## Greece (GRC)

### Under-five mortality rate

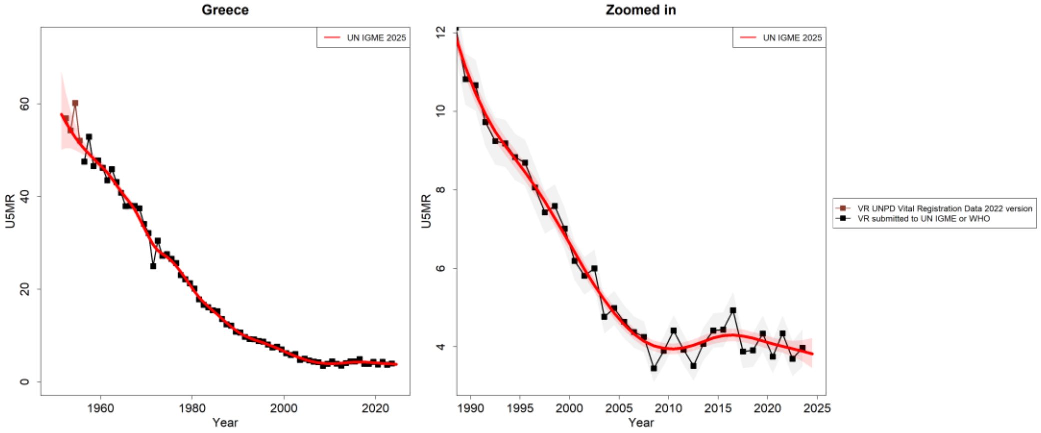


### Infant mortality rate

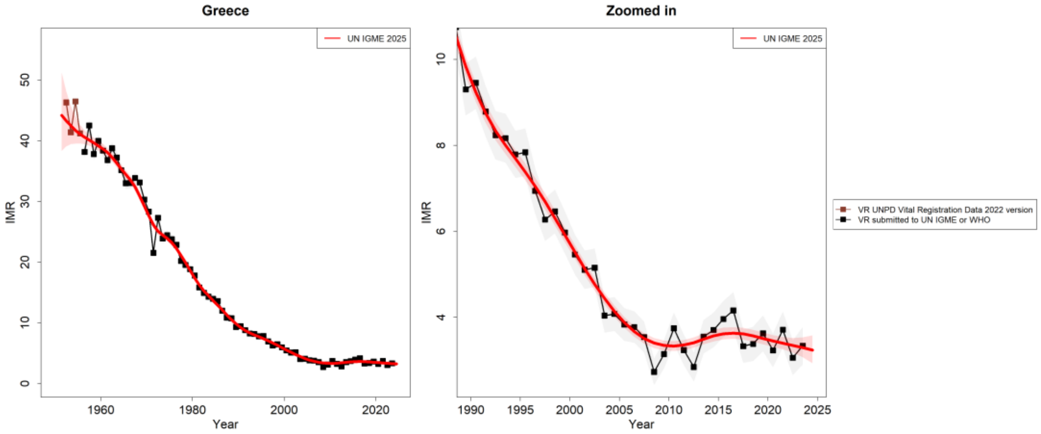


### Neonatal mortality rate

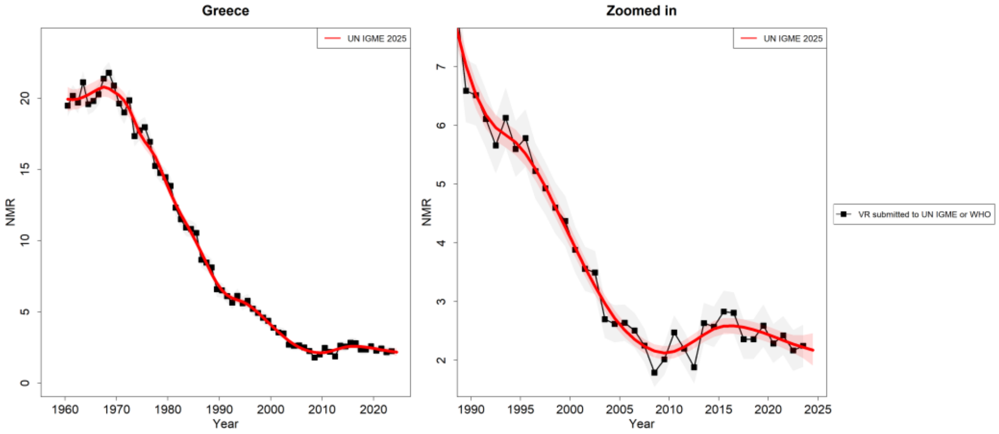

## Grenada (GRD)

### Under-five mortality rate

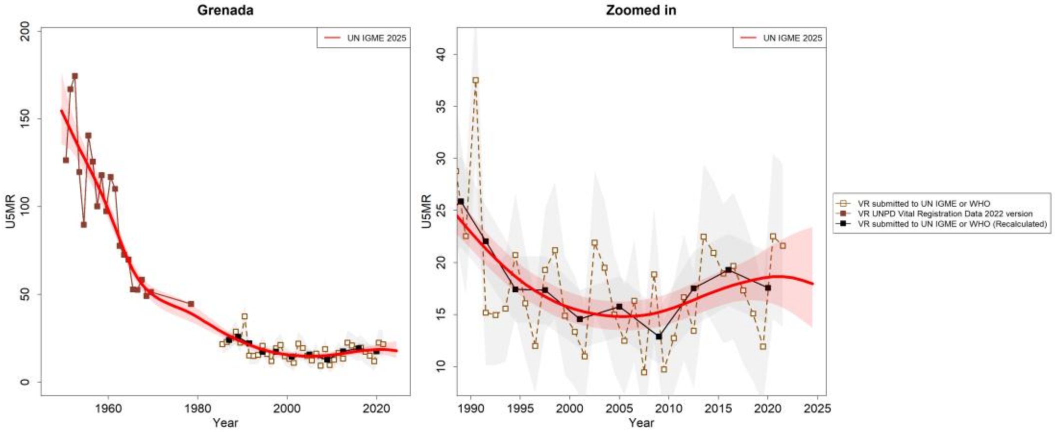


### Infant mortality rate

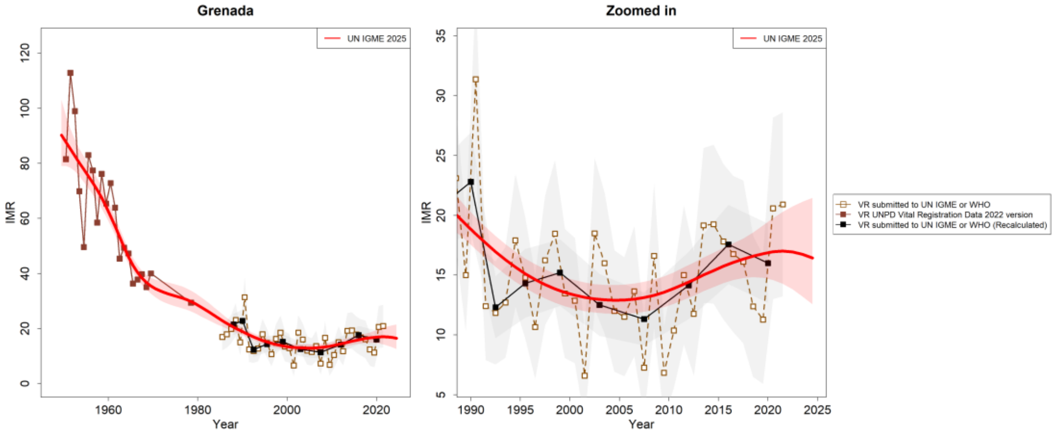


### Neonatal mortality rate

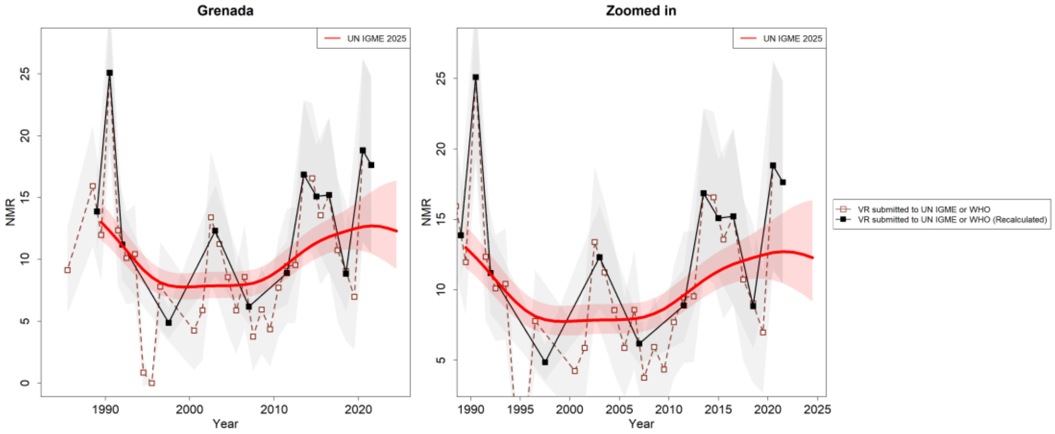

## Guatemala (GTM)

### Under-five mortality rate

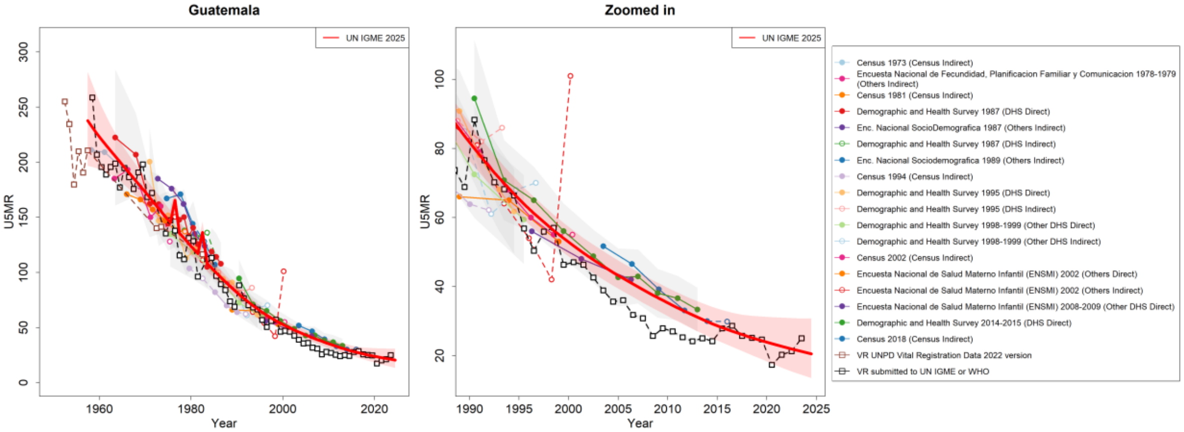


### Infant mortality rate

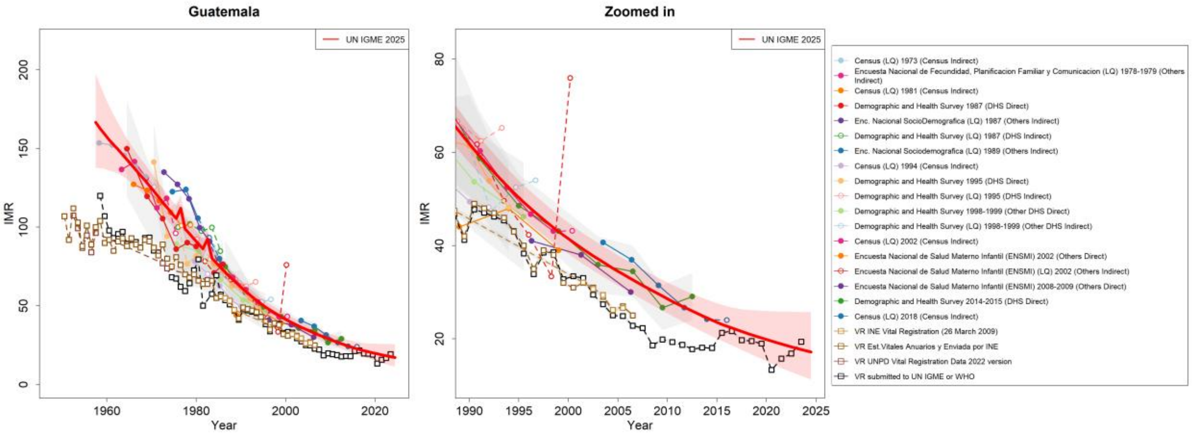


### Neonatal mortality rate

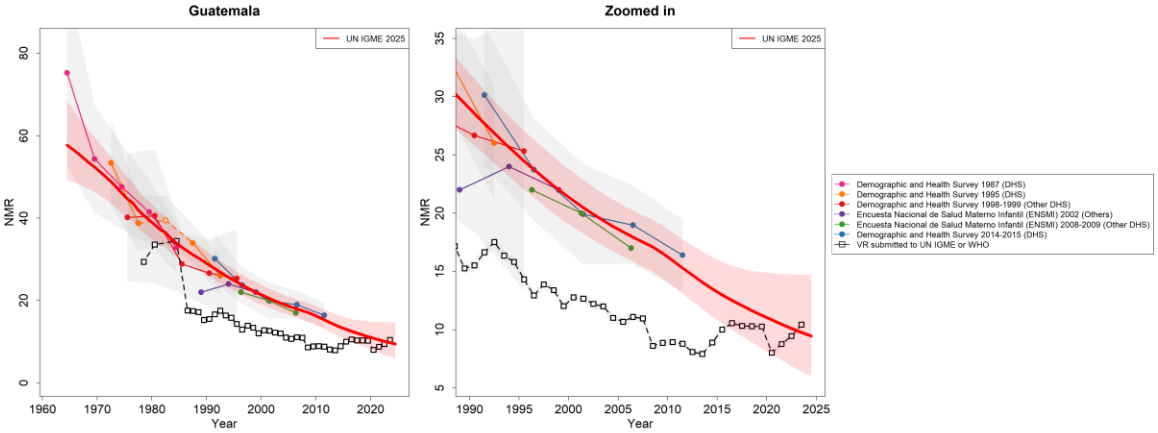

## Guinea (GIN)

### Under-five mortality rate

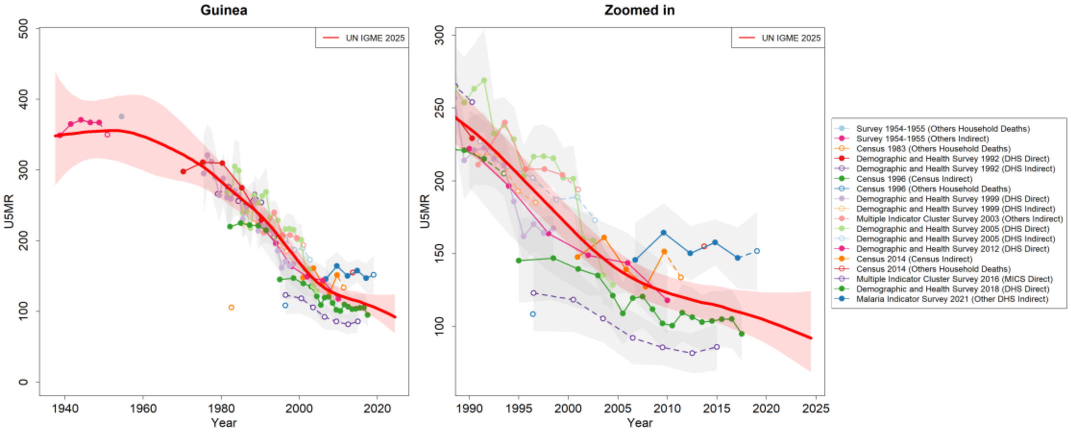


### Infant mortality rate

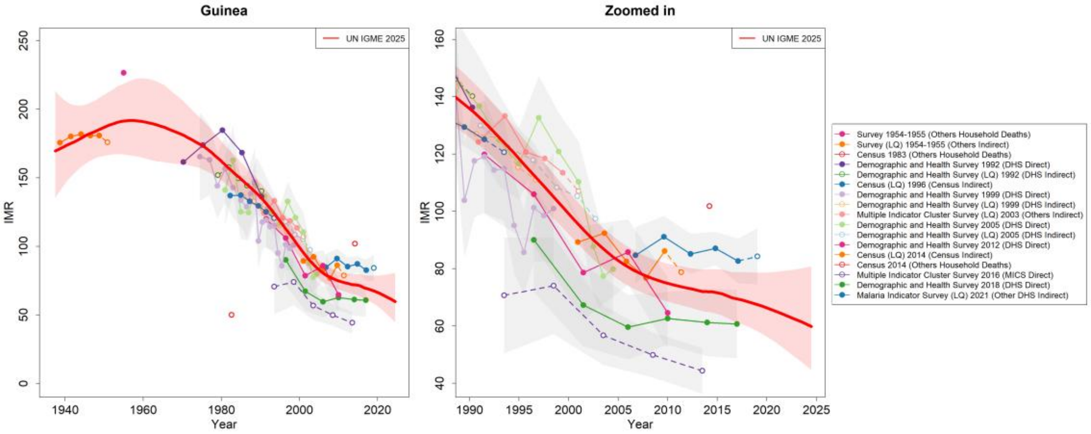


### Neonatal mortality rate

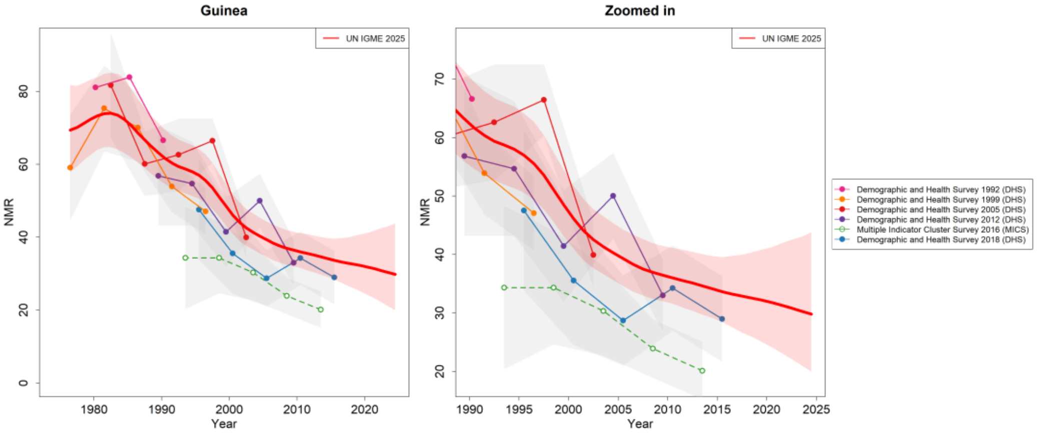

## Guinea-Bissau (GNB)

### Under-five mortality rate

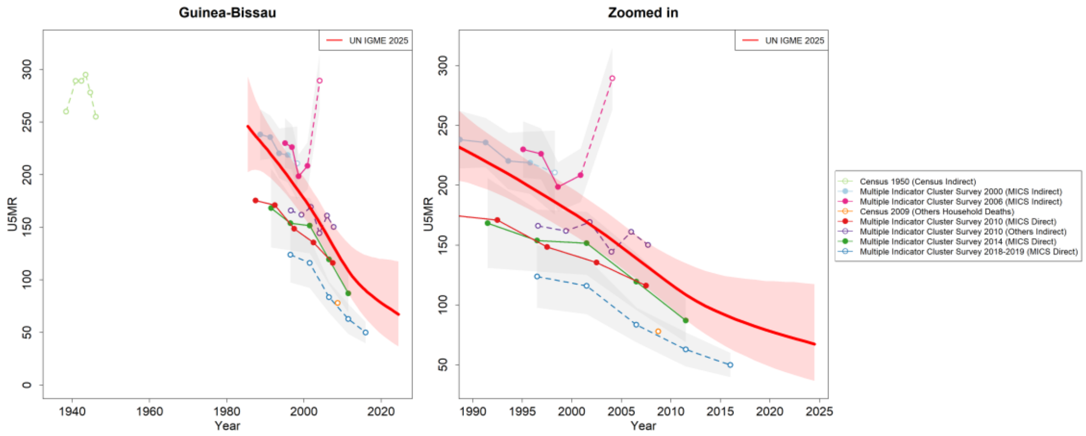


### Infant mortality rate

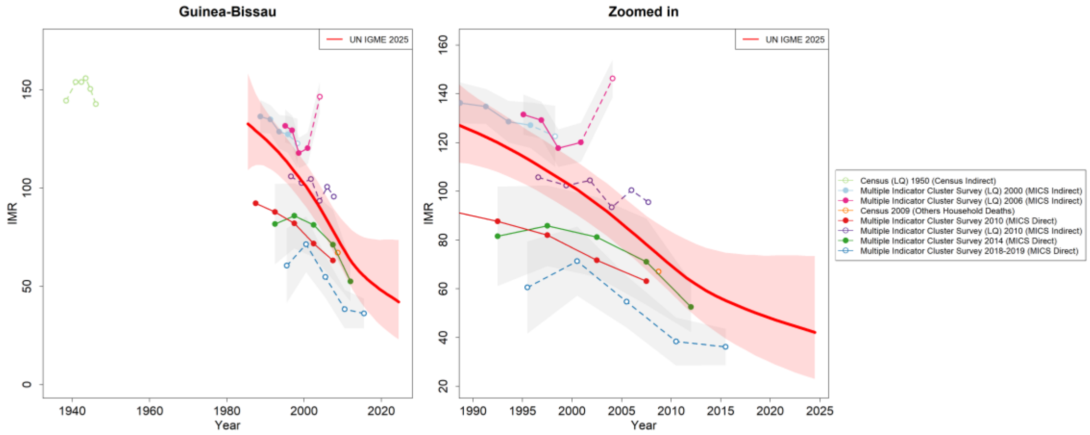


### Neonatal mortality rate

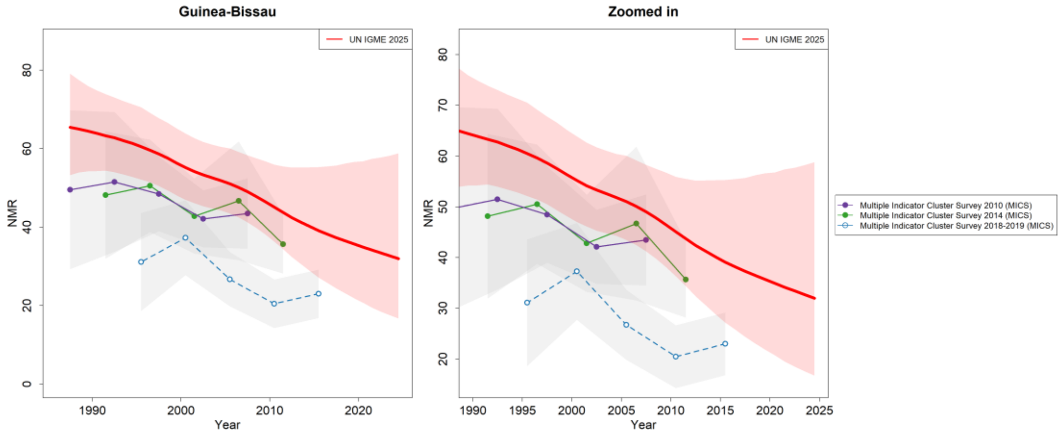

# Guyana (GUY)

## Under-five mortality rate

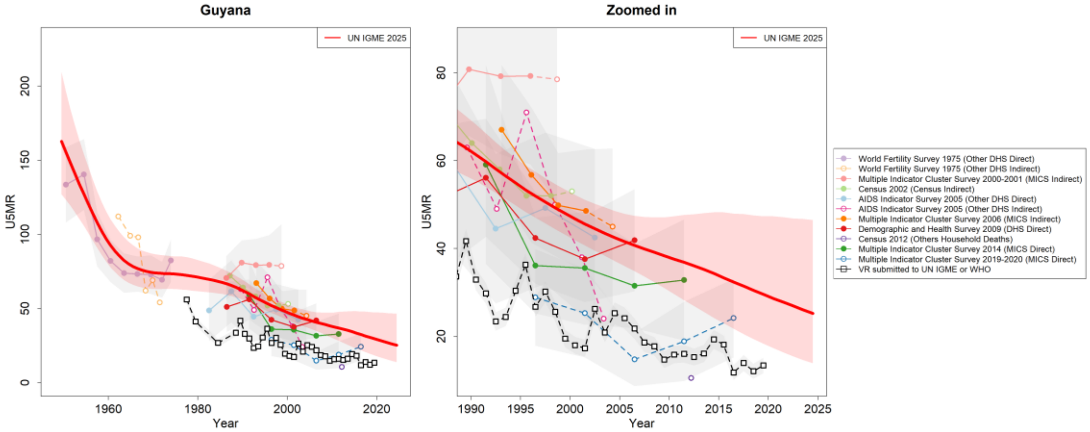


## Infant mortality rate

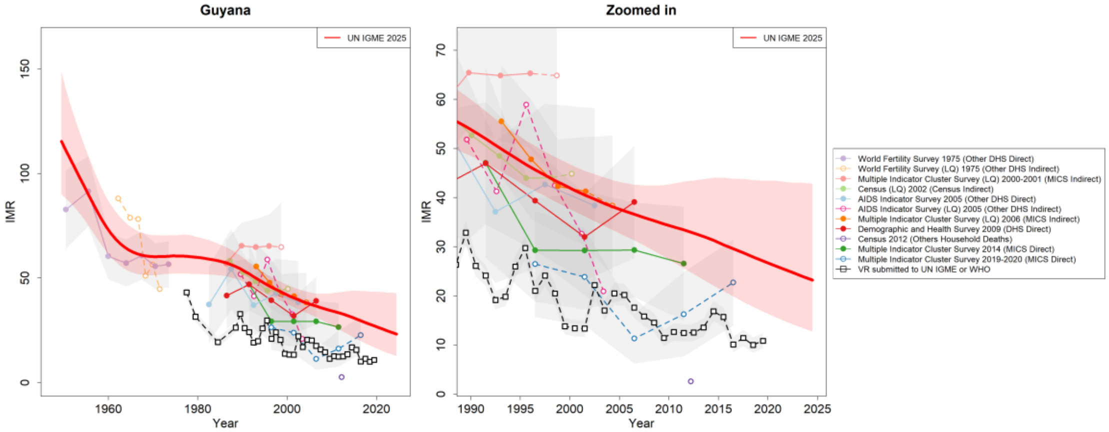


## Neonatal mortality rate

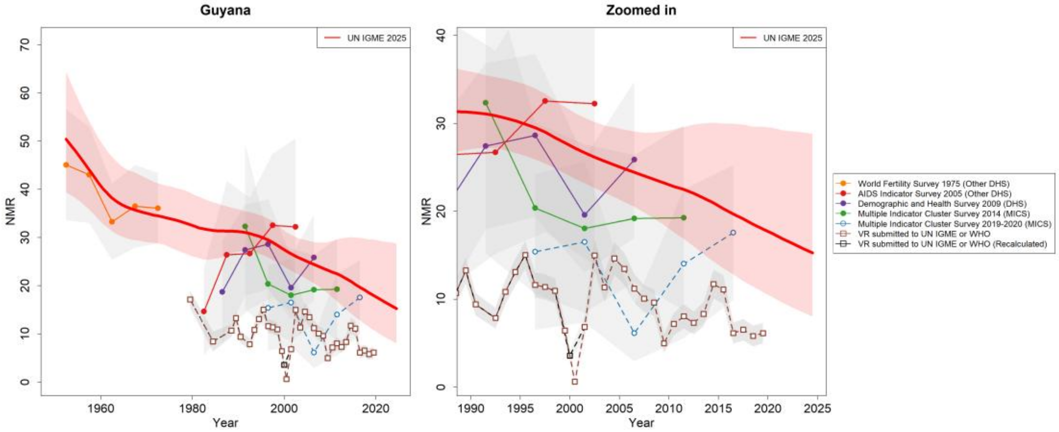

## Haiti (HTI)

### Under-five mortality rate

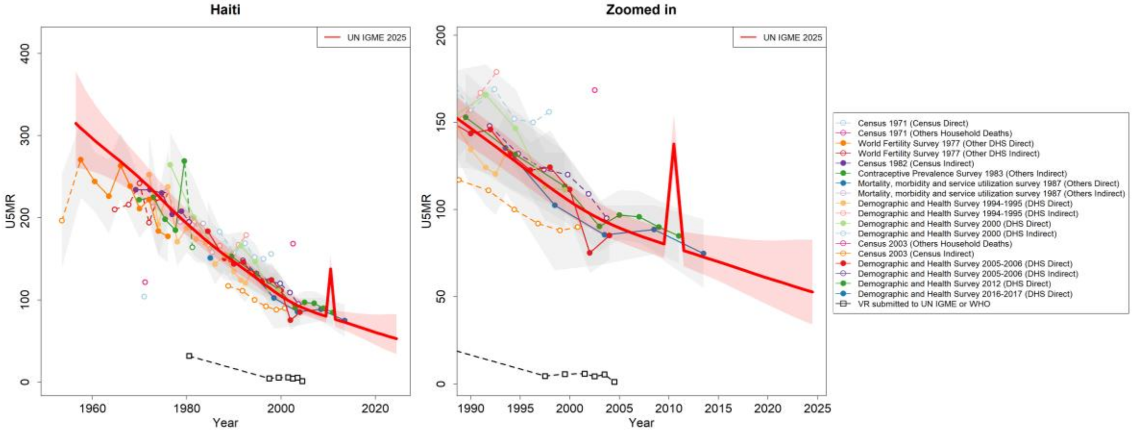


### Infant mortality rate

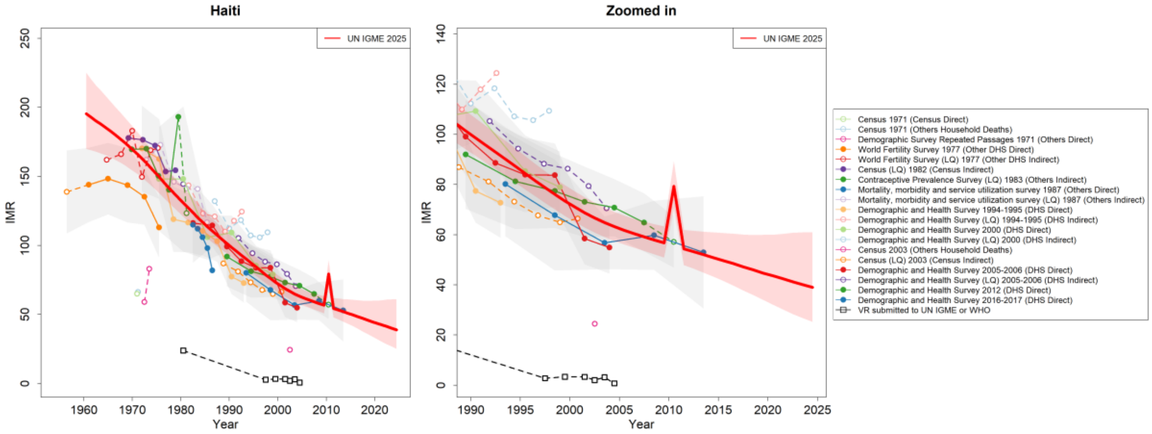


### Neonatal mortality rate

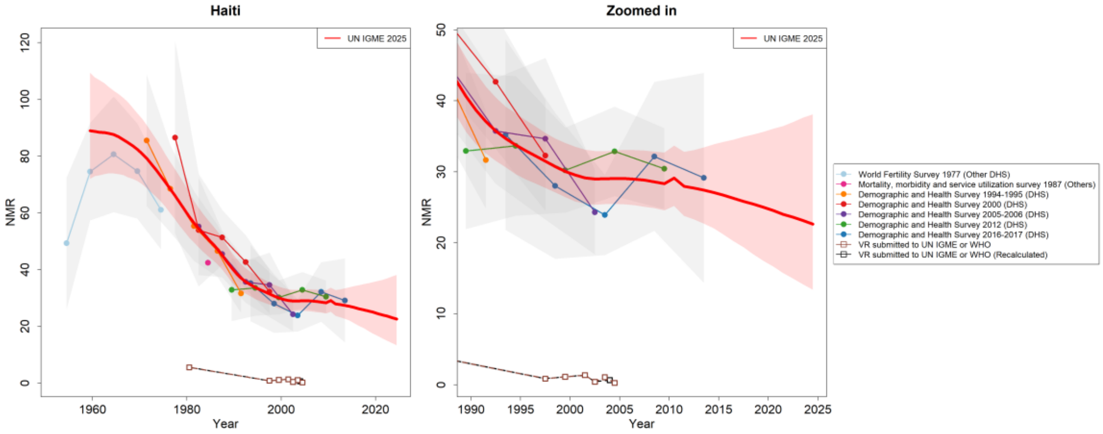

## Honduras (HND)

### Under-five mortality rate

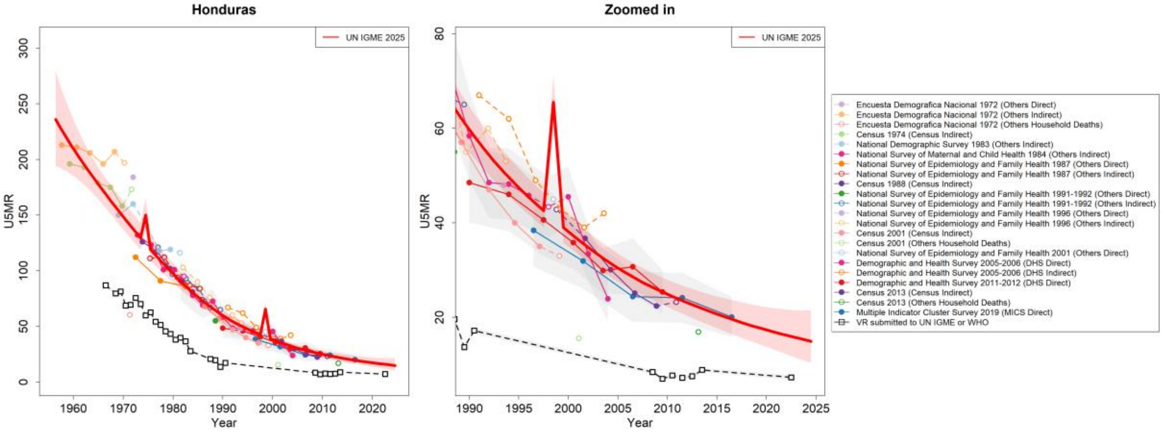


### Infant mortality rate

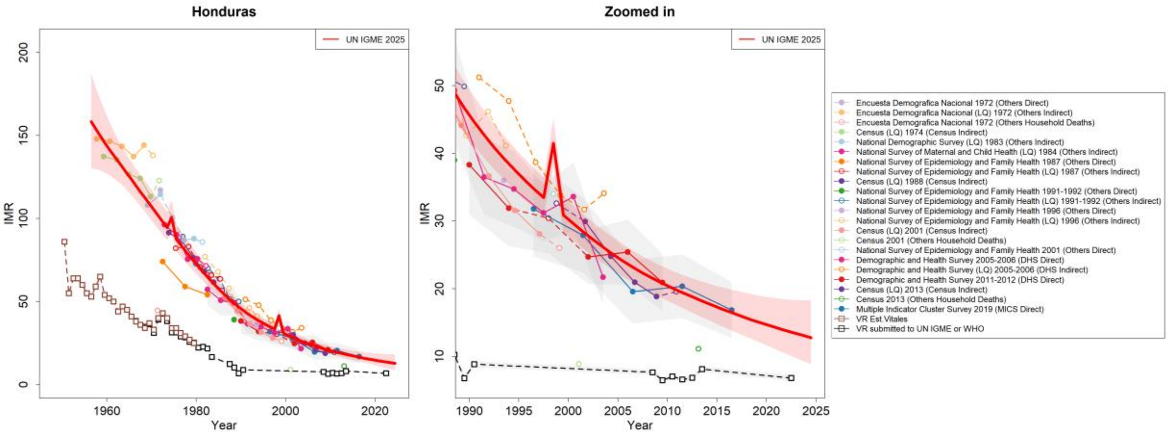


### Neonatal mortality rate

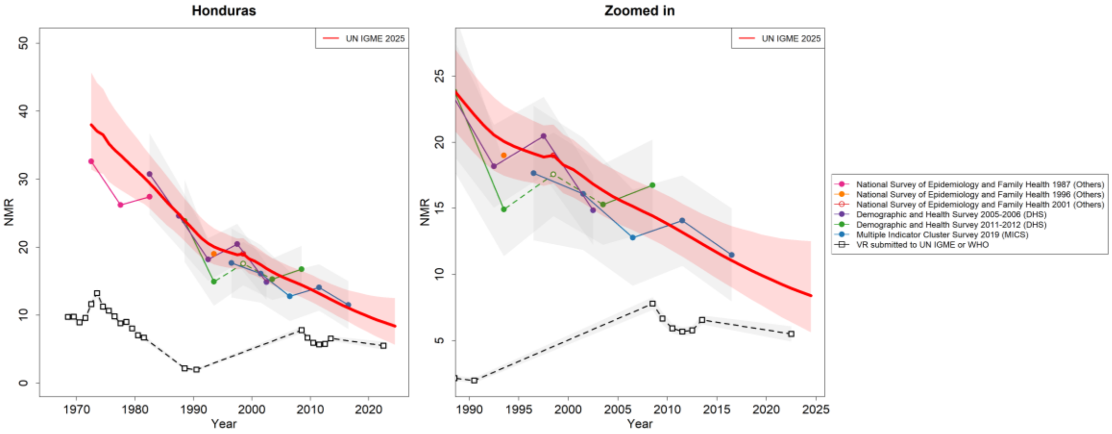

## Hungary (HUN)

### Under-five mortality rate

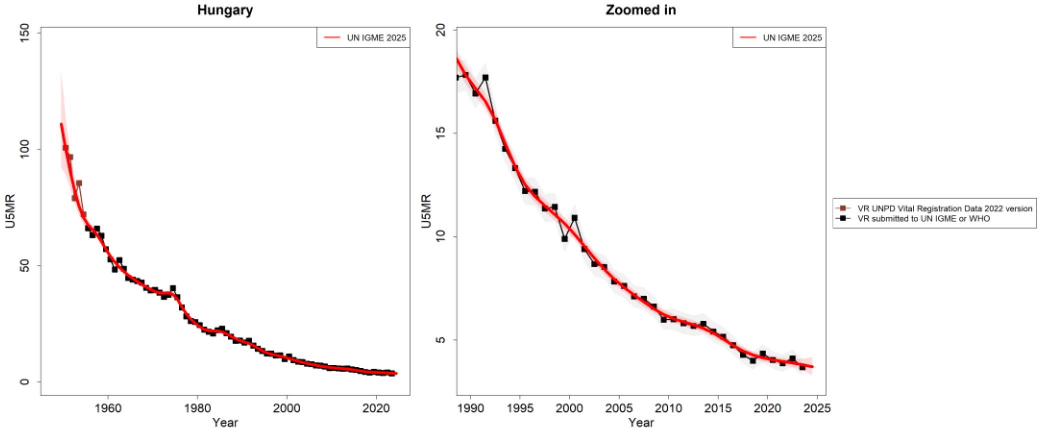


### Infant mortality rate

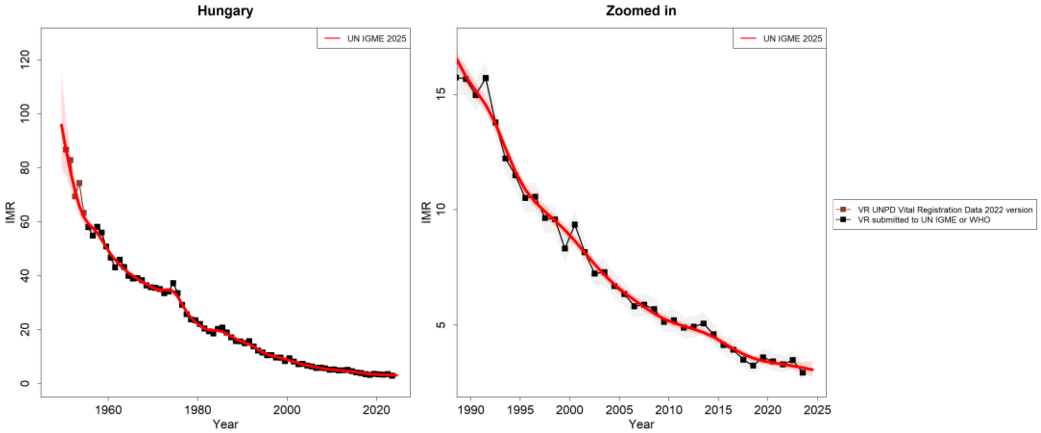


### Neonatal mortality rate

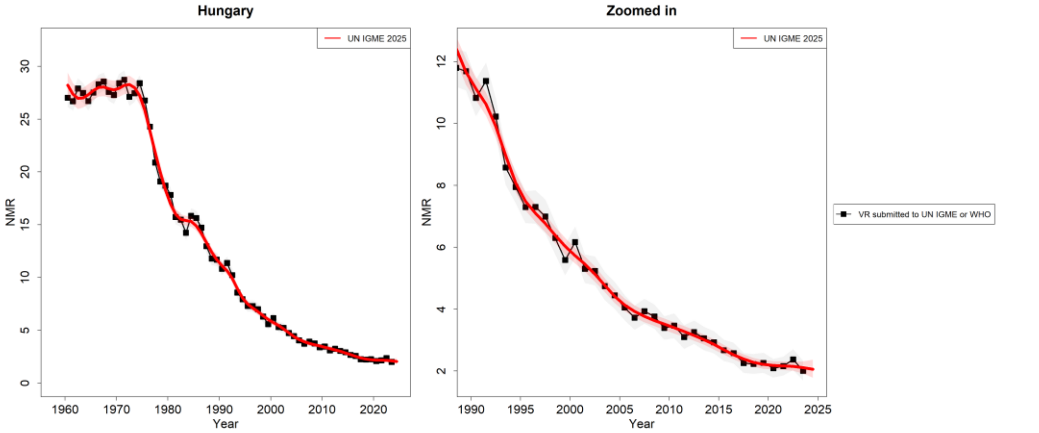

## Iceland (ISL)

### Under-five mortality rate

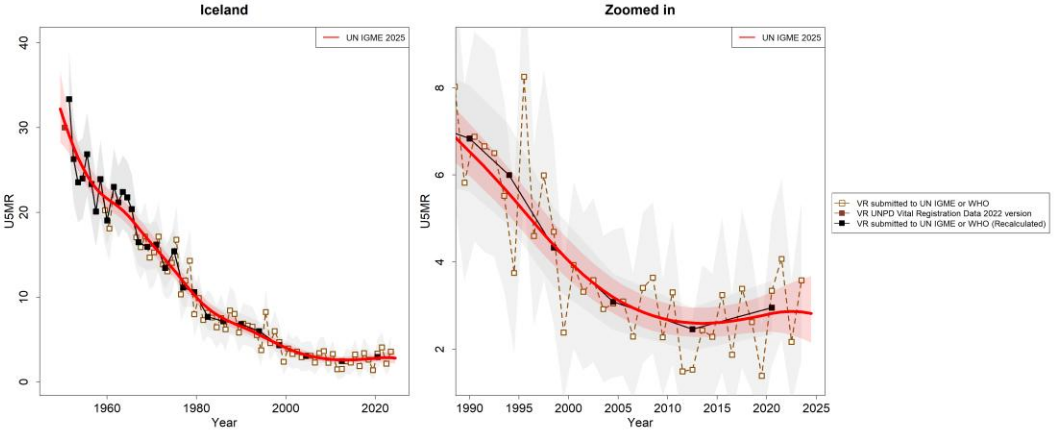


### Infant mortality rate

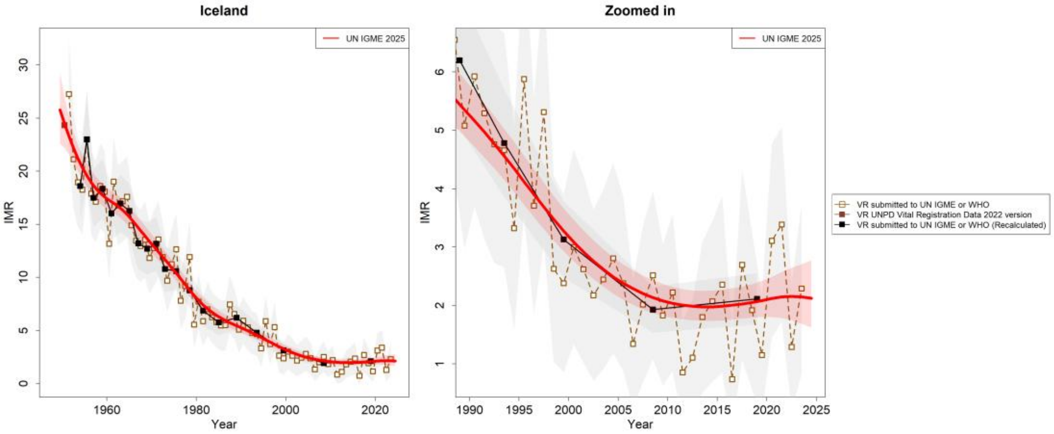


### Neonatal mortality rate

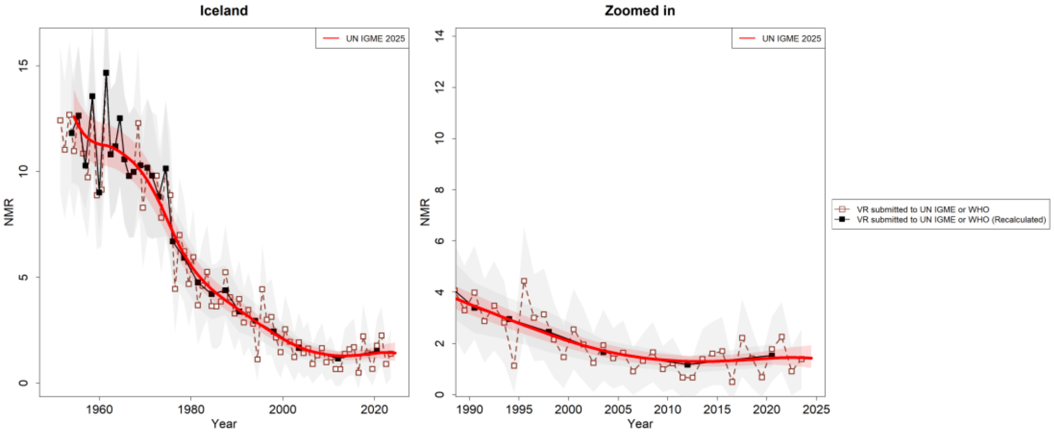

## India (IND)

### Under-five mortality rate

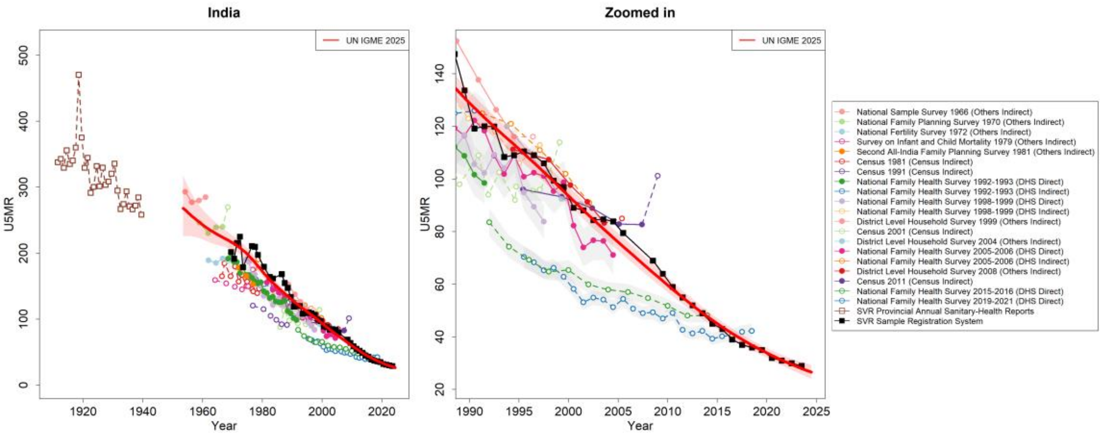


### Infant mortality rate

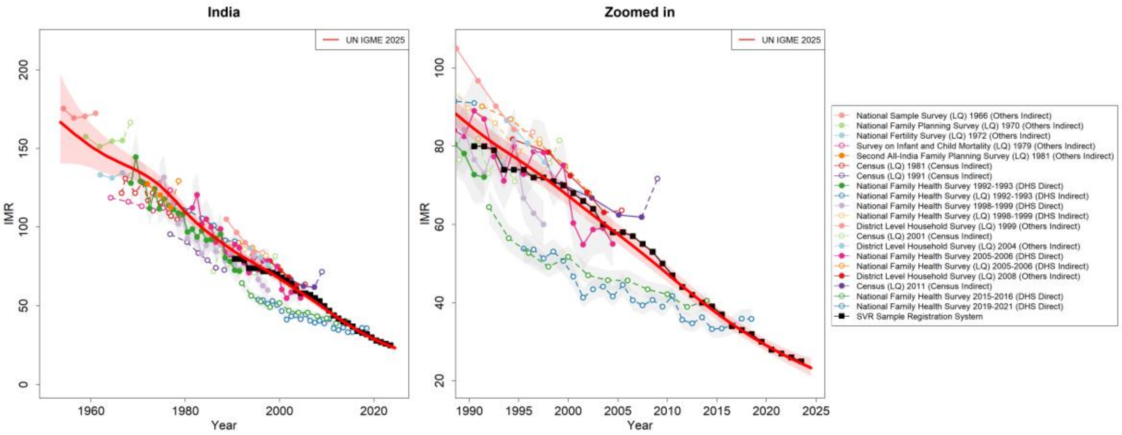


### Neonatal mortality rate

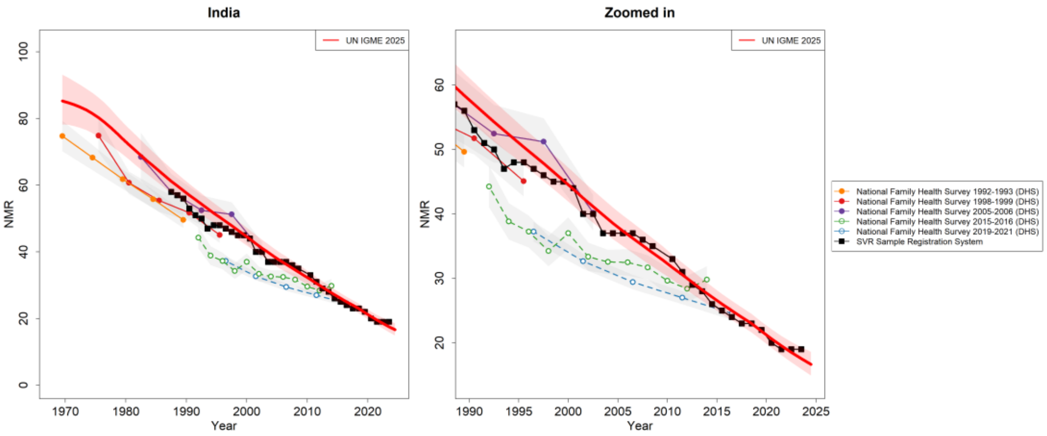

## Indonesia (IDN)

### Under-five mortality rate

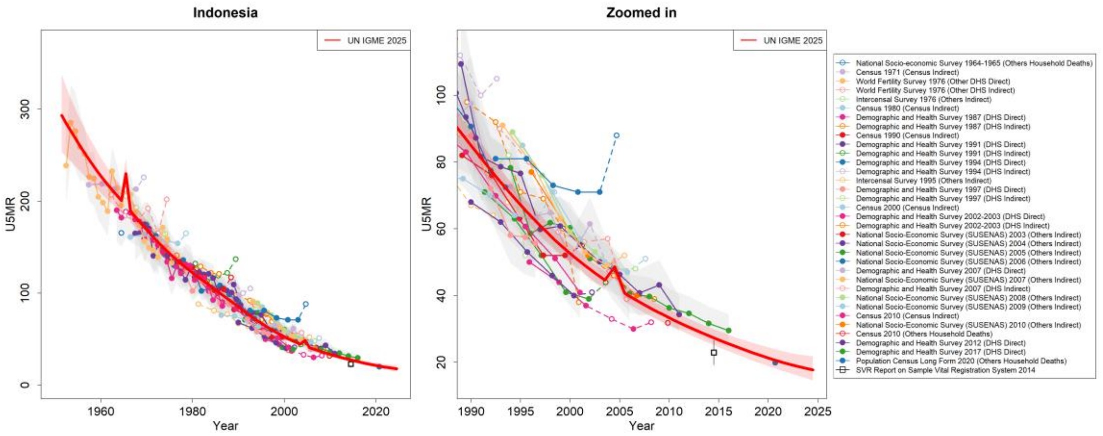


### Infant mortality rate

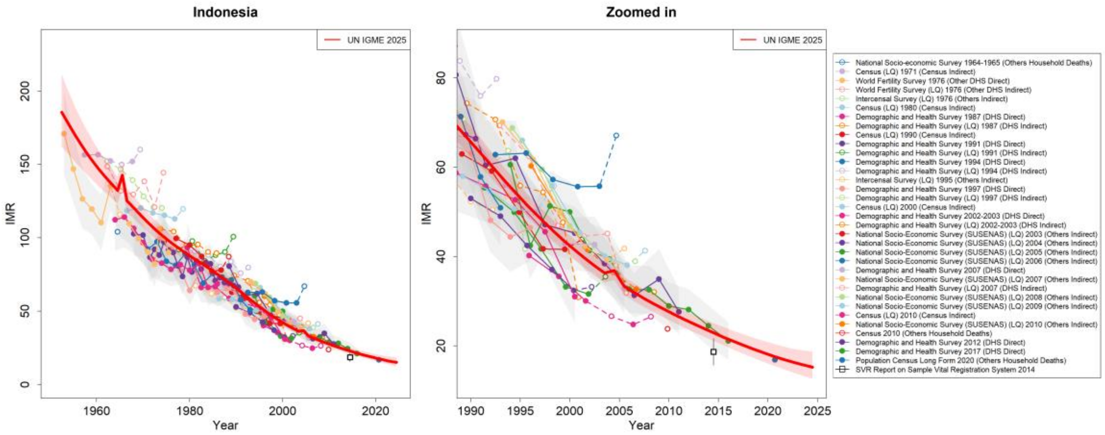


### Neonatal mortality rate

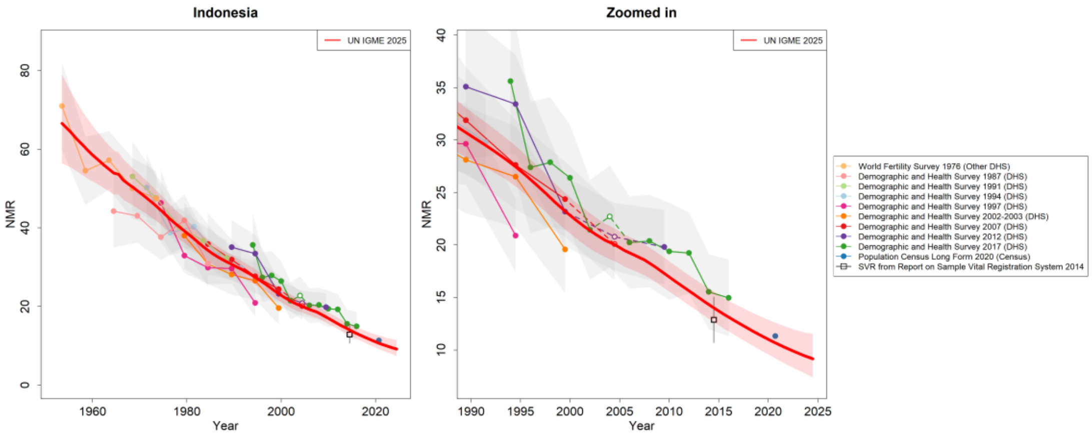

## Iran (Islamic Republic of) (IRN)

### Under-five mortality rate

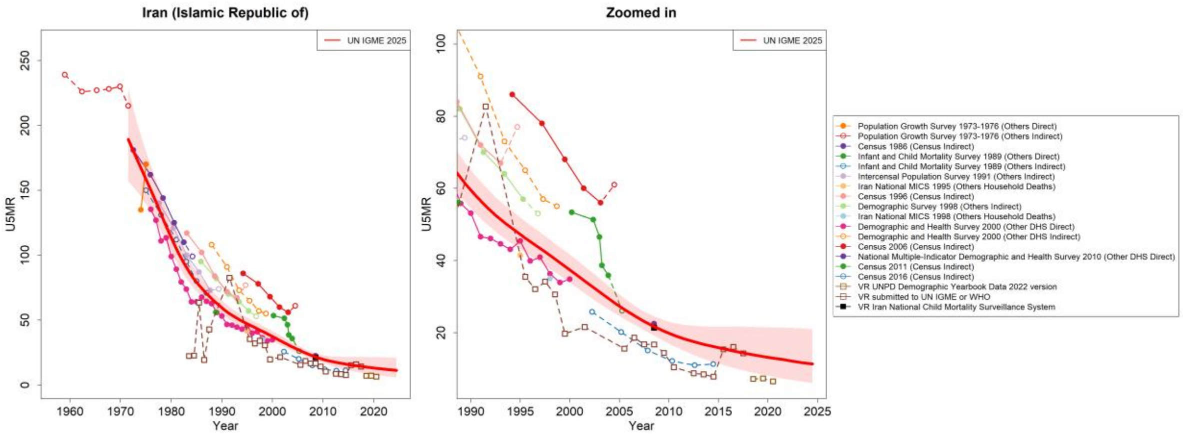


### Infant mortality rate

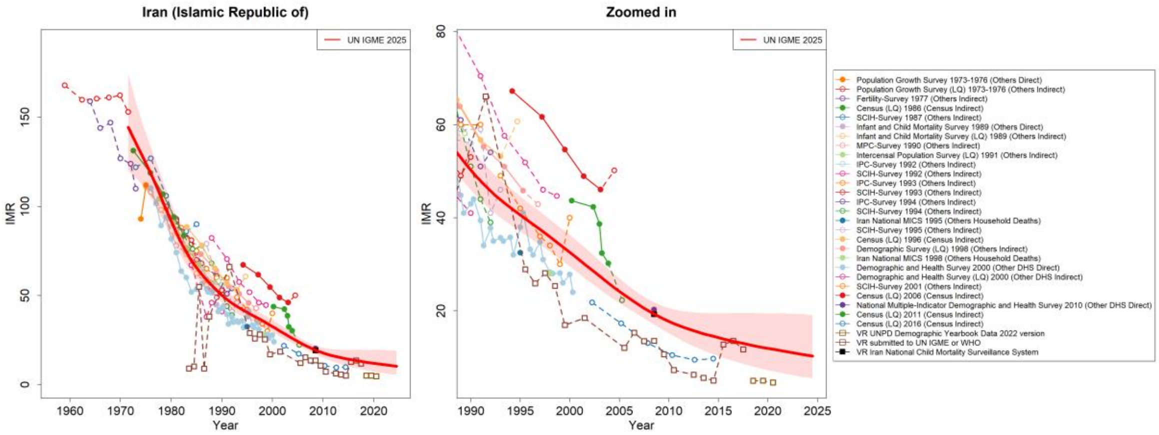


### Neonatal mortality rate

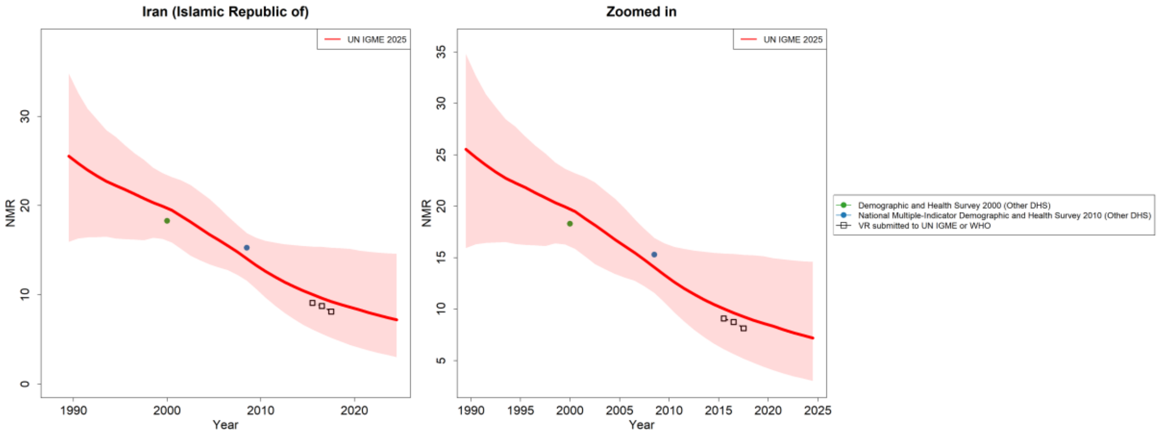

## Iraq (IRQ)

### Under-five mortality rate

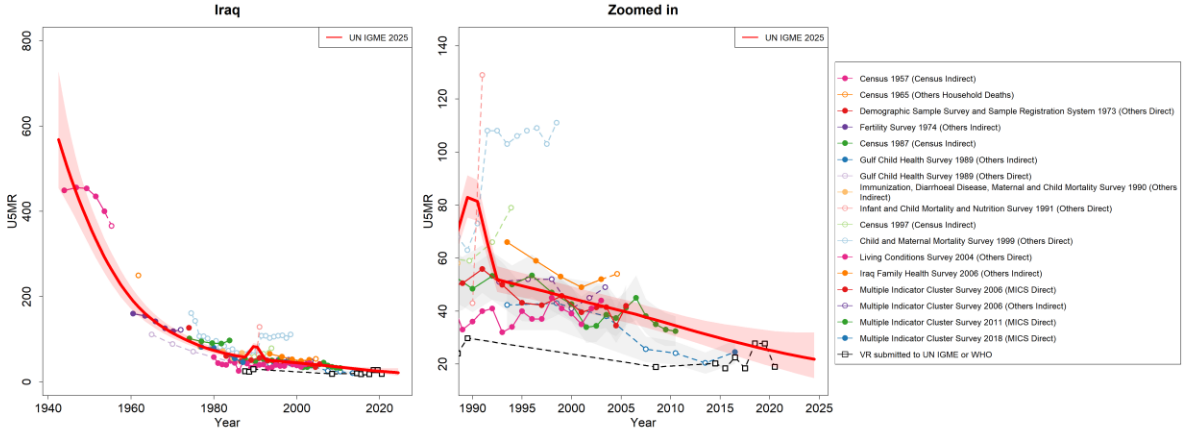


### Infant mortality rate

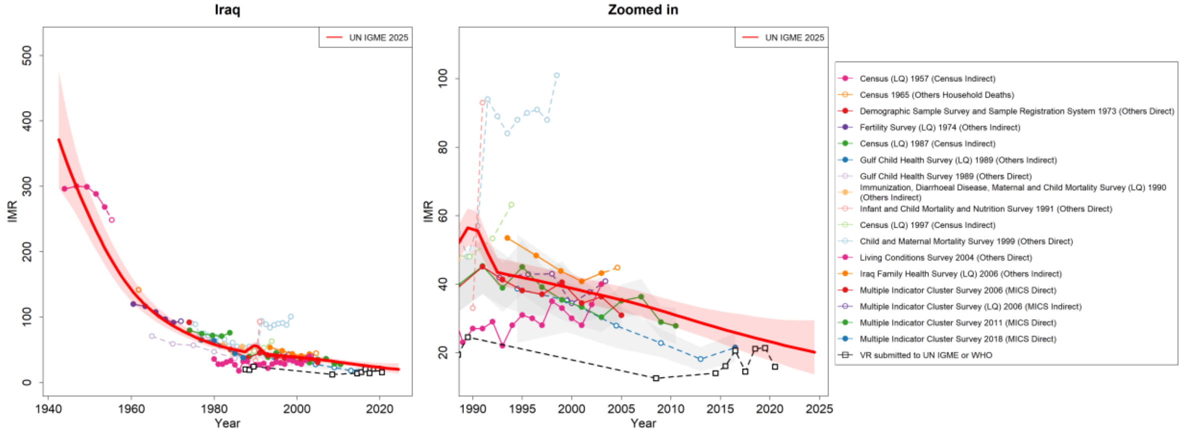


### Neonatal mortality rate

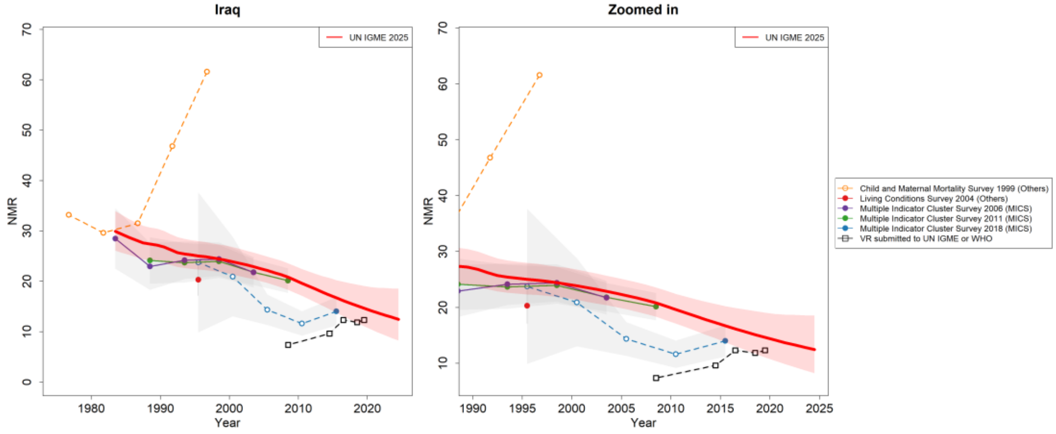

## Ireland (IRL)

### Under-five mortality rate

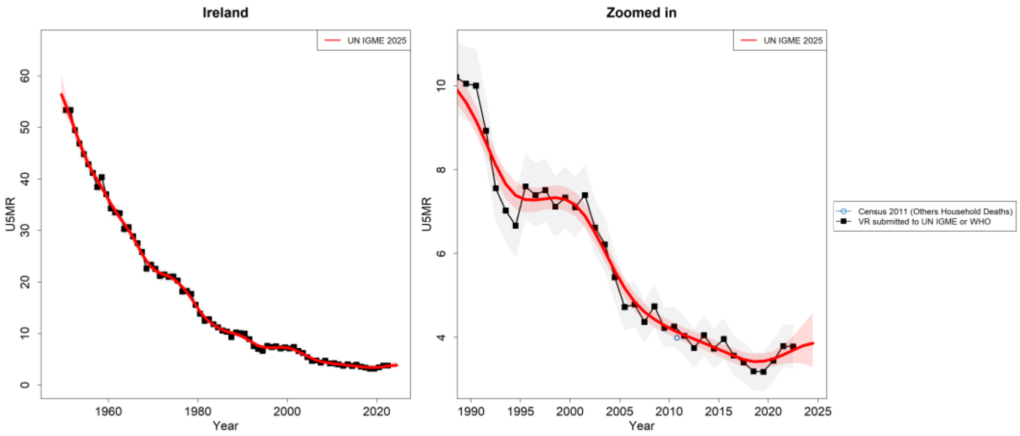


### Infant mortality rate

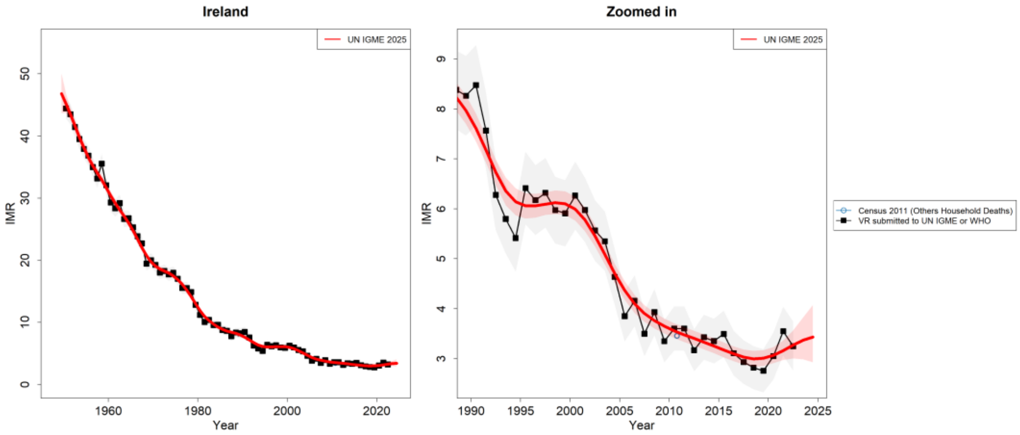


### Neonatal mortality rate

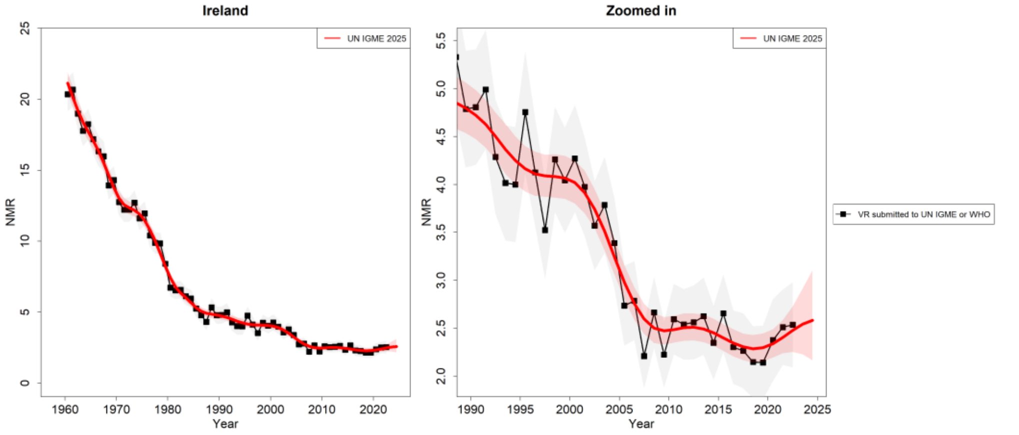

## Israel (ISR)

### Under-five mortality rate

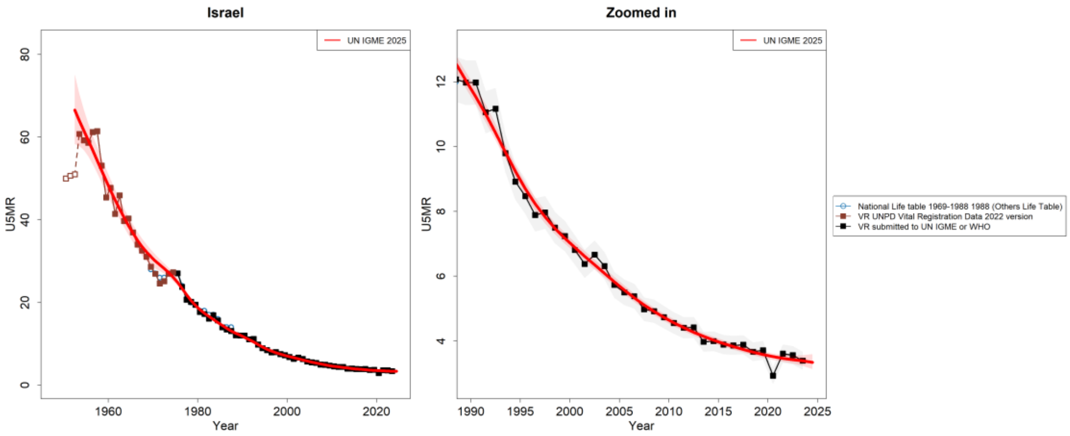


### Infant mortality rate

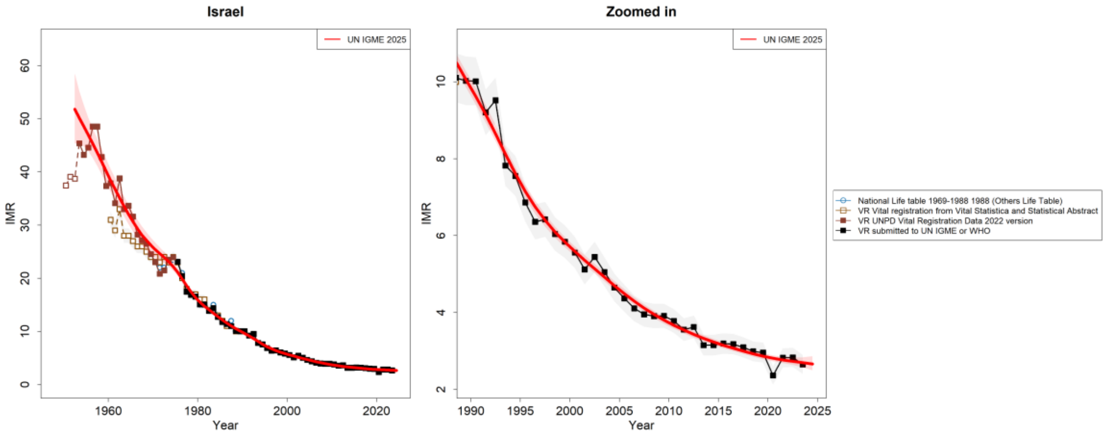


### Neonatal mortality rate

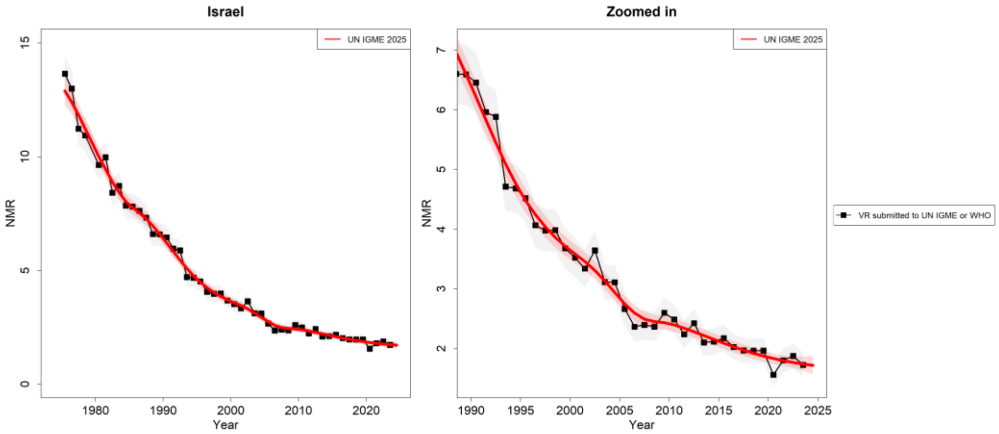

## Italy (ITA)

### Under-five mortality rate

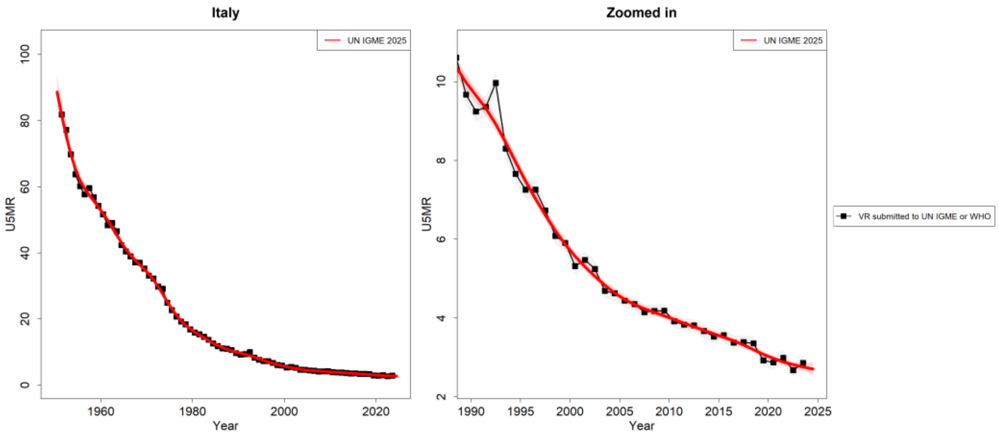


### Infant mortality rate

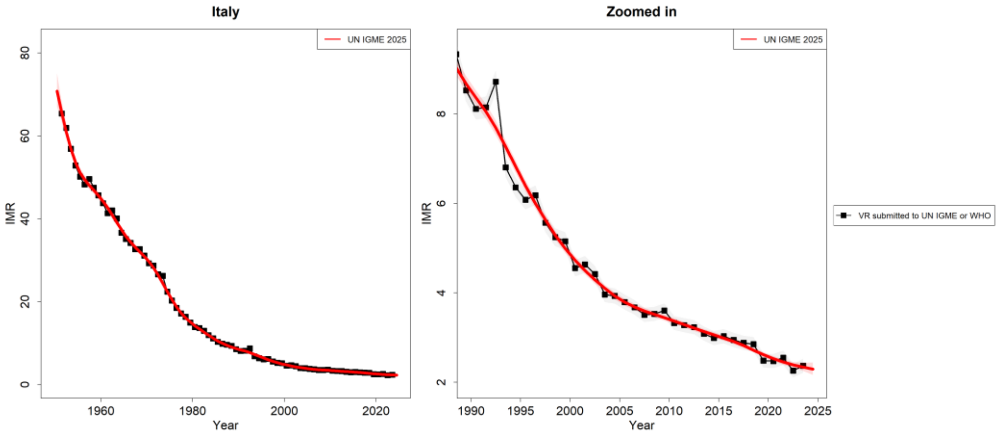


### Neonatal mortality rate

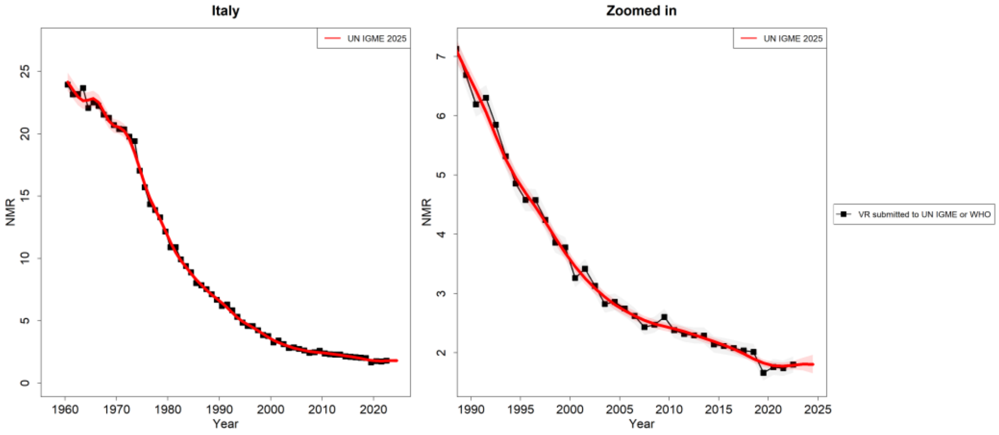

## Jamaica (JAM)

### Under-five mortality rate

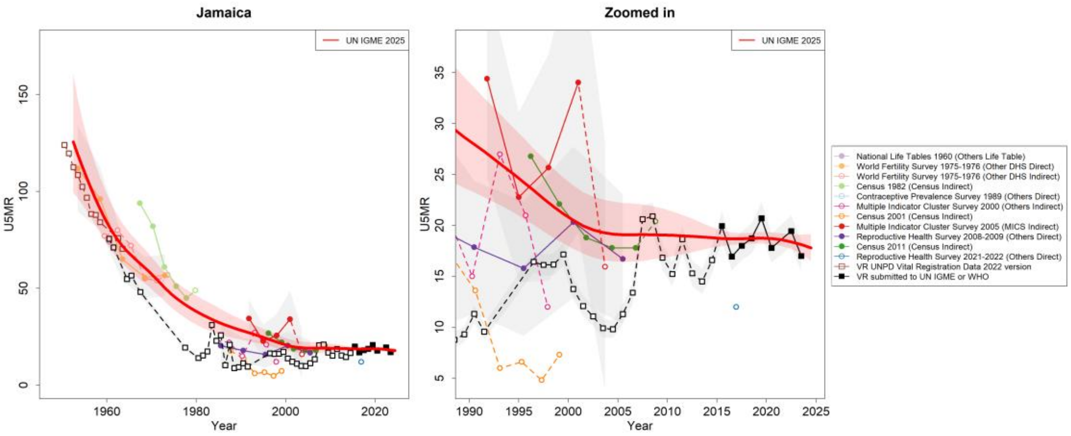


### Infant mortality rate

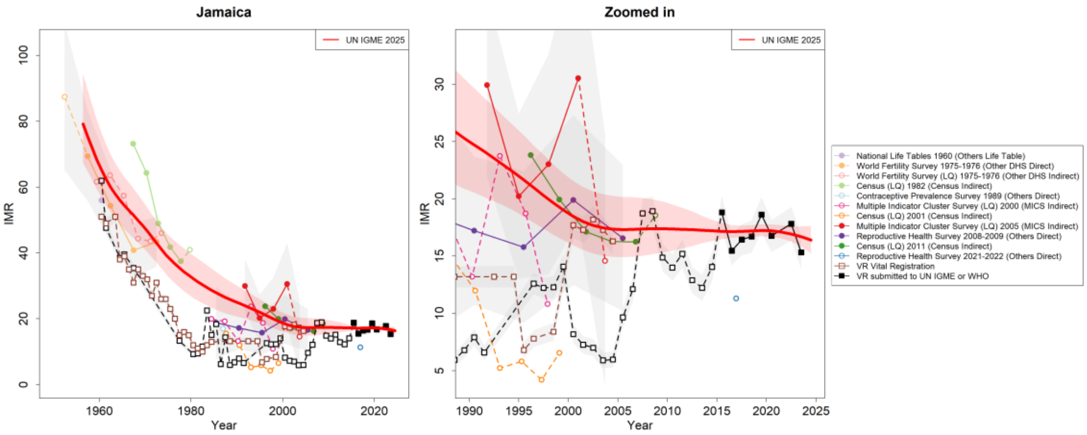


### Neonatal mortality rate

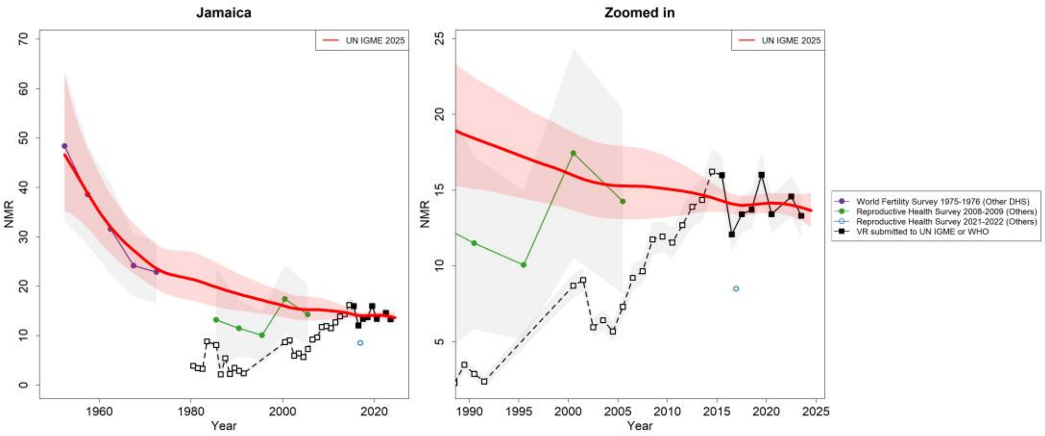

## Japan (JPN)

### Under-five mortality rate

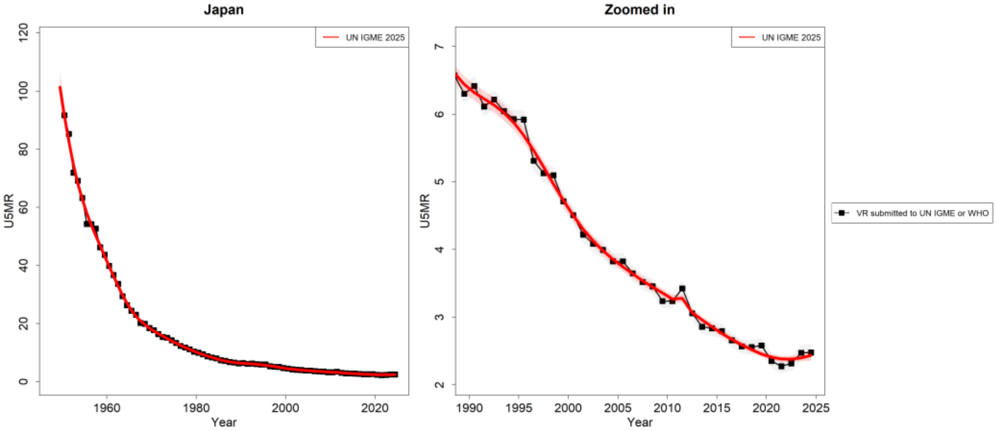


### Infant mortality rate

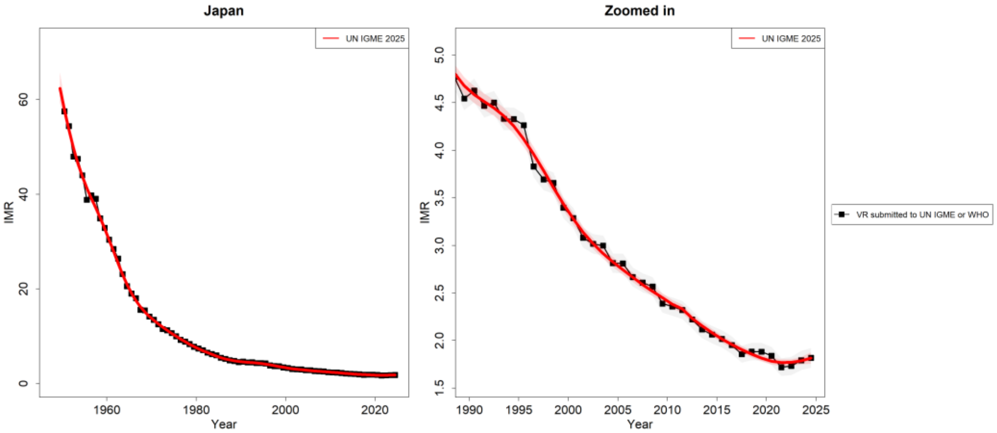


### Neonatal mortality rate

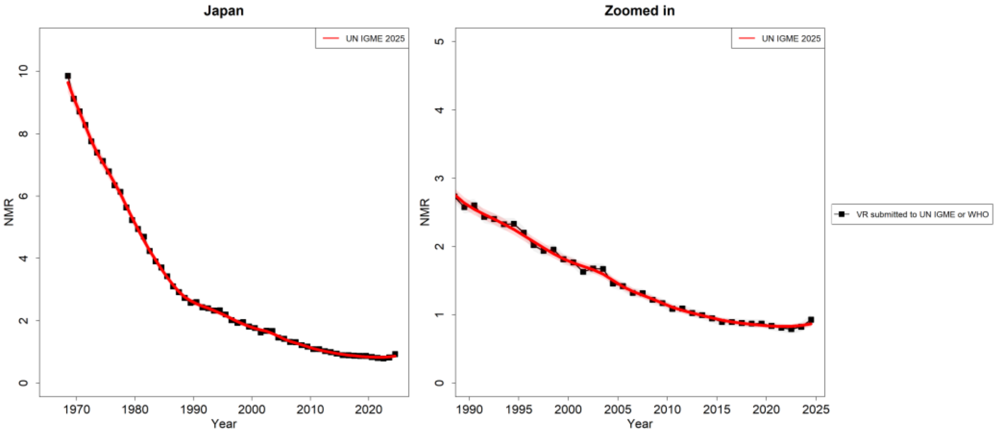

## Jordan (JOR)

### Under-five mortality rate

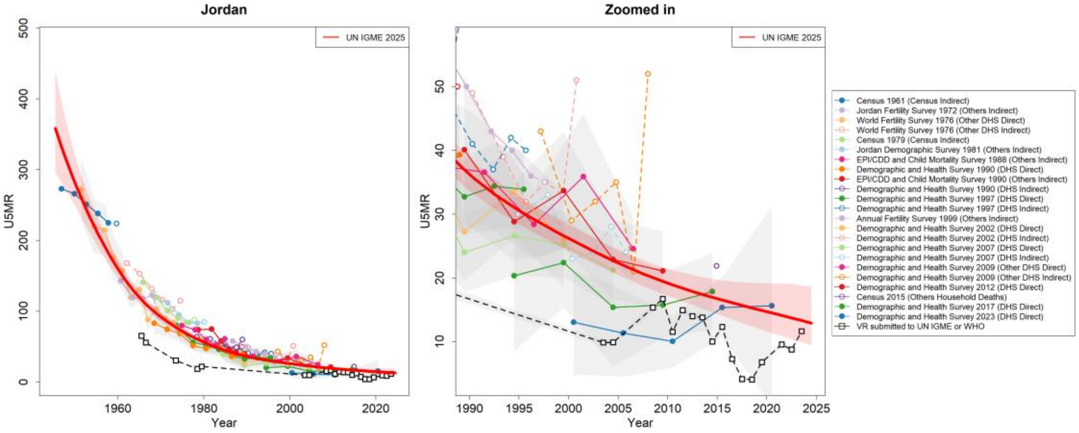


### Infant mortality rate

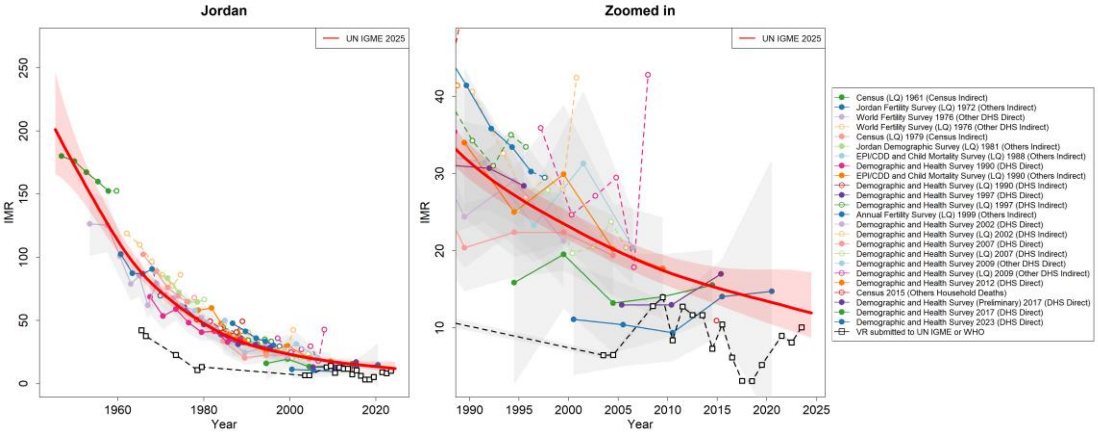


### Neonatal mortality rate

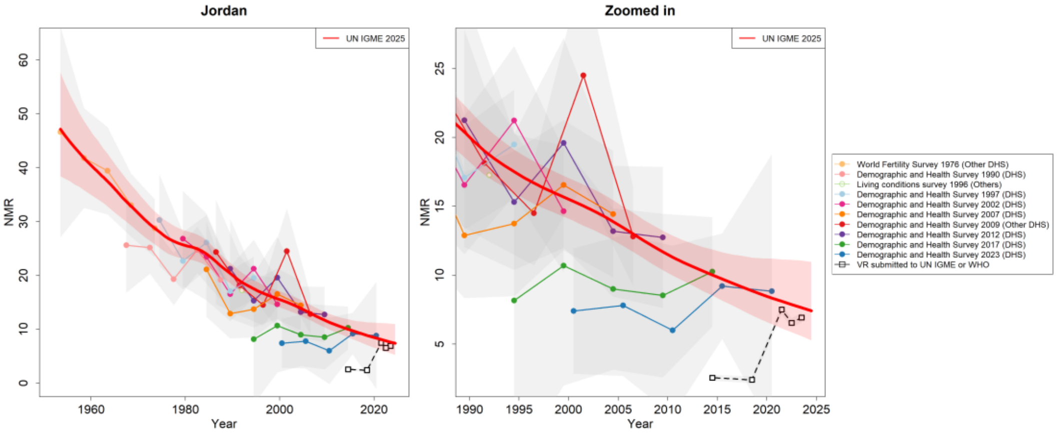

## Kazakhstan (KAZ)

### Under-five mortality rate

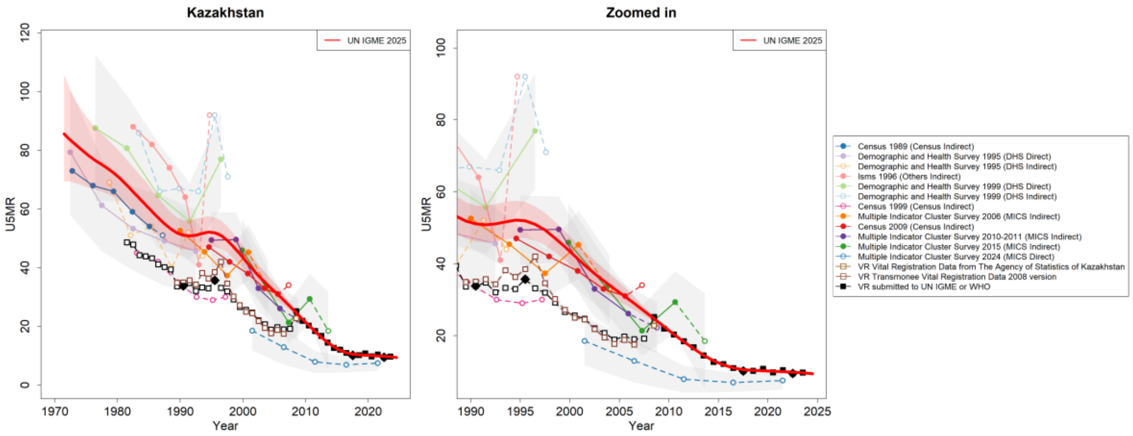


### Infant mortality rate

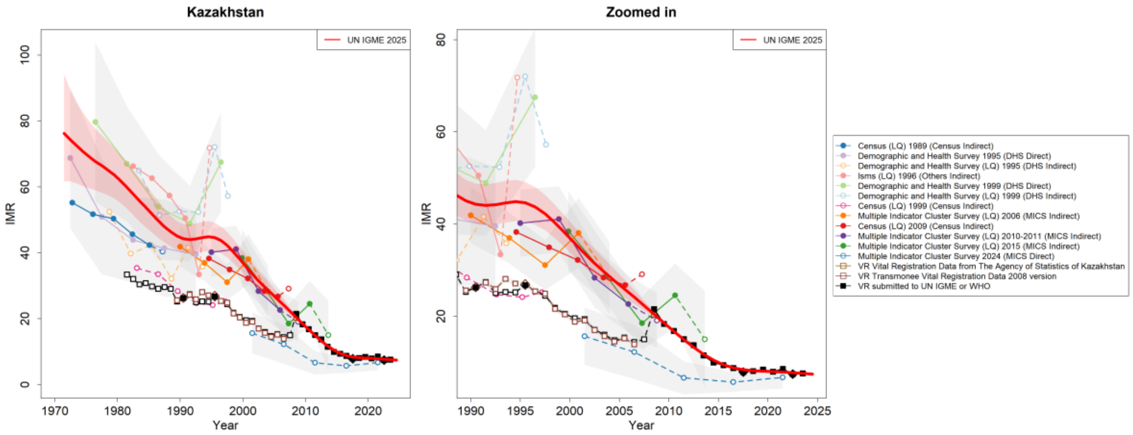


### Neonatal mortality rate

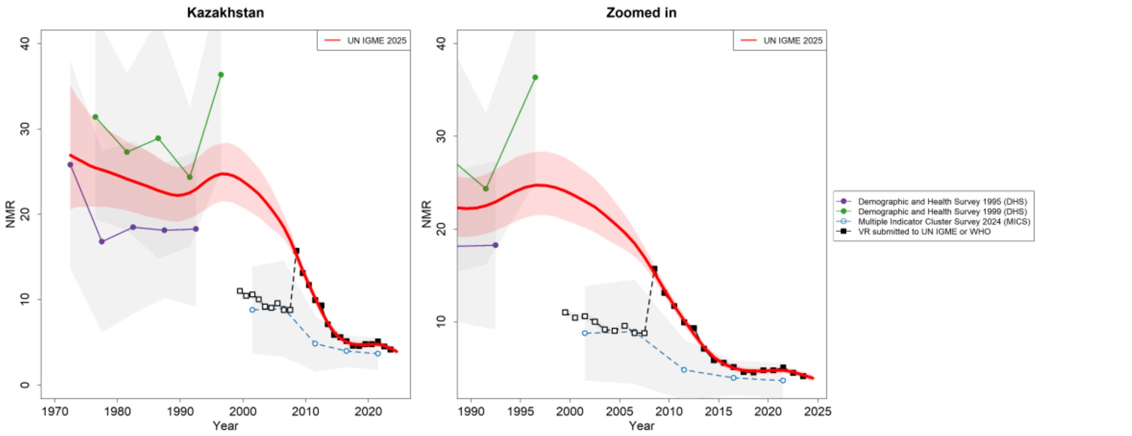

# Kenya (KEN)

## Under-five mortality rate

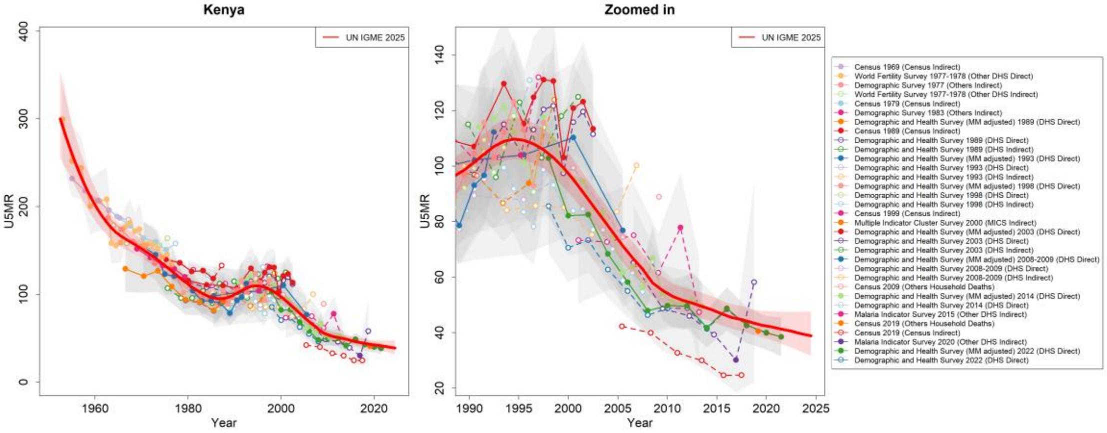


## Infant mortality rate

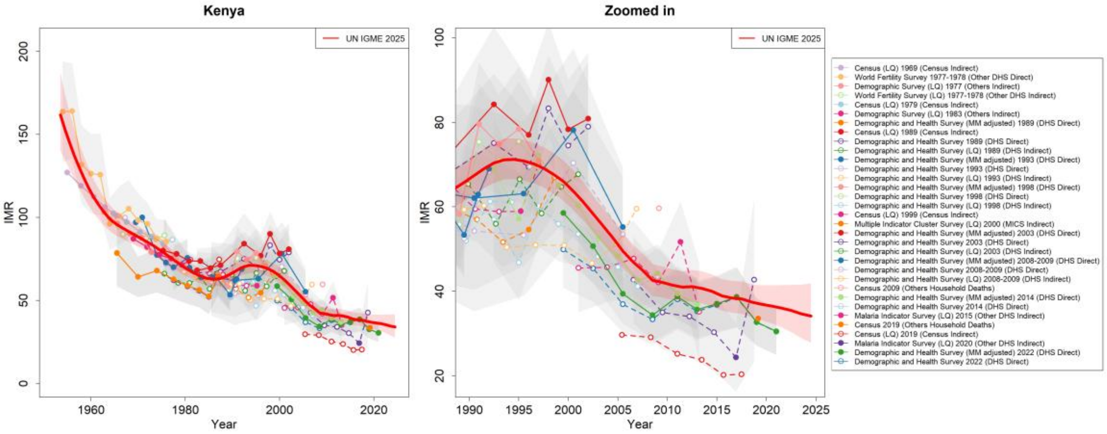


## Neonatal mortality rate

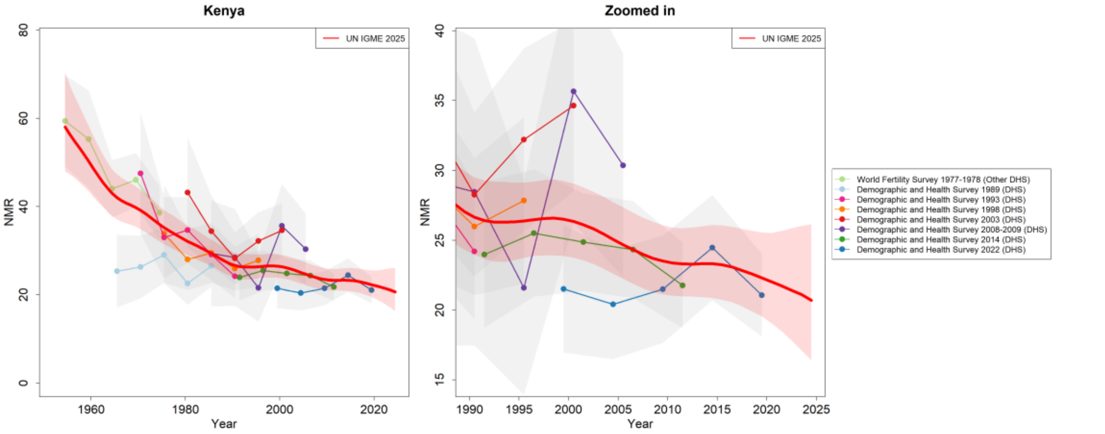

## Kiribati (KIR)

### Under-five mortality rate

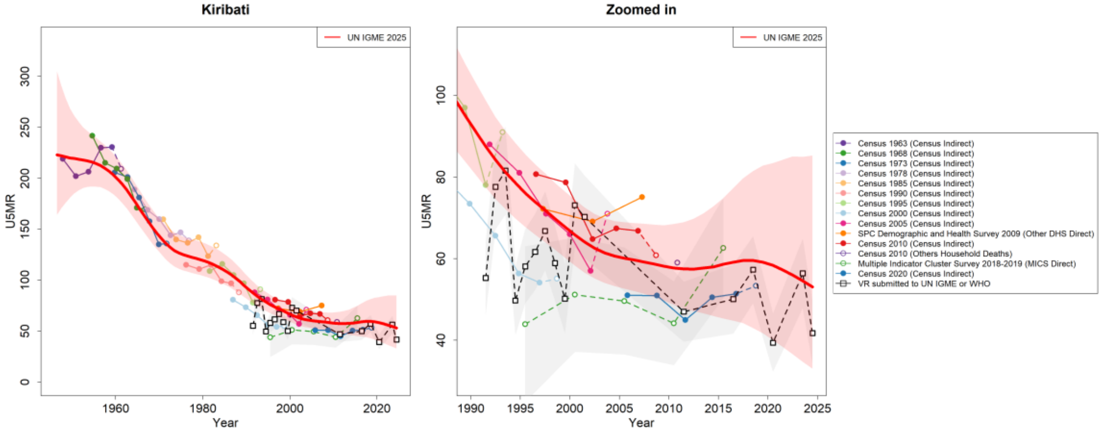


### Infant mortality rate

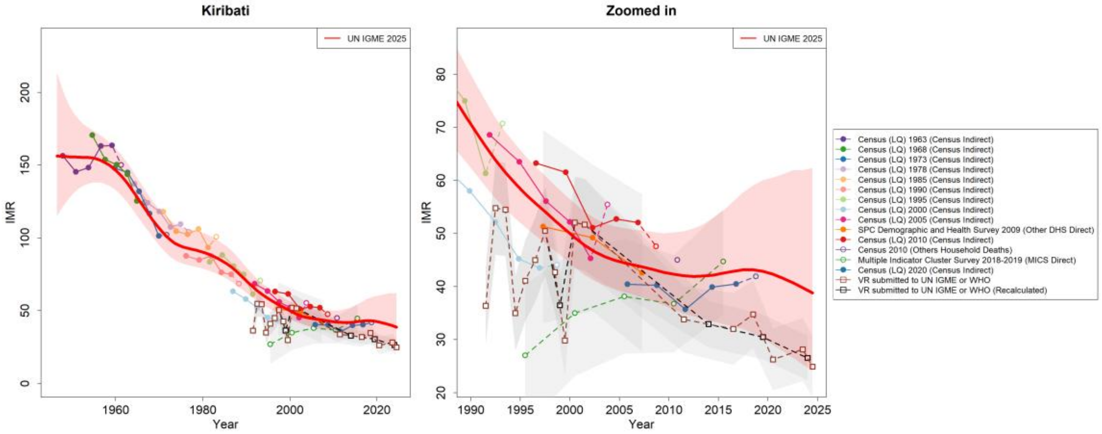


### Neonatal mortality rate

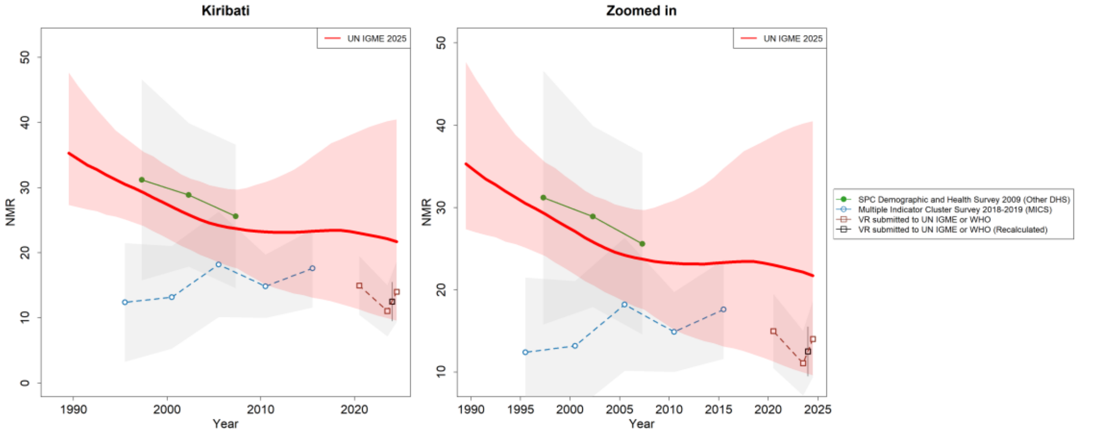

## Kosovo (UNSCR 1244) (RKS)

### Under-five mortality rate

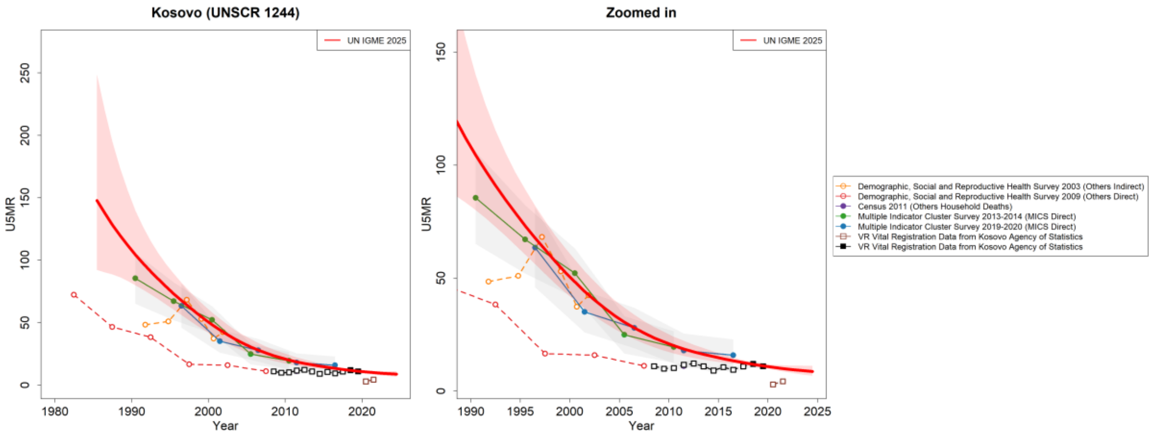


### Infant mortality rate

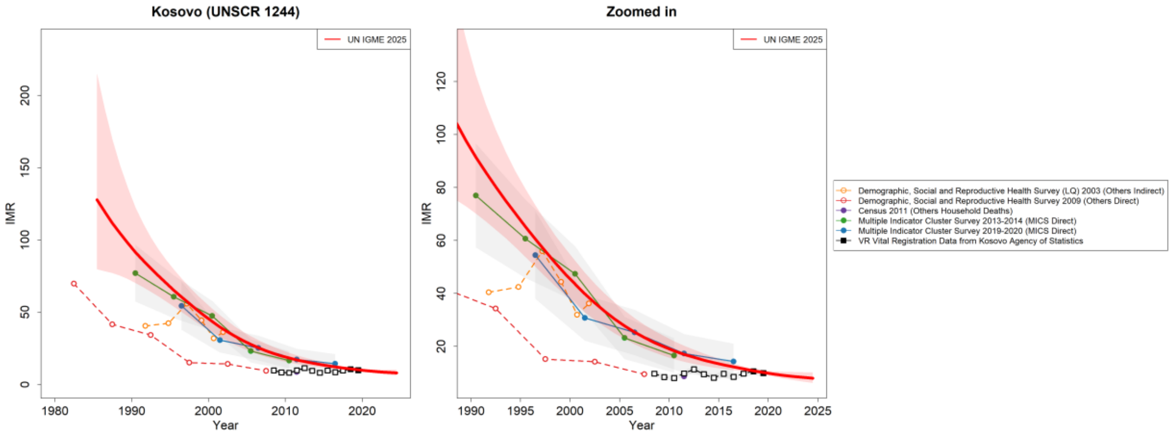


### Neonatal mortality rate

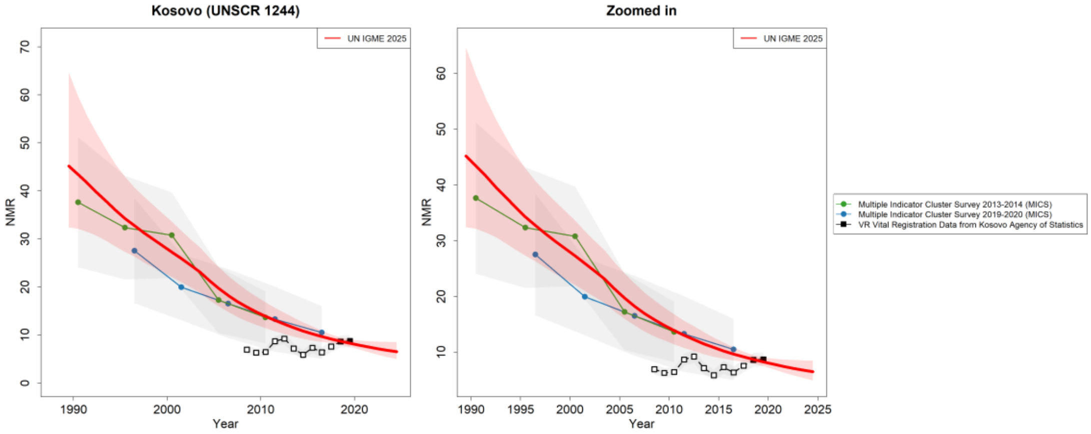

## Kuwait (KWT)

### Under-five mortality rate

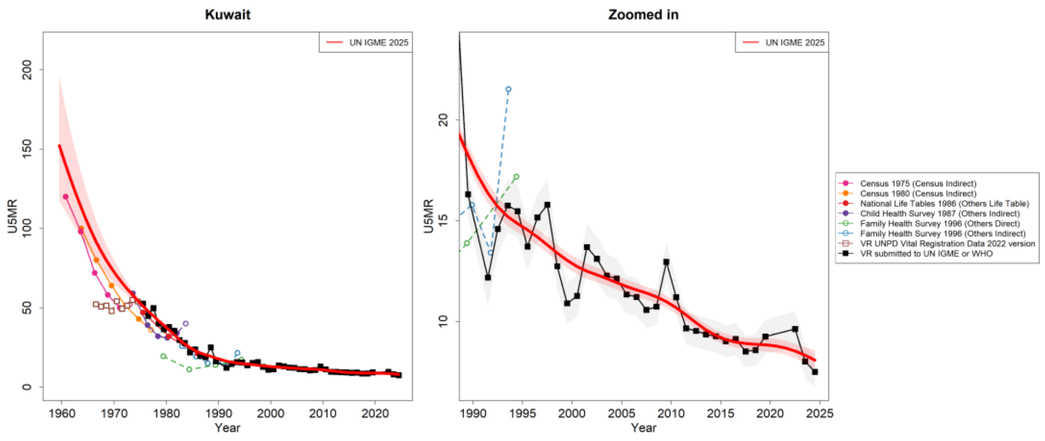


### Infant mortality rate

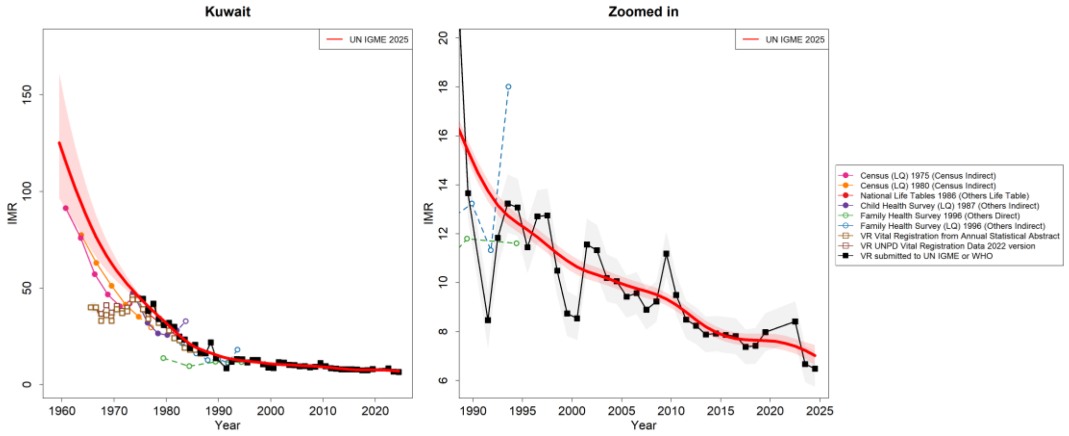


### Neonatal mortality rate

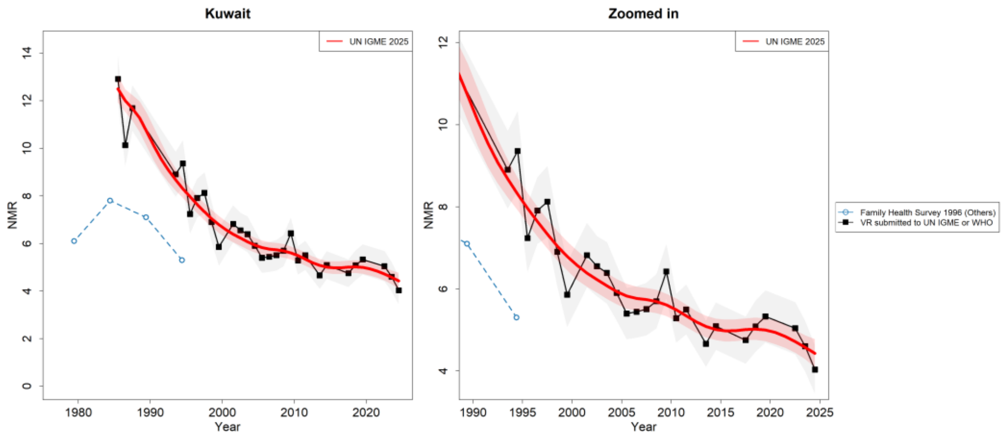

## Kyrgyzstan (KGZ)

### Under-five mortality rate

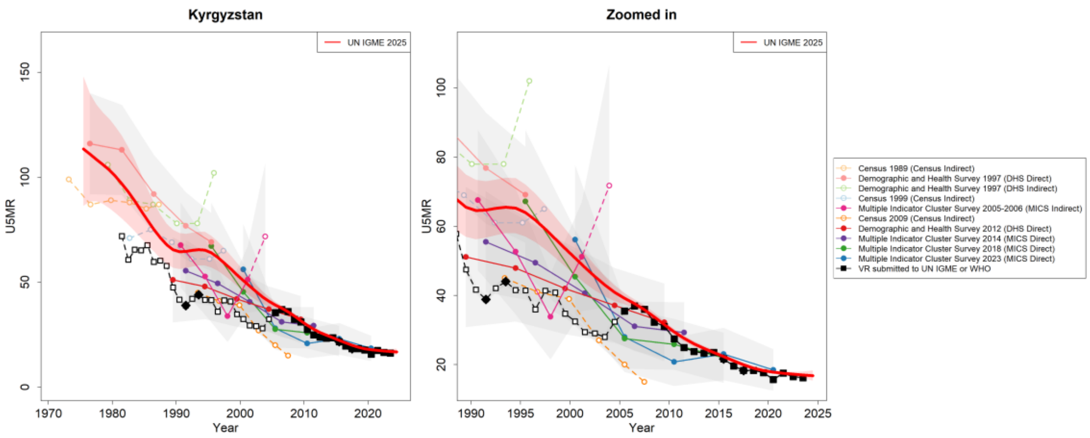


### Infant mortality rate

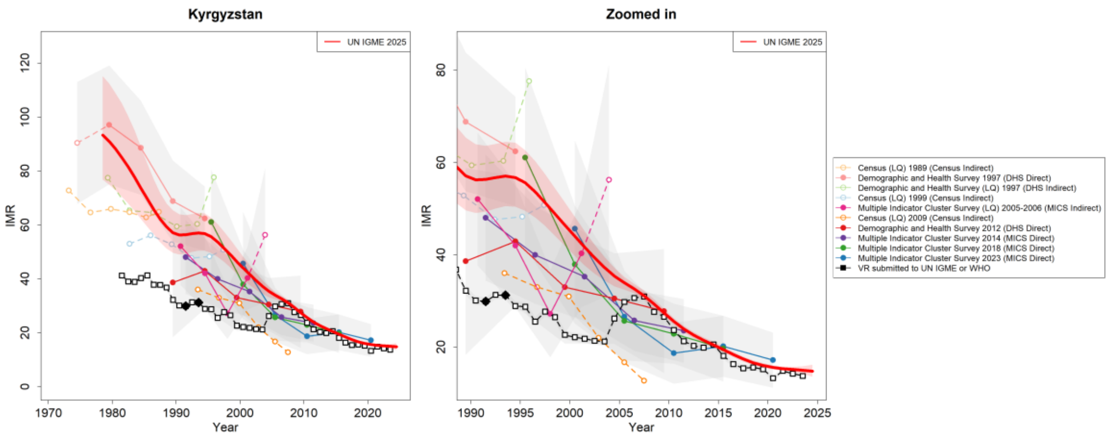


### Neonatal mortality rate

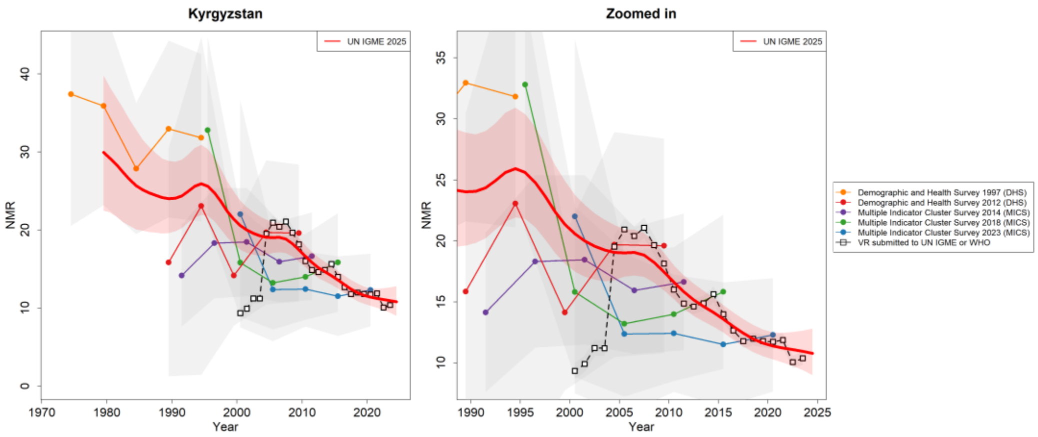

## Lao People’s Democratic Republic (LAO)

### Under-five mortality rate

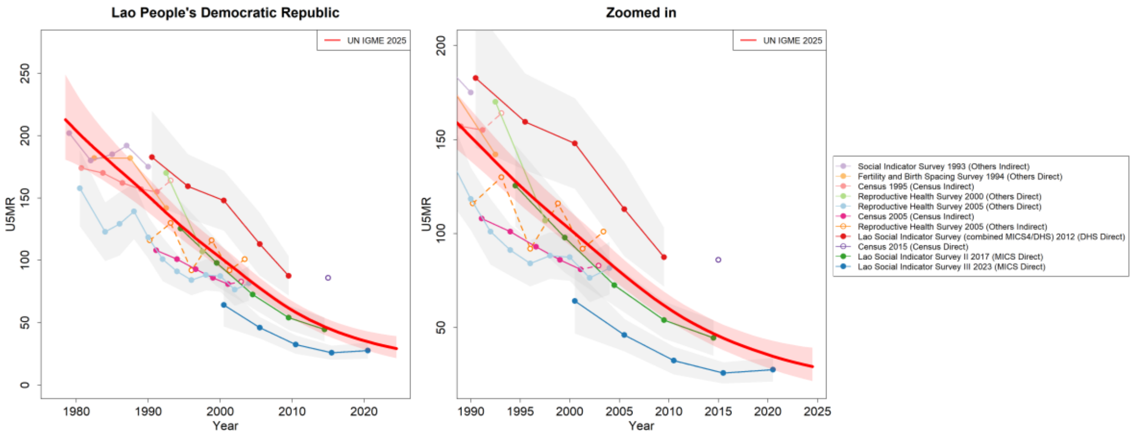


### Infant mortality rate

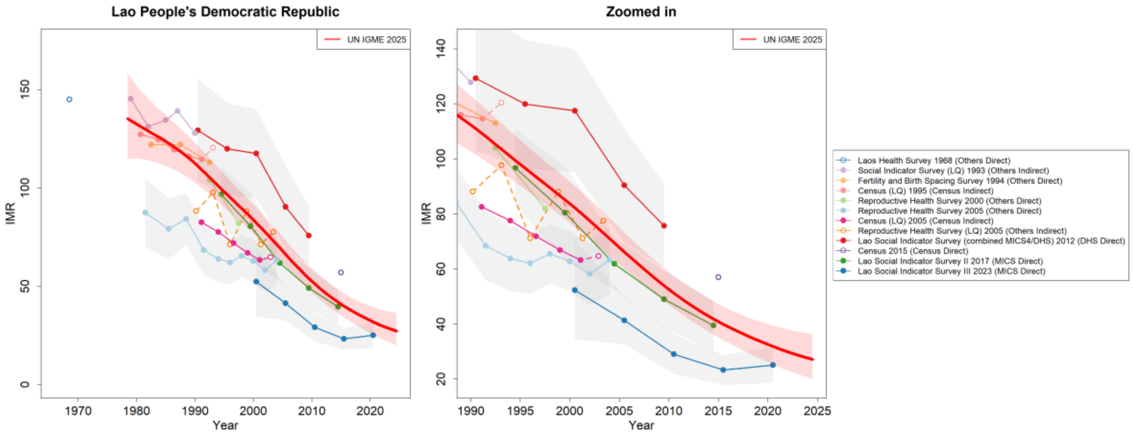


### Neonatal mortality rate

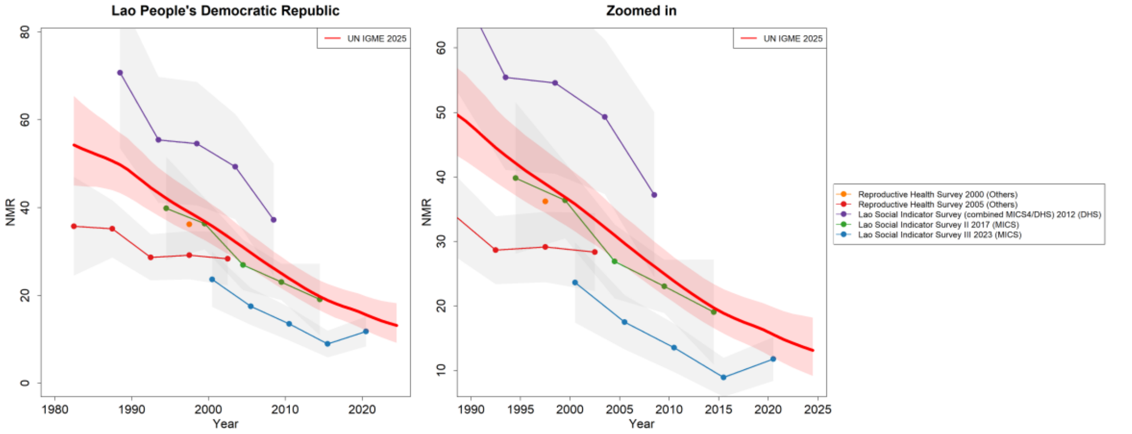

## Latvia (LVA)

### Under-five mortality rate

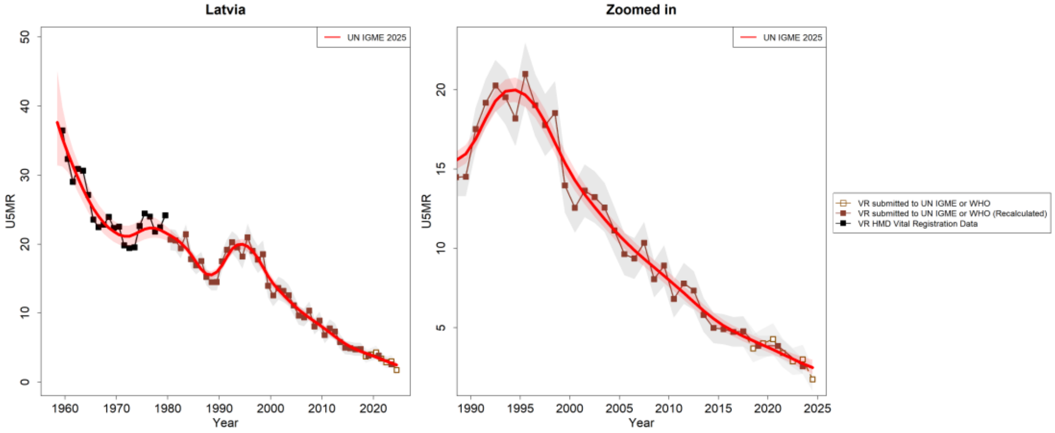


### Infant mortality rate

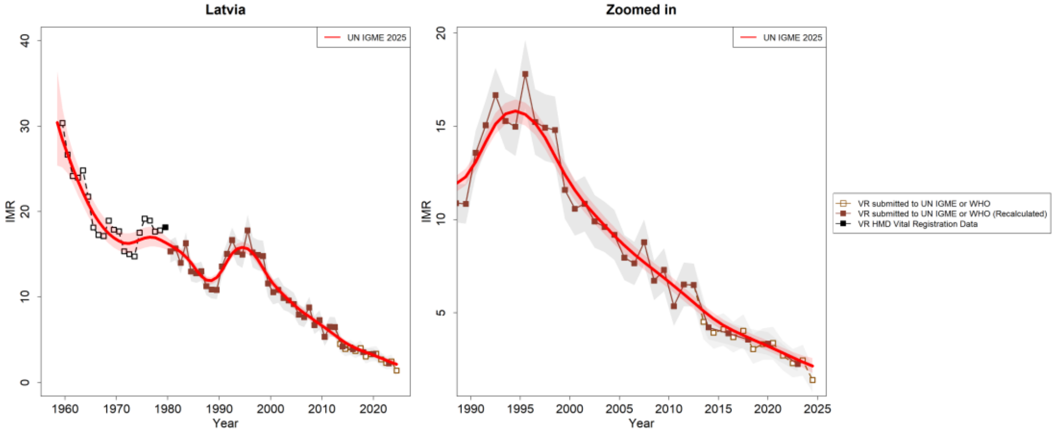


### Neonatal mortality rate

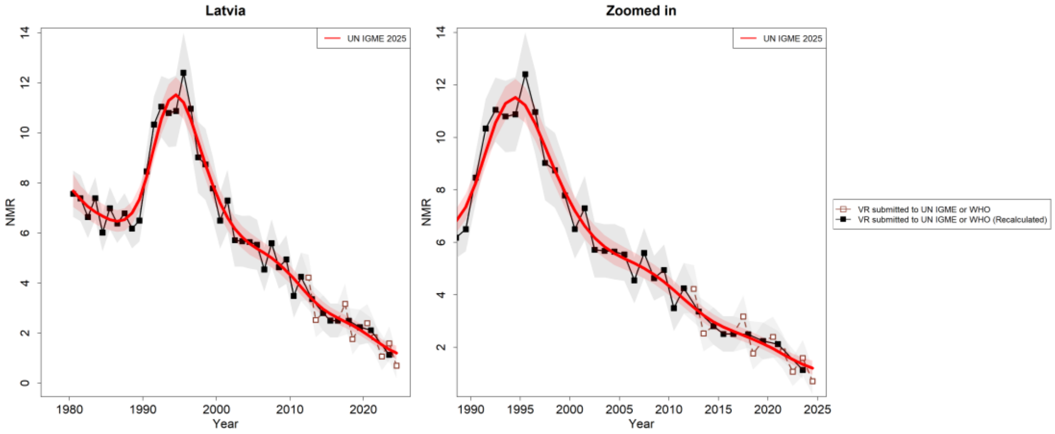

## Lebanon (LBN)

### Under-five mortality rate

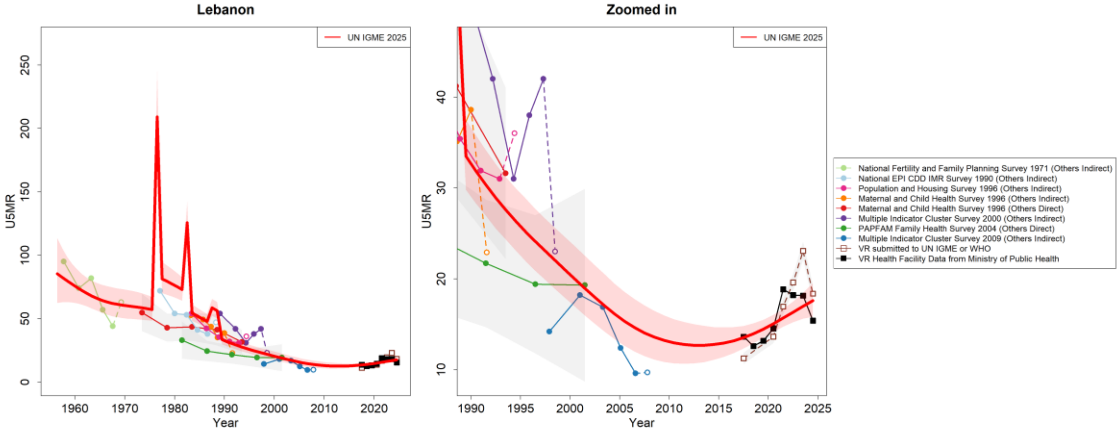


### Infant mortality rate

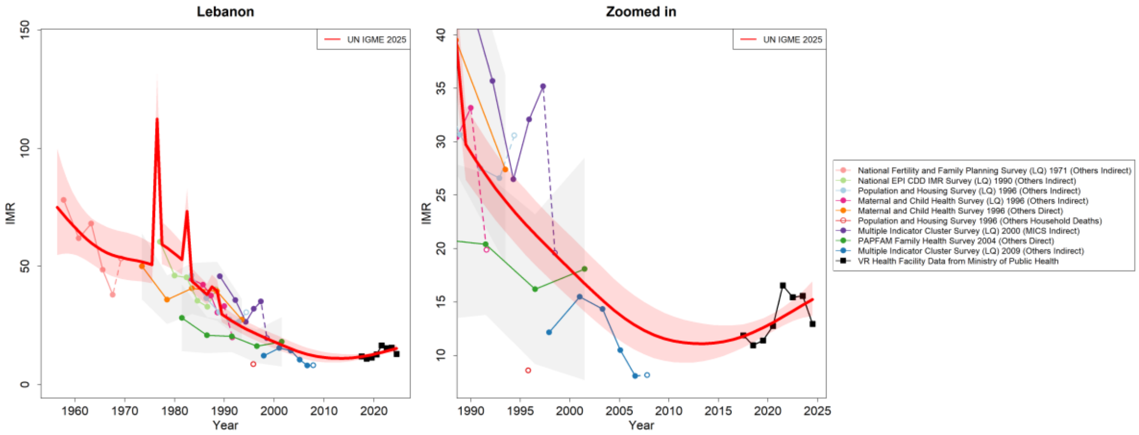


### Neonatal mortality rate

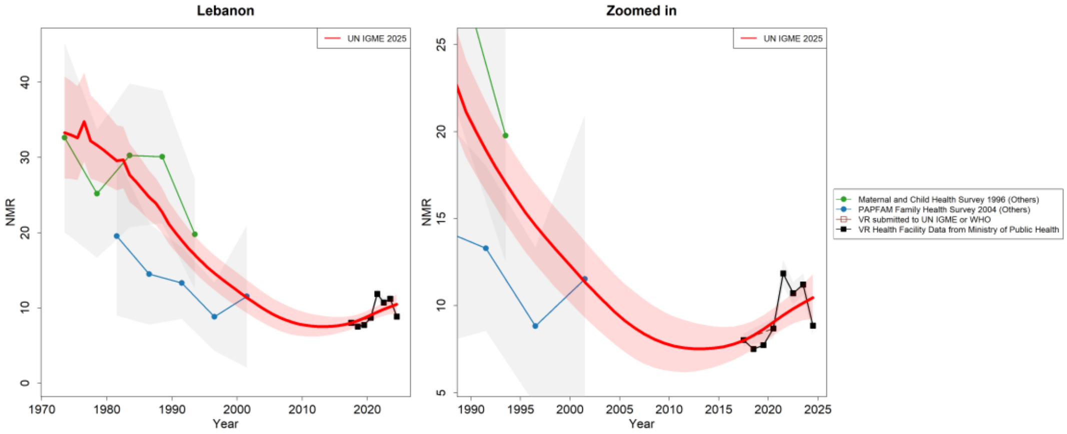

# Lesotho (LSO)

## Under-five mortality rate

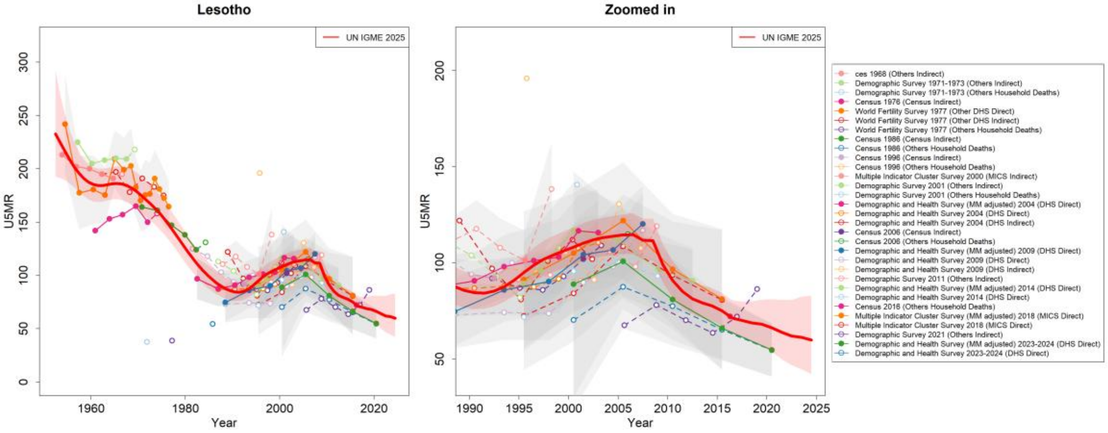


## Infant mortality rate

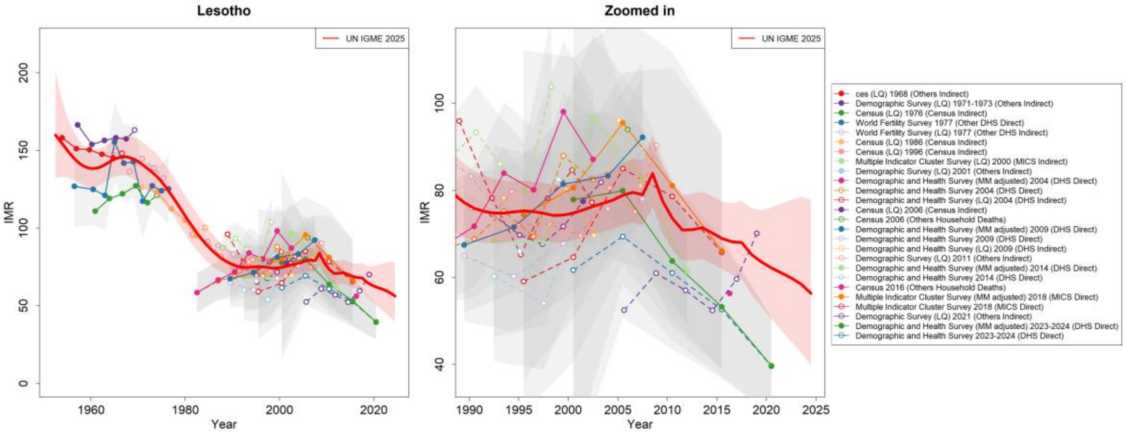


## Neonatal mortality rate

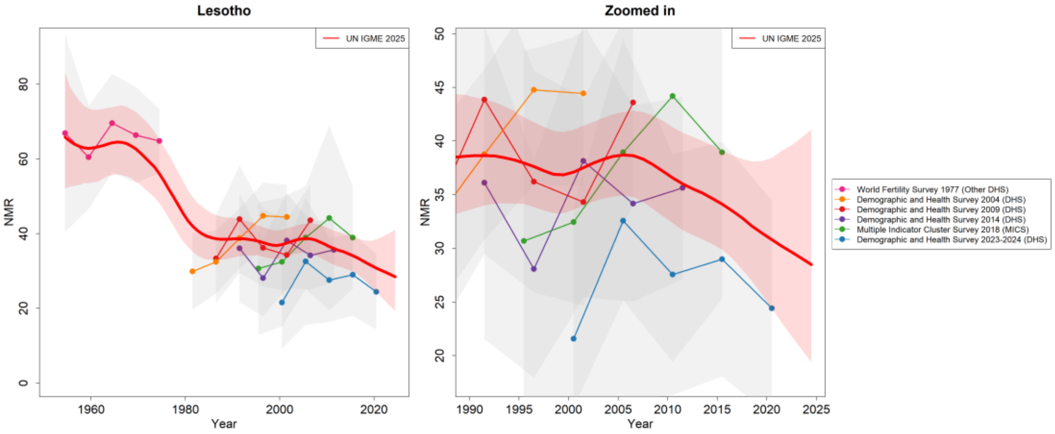

# Liberia (LBR)

## Under-five mortality rate

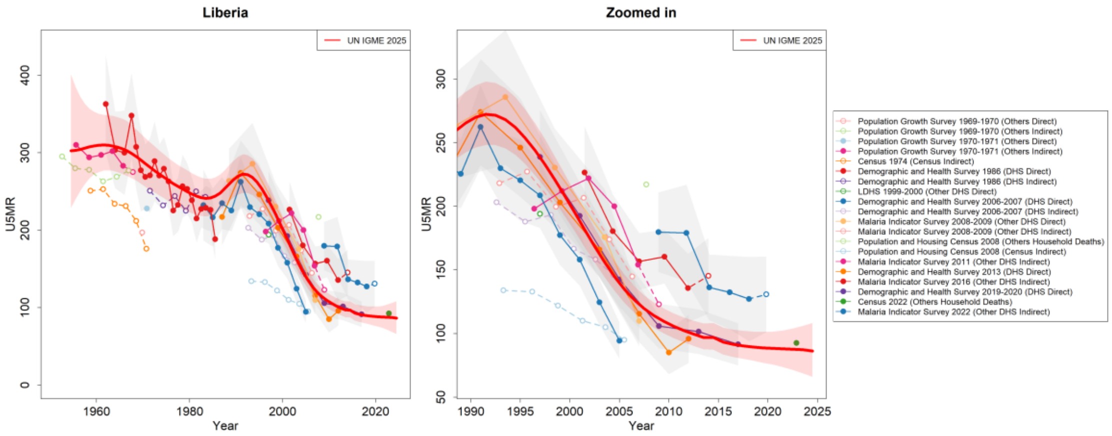


## Infant mortality rate

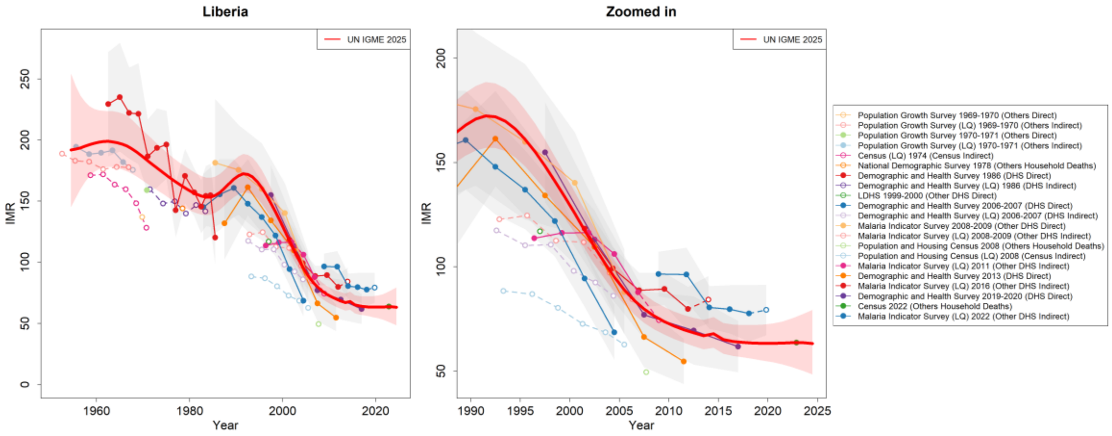


## Neonatal mortality rate

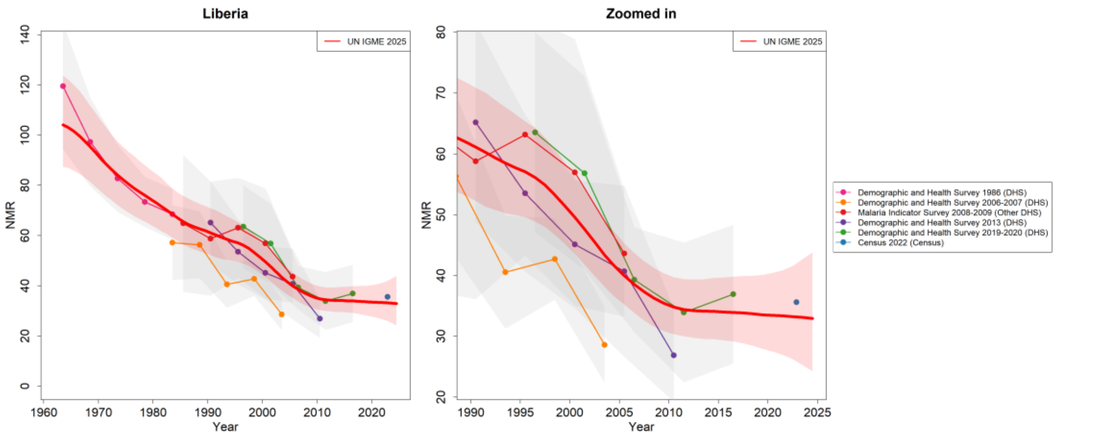

## Libya (LBY)

### Under-five mortality rate

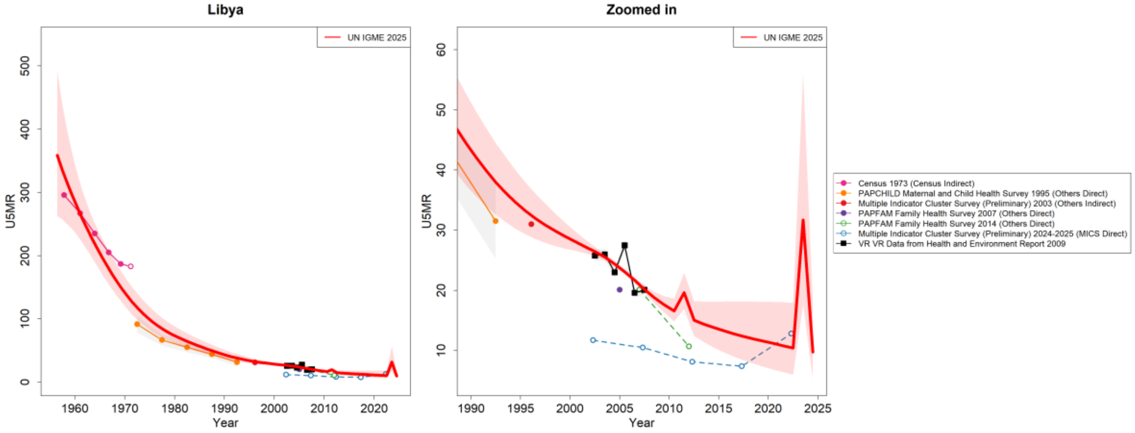


### Infant mortality rate

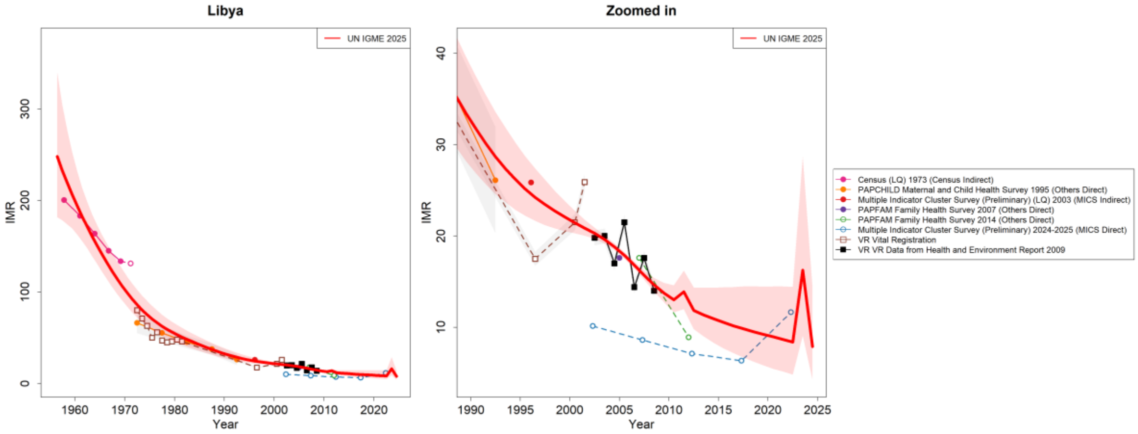


### Neonatal mortality rate

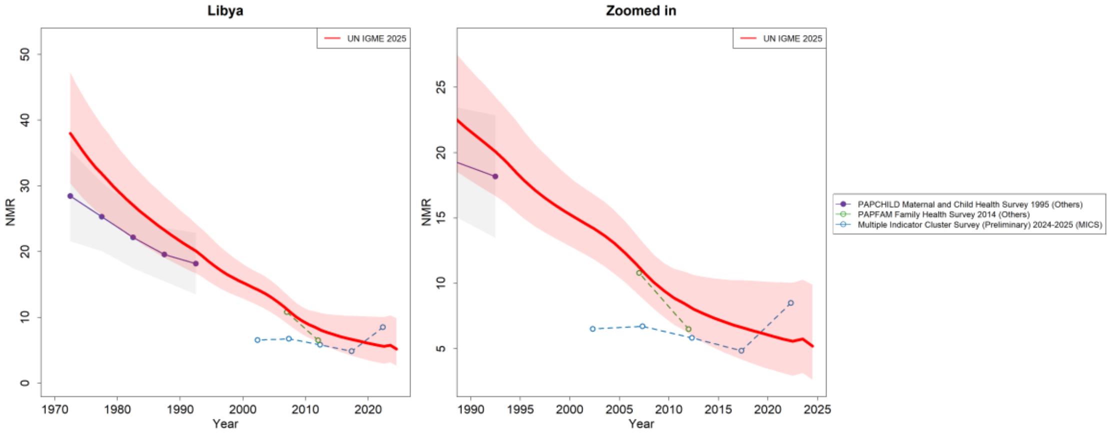

## Lithuania (LTU)

### Under-five mortality rate

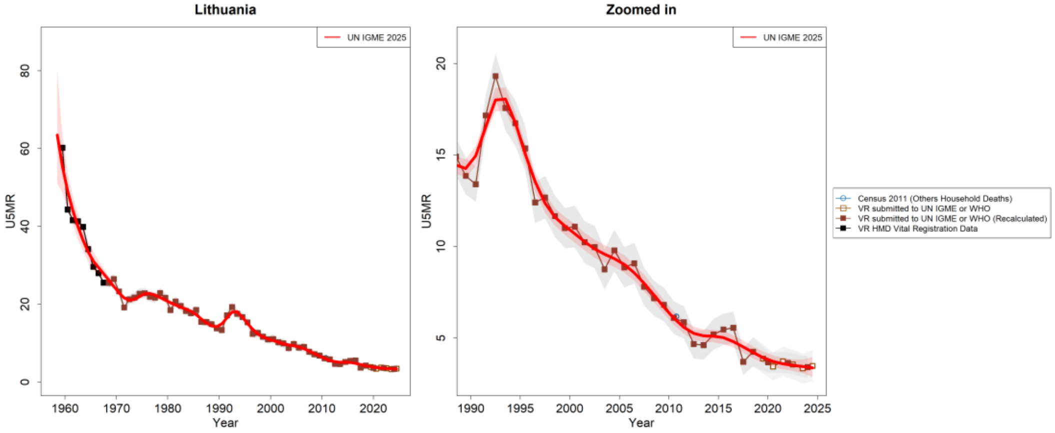


### Infant mortality rate

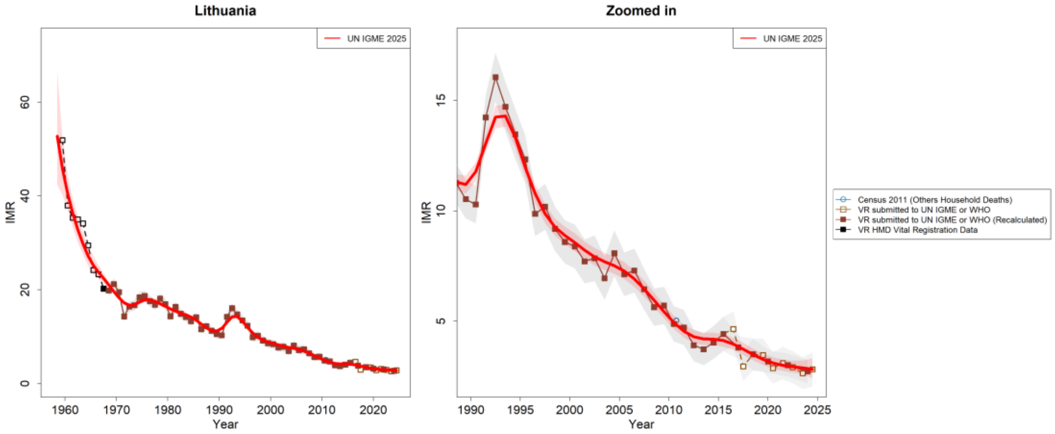


### Neonatal mortality rate

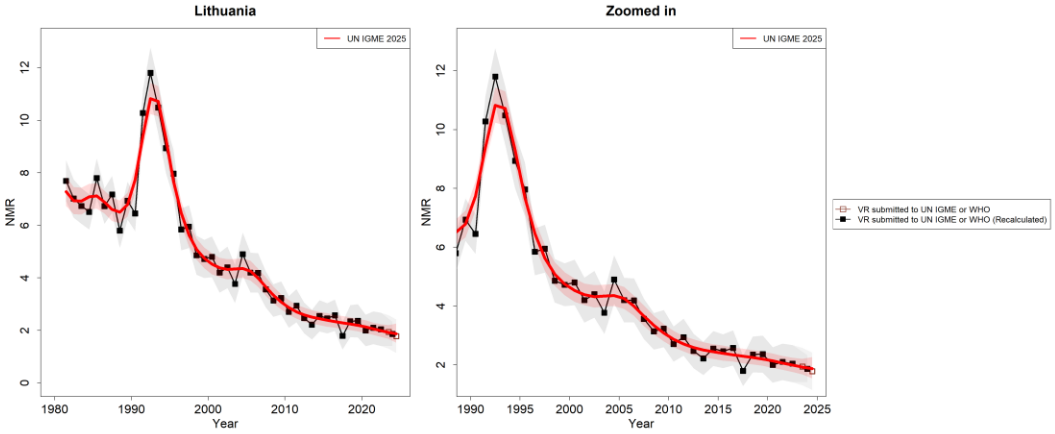

## Luxembourg (LUX)

### Under-five mortality rate

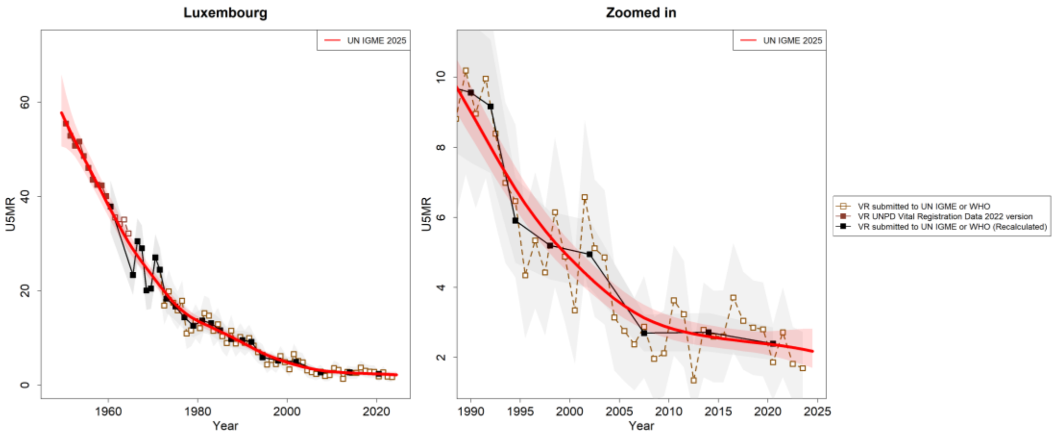


### Infant mortality rate

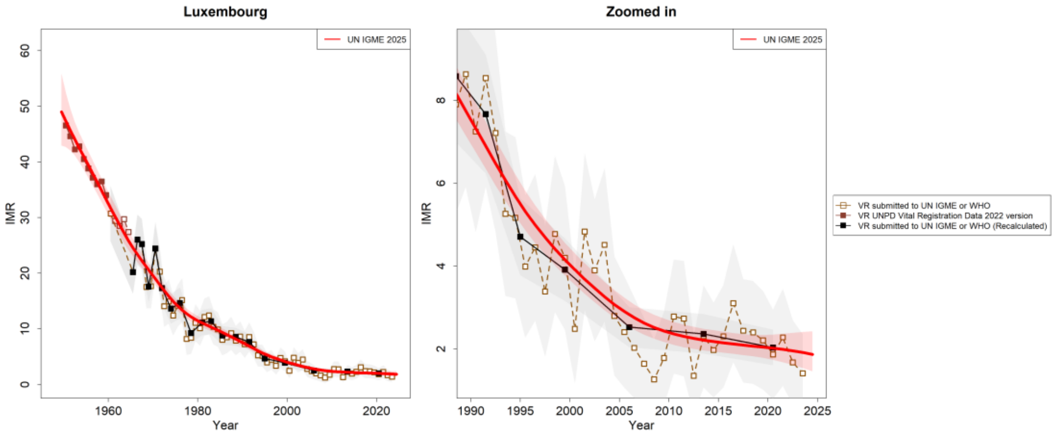


### Neonatal mortality rate

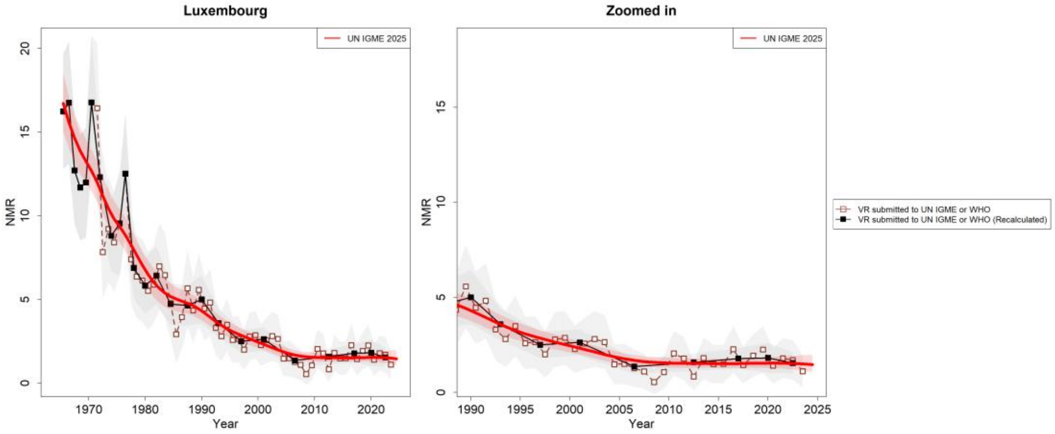

## Madagascar (MDG)

### Under-five mortality rate

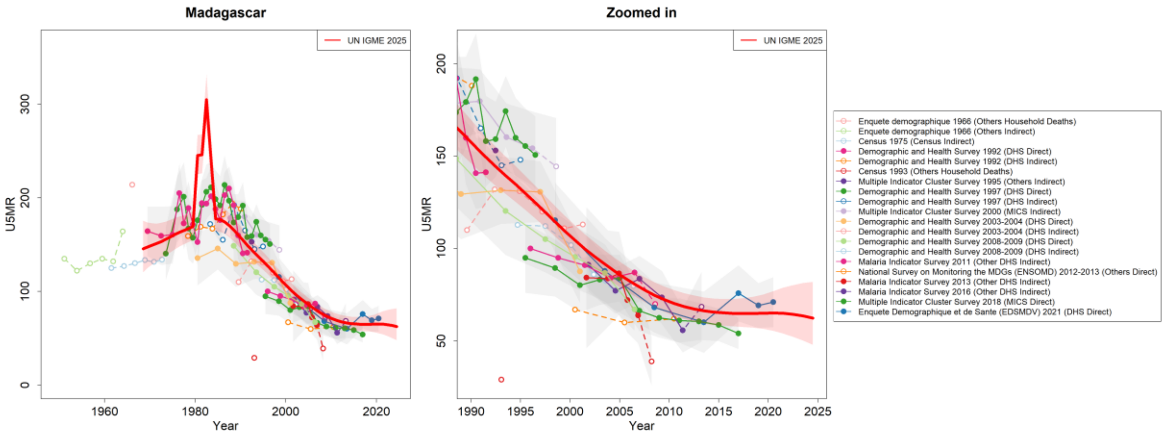


### Infant mortality rate

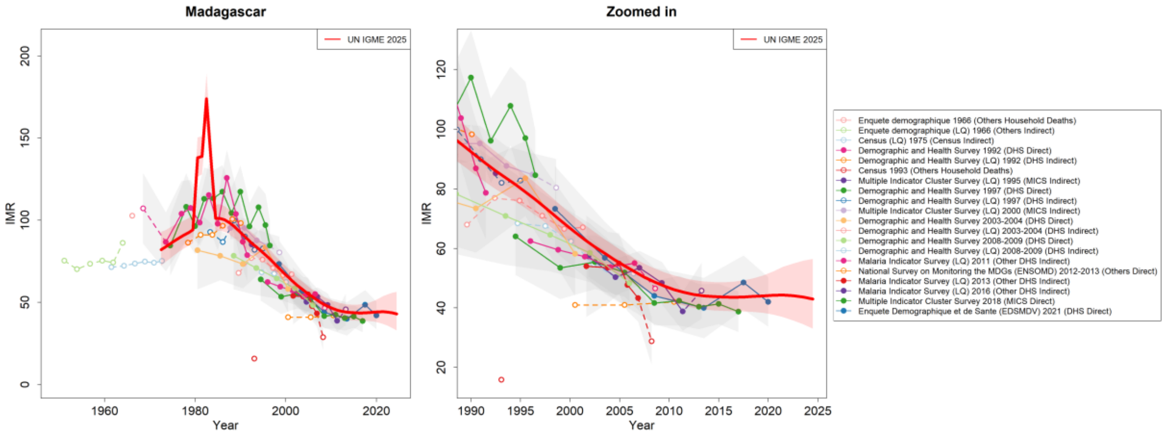


### Neonatal mortality rate

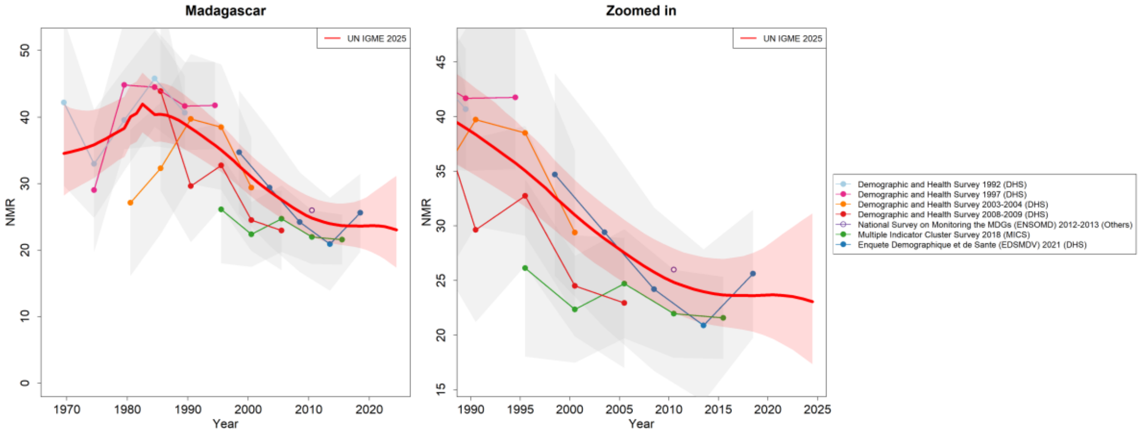

## Malawi (MWI)

### Under-five mortality rate

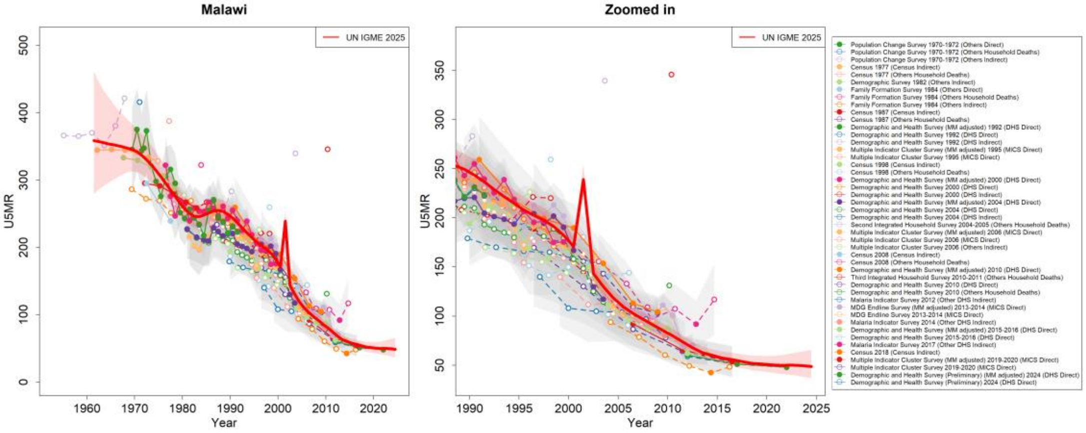


### Infant mortality rate

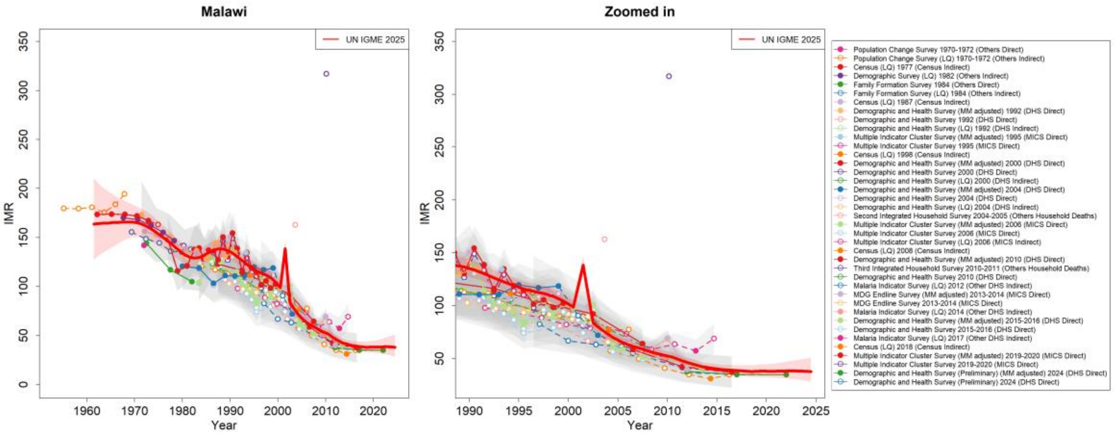


### Neonatal mortality rate

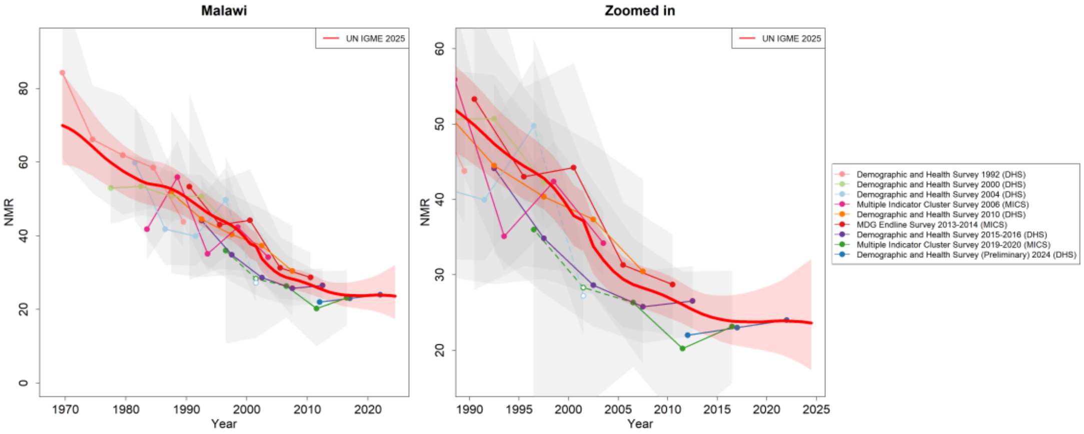

## Malaysia (MYS)

### Under-five mortality rate

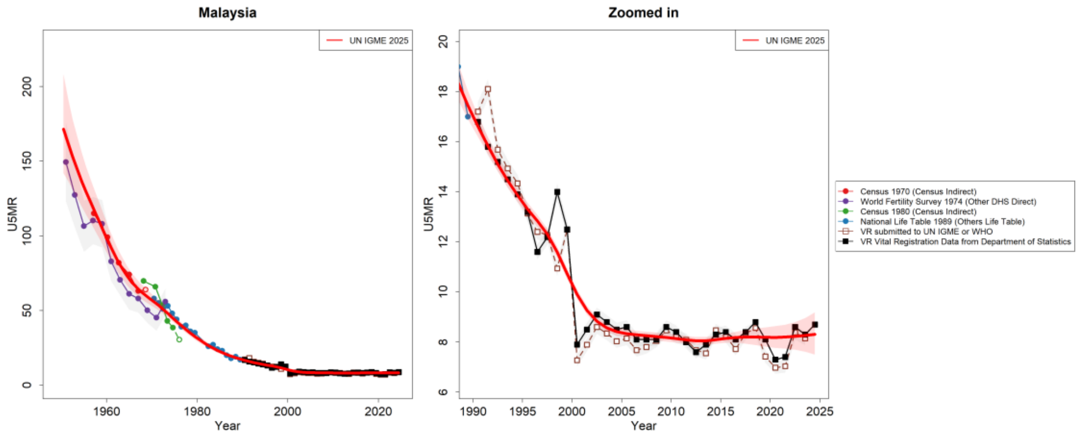


### Infant mortality rate

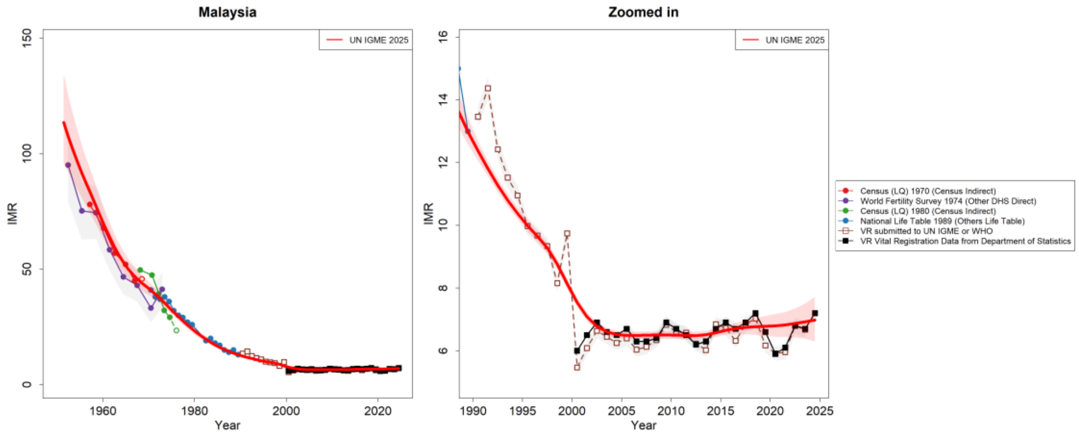


### Neonatal mortality rate

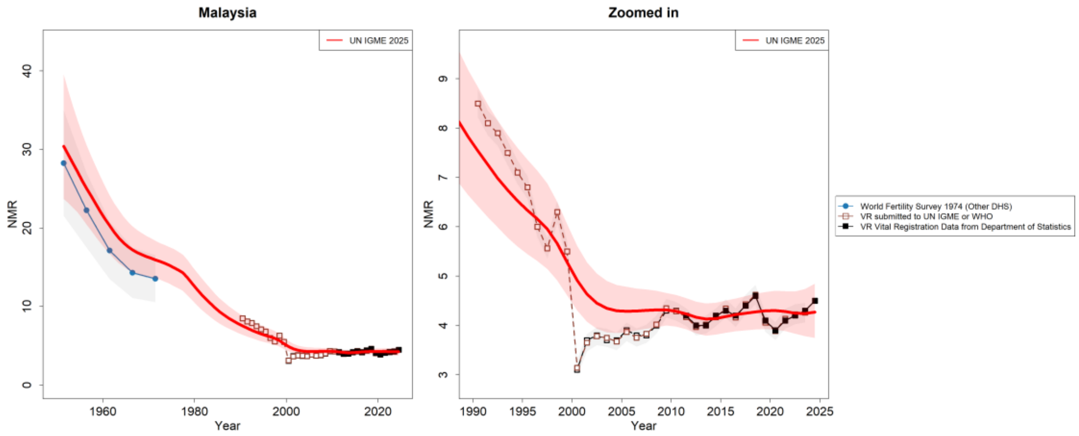

## Maldives (MDV)

### Under-five mortality rate

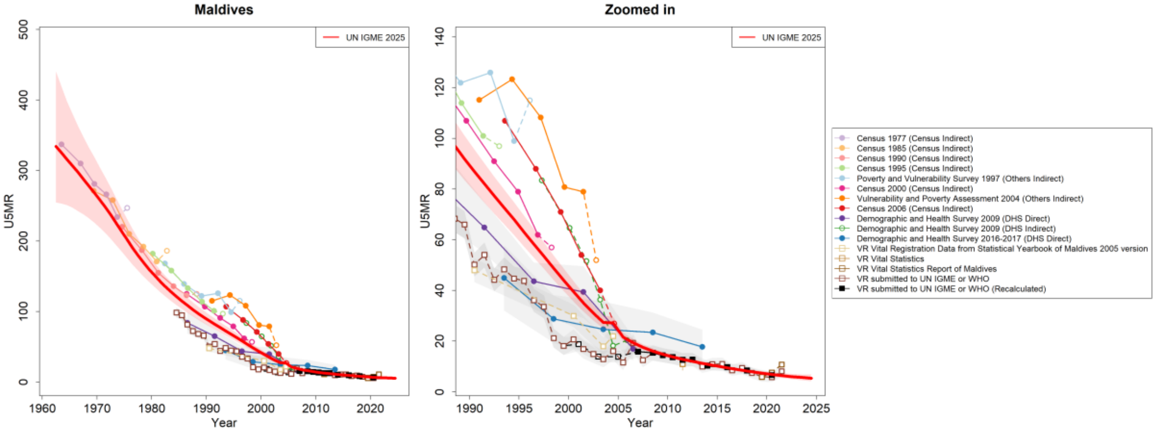


### Infant mortality rate

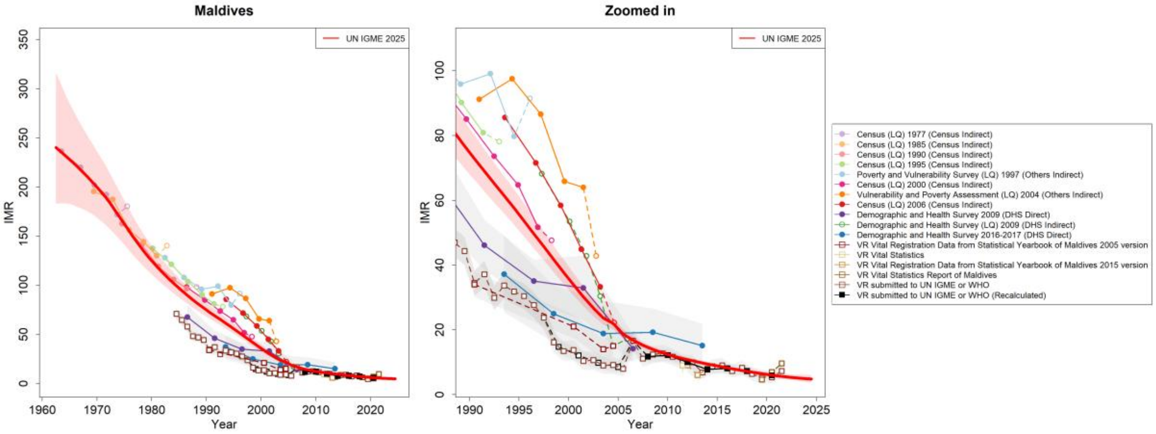


### Neonatal mortality rate

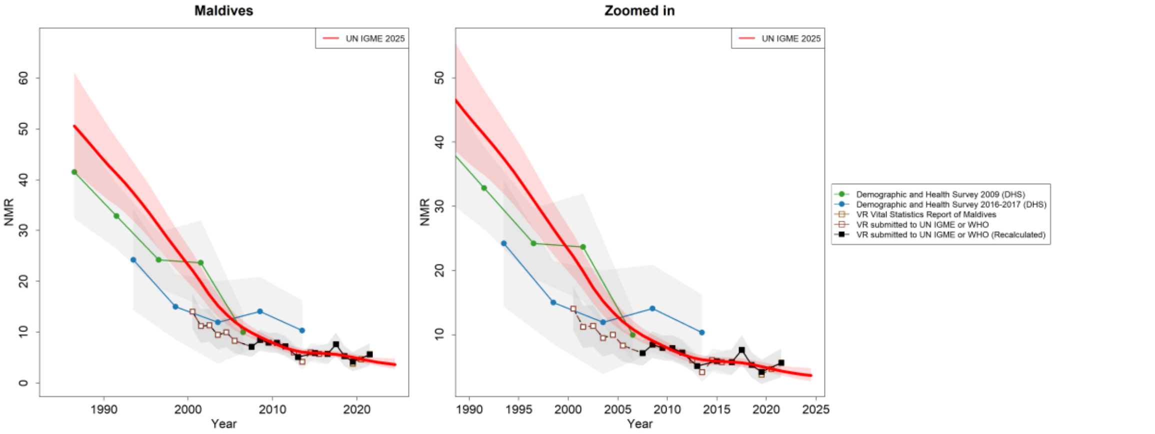

## Mali (MLI)

### Under-five mortality rate

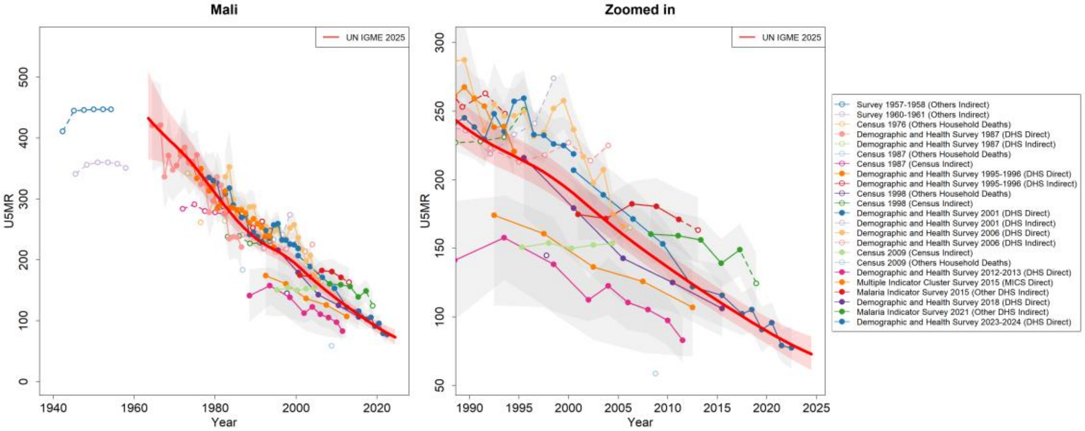


### Infant mortality rate

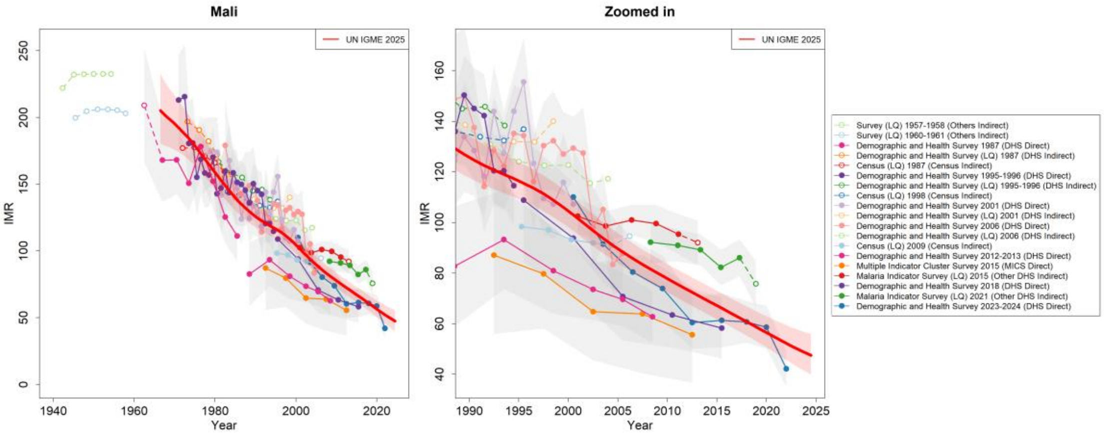


### Neonatal mortality rate

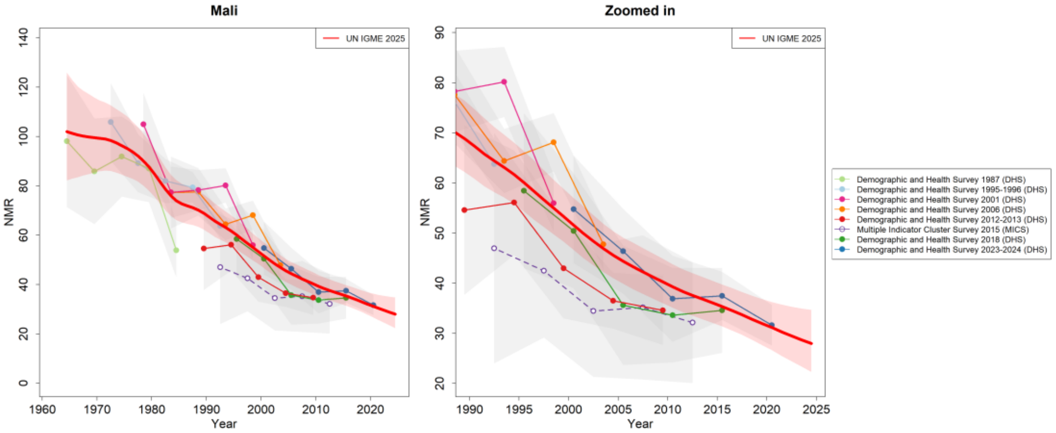

## Malta (MLT)

### Under-five mortality rate

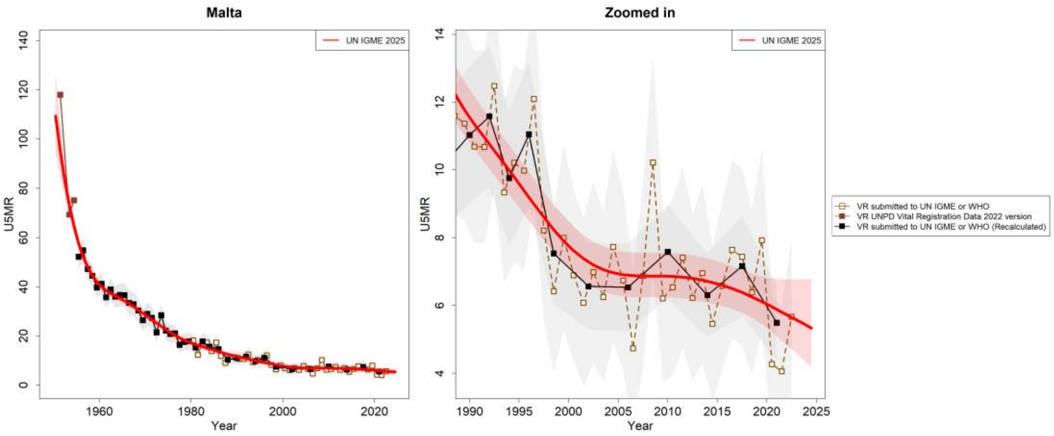


### Infant mortality rate

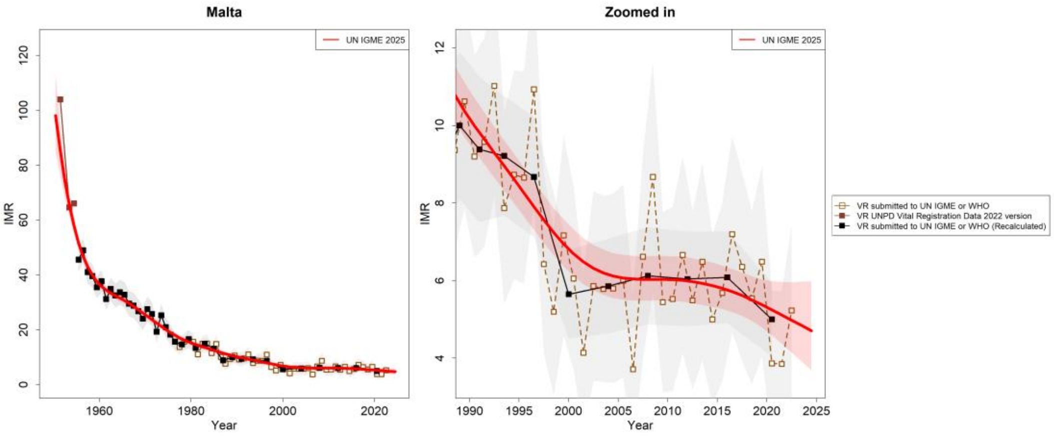


### Neonatal mortality rate

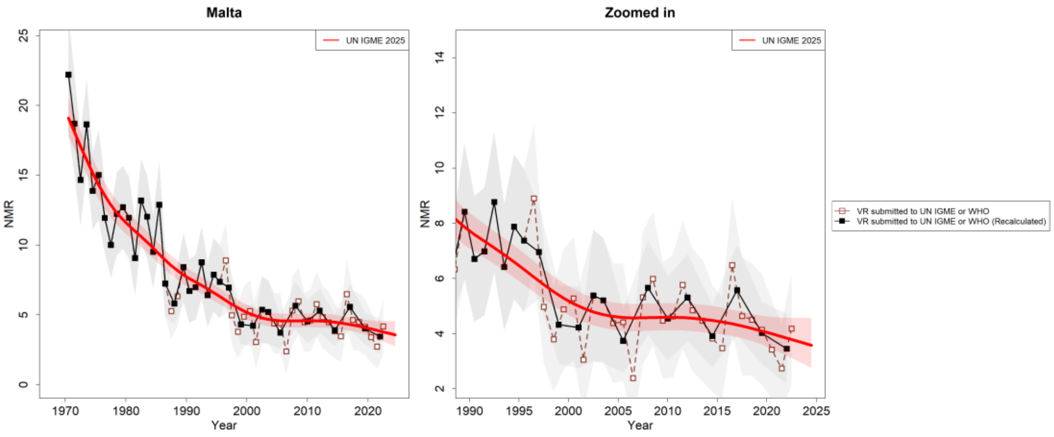

## Marshall Islands (MHL)

### Under-five mortality rate

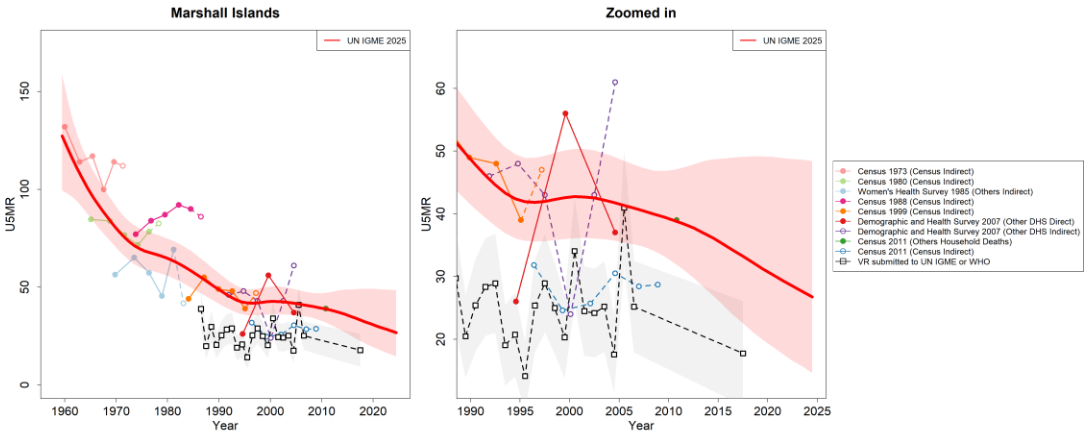


### Infant mortality rate

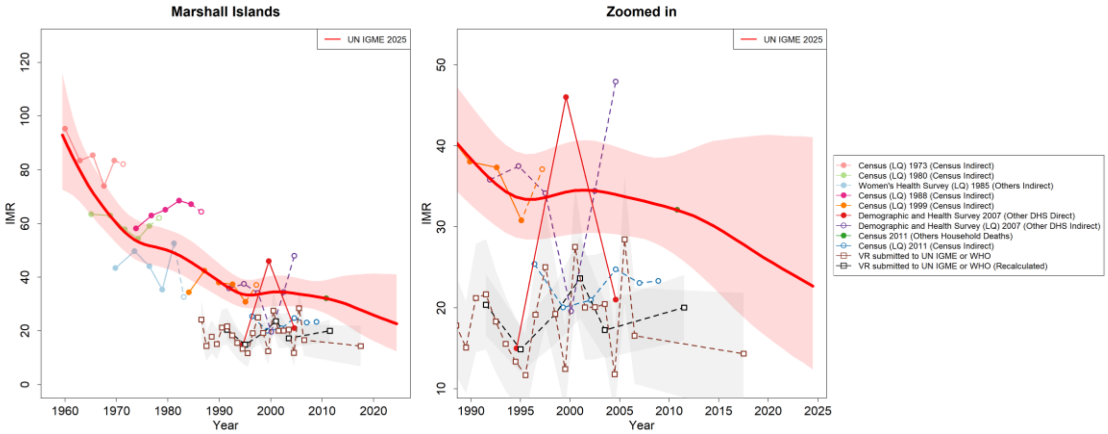


### Neonatal mortality rate

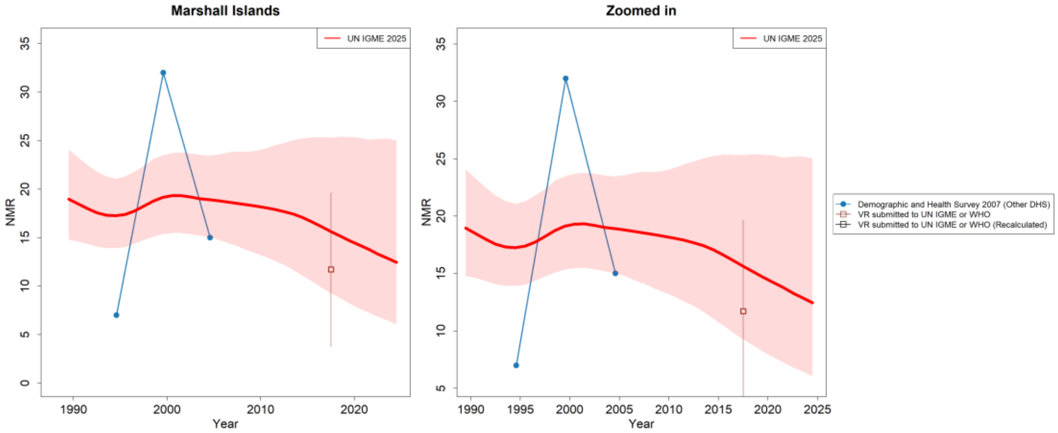

## Mauritania (MRT)

### Under-five mortality rate

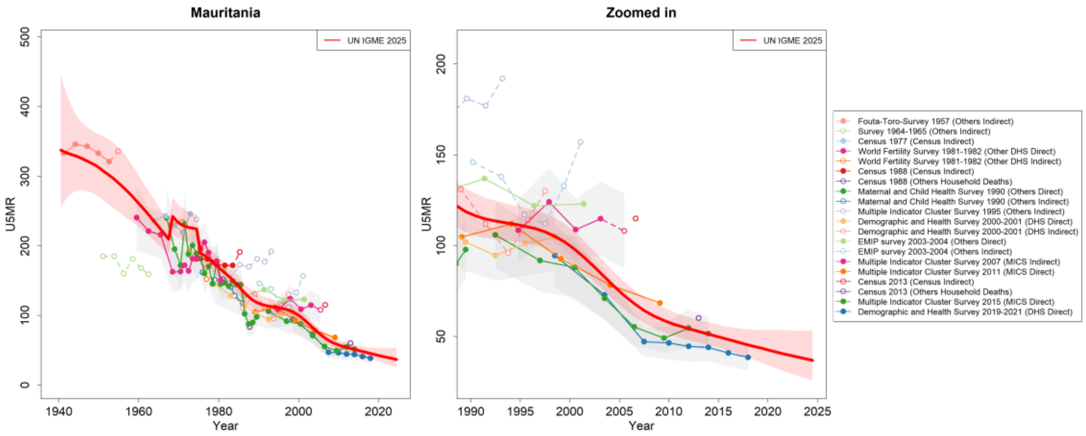


### Infant mortality rate

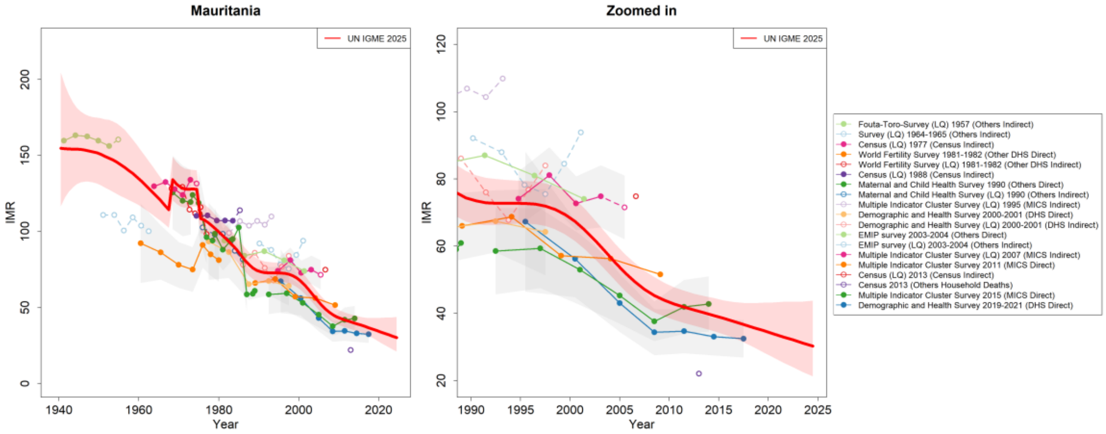


### Neonatal mortality rate

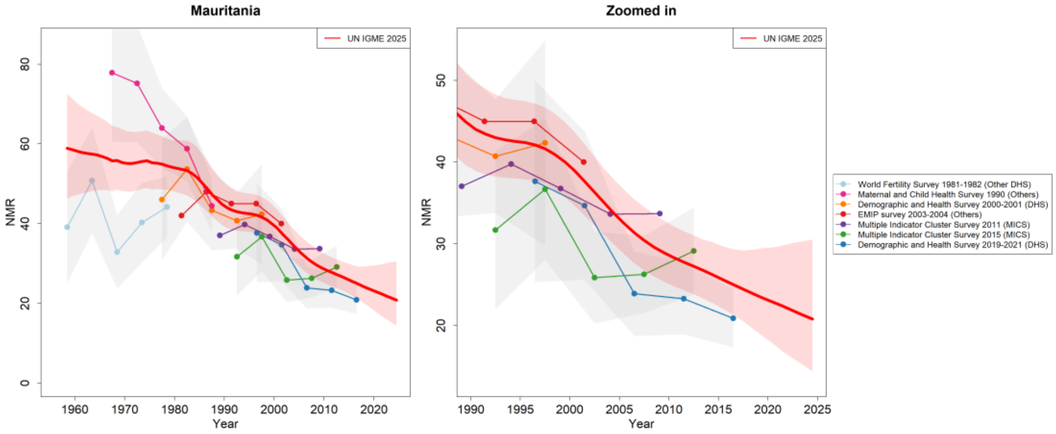

## Mauritius (MUS)

### Under-five mortality rate

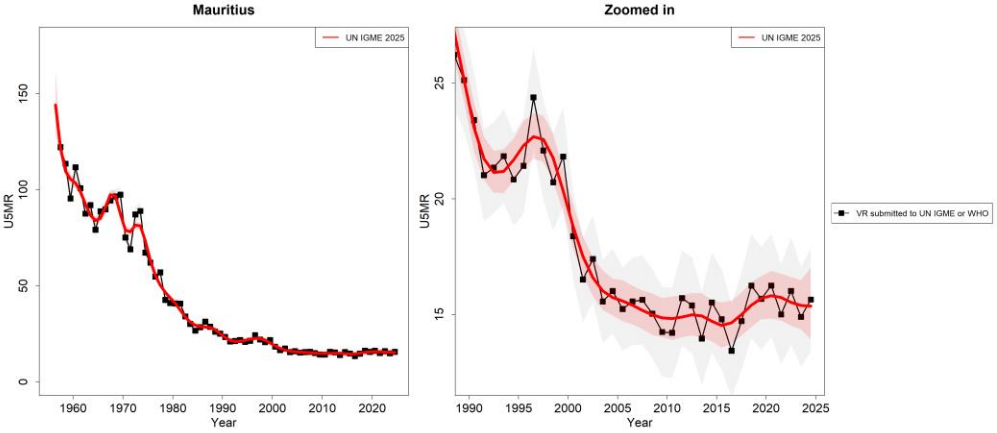


### Infant mortality rate

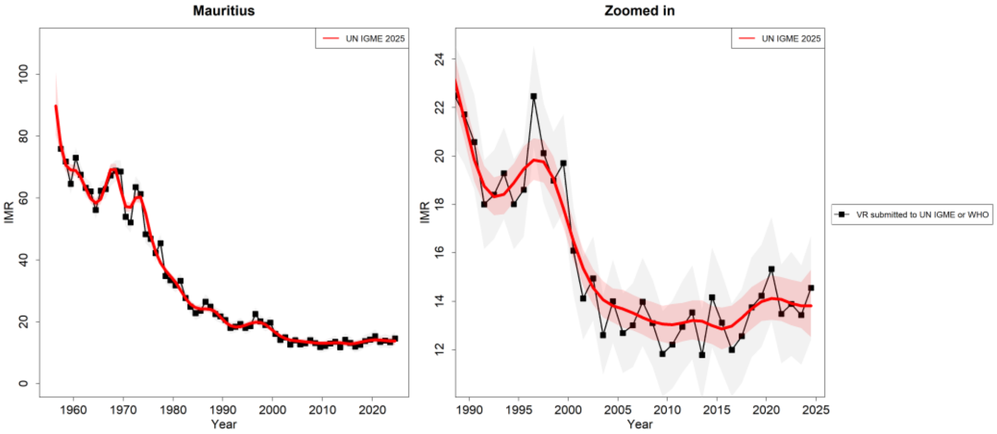


### Neonatal mortality rate

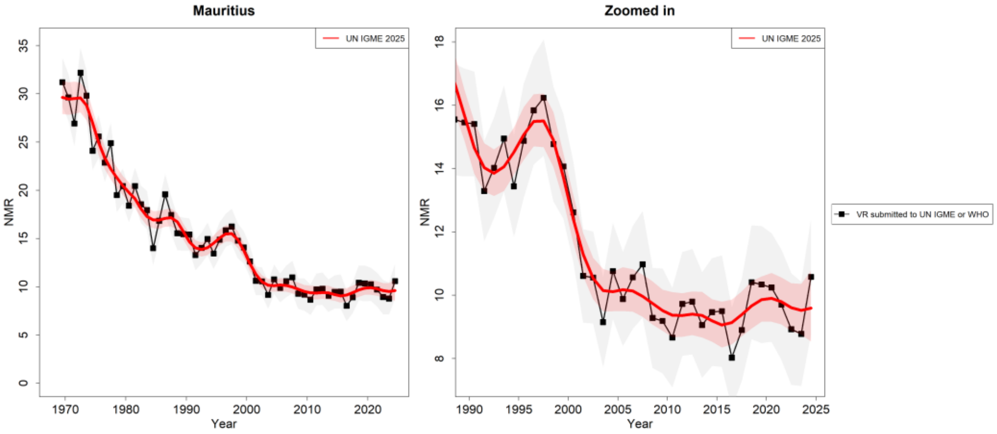

## Mexico (MEX)

### Under-five mortality rate

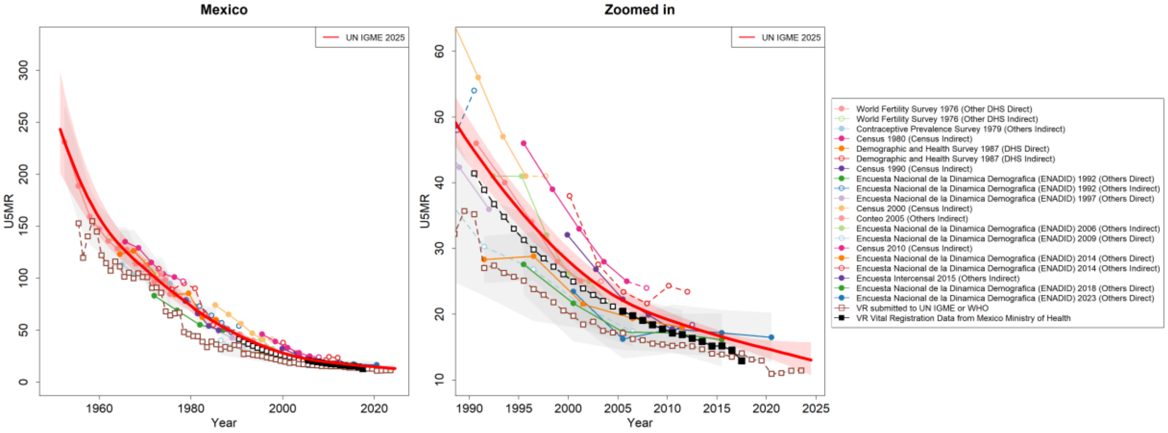


### Infant mortality rate

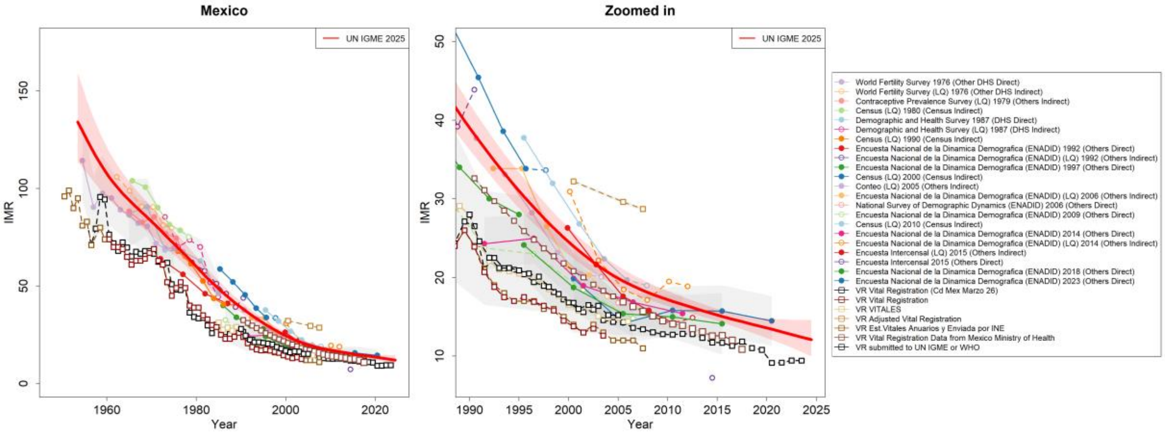


### Neonatal mortality rate

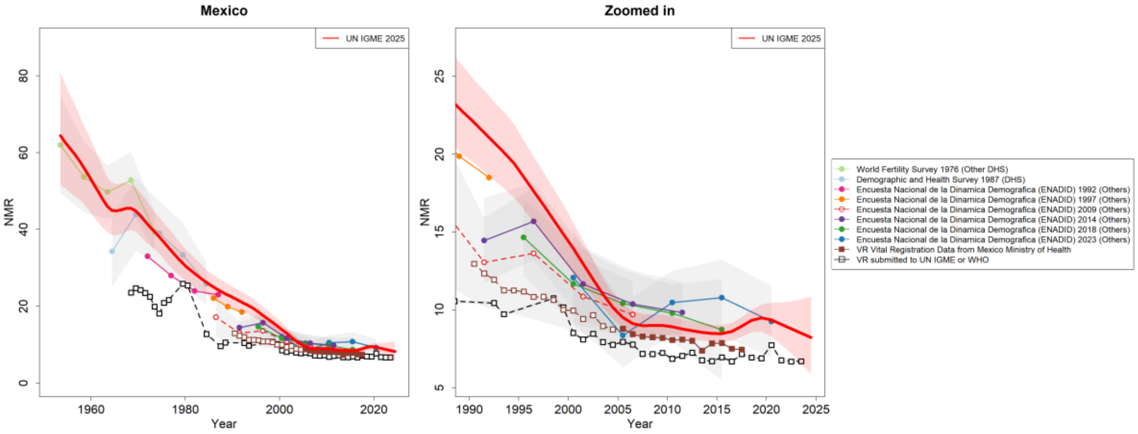

## Micronesia (Federated States of) (FSM)

### Under-five mortality rate

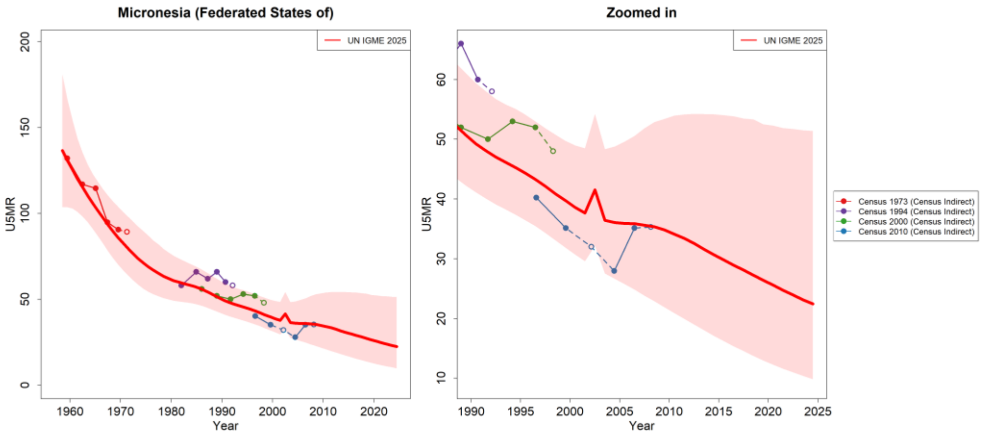


### Infant mortality rate

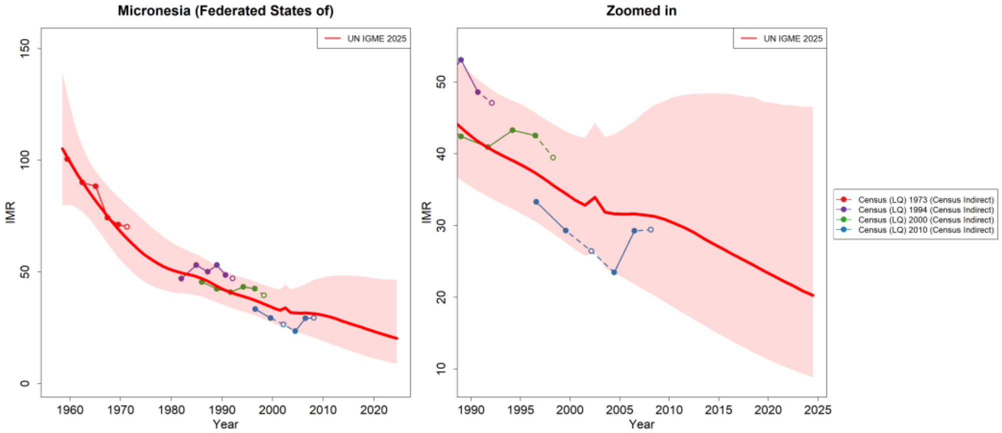


### Neonatal mortality rate

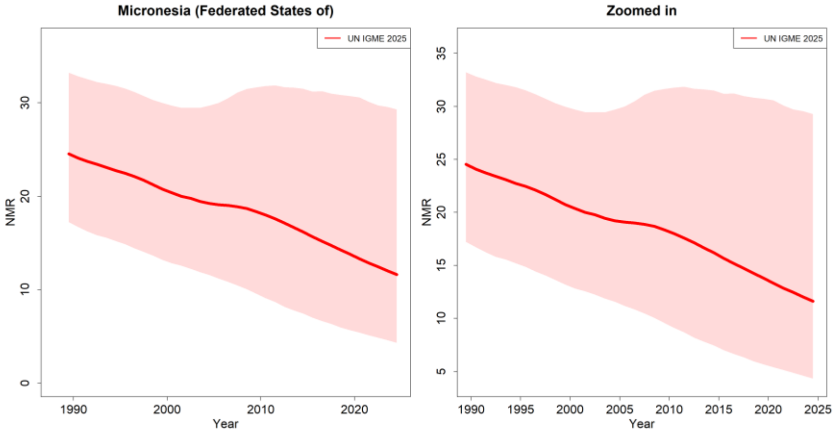

## Monaco (MCO)

### Under-five mortality rate

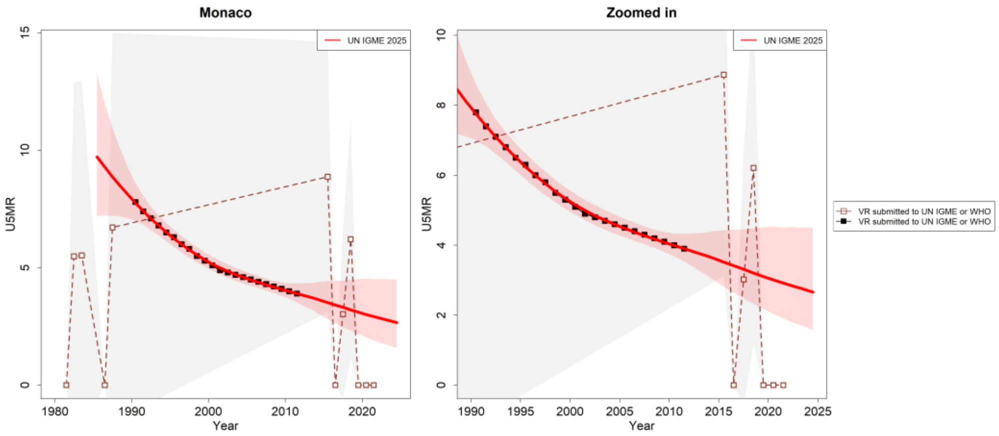


### Infant mortality rate

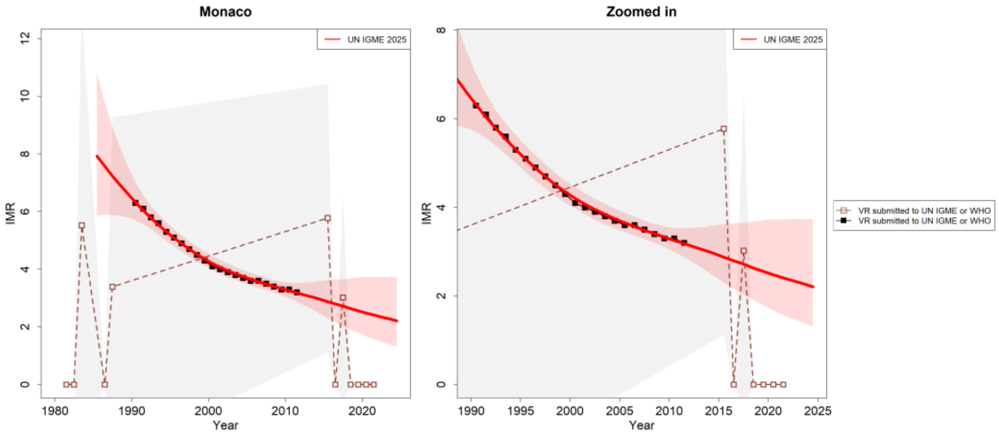


### Neonatal mortality rate

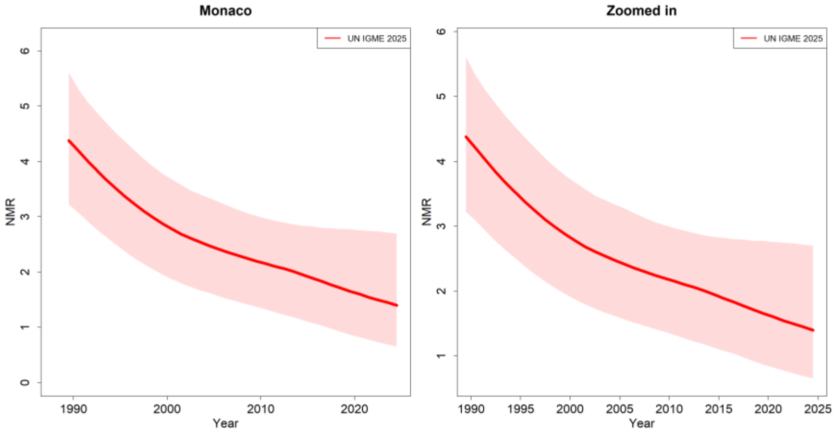

## Mongolia (MNG)

### Under-five mortality rate

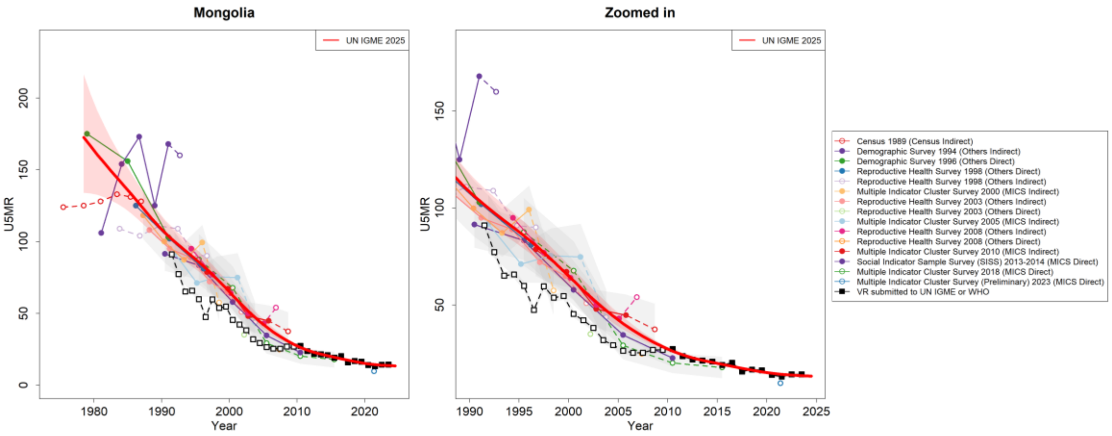


### Infant mortality rate

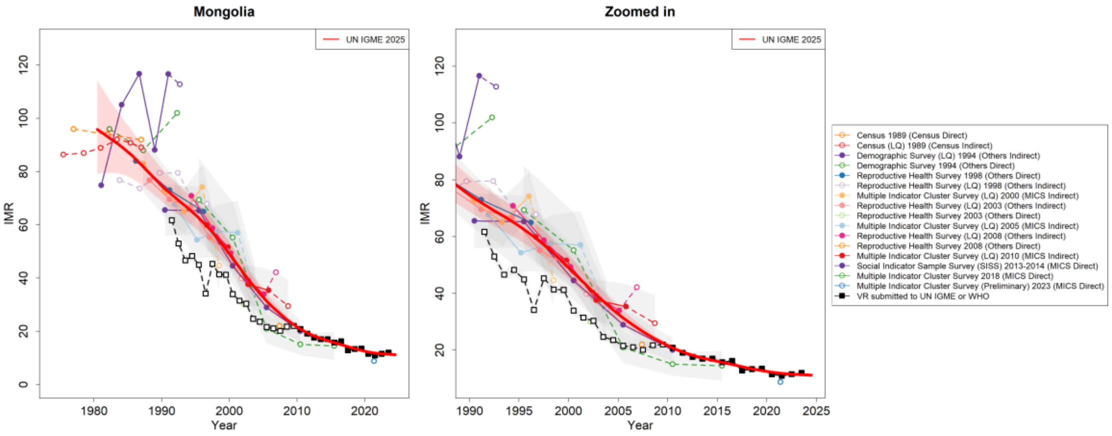


### Neonatal mortality rate

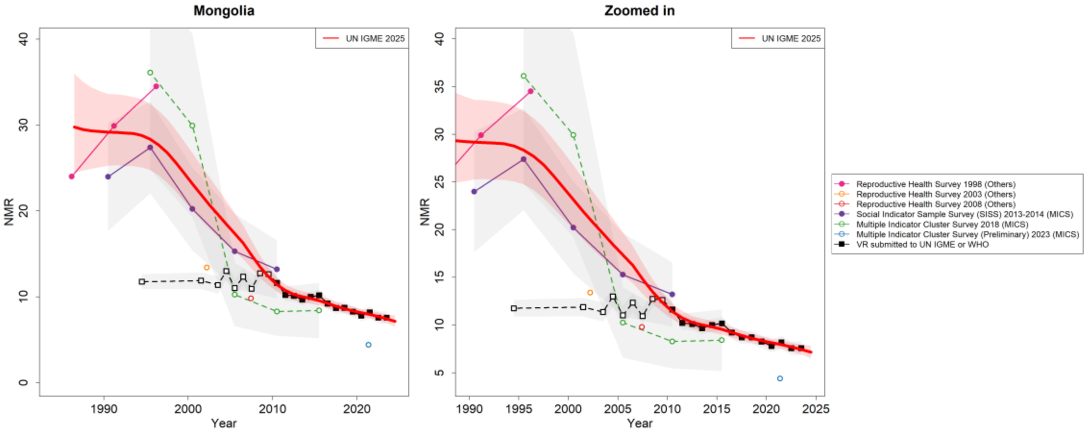

## Montenegro (MNE)

### Under-five mortality rate

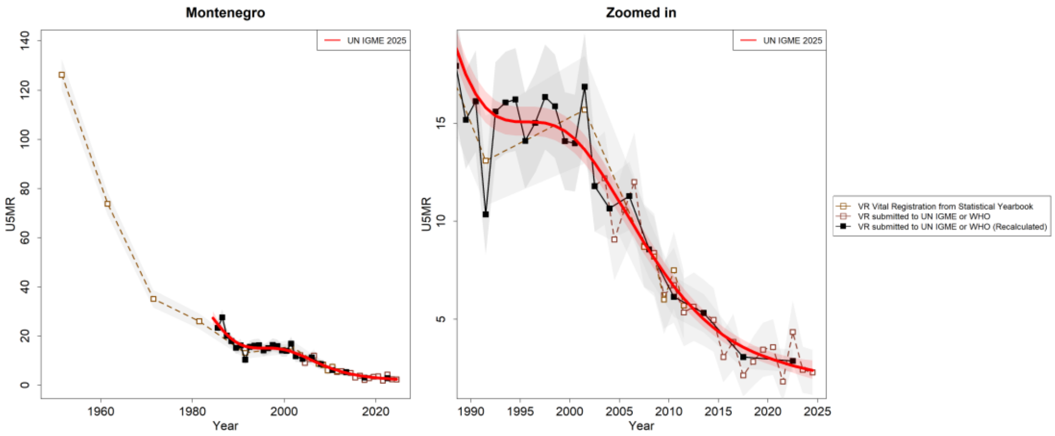


### Infant mortality rate

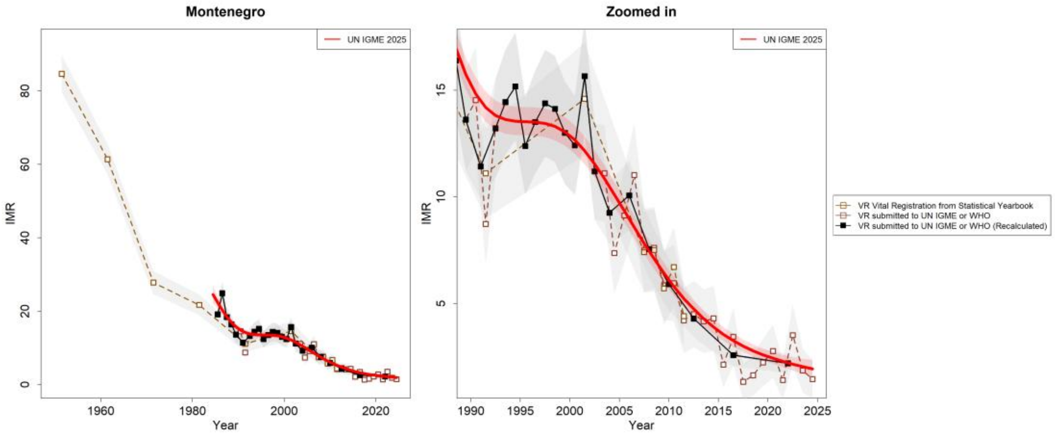


### Neonatal mortality rate

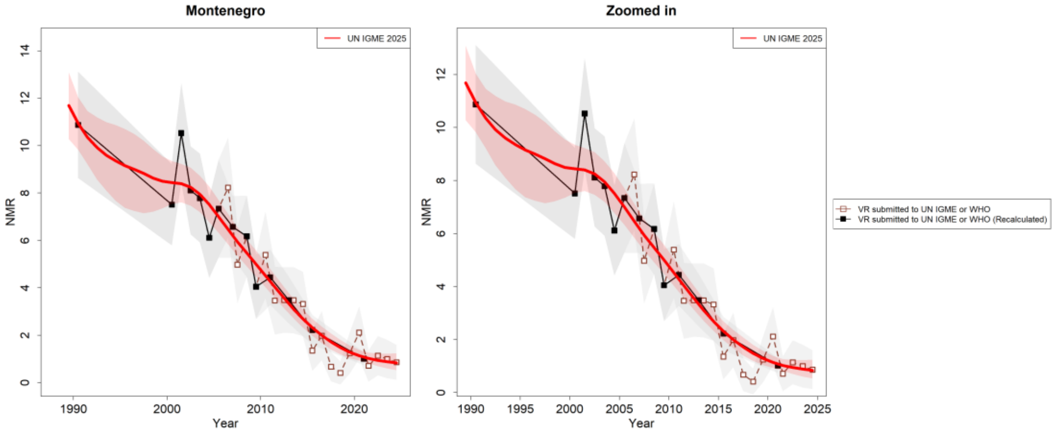

# Montserrat (MSR)

## Under-five mortality rate

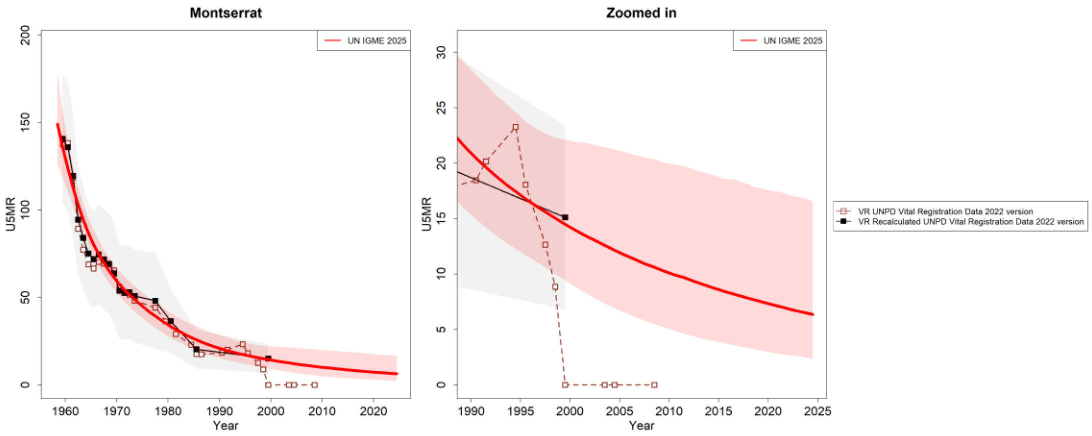


## Infant mortality rate

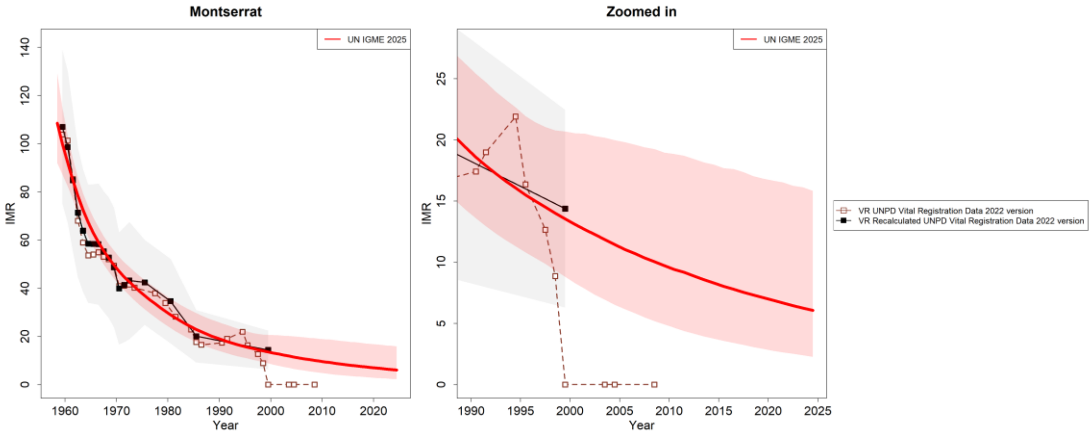


## Neonatal mortality rate

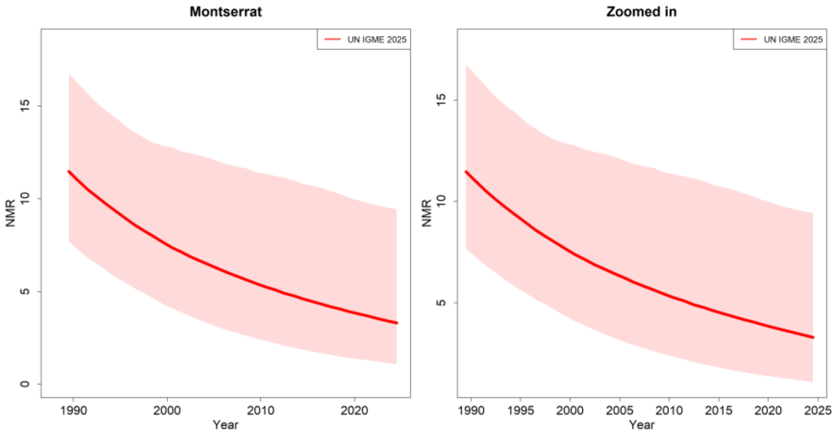

## Morocco (MAR)

### Under-five mortality rate

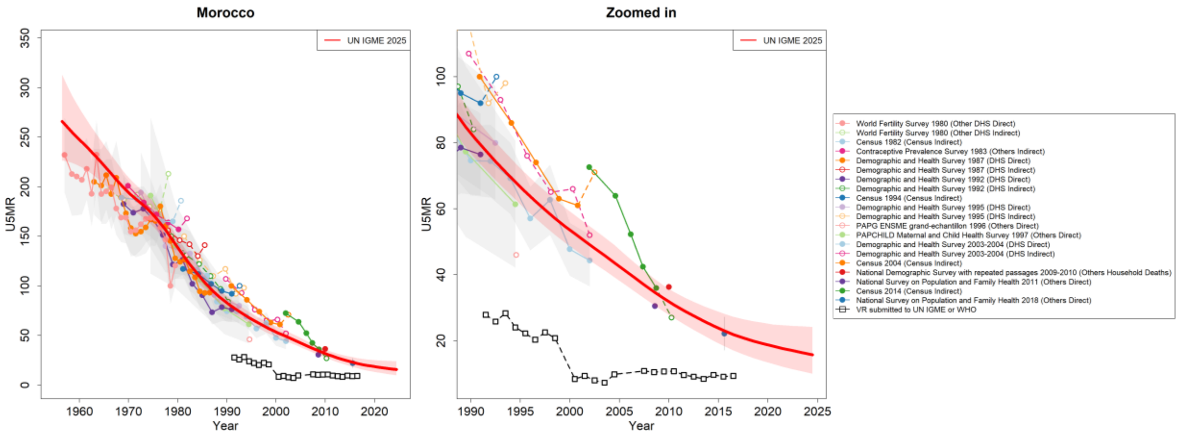


### Infant mortality rate

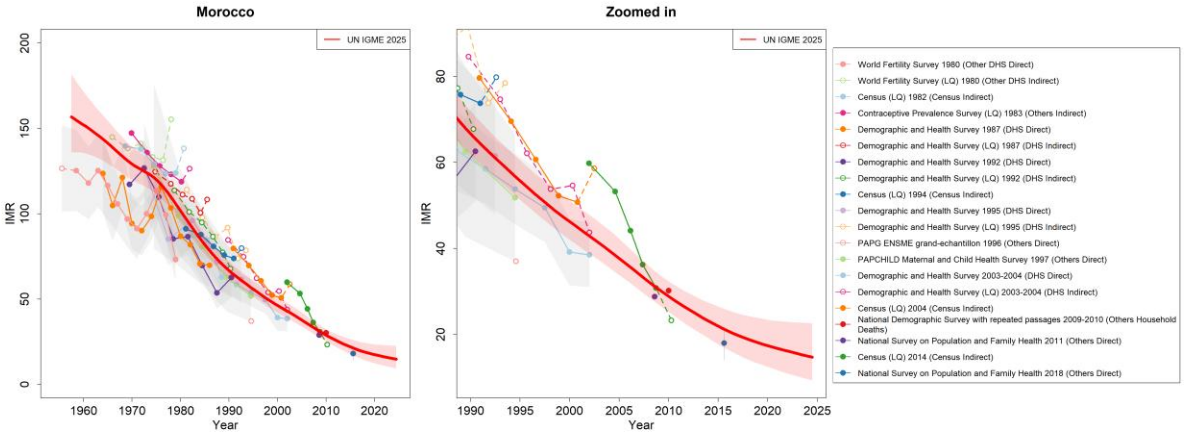


### Neonatal mortality rate

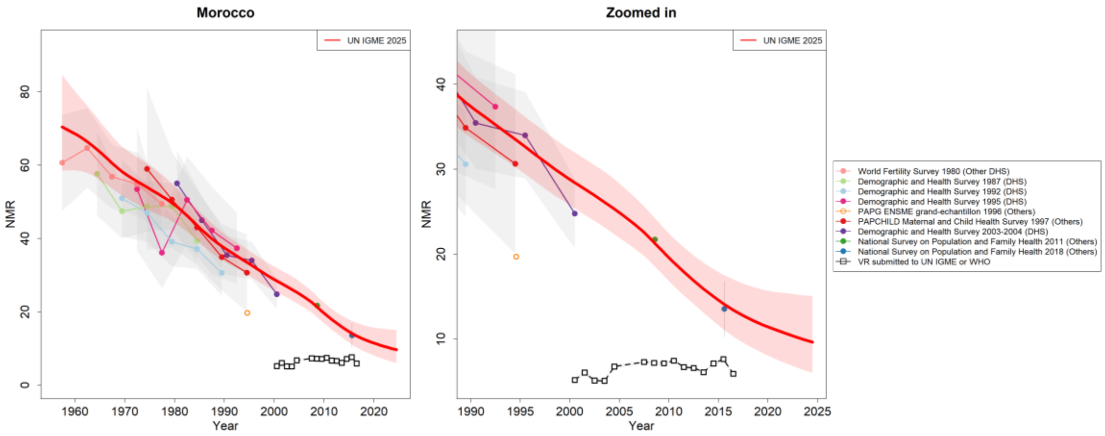

## Mozambique (MOZ)

### Under-five mortality rate

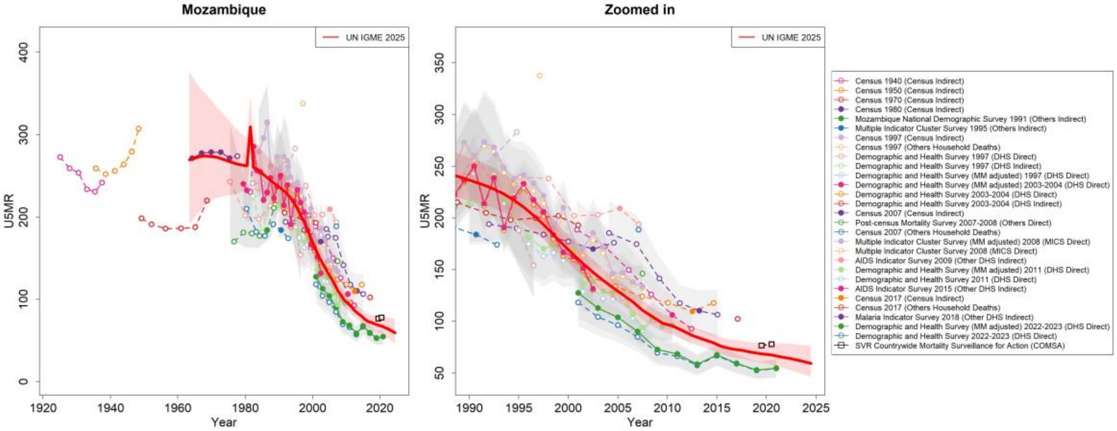


### Infant mortality rate

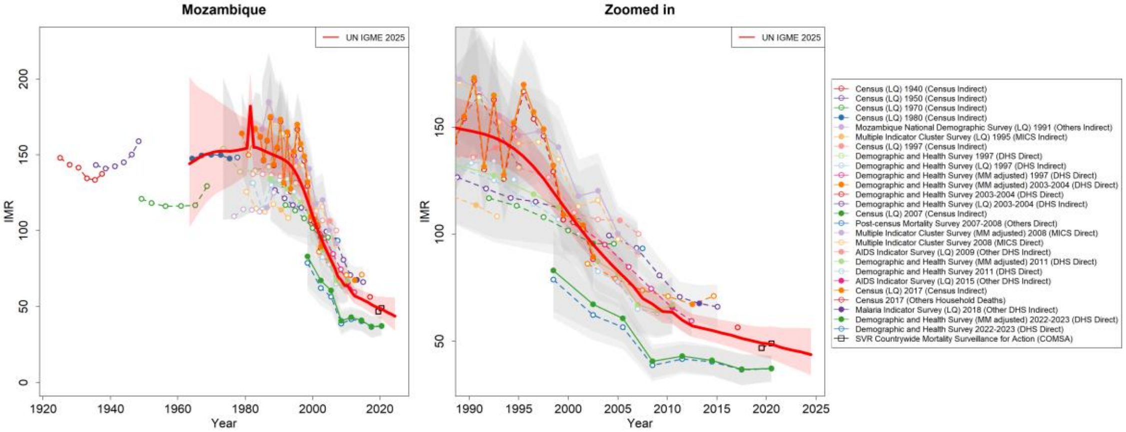


### Neonatal mortality rate

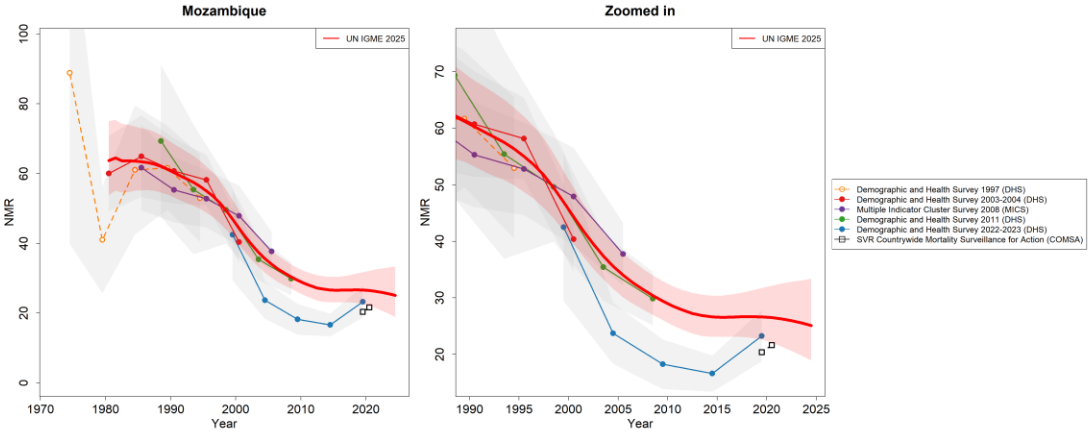

## Myanmar (MMR)

### Under-five mortality rate

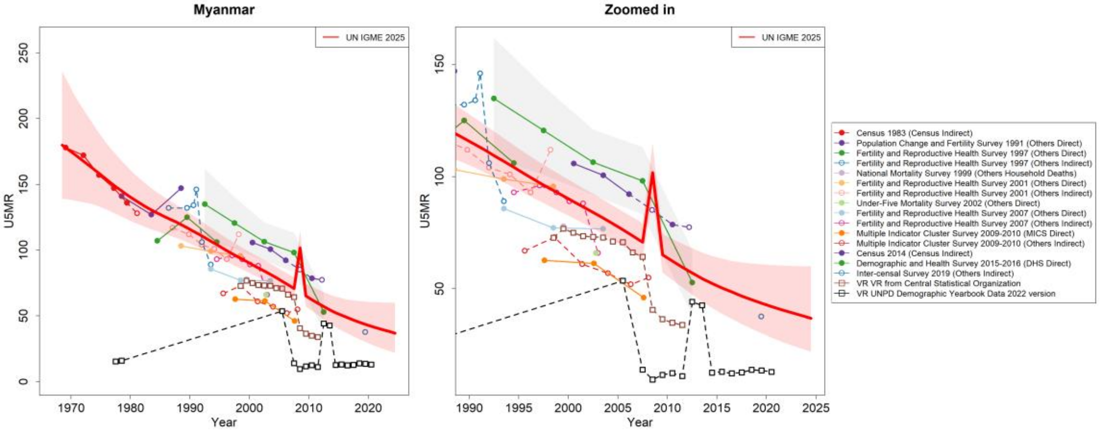


### Infant mortality rate

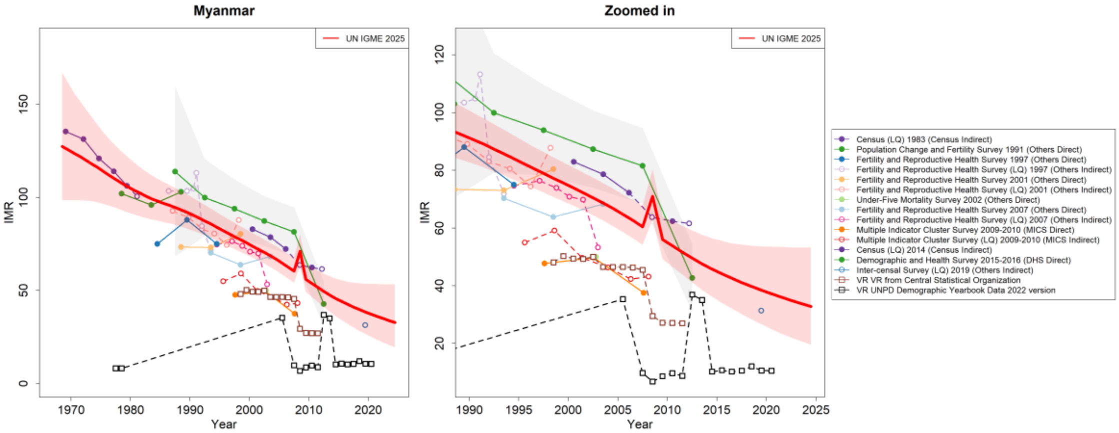


### Neonatal mortality rate

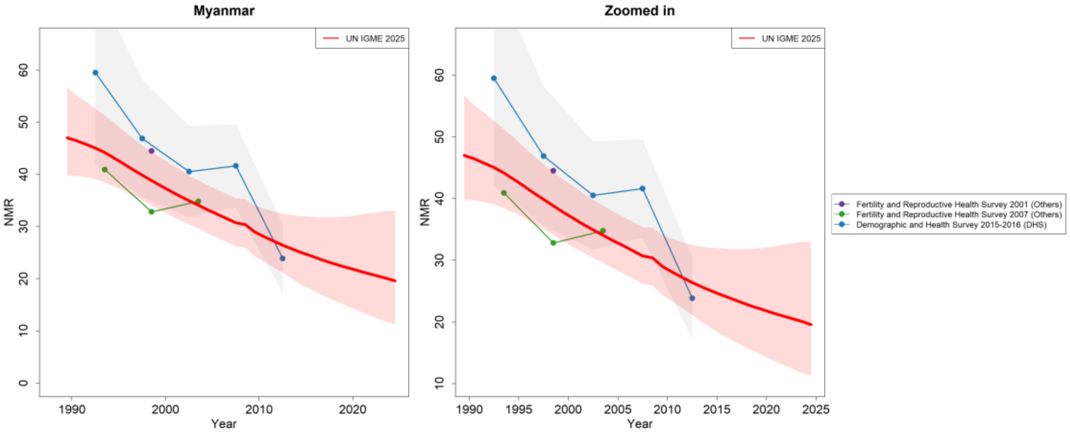

## Namibia (NAM)

### Under-five mortality rate

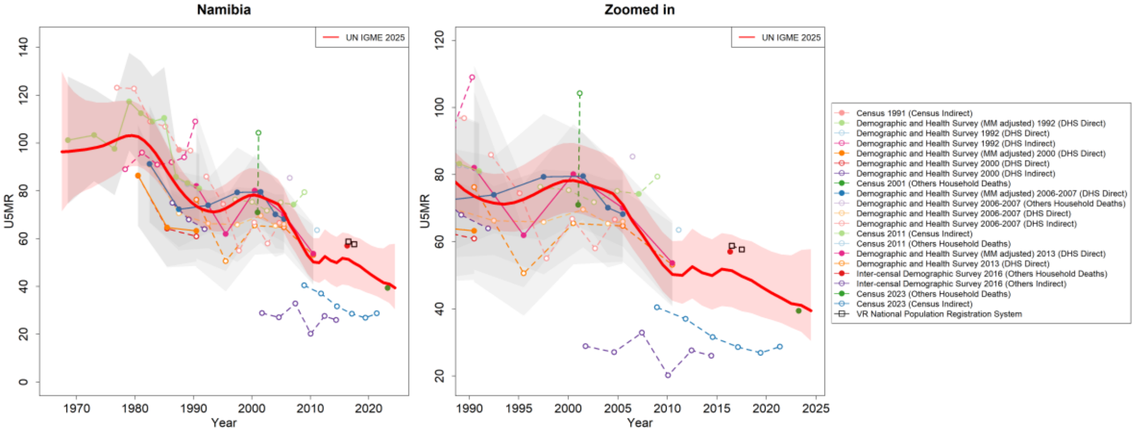


### Infant mortality rate

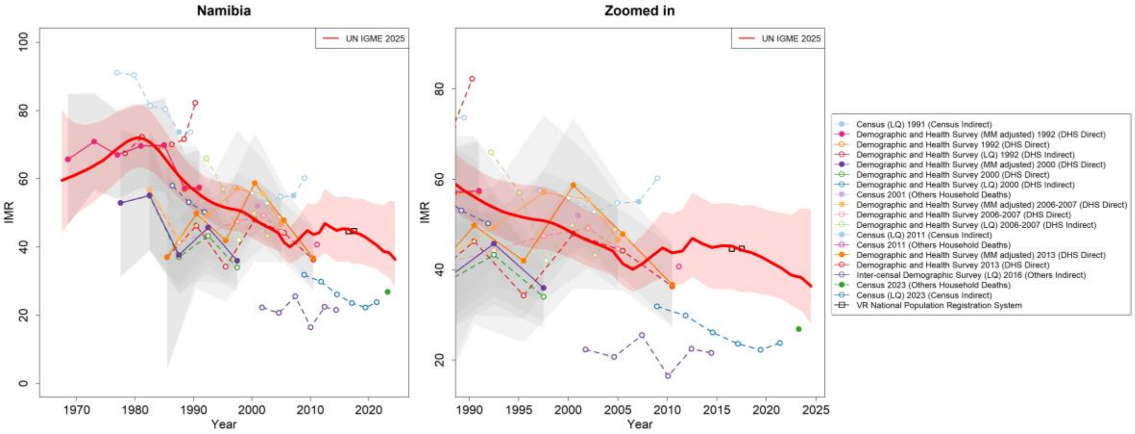


### Neonatal mortality rate

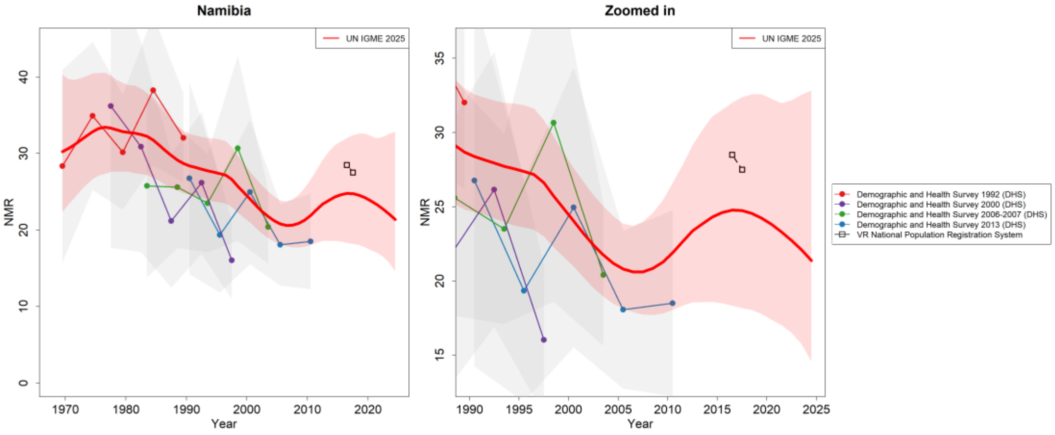

## Nauru (NRU)

### Under-five mortality rate

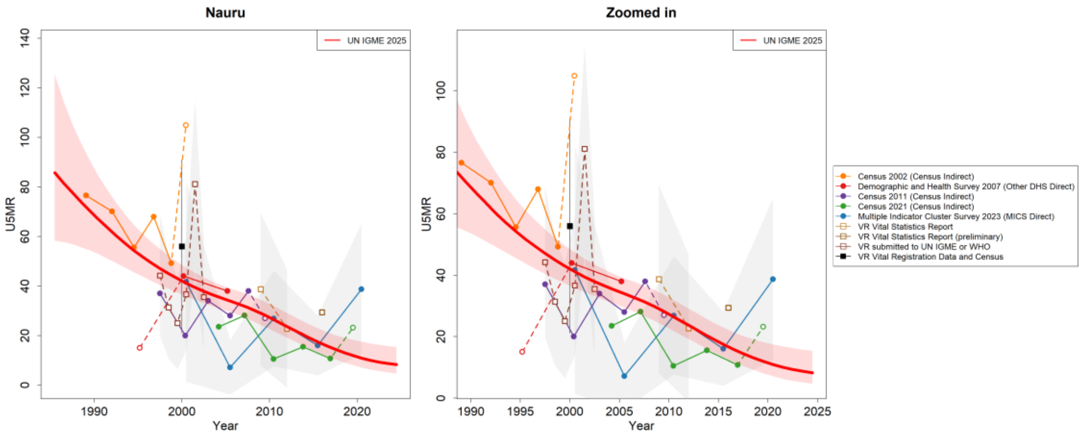


### Infant mortality rate

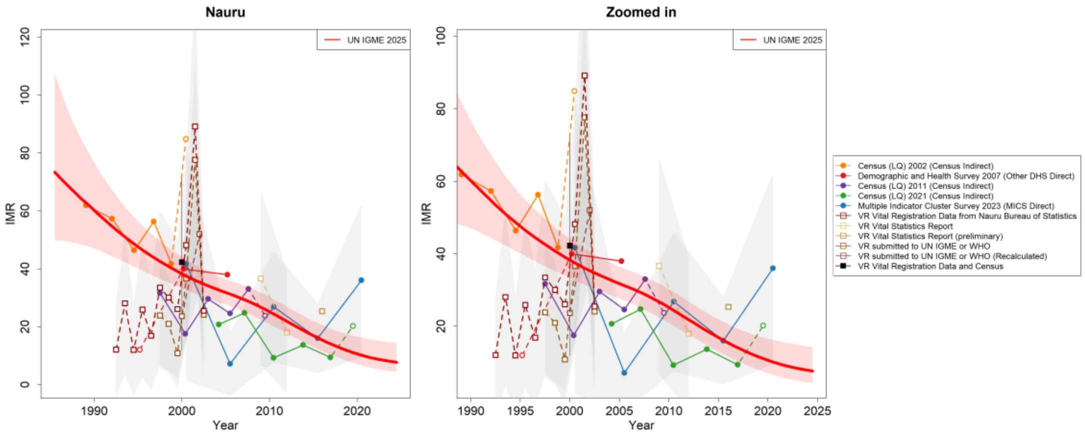


### Neonatal mortality rate

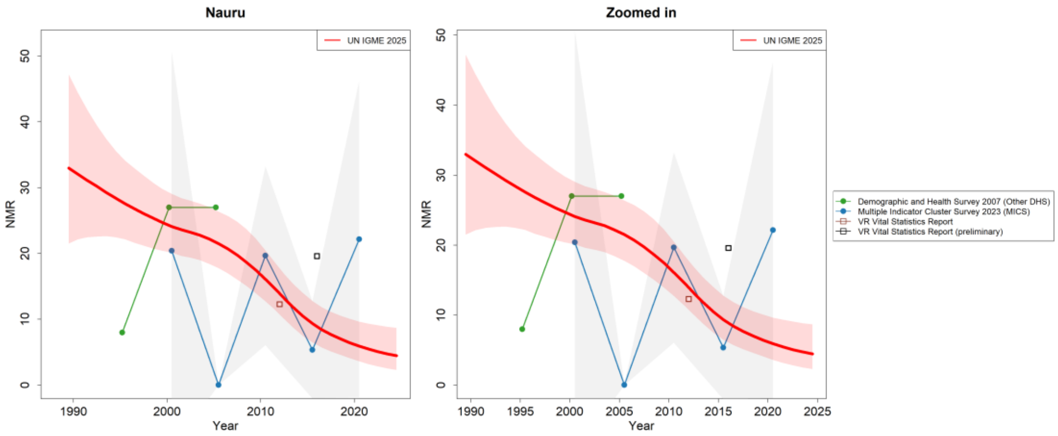

## Nepal (NPL)

### Under-five mortality rate

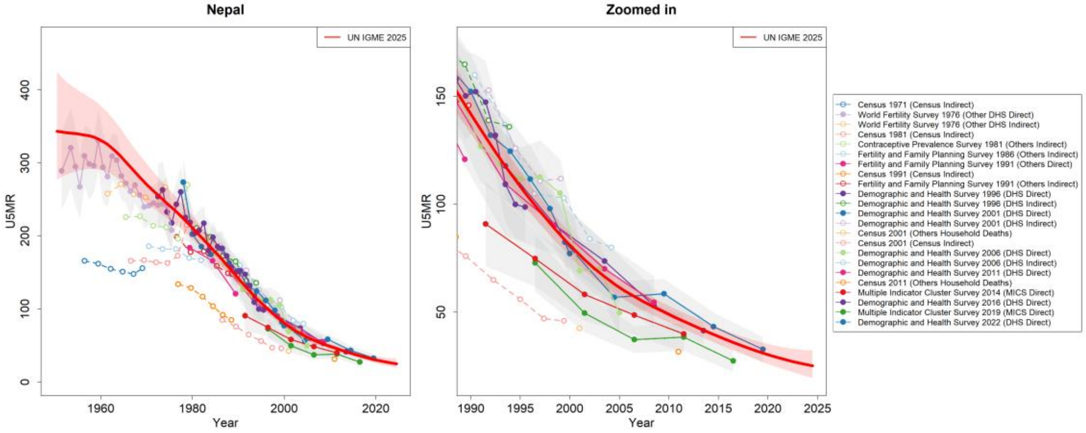


### Infant mortality rate

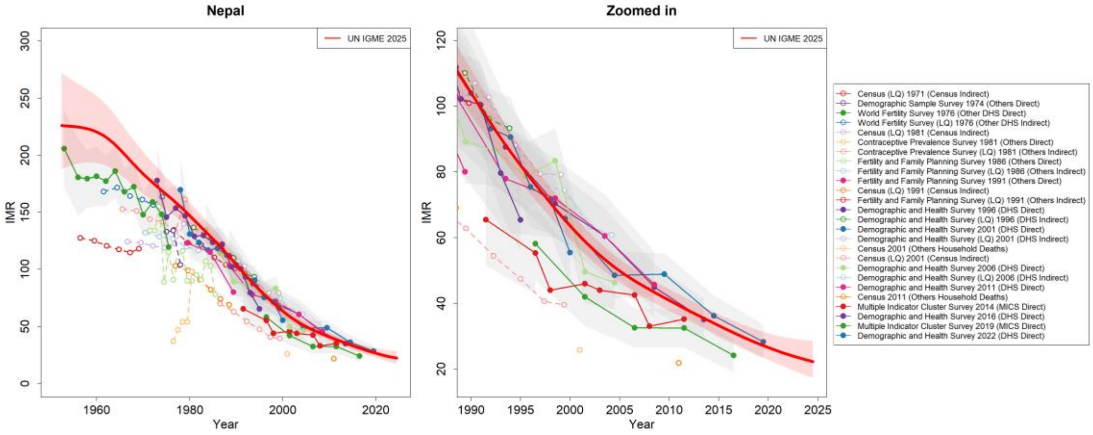


### Neonatal mortality rate

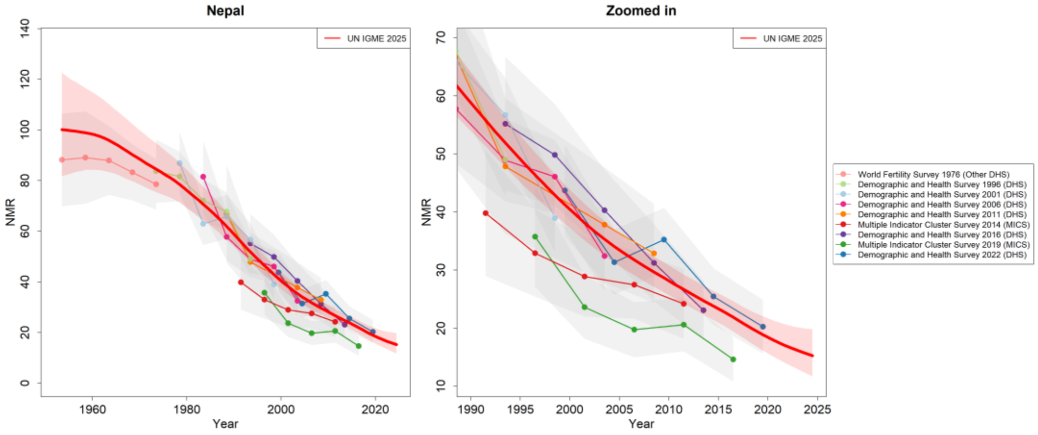

## Netherlands (Kingdom of the) (NLD)

### Under-five mortality rate

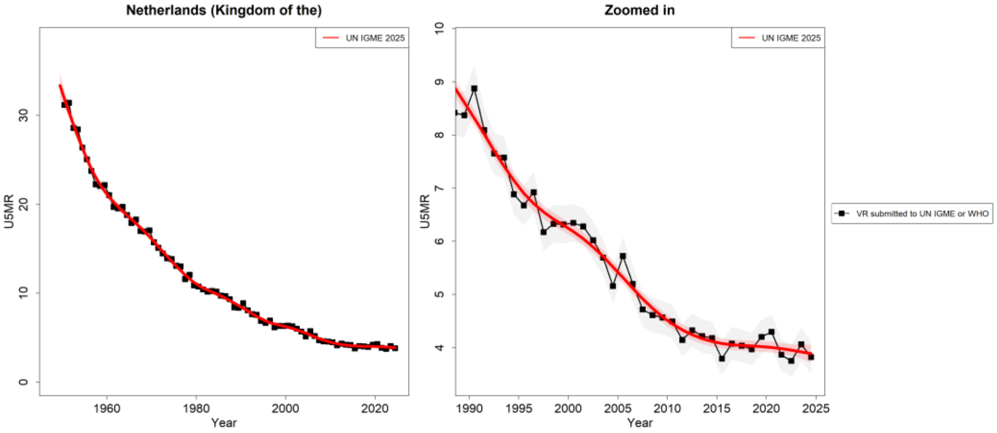


### Infant mortality rate

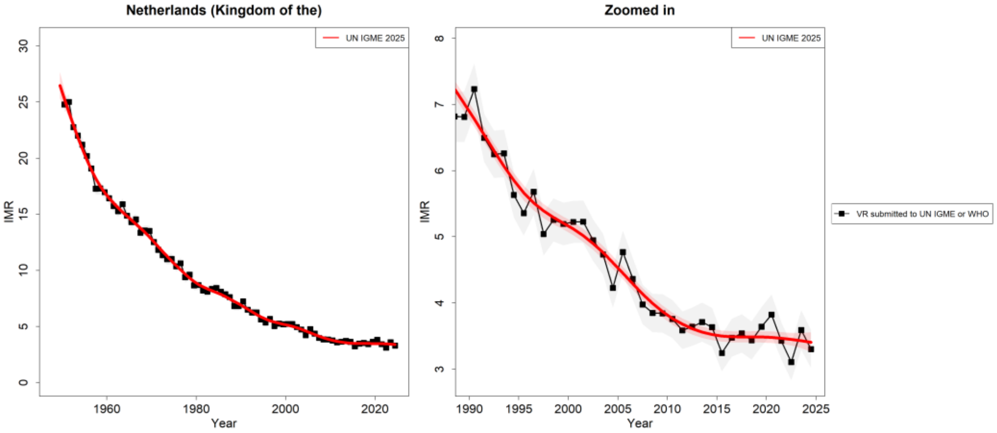


### Neonatal mortality rate

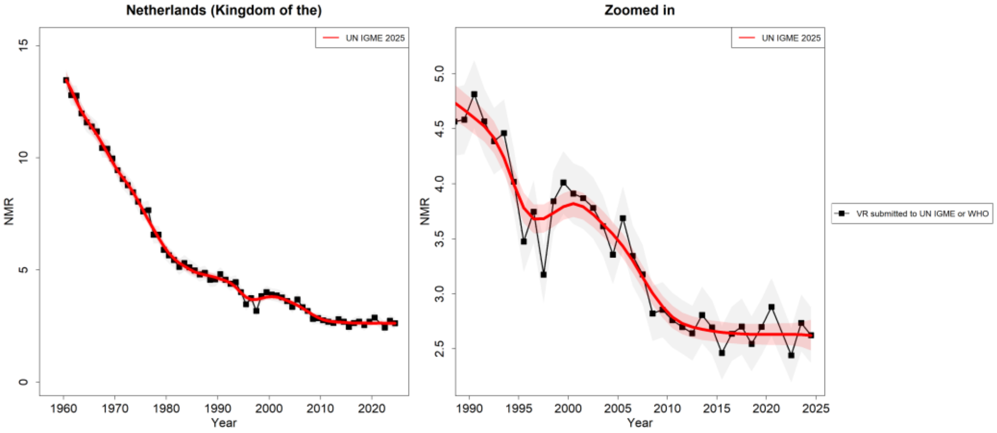

# New Zealand (NZL)

## Under-five mortality rate

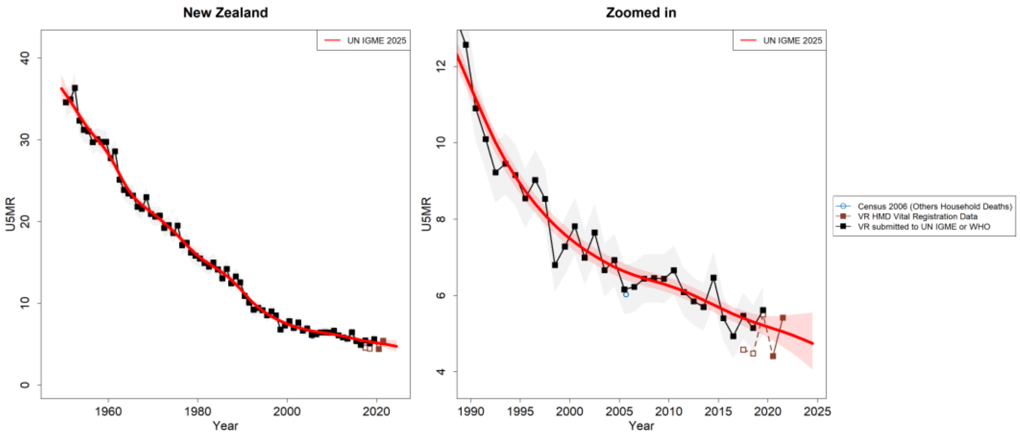


## Infant mortality rate

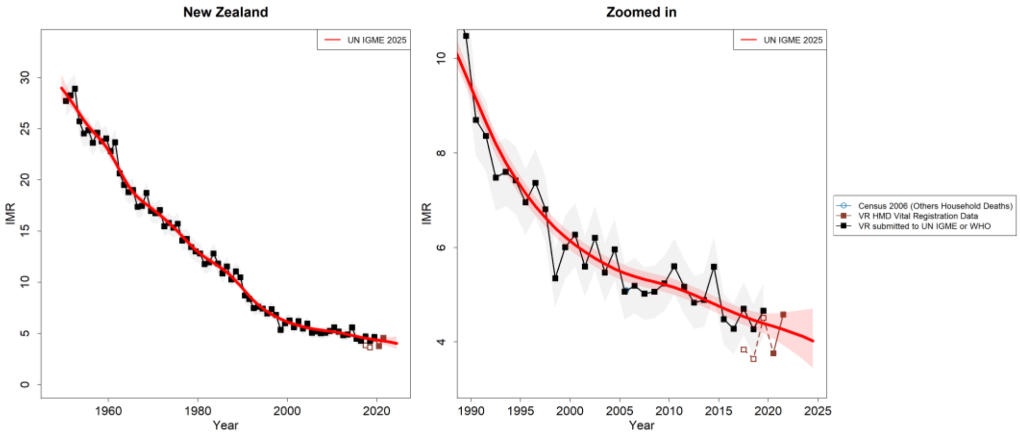


## Neonatal mortality rate

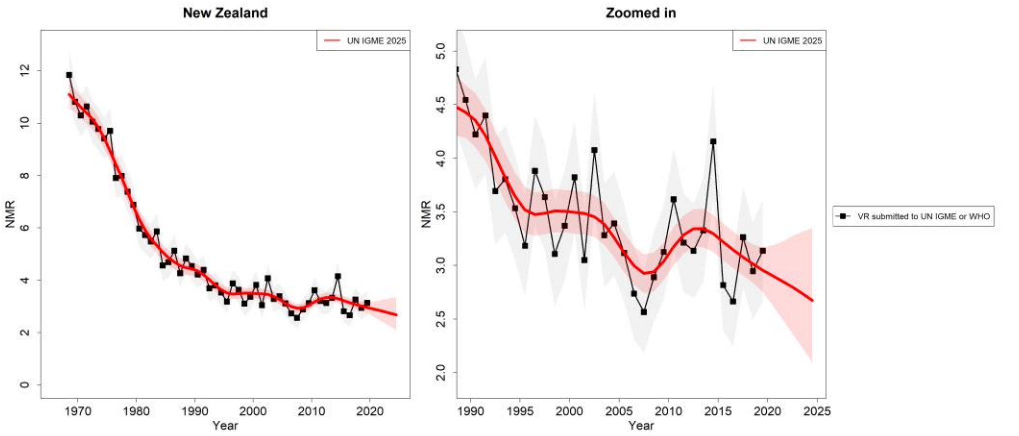

## Nicaragua (NIC)

### Under-five mortality rate

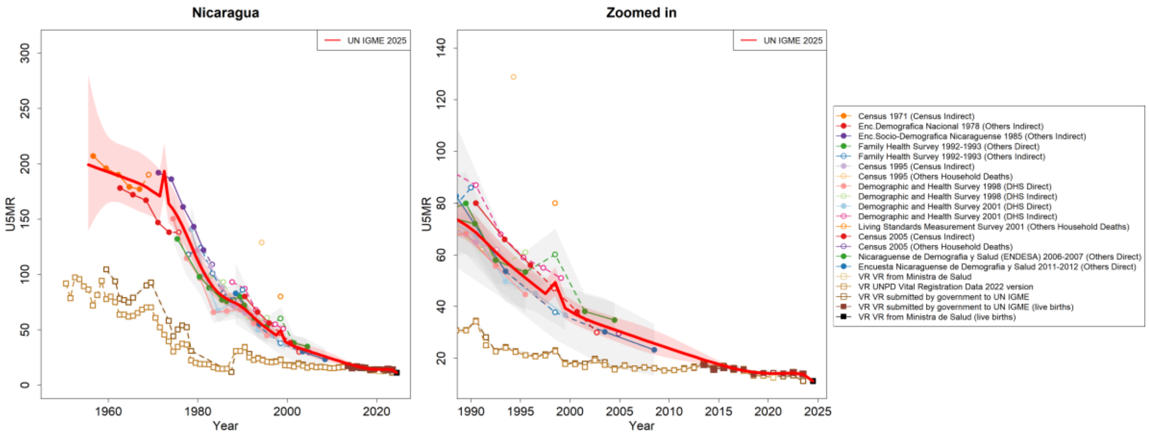


### Infant mortality rate

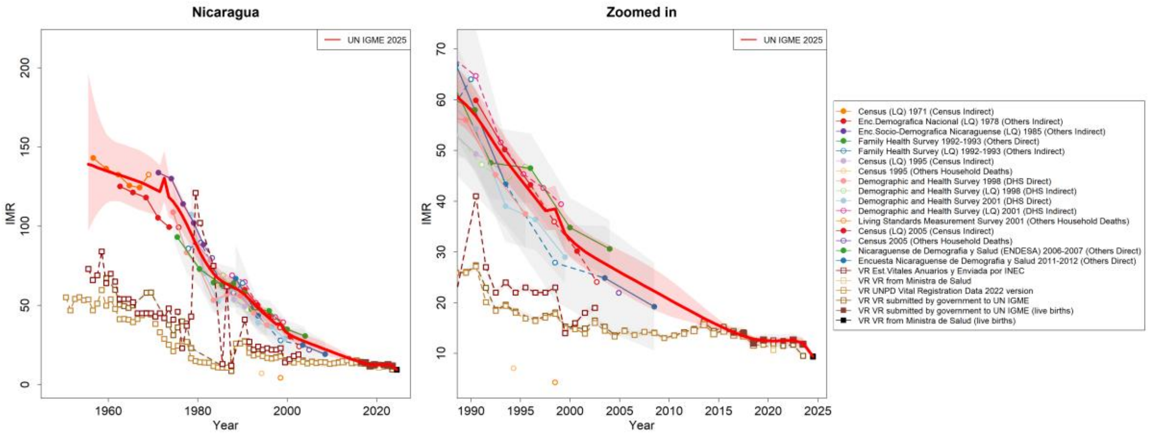


### Neonatal mortality rate

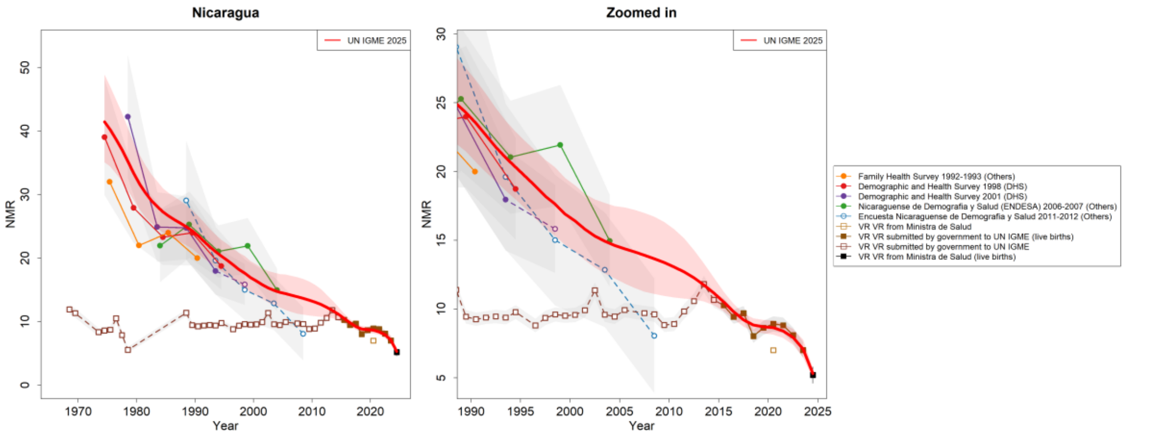

## Niger (NER)

### Under-five mortality rate

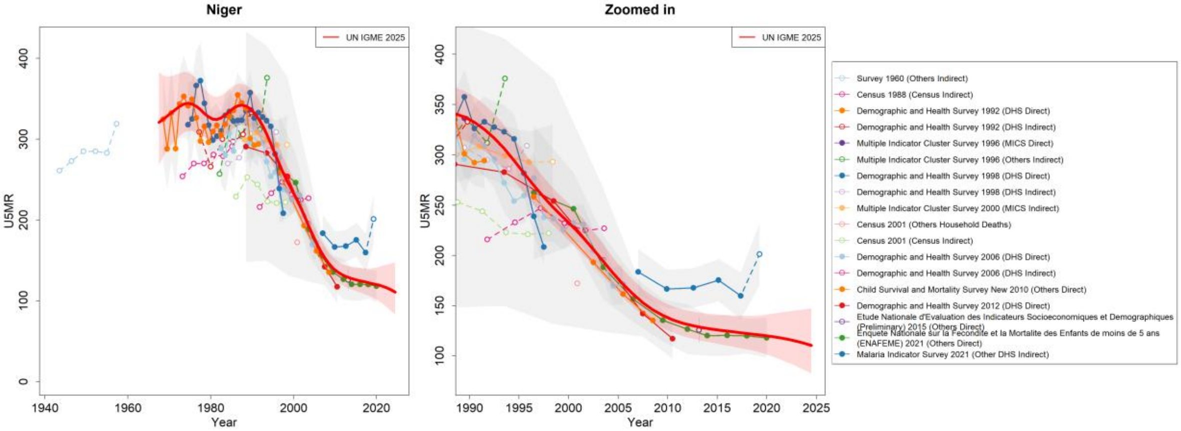


### Infant mortality rate

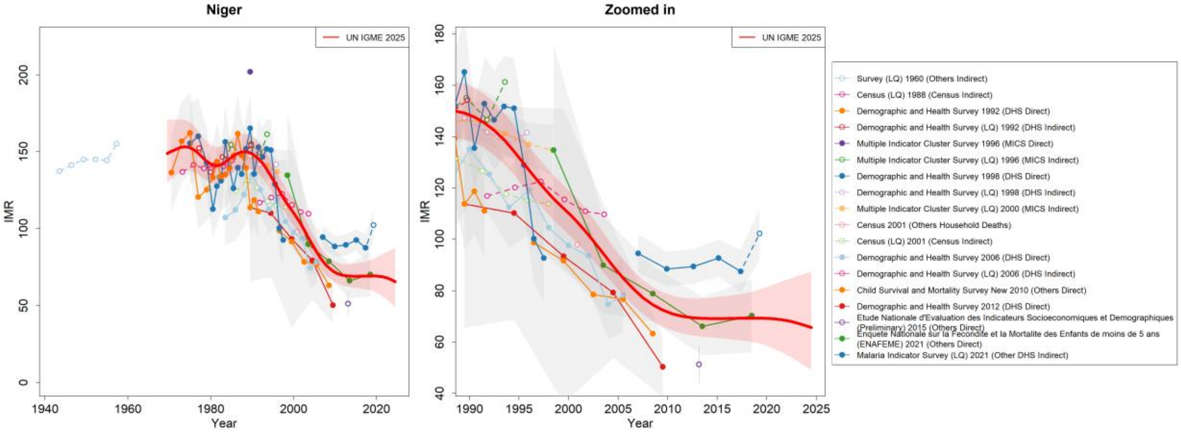


### Neonatal mortality rate

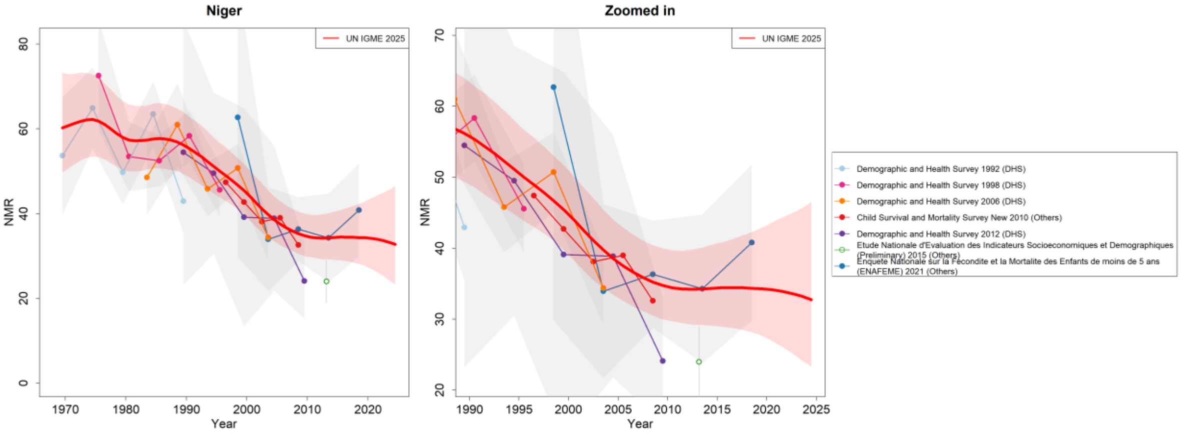

# Nigeria (NGA)

## Under-five mortality rate

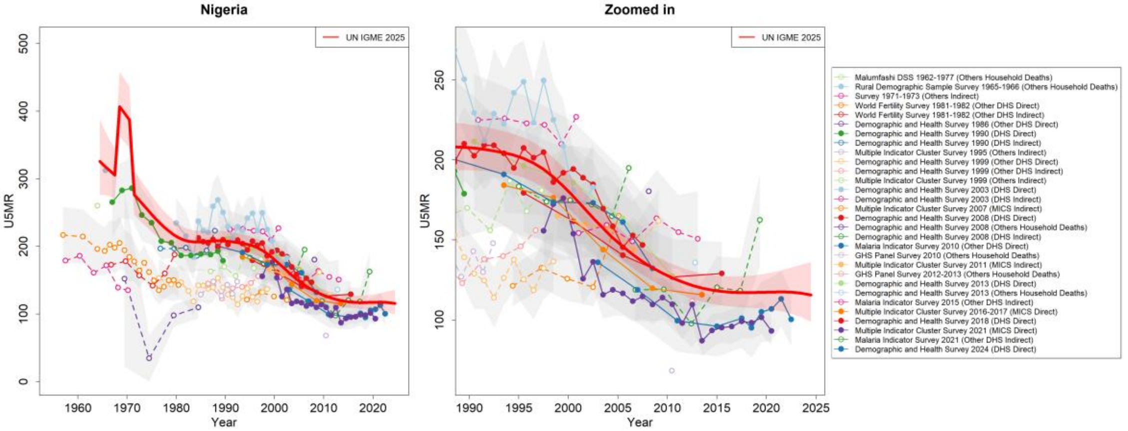


## Infant mortality rate

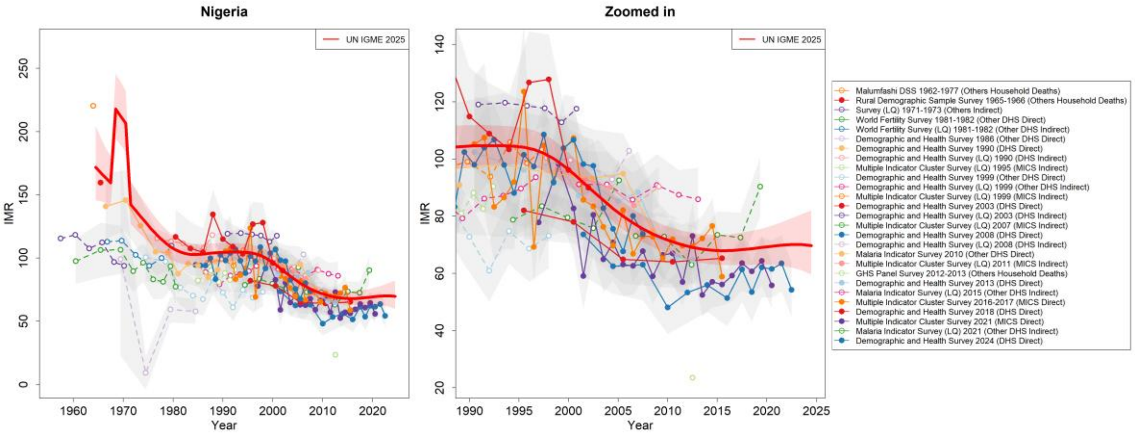


## Neonatal mortality rate

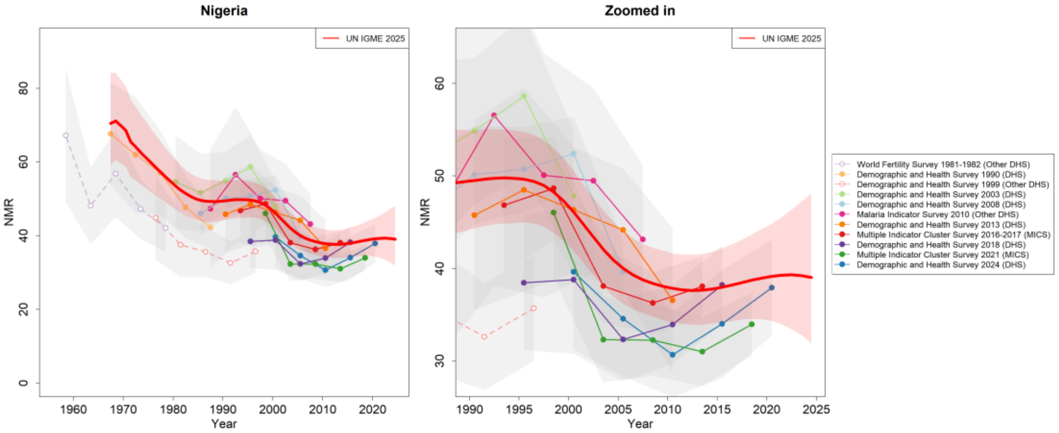

## Niue (NIU)

### Under-five mortality rate

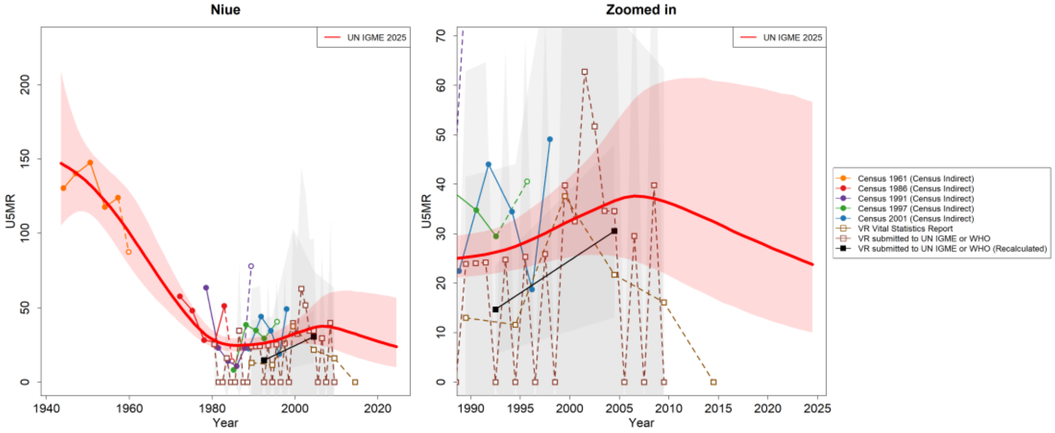


### Infant mortality rate

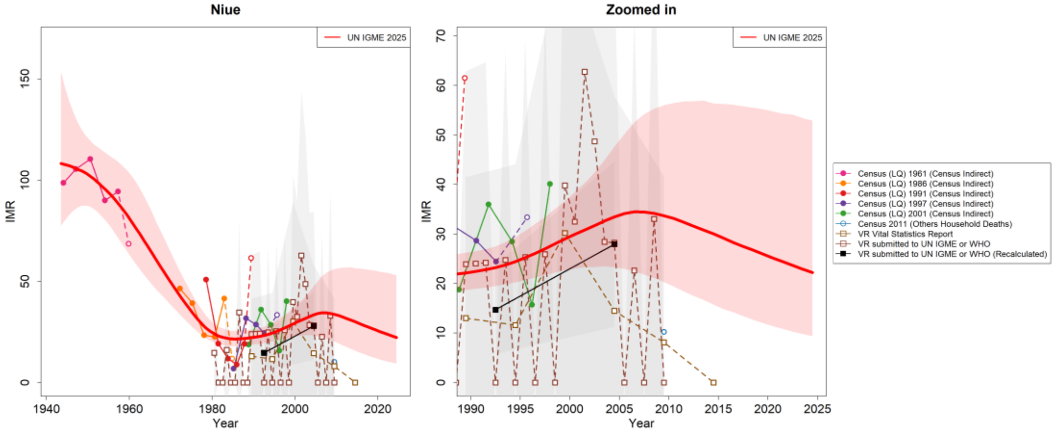


### Neonatal mortality rate

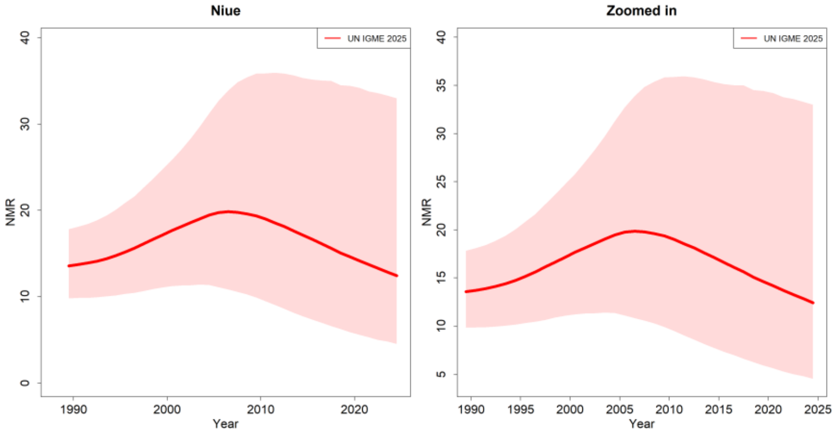

## North Macedonia (MKD)

### Under-five mortality rate

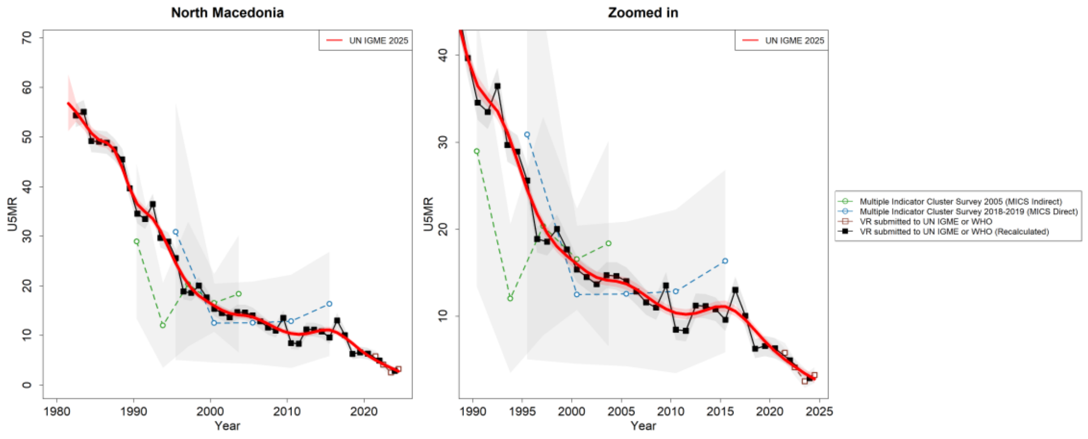


### Infant mortality rate

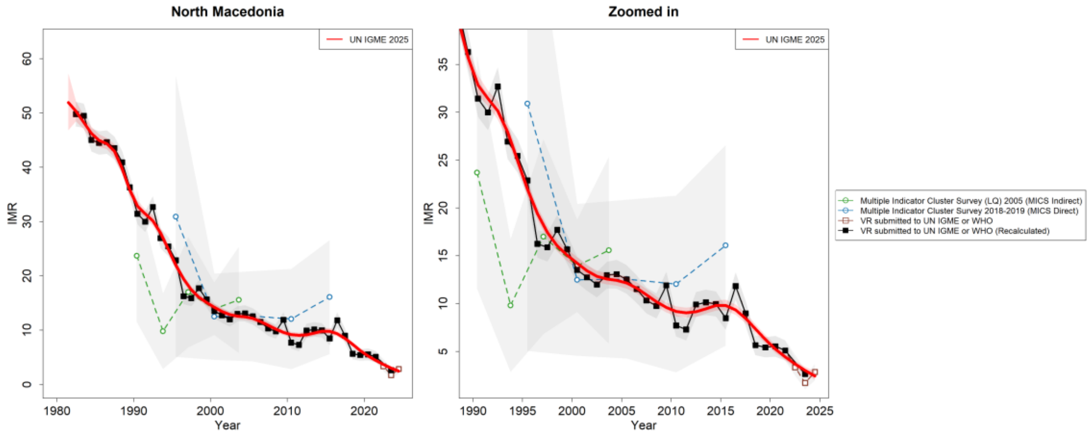


### Neonatal mortality rate

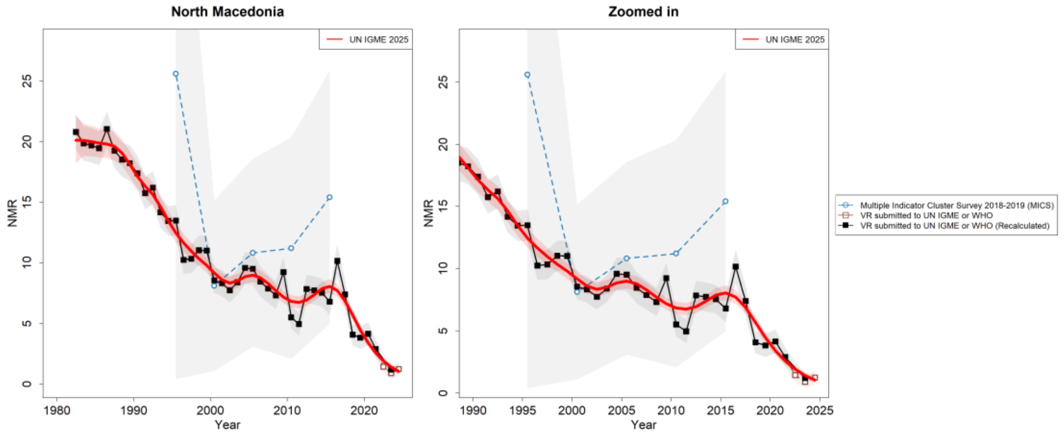

## Norway (NOR)

### Under-five mortality rate

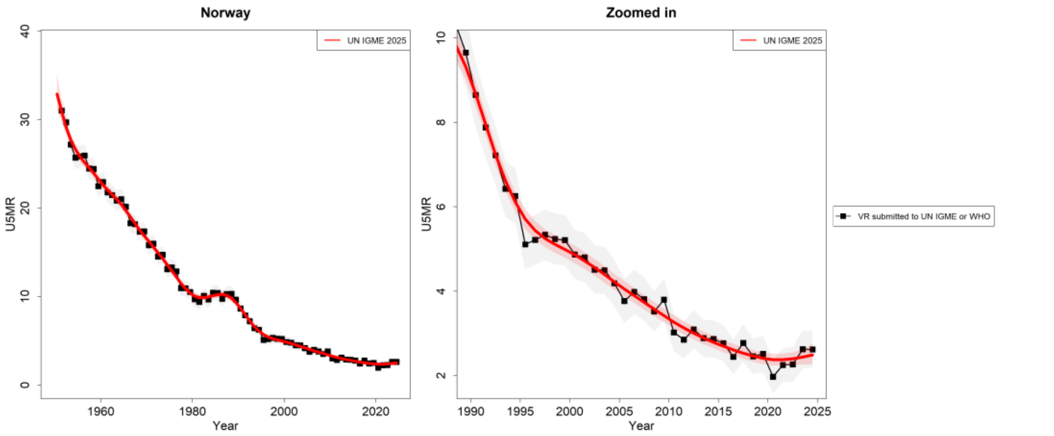


### Infant mortality rate

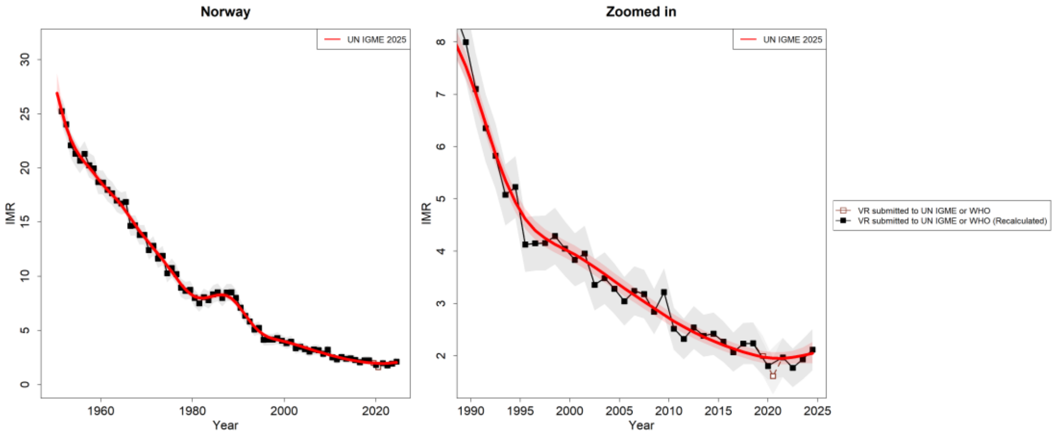


### Neonatal mortality rate

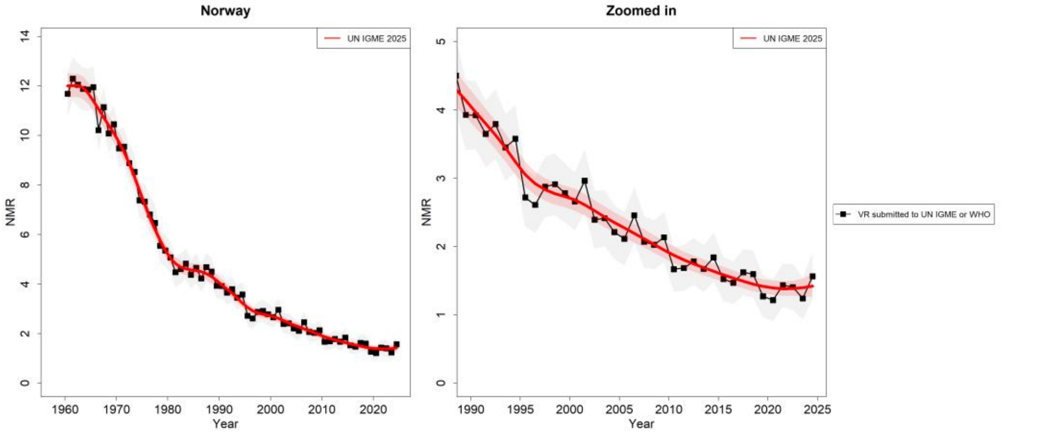

## Oman (OMN)

### Under-five mortality rate

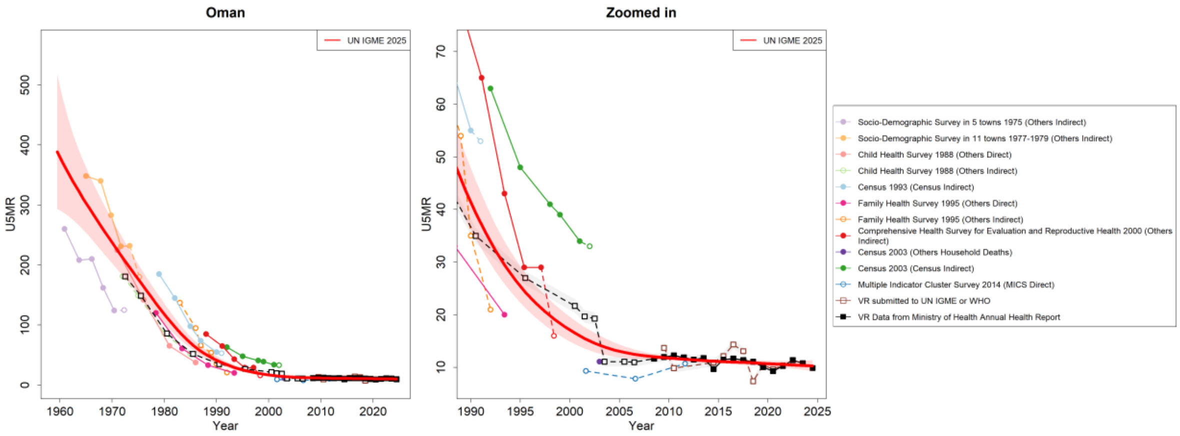


### Infant mortality rate

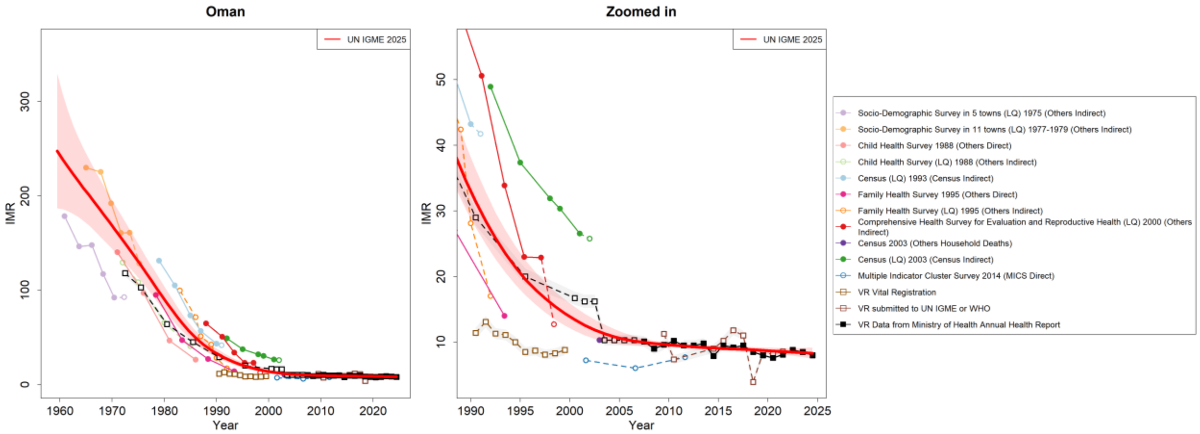


### Neonatal mortality rate

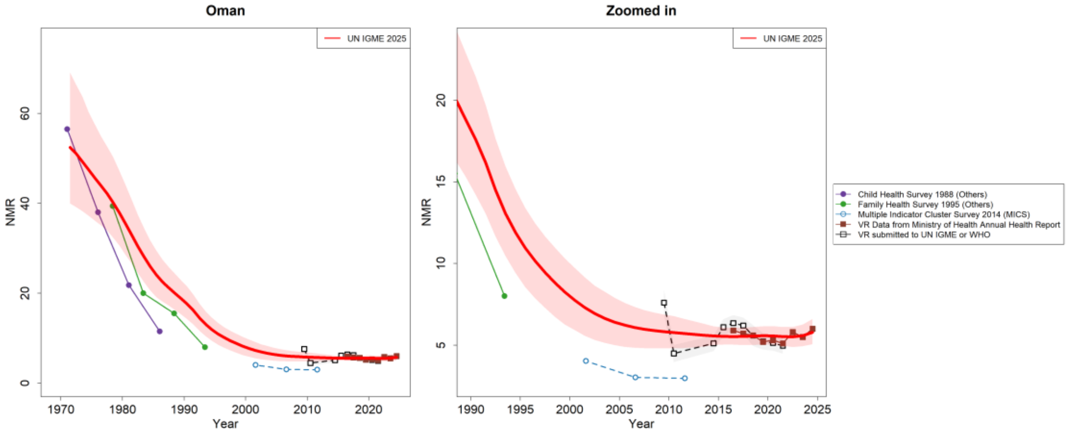

## Pakistan (PAK)

### Under-five mortality rate

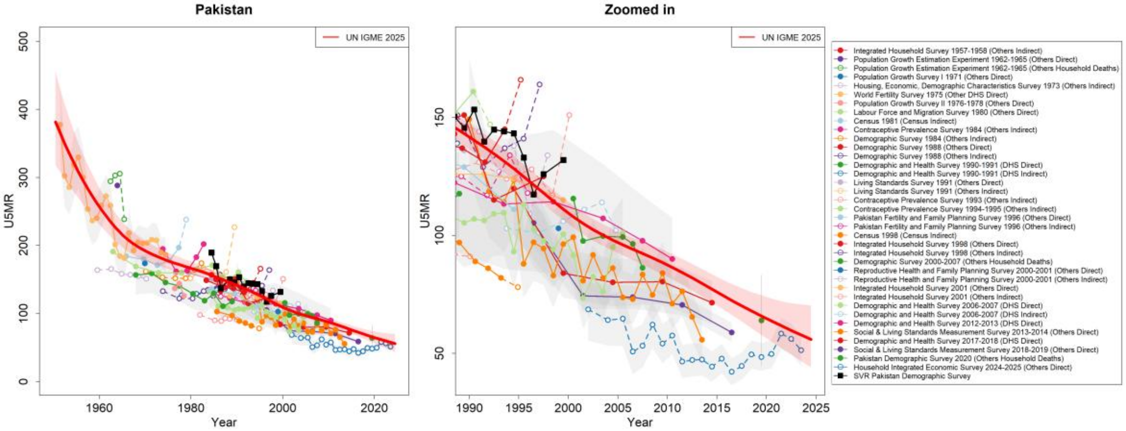


### Infant mortality rate

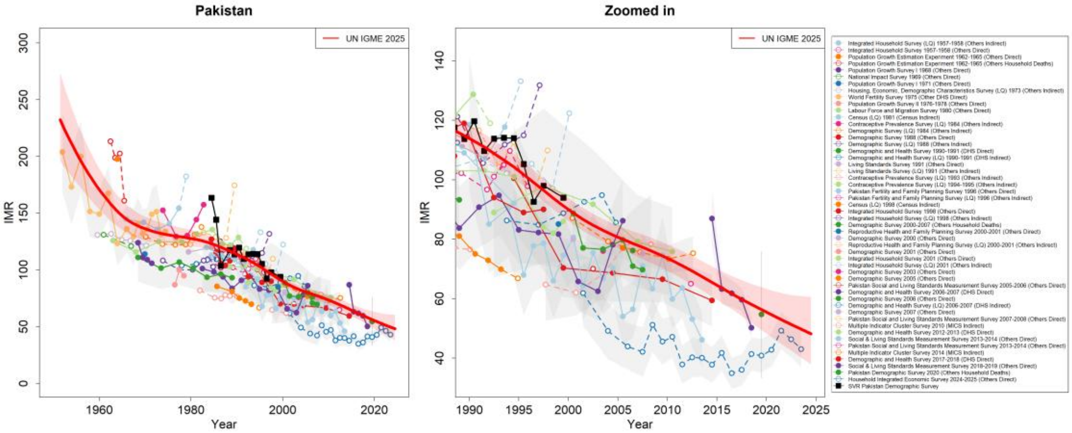


### Neonatal mortality rate

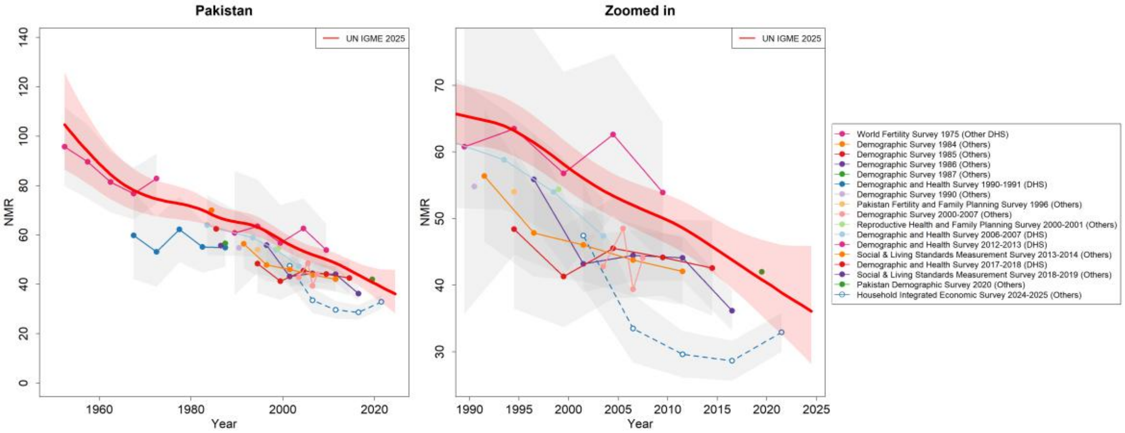

## Palau (PLW)

### Under-five mortality rate

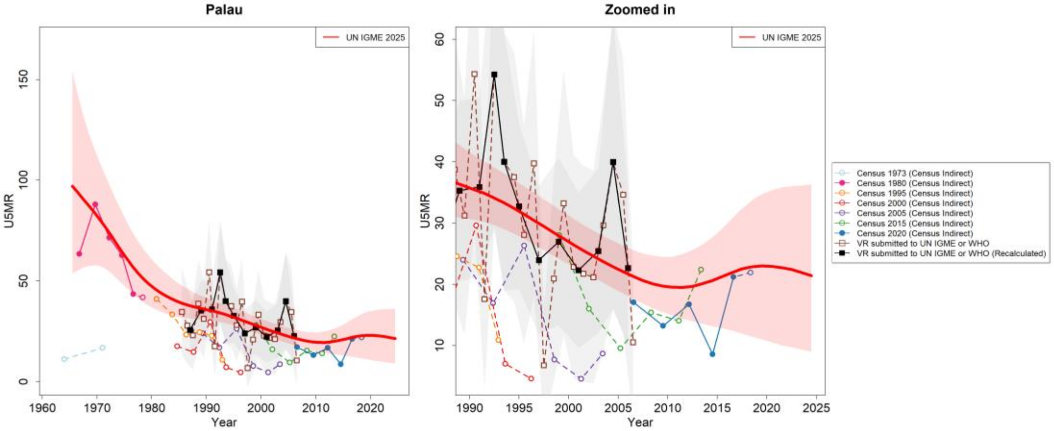


### Infant mortality rate

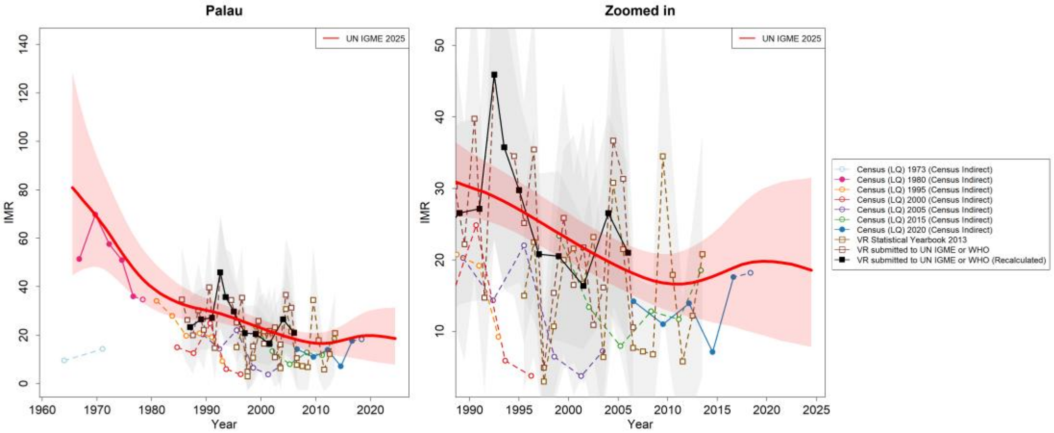


### Neonatal mortality rate

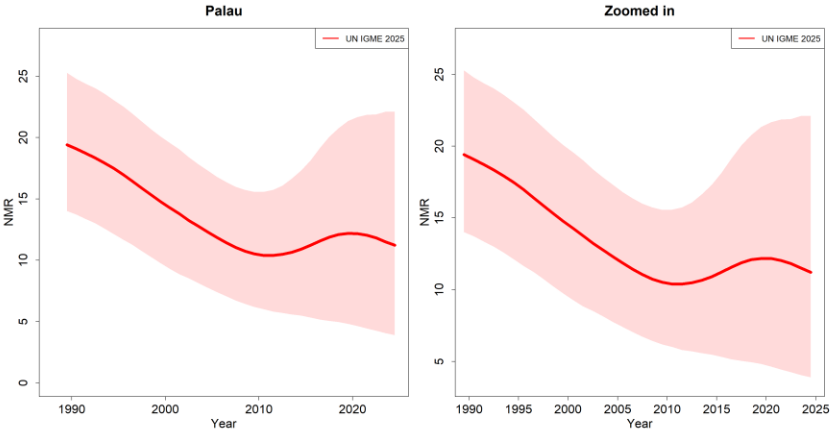

## Panama (PAN)

### Under-five mortality rate

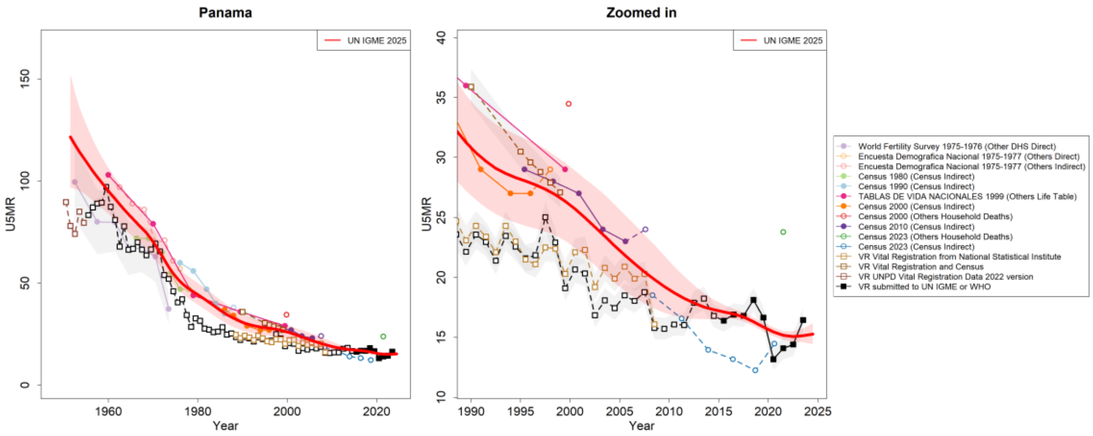


### Infant mortality rate

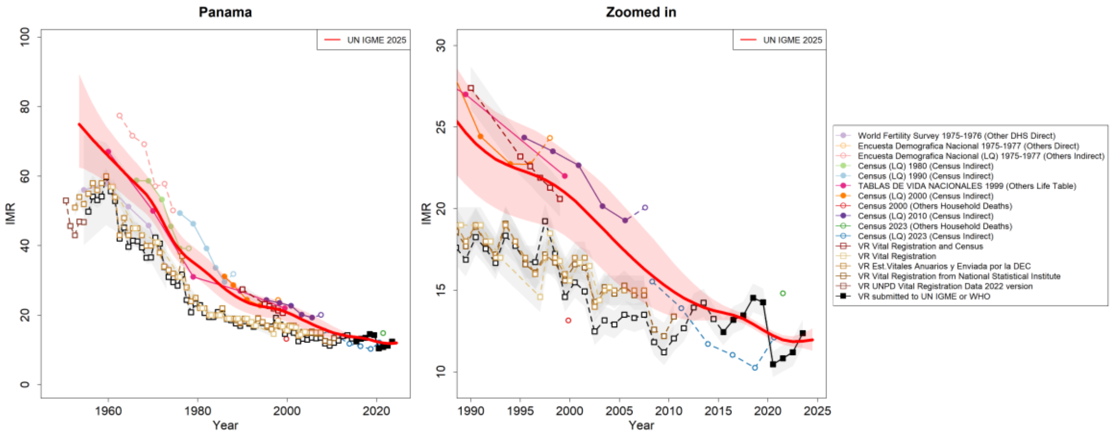


### Neonatal mortality rate

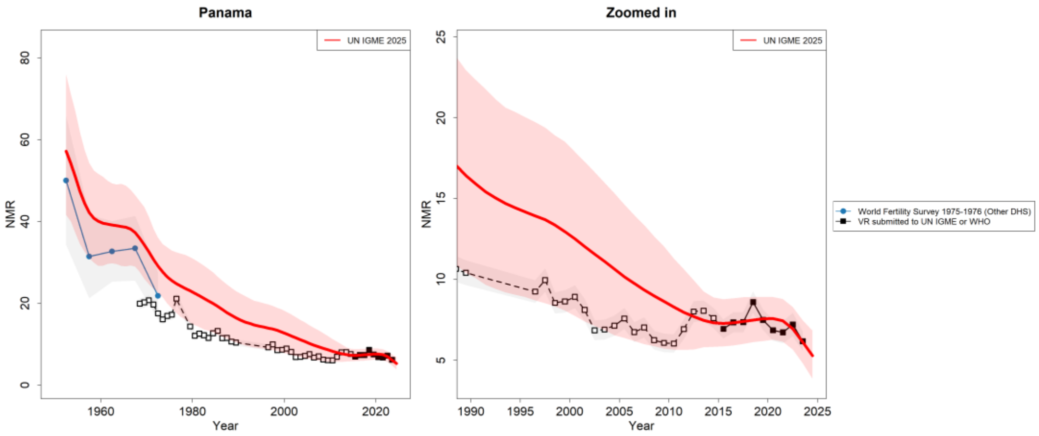

## Papua New Guinea (PNG)

### Under-five mortality rate

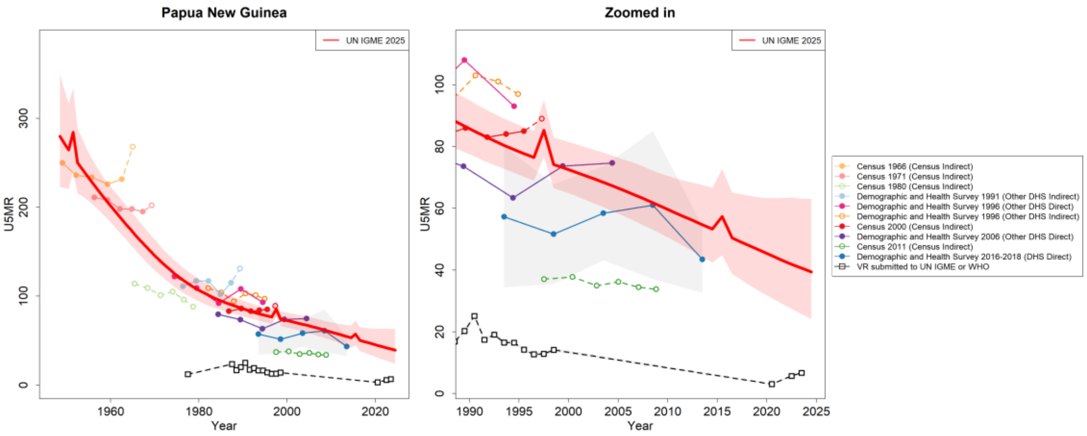


### Infant mortality rate

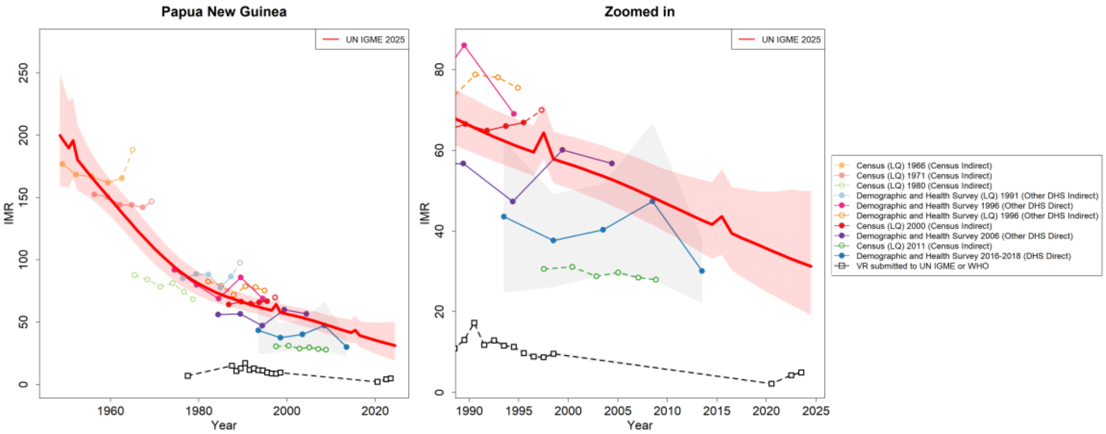


### Neonatal mortality rate

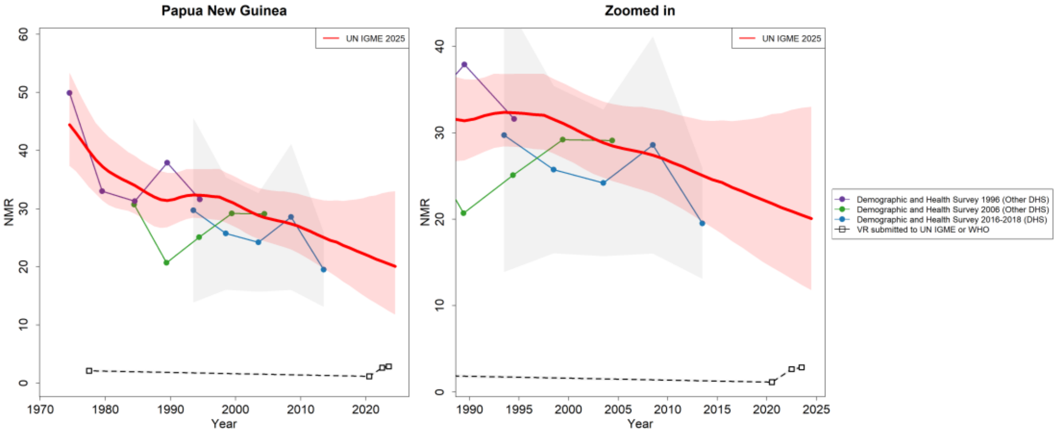

# Paraguay (PRY)

## Under-five mortality rate

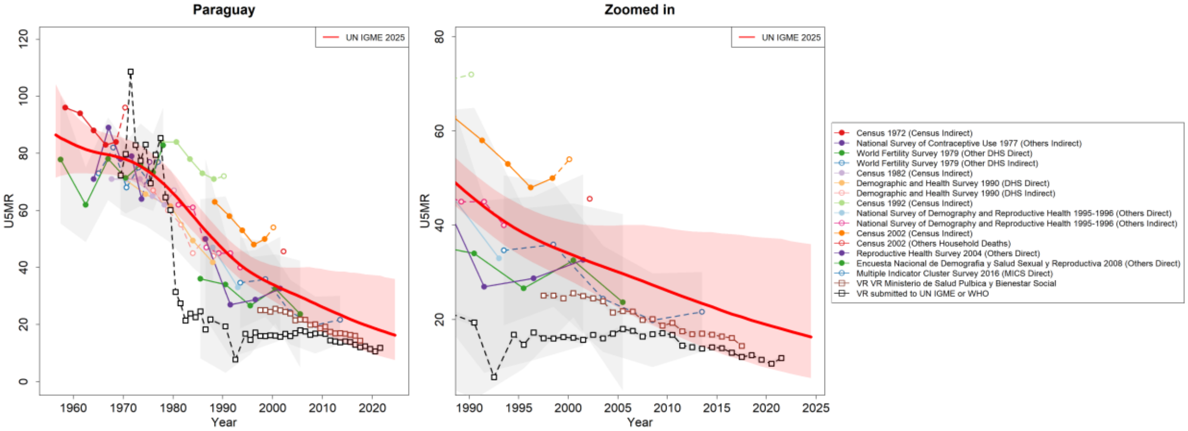


## Infant mortality rate

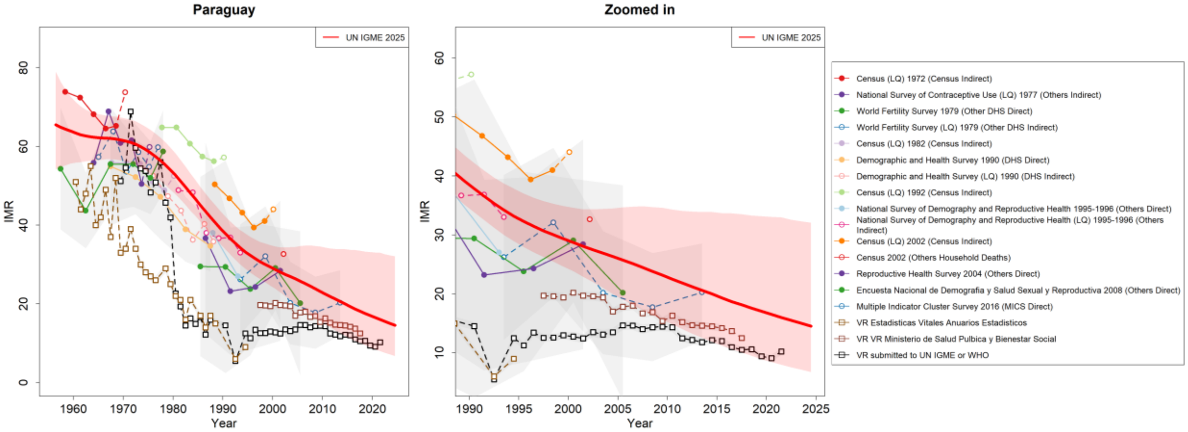


## Neonatal mortality rate

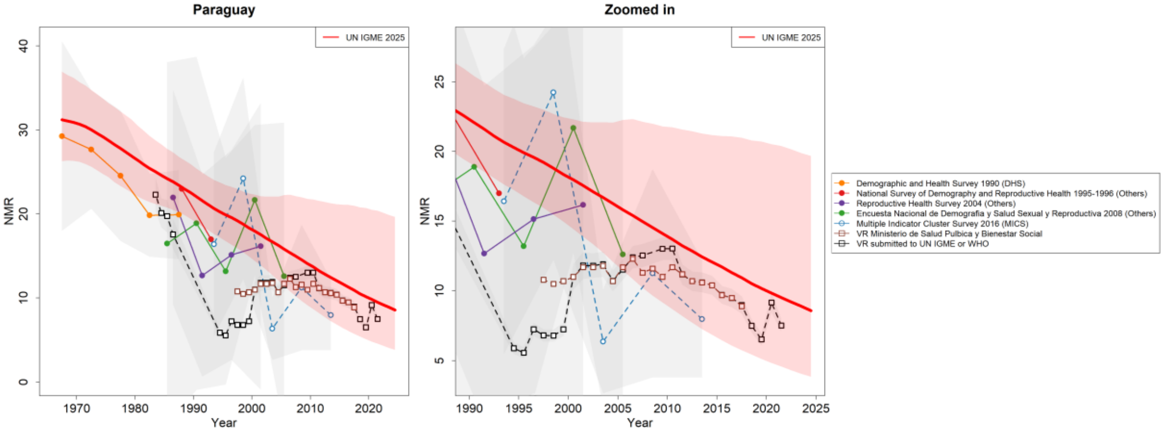

## Peru (PER)

### Under-five mortality rate

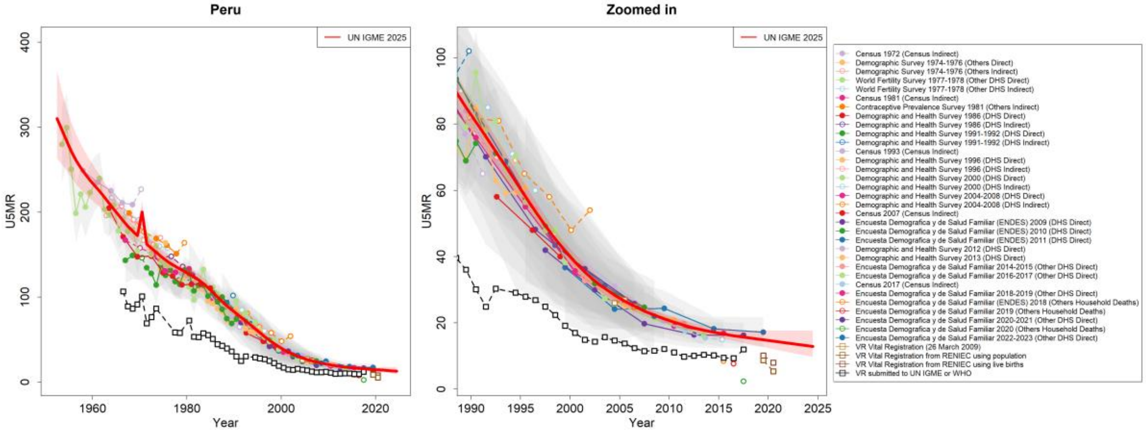


### Infant mortality rate

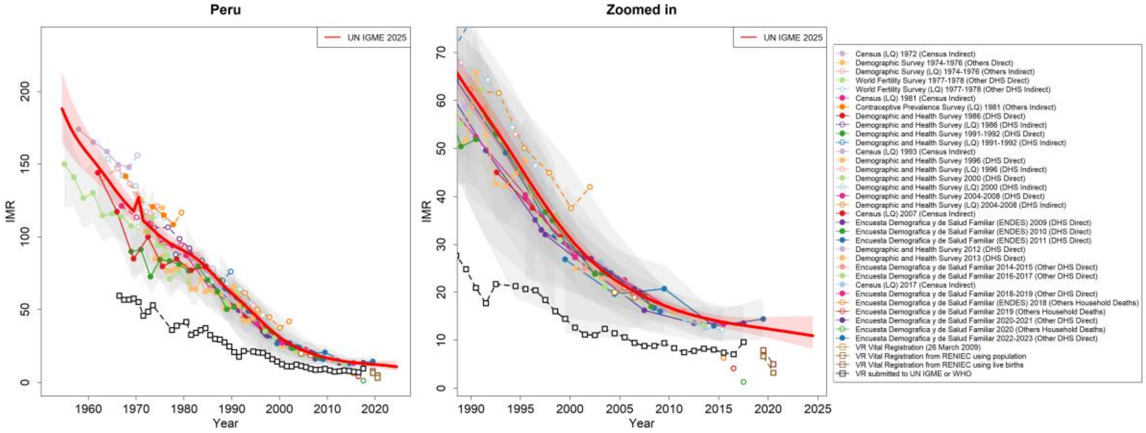


### Neonatal mortality rate

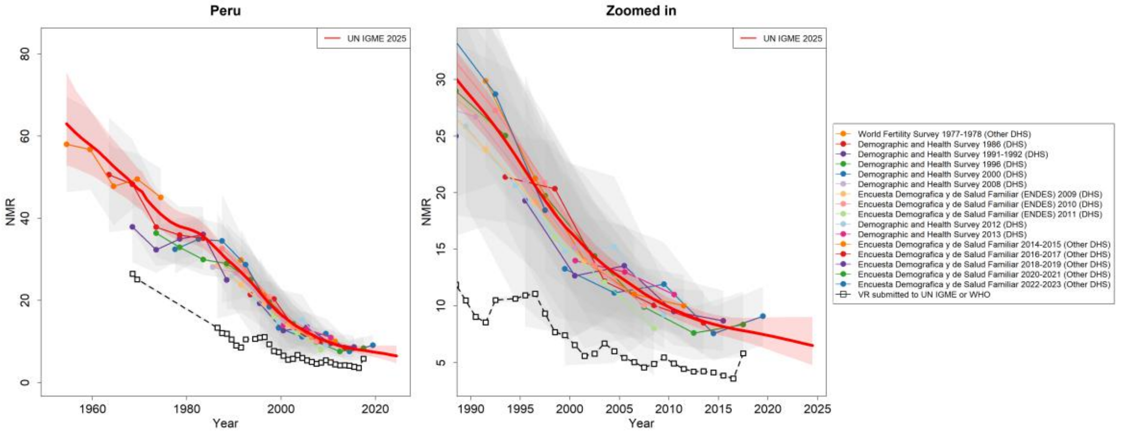

## Philippines (PHL)

### Under-five mortality rate

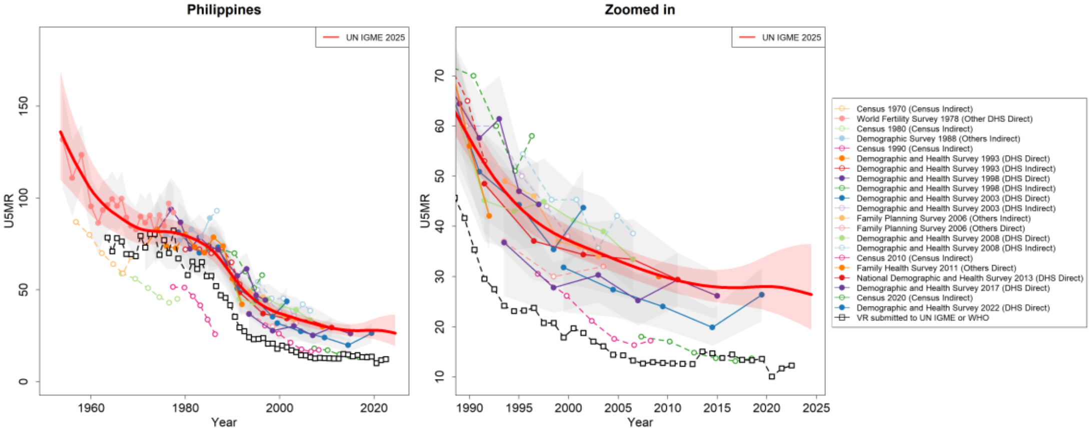


### Infant mortality rate

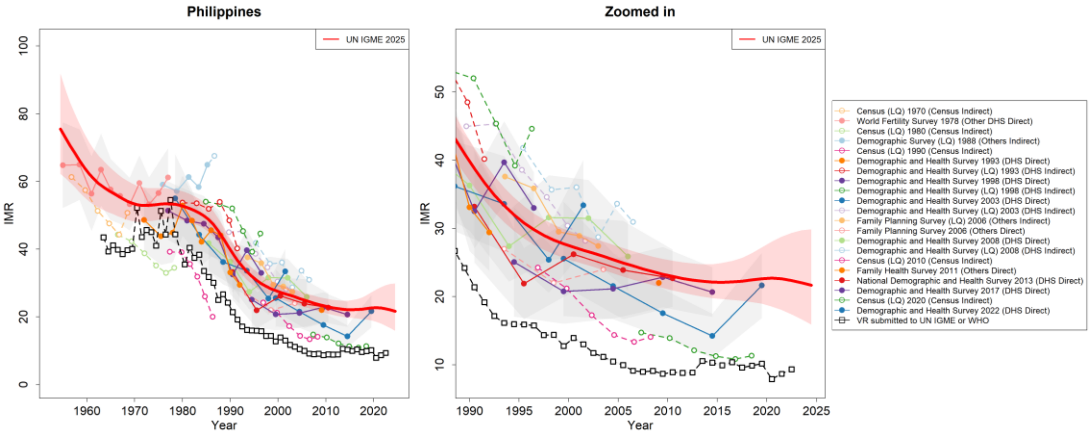


### Neonatal mortality rate

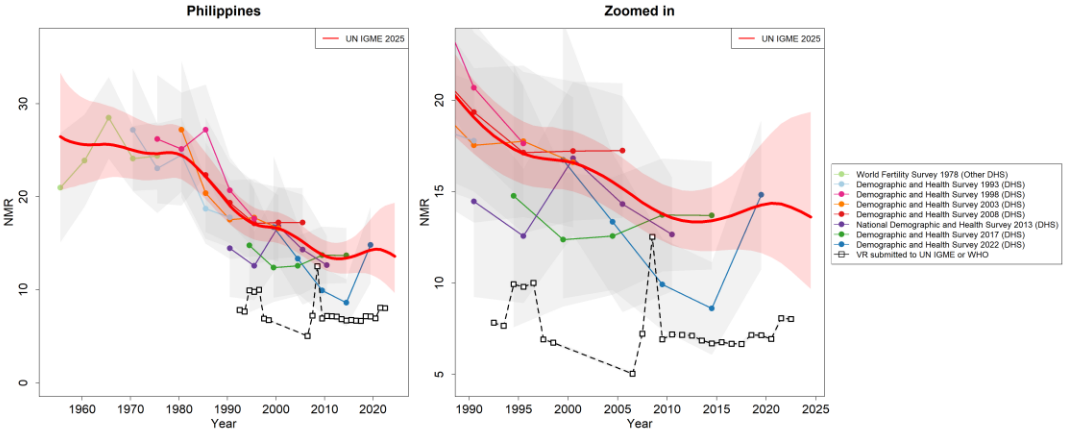

## Poland (POL)

### Under-five mortality rate

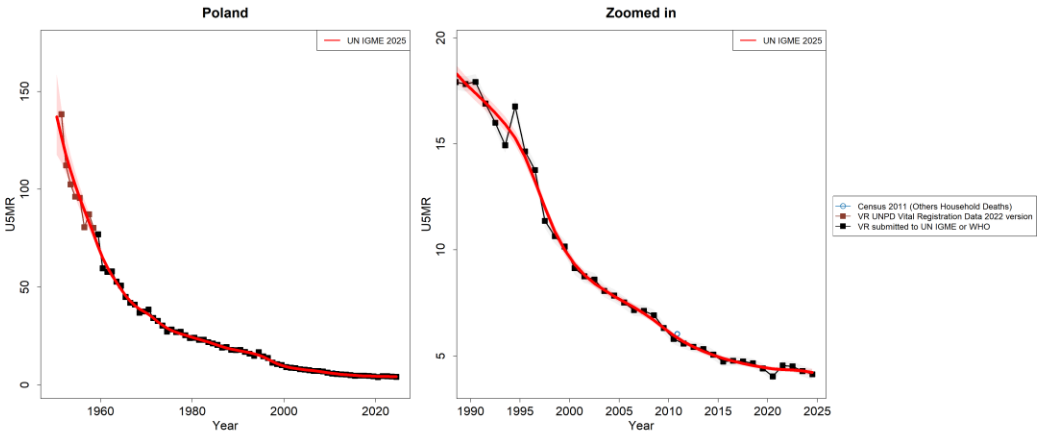


### Infant mortality rate

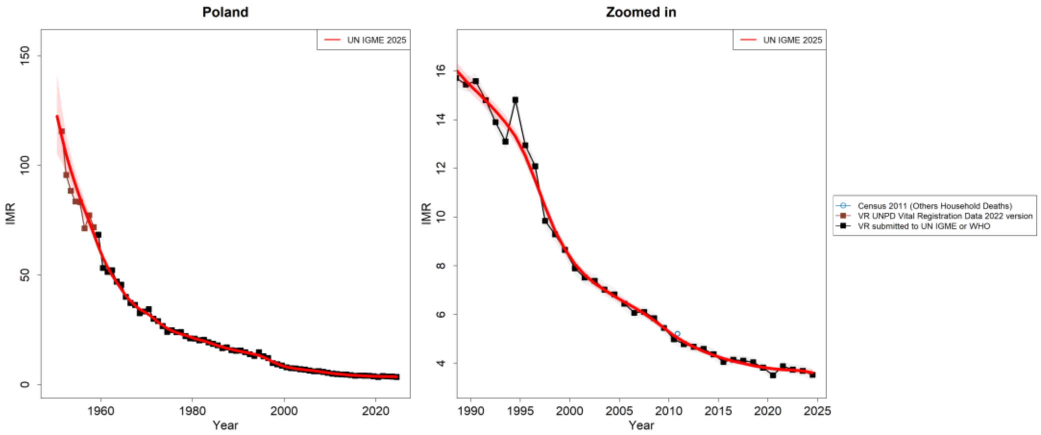


### Neonatal mortality rate

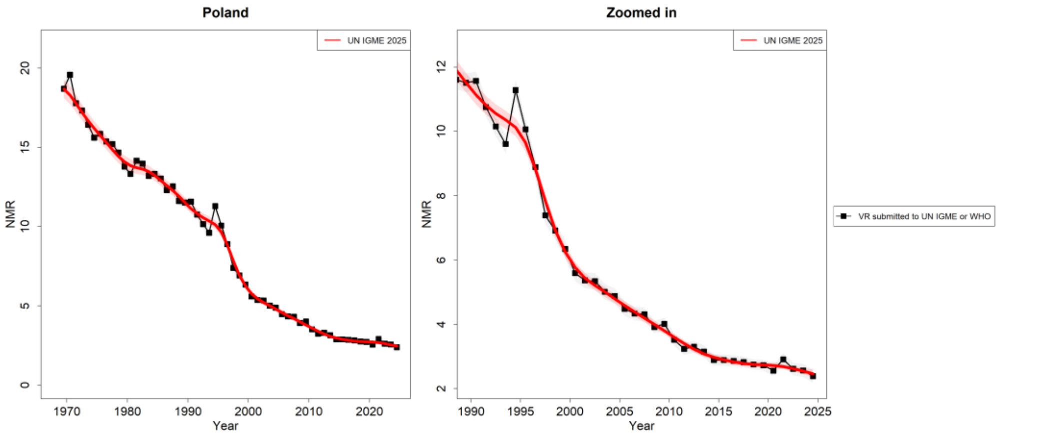

## Portugal (PRT)

### Under-five mortality rate

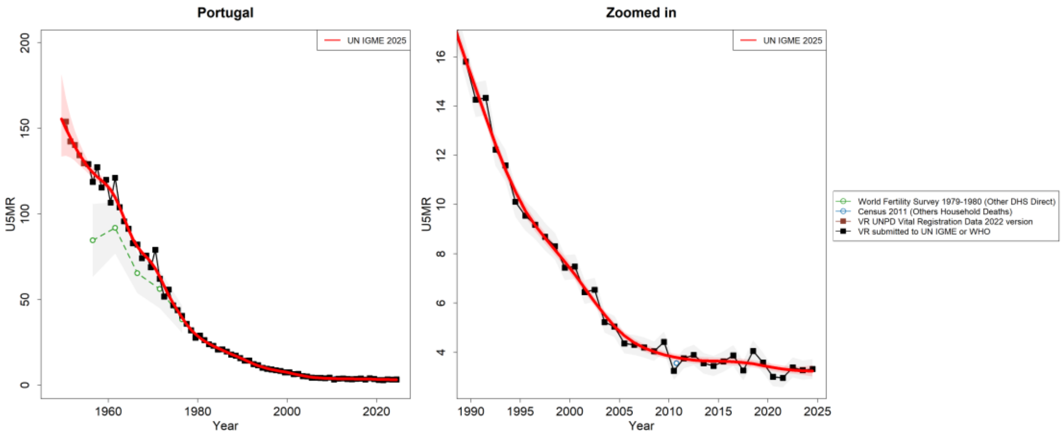


### Infant mortality rate

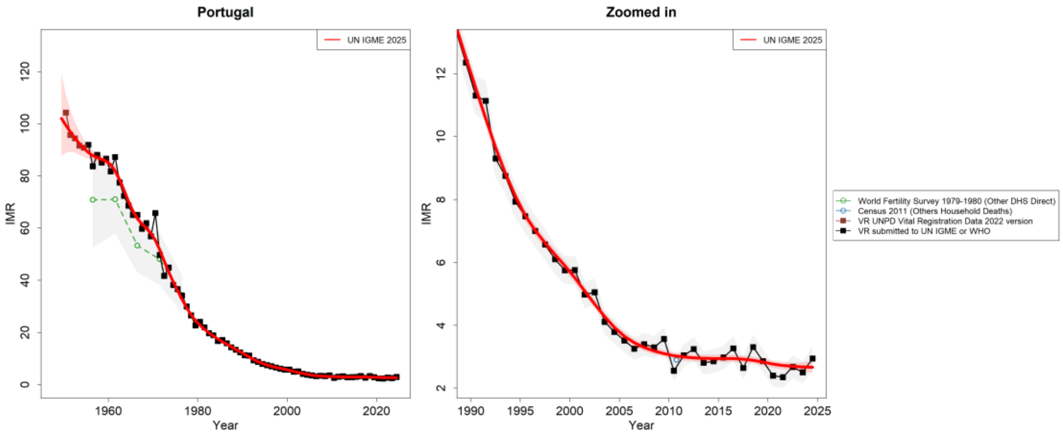


### Neonatal mortality rate

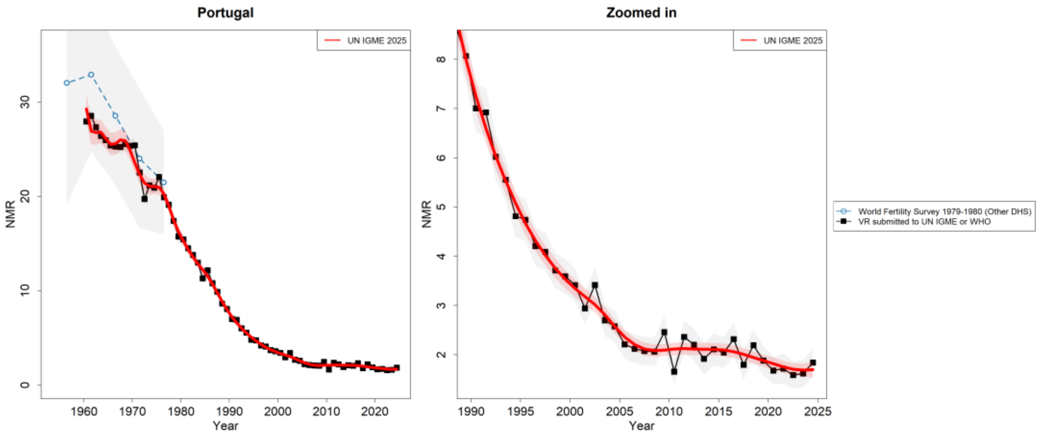

## Qatar (QAT)

### Under-five mortality rate

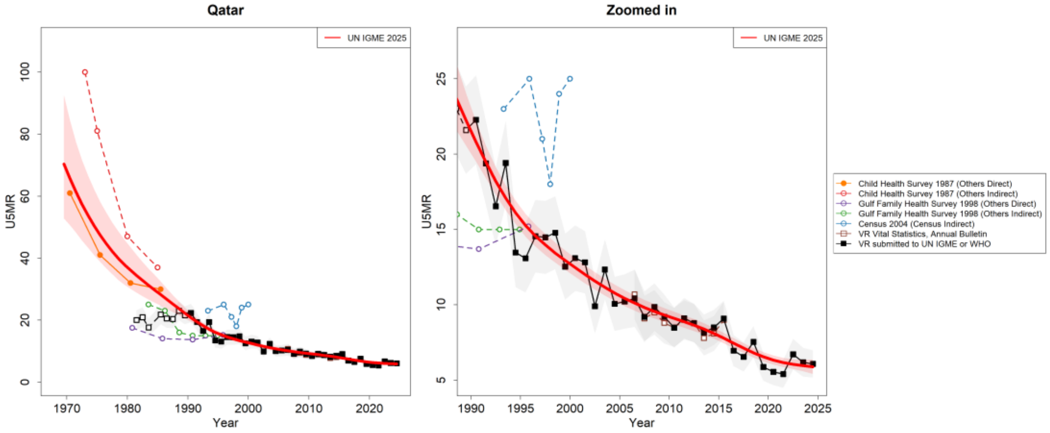


### Infant mortality rate

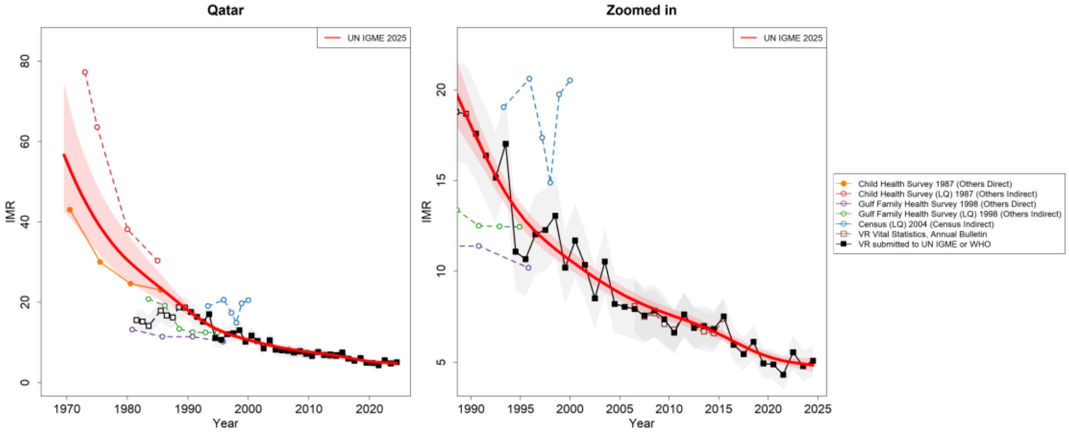


### Neonatal mortality rate

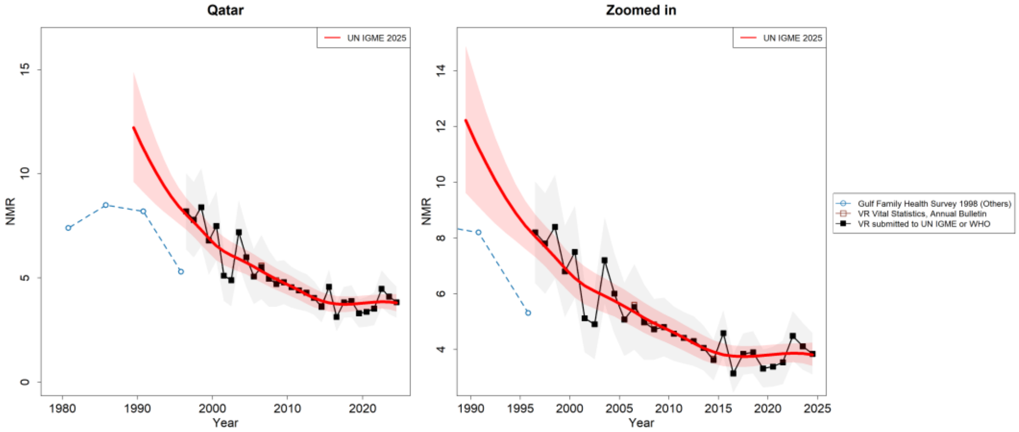

## Republic of Korea (KOR)

### Under-five mortality rate

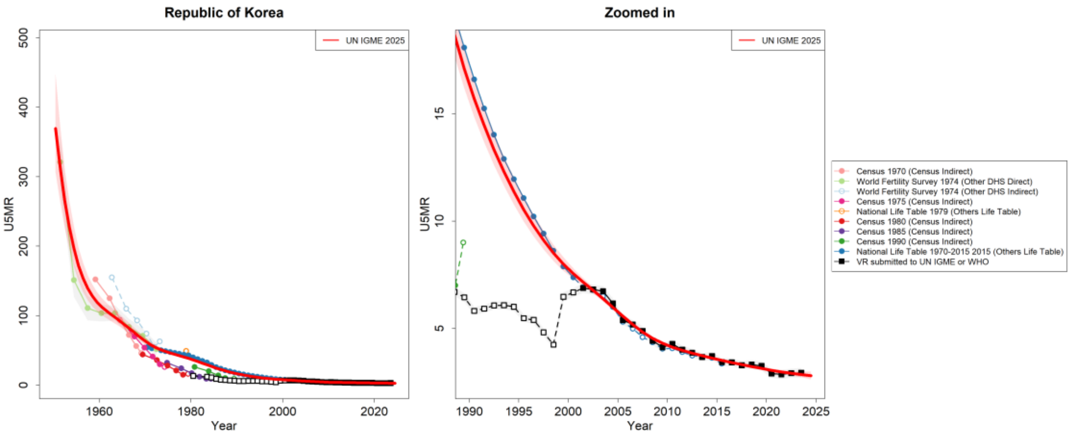


### Infant mortality rate

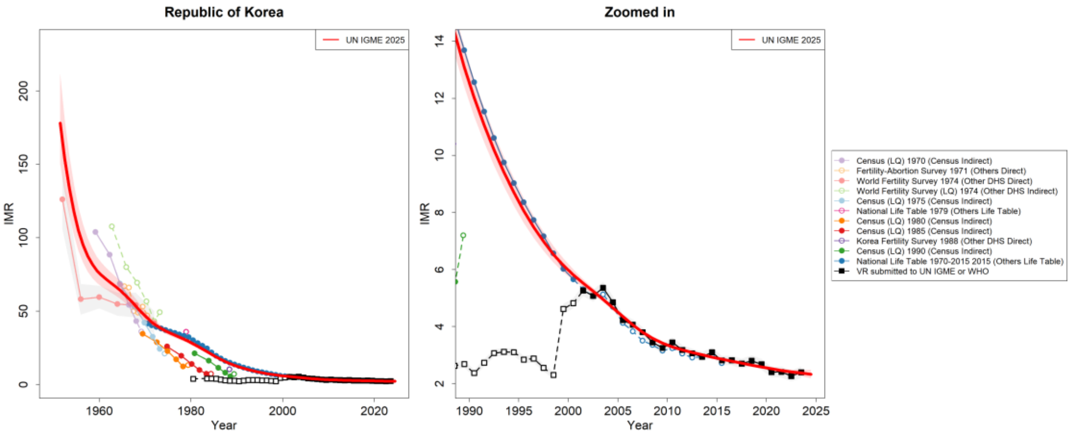


### Neonatal mortality rate

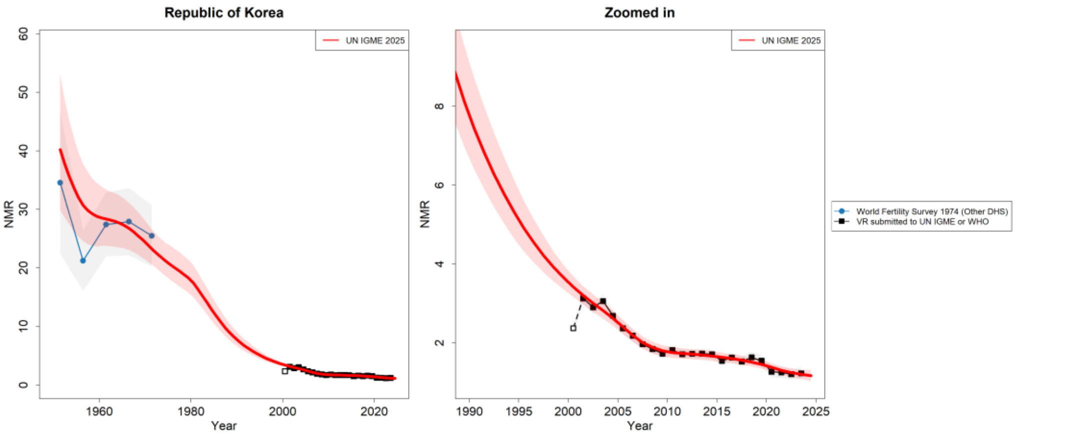

## Republic of Moldova (MDA)

### Under-five mortality rate

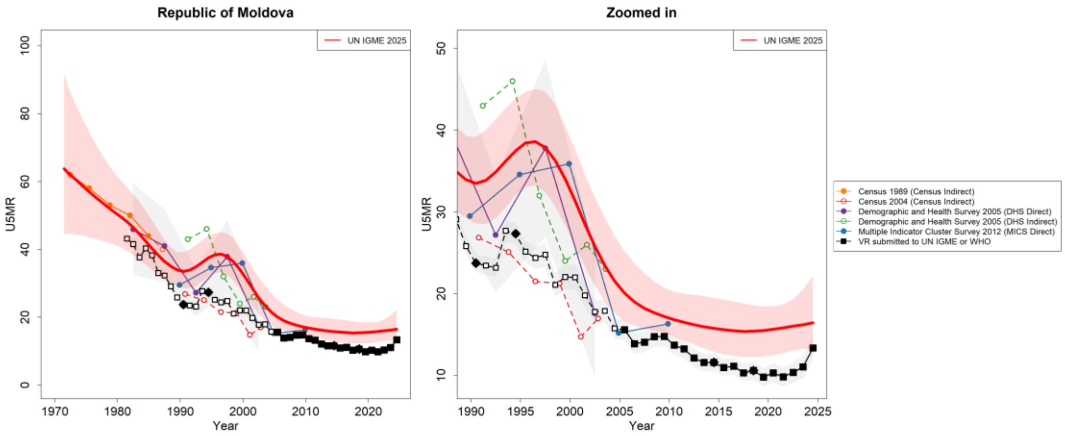


### Infant mortality rate

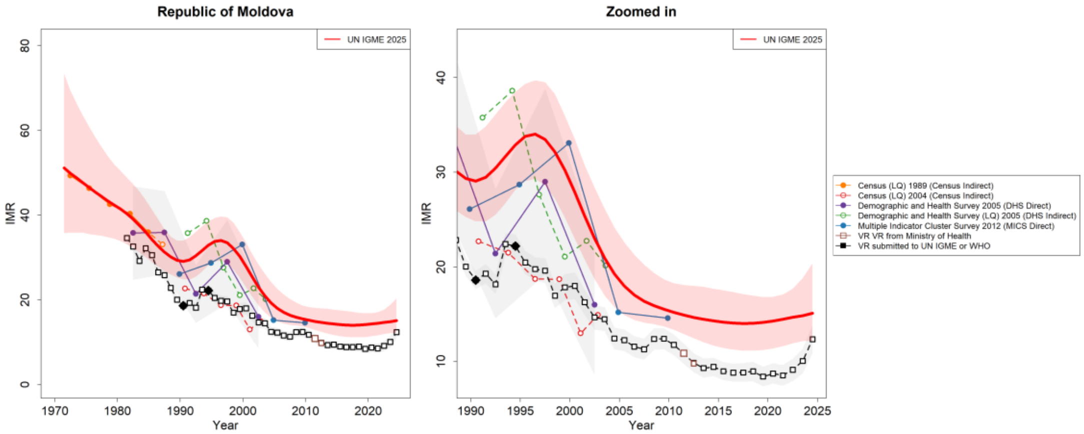


### Neonatal mortality rate

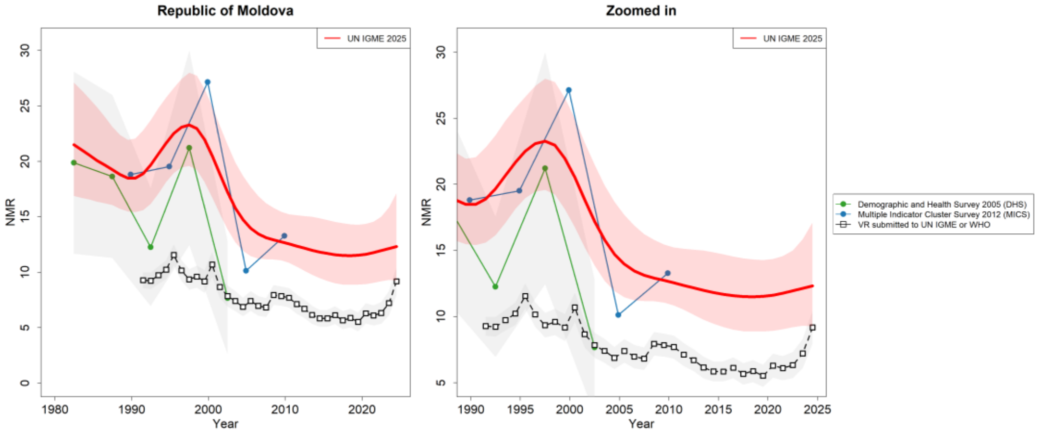

## Romania (ROU)

### Under-five mortality rate

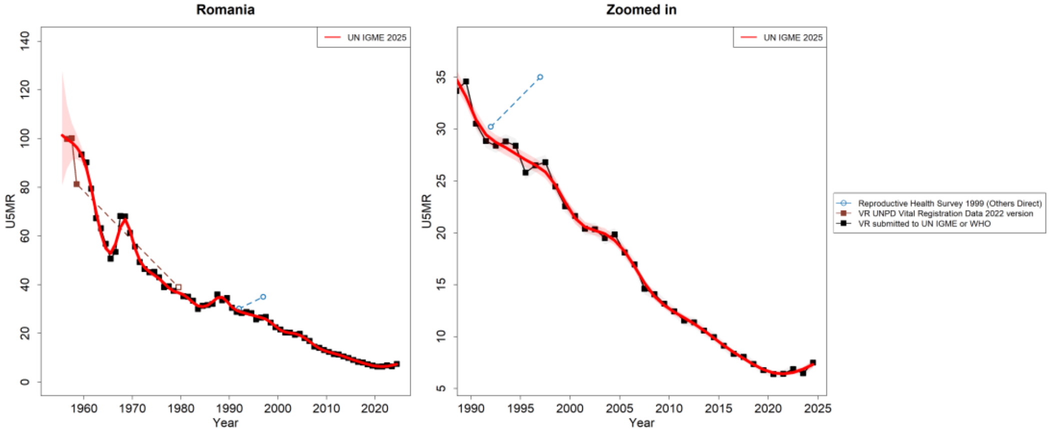


### Infant mortality rate

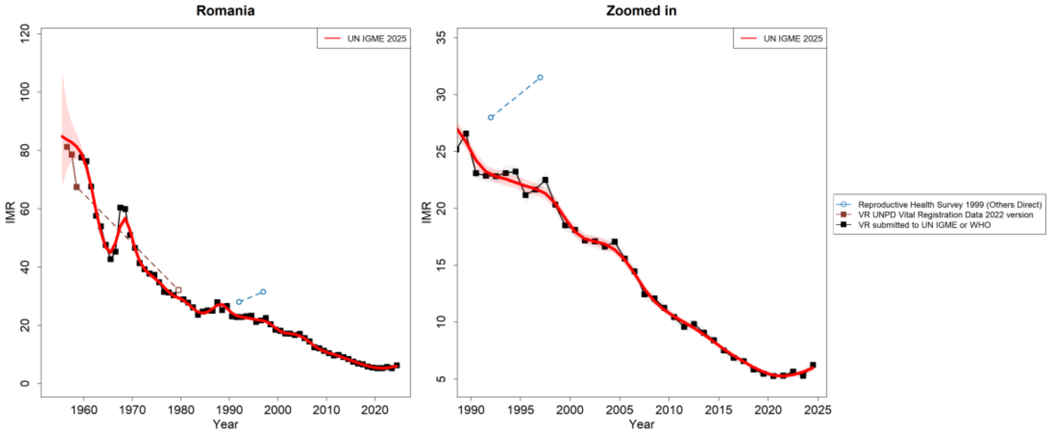


### Neonatal mortality rate

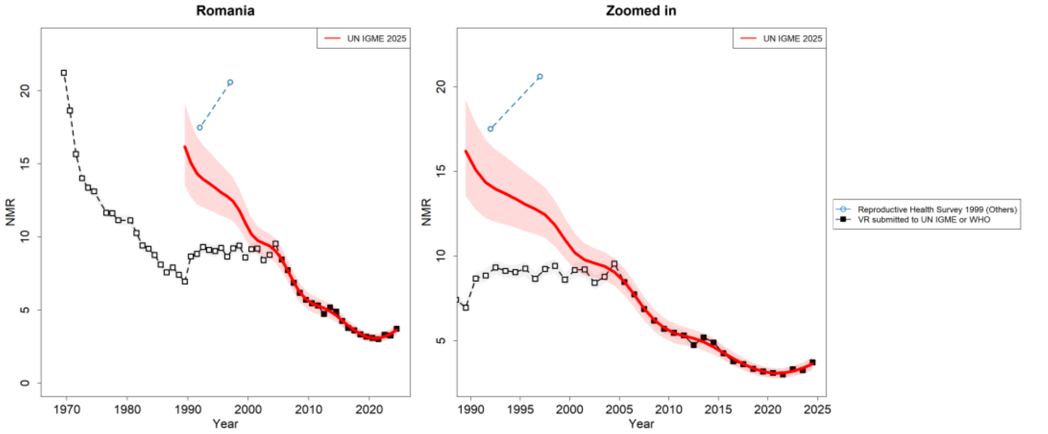

## Russian Federation (RUS)

### Under-five mortality rate

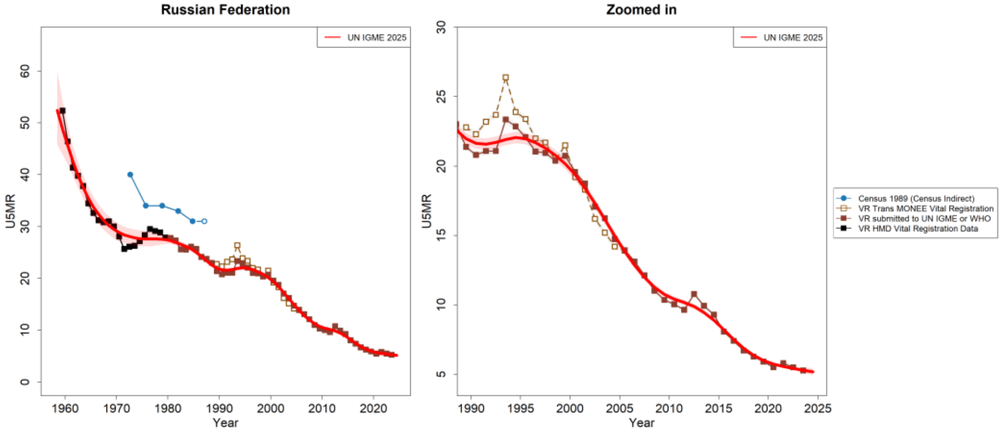


### Infant mortality rate

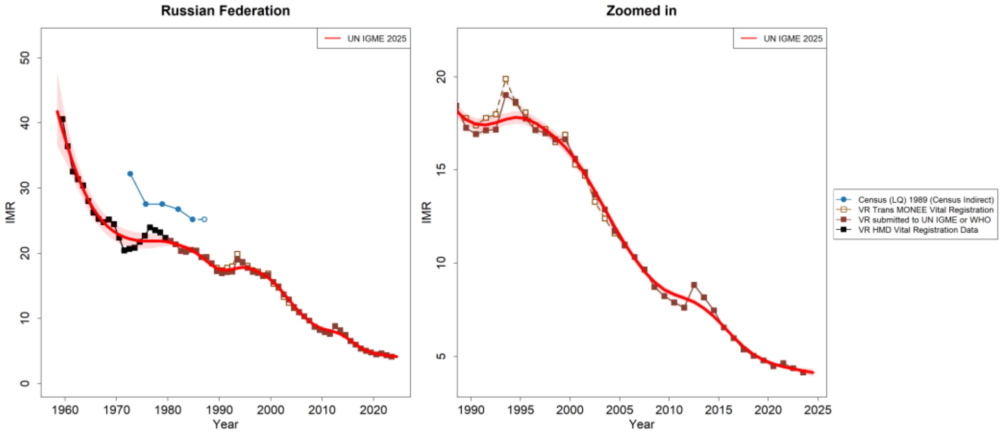


### Neonatal mortality rate

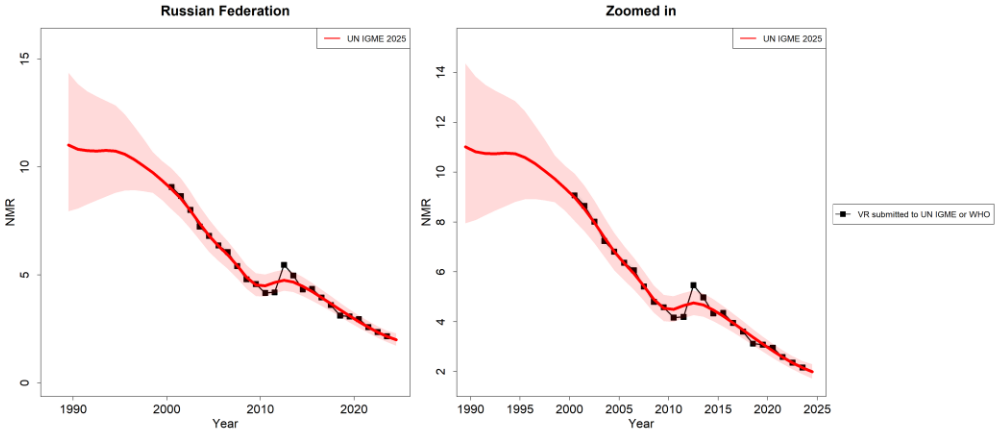

## Rwanda (RWA)

### Under-five mortality rate

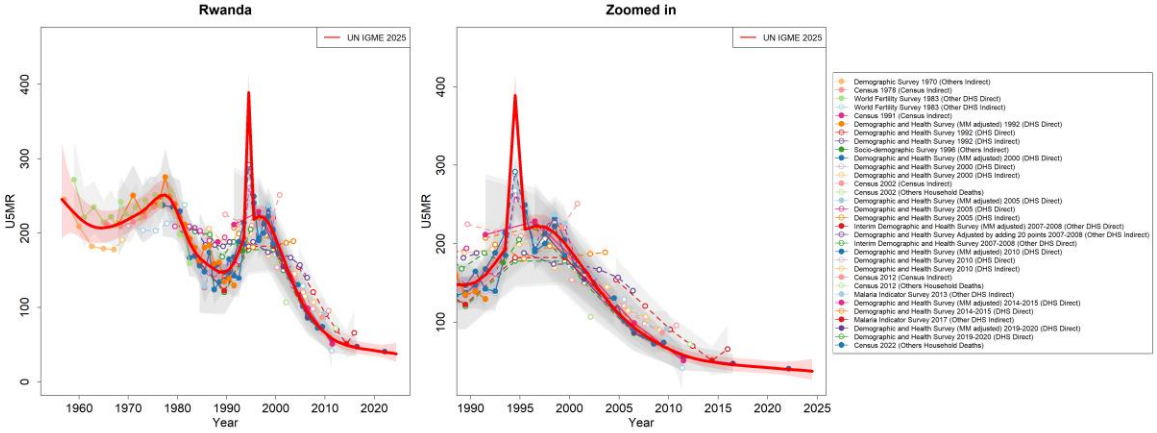


### Infant mortality rate

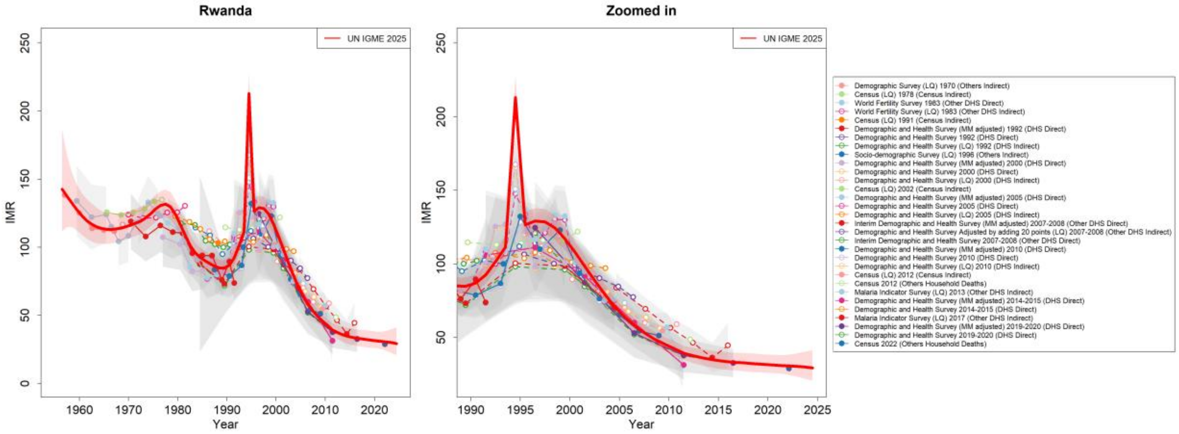


### Neonatal mortality rate

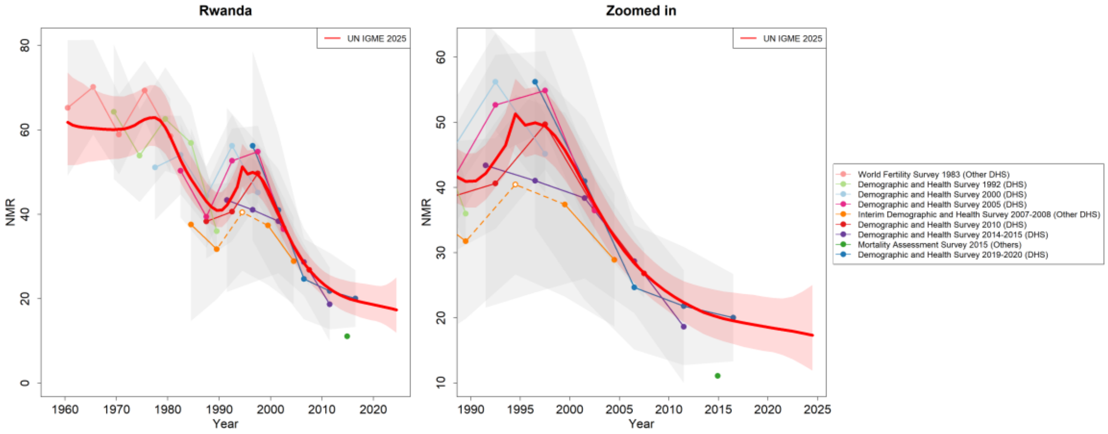

## Saint Kitts and Nevis (KNA)

### Under-five mortality rate

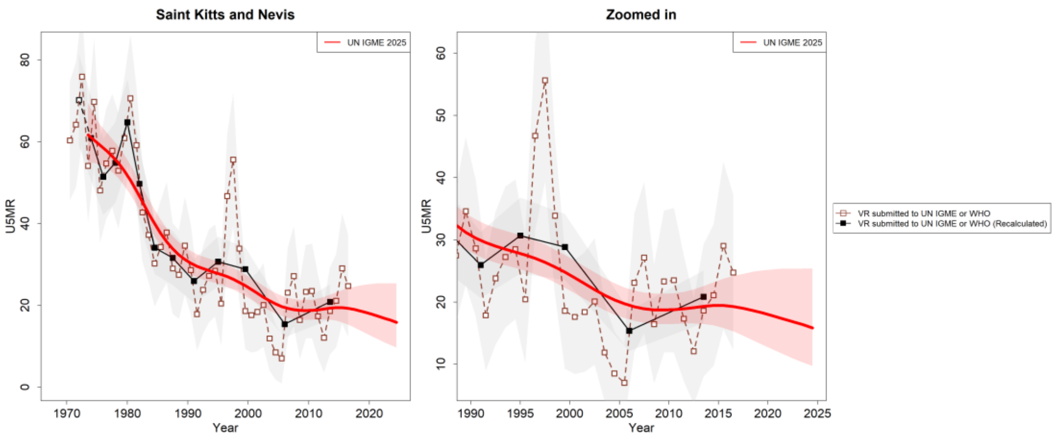


### Infant mortality rate

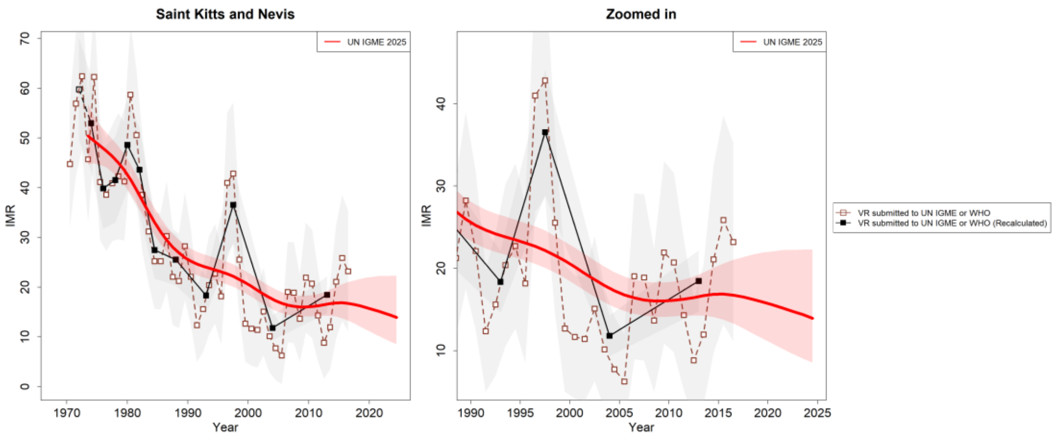


### Neonatal mortality rate

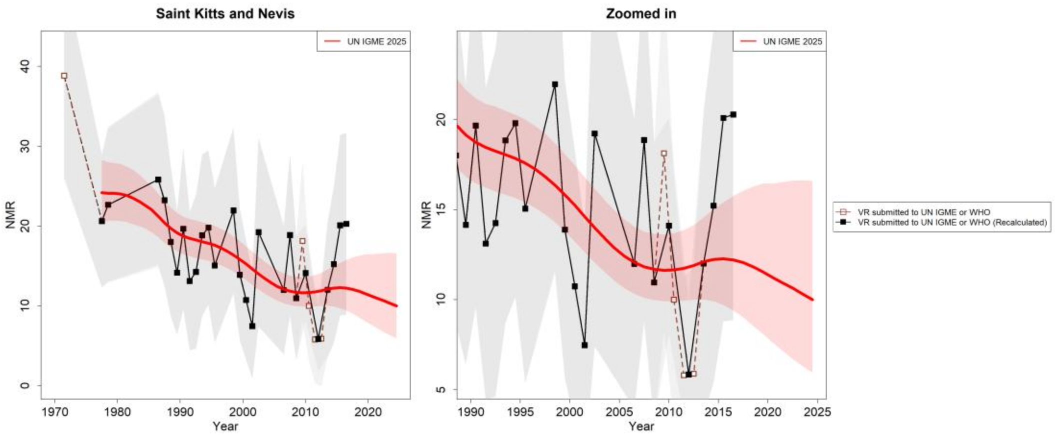

# Saint Lucia (LCA)

## Under-five mortality rate

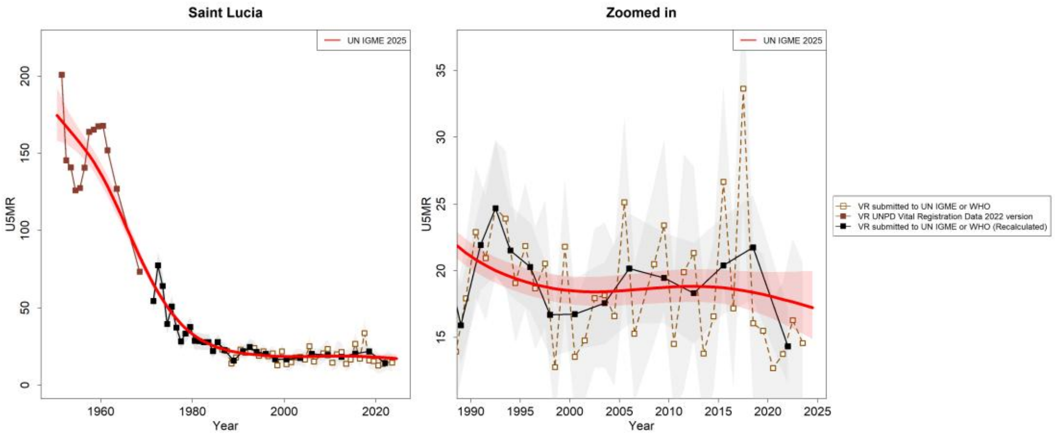


## Infant mortality rate

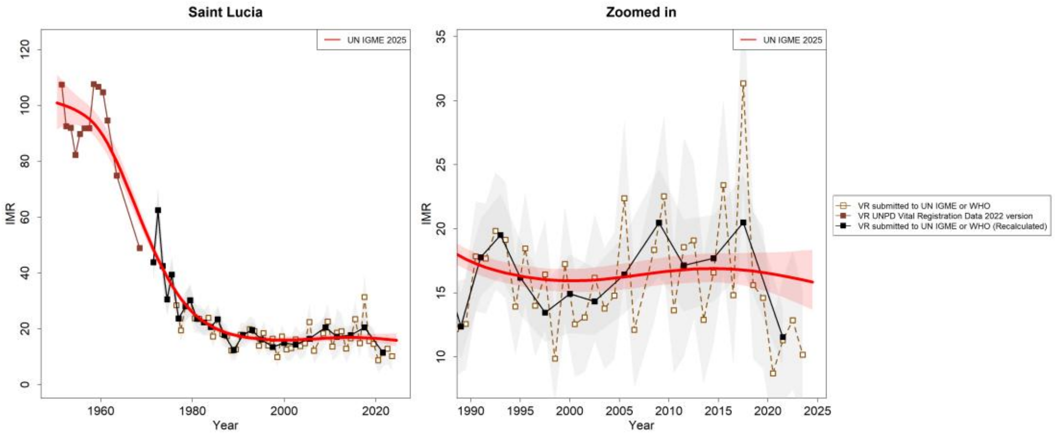


## Neonatal mortality rate

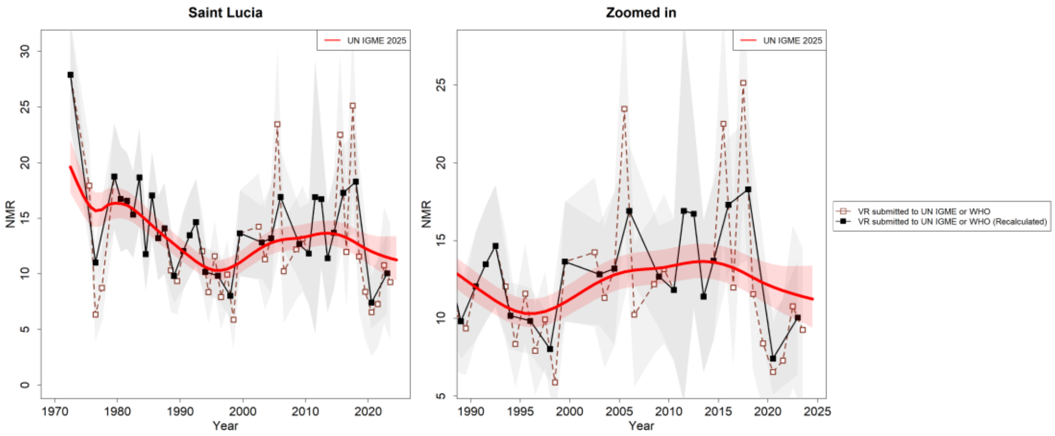

## Saint Vincent and the Grenadines (VCT)

### Under-five mortality rate

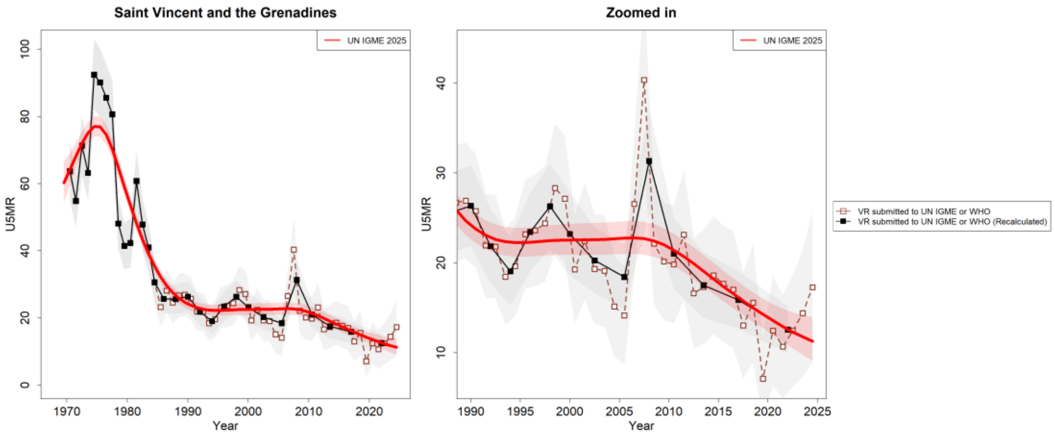


### Infant mortality rate

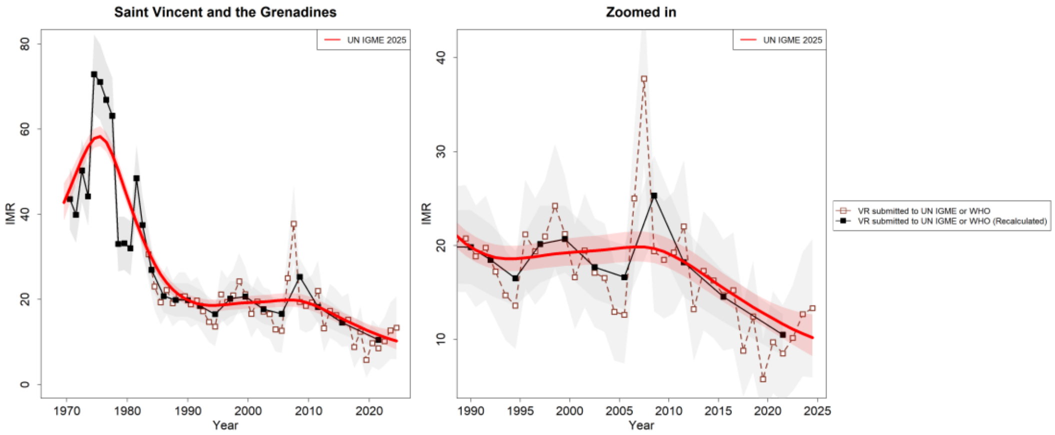


### Neonatal mortality rate

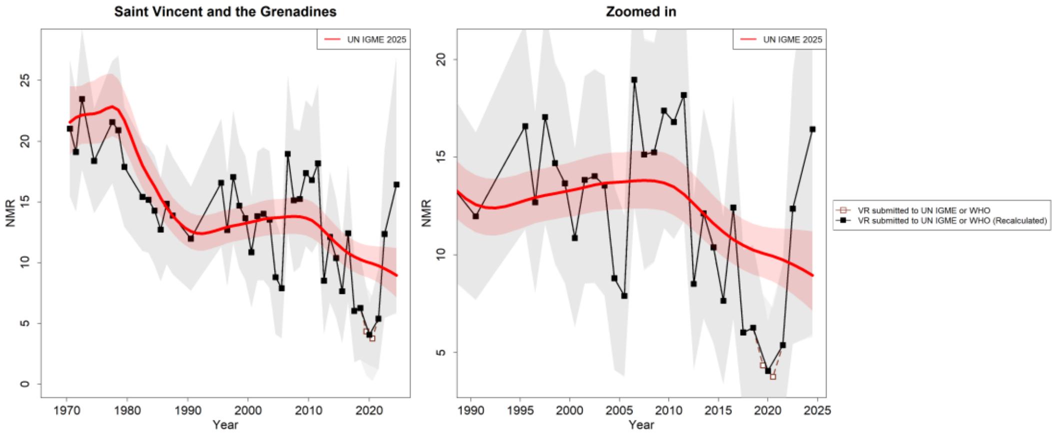

## Samoa (WSM)

### Under-five mortality rate

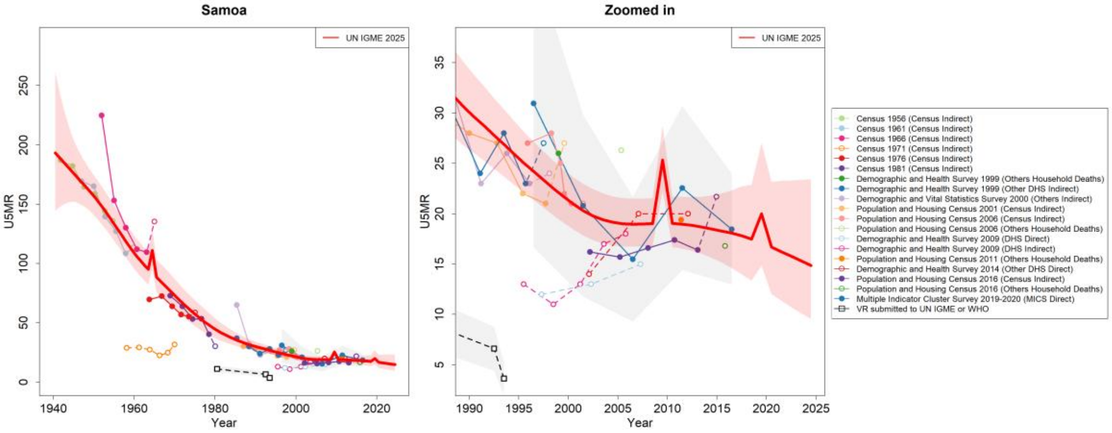


### Infant mortality rate

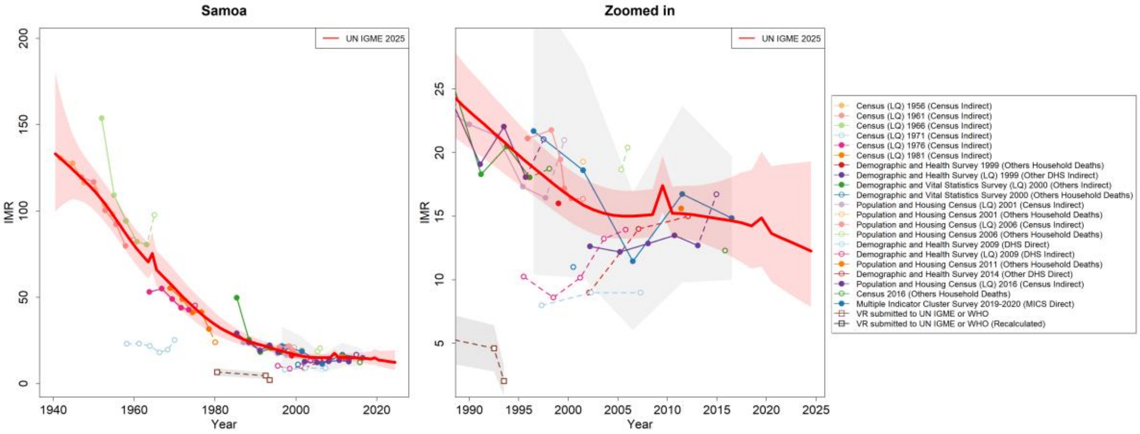


### Neonatal mortality rate

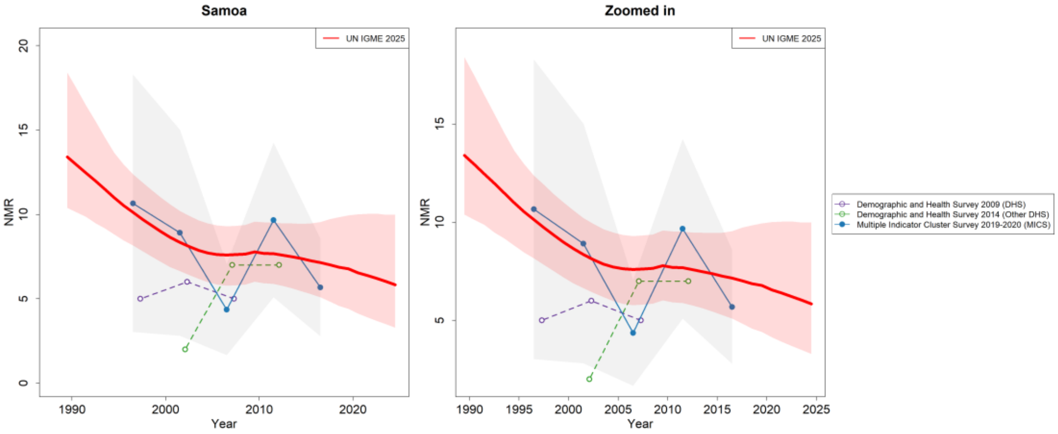

## San Marino (SMR)

### Under-five mortality rate

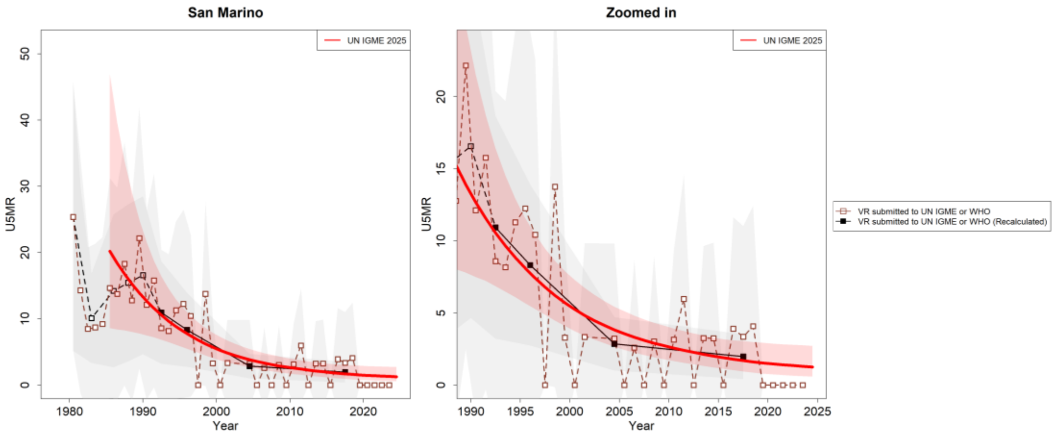


### Infant mortality rate

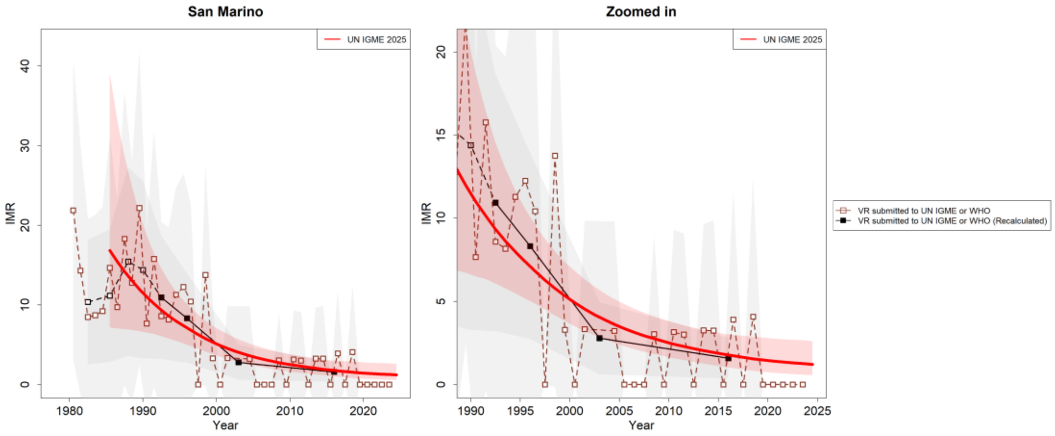


### Neonatal mortality rate

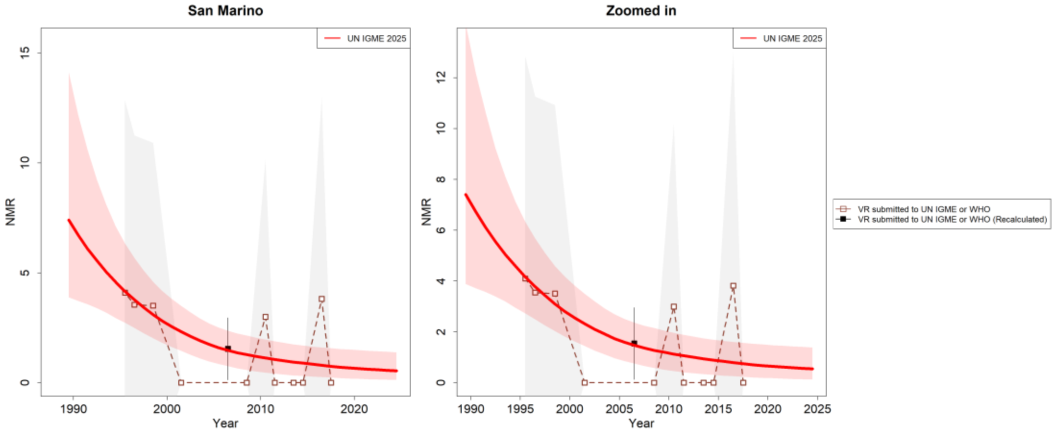

## Sao Tome and Principe (STP)

### Under-five mortality rate

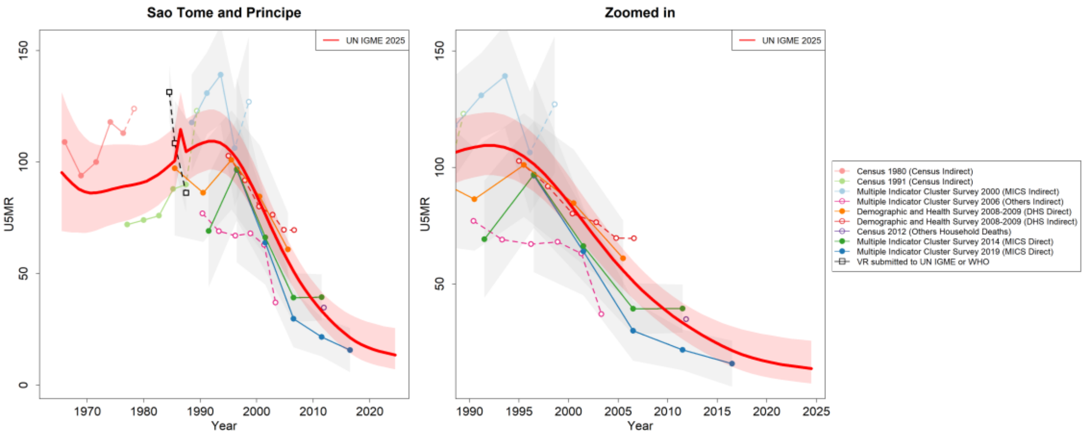


### Infant mortality rate

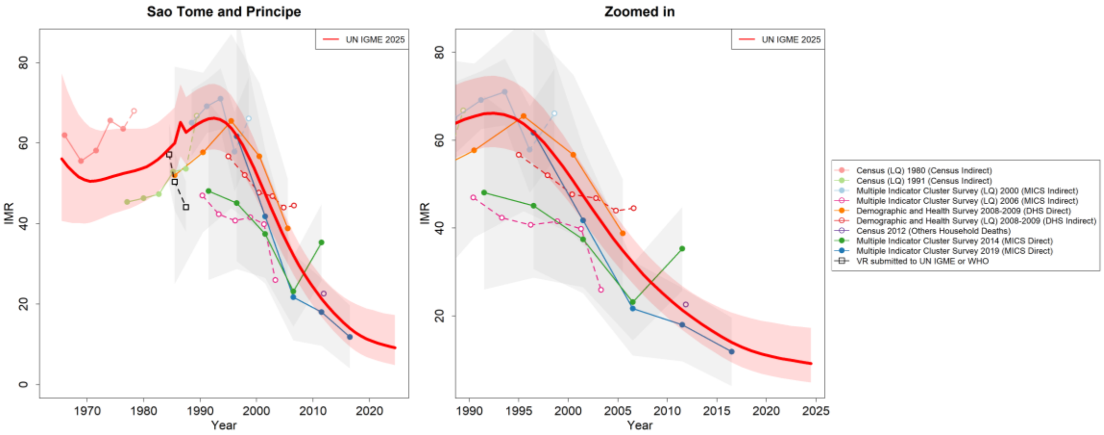


### Neonatal mortality rate

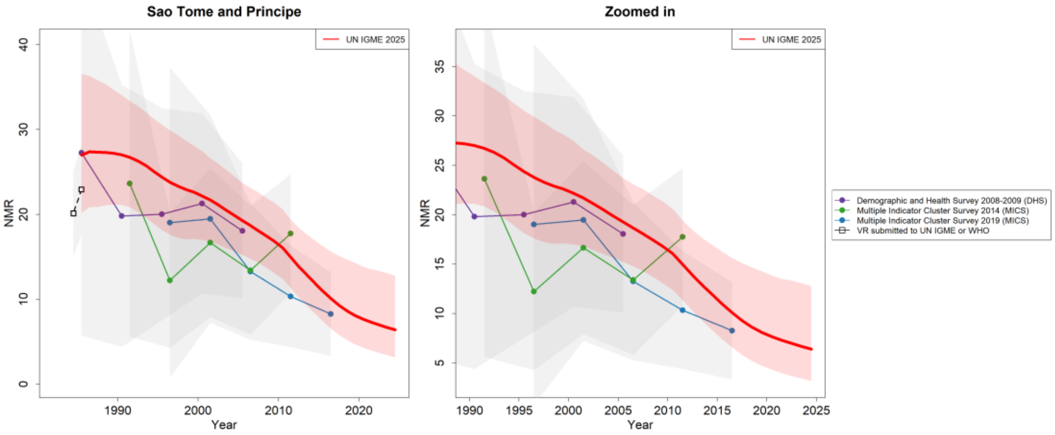

## Saudi Arabia (SAU)

### Under-five mortality rate

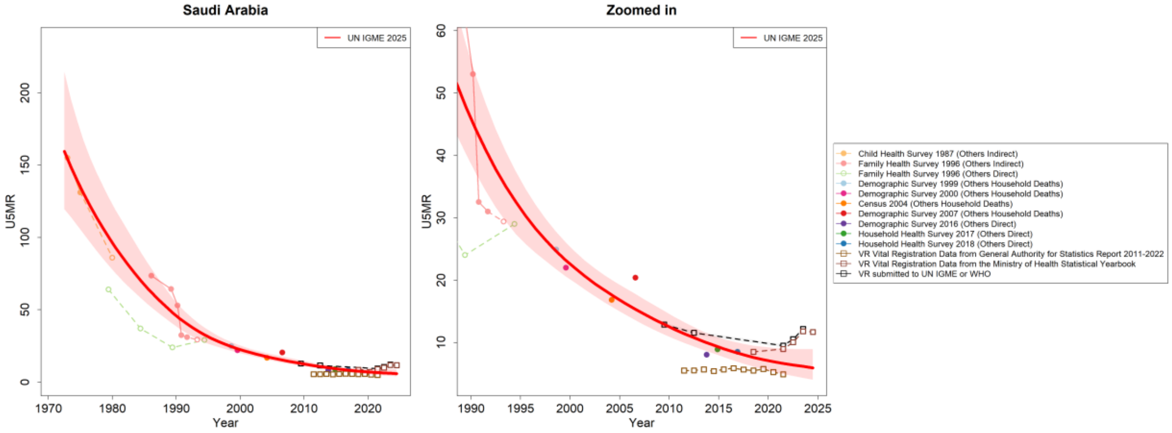


### Infant mortality rate

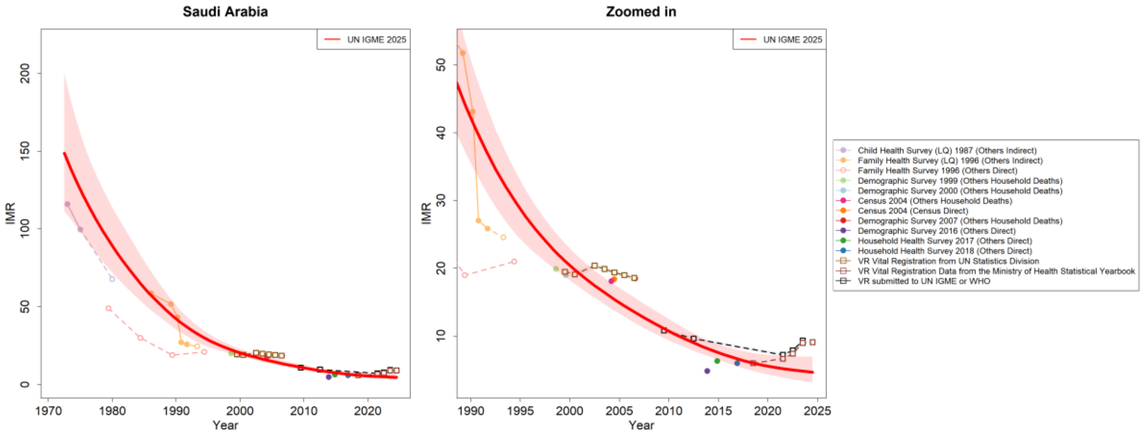


### Neonatal mortality rate

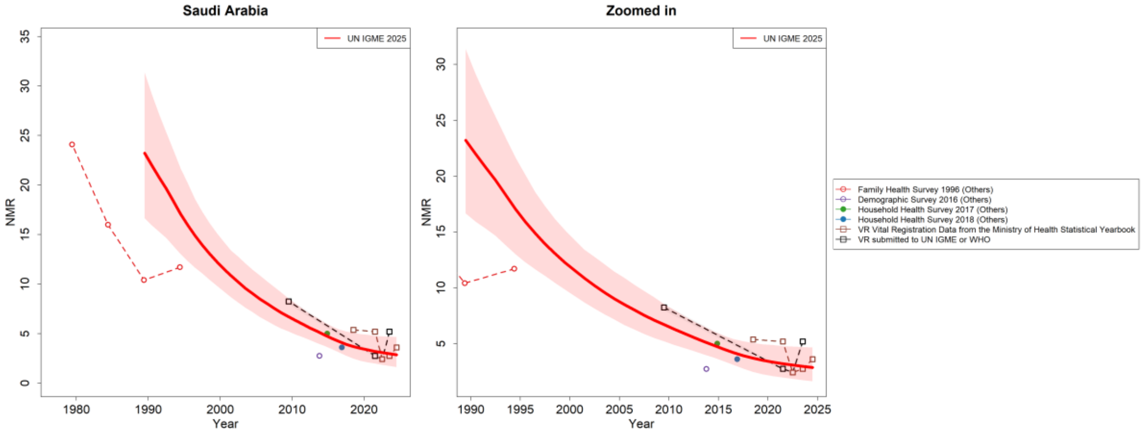

## Senegal (SEN)

### Under-five mortality rate

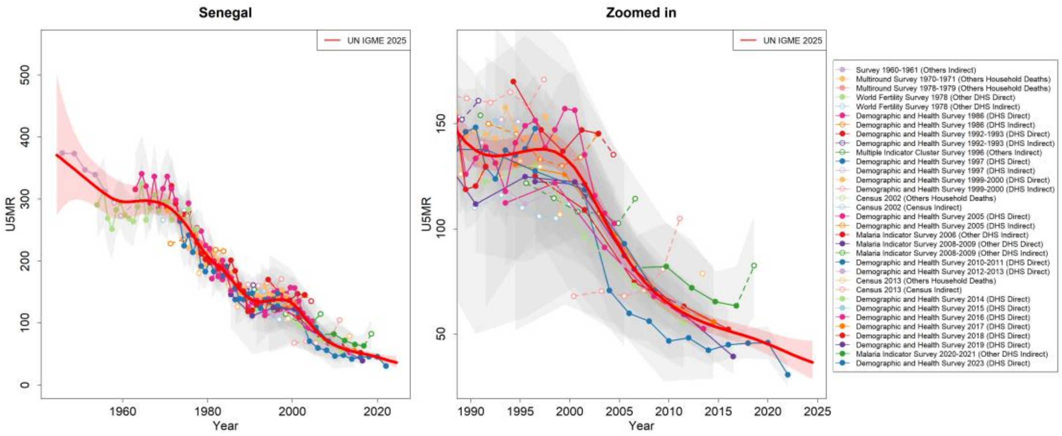


### Infant mortality rate

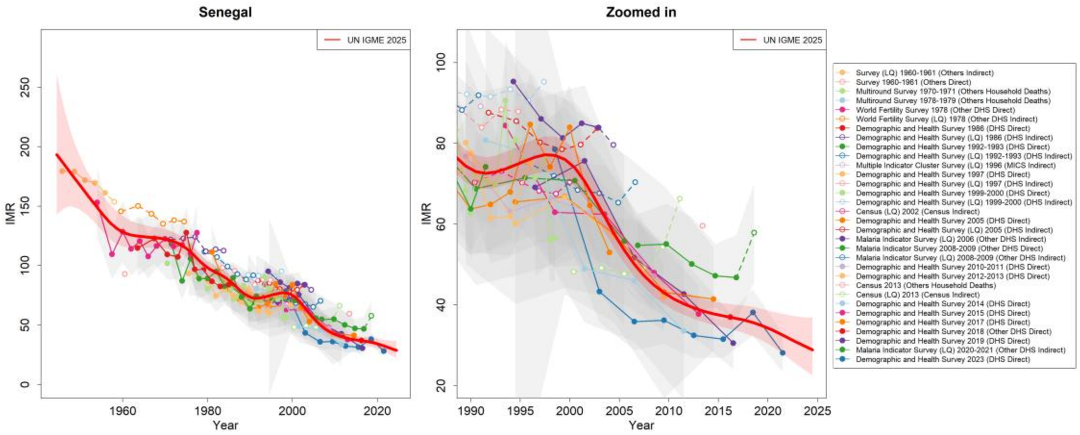


### Neonatal mortality rate

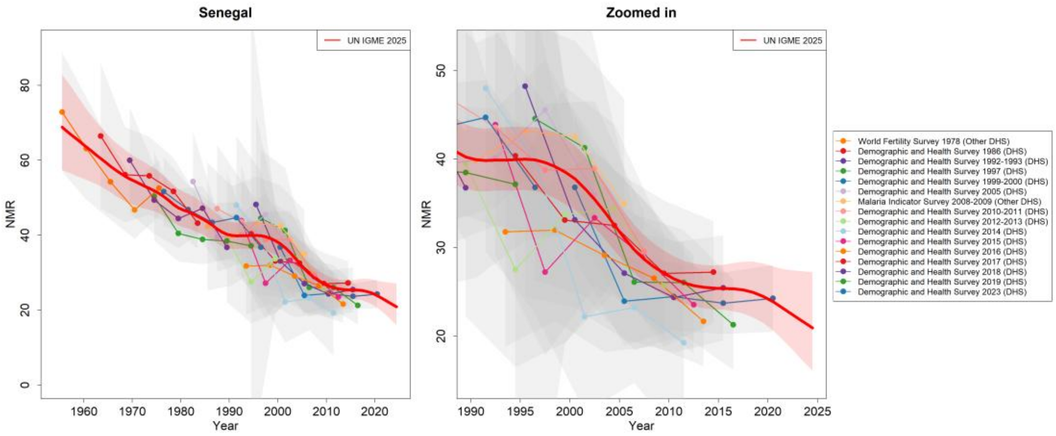

## Serbia (SRB)

### Under-five mortality rate

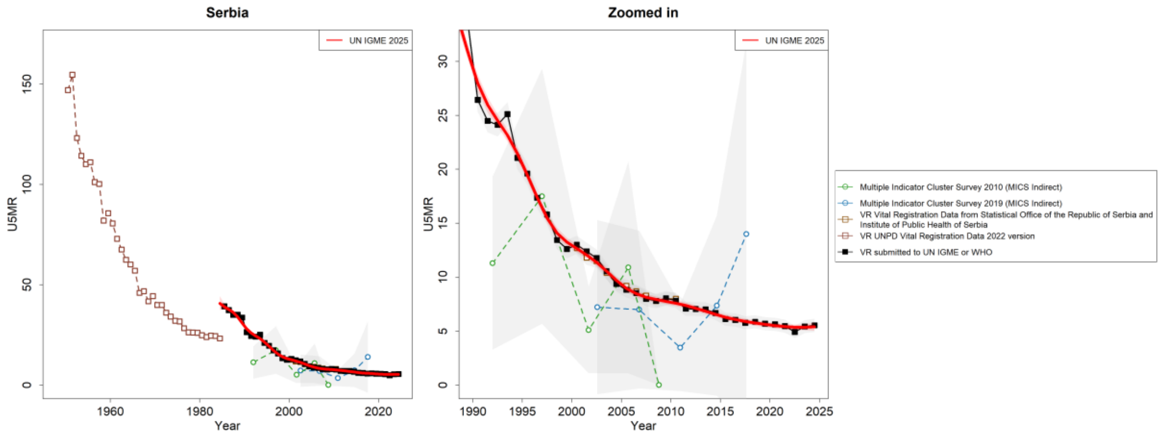


### Infant mortality rate

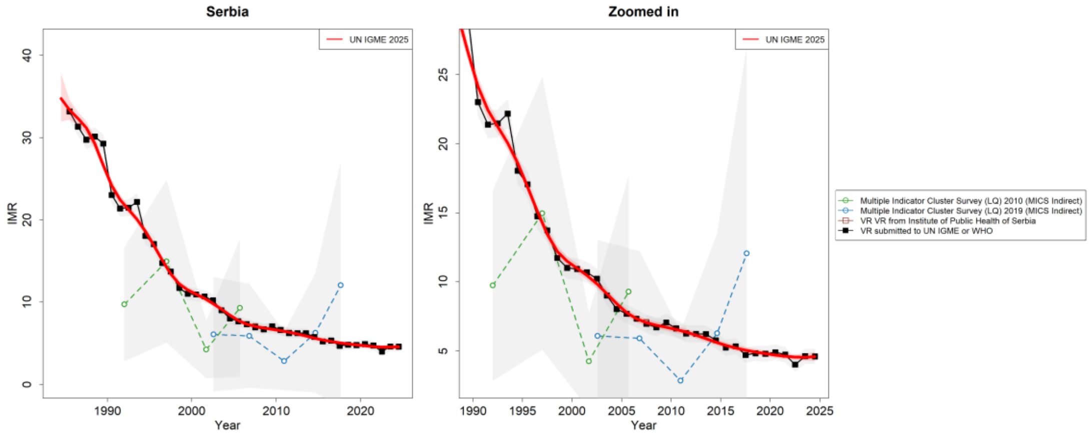


### Neonatal mortality rate

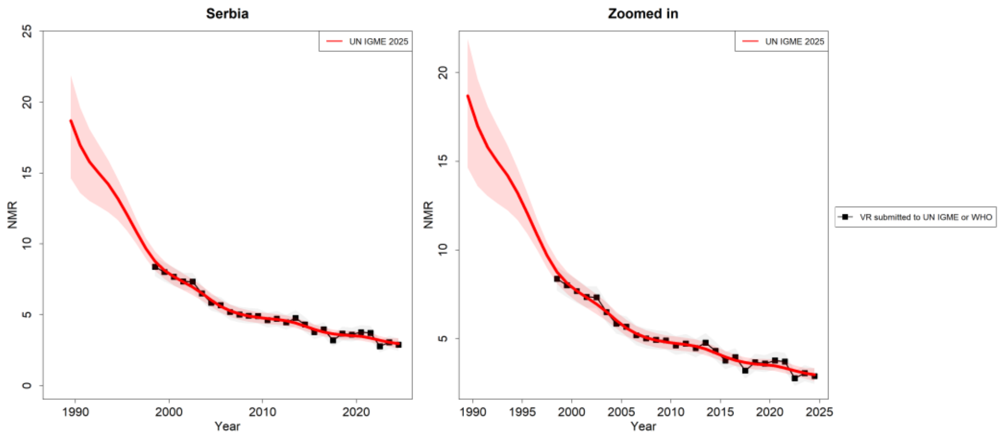

## Seychelles (SYC)

### Under-five mortality rate

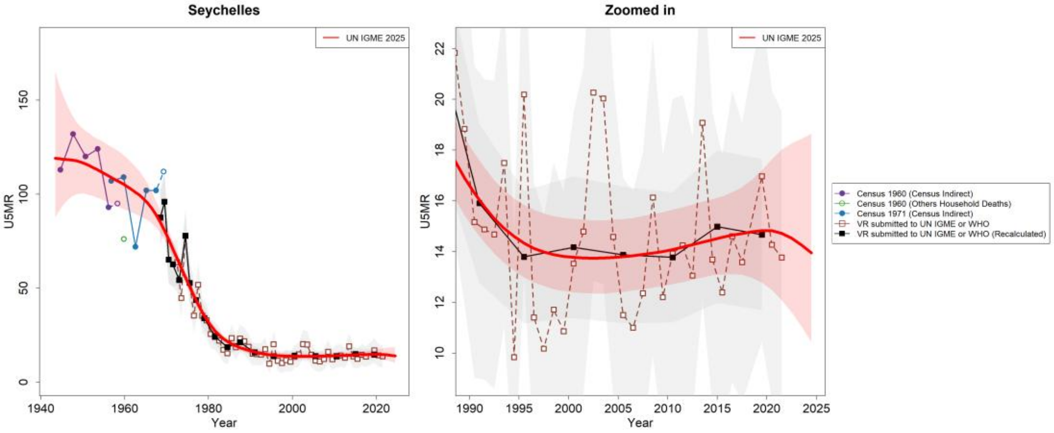


### Infant mortality rate

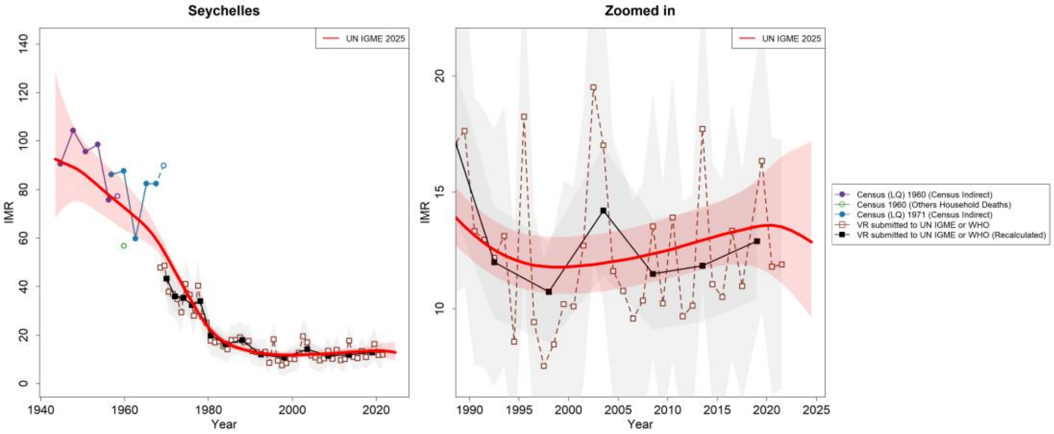


### Neonatal mortality rate

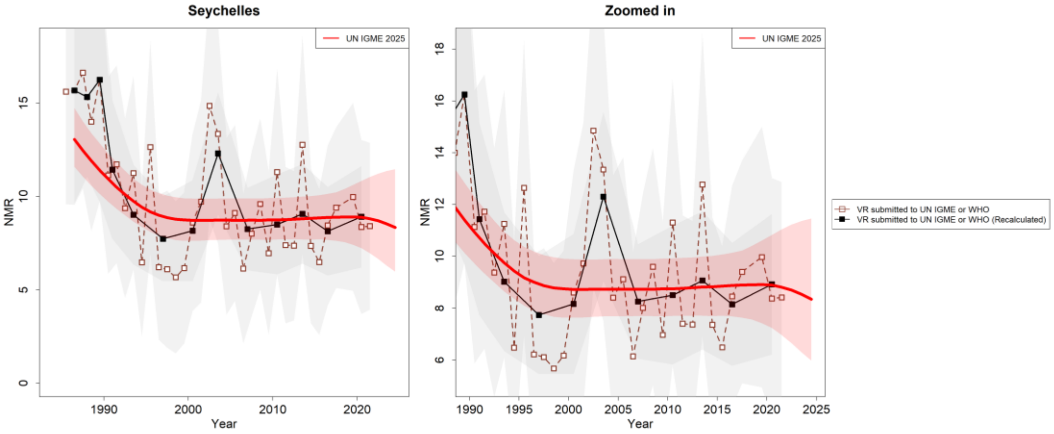

## Sierra Leone (SLE)

### Under-five mortality rate

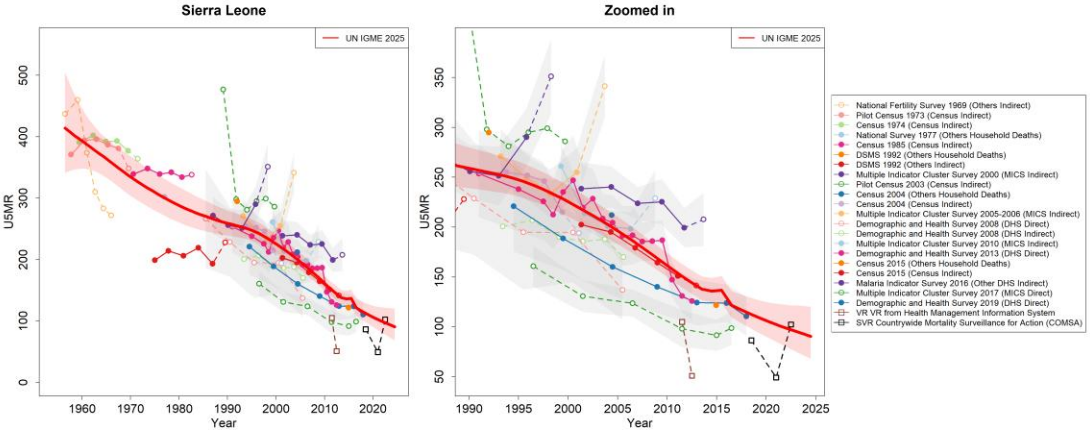


### Infant mortality rate

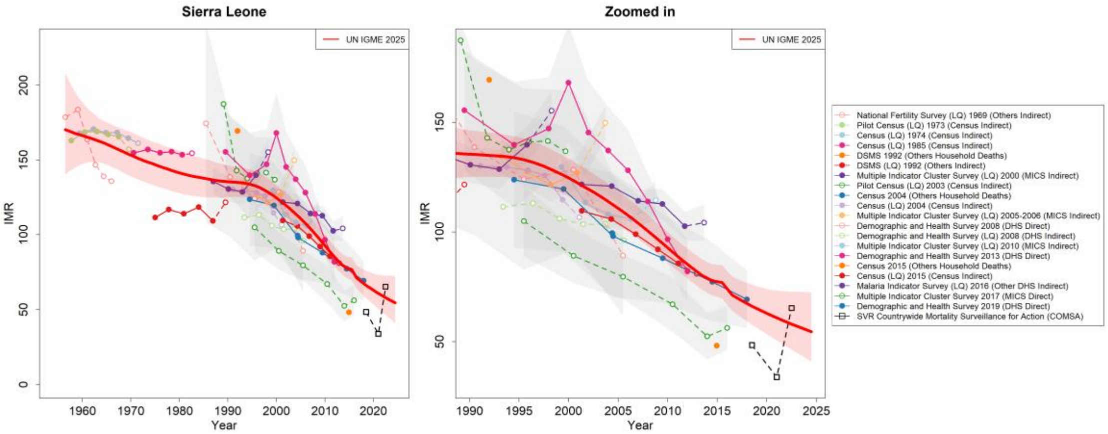


### Neonatal mortality rate

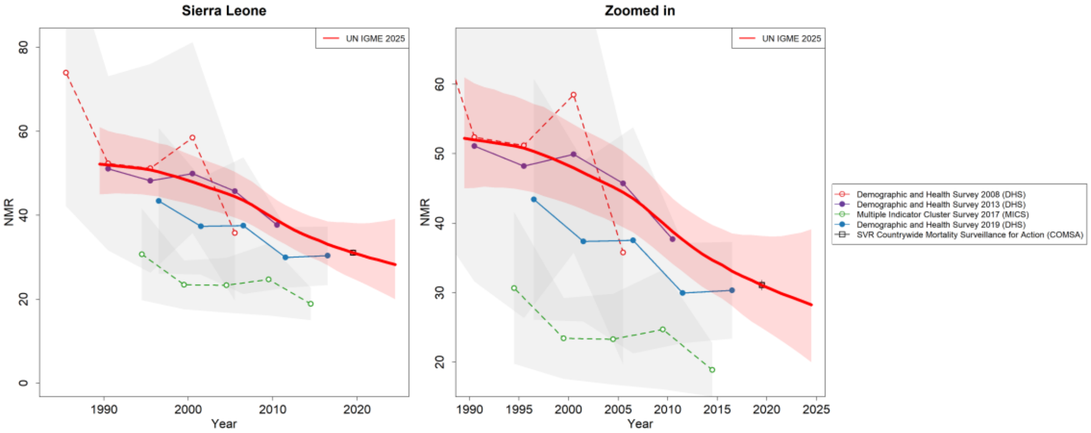

## Singapore (SGP)

### Under-five mortality rate

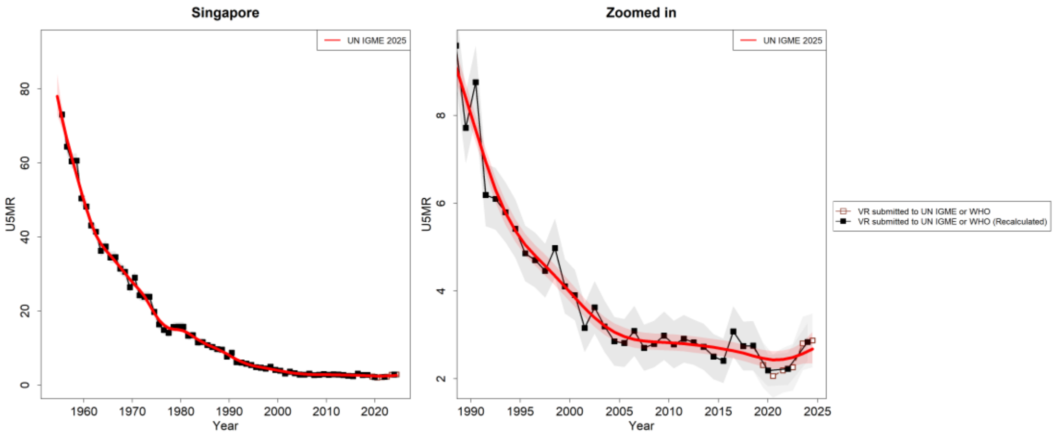


### Infant mortality rate

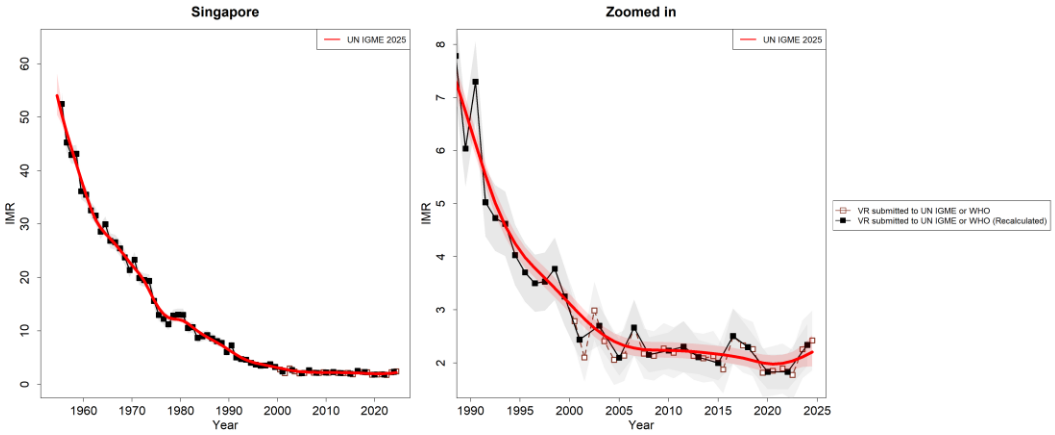


### Neonatal mortality rate

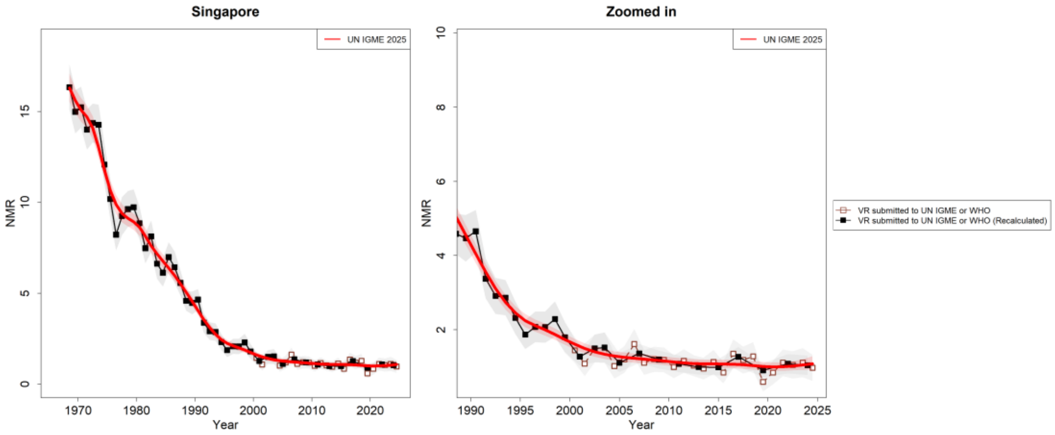

## Slovakia (SVK)

### Under-five mortality rate

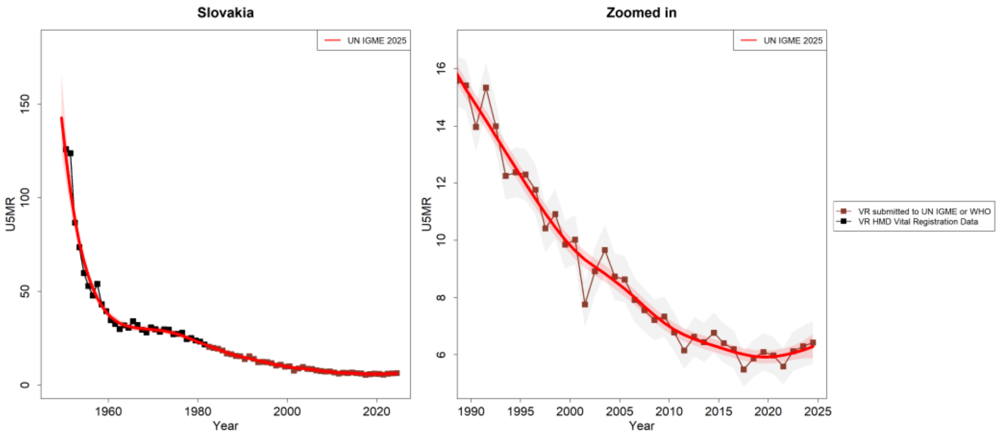


### Infant mortality rate

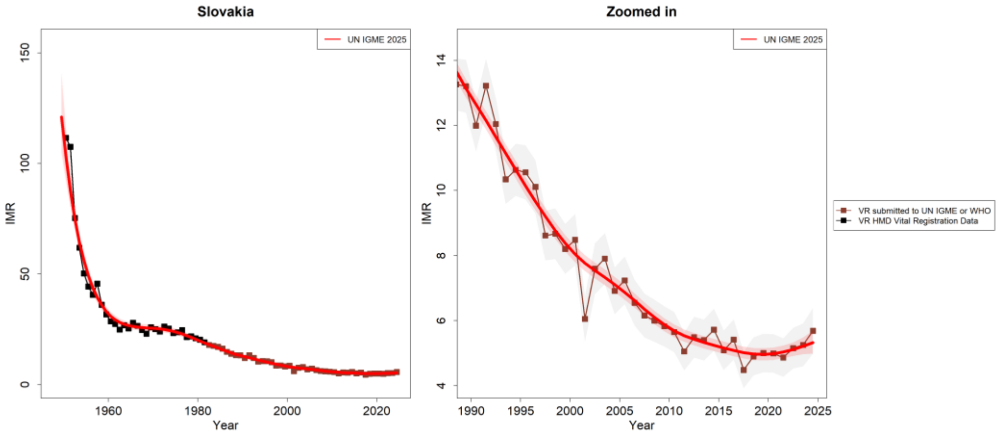


### Neonatal mortality rate

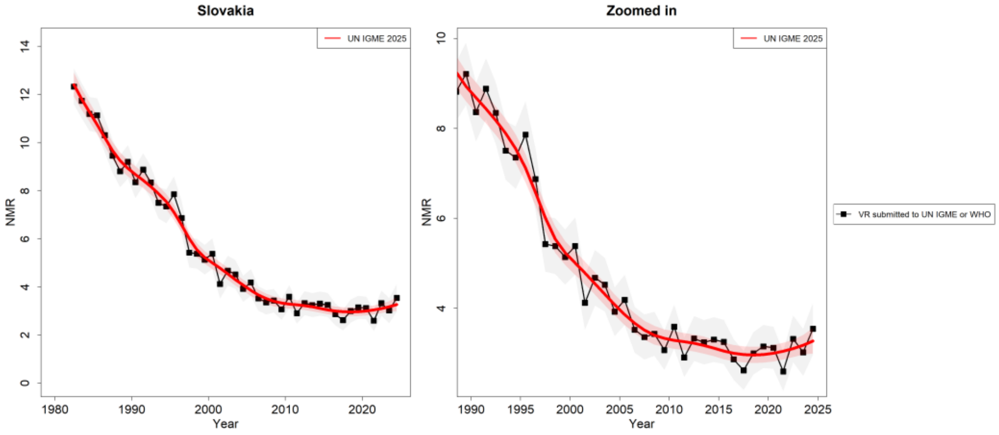

## Slovenia (SVN)

### Under-five mortality rate

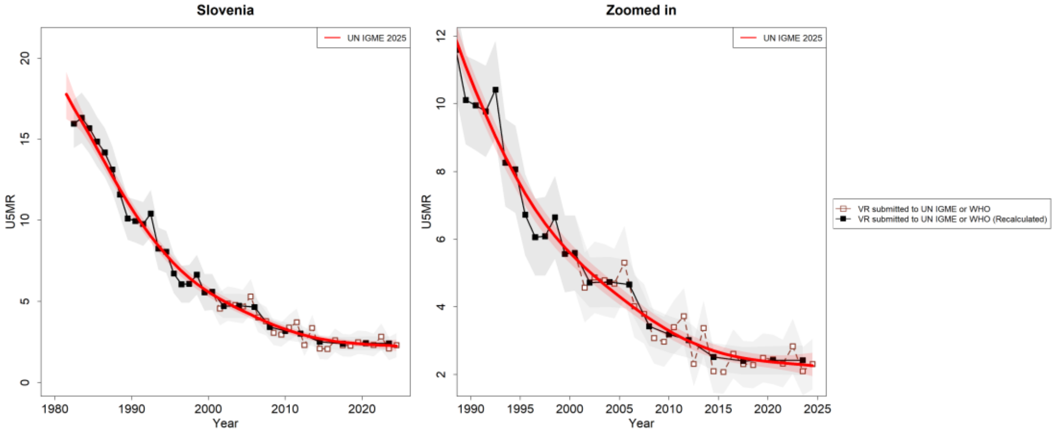


### Infant mortality rate

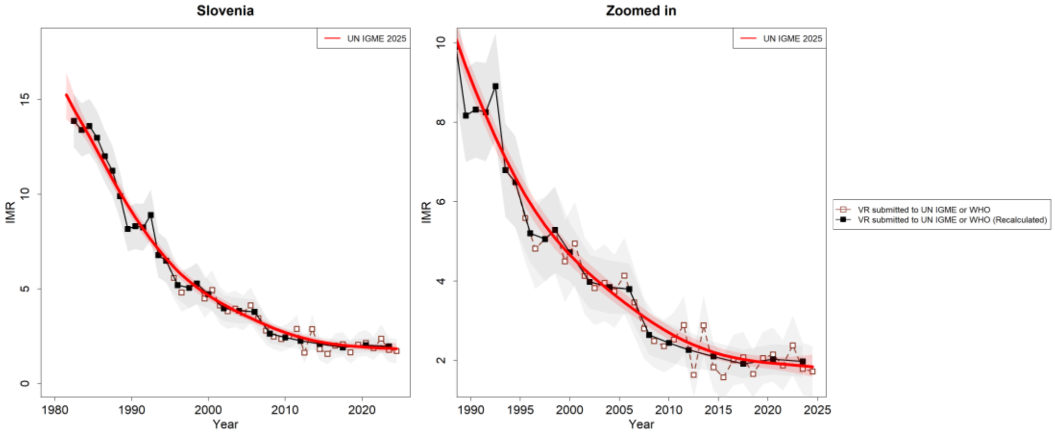


### Neonatal mortality rate

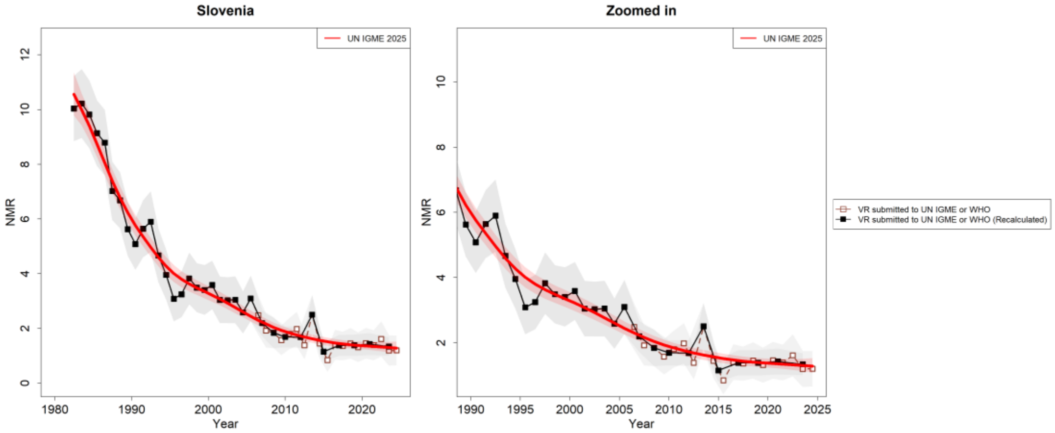

## Solomon Islands (SLB)

### Under-five mortality rate

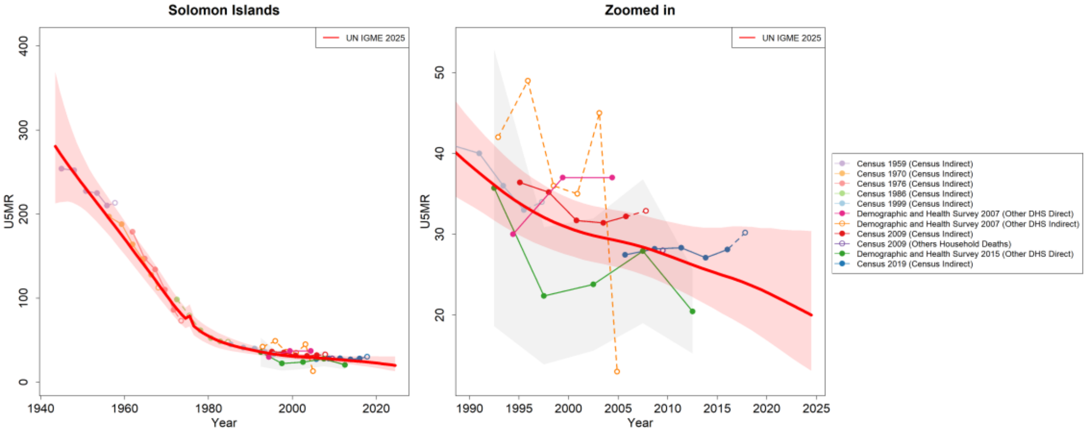


### Infant mortality rate

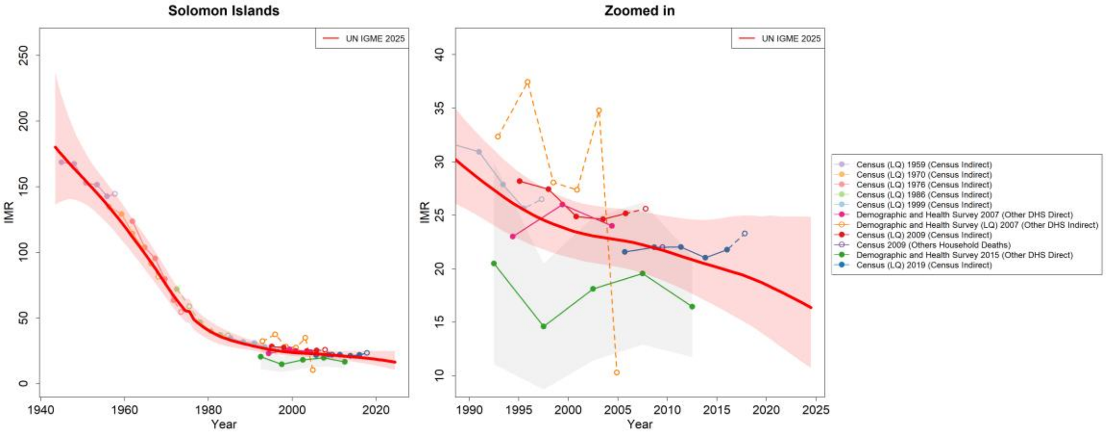


### Neonatal mortality rate

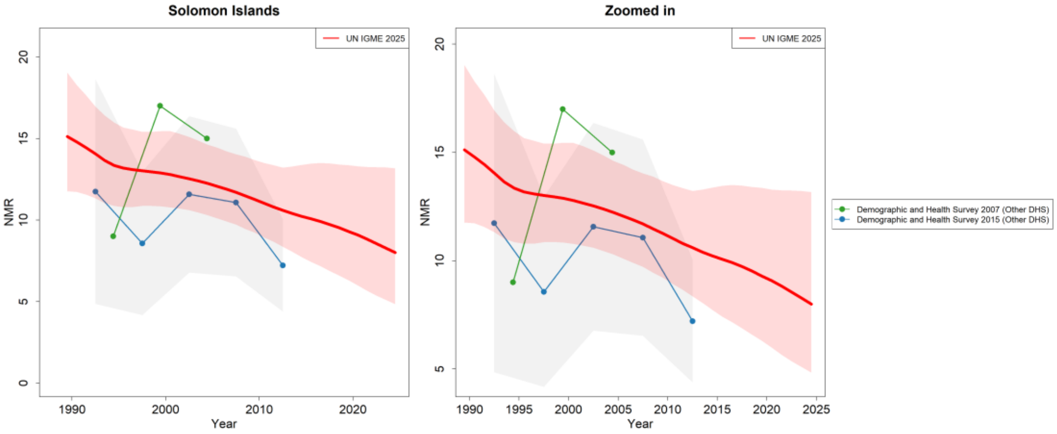

## Somalia (SOM)

### Under-five mortality rate

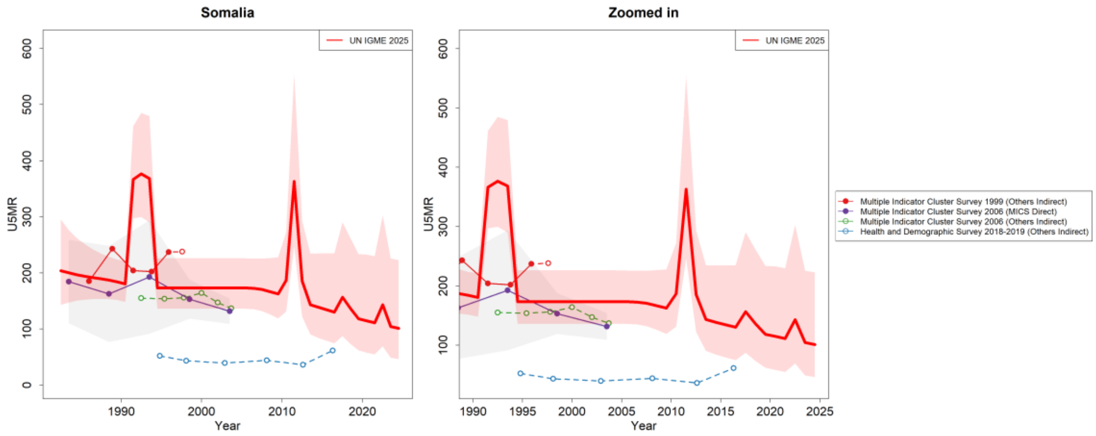


### Infant mortality rate

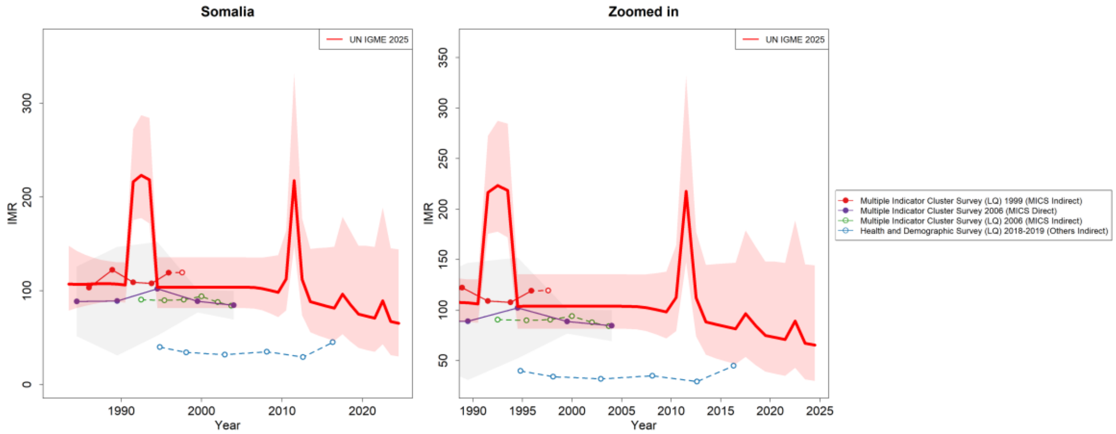


### Neonatal mortality rate

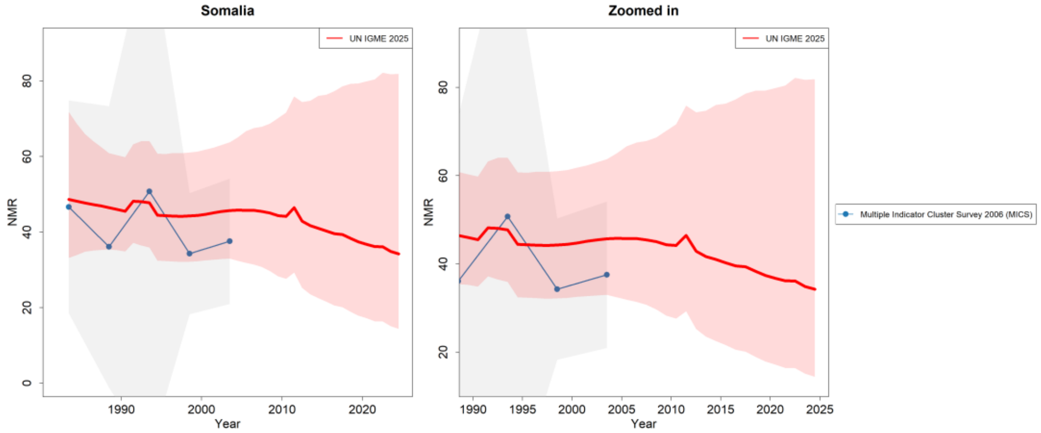

## South Africa (ZAF)

### Under-five mortality rate

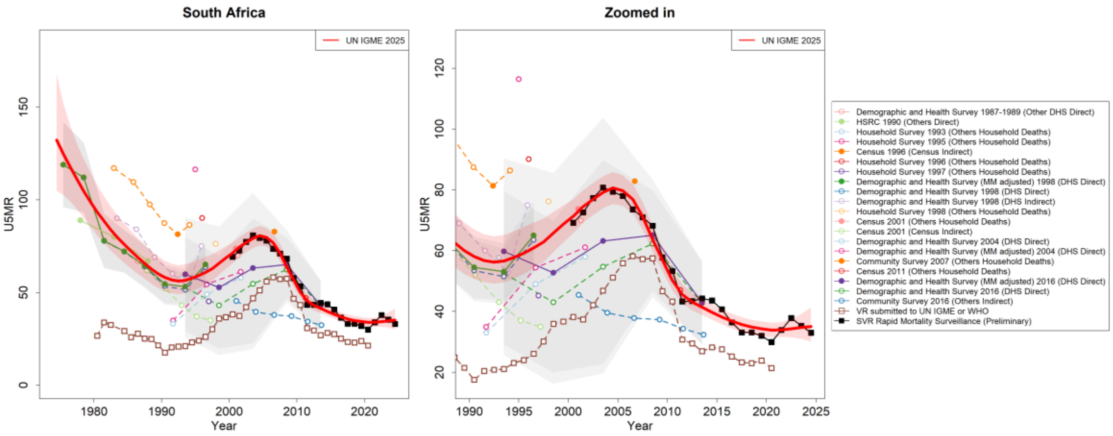


### Infant mortality rate

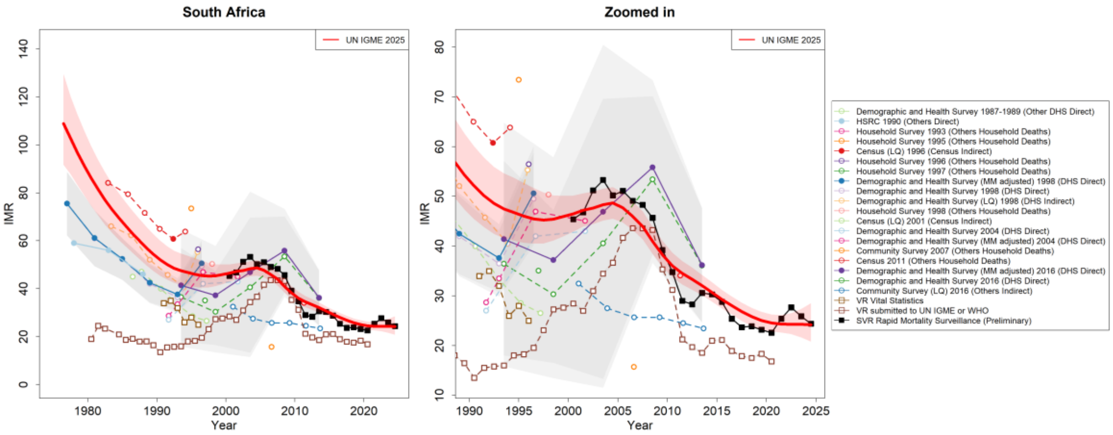


### Neonatal mortality rate

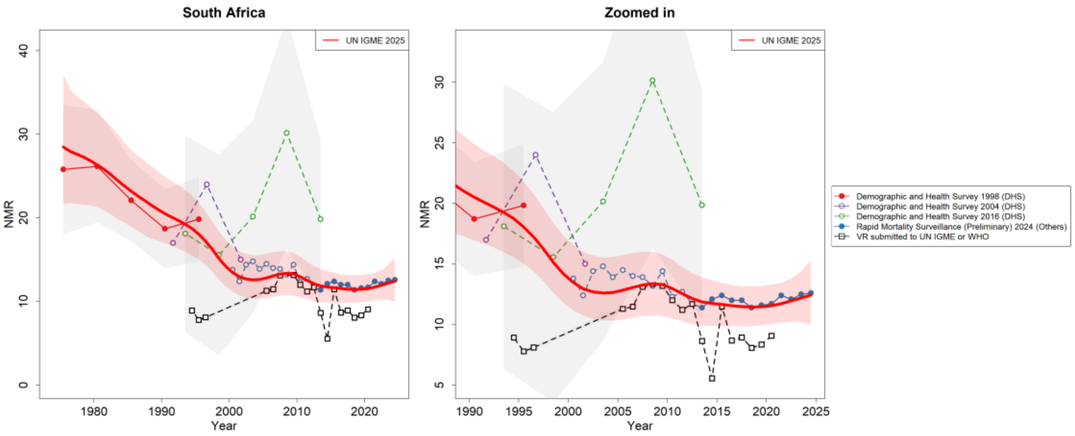

## South Sudan (SSD)

### Under-five mortality rate

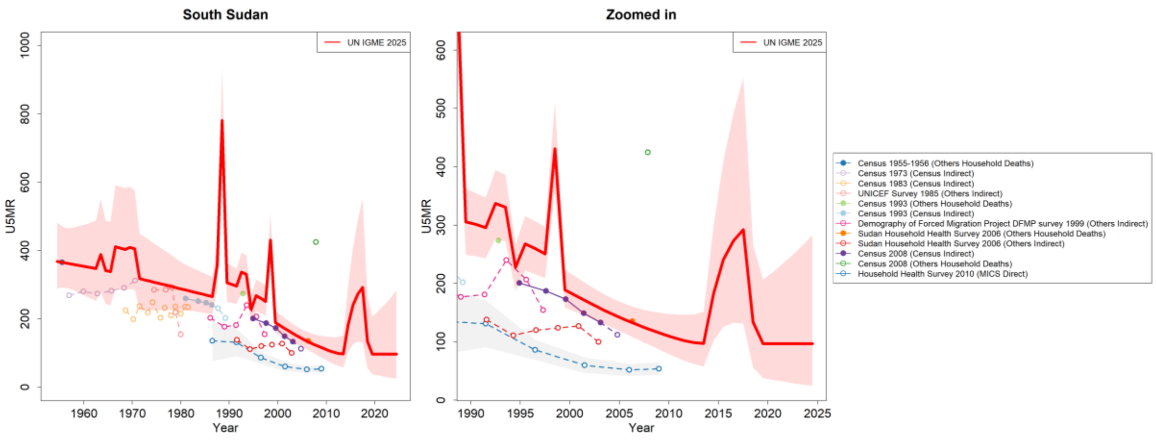


### Infant mortality rate

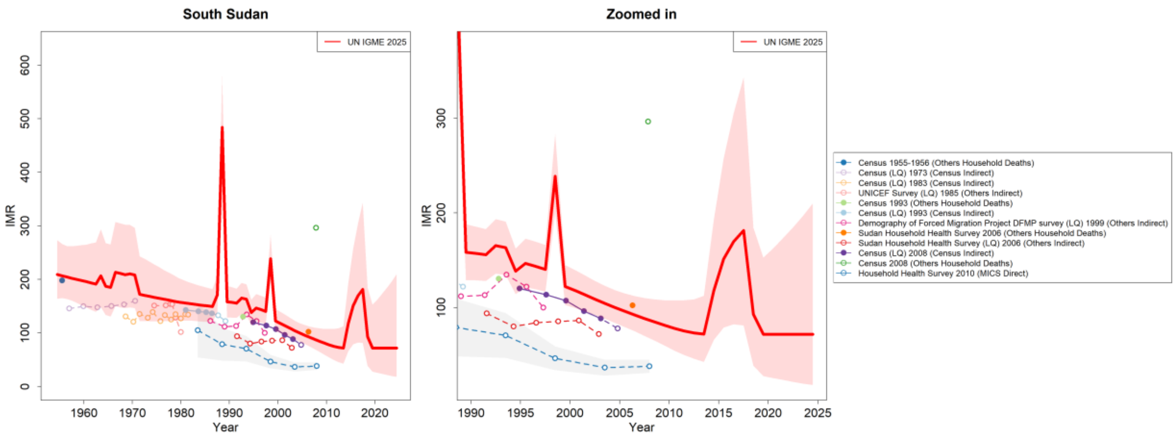


### Neonatal mortality rate

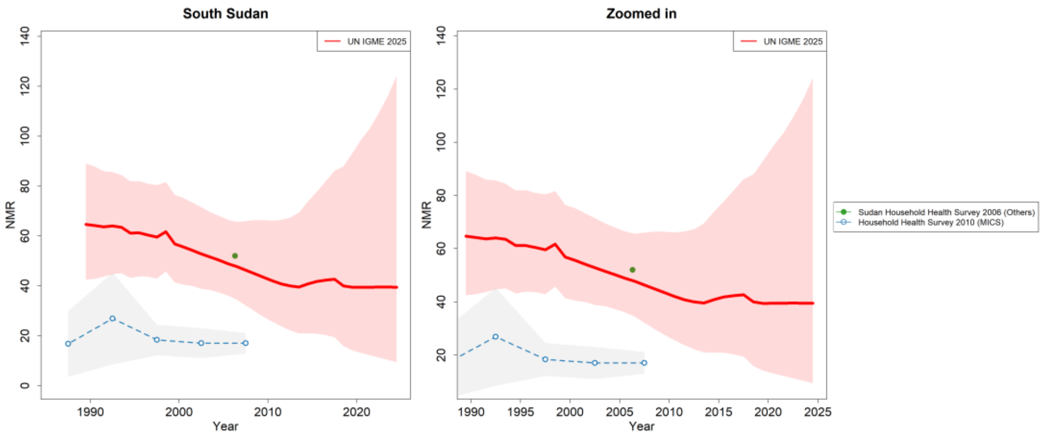

## Spain (ESP)

### Under-five mortality rate

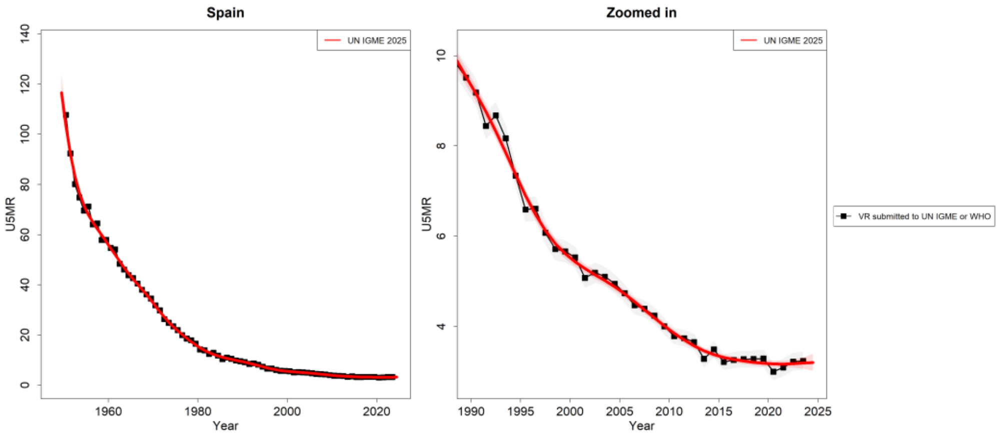


### Infant mortality rate

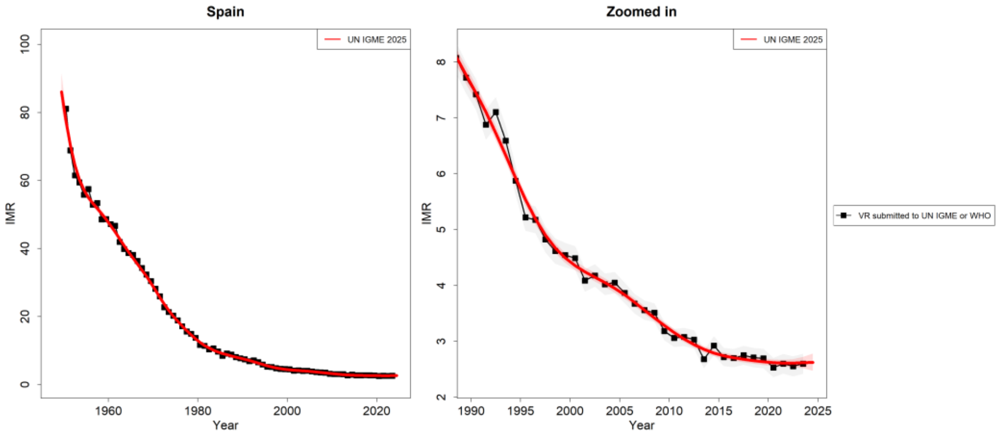


### Neonatal mortality rate

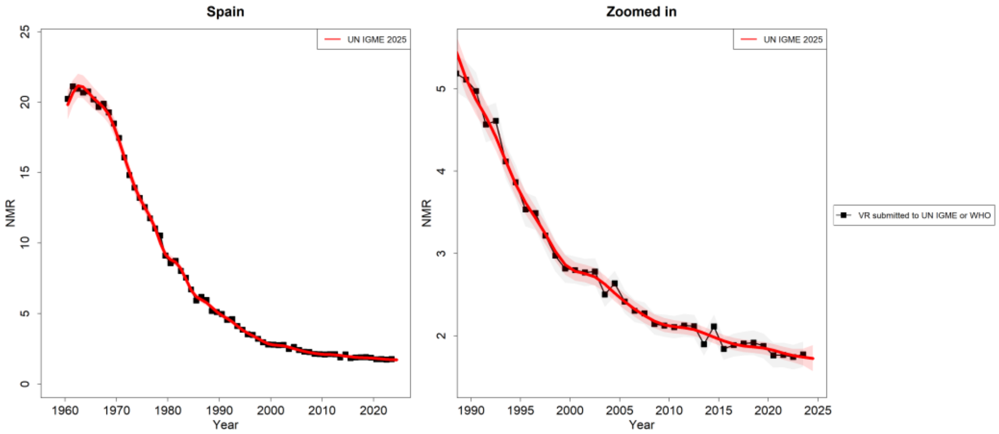

## Sri Lanka (LKA)

### Under-five mortality rate

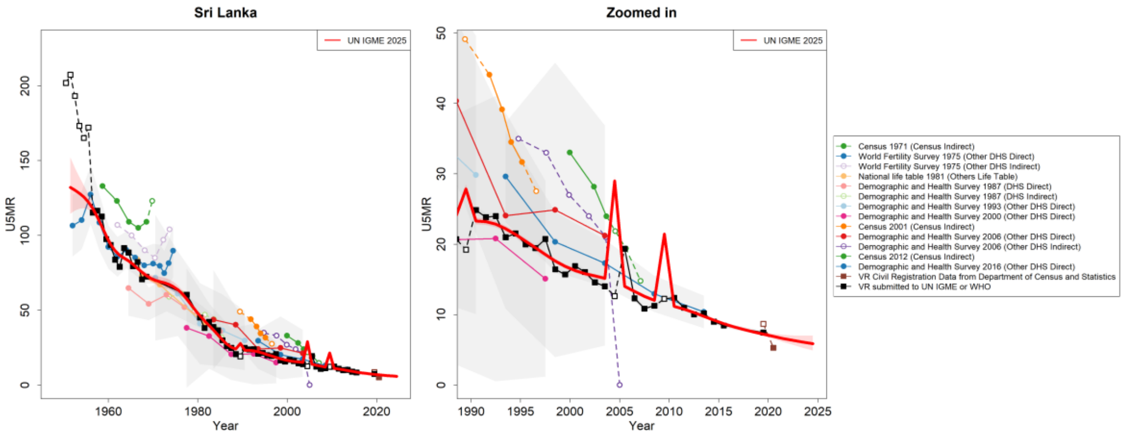


### Infant mortality rate

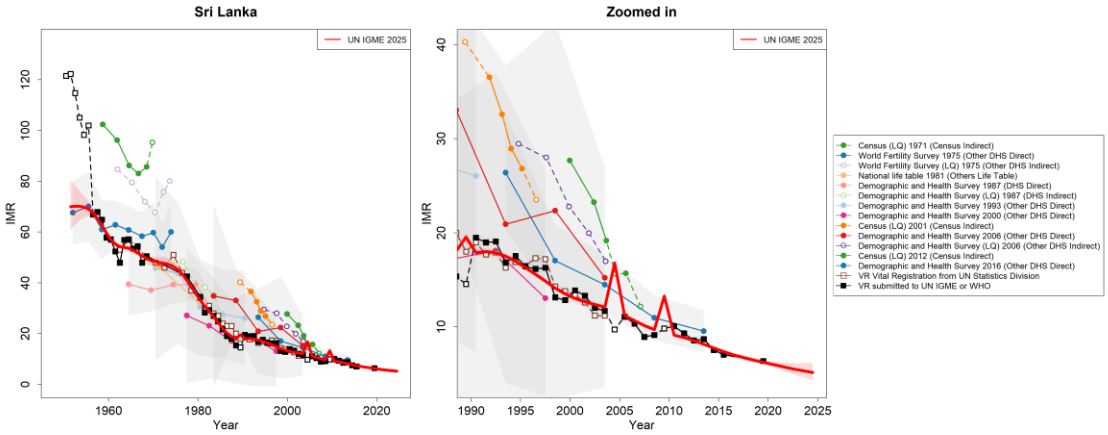


### Neonatal mortality rate

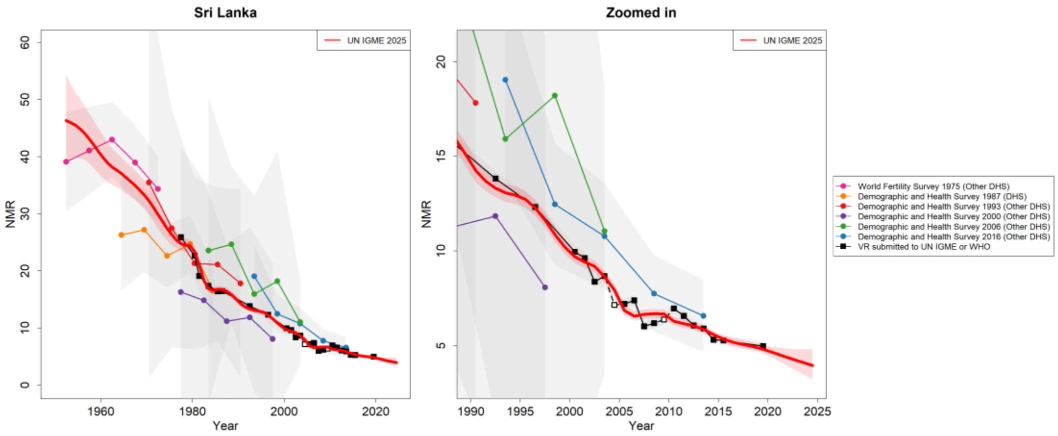

## State of Palestine (PSE)

### Under-five mortality rate

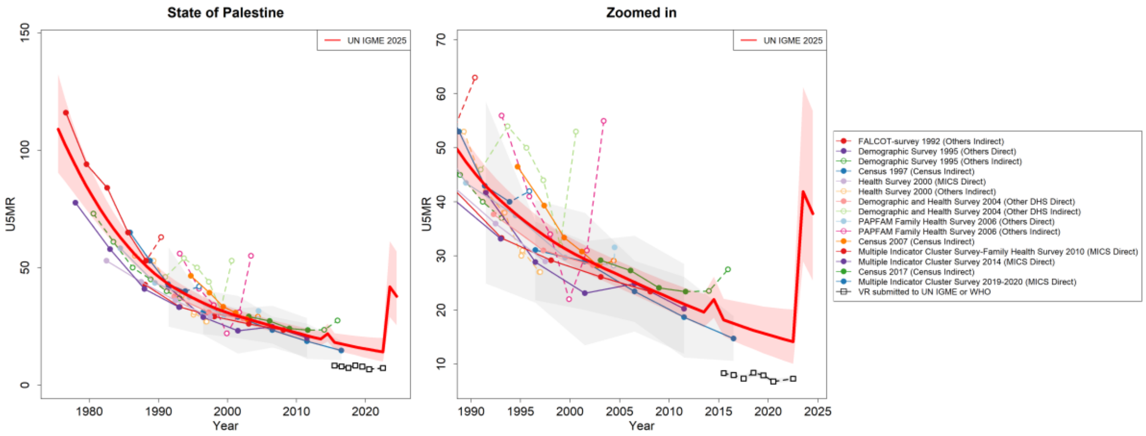


### Infant mortality rate

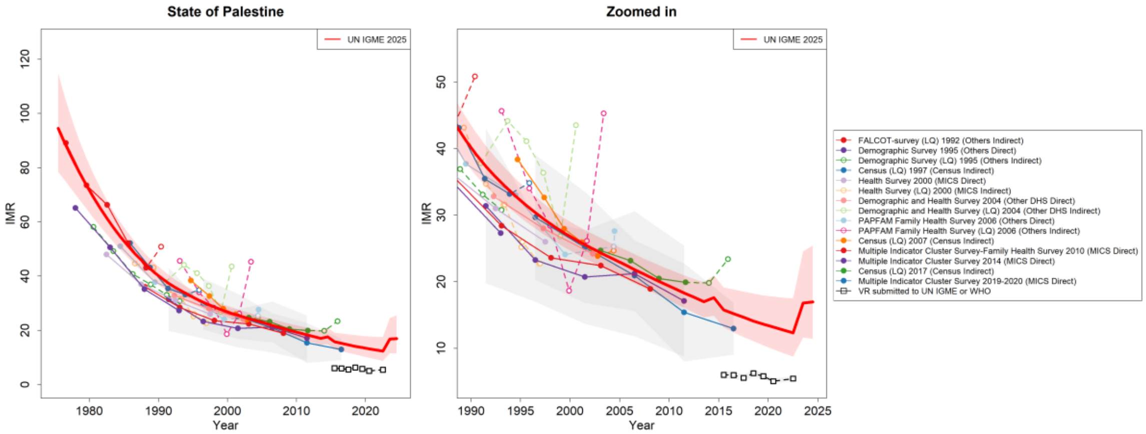


### Neonatal mortality rate

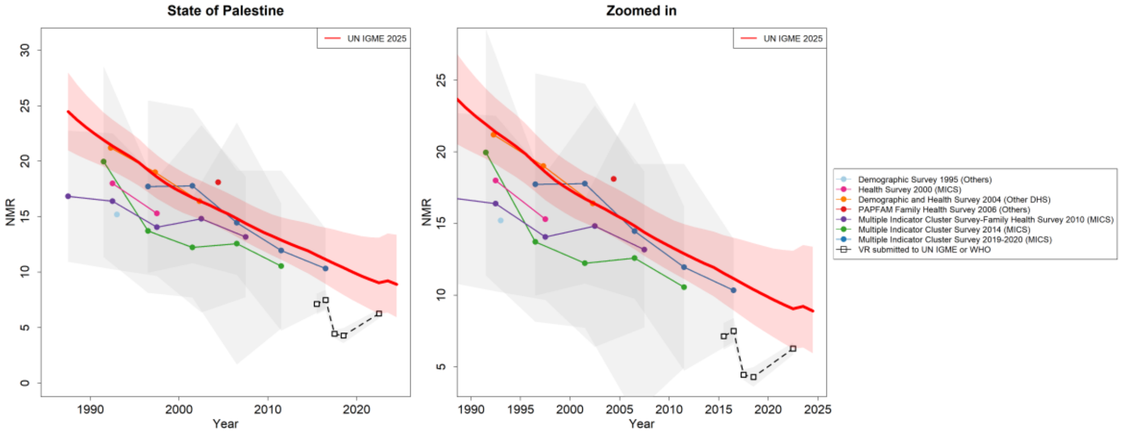

## Sudan (SDN)

### Under-five mortality rate

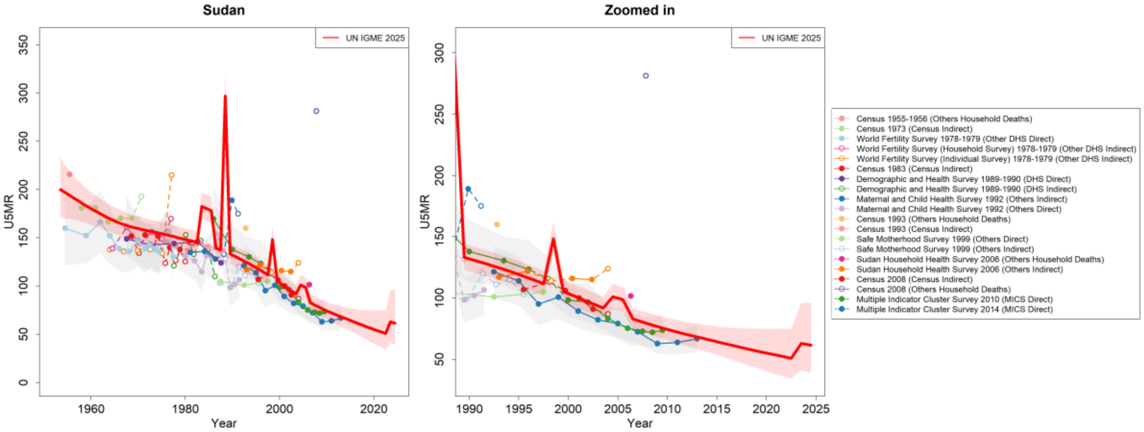


### Infant mortality rate

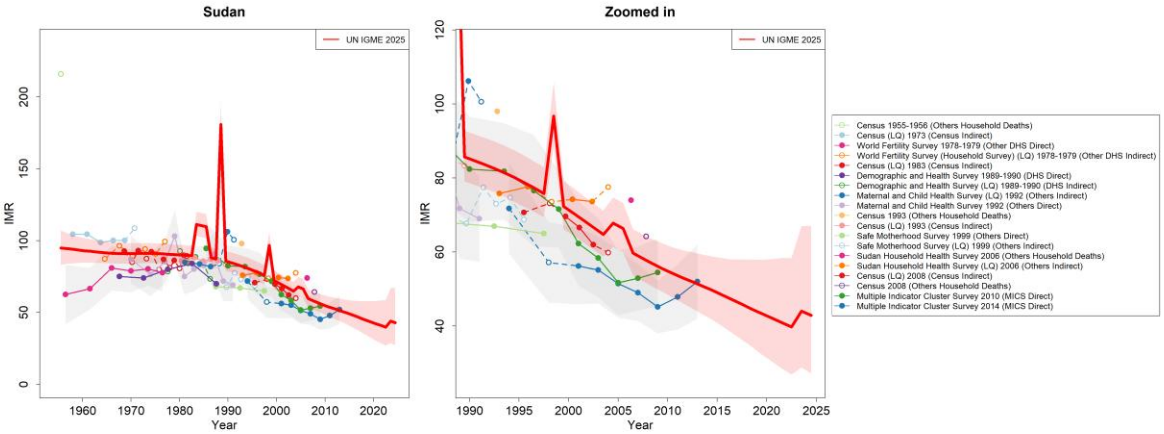


### Neonatal mortality rate

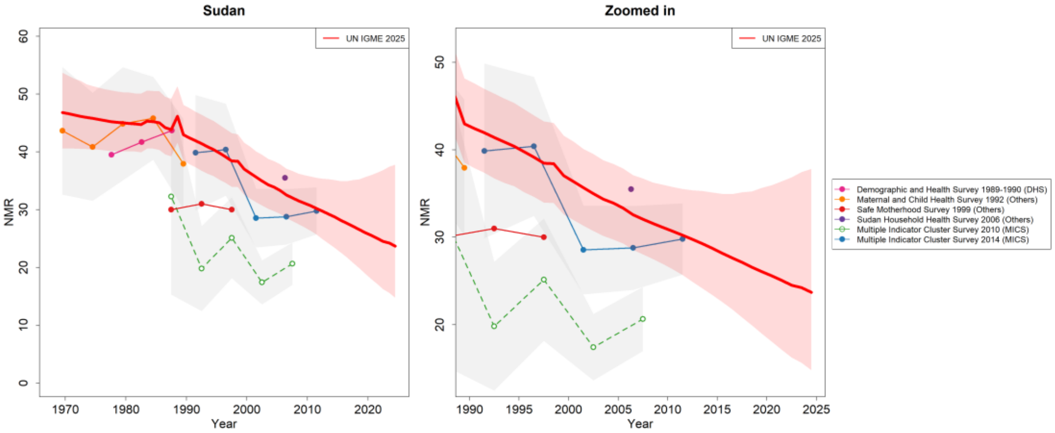

## Suriname (SUR)

### Under-five mortality rate

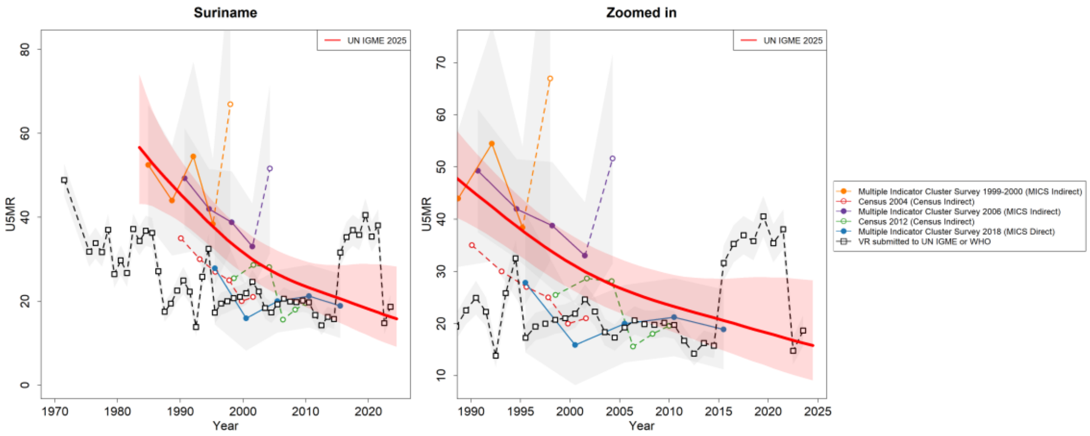


### Infant mortality rate

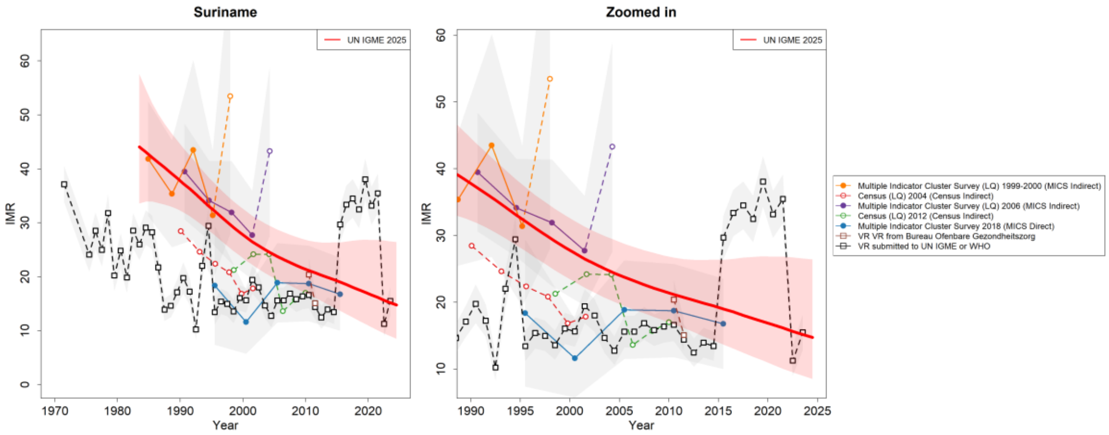


### Neonatal mortality rate

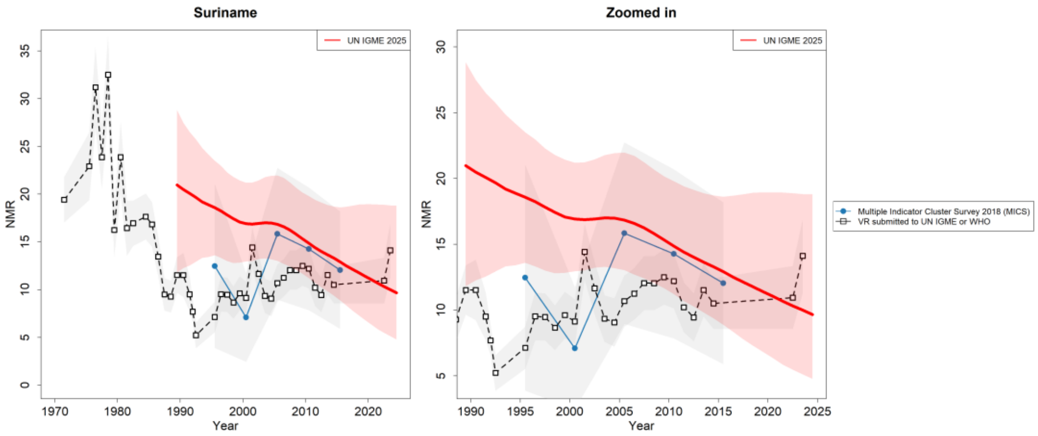

## Sweden (SWE)

### Under-five mortality rate

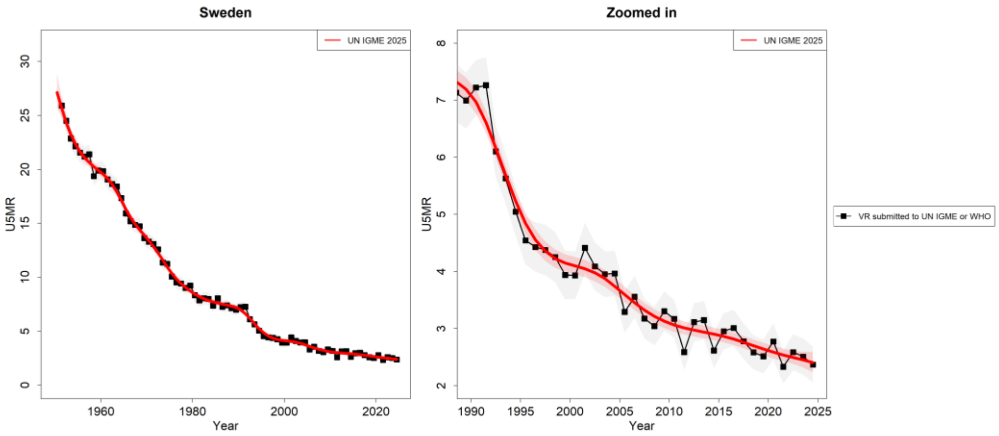


### Infant mortality rate

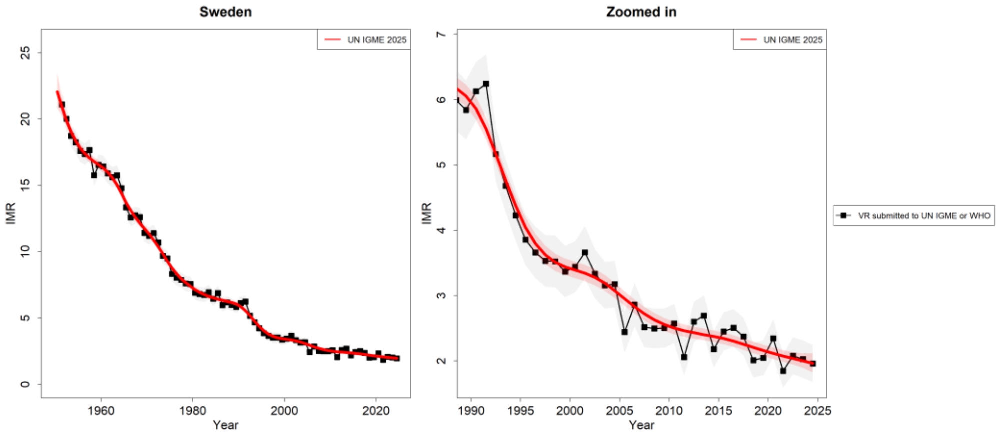


### Neonatal mortality rate

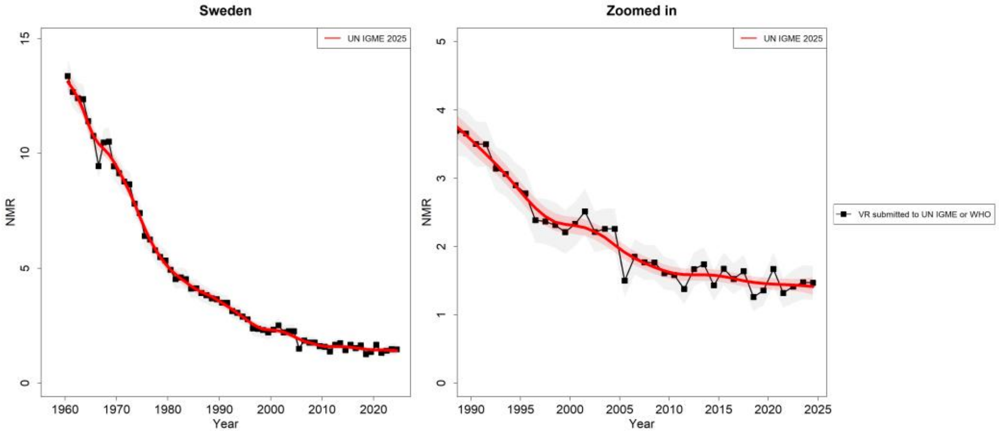

## Switzerland (CHE)

### Under-five mortality rate

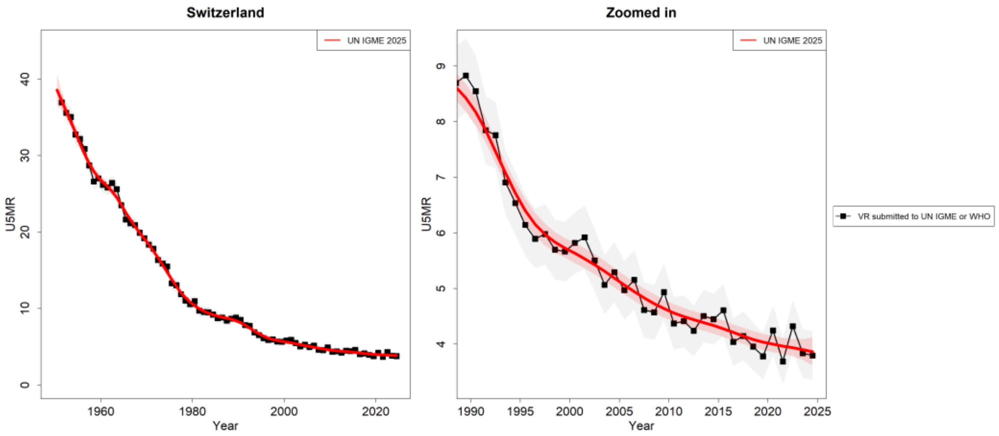


### Infant mortality rate

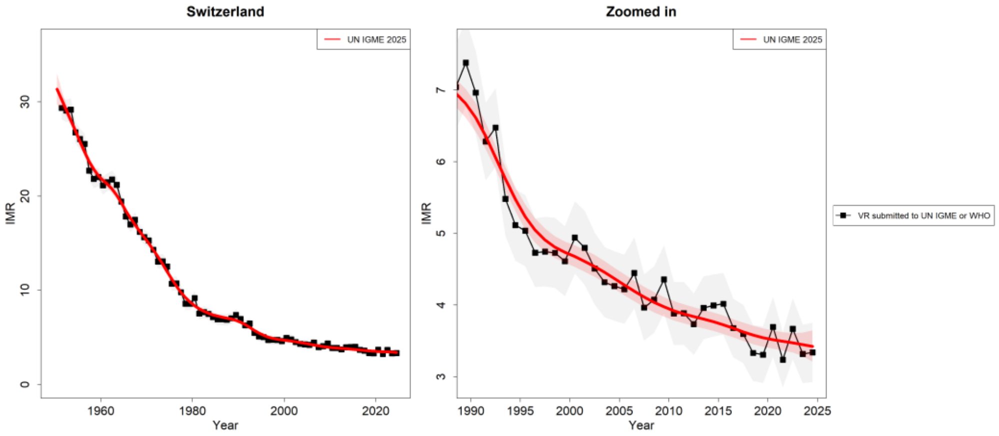


### Neonatal mortality rate

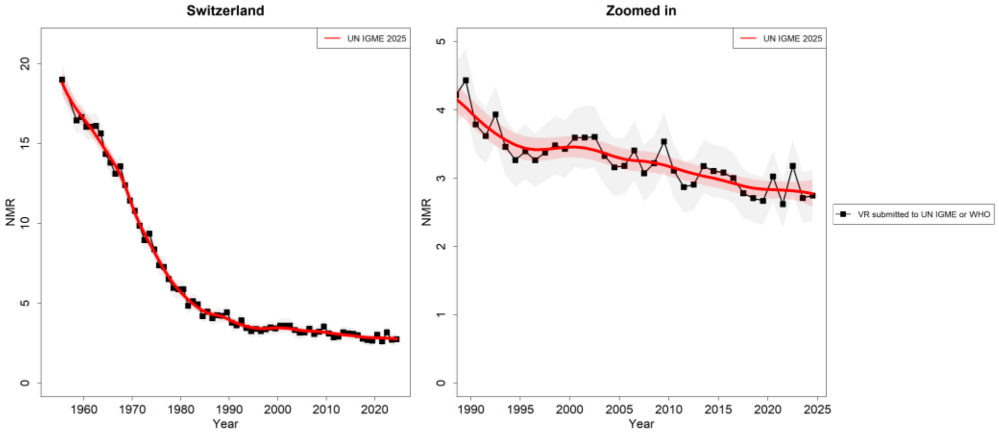

## Syrian Arab Republic (SYR)

### Under-five mortality rate

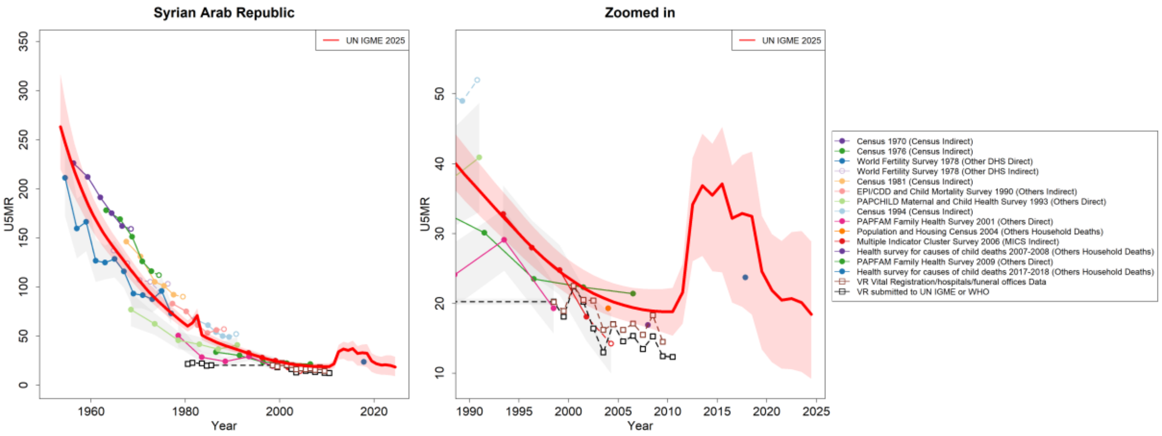


### Infant mortality rate

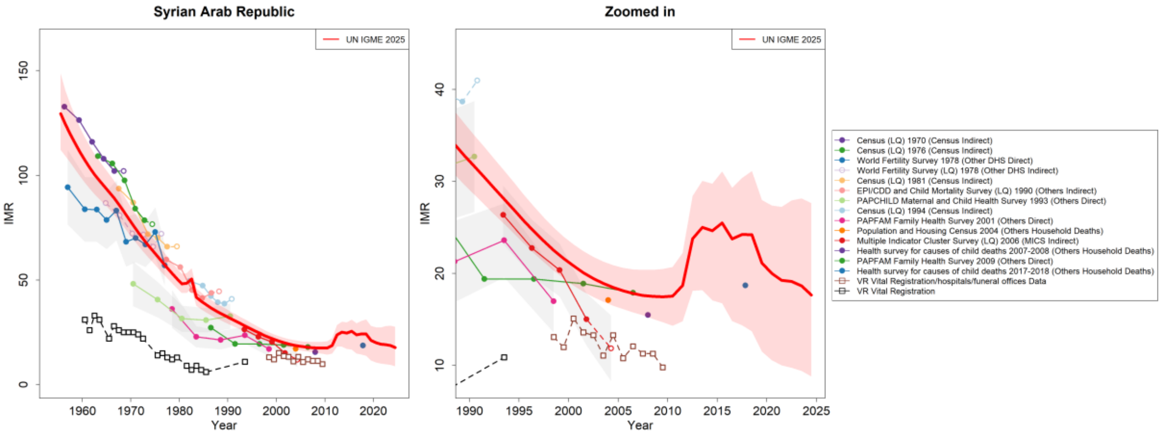


### Neonatal mortality rate

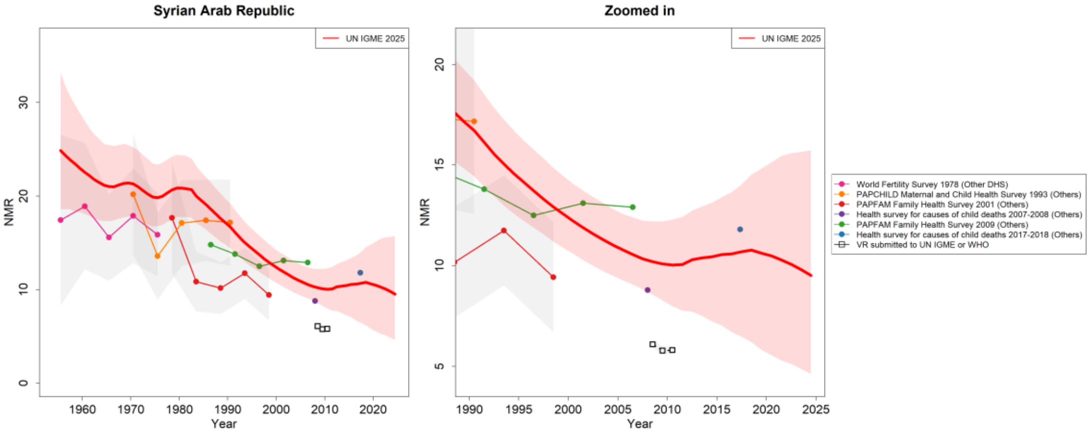

## Tajikistan (TJK)

### Under-five mortality rate

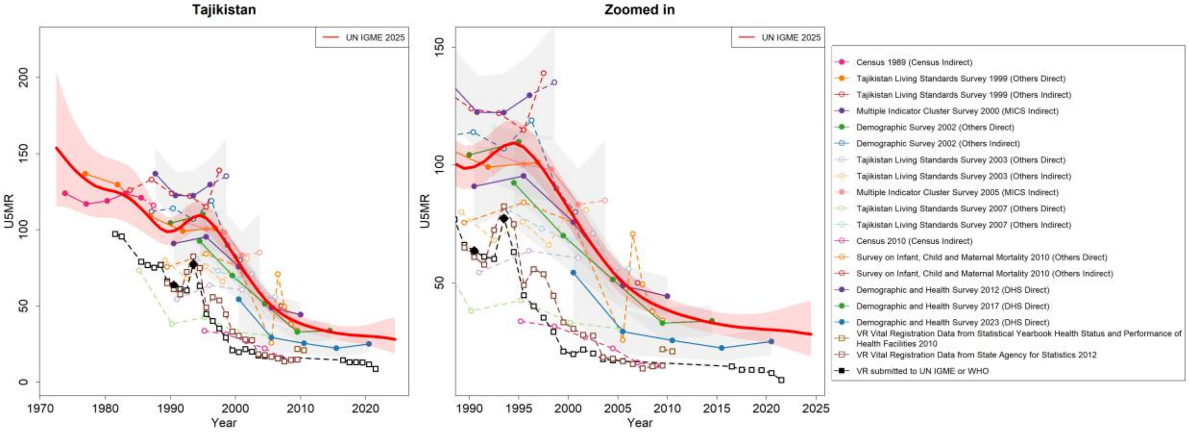


### Infant mortality rate

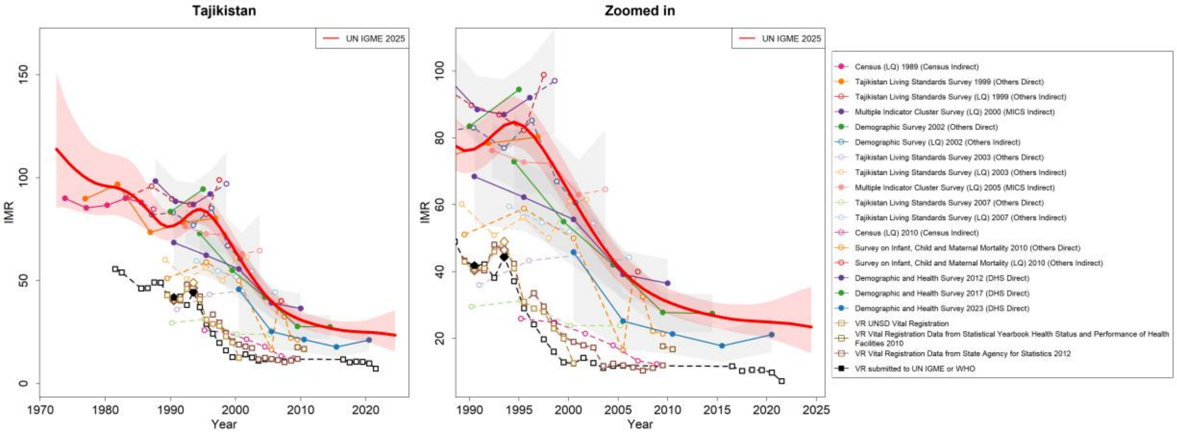


### Neonatal mortality rate

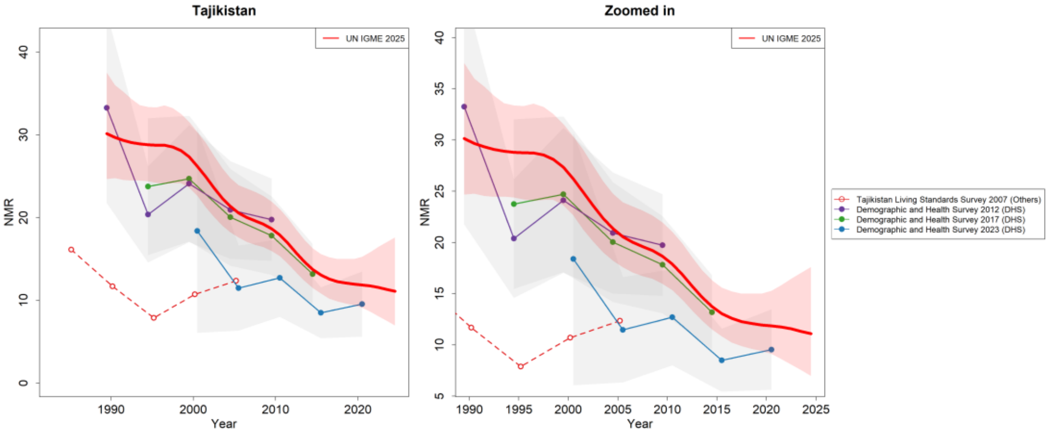

# Thailand (THA)

## Under-five mortality rate

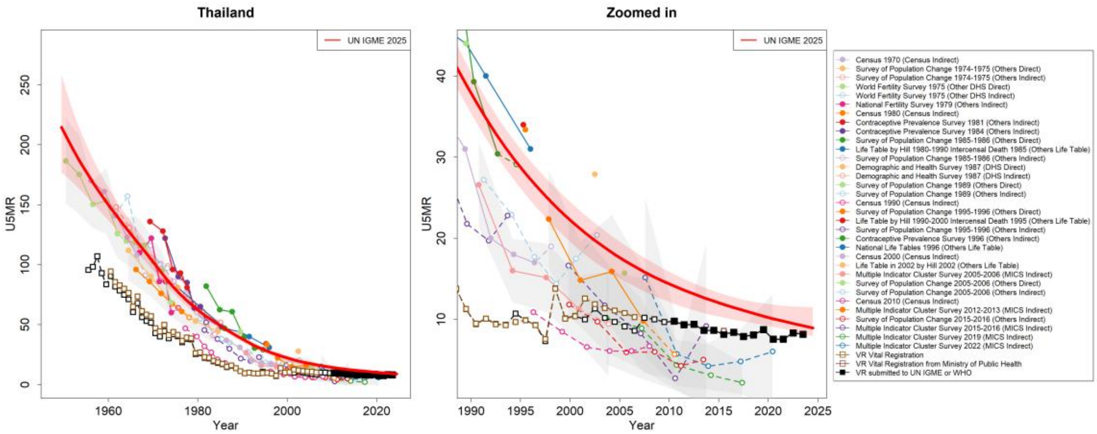


## Infant mortality rate

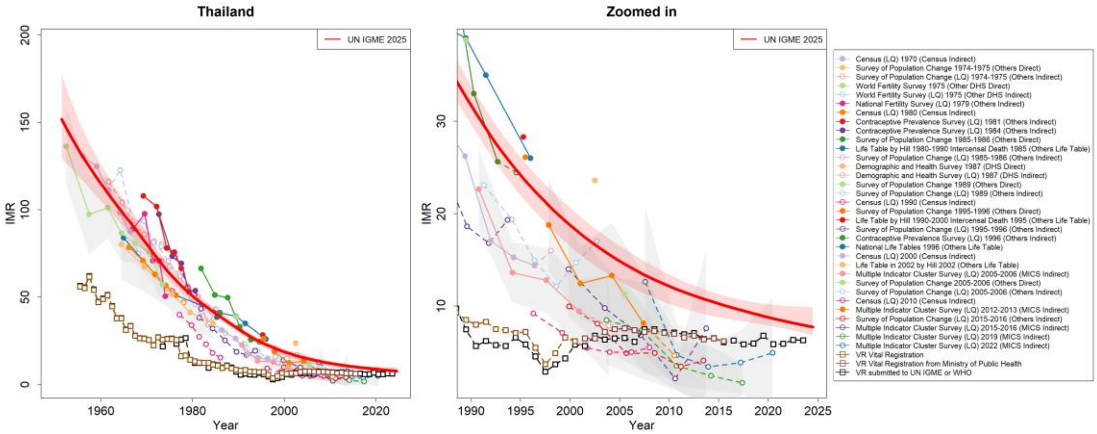


## Neonatal mortality rate

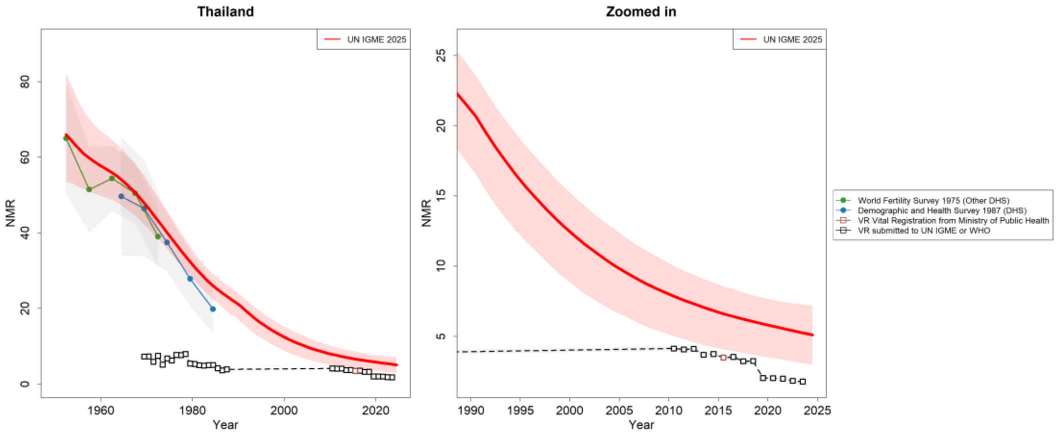

## Timor-Leste (TLS)

### Under-five mortality rate

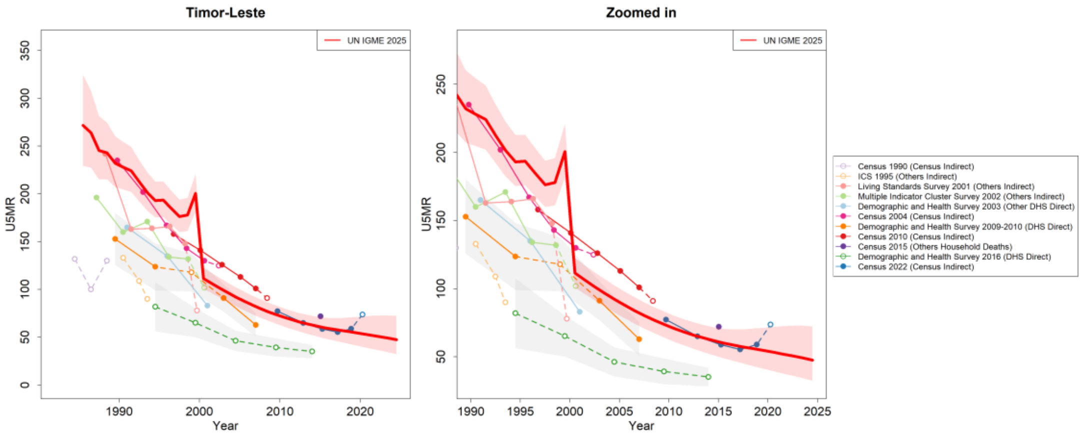


### Infant mortality rate

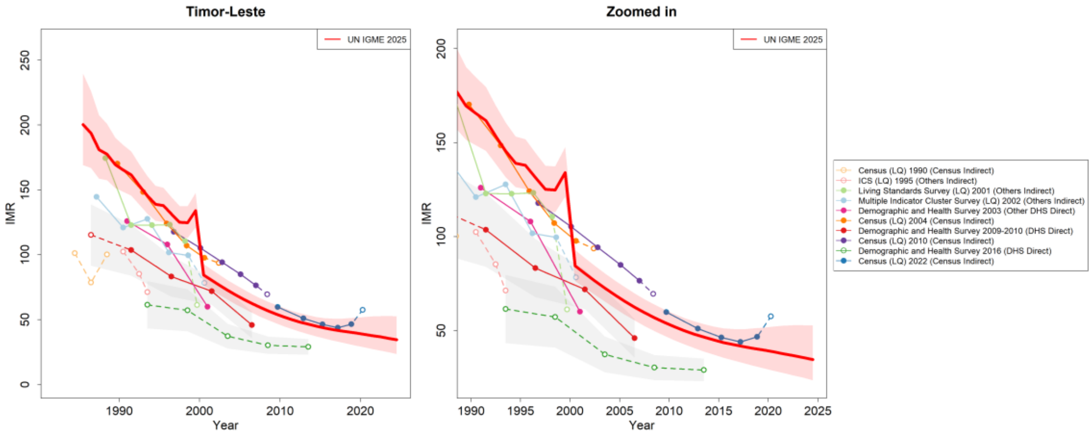


### Neonatal mortality rate

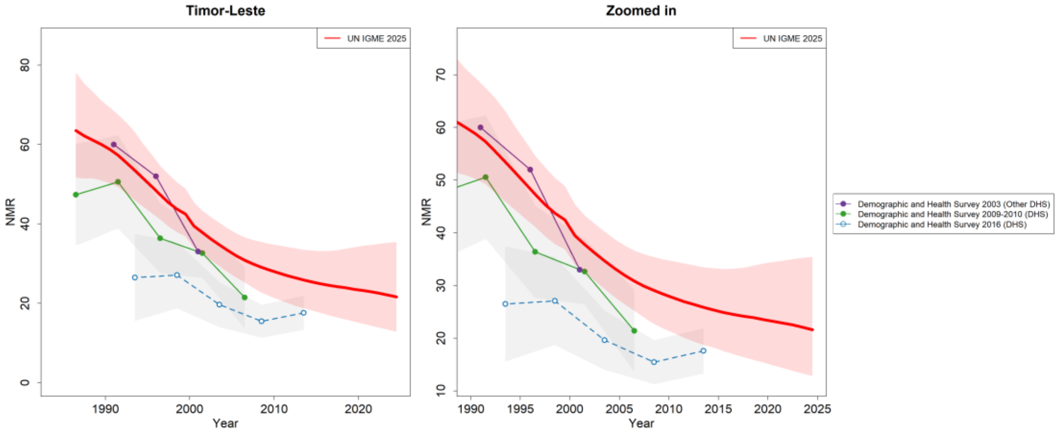

# Togo (TGO)

## Under-five mortality rate

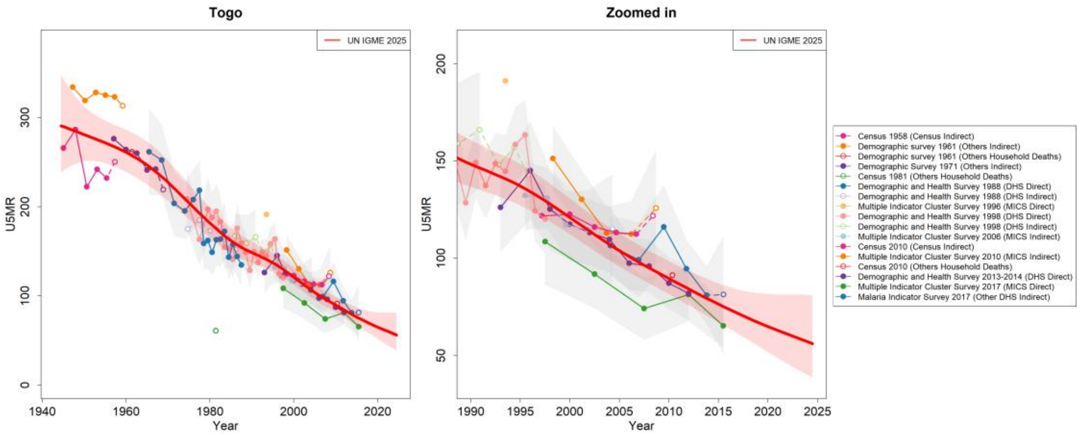


## Infant mortality rate

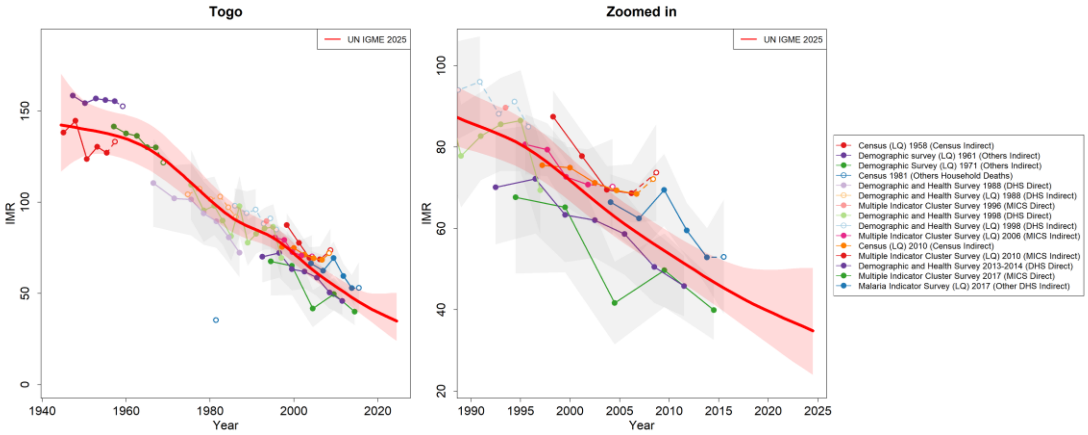


## Neonatal mortality rate

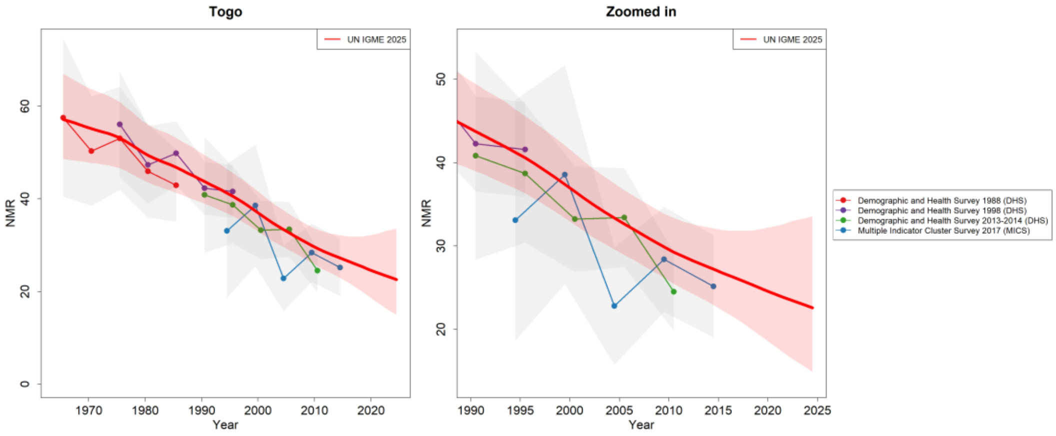

## Tonga (TON)

### Under-five mortality rate

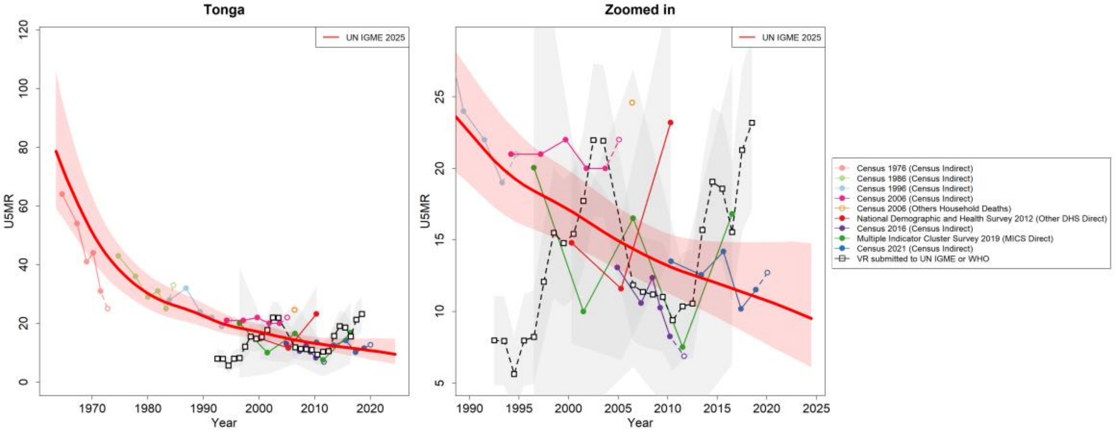


### Infant mortality rate

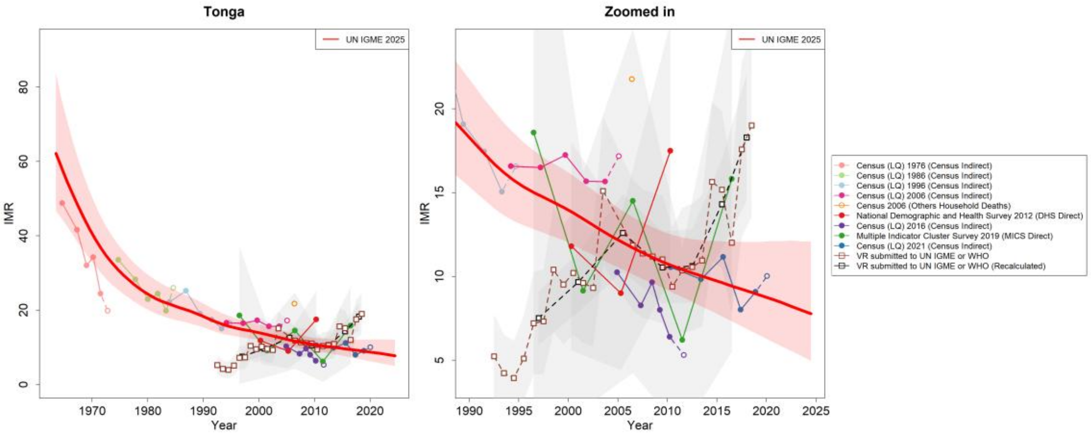


### Neonatal mortality rate

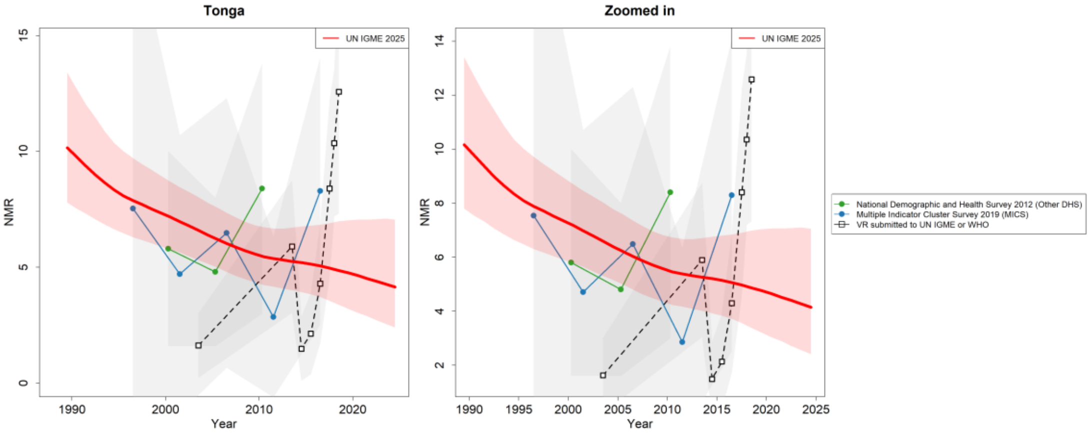

## Trinidad and Tobago (TTO)

### Under-five mortality rate

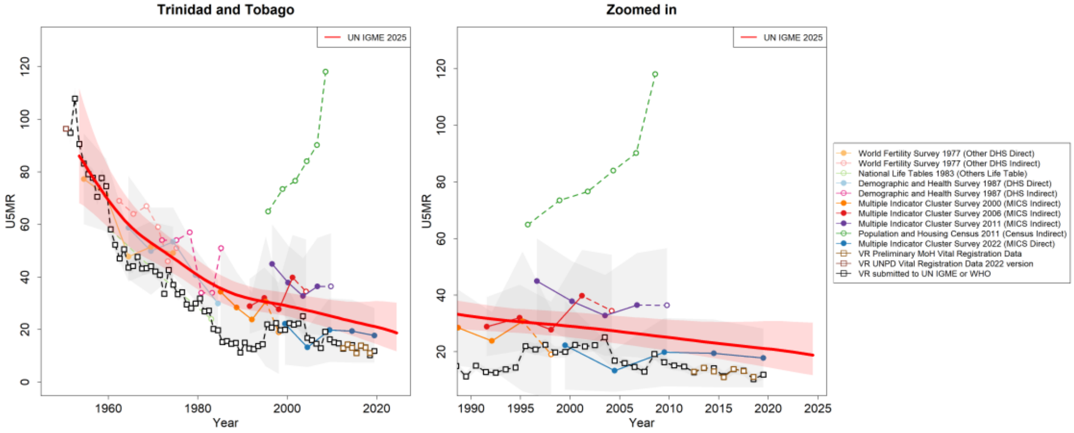


### Infant mortality rate

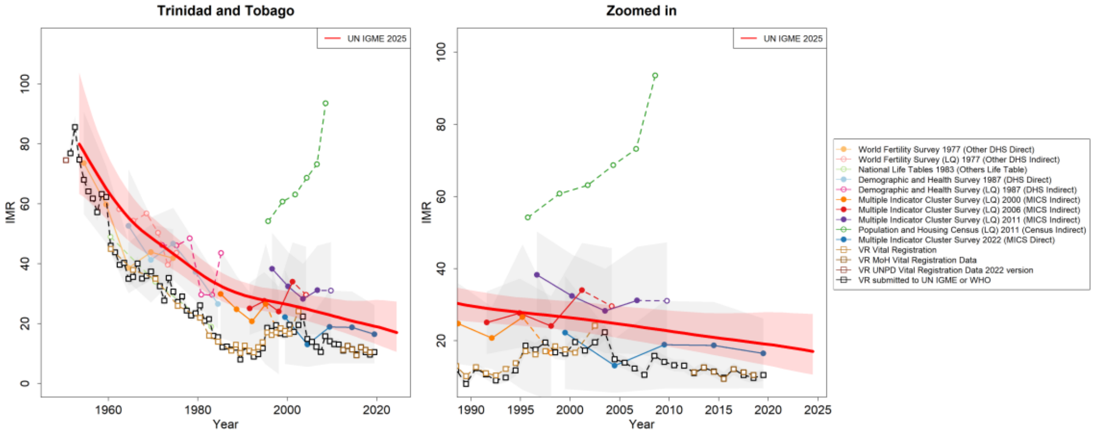


### Neonatal mortality rate

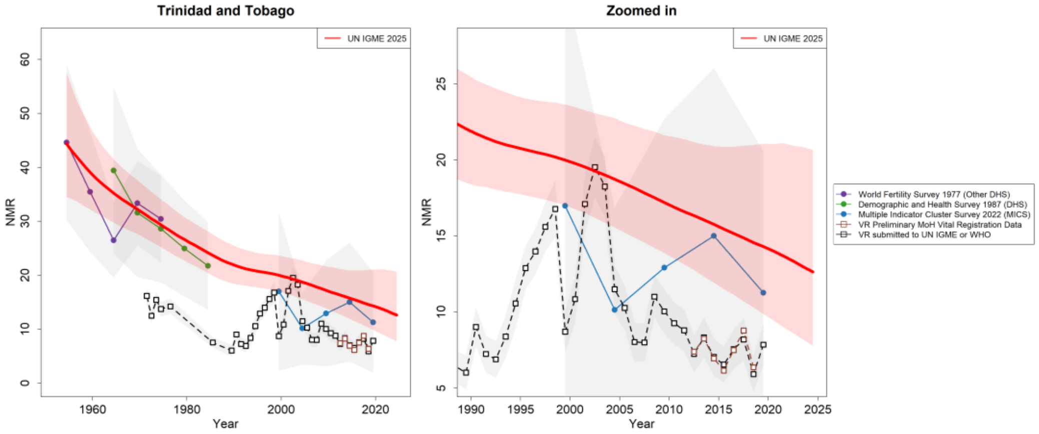

## Tunisia (TUN)

### Under-five mortality rate

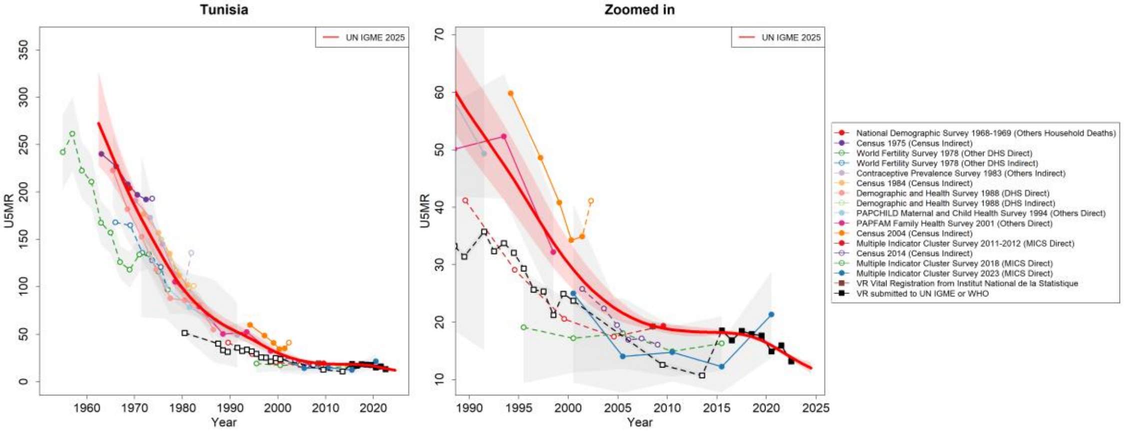


### Infant mortality rate

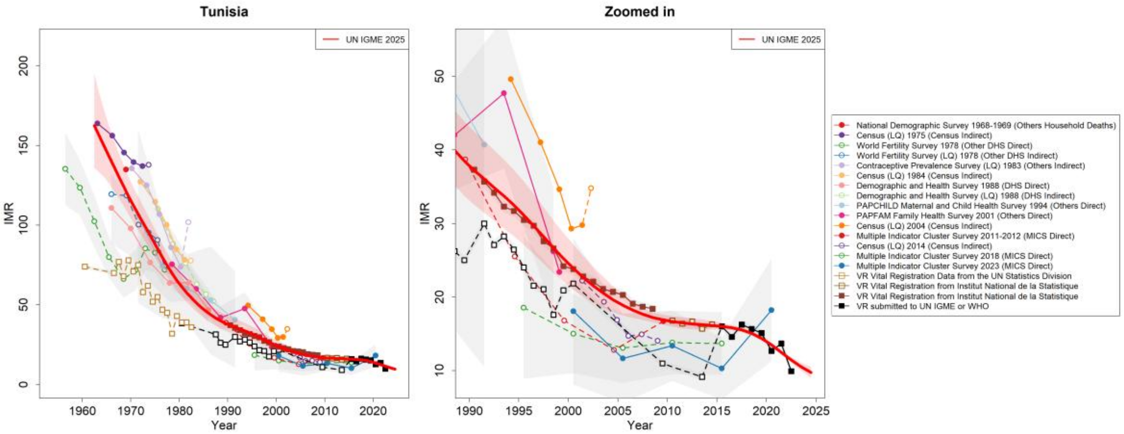


### Neonatal mortality rate

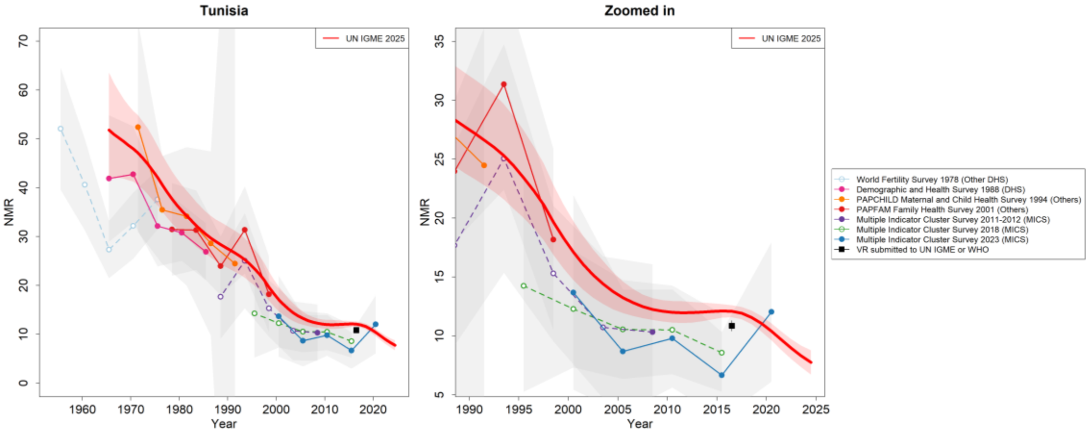

## Turkmenistan (TKM)

### Under-five mortality rate

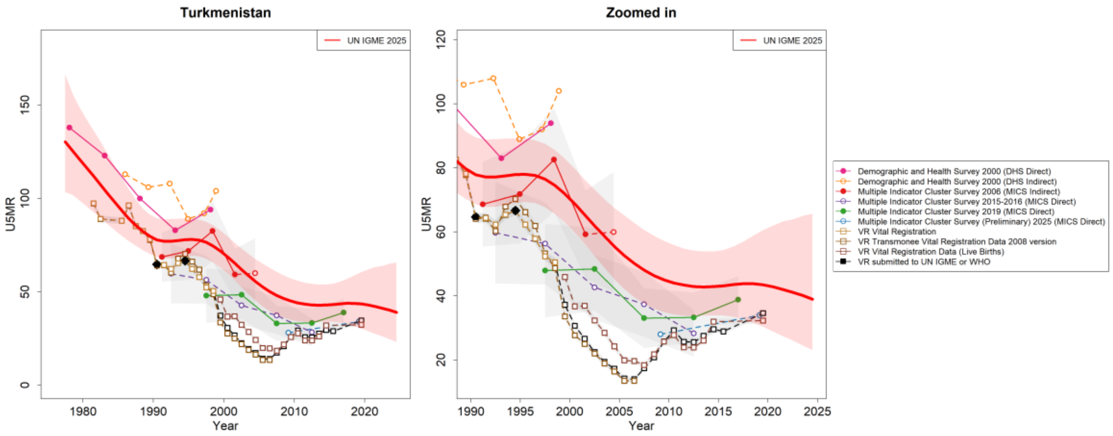


### Infant mortality rate

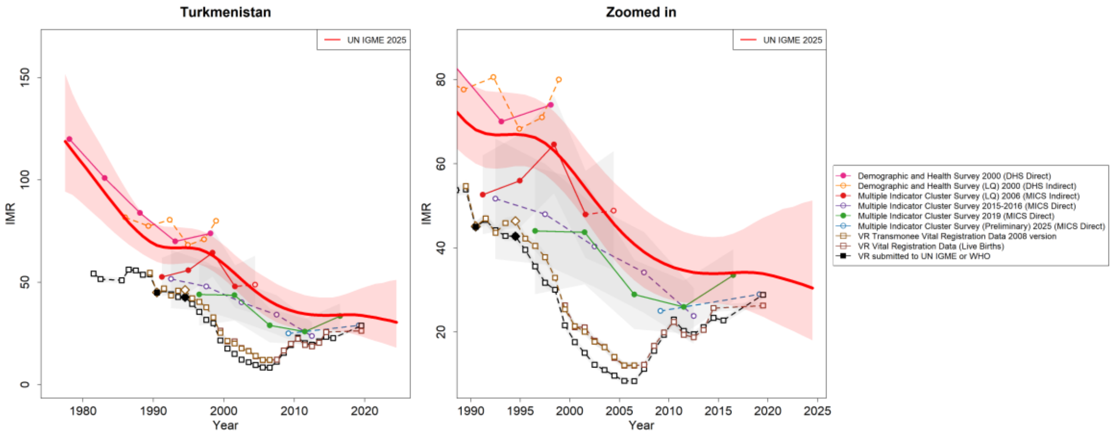


### Neonatal mortality rate

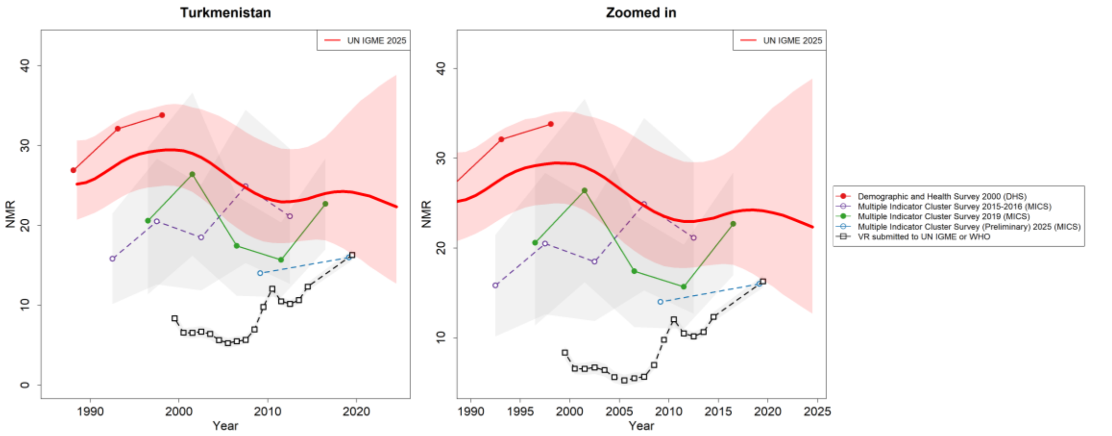

## Turks and Caicos Islands (TCA)

### Under-five mortality rate

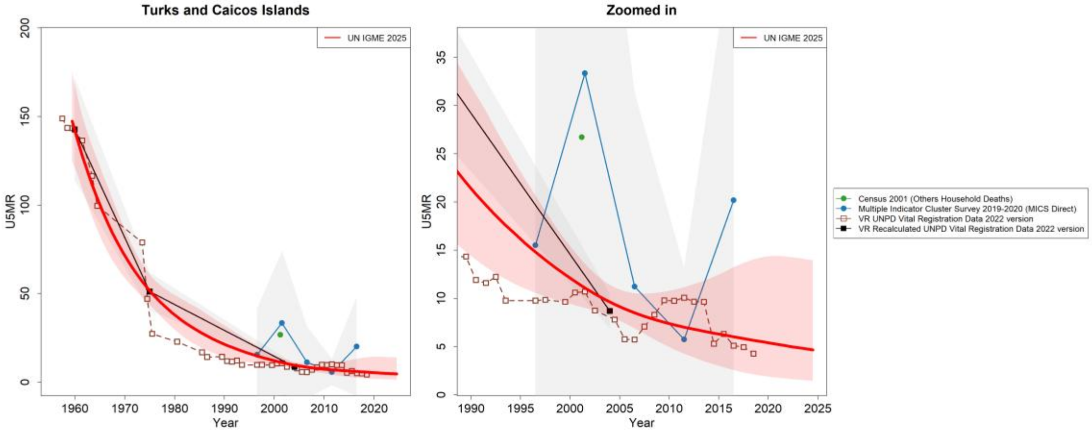


### Infant mortality rate

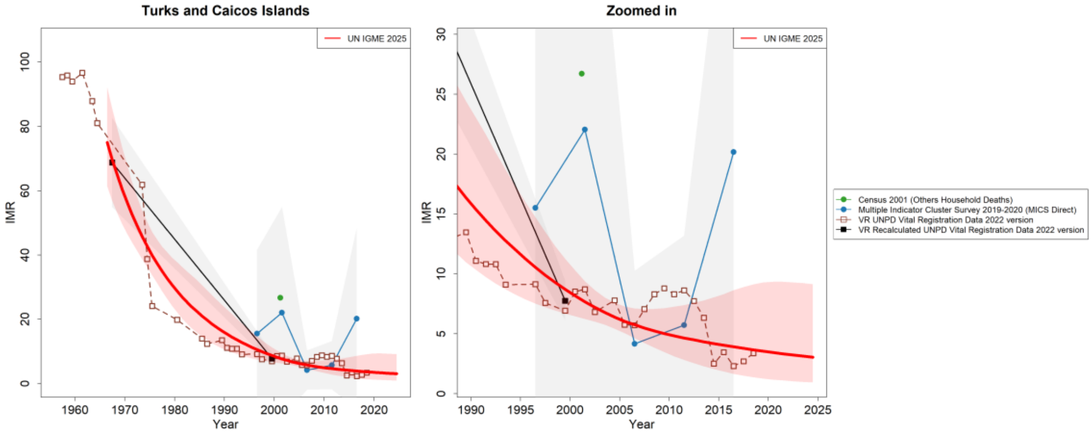


### Neonatal mortality rate

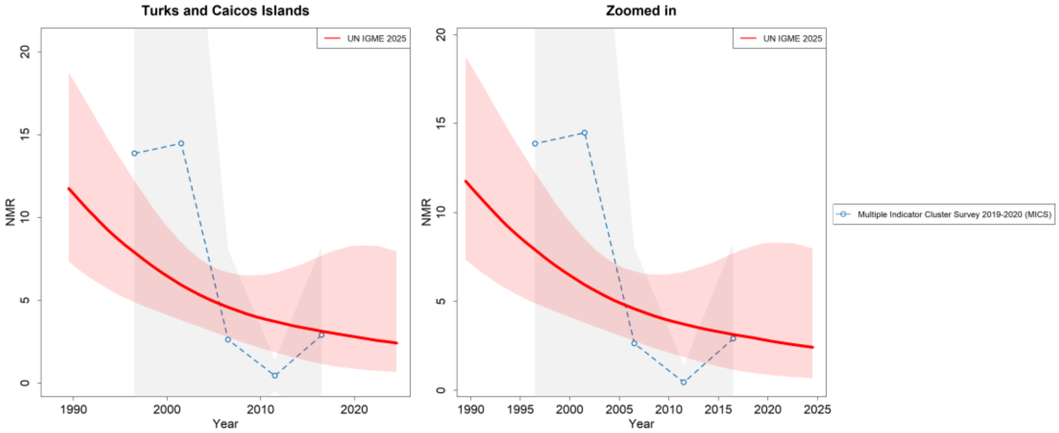

## Tuvalu (TUV)

### Under-five mortality rate

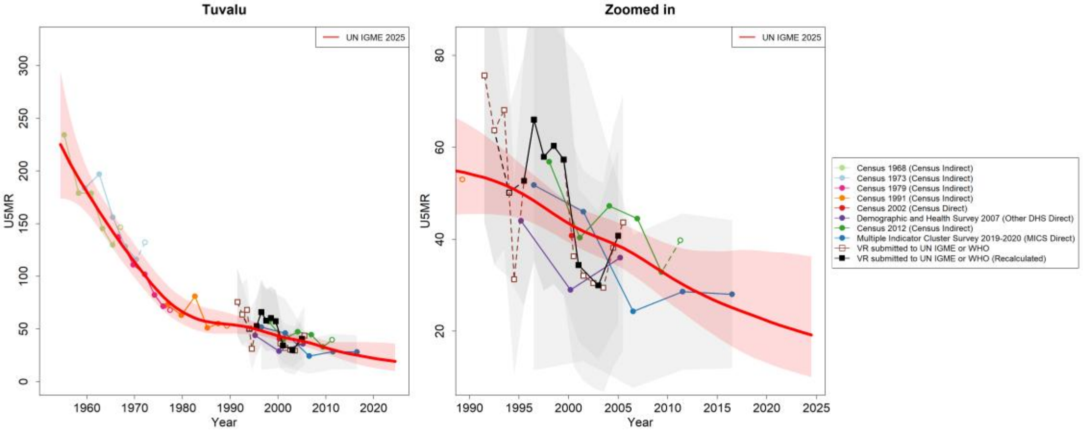


### Infant mortality rate

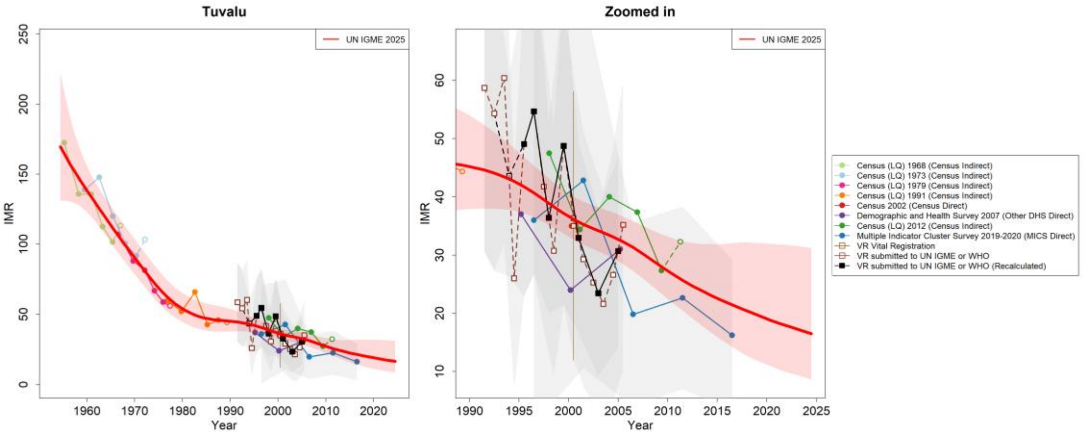


### Neonatal mortality rate

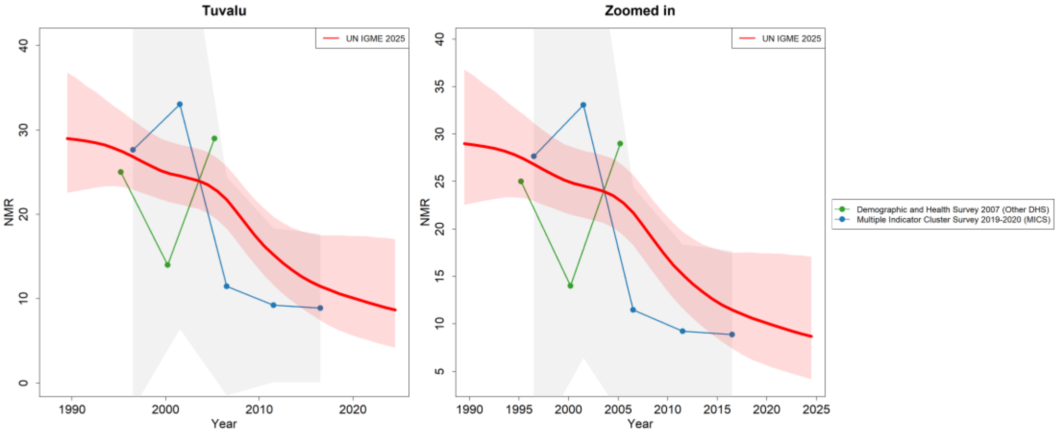

## Türkiye (TUR)

### Under-five mortality rate

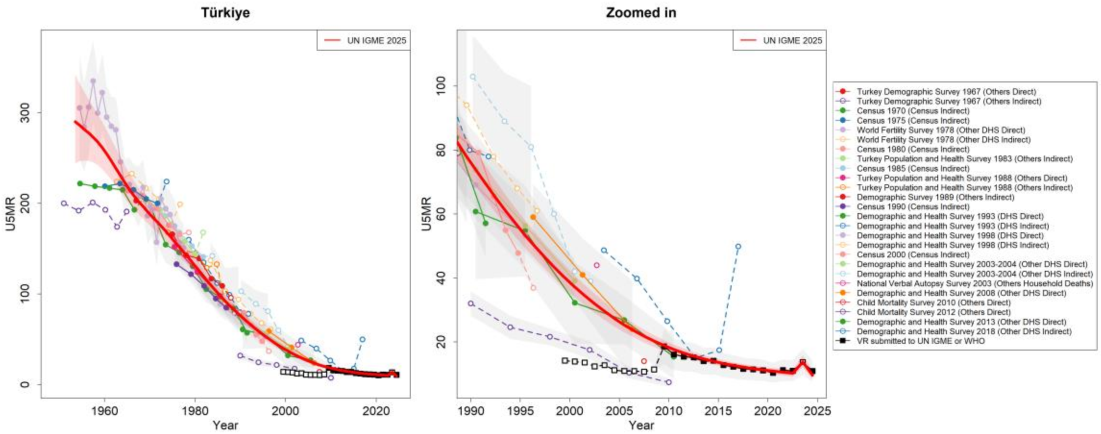


### Infant mortality rate

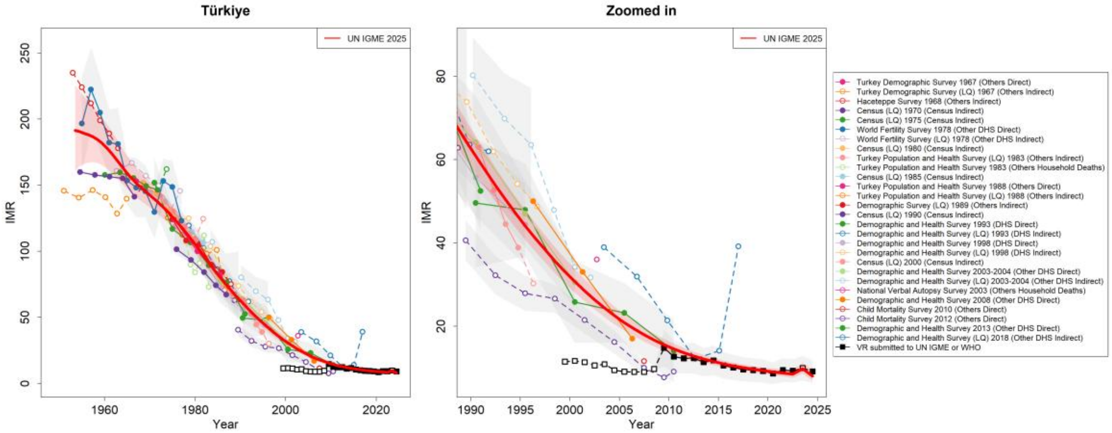


### Neonatal mortality rate

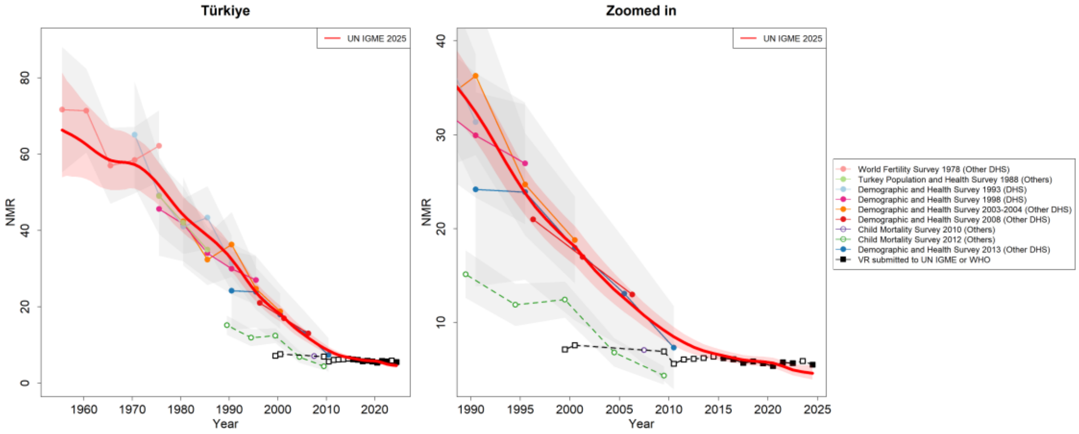

## Uganda (UGA)

### Under-five mortality rate

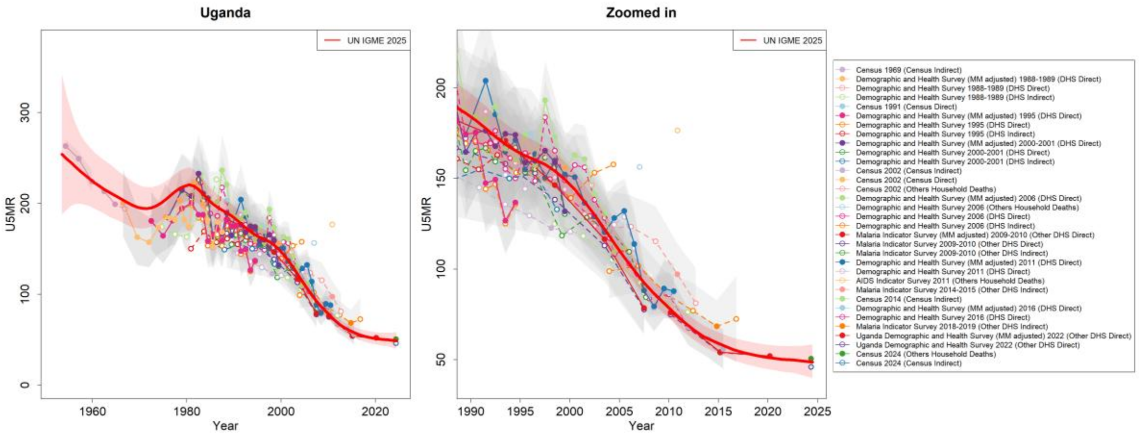


### Infant mortality rate

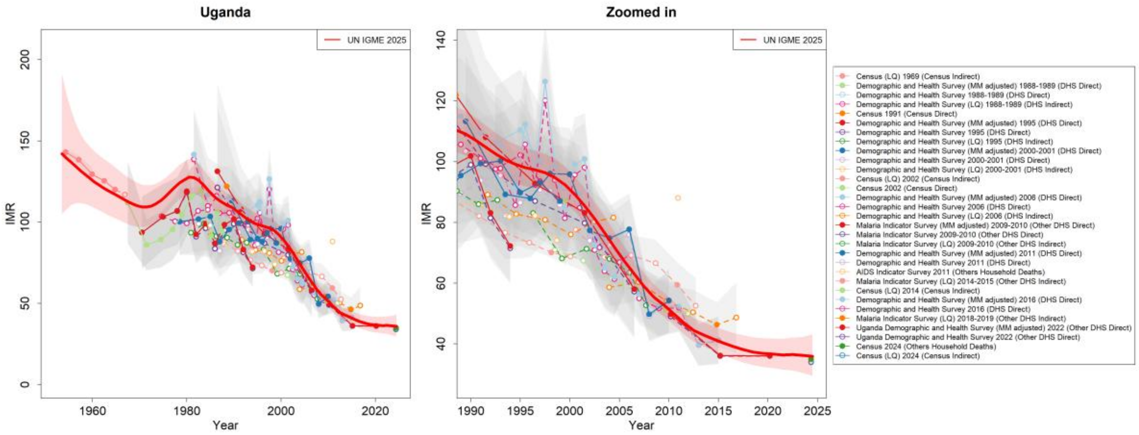


### Neonatal mortality rate

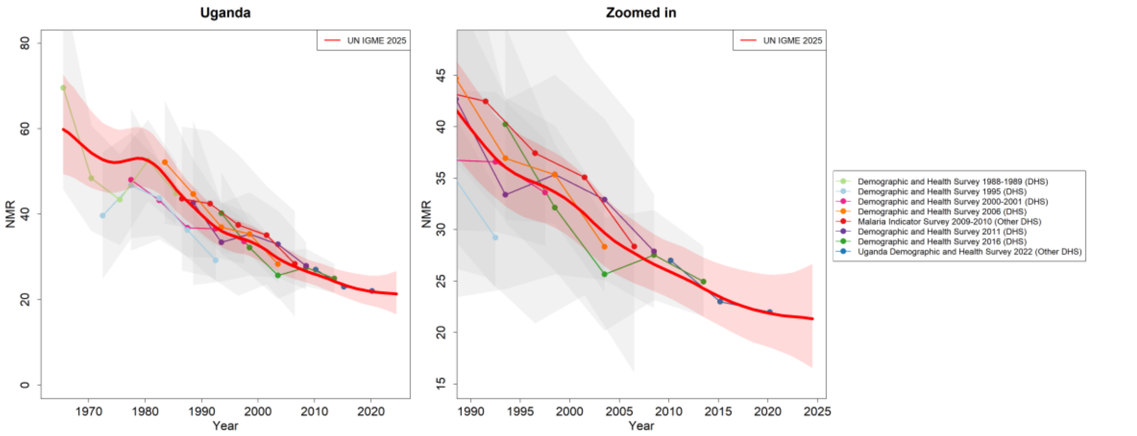

## Ukraine (UKR)

### Under-five mortality rate

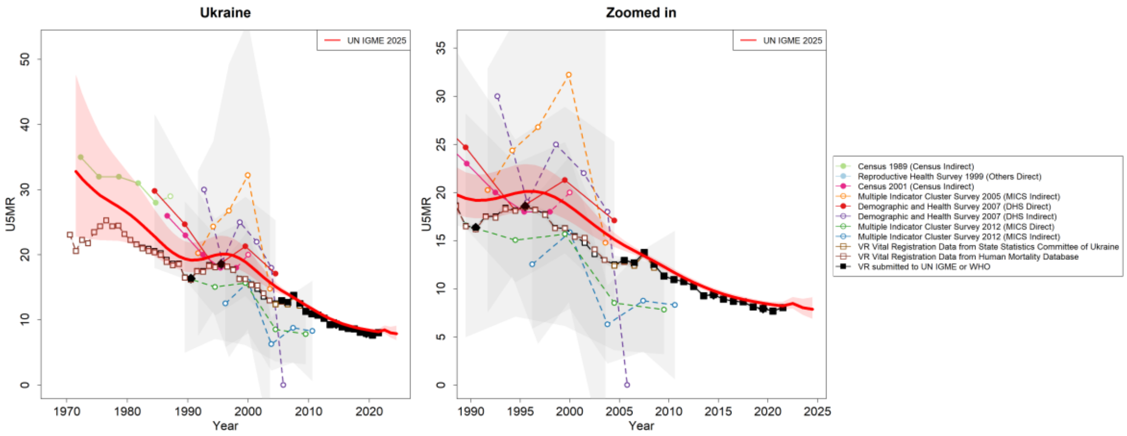


### Infant mortality rate

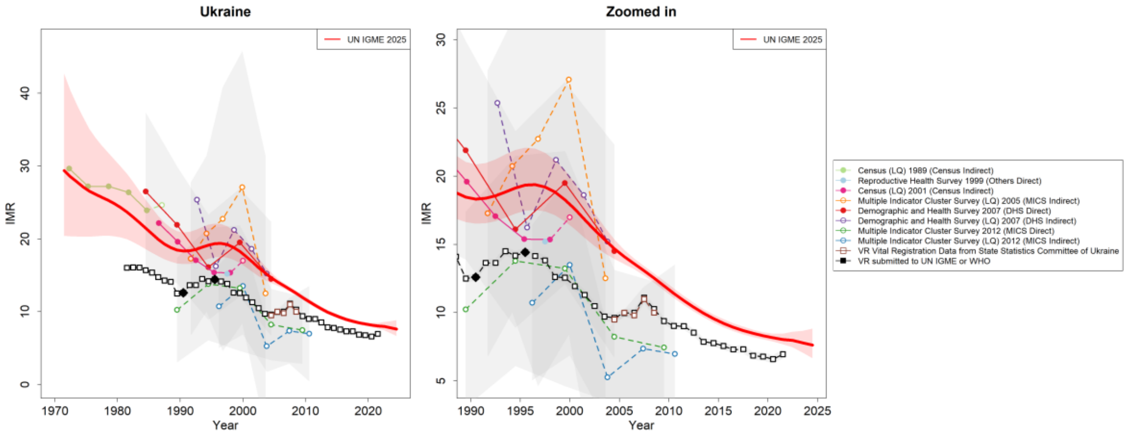


### Neonatal mortality rate

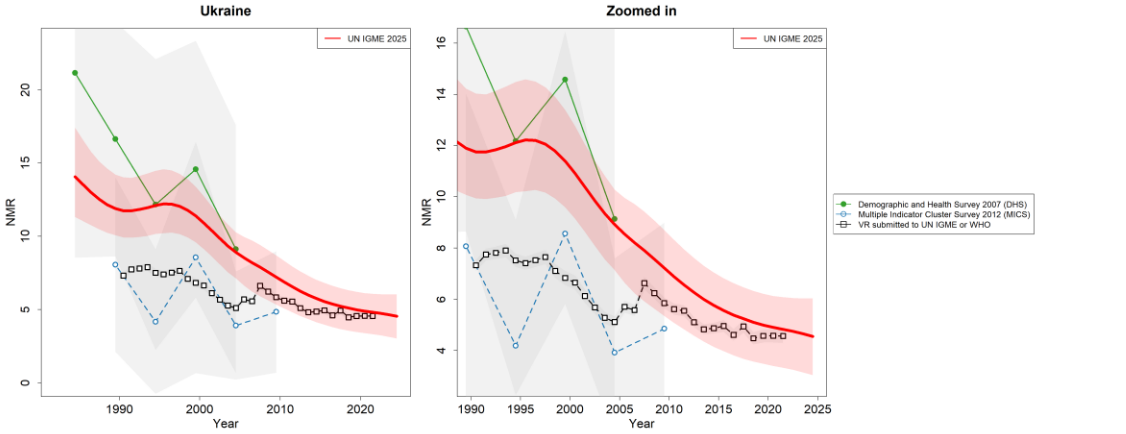

## United Arab Emirates (ARE)

### Under-five mortality rate

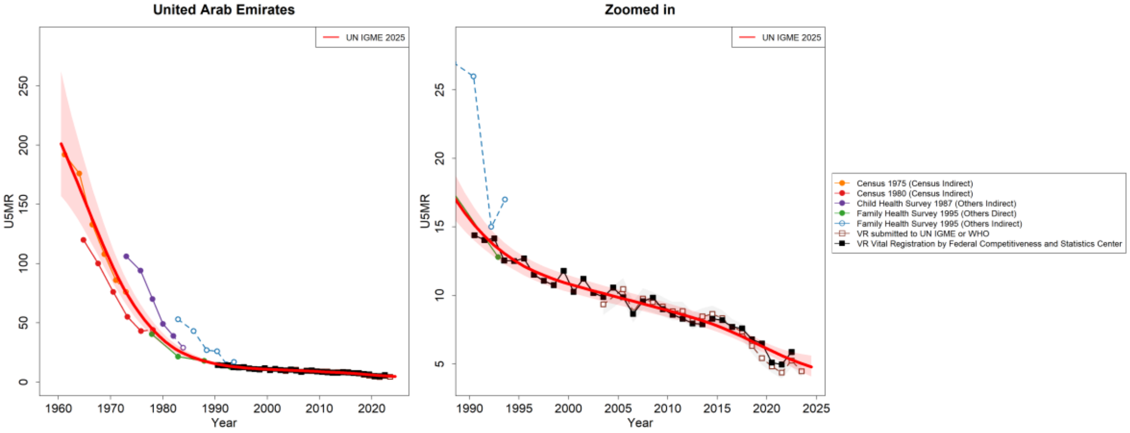


### Infant mortality rate

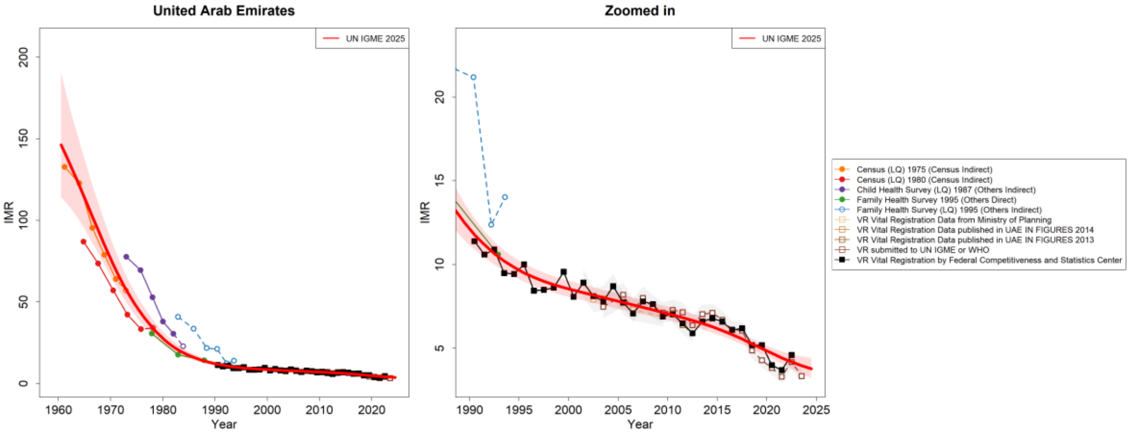


### Neonatal mortality rate

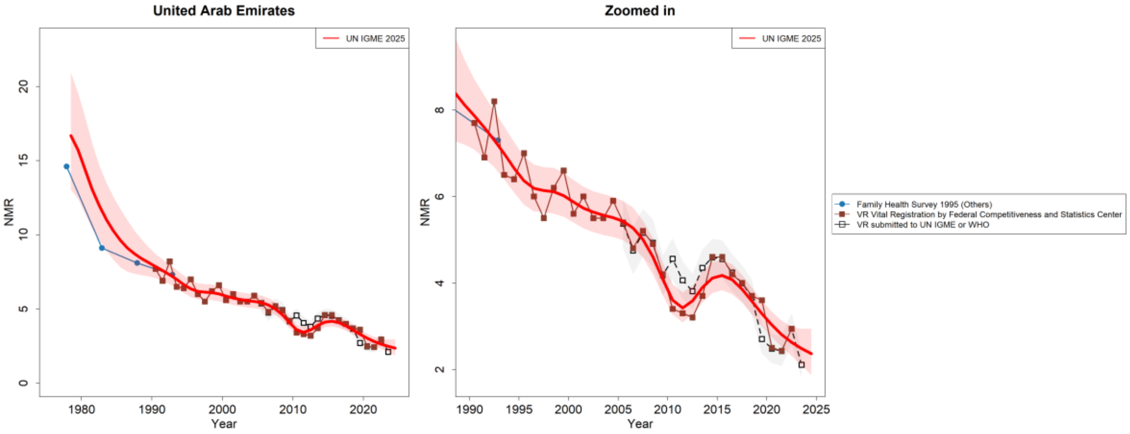

## United Kingdom (GBR)

### Under-five mortality rate

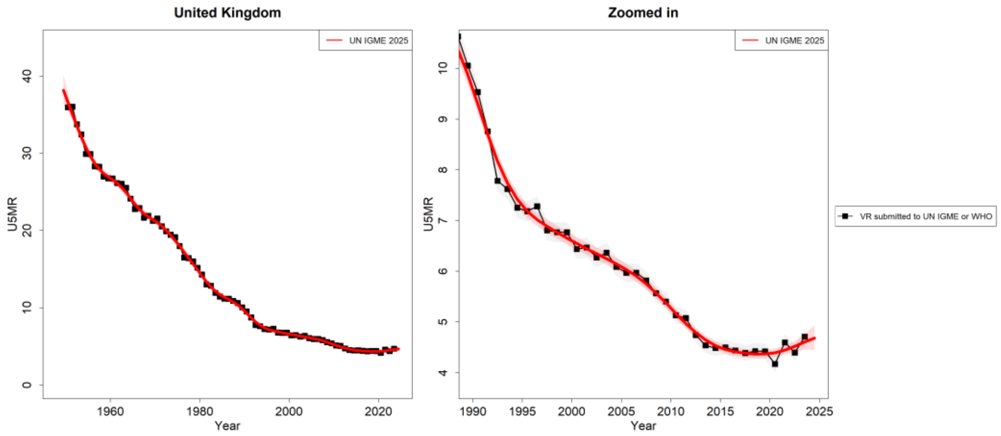


### Infant mortality rate

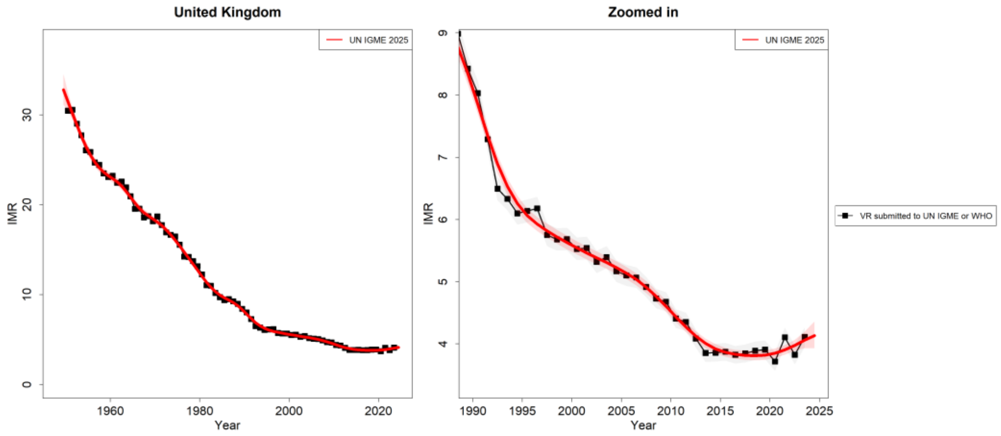


### Neonatal mortality rate

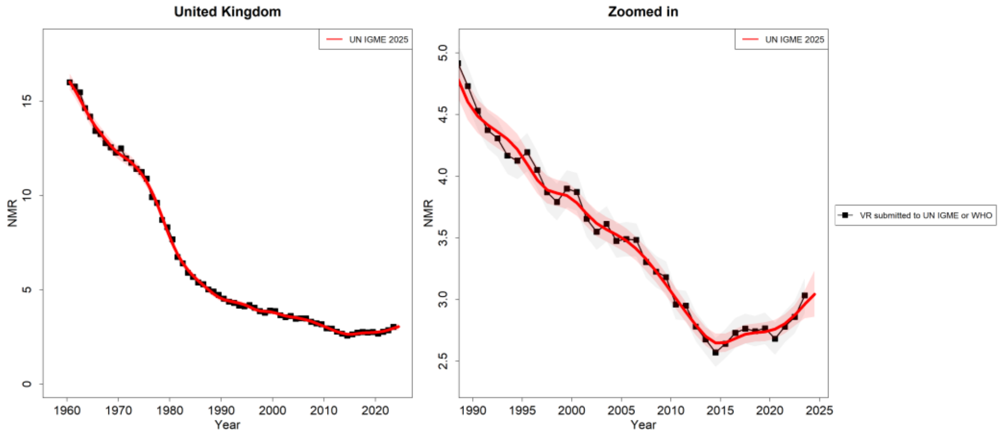

## United Republic of Tanzania (TZA)

### Under-five mortality rate

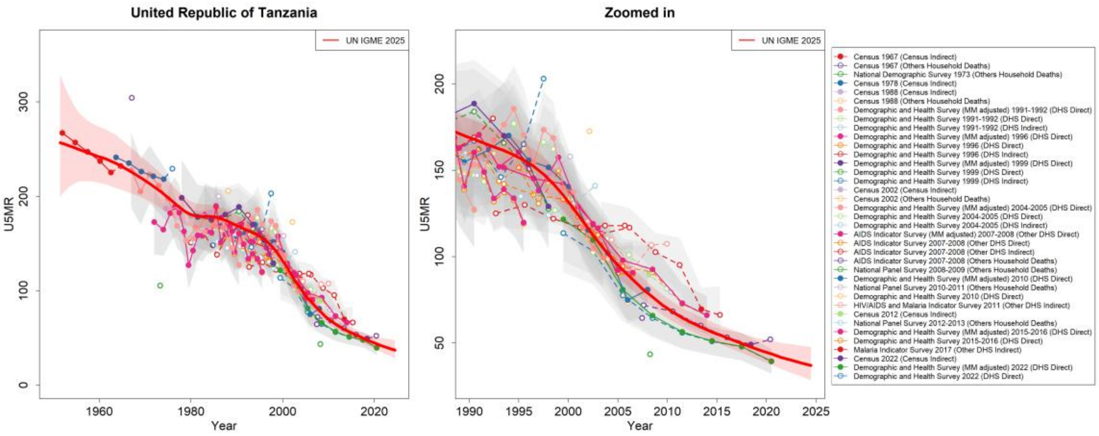


### Infant mortality rate

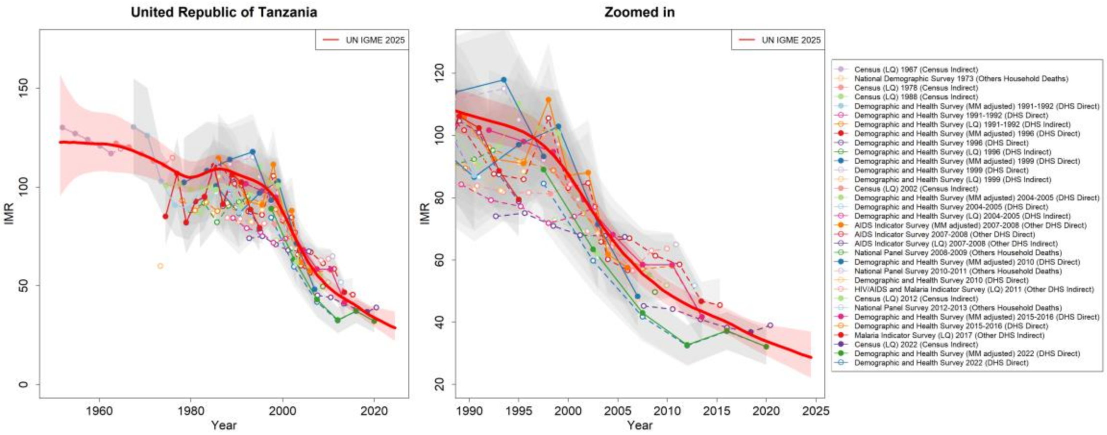


### Neonatal mortality rate

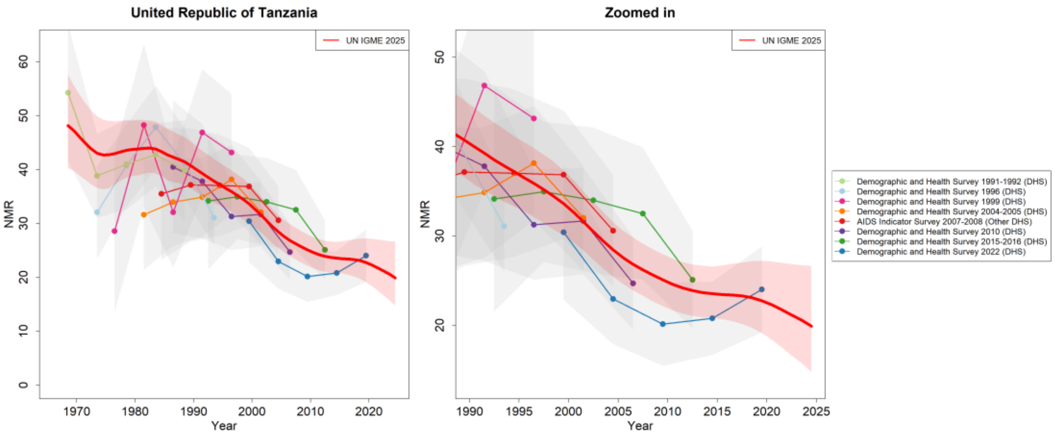

## United States (USA)

### Under-five mortality rate

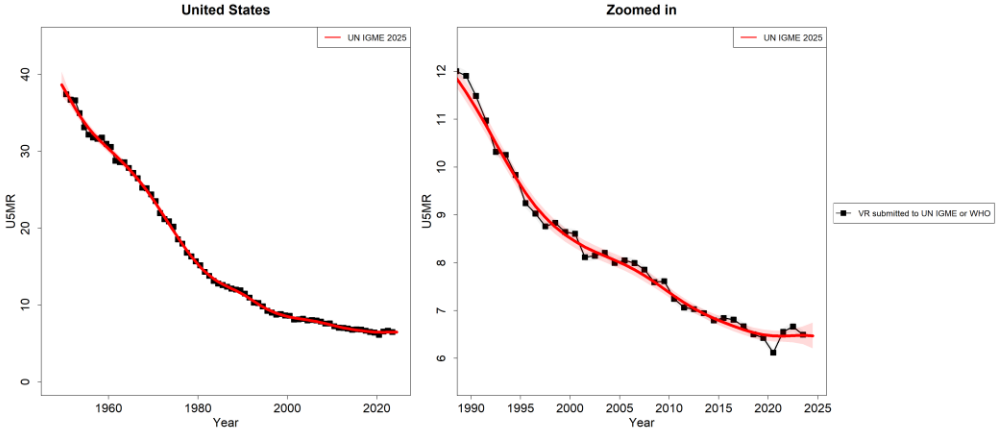


### Infant mortality rate

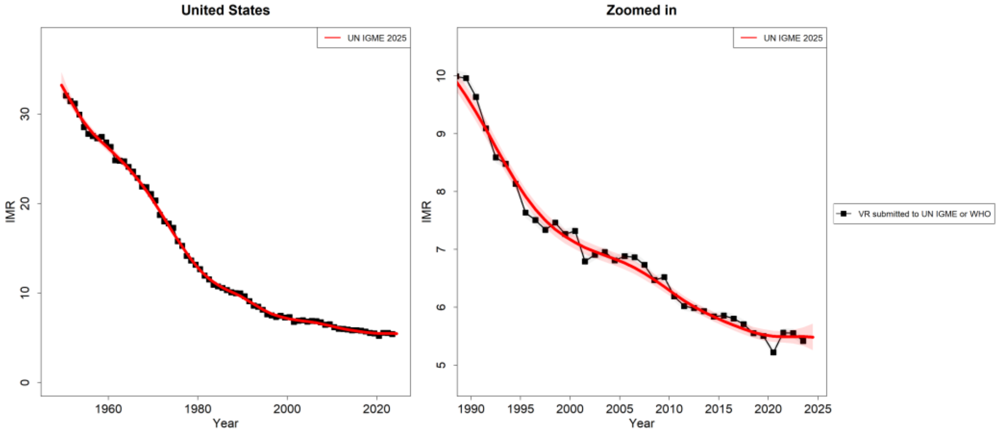


### Neonatal mortality rate

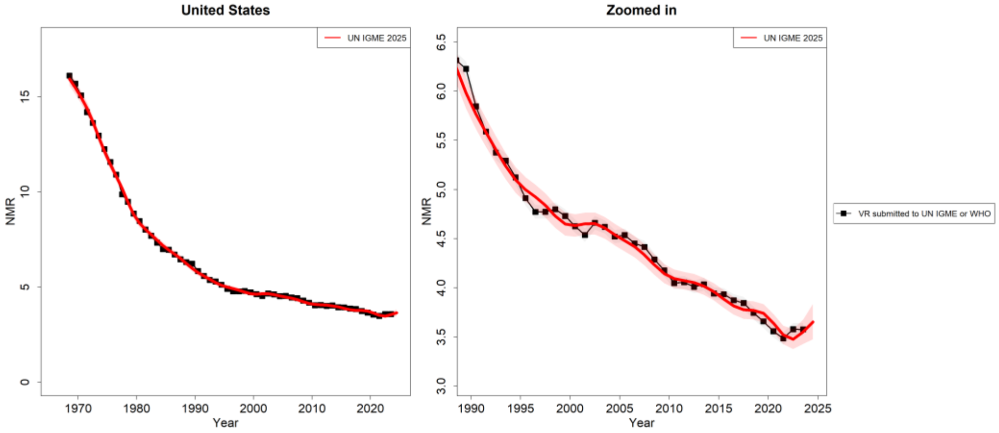

## Uruguay (URY)

### Under-five mortality rate

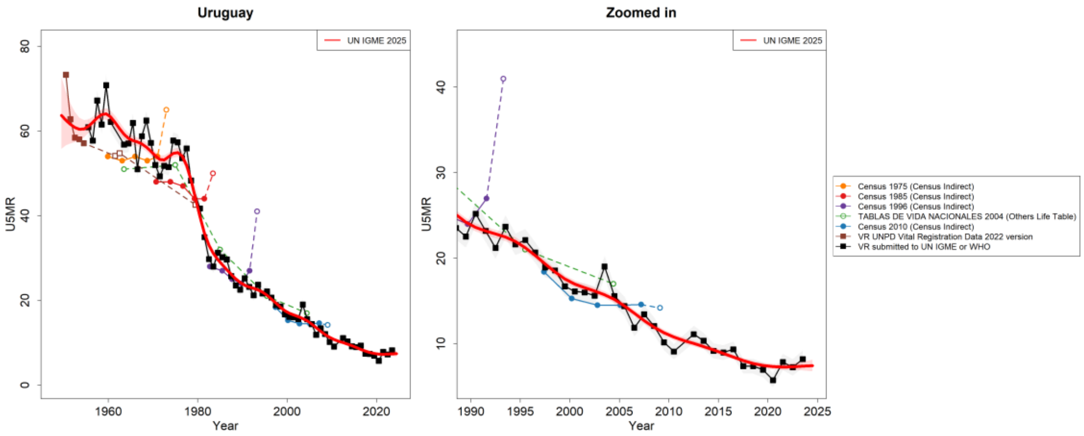


### Infant mortality rate

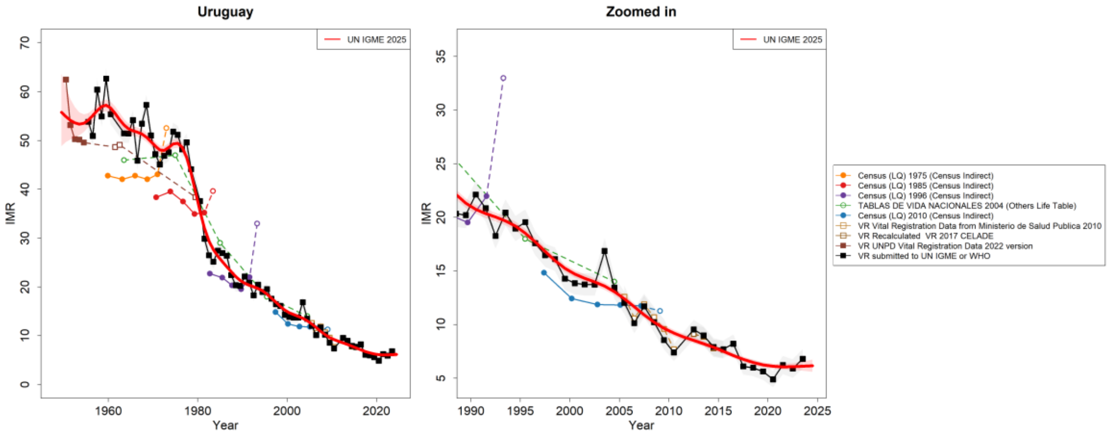


### Neonatal mortality rate

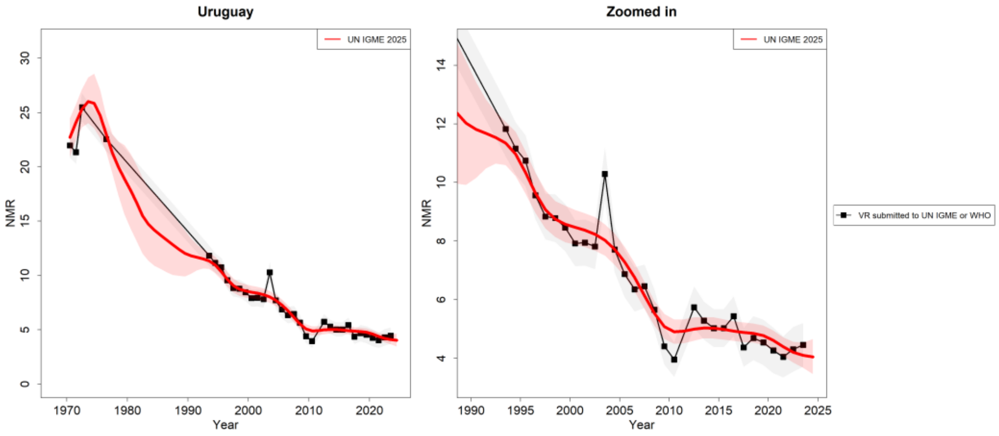

## Uzbekistan (UZB)

### Under-five mortality rate

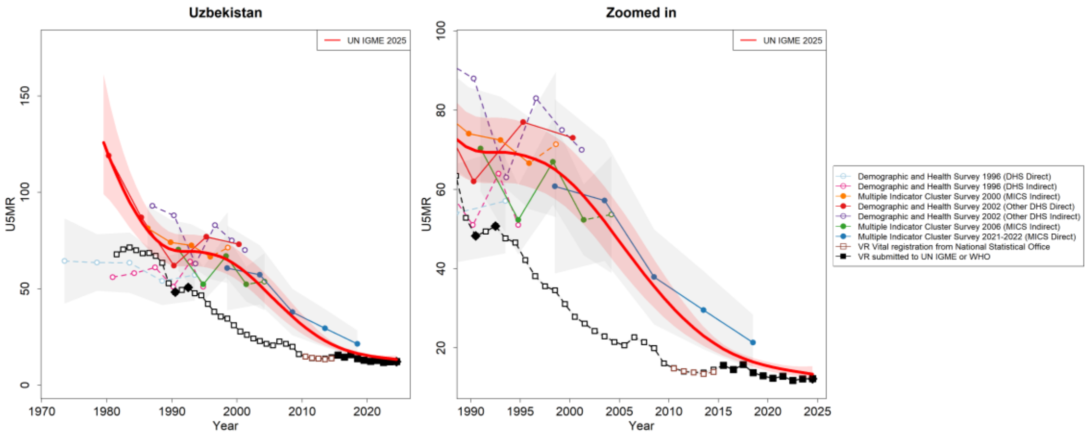


### Infant mortality rate

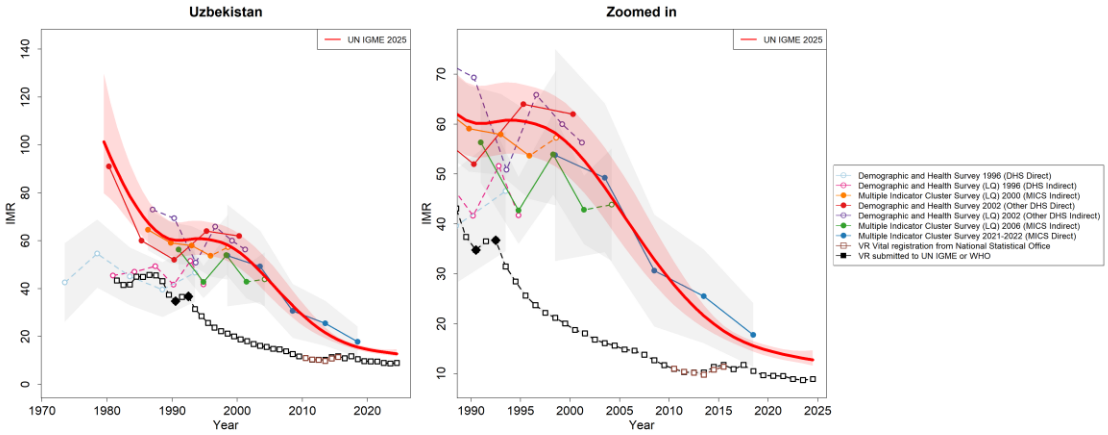


### Neonatal mortality rate

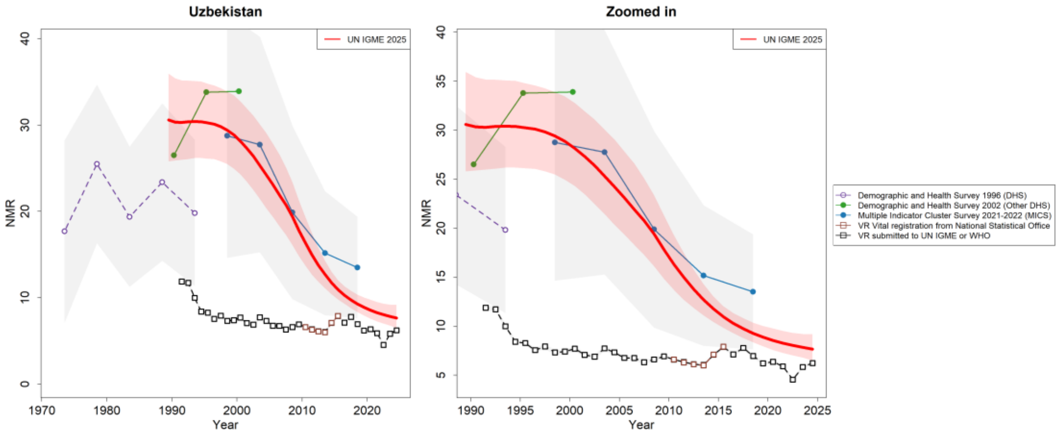

## Vanuatu (VUT)

### Under-five mortality rate

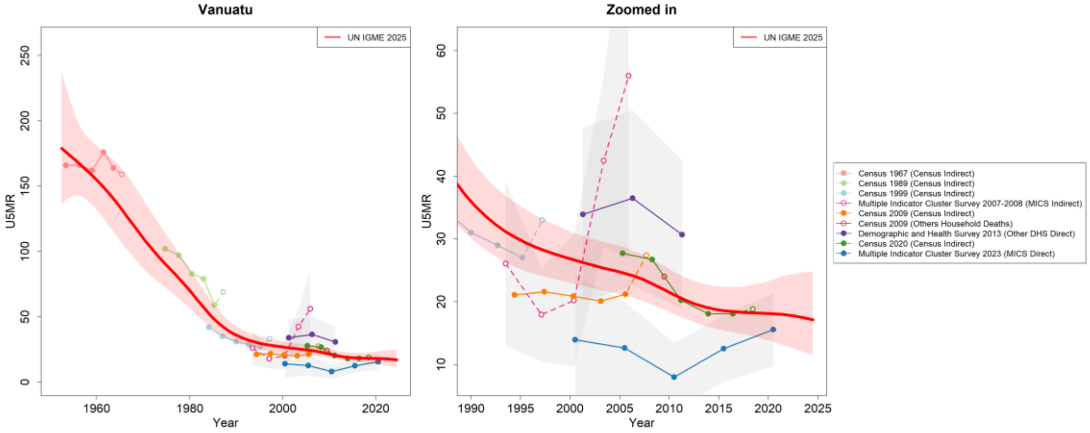


### Infant mortality rate

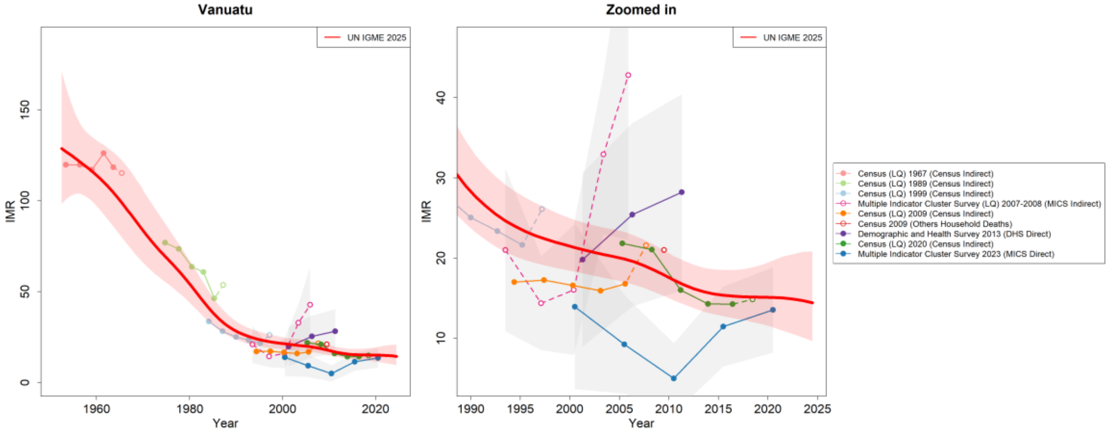


### Neonatal mortality rate

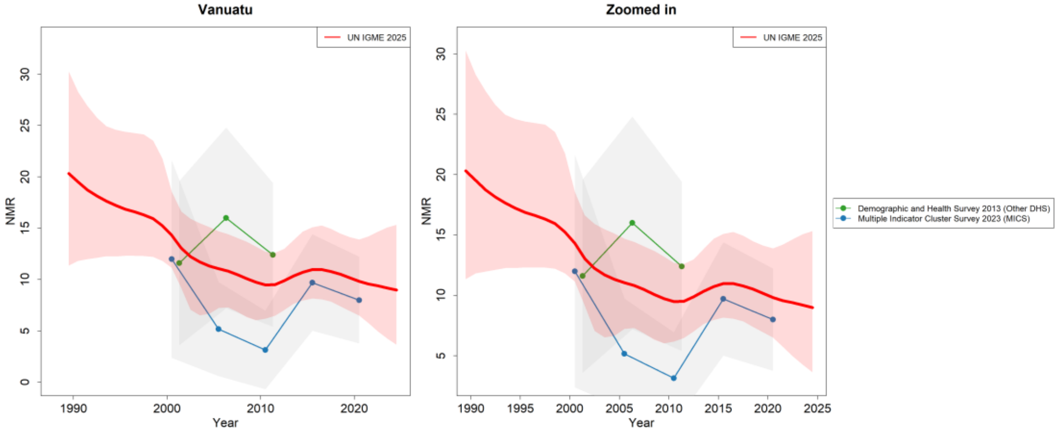

## Venezuela (Bolivarian Republic of) (VEN)

### Under-five mortality rate

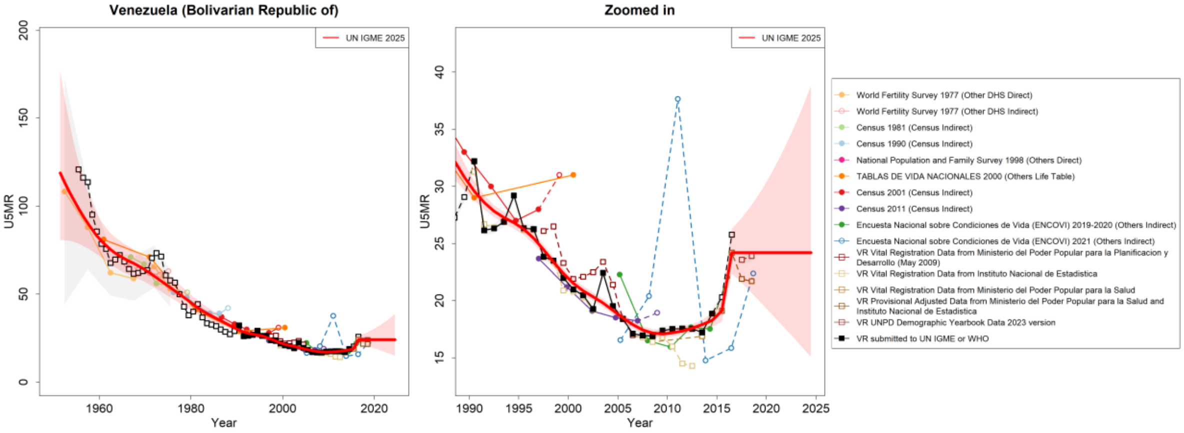


### Infant mortality rate

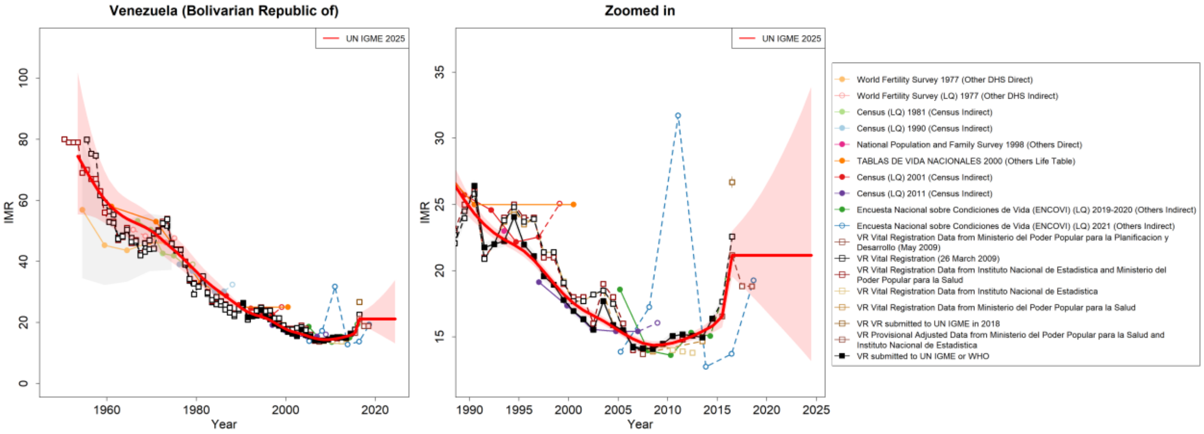


### Neonatal mortality rate

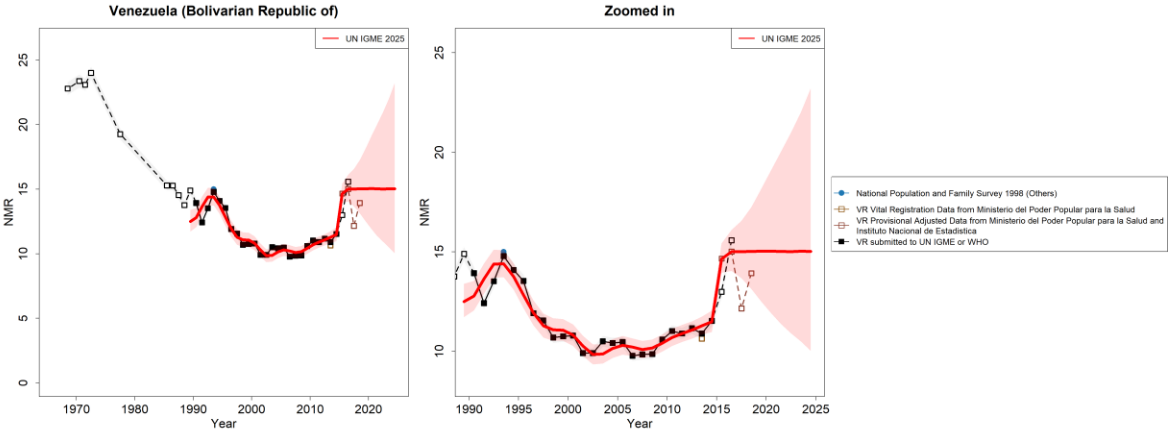

## Viet Nam (VNM)

### Under-five mortality rate

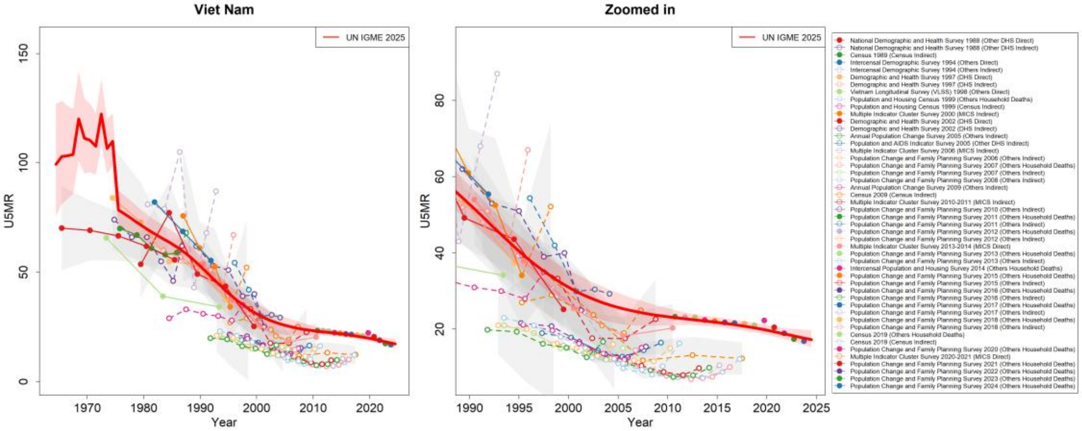


### Infant mortality rate

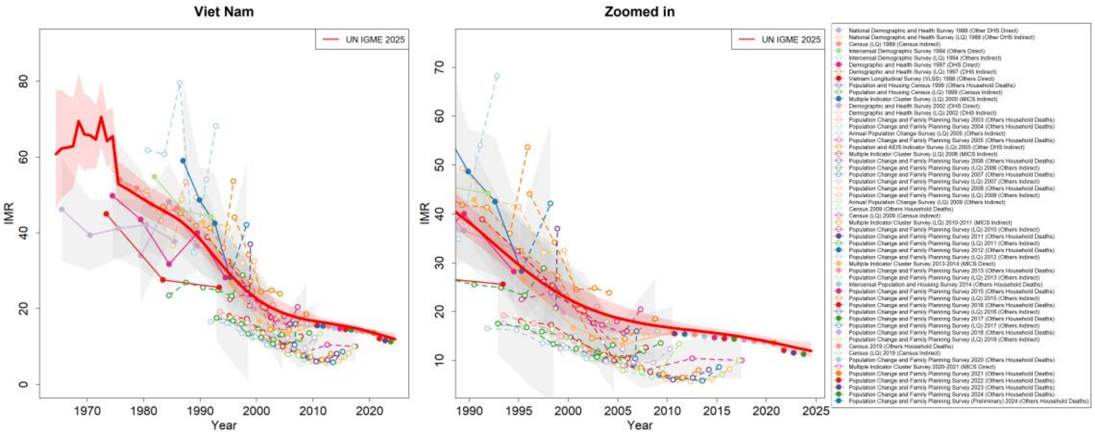


### Neonatal mortality rate

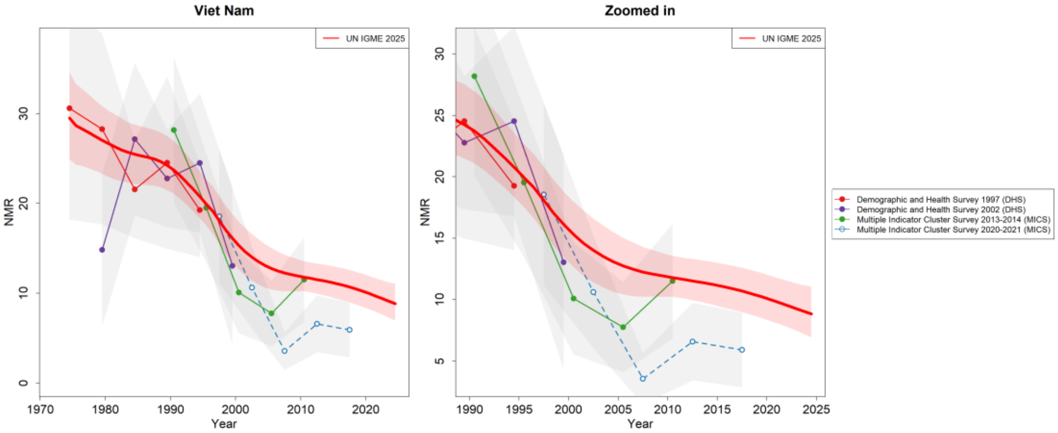

## Yemen (YEM)

### Under-five mortality rate

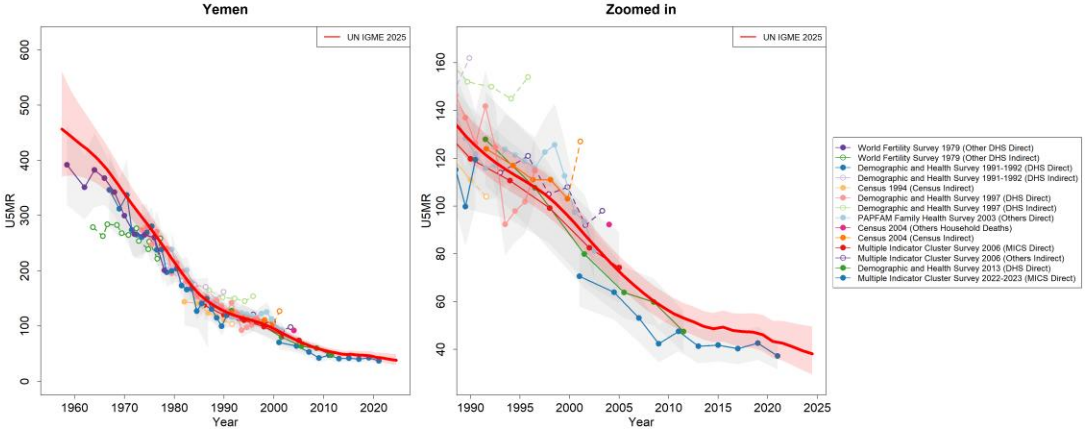


### Infant mortality rate

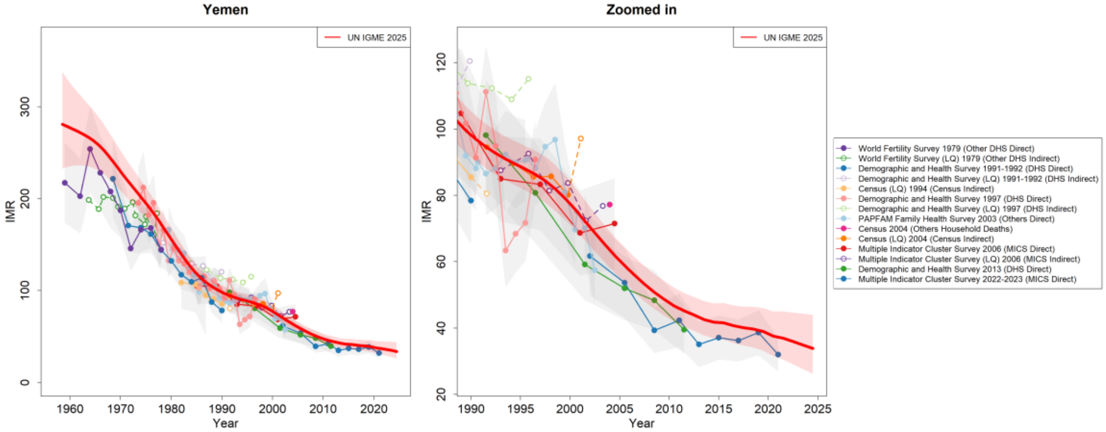


### Neonatal mortality rate

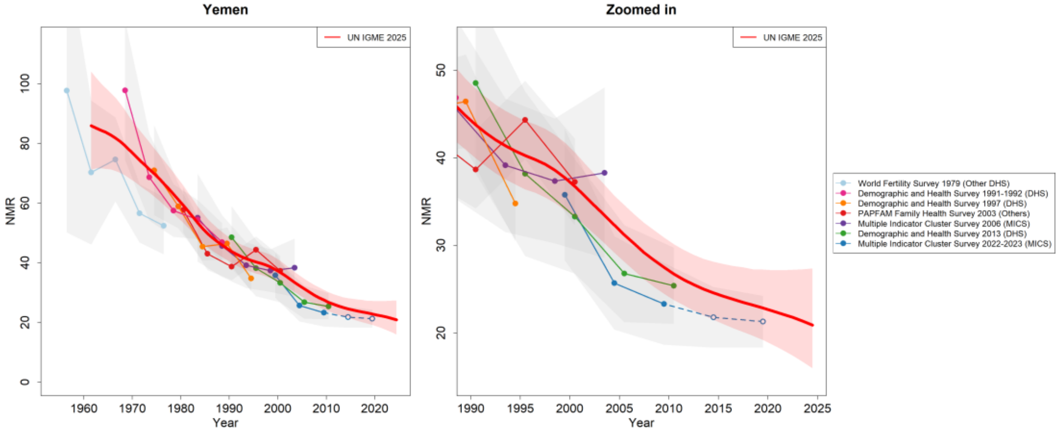

## Zambia (ZMB)

### Under-five mortality rate

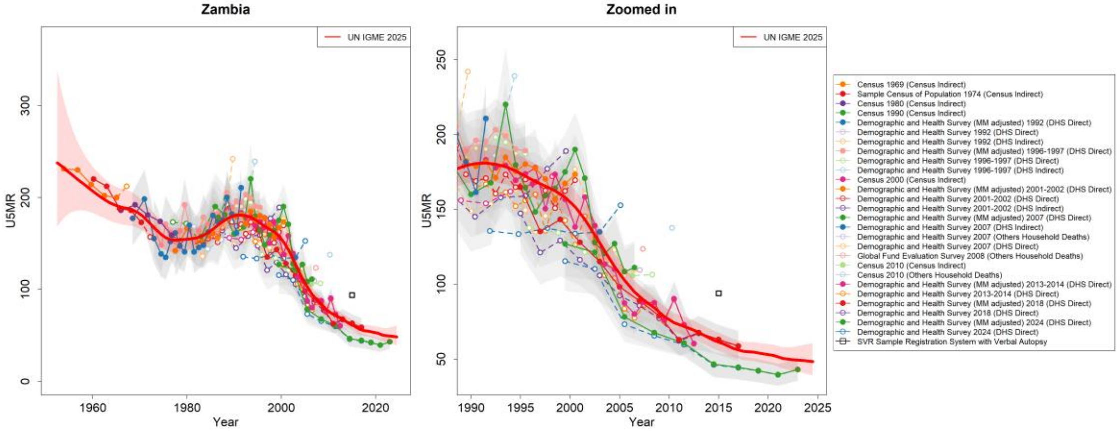


### Infant mortality rate

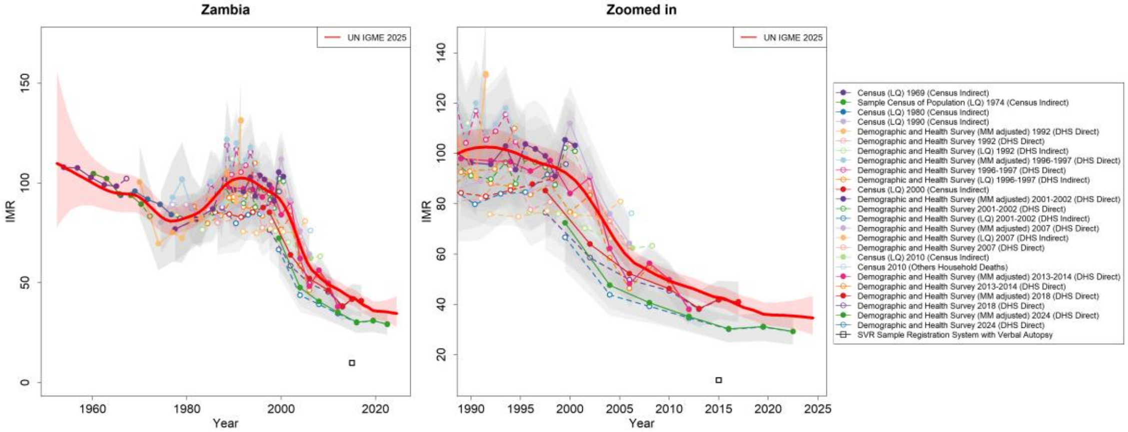


### Neonatal mortality rate

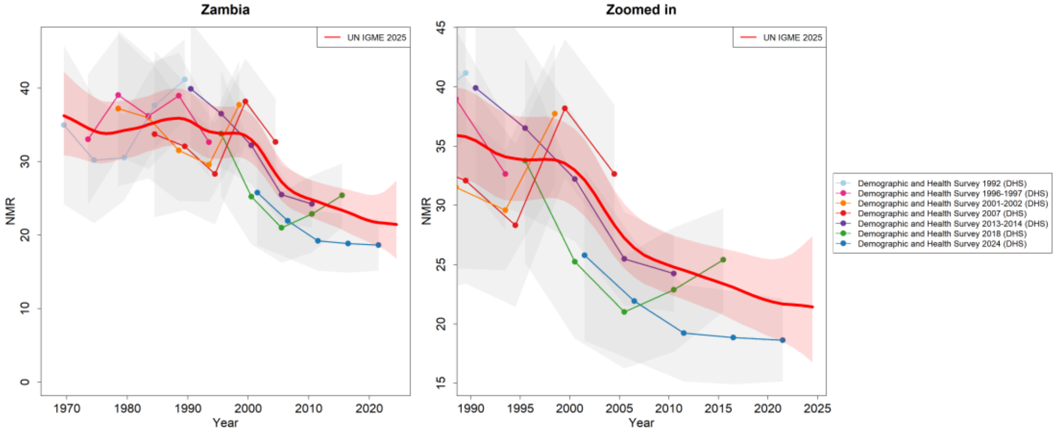

## Zimbabwe (ZWE)

### Under-five mortality rate

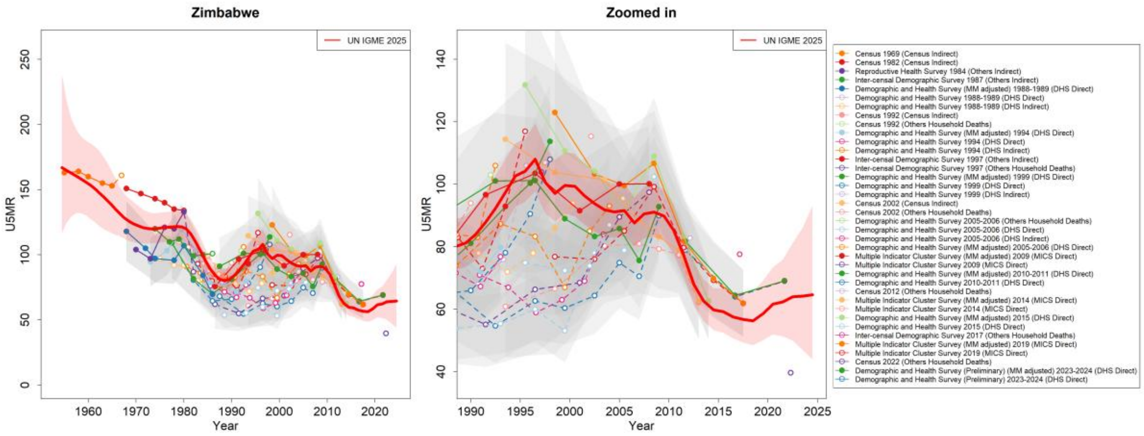


### Infant mortality rate

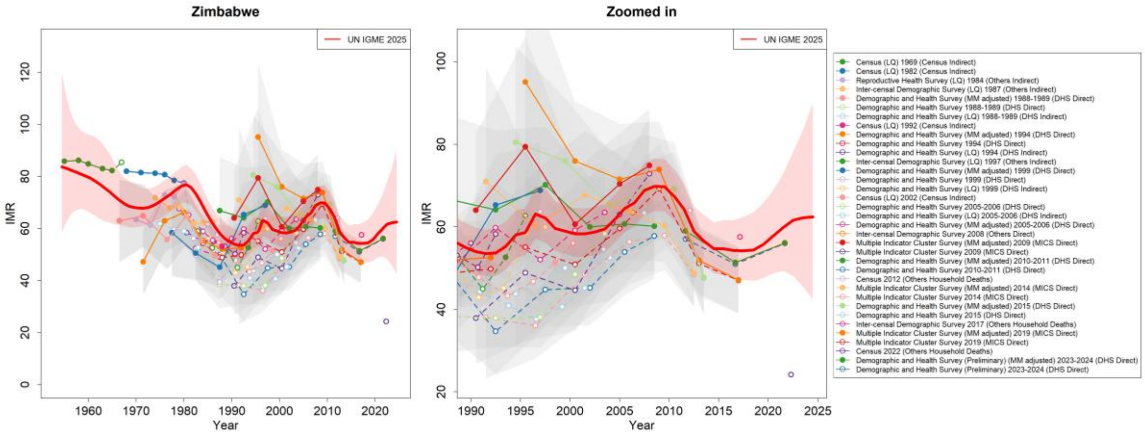


### Neonatal mortality rate

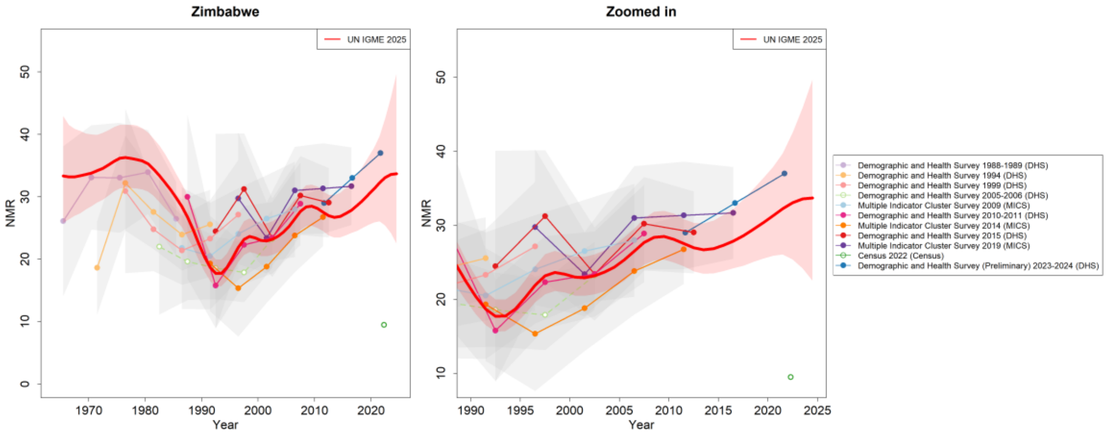

[1] Alkema, L., and New, J.R., ‘Global Estimation of Child Mortality Using a Bayesian B-spline Bias-reduction Method’, *The Annals of Applied Statistics*, vol. 8, no. 4, 2014, pp. 2122–2149.

[2] United Nations Children’s Fund, *MICS Tools*, UNICEF, New York, 2018.

[3] Croft, T., et al., et al. 2023. Guide to DHS Statistics. Rockville, Maryland, USA: ICF, 2023.

[4] Hill, K., ‘Child Mortality’, Ch. 15 in *Tools for Demographic Estimation*, edited by T. A. Moultrie, et al., International Union for the Scientific Study of Population, Paris, 2013.

[5] Pedersen, J., and J. Liu, ‘Child Mortality Estimation: Appropriate time periods for child mortality estimates from full birth histories’, *PLoS Medicine*, vol. 9, no. 8, 2012.

[6] Silva, R., ‘Child Mortality Estimation: Consistency of under-five mortality rate estimates using full birth histories and summary birth histories’, *PLoS Medicine*, vol. 9, no. 8, 2012.

[7] Walker, N., K. Hill and F. Zhao, ‘Child Mortality Estimation: Methods used to adjust for bias due to AIDS in estimating trends in under-five mortality’, *PLoS Medicine*, vol. 9, no. 8, 2012.

[8] Johnson P., N. Mizoguchi and A. Pantazis, ‘Improved Method for Adjusting for Bias due to HIV Mortality in Estimates of Child Mortality’, paper prepared for the Population Association of America Annual Meeting, Washington, D.C, 22–25 April 2020,

[9] United Nations Inter-Agency Group for Child Mortality Estimation, *Levels and Trends in Child Mortality: Report 2023*, UNICEF, New York, 2024.

[10] UN IGME, Child Mortality Estimates. <childmortality.org>.

[11] Hill, K., et al. ‘Child Mortality Estimation: Accelerated Progress in Reducing Global Child Mortality, 1990–2010’ *PLoS Medicine*, vol. 9, no. 8, 2012.

[12] Guillot M, Gerland P, Pelletier F, Saabneh A. Child Mortality Estimation: A Global Overview of Infant and Child Mortality Age Patterns in Light of New Empirical Data. Plos Medicine. 2012;9(8).

[13] Verhulst A., Romero Prieto J., Alam N., Eilerts H., Gerland P., Katz J., Lankoande B., Liu L., Pison G., Reniers G., Subedi S., Villavicencio F., and Guillot M. Divergent age patterns of under-5 mortality in south Asia and sub-Saharan Africa: a modelling study. The Lancet Global Health. 2022;10(11).

[14] Guillot M., Romero Prieto J., Verhulst A., Gerland P. Modeling age patterns of under-5 mortality: Results from a log-quadratic model applied to high-quality vital registration data. Demography. 2022;59(1).

[15] Joint United Nations Programme on HIV/AIDS (UNAIDS), 1990–2024 HIV and AIDS estimates, 2025.

[16] Alexander, M., and A. Leontine, ‘Global Estimation of Neonatal Mortality Using a Bayesian Hierarchical Splines Regression Model’, *Demographic Research*, vol. 38, 2018, pp. 335–372.

[17] Centre for Research on the Epidemiology of Disasters, EM-DAT: The International Disaster Database.

[18] Uppsala Conflict Data Program (UCDP) at the department of Peace and Conflict Research, Uppsala University.

[19] Lacina, B., and N.P. Gleditsch, ‘Monitoring Trends in Global Combat: A new dataset of battle deaths’, *European Journal of Population*, vol. 21, pp. 145–166, 2005.)

[20] Armed Conflict Location & Event Data Project (ACLED).

[21] Center for Systemic Peace/Integrated Network for Societal Conflict Research (INSCR) datasets.)

[22] Mathers C., et al., ‘Age-sex Patterns of Crisis Deaths: Towards a more standard mortality estimation approach’, Working paper, United Nations Children’s Fund, New York, 2023.

[23] United Nations Department of Economic and Social Affairs Population Division, *World Population Prospects 2024*, United Nations, New York, 2024.
